# A Customized Human Mitochondrial DNA Database (hMITO DB v1.0) for Rapid Sequence Analysis, Haplotyping and Geo-Mapping

**DOI:** 10.3390/ijms241713505

**Published:** 2023-08-31

**Authors:** Jane Shen-Gunther, Rutger S. Gunther, Hong Cai, Yufeng Wang

**Affiliations:** 1 Gynecologic Oncology & Clinical Investigation, Department of Clinical Investigation, Brooke Army Medical Center, Fort Sam Houston, San Antonio, TX 78234, USA; 2 Nuclear Medicine & Molecular Imaging, Department of Radiology, Brooke Army Medical Center, Fort Sam Houston, San Antonio, TX 78234, USA; rutger.s.gunther.mil@health.mil; 3 Department of Molecular Microbiology and Immunology, University of Texas at San Antonio, San Antonio, TX 78249, USA; hong.cai@utsa.edu; 4 South Texas Center for Emerging Infectious Diseases, University of Texas at San Antonio, San Antonio, TX 78249, USA

**Keywords:** bioinformatics, hypervariable region, mitochondrial DNA, mitochondrial genomics, mitochondrial haplogroup, molecular anthropology, next generation sequencing, oncogenetics, phylogeography

## Abstract

The field of mitochondrial genomics has advanced rapidly and has revolutionized disciplines such as molecular anthropology, population genetics, and medical genetics/oncogenetics. However, mtDNA next-generation sequencing (NGS) analysis for matrilineal haplotyping and phylogeographic inference remains hindered by the lack of a consolidated mitogenome database and an efficient bioinformatics pipeline. To address this, we developed a customized human mitogenome database (hMITO DB) embedded in a CLC Genomics workflow for read mapping, variant analysis, haplotyping, and geo-mapping. The database was constructed from 4286 mitogenomes. The macro-haplogroup (A to Z) distribution and representative phylogenetic tree were found to be consistent with published literature. The hMITO DB automated workflow was tested using mtDNA-NGS sequences derived from Pap smears and cervical cancer cell lines. The auto-generated read mapping, variants track, and table of haplotypes and geo-origins were completed in 15 min for 47 samples. The mtDNA workflow proved to be a rapid, efficient, and accurate means of sequence analysis for translational mitogenomics.

## 1. Introduction

“Mitochondria” were named by Carl Benda in 1898 to describe the threadlike granules found within the cytoplasm of eukaryotic cells [1,2]. Today, mitochondria are recognized as dynamic, ubiquitous organelles involved in various biological functions, including ATP synthesis, calcium signaling, metabolism, and apoptosis [3,4]. Mitochondrial DNA was first discovered in chick embryos by electron microscopy in 1963 [5]. However, it was not until 1981 that the first complete sequence of human mitochondrial DNA (mtDNA) was published and established as the mtDNA Cambridge Reference Sequence (CRS) [6,7]. Since then, the field of mitochondrial genomics has advanced rapidly and revolutionized matrilineal genetics, mitochondrial pathologies, and oncogenetics, providing insights into human evolution, population genetics, and disease mechanisms [7,8,9,10,11,12]. 

Mitochondria presumably originated as ancestral bacteria that were engulfed and integrated within host cells over 1.5 billion years ago [13]. This widely accepted “Endosymbiotic Theory” is supported by the presence of DNA (mitogenome) and a distinct RNA translation system within the eukaryotic cell [12,13,14,15]. The human mitogenome is a 16.6-kilobase (kb), double-stranded, circular DNA that is separate from the nuclear genome and matrilineally inherited [6,16,17]. It contains 37 genes, including 13 protein-coding genes, 22 transfer RNA (tRNA) genes, and two ribosomal RNA (rRNA) genes [6,16,17]. The non-coding region, or control region (CR), plays a crucial role in mitochondrial DNA replication and transcription. The hypervariable regions (HV-I, HV-II, and HV-III) within the CR have a higher mutation rate than coding regions [18,19,20]. Consequently, the discriminative power of HV-polymorphisms has been exploited for maternal lineage tracing, population studies, and forensics [18,19,20]. 

In 1987, Cann et al. published their groundbreaking discovery on lineage tracing of modern human mtDNA that led to a single tribe or “mother” in Africa, colloquially named, “Mitochondrial Eve” [21]. Torroni et al. later used the terms “haplotypes” and “haplogroups” (haplotype clusters or lineages), in particular, “A” through “D,” for the defining mtDNA polymorphisms of Indigenous tribes who peopled the Americas [22]. Today, next-generation sequencing (NGS) has revolutionized the field of mtDNA haplotyping. NGS enables rapid and cost-effective sequencing of the mitogenome, allowing for the identification of single-nucleotide polymorphisms (SNP), insertions, deletions, and structural variants, providing a comprehensive view of mtDNA diversity [18,19,20]. Over the last decade, the number of bioinformatics tools developed for mtDNA haplotyping has also increased [23]. García-Olivares et al. evaluated 11 software programs all using the preeminent PhyloTree_mt_ (online compendium with >5400 haplotypes) for classification [23,24]. The majority were exceptional, web-based tools using FASTA files as input [23]. However, the primary drawback for users of these online programs is the disjointed steps, i.e., uploading FASTA files, downloading haplotyping results, and concatenating results to sample information. Furthermore, geographic origins associated with the haplogroups are not readily accessible to infer phylogeography and race/ethnicity. To consolidate and streamline these time-consuming steps, we developed and tested a customized mitogenome database embedded within an automated workflow for sequence analysis, read mapping, variant analysis, haplotyping, and geo-mapping in CLC Genomics Workbench [25]. Our findings demonstrate that the streamlined workflow is a rapid and accurate means of mtDNA sequence analysis to advance human mitogenomics for molecular anthropology, population genetics, and medical genetics.

## 2. Results

### 2.1. hMITO DB Haplogroup Distribution and Geographic Origins

**Figure 1:**
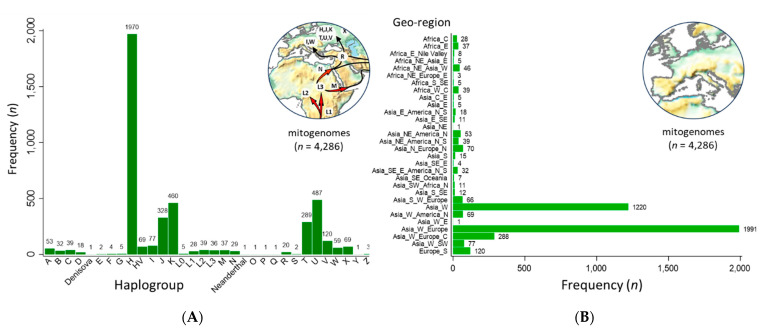
Distribution of haplogroups and geo-regions of the customized mitochondrial database. (**A**) The haplogroup distribution of the database is asymmetric with five predominant groups (H, J, K, T, and U). To complete the full spectrum of macro-haplogroups (A through Z), the Behar et al. dataset (*n* = 4265) was supplemented with rare mitogenomes (haplogroups O, P, Q, S, and Y) from Indigenous peoples living in remote regions of the world, i.e., Oceania and Siberia [30,31,32,33]; (**B**) Europe and West Asia dominated the geographic origins, which corresponds with the haplogroup distribution.

The hMITO DB was composed of 4286 mitogenomes. The macro-haplogroups identified by MITOMASTER and/or Haplogrep 3 revealed five predominant haplogroups (H, J, K, T, and U) (Figure 1A) [26,27,28,29]. Since the majority of mitogenomes (*n* = 4265) were contributed by Behar et al., this dataset exhibited a non-parametric distribution with underrepresentation of Indigenous peoples living in remote and isolated regions of the world [30]. The hMITO DB was supplemented with mitogenomes from haplogroups O, P, Q, S, and Y from Indigenous peoples of Oceania and Siberia to achieve the full spectrum of macro-haplogroups (“A” through “Z”) [31,32,33]. Like the haplogroup distribution, the geographic origin was dominated by Europe and West Asia with underrepresentation from other continental regions (Figure 1B). The biased distribution of haplogroup and geo-origin reflected the biased frequency of 59,389 human mitogenomes deposited in GenBank [27]. 

#### Phylogenetic Tree of Representative Human Mitogenomes

**Figure 2:**
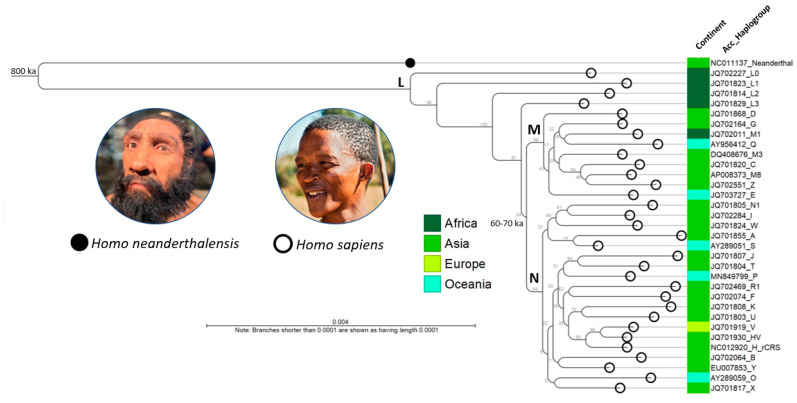
Phylogenetic tree of representative human mitogenomes from the customized database. The NJ tree was constructed after alignment of the mitogenomes of the Homo neanderthalensis RefSeq, revised Cambridge Reference Sequence (rCRS), and major haplogroups A through Z (*n* = 31). The metadata-decorated tree (color-coded by possible places of origin) reveals the phylogenetic, temporal, and spatial relationships between haplogroups. The tree is consistent with the out-of-Africa hypothesis and dispersal along the “Southern” (coastal) and “Northern” routes as shown by the respective macro-haplogroups, M and N (bolded), descending from L3 and branching out to distinct haplogroups across continents. Estimated times: Neanderthal-modern human divergence (~800 ka) and out-of-Africa migration (~60–70 ka) [34,35,36,37]. Photo credits (see Acknowledgments). Acc, NCBI accession number; ka, thousand years ago.

A neighbor joining (NJ) tree was constructed after alignment of the mitogenomes of the Homo neanderthalensis RefSeq, revised Cambridge Reference Sequence (rCRS), and major haplogroups A through Z (*n* = 31) from the hMITO DB (Figure 2) [6,34]. The rooted tree shows the Neanderthal-modern human divergence which transpired circa 800 ka (800,000 years ago) [34,35].

The metadata-decorated tree reveals the phylogenetic, temporal, and spatial relationships between haplogroups. The tree is consistent with the out-of-Africa hypothesis and dispersal along the “Southern” (coastal) and “Northern” routes as shown by the respective macro-haplogroups, M and N, descending from L3 and branching out to distinct haplogroups [30,36]. The out-of-Africa migration and ensuing human expansion dates back to ~60–70,000 years ago [30,37]. Computationally, the runtimes for the “create alignment” and “create tree” tools in CLC Genomics only took 28 min 20 s and 5 s, respectively, to visualize our ancestral past. 

### 2.2. Utility of hMITO DB for Sequence Analysis, Haplotyping, and Geo-Mapping of an mtDNA NGS Dataset from Cervical Cytology Samples and Cancer Cell Lines

#### 2.2.1. Read Mapping and Visualization of Mapped Tracks

**Figure 3:**
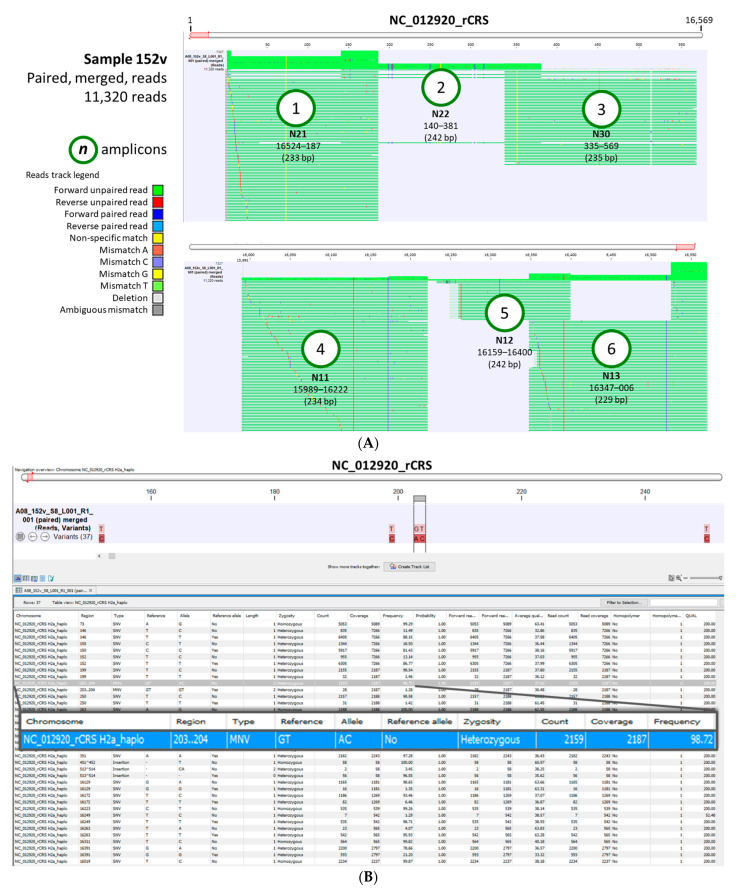
Mitochondrial reads and variant track views. (**A**) A representative, CR-sequenced mtDNA from clinical sample 152v was analyzed using the customized workflow. The read mapping displays the sequenced reads of 6 amplicons mapped against the rCRS reference genome. The overlapping amplicons, named “N*n*” according to Lee et al. [18], covered the entire control region. Flanking positions and amplicon sizes (bp) are shown below the names; (**B**) The variant track for sample 152v displays the locations of the variants (red brown) against the rCRS nucleotides (pink). The variant table lists the attributes of each variant. A row at positions 203–204 is magnified (blue) to show the sample’s “Allele” alongside the rCRS “Reference”, variant type, and frequency. For sample 152v, the heterozygous “AC” allele, typed as a multi-nucleotide variant (MNV), was identified in 98.72% of the reads.

The “Map Reads to Reference” tool within the CLC workflow generated two outputs: (1) a mapping report and (2) a reads track (Figure 3A). The mapping report summarized the total number of reads, mapped/unmapped reads, and intact/broken paired reads, as well as the matched/unmatched read length distribution per sample. A representative reads track shows 11,320 paired reads of sample 152v mapped onto the linearized rCRS genome (Figure 3A and Supplementary Video S1). Mapping fortuitously revealed lower but sufficient coverage (read depth > 30×), i.e., 58 and 566 for amplicons 3 and 5, respectively, presumably due to PCR or sequencing bias. According to Bentley et al., SNP discovery for homozygous and heterozygous positions plateaus at approximately 15× and 33× coverage, respectively [38]. However, to ensure adequate coverage, increasing DNA input and using MiSeq reagent kits v2 or v3 with greater sequencing output should suffice. Coverage bias or unevenness have been attributed to low-GC target regions, library preparation enzymes, library PCR amplification, cluster amplification, and sequencing [39].

The zoomed-in view at the nucleotide level allowed for comparison to the reference genome and detection of variants. The six amplicon start and end locations covered the entire mitochondrial control region (16,024 to 576 bp) and corresponded to the forward/reverse primer sequences. 

#### 2.2.2. Low Frequency Variant Track and Table

The “Low Frequency Variant Detection” tool within the hMITO DB workflow generated two outputs: (1) a variant track and (2) a variant table (Figure 3B). The variant track for representative sample 152v displays the location of the variants, while the variant table (Supplementary Table S1 and Video S1) lists the attributes of each variant. A row at positions 203–204 is magnified to show the sample’s “Allele” alongside the rCRS “Reference”. The percentage of variants (reads) is listed under “Frequency,” and is derived by dividing the read “Count” by the read “Coverage” × 100. For sample 152v, the heterozygous “AC” allele, typed as a multi-nucleotide variant (MNV), was identified in 98.72% of the reads. The “zygosity” column was helpful in deciphering homoplasty or heteroplasty (i.e., identical or non-identical copies of mtDNA). The variant tables for all 47 samples were compiled and presented as Supplementary Table S2. Data under the column headers, “Count”, “Coverage”, “Frequency”, and “Zygosity” for each variant nucleotide served as the determinants of polymorphism and/or heteroplasmy. An in-depth explanation of the variant metrics is beyond the scope of this article. The reader is referred to the CLC Microbial Genomics manual online for details [25]. For evolutionary analysis, haplotype classification is based on defining mtDNA polymorphisms, such as the variants shown in Figure 3B, that represent major branch points on the human phylogenetic tree [24]. 

### 2.3. mtDNA Haplotyping and Comparison to Self-Reported Race/Ethnicity 

The haplotypes and geo-origins of the five cervical carcinoma cell lines (controls) were consistent with the Genome Ancestry information (origin, % genome) published in Cellosaurus [40,41]. Specifically, the haplotypes and geo-origins by BLAST search against the hMITO DB were: HeLa (L3b1a; Africa_E), SiHa (X2b + 226; Asia_W_America_N), Ca Ski (HV; Asia_W), C33-A (U5a1b1a1; Asia_W_Europe_C), and DoTc2 (U2e1b1; Asia_S_W_ Europe). The respective Genome Ancestry information (origin, % genome) was: HeLa (African, 65%), SiHa (East Asian-North, 84%), Ca Ski (European-North, 68%), C33-A (European-North, 67%), and DoTc2 (European-North, 67%).

**Figure 4:**
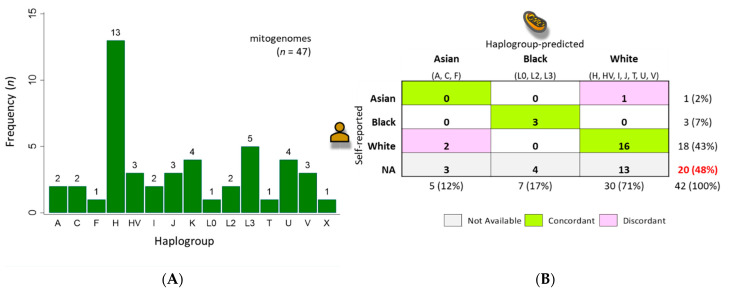
Haplogroup distribution of mtDNA-sequenced samples and comparison to self-reported race. (**A**) Mitochondrial haplotyping was achieved for all sequenced samples (*n* = 47); (**B**) For clinical samples (*n* = 42), comparison between self-reported and haplogroup-predicted race is shown as a cross tabulation. Self-reported race gleaned from the electronic health records (EHR) was missing (NA) for 20/42 (48%) samples. For the remaining samples (*n* = 22), inter-rater agreement was high 19/22 (86%) for Black and White races, whereas disagreement 3/22 (14%) occurred between Asian/White races. The geo-origins associated with the haplogroups (listed below race) were used to predict race. NA, not available.

The macro-haplogroup distribution of the clinical samples (*n* = 42) and cell lines (*n* = 5) after BLAST is shown in Figure 4A. The entire BLAST table is provided as Supplementary Table S3, which summarizes the statistical significance of the best sequence matches, mtDNA haplotype, and geo-origin. Haplotypes and predicted race were classified for all clinical samples. In contrast, self-reported race/ethnicity was missing in 20/42 (48%) of the electronic health records (EHR). The inter-rater agreement between Black and White races was high (86%), whereas inter-rater disagreement (14%) was found only between Asian/White races (Figure 4B).

### 2.4. FASTQ File Sizes and Workflow Runtimes

**Figure 5:**
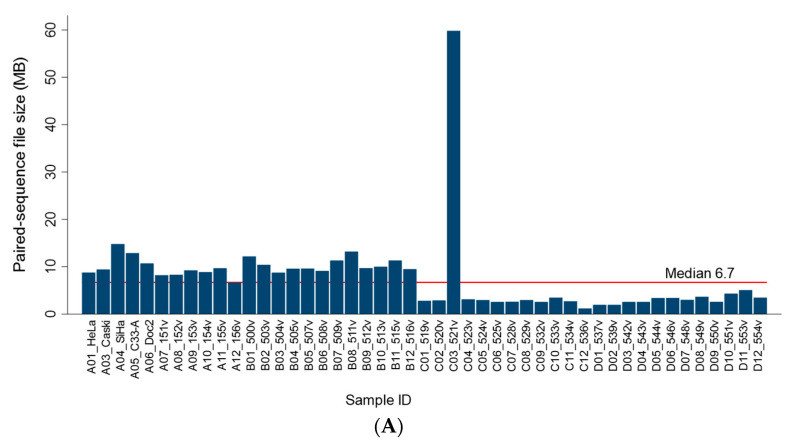

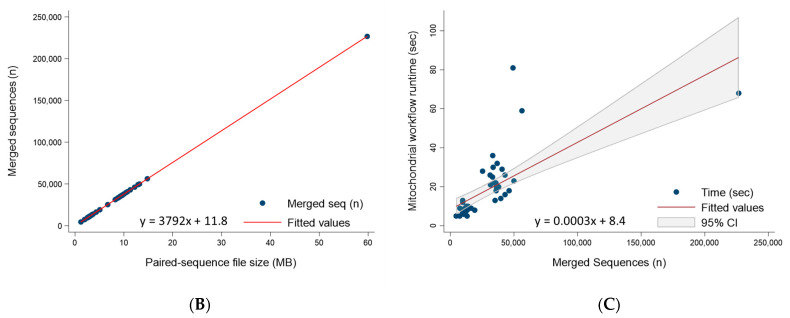
Correlation between NGS file size, reads, and mitochondrial workflow runtimes. (**A**) The sequencing file sizes of 47 samples ranged broadly between 1.21 and 59.8 MB, with a median of 6.7 MB. (**B**) The file size correlated perfectly with the number of merged sequences (R^2^ = 1). (**C**) The number of merged reads correlated positively with the mitochondrial CLC workflow runtimes in a linear relationship. The correlation was modest, with R^2^ = 0.50. The regression equations as shown may be utilized for estimation of workflow runtimes based on number of merged reads or file size.

The sequencing file size of the 47 samples ranged broadly between 1.21 and 59.8 MB, with a median of 6.7 MB (Figure 5A). The samples were sequenced on separate days as two separate batches (A01 to B12 and C01 to D12). The noticeably smaller file sizes for batch 2 (C01 to D12), except for C03, was presumed to be due to a lower quantity in the pooled DNA library submitted for sequencing.

Nonetheless, the reads per sample were sufficient for analysis and interpretation. The file size correlated perfectly with the number of merged sequences (Figure 5B), with a median of 25,354 (range, 4580 to 226,664) and an R^2^ = 1.0. The median runtime per sample for the mtDNA workflow was 13 s (ranging from 8 to 81 s). The cumulative runtime was 14.4 min for 47 samples. The timed results demonstrated exceptional efficiency and established benchmark metrics for future studies. A modest correlation between the number of merged sequences/samples and mtDNA workflow runtimes was found (R^2^ = 0.50) (Figure 5C). In practice, the regression equations derived from the correlation analysis may be utilized for estimating runtimes based on the number of merged reads/samples or file size (Figure 5B,C). Statistical analyses were performed using STATA/IC 17.0 (StataCorp LP, College Station, TX, USA).

## 3. Discussion

In this study, we developed a customized, human mitogenome database (hMITO DB) for use within CLC Genomics. A unified mtDNA database is crucial for advancing our understanding of population genetics, mitochondrial genetics, and human health. It would provide a centralized resource for cataloguing and organizing mtDNA genomes and polymorphisms to facilitate research on genetic variations and their functional consequences. Furthermore, associating mitochondrial polymorphisms with haplogroups and geographic origins will elucidate genetic predispositions to health or disease from an evolutionary and population perspective. 

After hMITO DB construction, we were able to visualize and examine the haplogroup distribution and associated places of origin of the NCBI-derived mitogenomes (*n* = 4286). The mass of the distribution was concentrated on five predominant haplogroups (H, J, K, T, and U) of European and West Asian origin. We supplemented the database with rare mitogenomes of Indigenous peoples to ensure representation from the entire spectrum of macro-haplogroups. Furthermore, representative mitogenomes from each macro-haplogroup were aligned for phylogenetic tree construction to confirm accuracy. The expanded, customized metadata of the hMITO DB enhanced visualization of the phylogenetic, temporal, and spatial relationships between haplogroups. The addition of the Neanderthal mitogenome and two crucial time points in human evolution, i.e., Neanderthal–modern human divergence (~800 ka) and out-of-Africa migration (~60–70 ka) bestowed a temporal perspective to human dispersal across continents [34,35,36,37]. 

The utility of the hMITO DB was demonstrated by using a Pap smear-derived mtDNA NGS dataset. By incorporating the curated database within the CLC workflow, we were able to process NGS data simply by inputting the FASTQ files, selecting the reference database, and setting the parameters. The read mapping and variant tracks with zoomable visualization provided effortless inspection of mapped regions and detected variants. A comprehensive analysis of each variant was provided in the auto-generated table of variants with columns of attributes. The mtDNA consensus sequences generated from the workflow were BLAST aligned very efficiently (5 s for 47 samples) for haplotyping and geo-mapping. More importantly, we were able to haplotype and predict the race for all clinical samples. In contrast, self-reported race/ethnicity was missing in 20/42 (48%) EHR records. The inter-rater agreement between Black and White races was high (86%), whereas inter-rater disagreement (14%) was found only between Asian/White races. The five samples with Asian haplogroups (A, C, and F) were self-reported as White (*n* = 2) or non-reported (*n* = 3). The geographic origins of haplogroups A and C are North and South America, and these haplogroups are most frequently found in American Indians (AI) and Indigenous peoples of Siberia, respectively [22,33]. The F haplogroup is common among the Lahu people of East Asia [42]. Our preliminary findings, although small in sample size, were consistent with a recent systematic review of 43 U.S.-based studies that showed EHRs frequently had “incomplete and/or inaccurate data on the race/ethnicity of patients” [43]. In contrast, disease registries or databases had highly accurate data for White and Black subjects, but relatively high rates of “misclassification and incomplete data for Hispanic/Latinx patients” [43]. The most misclassified populations were Asians, Pacific Islanders, and American Indian/Alaska Native (AI/AN) peoples [43]. Misclassification of race/ethnicity for Asians and American Indians has been problematic in cancer registries for years [44]. In fact, researchers have used the North American Association of Central Cancer Registries Asian/Pacific Islander Identification Algorithm (NAPIIA) to disaggregate and untangle Asian data to advance health disparity research [44]. Taken together, mitochondrial haplotyping is a highly relevant and valuable method of studying human health and disease, including demography, population genetics, social determinants of health (SDOH), genetic dispositions for disease, pharmacogenomics, and forensics [18,43,44,45,46,47]. 

The strength of this study is twofold. First, the customized workflow with the integrated mtDNA database unified numerous manual steps and automated time-consuming read mappings, variant detection, and consensus sequence generation. The incorporation of haplotypes and geographic origins in the hMITO DB eliminated manual searching, inputting, and outputting data to and from two indispensable online software programs, e.g., MITMAP and Haplogrep3, to deduce a result. The auto-generated tables, reports, and visualizations from the workflows abolished the shortcomings of manual data production to improve delivery speed, reduce costs, and minimize errors. Second, matrilineal haplotyping as a molecular tool will reduce the inherent problems of race/ethnicity classification based on self-reported data in EHRs, i.e., incomplete and/or inaccurate data [43]. The application of our streamlined mitogenomics approach will facilitate and improve data accuracy in studies of human health, disease, and beyond [48]. 

We acknowledge that our study has limitations. In the current version of the hMITO DB, we aimed to capture the entire spectrum of macro-haplogroups. However, each macro-haplogroup has descendants ranging from few to many sub-lineages, such as M1 through M52 of the M macro-haplogroup [24]. In fact, van Oven and Kayser’s PhyloTree*_mt_* Build 17 was based on 5400 mtDNA haplotypes [24]. To achieve a balance between the number of mitogenomes and workflow runtimes, we intend to expand our database with rare mitogenomes and avoid duplicating existing haplotypes in future versions of hMITO DB. As for the wet lab, alternative methods: (1) Illumina mtDNA kit (Illumina, San Diego, CA, USA) using two mitogenome-spanning amplicons for NGS and (2) IDT xGen Human mtDNA Hybridization Panel (IDT, Coralville, IA, USA) with distinct advantages for intact and degraded DNA, respectively, warrant performance testing. 

## 4. Materials and Methods

### 4.1. Construction and Content of Customized Reference Database

**Figure 6:**
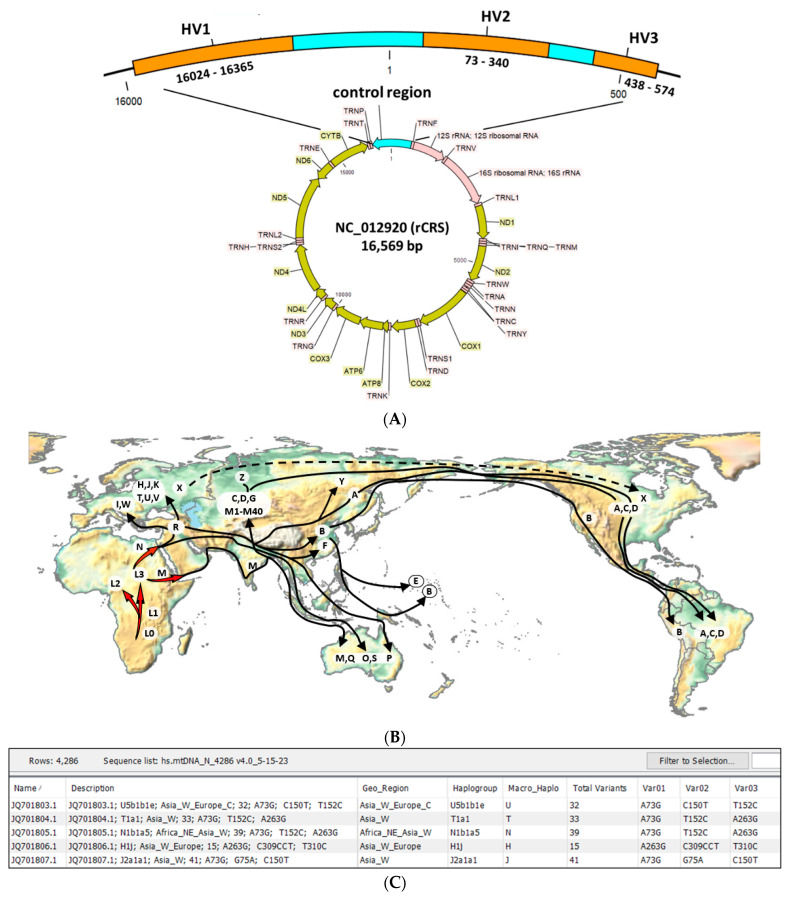
Customized human mitochondrial genome database constructed from mitogenomes, haplotypes, and places of origin. (**A**) Representative human mitochondrial genome with expanded control region and hypervariable regions (HV) used for haplotyping; (**B**) Map of places of origin and migration routes for macro-haplogroups A through Z adapted from [51,52]; (**C**) Table view of mitochondrial database in CLC (truncated) showing mitogenome attributes: name (accession number), description, geo-region, haplogroup, and nucleotide variants. See Supplementary Table S4 for full-length table.

A total of 4286 complete human mitochondrial genomes were downloaded to construct the customized reference database. The genomes included: (1) the rCRS RefSeq belonging to European haplogroup H2a2a1 (NCBI Genome accession no. NC_012920.1), (2) haplotyped mtDNA genome (*n* = 20) from the NCBI database (Supplementary Table S4, rows 4266 to 4286, excluding 4279), and (3) the Behar et al. dataset of mitogenomes (*n* = 4265) (NCBI PopSet accession nos. JQ701803 to JQ706067) [30]. The Behar et al. dataset in FASTA format is also available for batch download through PhyloTree (Rotterdam, The Netherlands) [24]. All mtDNA FASTA files were imported into CLC Genomics and customized as described below for use as an mtDNA genome and BLAST database. The annotated rCRS RefSeq mtDNA genome is shown in Figure 6A. 

The 4286 mtDNA FASTA files were subjected to haplotype classification and nucleotide variant determination using MITOMASTER (Philadelphia, PA, USA) [26,27]. The tabulated output included: (1) predicted haplotype, (2) total variants (*n*), and (3) variants by nucleotide position. For unclassifiable queries in MITOMASTER, Haplogrep 3 (3.2.1) (Innsbruck, AT, USA) was used for haplotype determination [28,29]. The Haplogrep parameter, “phylogenetic tree” was set at “rCRS PhyloTree 17.2” using the latest release and rCRS as the reference sequence. The parameter “distance function” was set at “Kulczynski (default)”, a weighted metric that returns the best hit for haplotype identification [29]. 

The presumed geographic origin of a particular macro-haplogroup was classified according to the seven-continent terminology (Africa, Asia, Europe, North America, South America, Australia, and Antarctica) (Figure 6B) [49]. For subregions of Africa, Asia, and Europe, the nomenclature of the United Nations geoscheme was used: (Africa-North, South, East, West, and Central; Asia-North, South, East, South-East, West, and Central; Europe-North, South, East, and West) [50]. For Australia, the United Nations term “Oceania”, inclusive of Australia, New Zealand, Melanesia, Micronesia, and Polynesia, was used as the collective place of origin [50]. For the database, the places-of-origin variable was named “Geo-region”. A map of macro-haplogroups and possible places of origin is shown in Figure 6B. The migration routes were adapted from Wallace et al. [51] and Vilar et al. for haplogroups B and E of the Chamorro people of the Marianas Islands [52]. The world map was created with Mathematica 13.2 (Wolfram Research, Champaign, IL, USA). 

Database customization involved incorporation of the haplogroup and geo-region data and creation of a new “Description” variable for each mtDNA genome file (Figure 6C). The “Description” was an author-defined, clinically relevant, concatenated seven-dimensional descriptor of each mtDNA file. Specifically, the seven dimensions are as follows: name (NCBI accession number), haplogroup, continent_subregion, total number of variants, Variant 1, Variant 2, and Variant 3. Only the first 3 variants identified by MITOMASTER and/or Haplogrep 3 are listed in the “Description” column due to space constraints. For completeness, the metadata of the database lists all identified variants (up to 81) for each mtDNA genome in separate columns. As an example, the “Description” of the homo sapiens isolate Aus23 of an Australian Aborigine (Accession no. AY289059) [31] was annotated as “AY289059; O; Oceania; 44; C44CC; A73G; A263G.” Finally, all customized data created and curated as a metadata file was incorporated into the attribute fields of the sequence file for downstream applications, as described in Section 2.2. See Supplementary Table S4 and Video S1 for the full-length hMITO DB (*n* = 4286) with select attribute columns.

### 4.2. mtDNA Sequence Analysis and BLAST Workflows

**Figure 7:**
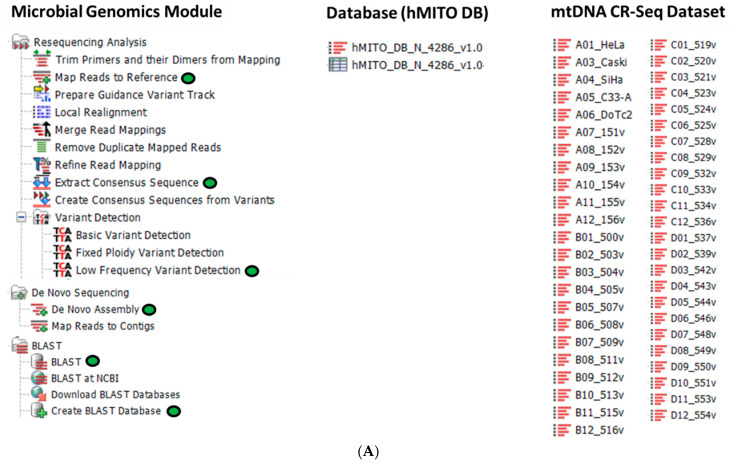

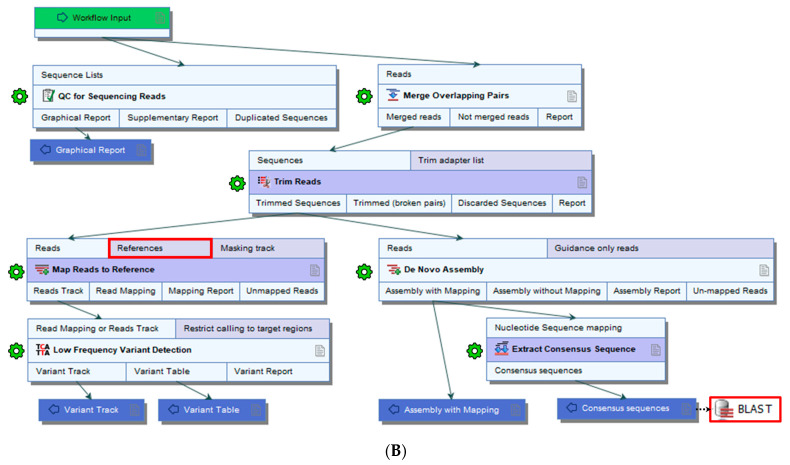
Bioinformatics methods. (**A**) CLC Microbial Genomics Module, mitochondrial database (hMITO DB), and dataset used for sequence analysis, haplotyping, and geo-mapping. The CLC tools used herein are designated by the green oval icons; (**B**) The custom CLC workflow for mtDNA sequence analysis consisted of seven major steps (green gears) with embedded hMITO DB (red rectangle) for read mapping and variant detection. The extracted consensus sequences were used for downstream BLAST search against the hMITO DB.

CLC Genomics Workbench Premium 23.0.4, inclusive of the CLC Microbial Genomics Module (CLC MGM) (Redwood City, CA, USA), was installed on an HP notebook computer (specifications: Windows 10 operating system, Intel i7–7500U dual-core processor @ 2.70 GHz and 8 GB RAM) for all analyses. The CLC system requirements are provided online [53]. A custom CLC workflow for mitochondrial DNA sequence analysis was constructed from seven primary CLC MGM tools and connected for automated data processing and output (Figure 7A,B). 

The analysis consisted of the following steps: (1) data importing and quality control (QC), (2) merging overlapping pairs, (3) trimming reads, (4) reads mapping to human mtDNA reference genomes, (5) low frequency variant detection, (6) de novo assembly, and (7) extracting consensus sequence(s) (Figure 7B). The embedded custom hMITO DB, as described in Section 4.1, was used for reads mapping, variant detection, and downstream BLAST search. The workflow generated genetic variant tracks and tables that are zoomable for inspection at the nucleotide level. The workflow also generated consensus sequences for BLAST query and identification of the most similar mitogenome, haplotype, and geographic origin. 

### 4.3. Cell Samples and mtDNA Control Region Sequencing

**Figure 8:**
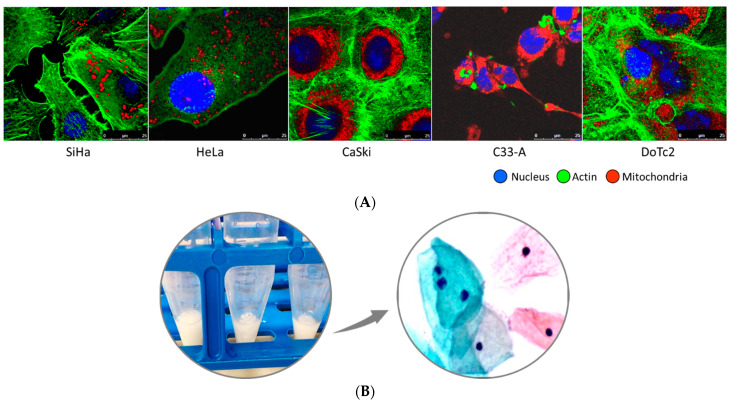
Representative images of cervical carcinoma cell lines and cervical cytology used in this study. (**A**) Five cervical carcinoma cell lines: SiHa, HeLa, Ca Ski, C33-A, and DoTc2 with distinguishing cytomorphologic features: nucleus (blue), nuclear-cytoplasmic ratio, actin cytoskeleton (green), and mitochondria (red). The dynamic nature of mitochondria regarding number, morphology, and distribution within the cytoplasm is demonstrated by two distinguishing patterns: a diffuse, dotted cytoplasmic pattern (SiHa, HeLa, and DoTc2) versus a dense peri-nuclear halo pattern (Ca Ski and C33-A). The cell lines were immunofluorescently labeled and imaged by confocal microscopy (X63 objective); (**B**) Representative images of residual cell pellets from liquid cervical cytology samples (**left**) and normal cellular morphology under light microscopy after Papanicolaou staining (ThinPrep, 50× magnification) (**right**).

Five cervical cancer cell lines (SiHA, HeLa, Ca Ski, C33-A, and DoTc2) acquired from the American Type Culture Collection (ATCC, Manassas, VA, USA) were cultured and extracted of genomic DNA (gDNA) for a previous study (Figure 8A) [54]. The stored gDNA was amplified using target-specific primers for mtDNA and sequenced to serve as controls. The haplogroup and geo-region results were verified against the documented genome ancestry of each cell line in Cellosaurus (Cell line encyclopedia) [40,41]. Cell culturing and imaging methods were described previously [54].

For clinical samples (*n* = 42), the stored gDNA (−80 C) extracted previously from liquid-based cervical cytology for another study (Figure 8B) [54] was used for mtDNA deep sequencing. Amplification of the entire mtDNA control region (CR) and sequencing were performed at Lucigen/LGC (Middleton, WI, USA). For mtDNA CR amplification, the “Midiplex primer sets I and II” designed by Lee et al. for forensic science were used [18]. Six fragments (N11, N13, N22) and (N12, N21, and N30) were generated by two multiplex PCR reactions, i.e., “Midiplex I and II,” using 3 primer sets each. The forward and reverse primer sequences of Lee et al. were comprised of a common Nextera forward (Rd1) or reverse (Rd2) read sequence on the 5′ end joined by the mt-DNA specific sequence on the 3′ end [18]. For this study, the NxSeq (LGC, Middleton, WI, USA) primers comprised of universal forward read sequence-(mtDNA-specific sequences)-3′ and universal reverse read sequence-(mtDNA-specific sequences)-3′ (listed in Supplementary Table S5) were used for the Midplex I and II PCR reactions per manufacturer’s instructions. The cycling protocol was as follows: activation [95 C × 11 min], 25 cycles [94 C × 24 s, 56 C × 60 s, 72 C × 30 s], final extension [72 C × 7 min], hold 4 C. The PCR products from Midiplex I and II were pooled for each sample (20 uL total) prior to the second PCR reaction for ligation of dual indices and platform-specific sequences [18]. The limited-cycle PCR protocol was as follows: activation [98 C × 30 s], five cycles [98 C × 10 s, 72 C × 75 s], final extension [72 C × 5 min], hold 4 C.

After bead clean-up, the DNA libraries were normalized to ensure a pooled DNA library concentration of 4 ng/uL with an average amplicon size of 374 bp. Paired-end sequencing using the MiSeq 2 × 150 kit v2 nano format (300-cycles) was performed on the MiSeq sequencer (Illumina, San Diego, CA, USA). The mtDNA CR-sequenced FASTQ dataset is shown in Figure 7A.

## 5. Conclusions

In conclusion, our customized human mitochondrial database (hMITO DB) embedded within a CLC automated workflow provided a rapid and accurate means of sequence analysis. The pipeline for mitogenome analysis, haplotyping, and phylogeography will facilitate discoveries and advancements in human mitogenomics.

## Data Availability

The data presented in this study are openly available in the NCBI Sequence Read Archive (SRA). Title: A Customized Human Mitochondrial DNA Database (hMITO DB v1.0) for Rapid Sequence Analysis, Haplotyping and Geo-Mapping. SRA Accession Number: SRP450940; BioProject: PRJNA997736; BioSample Accession Numbers: SAMN36688595 to 36688641. The hMITO DB v1.0 (.clc format) that supports the findings of this study is available as a supplementary file, Database S1.

## Supplementary Materials

The following are available online at https://www.mdpi.com/article/10.3390/ijms241713505/s1. Reference [55] is cited in the Table S3.

## Abbreviations

Acc, NCBI accession number; AI/AN, American Indian/Alaska Native; BLAST, Basic local alignment search tool; CLC MGM, CLC Microbial Genomics Module; CR, control region; EHR, electronic health record; GB, gigabyte; hMITO DB, human mitochondrial DNA database; HSIL, high-grade squamous intraepithelial lesion; HV, hypervariable region; ka, thousand years; kb, kilobase, MB, megabyte; MNV, multi-nucleotide variant; mtDNA, mitochondrial DNA; NCBI, National Center for Biotechnology Information; NILM, negative for intraepithelial lesion or malignancy; NGS, Next-generation sequencing; PopSet, population sequence database in GenBank; QC, quality control; rCRS, revised Cambridge Reference Sequence; RefSeq, reference sequence collection in GenBank.
