# Supplementary material for: A Customized Human Mitochondrial DNA Database (hMITO DB v1.0) for Rapid Sequence Analysis, Haplotyping and Geo-Mapping
Source: Int J Mol Sci. 2023 Aug 31;24(17):13505. doi: 10.3390/ijms241713505 (PMC10488239; doi:10.3390/ijms241713505)
Supplement: Supplementary file 1 [file ijms-24-13505-s001.zip › ijms-2550861-supplementary/Table S1.pdf]

Supplementary Table S1 Variant table[a] for sample 152v

Reference Position	Type	Length	Reference	Allele	Zygosity	Count	Coverage	Frequency	Average quality
73	SNV	1	A	G	Homozygous	5053	5089	99.29259186	63.41104294
146	SNV	1	T	C	Heterozygous	835	7266	11.49187999	32.85508982
150	SNV	1	C	T	Heterozygous	1344	7266	18.49710983	36.4389881
152	SNV	1	T	C	Heterozygous	955	7266	13.14340765	37.03141361
199	SNV	1	T	C	Heterozygous	2155	2187	98.53680841	37.80464037
203	MNV	2	GT	AC	Heterozygous	2159	2187	98.71970736	37.61195881
250	SNV	1	T	C	Heterozygous	2157	2188	98.58318099	63.36439499
263	SNV	1	A	G	Homozygous	2188	2188	100	62.54981718
303	Insertion	1	-	C	Heterozygous	1883	2188	86.06032907	35.72545717
303	Insertion	2	-	CC	Heterozygous	136	2188	6.215722121	35.02180358
311	Insertion	1	-	C	Heterozygous	2147	2187	98.17101052	37.38751747
351	SNV	1	A	G	Heterozygous	59	2243	2.630405707	36.01694915
452	Insertion	1	-	T	Homozygous	58	58	100	60.96551724
514	Insertion	2	-	CA	Heterozygous	2	58	3.448275862	38.25
16129	SNV	1	G	A	Heterozygous	1165	1181	98.64521592	63.66008584
16172	SNV	1	T	C	Heterozygous	1186	1269	93.45941686	37.06661046
16223	SNV	1	C	T	Homozygous	535	539	99.25788497	38.13831776
16249	SNV	1	T	C	Heterozygous	7	542	1.291512915	38.57142857
16263	SNV	1	T	A	Heterozygous	23	565	4.07079646	63.82608696
16311	SNV	1	T	C	Homozygous	564	565	99.82300885	40.17553191
16391	SNV	1	G	A	Heterozygous	2200	2797	78.65570254	36.57272727
16519	SNV	1	T	C	Homozygous	2234	2237	99.86589182	38.18487019

SNV, single nucleotide variant; MNV, multi-nucleotide variant

[a]The variant table was auto-generated by the CLC Microbial Genomics workflow. Definitions of variant metrics are provided in the CLC online manual [25].
