# Supplementary material for: A Customized Human Mitochondrial DNA Database (hMITO DB v1.0) for Rapid Sequence Analysis, Haplotyping and Geo-Mapping
Source: Int J Mol Sci. 2023 Aug 31;24(17):13505. doi: 10.3390/ijms241713505 (PMC10488239; doi:10.3390/ijms241713505)
Supplement: Supplementary file 1 [file ijms-24-13505-s001.zip › ijms-2550861-supplementary/Table S2.pdf]

Supplementary Table S2 A01_HeLa Variant Table[a]

Reference mitogenome	Region	Type	Reference	Allele	Reference allele	Length	Linkage	Zygosity
NC_012920_rCRS H2a_haplo	73	SNV	A	G	No	1		Homozygous
NC_012920_rCRS H2a_haplo	146	SNV	T	C	No	1		Heterozygous
NC_012920_rCRS H2a_haplo	146	SNV	T	T	Yes	1		Heterozygous
NC_012920_rCRS H2a_haplo	150	SNV	C	T	No	1		Heterozygous
NC_012920_rCRS H2a_haplo	150	SNV	C	C	Yes	1		Heterozygous
NC_012920_rCRS H2a_haplo	152	SNV	T	C	No	1		Heterozygous
NC_012920_rCRS H2a_haplo	152	SNV	T	T	Yes	1		Heterozygous
NC_012920_rCRS H2a_haplo	263	SNV	A	G	No	1		Homozygous
NC_012920_rCRS H2a_haplo	302^303	Insertion	-	C	No	1		Heterozygous
NC_012920_rCRS H2a_haplo	302^303	Insertion	-	CC	No	2		Heterozygous
NC_012920_rCRS H2a_haplo	302^303	Insertion	-	-	Yes	0		Heterozygous
NC_012920_rCRS H2a_haplo	310^311	Insertion	-	C	No	1		Homozygous
NC_012920_rCRS H2a_haplo	351	SNV	A	G	No	1		Heterozygous
NC_012920_rCRS H2a_haplo	351	SNV	A	A	Yes	1		Heterozygous
NC_012920_rCRS H2a_haplo	514..515	Deletion	CA	-	No	2		Homozygous
NC_012920_rCRS H2a_haplo	16124	SNV	T	C	No	1		Homozygous
NC_012920_rCRS H2a_haplo	16223	SNV	C	T	No	1		Homozygous
NC_012920_rCRS H2a_haplo	16249	SNV	T	C	No	1		Heterozygous
NC_012920_rCRS H2a_haplo	16249	SNV	T	T	Yes	1		Heterozygous
NC_012920_rCRS H2a_haplo	16263	SNV	T	A	No	1		Heterozygous
NC_012920_rCRS H2a_haplo	16263	SNV	T	T	Yes	1		Heterozygous
NC_012920_rCRS H2a_haplo	16278	SNV	C	T	No	1		Homozygous
NC_012920_rCRS H2a_haplo	16362	SNV	T	C	No	1		Heterozygous
NC_012920_rCRS H2a_haplo	16362	SNV	T	T	Yes	1		Heterozygous
NC_012920_rCRS H2a_haplo	16519	SNV	T	C	No	1		Homozygous

Supplementary Table S2 A01_HeLa Variant Table

**Count**	**Coverage**	**Frequency**	Probability	Forward read count	Reverse read count	Forward read coverage	Reverse read coverage	Forward/reverse balance	Average quality
5382	5389	99.87010577	1	5382	0	5389	0	0	63.44760312
447	6596	6.776834445	1	447	0	6596	0	0	33.08501119
6137	6596	93.04123711	1	6137	0	6596	0	0	37.73798273
730	6597	11.0656359	1	730	0	6597	0	0	36.27123288
5865	6597	88.90404729	1	5865	0	6597	0	0	38.25319693
509	6597	7.715628316	1	509	0	6597	0	0	36.90962672
6082	6597	92.19342125	1	6082	0	6597	0	0	38.0881289
1210	1210	100	1	1210	0	1210	0	0	62.45041322
1095	1210	90.49586777	1	1095	0	1210	0	0	35.37716895
47	1210	3.884297521	1	47	0	1210	0	0	34.34042553
66	1210	5.454545455	1	66	0	1210	0	0	29.71212121
1197	1209	99.00744417	1	1197	0	1209	0	0	37.37844612
23	1232	1.866883117	1	23	0	1232	0	0	36.56521739
1209	1232	98.13311688	1	1209	0	1232	0	0	36.14309347
22	23	95.65217391	1	22	0	23	0	0	35
1822	1823	99.94514536	1	1822	0	1823	0	0	63.66300768
413	414	99.75845411	1	413	0	414	0	0	37.63922518
6	420	1.428571429	0.995888926	6	0	420	0	0	29.83333333
414	420	98.57142857	1	414	0	420	0	0	38.69806763
41	463	8.855291577	1	41	0	463	0	0	62.17073171
422	463	91.14470842	1	422	0	463	0	0	62.67772512
463	463	100	1	463	0	463	0	0	63.5399568
461	4028	11.4448858	1	461	0	4028	0	0	39.77223427
3567	4028	88.5551142	1	3567	0	4028	0	0	36.79394449
3563	3567	99.88786095	1	3563	0	3567	0	0	38.20965479

Supplementary Table S2 A01_HeLa Variant Table

Read count	Read coverage	# unique start positions	# unique end positions	BaseQRankSum	Read position test probability	Read direction test probability	Homopolymer	Homopolymer length	QUAL
5382	5389	7	7		1	1	No	1	200
447	6596	3	4	-32.23	0	1	No	1	200
6137	6596	9	10		4.07452E-14	1	No	1	200
730	6597	3	4	-36.12	0	1	No	1	200
5865	6597	9	11		0	1	No	1	200
509	6597	3	3	-24.4	0	1	No	1	200
6082	6597	9	12		0	1	No	1	200
1210	1210	2	5		1	1	No	1	200
1095	1210	3	5	12.09	0.966164122	1	Yes	7	200
47	1210	1	1	5.76	0.84899659	1	Yes	7	200
66	1210	1	2		0.821664033	1	Yes	7	200
1197	1209	2	4	3.91	1	1	Yes	5	200
23	1232	3	2	1.34	0	1	No	1	200
1209	1232	3	5		7.89018E-05	1	No	1	200
22	23	2	1		1	1	No	1	200
1822	1823	3	6		1	1	No	1	200
413	414	11	3		1	1	No	1	200
6	420	4	1	-3.05	0	1	No	1	23.86044664
414	420	12	3		0.408921771	1	No	1	200
41	463	1	1	-0.58	0	1	No	1	200
422	463	14	3		3.84708E-06	1	No	1	200
463	463	15	3		1	1	No	1	200
461	4028	16	4	7.17	0	1	No	1	200
3567	4028	5	7		0	1	No	1	200
3563	3567	4	6		1	1	No	1	200

Supplementary Table S2 A03_CaSki Variant Table

Reference mitogenome	Region	Type	Reference	Allele	Reference allele	Length	Linkage	Zygosity
NC_012920_rCRS	146	SNV	T	C	No	1		Heterozygous
NC_012920_rCRS	146	SNV	T	T	Yes	1		Heterozygous
NC_012920_rCRS	150	SNV	C	T	No	1		Heterozygous
NC_012920_rCRS	150	SNV	C	C	Yes	1		Heterozygous
NC_012920_rCRS	152	SNV	T	C	No	1		Heterozygous
NC_012920_rCRS	152	SNV	T	T	Yes	1		Heterozygous
NC_012920_rCRS	263	SNV	A	G	No	1		Homozygous
NC_012920_rCRS	302^303	Insertion	-	C	No	1		Heterozygous
NC_012920_rCRS	302^303	Insertion	-	-	Yes	0		Heterozygous
NC_012920_rCRS	310	SNV	T	C	No	1		Heterozygous
NC_012920_rCRS	310	SNV	T	T	Yes	1		Heterozygous
NC_012920_rCRS	310^311	Insertion	-	C	No	1		Heterozygous
NC_012920_rCRS	310^311	Insertion	-	-	Yes	0		Heterozygous
NC_012920_rCRS	314^315	Insertion	-	A	No	1		Heterozygous
NC_012920_rCRS	314^315	Insertion	-	-	Yes	0		Heterozygous
NC_012920_rCRS	351	SNV	A	G	No	1		Heterozygous
NC_012920_rCRS	351	SNV	A	A	Yes	1		Heterozygous
NC_012920_rCRS	16140	SNV	T	A	No	1		Heterozygous
NC_012920_rCRS	16140	SNV	T	T	Yes	1		Heterozygous
NC_012920_rCRS	16143	SNV	T	C	No	1		Heterozygous
NC_012920_rCRS	16143	SNV	T	G	No	1		Heterozygous
NC_012920_rCRS	16143	SNV	T	T	Yes	1		Heterozygous
NC_012920_rCRS	16146	SNV	A	G	No	1		Heterozygous
NC_012920_rCRS	16146	SNV	A	A	Yes	1		Heterozygous
NC_012920_rCRS	16149	SNV	A	C	No	1		Heterozygous
NC_012920_rCRS	16149	SNV	A	A	Yes	1		Heterozygous
NC_012920_rCRS	16152	SNV	T	C	No	1		Heterozygous
NC_012920_rCRS	16152..16153	MNV	TG	TG	Yes	2		Heterozygous
NC_012920_rCRS	16153	SNV	G	T	No	1		Heterozygous
NC_012920_rCRS	16161	SNV	T	A	No	1		Heterozygous
NC_012920_rCRS	16161	SNV	T	T	Yes	1		Heterozygous
NC_012920_rCRS	16172	SNV	T	A	No	1		Heterozygous
NC_012920_rCRS	16172	SNV	T	C	No	1		Heterozygous
NC_012920_rCRS	16172	SNV	T	T	Yes	1		Heterozygous
NC_012920_rCRS	16175	SNV	A	C	No	1		Heterozygous
NC_012920_rCRS	16175	SNV	A	A	Yes	1		Heterozygous
NC_012920_rCRS	16178	SNV	T	A	No	1		Heterozygous
NC_012920_rCRS	16178	SNV	T	T	Yes	1		Heterozygous
NC_012920_rCRS	16180	Deletion	A	-	No	1		Heterozygous
NC_012920_rCRS	16180	SNV	A	A	Yes	1		Heterozygous
NC_012920_rCRS	16182..16183	MNV	AA	CC	No	2		Heterozygous
NC_012920_rCRS	16182..16183	MNV	AA	AA	Yes	2		Heterozygous
NC_012920_rCRS	16183	SNV	A	C	No	1		Heterozygous
NC_012920_rCRS	16183	Replacement	A	CC	No	2		Heterozygous
NC_012920_rCRS	16183	Replacement	A	CCC	No	3		Heterozygous
NC_012920_rCRS	16189	SNV	T	C	No	1		Heterozygous
NC_012920_rCRS	16189	Deletion	T	-	No	1		Heterozygous
NC_012920_rCRS	16194	SNV	A	C	No	1		Heterozygous
NC_012920_rCRS	16194	SNV	A	A	Yes	1		Heterozygous
NC_012920_rCRS	16196	SNV	G	C	No	1		Heterozygous
NC_012920_rCRS	16196	SNV	G	G	Yes	1		Heterozygous
NC_012920_rCRS	16200	SNV	A	C	No	1		Heterozygous

NC_012920_rCRS	16200	SNV	A	A	Yes	1		Heterozygous
NC_012920_rCRS	16204	SNV	G	C	No	1		Heterozygous
NC_012920_rCRS	16204	SNV	G	G	Yes	1		Heterozygous
NC_012920_rCRS	16208..16209	MNV	GT	AC	No	2		Heterozygous
NC_012920_rCRS	16208..16209	MNV	GT	CC	No	2		Heterozygous
NC_012920_rCRS	16208..16209	MNV	GT	GT	Yes	2		Heterozygous
NC_012920_rCRS	16209	SNV	T	C	No	1		Heterozygous
NC_012920_rCRS	16213	SNV	G	C	No	1		Heterozygous
NC_012920_rCRS	16213	SNV	G	G	Yes	1		Heterozygous
NC_012920_rCRS	16224	SNV	T	C	No	1		Heterozygous
NC_012920_rCRS	16224	Deletion	T	-	No	1		Heterozygous
NC_012920_rCRS	16224	SNV	T	T	Yes	1		Heterozygous
NC_012920_rCRS	16231	SNV	T	C	No	1		Heterozygous
NC_012920_rCRS	16231	SNV	T	T	Yes	1		Heterozygous
NC_012920_rCRS	16233	SNV	A	C	No	1		Heterozygous
NC_012920_rCRS	16233	SNV	A	A	Yes	1		Heterozygous
NC_012920_rCRS	16235	SNV	A	C	No	1		Heterozygous
NC_012920_rCRS	16235	SNV	A	A	Yes	1		Heterozygous
NC_012920_rCRS	16238	SNV	T	C	No	1		Heterozygous
NC_012920_rCRS	16238	SNV	T	T	Yes	1		Heterozygous
NC_012920_rCRS	16243	SNV	T	A	No	1		Heterozygous
NC_012920_rCRS	16243	SNV	T	C	No	1		Heterozygous
NC_012920_rCRS	16243..16244	MNV	TG	CC	No	2		Heterozygous
NC_012920_rCRS	16243..16245	MNV	TGC	TGC	Yes	3		Heterozygous
NC_012920_rCRS	16244	SNV	G	C	No	1		Heterozygous
NC_012920_rCRS	16244	SNV	G	T	No	1		Heterozygous
NC_012920_rCRS	16244..16245	MNV	GC	CA	No	2		Heterozygous
NC_012920_rCRS	16249	SNV	T	C	No	1		Heterozygous
NC_012920_rCRS	16249	SNV	T	T	Yes	1		Heterozygous
NC_012920_rCRS	16263	SNV	T	A	No	1		Heterozygous
NC_012920_rCRS	16263	SNV	T	T	Yes	1		Heterozygous
NC_012920_rCRS	16519	SNV	T	C	No	1		Homozygous

**Count**	**Coverage**	**Frequency**	Probability	Forward read count	Reverse read count	Forward read coverage	Reverse read coverage	Forward/reverse balance	Average quality
925	9834	9.40614196	1	925	0	9834	0	0	32.9275676
8884	9834	90.339638	1	8884	0	9834	0	0	37.6517335
1506	9838	15.3079894	1	1506	0	9838	0	0	36.4435591
8326	9838	84.6310226	1	8326	0	9838	0	0	38.1429258
1041	9839	10.5803435	1	1041	0	9839	0	0	36.6887608
8789	9839	89.3281838	1	8789	0	9839	0	0	38.0209353
2414	2414	100	1	2414	0	2414	0	0	63.1922121
72	2414	2.98260149	1	72	0	2414	0	0	33.6527778
2342	2414	97.0173985	1	2342	0	2414	0	0	30.9842015
47	2414	1.94697597	1	47	0	2414	0	0	31.3829787
2367	2414	98.053024	1	2367	0	2414	0	0	26.7536967
2331	2414	96.5617233	1	2331	0	2414	0	0	37.533251
82	2414	3.3968517	1	82	0	2414	0	0	30.5487122
30	2413	1.24326564	1	30	0	2413	0	0	36.8333333
2383	2413	98.7567344	1	2383	0	2413	0	0	36.5241292
59	2470	2.38866397	1	59	0	2470	0	0	36.779661
2410	2470	97.5708502	1	2410	0	2470	0	0	36.4941909
94	762	12.335958	1	94	0	762	0	0	14.5957447
658	762	86.351706	1	658	0	762	0	0	20.5638298
73	762	9.58005249	1	73	0	762	0	0	14.2191781
26	762	3.41207349	1	26	0	762	0	0	16.3461538
660	762	86.6141732	1	660	0	762	0	0	21.180303
25	762	3.2808399	0.99976256	25	0	762	0	0	12.24
718	762	94.2257218	1	718	0	762	0	0	24.9192201
193	762	25.328084	1	193	0	762	0	0	13.4041451
565	762	74.1469816	1	565	0	762	0	0	21.7557522
60	762	7.87401575	0.99999988	60	0	762	0	0	13.2500285
674	762	88.4514436	1	674	0	762	0	0	24.7200363
20	762	2.62467192	0.9999979	20	0	762	0	0	14.597868
167	821	20.3410475	1	167	0	821	0	0	13.4311377
647	821	78.8063337	1	647	0	821	0	0	21.5177743
85	827	10.2781137	1	85	0	827	0	0	15.8117647
35	827	4.23216445	1	35	0	827	0	0	21.8571429
688	827	83.1922612	1	688	0	827	0	0	22.4258721
120	1028	11.6731518	0.99999281	120	0	1028	0	0	14.0333333
902	1028	87.7431907	1	902	0	1028	0	0	26.5687361
148	1028	14.3968872	1	148	0	1028	0	0	14.7297297
855	1028	83.1712062	1	855	0	1028	0	0	24.4409357
122	1029	11.856171	1	122	0	1029	0	0	33.942623
898	1029	87.2691934	1	898	0	1029	0	0	32.0467706
18	1029	1.74927114	1	18	0	1029	0	0	22.5655268
156	1029	15.1603499	1	156	0	1029	0	0	31.864278
369	1029	35.8600583	1	369	0	1029	0	0	31.2162895
411	1029	39.941691	1	411	0	1029	0	0	30.5680652
74	1029	7.19144801	1	74	0	1029	0	0	32.7128378
1014	1034	98.065764	1	1014	0	1034	0	0	36.5128205
15	1034	1.45067698	1	15	0	1034	0	0	35.7333333
93	1126	8.25932504	1	93	0	1126	0	0	18.3225806
1029	1126	91.3854352	1	1029	0	1126	0	0	35.2896016
60	1126	5.3285968	1	60	0	1126	0	0	17.1833333
1059	1126	94.0497336	1	1059	0	1126	0	0	34.680831
68	1126	6.03907638	1	68	0	1126	0	0	18.7794118

1047	1126	92.9840142	1	1047	0	1126	0	0	35.5367717
49	1126	4.35168739	1	49	0	1126	0	0	19.244898
1036	1126	92.0071048	1	1036	0	1126	0	0	34.5511583
34	1126	3.01953819	1	34	0	1126	0	0	18.8397746
25	1126	2.22024867	1	25	0	1126	0	0	18.2311678
769	1126	68.294849	1	769	0	1126	0	0	36.8055219
290	1126	25.7548845	1	290	0	1126	0	0	28.4420788
36	1125	3.2	0.9928463	36	0	1125	0	0	15.3333333
1063	1125	94.4888889	1	1063	0	1125	0	0	32.7826905
108	368	29.3478261	1	108	0	368	0	0	16
4	368	1.08695652	0.99864225	4	0	368	0	0	18
252	368	68.4782609	1	252	0	368	0	0	28.3293651
23	368	6.25	0.99999858	23	0	368	0	0	17.4782609
334	368	90.7608696	1	334	0	368	0	0	27.0718563
43	372	11.5591398	0.99999934	43	0	372	0	0	16.7674419
329	372	88.4408602	1	329	0	372	0	0	26.9969605
48	372	12.9032258	0.999854	48	0	372	0	0	15.1666667
323	372	86.827957	1	323	0	372	0	0	26.371517
44	372	11.827957	0.99999807	44	0	372	0	0	14.0227273
315	372	84.6774194	1	315	0	372	0	0	26.5111111
6	372	1.61290323	0.48047384	6	0	372	0	0	15.999205
14	372	3.76344086	0.99978637	14	0	372	0	0	17.809658
9	372	2.41935484	0.99999854	9	0	372	0	0	17.711286
272	372	73.1182796	1	272	0	372	0	0	29.9021499
49	372	13.172043	1	49	0	372	0	0	18.177752
10	372	2.68817204	0.99999327	10	0	372	0	0	19.4983718
6	372	1.61290323	0.91946908	6	0	372	0	0	16.3694029
83	374	22.1925134	1	83	0	374	0	0	15.8915663
287	374	76.7379679	1	287	0	374	0	0	27.8675958
48	423	11.3475177	1	48	0	423	0	0	63.8125
363	423	85.8156028	1	363	0	423	0	0	40.7961433
2561	2562	99.960968	1	2561	0	2562	0	0	38.3557204

Read count	Read coverage	# unique start positions	# unique end positions	BaseQRankSum	Read position test probability	Read direction test probability	Homopolymer	Homopolymer length	QUAL
925	9834	3	8	-45.5	0	1	No	1	200
8884	9834	9	11		0	1	No	1	200
1506	9838	4	7	-48.41	0	1	No	1	200
8326	9838	9	13		0	1	No	1	200
1041	9839	6	9	-32.68	0	1	No	1	200
8789	9839	8	9		0	1	No	1	200
2414	2414	5	9		1	1	No	1	200
72	2414	1	1	4.1	0.97247682	1	Yes	7	200
2342	2414	5	9		1	1	Yes	7	200
47	2414	1	3	5.48	6.7682E-06	1	No	1	200
2367	2414	5	9		0.55493004	1	Yes	7	200
2331	2414	5	8	15.16	0.17769997	1	Yes	5	200
82	2414	1	4		1.4758E-10	1	Yes	5	200
30	2413	1	1	-2.27	0.91352072	1	No	1	200
2383	2413	5	8		1	1	No	1	200
59	2470	2	5	1.36	0	1	No	1	200
2410	2470	6	8		6.7067E-12	1	No	1	200
94	762	1	2	-5.11	0.73455026	1	No	1	200
658	762	2	4		0.93967753	1	No	1	200
73	762	1	1	-9.59	0.76518834	1	No	1	200
26	762	1	1	-2.1	0.85828877	1	No	1	96.9896997
660	762	2	4		0.94139229	1	No	1	200
25	762	1	2	-6.54	0.00016525	1	No	1	36.2444173
718	762	2	3		0.34175244	1	No	1	200
193	762	2	2	-18.27	0.62600794	1	No	1	200
565	762	2	3		0.85371792	1	No	1	200
60	762	1	4	-13.41	0.00764048	1	No	1	69.1080163
674	762	2	2		0.36053402	1	No	1	200
20	762	1	1	-5.64	0.87414284	1	No	1	56.7807033
167	821	2	2	-16.2	0.00068694	1	No	1	200
647	821	4	5		0.27957538	1	No	1	200
85	827	1	2	-8.8	0.01093232	1	No	1	200
35	827	2	3	-2.63	1.4166E-07	1	No	1	200
688	827	5	5		1	1	No	1	200
120	1028	2	1	-13.34	1.4701E-09	1	No	1	51.4312013
902	1028	8	7		0.5265702	1	No	1	200
148	1028	2	3	-14.99	2.581E-11	1	No	1	200
855	1028	8	5		0.1354326	1	No	1	200
122	1029	5	2	3.95	0.1580416	1	Yes	4	200
898	1029	8	7		0.98734862	1	Yes	4	200
18	1029	1	1	-9.47	0.05140115	1	No	1	200
156	1029	6	2		0.02651074	1	No	1	200
369	1029	5	4	-4.92	0.66734674	1	Yes	5	200
411	1029	6	7	-6.32	0.7067957	1	No	1	200
74	1029	3	3	-3.1	7.1788E-05	1	Yes	5	200
1014	1034	12	7		1	1	No	1	200
15	1034	2	2		0.23840618	1	Yes	5	200
93	1126	6	3	-14.21	0	1	No	1	200
1029	1126	14	7		0.05797929	1	No	1	200
60	1126	3	1	-10.99	0	1	No	1	200
1059	1126	15	7		0.39127033	1	No	1	200
68	1126	5	3	-11.92	0	1	No	1	200

1047	1126	15	7		0.24063712	1	No	1	200
49	1126	3	2	-9.68	0	1	No	1	200
1036	1126	15	6		0.11557587	1	No	1	200
34	1126	4	1	-13.37	0	1	No	1	200
25	1126	2	2	-11.54	1.1102E-16	1	No	1	200
769	1126	4	5		0	1	No	1	200
290	1126	13	4	-12.81	0	1	No	1	200
36	1125	2	2	-9.56	0	1	No	1	21.4546945
1063	1125	15	7		0.21449158	1	No	1	200
108	368	3	3	-12.91	1	1	No	1	200
4	368	3	1	-2.67	1	1	Yes	3	28.671806
252	368	13	1		1	1	No	1	200
23	368	3	1	-6.11	1	1	No	1	58.4905945
334	368	14	3		1	1	No	1	200
43	372	4	2	-7.66	0.51159227	1	No	1	61.7789733
329	372	15	3		0.90653121	1	No	1	200
48	372	6	1	-9.15	0.48789844	1	No	1	38.3563495
323	372	15	3		0.88586419	1	No	1	200
44	372	4	1	-10.56	0.50670182	1	No	1	57.1412777
315	372	14	3		0.85780194	1	No	1	200
6	372	1	1	-6.79	0.80332512	1	No	1	2.84392577
14	372	4	1	-9.7	0.70366187	1	No	1	36.7034162
9	372	2	1	-7.3	0.7638586	1	No	1	58.3585542
272	372	14	2		1	1	No	1	200
49	372	4	2	-14.64	0.48480022	1	No	1	200
10	372	4	2	-5.94	0.74866564	1	No	1	51.7225605
6	372	2	1	-5.77	0.81070252	1	No	1	10.9403735
83	374	7	1	-11.89	0.25640015	1	No	1	200
287	374	13	3		0.58161106	1	No	1	200
48	423	1	3	7.21	0	1	No	1	200
363	423	16	2		1.7911E-07	1	No	1	200
2561	2562	7	4		1	1	No	1	200

Reference mitogenome	Region	Type	Reference	Allele	Reference allele	Length	Linkage	Zygosity
NC_012920_rCRS H2a_haplo	73	SNV	A	G	No	1		Homozygous
NC_012920_rCRS H2a_haplo	146	SNV	T	C	No	1		Heterozygous
NC_012920_rCRS H2a_haplo	146	SNV	T	T	Yes	1		Heterozygous
NC_012920_rCRS H2a_haplo	150	SNV	C	T	No	1		Heterozygous
NC_012920_rCRS H2a_haplo	150	SNV	C	C	Yes	1		Heterozygous
NC_012920_rCRS H2a_haplo	152	SNV	T	C	No	1		Heterozygous
NC_012920_rCRS H2a_haplo	152	SNV	T	T	Yes	1		Heterozygous
NC_012920_rCRS H2a_haplo	252	SNV	T	C	No	1		Homozygous
NC_012920_rCRS H2a_haplo	263	SNV	A	G	No	1		Homozygous
NC_012920_rCRS H2a_haplo	302^303	Insertion	-	C	No	1		Heterozygous
NC_012920_rCRS H2a_haplo	302^303	Insertion	-	CC	No	2		Heterozygous
NC_012920_rCRS H2a_haplo	302^303	Insertion	-	-	Yes	0		Heterozygous
NC_012920_rCRS H2a_haplo	310^311	Insertion	-	C	No	1		Heterozygous
NC_012920_rCRS H2a_haplo	310^311	Insertion	-	-	Yes	0		Heterozygous
NC_012920_rCRS H2a_haplo	351	SNV	A	G	No	1		Heterozygous
NC_012920_rCRS H2a_haplo	351	SNV	A	A	Yes	1		Heterozygous
NC_012920_rCRS H2a_haplo	375	SNV	C	A	No	1		Heterozygous
NC_012920_rCRS H2a_haplo	375	SNV	C	C	Yes	1		Heterozygous
NC_012920_rCRS H2a_haplo	456	SNV	C	T	No	1		Homozygous
NC_012920_rCRS H2a_haplo	489	SNV	T	C	No	1		Homozygous
NC_012920_rCRS H2a_haplo	16140	SNV	T	A	No	1		Heterozygous
NC_012920_rCRS H2a_haplo	16140	SNV	T	T	Yes	1		Heterozygous
NC_012920_rCRS H2a_haplo	16143	SNV	T	C	No	1		Heterozygous
NC_012920_rCRS H2a_haplo	16143	SNV	T	T	Yes	1		Heterozygous
NC_012920_rCRS H2a_haplo	16146	SNV	A	G	No	1		Heterozygous
NC_012920_rCRS H2a_haplo	16146	SNV	A	A	Yes	1		Heterozygous
NC_012920_rCRS H2a_haplo	16149	SNV	A	C	No	1		Heterozygous
NC_012920_rCRS H2a_haplo	16149	SNV	A	A	Yes	1		Heterozygous
NC_012920_rCRS H2a_haplo	6152..16153	MNV	TG	CT	No	2		Heterozygous
NC_012920_rCRS H2a_haplo	6152..16153	MNV	TG	TG	Yes	2		Heterozygous
NC_012920_rCRS H2a_haplo	16153	SNV	G	T	No	1		Heterozygous
NC_012920_rCRS H2a_haplo	16161	SNV	T	A	No	1		Heterozygous
NC_012920_rCRS H2a_haplo	16161	SNV	T	T	Yes	1		Heterozygous
NC_012920_rCRS H2a_haplo	16172	SNV	T	A	No	1		Heterozygous
NC_012920_rCRS H2a_haplo	16172	SNV	T	C	No	1		Heterozygous
NC_012920_rCRS H2a_haplo	16172	SNV	T	T	Yes	1		Heterozygous
NC_012920_rCRS H2a_haplo	16175	SNV	A	C	No	1		Heterozygous
NC_012920_rCRS H2a_haplo	16175	SNV	A	A	Yes	1		Heterozygous
NC_012920_rCRS H2a_haplo	16178	SNV	T	A	No	1		Heterozygous
NC_012920_rCRS H2a_haplo	16178	SNV	T	T	Yes	1		Heterozygous
NC_012920_rCRS H2a_haplo	16180	Deletion	A	-	No	1		Heterozygous
NC_012920_rCRS H2a_haplo	16180	SNV	A	A	Yes	1		Heterozygous
NC_012920_rCRS H2a_haplo	6182..16183	MNV	AA	CC	No	2		Heterozygous
NC_012920_rCRS H2a_haplo	6182..16183	MNV	AA	AA	Yes	2		Heterozygous
NC_012920_rCRS H2a_haplo	16183	SNV	A	C	No	1		Heterozygous
NC_012920_rCRS H2a_haplo	16183	Replacement	A	CC	No	2		Heterozygous
NC_012920_rCRS H2a_haplo	16183	Replacement	A	CCC	No	3		Heterozygous
NC_012920_rCRS H2a_haplo	16189	SNV	T	C	No	1		Heterozygous
NC_012920_rCRS H2a_haplo	16189	Deletion	T	-	No	1		Heterozygous
NC_012920_rCRS H2a_haplo	16194	SNV	A	C	No	1		Heterozygous
NC_012920_rCRS H2a_haplo	16194	SNV	A	A	Yes	1		Heterozygous
NC_012920_rCRS H2a_haplo	16196	SNV	G	C	No	1		Heterozygous

NC_012920_rCRS H2a_haplo	16196	SNV	G	G	Yes	1		Heterozygous
NC_012920_rCRS H2a_haplo	16200	SNV	A	C	No	1		Heterozygous
NC_012920_rCRS H2a_haplo	16200	SNV	A	A	Yes	1		Heterozygous
NC_012920_rCRS H2a_haplo	16204	SNV	G	C	No	1		Heterozygous
NC_012920_rCRS H2a_haplo	16204	SNV	G	G	Yes	1		Heterozygous
NC_012920_rCRS H2a_haplo	16209	SNV	T	A	No	1		Heterozygous
NC_012920_rCRS H2a_haplo	16209	SNV	T	T	Yes	1		Heterozygous
NC_012920_rCRS H2a_haplo	16213	SNV	G	C	No	1		Heterozygous
NC_012920_rCRS H2a_haplo	16213	SNV	G	G	Yes	1		Heterozygous
NC_012920_rCRS H2a_haplo	16217	SNV	T	A	No	1		Heterozygous
NC_012920_rCRS H2a_haplo	16217	SNV	T	T	Yes	1		Heterozygous
NC_012920_rCRS H2a_haplo	16223	SNV	C	T	No	1		Heterozygous
NC_012920_rCRS H2a_haplo	6223..16224	MNV	CT	TC	No	2		Heterozygous
NC_012920_rCRS H2a_haplo	6223..16224	MNV	CT	CT	Yes	2		Heterozygous
NC_012920_rCRS H2a_haplo	16229	SNV	T	A	No	1		Heterozygous
NC_012920_rCRS H2a_haplo	16229	SNV	T	C	No	1		Heterozygous
NC_012920_rCRS H2a_haplo	6229..16231	MNV	TAT	TAT	Yes	3		Heterozygous
NC_012920_rCRS H2a_haplo	16230	SNV	A	T	No	1		Heterozygous
NC_012920_rCRS H2a_haplo	6230..16231	MNV	AT	TC	No	2		Heterozygous
NC_012920_rCRS H2a_haplo	16231	SNV	T	A	No	1		Heterozygous
NC_012920_rCRS H2a_haplo	16231	SNV	T	C	No	1		Heterozygous
NC_012920_rCRS H2a_haplo	16233	SNV	A	C	No	1		Heterozygous
NC_012920_rCRS H2a_haplo	16233	SNV	A	A	Yes	1		Heterozygous
NC_012920_rCRS H2a_haplo	16238	SNV	T	C	No	1		Heterozygous
NC_012920_rCRS H2a_haplo	16238	SNV	T	T	Yes	1		Heterozygous
NC_012920_rCRS H2a_haplo	16243	SNV	T	C	No	1		Heterozygous
NC_012920_rCRS H2a_haplo	6243..16244	MNV	TG	AC	No	2		Heterozygous
NC_012920_rCRS H2a_haplo	6243..16244	MNV	TG	CC	No	2		Heterozygous
NC_012920_rCRS H2a_haplo	6243..16244	MNV	TG	TG	Yes	2		Heterozygous
NC_012920_rCRS H2a_haplo	16244	SNV	G	C	No	1		Heterozygous
NC_012920_rCRS H2a_haplo	16244	SNV	G	T	No	1		Heterozygous
NC_012920_rCRS H2a_haplo	16249	SNV	T	C	No	1		Heterozygous
NC_012920_rCRS H2a_haplo	16249	SNV	T	T	Yes	1		Heterozygous
NC_012920_rCRS H2a_haplo	16256	SNV	C	A	No	1		Heterozygous
NC_012920_rCRS H2a_haplo	16256	SNV	C	C	Yes	1		Heterozygous
NC_012920_rCRS H2a_haplo	16263	SNV	T	A	No	1		Heterozygous
NC_012920_rCRS H2a_haplo	16263	SNV	T	T	Yes	1		Heterozygous
NC_012920_rCRS H2a_haplo	16291	SNV	C	G	No	1		Heterozygous
NC_012920_rCRS H2a_haplo	16291	SNV	C	C	Yes	1		Heterozygous
NC_012920_rCRS H2a_haplo	16362	SNV	T	C	No	1		Heterozygous
NC_012920_rCRS H2a_haplo	16362	SNV	T	T	Yes	1		Heterozygous
NC_012920_rCRS H2a_haplo	16519	SNV	T	C	No	1		Homozygous

**Count**	**Coverage**	**Frequency**	Probability	Forward read count	Reverse read count	Forward read coverage	Reverse read coverage	Forward/reverse balance	Average quality
11690	11700	99.91452991	1	11690	0	11700	0	0	63.4710864
13098	15611	83.90237653	1	13098	0	15611	0	0	37.32722553
2492	15611	15.96310294	1	2492	0	15611	0	0	36.40048154
14010	15615	89.72142171	1	14010	0	15615	0	0	37.60913633
1594	15615	10.20813321	1	1594	0	15615	0	0	36.20263488
13311	15615	85.24495677	1	13311	0	15615	0	0	38.29036136
2287	15615	14.64617355	1	2287	0	15615	0	0	37.30301705
3921	3925	99.89808917	1	3921	0	3925	0	0	63.52665136
3924	3925	99.97452229	1	3924	0	3925	0	0	63.09480122
3609	3923	91.99592149	1	3609	0	3923	0	0	35.96065392
67	3923	1.707876625	1	67	0	3923	0	0	34.31343284
243	3923	6.194239103	1	243	0	3923	0	0	31.2962963
3867	3923	98.57252103	1	3867	0	3923	0	0	37.69640548
55	3923	1.401988274	1	55	0	3923	0	0	31.30909091
73	3997	1.826369777	1	73	0	3997	0	0	36.98630137
3919	3997	98.0485364	1	3919	0	3997	0	0	36.77468742
77	3992	1.928857715	1	77	0	3992	0	0	37.22077922
3911	3992	97.97094188	1	3911	0	3992	0	0	35.91306571
76	76	100	1	76	0	76	0	0	62.98684211
76	76	100	1	76	0	76	0	0	36.17105263
112	1046	10.70745698	1	112	0	1046	0	0	14.625
927	1046	88.62332696	1	927	0	1046	0	0	21.99568501
92	1046	8.79541109	1	92	0	1046	0	0	14.2173913
938	1046	89.6749522	1	938	0	1046	0	0	22.7761194
29	1046	2.772466539	0.999998267	29	0	1046	0	0	12.31034483
989	1046	94.55066922	1	989	0	1046	0	0	26.04246714
281	1046	26.86424474	1	281	0	1046	0	0	13.65124555
763	1046	72.94455067	1	763	0	1046	0	0	22.04456094
12	1046	1.147227533	1	12	0	1046	0	0	14.8125012
1000	1046	95.60229446	1	1000	0	1046	0	0	24.89902696
28	1046	2.676864245	0.999998797	28	0	1046	0	0	14.93938436
210	1107	18.9701897	1	210	0	1107	0	0	12.90952381
893	1107	80.66847335	1	893	0	1107	0	0	21.22508399
148	1110	13.33333333	1	148	0	1110	0	0	14.55405405
30	1110	2.702702703	0.999999807	30	0	1110	0	0	19.3
922	1110	83.06306306	1	922	0	1110	0	0	21.87744035
191	1310	14.58015267	1	191	0	1310	0	0	13.96335079
1115	1310	85.11450382	1	1115	0	1310	0	0	25.7632287
171	1310	13.05343511	1	171	0	1310	0	0	15.00584795
1108	1310	84.58015267	1	1108	0	1310	0	0	23.86101083
159	1310	12.13740458	1	159	0	1310	0	0	34.08176101
1146	1310	87.48091603	1	1146	0	1310	0	0	32.20855148
17	1316	1.291793313	1	17	0	1316	0	0	23.8333331
185	1316	14.05775076	1	185	0	1316	0	0	32.99775524
464	1316	35.25835866	1	464	0	1316	0	0	31.38061037
521	1316	39.58966565	1	521	0	1316	0	0	30.85910821
113	1316	8.58662614	1	113	0	1316	0	0	32.6778761
1291	1317	98.02581625	1	1291	0	1317	0	0	36.83423703
22	1317	1.670463174	1	22	0	1317	0	0	36.86363636
96	1411	6.80368533	1	96	0	1411	0	0	18.15625
1311	1411	92.91282778	1	1311	0	1411	0	0	35.93897788
56	1411	3.968816442	1	56	0	1411	0	0	16.375

1346	1411	95.39333806	1	1346	0	1411	0	0	35.54829123
65	1411	4.606661942	0.999999994	65	0	1411	0	0	19.29230769
1329	1411	94.18851878	1	1329	0	1411	0	0	36.32129421
65	1411	4.606661942	1	65	0	1411	0	0	20.07692308
1323	1411	93.76328845	1	1323	0	1411	0	0	35.25774754
59	1411	4.181431609	0.999999867	59	0	1411	0	0	16.86440678
1306	1411	92.55846917	1	1306	0	1411	0	0	35.5704441
41	1411	2.905740609	0.996427203	41	0	1411	0	0	16.58536585
1353	1411	95.88944011	1	1353	0	1411	0	0	33.61788618
30	1411	2.126151665	0.999864929	30	0	1411	0	0	18.43333333
1356	1411	96.10205528	1	1356	0	1411	0	0	32.14159292
325	365	89.04109589	1	325	0	365	0	0	30.40296093
15	366	4.098360656	0.99994647	15	0	366	0	0	20.00124984
23	366	6.284153005	1	23	0	366	0	0	19.07410142
7	366	1.912568306	0.999962268	7	0	366	0	0	17.03298782
4	366	1.092896175	0.99957759	4	0	366	0	0	16.30764655
313	366	85.51912568	1	313	0	366	0	0	27.48696422
13	366	3.551912568	1	13	0	366	0	0	17.84060535
4	366	1.092896175	0.99999848	4	0	366	0	0	16.87303036
10	366	2.732240437	0.999395789	10	0	366	0	0	16.53648172
13	366	3.551912568	0.999997205	13	0	366	0	0	17.09868394
47	368	12.77173913	0.999999999	47	0	368	0	0	17.10638298
317	368	86.14130435	1	317	0	368	0	0	27.55835962
32	370	8.648648649	0.950805325	32	0	370	0	0	14.59375
327	370	88.37837838	1	327	0	370	0	0	26.43119266
13	370	3.513513514	0.998959754	13	0	370	0	0	16.31432487
7	370	1.891891892	0.998876182	7	0	370	0	0	15.7863391
10	370	2.702702703	0.999981946	10	0	370	0	0	15.85018621
273	370	73.78378378	1	273	0	370	0	0	30.67456618
55	370	14.86486486	1	55	0	370	0	0	19.05458986
8	370	2.162162162	0.998664382	8	0	370	0	0	18.59994784
63	372	16.93548387	1	63	0	372	0	0	16.98412698
304	372	81.72043011	1	304	0	372	0	0	27.13157895
4	375	1.066666667	0.999997025	4	0	375	0	0	64
371	375	98.93333333	1	371	0	375	0	0	58.64959569
40	415	9.638554217	1	40	0	415	0	0	63.25
368	415	88.6746988	1	368	0	415	0	0	41.80434783
6	415	1.445783133	0.999999996	6	0	415	0	0	50.5
409	415	98.55421687	1	409	0	415	0	0	62.01222494
413	2212	18.67088608	1	413	0	2212	0	0	40.17917676
1799	2212	81.32911392	1	1799	0	2212	0	0	37.15842135
1815	1816	99.94493392	1	1815	0	1816	0	0	38.4584022

Read count	Read coverage	# unique start positions	# unique end positions	BaseQRankSum	Read position test probability	Read direction test probability	Homopolymer	Homopolymer length	QUAL
11690	11700	8	7		1	1	No	1	200
13098	15611	11	12	47.43	0	1	No	1	200
2492	15611	6	8		0	1	No	1	200
14010	15615	12	13	42.64	0	1	No	1	200
1594	15615	5	9		0	1	No	1	200
13311	15615	12	13	53.3	0	1	No	1	200
2287	15615	5	8		0	1	No	1	200
3921	3925	5	10		1	1	No	1	200
3924	3925	5	10		1	1	No	1	200
3609	3923	6	7	23.53	0.643079571	1	Yes	7	200
67	3923	2	1	6.26	0.984002327	1	Yes	7	200
243	3923	2	3		0.028857129	1	Yes	7	200
3867	3923	5	7	8.65	0.330634083	1	Yes	5	200
55	3923	1	2		4.06228E-09	1	Yes	5	200
73	3997	2	4	1.62	0	1	No	1	200
3919	3997	6	8		7.00551E-14	1	No	1	200
77	3992	2	4	10.67	0	1	No	1	200
3911	3992	5	6		0	1	No	1	200
76	76	1	3		1	1	No	1	200
76	76	1	3		1	1	No	1	200
112	1046	1	2	-6.38	1	1	No	1	200
927	1046	5	4		1	1	No	1	200
92	1046	1	2	-10.83	1	1	No	1	200
938	1046	5	4		1	1	No	1	200
29	1046	2	2	-6.82	1	1	No	1	57.61201437
989	1046	5	4		1	1	No	1	200
281	1046	3	3	-21.64	1	1	No	1	200
763	1046	4	3		1	1	No	1	200
12	1046	1	1	-4.99	1	1	No	1	200
1000	1046	5	4		1	1	No	1	200
28	1046	1	2	-6.96	1	1	No	1	59.19842689
210	1107	2	3	-19.16	0.000920249	1	No	1	200
893	1107	6	6		0.332382039	1	No	1	200
148	1110	3	3	-12.5	0.004389615	1	No	1	200
30	1110	2	4	-3.96	4.99853E-06	1	No	1	67.13768146
922	1110	7	6		0.944745507	1	No	1	200
191	1310	3	1	-16.65	7.74936E-14	1	No	1	200
1115	1310	9	7		0.252690307	1	No	1	200
171	1310	3	3	-14.93	1.46961E-10	1	No	1	200
1108	1310	9	6		0.100280875	1	No	1	200
159	1310	5	4	4.97	0.011927575	1	Yes	4	200
1146	1310	10	6		0.923396514	1	Yes	4	200
17	1316	2	3	-12.75	0.36103491	1	No	1	200
185	1316	6	4		0.000551947	1	No	1	200
464	1316	7	5	-8.87	0.005922731	1	Yes	5	200
521	1316	8	4	-13.35	0.021663421	1	No	1	200
113	1316	3	3	-11.27	6.16048E-06	1	Yes	5	200
1291	1317	12	7		1	1	No	1	200
22	1317	4	2		0.001140401	1	Yes	5	200
96	1411	4	2	-14.63	0	1	No	1	200
1311	1411	18	7		0.008626601	1	No	1	200
56	1411	3	1	-11.52	0	1	No	1	200

1346	1411	17	7		0.172661511	1	No	1	200
65	1411	4	2	-12.11	0	1	No	1	81.93820028
1329	1411	18	7		0.045042523	1	No	1	200
65	1411	4	1	-11.68	0	1	No	1	200
1323	1411	18	7		0.064325173	1	No	1	200
59	1411	6	2	-12.19	0	1	No	1	68.7516985
1306	1411	17	7		0.000531387	1	No	1	200
41	1411	2	2	-9.76	0	1	No	1	24.4699161
1353	1411	18	7		0.240616989	1	No	1	200
30	1411	4	1	-7.69	0	1	No	1	38.69436277
1356	1411	18	7		0.432870824	1	No	1	200
325	365	13	3	9.04	1	1	No	1	200
15	366	2	1	3.91	1	1	No	1	42.713987
23	366	3	1		1	1	No	1	200
7	366	2	1	-4.79	1	1	No	1	44.23294778
4	366	3	1	-5.07	1	1	No	1	33.74266117
313	366	13	3		1	1	No	1	200
13	366	2	1	-4.47	1	1	No	1	95.22878709
4	366	1	1	-4.62	1	1	No	1	58.18070704
10	366	2	1	-6.01	1	1	No	1	32.18811229
13	366	3	1	-6.77	1	1	No	1	55.53680345
47	368	2	1	-8.56	0.625571396	1	No	1	90.96910037
317	368	14	3		0.912828866	1	No	1	200
32	370	3	1	-8.9	0.570364318	1	No	1	13.08081908
327	370	15	3		0.905736677	1	No	1	200
13	370	3	1	-7.91	0.713745882	1	No	1	29.82864029
7	370	2	1	-5.64	0.788291473	1	No	1	29.49303823
10	370	2	1	-6.96	0.748894859	1	No	1	47.4341694
273	370	13	3		0.663102146	1	No	1	200
55	370	3	1	-11.37	0.455689408	1	No	1	200
8	370	4	1	-4.35	0.773176921	1	No	1	28.74317607
63	372	6	1	-9.72	0.117261199	1	No	1	200
304	372	14	3		0.520797122	1	No	1	200
4	375	2	1	1.37	0	1	No	1	55.26454641
371	375	16	3		0.566036415	1	No	1	200
40	415	1	2	6.76	0	1	No	1	200
368	415	17	3		2.9109E-06	1	No	1	200
6	415	4	1	-2.21	0.722888849	1	No	1	83.5654732
409	415	18	4		1	1	No	1	200
413	2212	19	4	6.42	0	1	No	1	200
1799	2212	7	8		0	1	No	1	200
1815	1816	6	6		1	1	No	1	200

Supplementary Table S2 A05_C33-A Variant Table

Reference mitogenome	Region	Type	Reference	Allele	Reference allele	Length	Linkage	Zygosity
NC_012920_rCRS H2a_haplo	73	SNV	A	G	No	1		Homozygous
NC_012920_rCRS H2a_haplo	146	SNV	T	C	No	1		Heterozygous
NC_012920_rCRS H2a_haplo	146	SNV	T	T	Yes	1		Heterozygous
NC_012920_rCRS H2a_haplo	150	SNV	C	T	No	1		Heterozygous
NC_012920_rCRS H2a_haplo	150	SNV	C	C	Yes	1		Heterozygous
NC_012920_rCRS H2a_haplo	152	SNV	T	C	No	1		Heterozygous
NC_012920_rCRS H2a_haplo	152	SNV	T	T	Yes	1		Heterozygous
NC_012920_rCRS H2a_haplo	182	SNV	C	T	No	1		Heterozygous
NC_012920_rCRS H2a_haplo	182	SNV	C	C	Yes	1		Heterozygous
NC_012920_rCRS H2a_haplo	215	SNV	A	G	No	1		Homozygous
NC_012920_rCRS H2a_haplo	263	SNV	A	G	No	1		Homozygous
NC_012920_rCRS H2a_haplo	302^303	Insertion	-	C	No	1		Heterozygous
NC_012920_rCRS H2a_haplo	302^303	Insertion	-	-	Yes	0		Heterozygous
NC_012920_rCRS H2a_haplo	310	SNV	T	C	No	1		Heterozygous
NC_012920_rCRS H2a_haplo	310	SNV	T	T	Yes	1		Heterozygous
NC_012920_rCRS H2a_haplo	310^311	Insertion	-	C	No	1		Heterozygous
NC_012920_rCRS H2a_haplo	310^311	Insertion	-	-	Yes	0		Heterozygous
NC_012920_rCRS H2a_haplo	351	SNV	A	G	No	1		Heterozygous
NC_012920_rCRS H2a_haplo	351	SNV	A	A	Yes	1		Heterozygous
NC_012920_rCRS H2a_haplo	16126	SNV	T	C	No	1		Homozygous
NC_012920_rCRS H2a_haplo	16263	SNV	T	A	No	1		Heterozygous
NC_012920_rCRS H2a_haplo	16263	SNV	T	T	Yes	1		Heterozygous
NC_012920_rCRS H2a_haplo	16294	SNV	C	T	No	1		Homozygous
NC_012920_rCRS H2a_haplo	16296	SNV	C	T	No	1		Homozygous
NC_012920_rCRS H2a_haplo	16301	SNV	C	T	No	1		Homozygous
NC_012920_rCRS H2a_haplo	16519	SNV	T	C	No	1		Homozygous

Supplementary Table S2 A05_C33-A Variant Table

**Count**	**Coverage**	**Frequency**	Probability	Forward read count	Reverse read count	Forward read coverage	Reverse read coverage	Forward/reverse balance	Average quality
8739	8754	99.82864976	1	8739	0	8754	0	0	63.35862227
1193	11834	10.08112219	1	1193	0	11834	0	0	32.95641241
10620	11834	89.74142302	1	10620	0	11834	0	0	37.34227872
1944	11834	16.42724354	1	1944	0	11834	0	0	36.3132716
9887	11834	83.54740578	1	9887	0	11834	0	0	37.98088399
1401	11833	11.83977013	1	1401	0	11833	0	0	37.06352605
10422	11833	88.07572044	1	10422	0	11833	0	0	37.83304548
143	11824	1.209404601	1	143	0	11824	0	0	37.72027972
11660	11824	98.61299053	1	11660	0	11824	0	0	33.89253859
3072	3094	99.28894635	1	3072	0	3094	0	0	36.7405599
3092	3094	99.93535876	1	3092	0	3094	0	0	62.95116429
56	3094	1.809954751	1	56	0	3094	0	0	32.94642857
3038	3094	98.19004525	1	3038	0	3094	0	0	30.7116524
60	3092	1.940491591	1	60	0	3092	0	0	32.09866001
2994	3092	96.8305304	1	2994	0	3092	0	0	25.68204806
2984	3092	96.50711514	1	2984	0	3092	0	0	36.99631367
108	3092	3.492884864	1	108	0	3092	0	0	30.05555556
86	3176	2.707808564	1	86	0	3176	0	0	35.40697674
3085	3176	97.13476071	1	3085	0	3176	0	0	35.83435981
2386	2390	99.83263598	1	2386	0	2390	0	0	63.77242246
156	1538	10.14304291	1	156	0	1537	1	0	62.90384615
1380	1538	89.72691808	1	1379	1	1537	1	0.000724638	63.02463768
1539	1540	99.93506494	1	1538	1	1539	1	0.000649773	62.97920728
1538	1540	99.87012987	1	1537	1	1539	1	0.000650195	63.48114434
1540	1540	100	1	1539	1	1539	1	0.000649351	63.18831169
5171	5175	99.92270531	1	5171	0	5175	0	0	37.97234577

Supplementary Table S2 A05_C33-A Variant Table

Read count	Read coverage	# unique start positions	# unique end positions	BaseQRankSum	Read position test probability	Read direction test probability	Homopolymer	Homopolymer length	QUAL
8739	8754	6	9		1	1	No	1	200
1193	11834	3	6	-49.89	0	1	No	1	200
10620	11834	9	17		0	1	No	1	200
1944	11834	4	7	-53.06	0	1	No	1	200
9887	11834	9	16		0	1	No	1	200
1401	11833	4	7	-34.46	0	1	No	1	200
10422	11833	9	14		0	1	No	1	200
143	11824	2	2	12.96	0	1	No	1	200
11660	11824	11	14		1	1	No	1	200
3072	3094	5	8		1	1	No	1	200
3092	3094	5	8		1	1	No	1	200
56	3094	1	1	1.34	0.974714505	1	Yes	7	200
3038	3094	5	8		1	1	Yes	7	200
60	3092	1	4	0.91	1	1	No	1	200
2994	3092	5	5		1	1	Yes	7	200
2984	3092	5	5	9.9	1	1	Yes	5	200
108	3092	2	4		1	1	Yes	5	200
86	3176	3	2	1.95	0	1	No	1	200
3085	3176	6	7		1.11022E-16	1	No	1	200
2386	2390	6	6		1	1	No	1	200
156	1538	2	2	-0.82	0	0.958144917	No	1	200
1380	1538	20	5		0	0.999974184	No	1	200
1539	1540	22	6		1	1	No	1	200
1538	1540	22	6		1	1	No	1	200
1540	1540	22	6		1	1	No	1	200
5171	5175	8	7		1	1	No	1	200

Supplementary Table S2 A06_DoTc2 Variant Table

Reference mitogenome	Region	Type	Reference	Allele	Reference allele	Length	Linkage	Zygosity
NC_012920_rCRS H2a_haplo	73	SNV	A	G	No	1		Homozygous
NC_012920_rCRS H2a_haplo	146	SNV	T	C	No	1		Heterozygous
NC_012920_rCRS H2a_haplo	146	SNV	T	T	Yes	1		Heterozygous
NC_012920_rCRS H2a_haplo	150	SNV	C	T	No	1		Heterozygous
NC_012920_rCRS H2a_haplo	150	SNV	C	C	Yes	1		Heterozygous
NC_012920_rCRS H2a_haplo	152	SNV	T	C	No	1		Heterozygous
NC_012920_rCRS H2a_haplo	152	SNV	T	T	Yes	1		Heterozygous
NC_012920_rCRS H2a_haplo	217	SNV	T	C	No	1		Homozygous
NC_012920_rCRS H2a_haplo	263	SNV	A	G	No	1		Homozygous
NC_012920_rCRS H2a_haplo	310	SNV	T	C	No	1		Heterozygous
NC_012920_rCRS H2a_haplo	310	SNV	T	T	Yes	1		Heterozygous
NC_012920_rCRS H2a_haplo	310^311	Insertion	-	C	No	1		Heterozygous
NC_012920_rCRS H2a_haplo	310^311	Insertion	-	-	Yes	0		Heterozygous
NC_012920_rCRS H2a_haplo	340	SNV	C	T	No	1		Homozygous
NC_012920_rCRS H2a_haplo	508	SNV	A	G	No	1		Homozygous
NC_012920_rCRS H2a_haplo	513^514	Insertion	-	CA	No	2		Homozygous
NC_012920_rCRS H2a_haplo	16051	SNV	A	G	No	1		Homozygous
NC_012920_rCRS H2a_haplo	16129	SNV	G	C	No	1		Homozygous
NC_012920_rCRS H2a_haplo	16140	SNV	T	A	No	1		Heterozygous
NC_012920_rCRS H2a_haplo	16140	SNV	T	T	Yes	1		Heterozygous
NC_012920_rCRS H2a_haplo	16149	SNV	A	C	No	1		Heterozygous
NC_012920_rCRS H2a_haplo	16149	SNV	A	A	Yes	1		Heterozygous
NC_012920_rCRS H2a_haplo	16172	SNV	T	A	No	1		Heterozygous
NC_012920_rCRS H2a_haplo	16172	SNV	T	C	No	1		Heterozygous
NC_012920_rCRS H2a_haplo	16172	SNV	T	T	Yes	1		Heterozygous
NC_012920_rCRS H2a_haplo	16178	SNV	T	A	No	1		Heterozygous
NC_012920_rCRS H2a_haplo	16178	SNV	T	T	Yes	1		Heterozygous
NC_012920_rCRS H2a_haplo	16183^16184	Insertion	-	C	No	1		Heterozygous
NC_012920_rCRS H2a_haplo	16183^16184	Insertion	-	CC	No	2		Heterozygous
NC_012920_rCRS H2a_haplo	16183^16184	Insertion	-	CCC	No	3		Heterozygous
NC_012920_rCRS H2a_haplo	16183^16184	Insertion	-	-	Yes	0		Heterozygous
NC_012920_rCRS H2a_haplo	16189	SNV	T	C	No	1		Heterozygous
NC_012920_rCRS H2a_haplo	16189	Deletion	T	-	No	1		Heterozygous
NC_012920_rCRS H2a_haplo	16224	SNV	T	C	No	1		Heterozygous
NC_012920_rCRS H2a_haplo	16224	SNV	T	T	Yes	1		Heterozygous
NC_012920_rCRS H2a_haplo	16244	SNV	G	C	No	1		Heterozygous
NC_012920_rCRS H2a_haplo	16244	SNV	G	G	Yes	1		Heterozygous
NC_012920_rCRS H2a_haplo	16244^16245	Insertion	-	C	No	1		Heterozygous
NC_012920_rCRS H2a_haplo	16244^16245	Insertion	-	-	Yes	0		Heterozygous
NC_012920_rCRS H2a_haplo	16249	SNV	T	C	No	1		Heterozygous
NC_012920_rCRS H2a_haplo	16249	SNV	T	T	Yes	1		Heterozygous
NC_012920_rCRS H2a_haplo	16256	SNV	C	A	No	1		Heterozygous
NC_012920_rCRS H2a_haplo	16256	SNV	C	T	No	1		Heterozygous
NC_012920_rCRS H2a_haplo	16263	SNV	T	A	No	1		Heterozygous
NC_012920_rCRS H2a_haplo	16263	SNV	T	T	Yes	1		Heterozygous
NC_012920_rCRS H2a_haplo	16519	SNV	T	C	No	1		Homozygous

Supplementary Table S2 A06_DoTc2 Variant Table

**Count**	**Coverage**	**Frequency**	Probability	Forward read count	Reverse read count	Forward read coverage	Reverse read coverage	Forward/reverse balance	Average quality
8195	8205	99.8781231	1	8195	0	8205	0	0	63.43599756
1365	11790	11.57760814	1	1365	0	11790	0	0	32.96410256
10398	11790	88.19338422	1	10398	0	11790	0	0	37.53221773
2216	11795	18.78762187	1	2216	0	11795	0	0	36.42057762
9574	11795	81.16998728	1	9574	0	11795	0	0	38.15448089
9698	11797	82.20734085	1	9698	0	11797	0	0	38.1703444
2090	11797	17.71636857	1	2090	0	11797	0	0	37.19760766
3596	3602	99.83342587	1	3596	0	3602	0	0	37.94521691
3600	3602	99.94447529	1	3600	0	3602	0	0	63.23916667
66	3602	1.83231538	1	66	0	3602	0	0	34.12121212
3517	3602	97.64019989	1	3517	0	3602	0	0	27.61472846
3509	3602	97.41810105	1	3509	0	3602	0	0	37.57395269
93	3602	2.581898945	1	93	0	3602	0	0	32.12903226
3591	3611	99.4461368	1	3591	0	3611	0	0	37.43609023
10	10	100	1	10	0	10	0	0	35.9
9	10	90	1	9	0	10	0	0	37.44444444
1036	1042	99.42418426	1	1036	0	1042	0	0	35.66602317
1042	1042	100	1	1042	0	1042	0	0	62.73512476
51	1042	4.894433781	0.999979401	51	0	1042	0	0	14.78431373
974	1042	93.47408829	1	974	0	1042	0	0	27.20431211
187	1042	17.9462572	1	187	0	1042	0	0	13.93582888
852	1042	81.76583493	1	852	0	1042	0	0	25.65610329
78	1100	7.090909091	1	78	0	1100	0	0	14.80769231
33	1100	3	1	33	0	1100	0	0	21.6969697
981	1100	89.18181818	1	981	0	1100	0	0	26.81141692
97	1249	7.76621297	1	97	0	1249	0	0	14.56701031
1123	1249	89.91192954	1	1123	0	1249	0	0	24.8254675
288	1250	23.04	1	288	0	1250	0	0	30.68051594
137	1250	10.96	1	137	0	1250	0	0	31.39786995
28	1250	2.24	1	28	0	1250	0	0	32.05938801
792	1250	63.36	1	792	0	1250	0	0	31.8560606
1100	1253	87.78930567	1	1100	0	1253	0	0	36.30909091
151	1253	12.05107741	1	151	0	1253	0	0	36.50993377
28	252	11.11111111	0.999690586	28	0	252	0	0	15.78571429
222	252	88.0952381	1	222	0	252	0	0	31.78378378
17	255	6.666666667	1	17	0	255	0	0	18.33819823
238	255	93.33333333	1	238	0	255	0	0	31.76432498
3	255	1.176470588	0.999998293	3	0	255	0	0	34.33333333
252	255	98.82352941	1	252	0	255	0	0	29.8015873
19	256	7.421875	1	19	0	256	0	0	18.94736842
235	256	91.796875	1	235	0	256	0	0	33.29361702
5	257	1.945525292	0.99982754	5	0	257	0	0	47.8
252	257	98.05447471	1	252	0	257	0	0	55.94047619
9	267	3.370786517	1	9	0	267	0	0	64
255	267	95.50561798	1	255	0	267	0	0	52.99215686
2495	2495	100	1	2495	0	2495	0	0	38.19719439

Supplementary Table S2 A06_DoTc2 Variant Table

Read count	Read coverage	# unique start positions	# unique end positions	BaseQRankSum	Read position test probability	Read direction test probability	Homopolymer	Homopolymer length	QUAL
8195	8205	5	9		1	1	No	1	200
1365	11790	4	8	-54.32	0	1	No	1	200
10398	11790	9	14		0	1	No	1	200
2216	11795	4	8	-56.4	0	1	No	1	200
9574	11795	9	11		0	1	No	1	200
9698	11797	8	12	46.53	0	1	No	1	200
2090	11797	5	8		0	1	No	1	200
3596	3602	5	8		1	1	No	1	200
3600	3602	5	8		1	1	No	1	200
66	3602	1	2	3.25	1	1	No	1	200
3517	3602	6	7		1	1	Yes	7	200
3509	3602	6	7	6.91	1	1	Yes	5	200
93	3602	1	5		1	1	Yes	5	200
3591	3611	5	8		0.008439247	1	No	1	200
10	10	1	1		1	1	No	1	200
9	10	1	1		1	1	No	1	200
1036	1042	4	6		1	1	No	1	200
1042	1042	4	6		1	1	No	1	200
51	1042	1	1	-7.41	0.830883587	1	No	1	46.86162296
974	1042	4	6		0.984455	1	No	1	200
187	1042	1	2	-18.79	0.369970759	1	No	1	200
852	1042	4	5		0.671938406	1	No	1	200
78	1100	2	1	-11.62	0.046206283	1	No	1	200
33	1100	4	4	-3.05	8.46656E-13	1	No	1	200
981	1100	7	7		0.709629894	1	No	1	200
97	1249	1	3	-7.38	2.03276E-10	1	No	1	200
1123	1249	9	8		0.206205691	1	No	1	200
288	1250	7	3	-11.07	0.897708027	1	Yes	5	200
137	1250	4	4	-1.18	0.030662093	1	Yes	5	200
28	1250	2	5	2.28	5.74081E-06	1	Yes	5	200
792	1250	8	6		0.509086249	1	Yes	5	200
1100	1253	12	8		0.947326742	1	No	1	200
151	1253	5	4		0.051032952	1	Yes	5	200
28	252	4	3	-7.35	0.747656167	1	No	1	35.09459759
222	252	15	2		0.950972561	1	No	1	200
17	255	4	1	-5.05	0.664368168	1	No	1	200
238	255	16	4		0.961936293	1	No	1	200
3	255	2	1	0.49	0	1	No	1	57.67664723
252	255	17	4		0.314039652	1	No	1	200
19	256	6	2	-5.54	0.000172004	1	No	1	200
235	256	16	3		0.229182737	1	No	1	200
5	257	3	1		0	1	No	1	37.63310863
252	257	17	4		0.296166413	1	No	1	200
9	267	1	1	2.44	0	1	No	1	200
255	267	19	3		0.17148511	1	No	1	200
2495	2495	7	8		1	1	No	1	200

Supplementary Table S2 A07_151v Variant Table

Reference mitogenome	Region	Type	Reference	Allele	Reference allele	Length	Linkage	Zygosity
NC_012920_rCRS H2a_haplo	146	SNV	T	C	No	1		Heterozygous
NC_012920_rCRS H2a_haplo	146	SNV	T	T	Yes	1		Heterozygous
NC_012920_rCRS H2a_haplo	150	SNV	C	T	No	1		Heterozygous
NC_012920_rCRS H2a_haplo	150	SNV	C	C	Yes	1		Heterozygous
NC_012920_rCRS H2a_haplo	152	SNV	T	C	No	1		Heterozygous
NC_012920_rCRS H2a_haplo	152	SNV	T	T	Yes	1		Heterozygous
NC_012920_rCRS H2a_haplo	263	SNV	A	G	No	1		Homozygous
NC_012920_rCRS H2a_haplo	302^303	Insertion	-	C	No	1		Heterozygous
NC_012920_rCRS H2a_haplo	302^303	Insertion	-	CC	No	2		Heterozygous
NC_012920_rCRS H2a_haplo	302^303	Insertion	-	-	Yes	0		Heterozygous
NC_012920_rCRS H2a_haplo	310^311	Insertion	-	C	No	1		Heterozygous
NC_012920_rCRS H2a_haplo	310^311	Insertion	-	-	Yes	0		Heterozygous
NC_012920_rCRS H2a_haplo	351	SNV	A	G	No	1		Heterozygous
NC_012920_rCRS H2a_haplo	351	SNV	A	A	Yes	1		Heterozygous
NC_012920_rCRS H2a_haplo	477	SNV	T	C	No	1		Homozygous
NC_012920_rCRS H2a_haplo	16172	SNV	T	C	No	1		Heterozygous
NC_012920_rCRS H2a_haplo	16172	SNV	T	T	Yes	1		Heterozygous
NC_012920_rCRS H2a_haplo	16263	SNV	T	A	No	1		Heterozygous
NC_012920_rCRS H2a_haplo	16263	SNV	T	T	Yes	1		Heterozygous
NC_012920_rCRS H2a_haplo	16318	SNV	A	G	No	1		Heterozygous
NC_012920_rCRS H2a_haplo	16318	SNV	A	A	Yes	1		Heterozygous
NC_012920_rCRS H2a_haplo	16519	SNV	T	C	No	1		Homozygous

Supplementary Table S2 A07_151v Variant Table

**Count**	**Coverage**	**Frequency**	Probability	Forward read count	Reverse read count	Forward read coverage	Reverse read coverage	Forward/reverse balance	Average quality
601	5764	10.42678695	1	601	0	5764	0	0	33.02828619
5152	5764	89.38237335	1	5152	0	5764	0	0	37.47204969
986	5767	17.09727761	1	986	0	5767	0	0	36.46450304
4774	5767	82.78134212	1	4774	0	5767	0	0	38.10347717
4832	5768	83.77253814	1	4832	0	5768	0	0	38.13845199
927	5768	16.07142857	1	927	0	5768	0	0	37.26213592
1604	1605	99.9376947	1	1604	0	1605	0	0	62.63965087
1288	1605	80.24922118	1	1288	0	1605	0	0	35.57453416
145	1605	9.034267913	1	145	0	1605	0	0	34.84827586
155	1605	9.657320872	1	155	0	1605	0	0	30.48387097
1575	1605	98.13084112	1	1575	0	1605	0	0	37.48825397
28	1605	1.744548287	1	28	0	1605	0	0	28
27	1632	1.654411765	1	27	0	1632	0	0	36.74074074
1601	1632	98.1004902	1	1601	0	1632	0	0	36.46346034
27	27	100	1	27	0	27	0	0	62
10	679	1.47275405	0.999999566	10	0	679	0	0	35.4
669	679	98.52724595	1	669	0	679	0	0	37.25112108
30	285	10.52631579	1	30	0	285	0	0	62.7
255	285	89.47368421	1	255	0	285	0	0	63.60784314
16	285	5.614035088	1	16	0	285	0	0	39.9375
269	285	94.38596491	1	269	0	285	0	0	41.89591078
1049	1054	99.5256167	1	1049	0	1054	0	0	38.55767398

Supplementary Table S2 A07_151v Variant Table

Read count	Read coverage	# unique start positions	# unique end positions	BaseQRankSum	Read position test probability	Read direction test probability	Homopolymer	Homopolymer length	QUAL
601	5764	3	6	-35.93	0	1	No	1	200
5152	5764	8	11		5.55112E-16	1	No	1	200
986	5767	3	5	-37.67	0	1	No	1	200
4774	5767	8	13		0	1	No	1	200
4832	5768	8	10	30.77	0	1	No	1	200
927	5768	3	8		0	1	No	1	200
1604	1605	3	6		1	1	No	1	200
1288	1605	3	6	22.04	0.722217485	1	Yes	7	200
145	1605	2	2	12.42	0.681691273	1	Yes	7	200
155	1605	2	3		0.138965224	1	Yes	7	200
1575	1605	3	6	6.8	1	1	Yes	5	200
28	1605	2	2		1	1	Yes	5	200
27	1632	2	2	1.48	0	1	No	1	200
1601	1632	4	7		1.2183E-05	1	No	1	200
27	27	1	1		1	1	No	1	200
10	679	2	2	-2.18	0	1	No	1	63.62310181
669	679	7	5		0.542816267	1	No	1	200
30	285	1	3	-0.81	0	1	No	1	200
255	285	9	2		1.09309E-05	1	No	1	200
16	285	3	1	-1.08	0.554575202	1	No	1	200
269	285	10	3		1	1	No	1	200
1049	1054	5	2		1	1	No	1	200

Supplementary Table S2 A08_152v Variant Table

Reference mitogenome	Region	Type	Reference	Allele	Reference allele	Length	Linkage	Zygosity
NC_012920_rCRS H2a_haplo	73	SNV	A	G	No	1		Homozygous
NC_012920_rCRS H2a_haplo	146	SNV	T	C	No	1		Heterozygous
NC_012920_rCRS H2a_haplo	146	SNV	T	T	Yes	1		Heterozygous
NC_012920_rCRS H2a_haplo	150	SNV	C	T	No	1		Heterozygous
NC_012920_rCRS H2a_haplo	150	SNV	C	C	Yes	1		Heterozygous
NC_012920_rCRS H2a_haplo	152	SNV	T	C	No	1		Heterozygous
NC_012920_rCRS H2a_haplo	152	SNV	T	T	Yes	1		Heterozygous
NC_012920_rCRS H2a_haplo	199	SNV	T	C	No	1		Heterozygous
NC_012920_rCRS H2a_haplo	199	SNV	T	T	Yes	1		Heterozygous
NC_012920_rCRS H2a_haplo	203..204	MNV	GT	AC	No	2		Heterozygous
NC_012920_rCRS H2a_haplo	203..204	MNV	GT	GT	Yes	2		Heterozygous
NC_012920_rCRS H2a_haplo	250	SNV	T	C	No	1		Heterozygous
NC_012920_rCRS H2a_haplo	250	SNV	T	T	Yes	1		Heterozygous
NC_012920_rCRS H2a_haplo	263	SNV	A	G	No	1		Homozygous
NC_012920_rCRS H2a_haplo	302^303	Insertion	-	C	No	1		Heterozygous
NC_012920_rCRS H2a_haplo	302^303	Insertion	-	CC	No	2		Heterozygous
NC_012920_rCRS H2a_haplo	302^303	Insertion	-	-	Yes	0		Heterozygous
NC_012920_rCRS H2a_haplo	310^311	Insertion	-	C	No	1		Heterozygous
NC_012920_rCRS H2a_haplo	310^311	Insertion	-	-	Yes	0		Heterozygous
NC_012920_rCRS H2a_haplo	351	SNV	A	G	No	1		Heterozygous
NC_012920_rCRS H2a_haplo	351	SNV	A	A	Yes	1		Heterozygous
NC_012920_rCRS H2a_haplo	451^452	Insertion	-	T	No	1		Homozygous
NC_012920_rCRS H2a_haplo	513^514	Insertion	-	CA	No	2		Heterozygous
NC_012920_rCRS H2a_haplo	513^514	Insertion	-	-	Yes	0		Heterozygous
NC_012920_rCRS H2a_haplo	16129	SNV	G	A	No	1		Heterozygous
NC_012920_rCRS H2a_haplo	16129	SNV	G	G	Yes	1		Heterozygous
NC_012920_rCRS H2a_haplo	16172	SNV	T	C	No	1		Heterozygous
NC_012920_rCRS H2a_haplo	16172	SNV	T	T	Yes	1		Heterozygous
NC_012920_rCRS H2a_haplo	16223	SNV	C	T	No	1		Homozygous
NC_012920_rCRS H2a_haplo	16249	SNV	T	C	No	1		Heterozygous
NC_012920_rCRS H2a_haplo	16249	SNV	T	T	Yes	1		Heterozygous
NC_012920_rCRS H2a_haplo	16263	SNV	T	A	No	1		Heterozygous
NC_012920_rCRS H2a_haplo	16263	SNV	T	T	Yes	1		Heterozygous
NC_012920_rCRS H2a_haplo	16311	SNV	T	C	No	1		Homozygous
NC_012920_rCRS H2a_haplo	16391	SNV	G	A	No	1		Heterozygous
NC_012920_rCRS H2a_haplo	16391	SNV	G	G	Yes	1		Heterozygous
NC_012920_rCRS H2a_haplo	16519	SNV	T	C	No	1		Homozygous

Supplementary Table S2 A08_152v Variant Table

**Count**	**Coverage**	**Frequency**	Probability	Forward read count	Reverse read count	Forward read coverage	Reverse read coverage	Forward/reverse balance
5053	5089	99.29259186	1	5053	0	5089	0	0
835	7266	11.49187999	1	835	0	7266	0	0
6405	7266	88.15028902	1	6405	0	7266	0	0
1344	7266	18.49710983	1	1344	0	7266	0	0
5917	7266	81.43407652	1	5917	0	7266	0	0
955	7266	13.14340765	1	955	0	7266	0	0
6305	7266	86.77401596	1	6305	0	7266	0	0
2155	2187	98.53680841	1	2155	0	2187	0	0
32	2187	1.463191587	1	32	0	2187	0	0
2159	2187	98.71970736	1	2159	0	2187	0	0
28	2187	1.280292638	1	28	0	2187	0	0
2157	2188	98.58318099	1	2157	0	2188	0	0
31	2188	1.416819013	1	31	0	2188	0	0
2188	2188	100	1	2188	0	2188	0	0
1883	2188	86.06032907	1	1883	0	2188	0	0
136	2188	6.215722121	1	136	0	2188	0	0
157	2188	7.175502742	1	157	0	2188	0	0
2147	2187	98.17101052	1	2147	0	2187	0	0
40	2187	1.828989483	1	40	0	2187	0	0
59	2243	2.630405707	1	59	0	2243	0	0
2182	2243	97.280428	1	2182	0	2243	0	0
58	58	100	1	58	0	58	0	0
2	58	3.448275862	1	2	0	58	0	0
56	58	96.55172414	1	56	0	58	0	0
1165	1181	98.64521592	1	1165	0	1181	0	0
16	1181	1.354784081	1	16	0	1181	0	0
1186	1269	93.45941686	1	1186	0	1269	0	0
82	1269	6.46178093	1	82	0	1269	0	0
535	539	99.25788497	1	535	0	539	0	0
7	542	1.291512915	0.999994354	7	0	542	0	0
535	542	98.70848708	1	535	0	542	0	0
23	565	4.07079646	1	23	0	565	0	0
542	565	95.92920354	1	542	0	565	0	0
564	565	99.82300885	1	564	0	565	0	0
2200	2797	78.65570254	1	2200	0	2797	0	0
593	2797	21.20128709	1	593	0	2797	0	0
2234	2237	99.86589182	1	2234	0	2237	0	0

Supplementary Table S2 A08_152v Variant Table

Average quality	Read count	Read coverage	# unique start positions	# unique end positions	BaseQRankSum	Read position test probability	Read direction test probability	Homopolymer	Homopolymer length
63.41104294	5053	5089	6	6		1	1	No	1
32.85508982	835	7266	4	7	-42.91	0	1	No	1
37.58298205	6405	7266	11	10		0	1	No	1
36.4389881	1344	7266	4	7	-44.36	0	1	No	1
38.16461044	5917	7266	10	10		0	1	No	1
37.03141361	955	7266	5	5	-28.05	0	1	No	1
37.99429025	6305	7266	10	12		0	1	No	1
37.80464037	2155	2187	5	8	0.64	1	1	No	1
36.125	32	2187	1	2		1	1	No	1
37.61195881	2159	2187	5	8	0.76	1	1	No	1
36.47534812	28	2187	1	2		1	1	No	1
63.36439499	2157	2188	5	8	0.08	1	1	No	1
61.4516129	31	2188	1	2		0.907785749	1	No	1
62.54981718	2188	2188	5	8		1	1	No	1
35.72545717	1883	2188	6	7	21.61	0.4386737	1	Yes	7
35.02180358	136	2188	2	2	12.74	0.943766705	1	Yes	7
29.78343948	157	2188	2	4		0.002643788	1	Yes	7
37.38751747	2147	2187	5	5	7.31	0.331641049	1	Yes	5
29.975	40	2187	1	3		2.96711E-07	1	Yes	5
36.01694915	59	2243	2	3	1.3	0	1	No	1
36.43263061	2182	2243	7	6		1.78807E-11	1	No	1
60.96551724	58	58	2	2		1	1	No	1
38.25	2	58	1	1	1.01	1	1	No	1
35.625	56	58	2	2		1	1	No	1
63.66008584	1165	1181	3	6	0.25	1	1	No	1
63.3125	16	1181	2	1		0.445029647	1	No	1
37.06661046	1186	1269	5	7	-0.46	4.37939E-09	1	No	1
36.86585366	82	1269	5	2		0	1	No	1
38.13831776	535	539	6	2		1	1	No	1
38.57142857	7	542	4	1	-1.48	0	1	No	1
38.93457944	535	542	6	2		0.168584035	1	No	1
63.82608696	23	565	1	2	-0.16	0	1	No	1
63.28413284	542	565	8	2		0.000138405	1	No	1
40.17553191	564	565	10	2		1	1	No	1
36.57272727	2200	2797	5	4	26.8	0	1	No	1
33.31703204	593	2797	11	3		0	1	No	1
38.18487019	2234	2237	6	4		1	1	No	1

QUAL
200
200
200
200
200
200
200
200
200
200
200
200
200
200
200
200
200
200
200
200
200
200
200
200
200
200
200
200
200
52.48228359
200
200
200
200
200
200
200

Supplementary Table S2 A09_153v Variant Table

Reference mitogenome	Region	Type	Reference	Allele	Reference allele	Length	Linkage	Zygosity
NC_012920_rCRS H2a_haplo	73	SNV	A	G	No	1		Homozygous
NC_012920_rCRS H2a_haplo	146	SNV	T	C	No	1		Heterozygous
NC_012920_rCRS H2a_haplo	146	SNV	T	T	Yes	1		Heterozygous
NC_012920_rCRS H2a_haplo	150	SNV	C	T	No	1		Heterozygous
NC_012920_rCRS H2a_haplo	150	SNV	C	C	Yes	1		Heterozygous
NC_012920_rCRS H2a_haplo	152	SNV	T	C	No	1		Heterozygous
NC_012920_rCRS H2a_haplo	152	SNV	T	T	Yes	1		Heterozygous
NC_012920_rCRS H2a_haplo	199	SNV	T	C	No	1		Heterozygous
NC_012920_rCRS H2a_haplo	199	SNV	T	T	Yes	1		Heterozygous
NC_012920_rCRS H2a_haplo	203..204	MNV	GT	AC	No	2		Heterozygous
NC_012920_rCRS H2a_haplo	203..204	MNV	GT	GT	Yes	2		Heterozygous
NC_012920_rCRS H2a_haplo	250	SNV	T	C	No	1		Heterozygous
NC_012920_rCRS H2a_haplo	250	SNV	T	T	Yes	1		Heterozygous
NC_012920_rCRS H2a_haplo	263	SNV	A	G	No	1		Homozygous
NC_012920_rCRS H2a_haplo	302^303	Insertion	-	C	No	1		Heterozygous
NC_012920_rCRS H2a_haplo	302^303	Insertion	-	CC	No	2		Heterozygous
NC_012920_rCRS H2a_haplo	302^303	Insertion	-	-	Yes	0		Heterozygous
NC_012920_rCRS H2a_haplo	310	SNV	T	C	No	1		Heterozygous
NC_012920_rCRS H2a_haplo	310	SNV	T	T	Yes	1		Heterozygous
NC_012920_rCRS H2a_haplo	310^311	Insertion	-	C	No	1		Heterozygous
NC_012920_rCRS H2a_haplo	310^311	Insertion	-	-	Yes	0		Heterozygous
NC_012920_rCRS H2a_haplo	351	SNV	A	G	No	1		Heterozygous
NC_012920_rCRS H2a_haplo	351	SNV	A	A	Yes	1		Heterozygous
NC_012920_rCRS H2a_haplo	16126	SNV	T	C	No	1		Heterozygous
NC_012920_rCRS H2a_haplo	16126	SNV	T	T	Yes	1		Heterozygous
NC_012920_rCRS H2a_haplo	16129	SNV	G	A	No	1		Heterozygous
NC_012920_rCRS H2a_haplo	16129	SNV	G	G	Yes	1		Heterozygous
NC_012920_rCRS H2a_haplo	16172	SNV	T	C	No	1		Heterozygous
NC_012920_rCRS H2a_haplo	16172	SNV	T	T	Yes	1		Heterozygous
NC_012920_rCRS H2a_haplo	16223	SNV	C	T	No	1		Heterozygous
NC_012920_rCRS H2a_haplo	16223	SNV	C	C	Yes	1		Heterozygous
NC_012920_rCRS H2a_haplo	16231	SNV	T	C	No	1		Heterozygous
NC_012920_rCRS H2a_haplo	16231	SNV	T	T	Yes	1		Heterozygous
NC_012920_rCRS H2a_haplo	16249	SNV	T	C	No	1		Heterozygous
NC_012920_rCRS H2a_haplo	16249	SNV	T	T	Yes	1		Heterozygous
NC_012920_rCRS H2a_haplo	16266	SNV	C	T	No	1		Heterozygous
NC_012920_rCRS H2a_haplo	16266	SNV	C	C	Yes	1		Heterozygous
NC_012920_rCRS H2a_haplo	16311	SNV	T	C	No	1		Heterozygous
NC_012920_rCRS H2a_haplo	16311	SNV	T	T	Yes	1		Heterozygous
NC_012920_rCRS H2a_haplo	16319	SNV	G	A	No	1		Heterozygous
NC_012920_rCRS H2a_haplo	16319	SNV	G	G	Yes	1		Heterozygous
NC_012920_rCRS H2a_haplo	16391	SNV	G	A	No	1		Heterozygous
NC_012920_rCRS H2a_haplo	16391	SNV	G	G	Yes	1		Heterozygous
NC_012920_rCRS H2a_haplo	16399	SNV	A	G	No	1		Heterozygous
NC_012920_rCRS H2a_haplo	16399	SNV	A	A	Yes	1		Heterozygous
NC_012920_rCRS H2a_haplo	16519	SNV	T	C	No	1		Homozygous

Supplementary Table S2 A09_153v Variant Table

**Count**	**Coverage**	**Frequency**	Probability	Forward read count	Reverse read count	Forward read coverage	Reverse read coverage	Forward/reverse balance	Average quality
2814	2840	99.08450704	1	2814	0	2840	0	0	63.35963042
3092	4190	73.7947494	1	3092	0	4190	0	0	36.01811125
1090	4190	26.01431981	1	1090	0	4190	0	0	36.66513761
831	4190	19.83293556	1	831	0	4190	0	0	36.41395909
3357	4190	80.11933174	1	3357	0	4190	0	0	38.04051236
603	4190	14.39140811	1	603	0	4190	0	0	37.04975124
3579	4190	85.4176611	1	3579	0	4190	0	0	37.73176865
106	1354	7.828655835	1	106	0	1354	0	0	38.31132075
1248	1354	92.17134417	1	1248	0	1354	0	0	38.03205128
106	1354	7.828655835	1	106	0	1354	0	0	38.37222112
1248	1354	92.17134417	1	1248	0	1354	0	0	37.43593027
104	1354	7.680945347	1	104	0	1354	0	0	63.61538462
1250	1354	92.31905465	1	1250	0	1354	0	0	62.2792
1352	1354	99.85228951	1	1352	0	1354	0	0	62.48224852
1168	1354	86.26292467	1	1168	0	1354	0	0	35.31678082
92	1354	6.794682422	1	92	0	1354	0	0	34.30978261
84	1354	6.203840473	1	84	0	1354	0	0	29.26190476
24	1353	1.77383592	1	24	0	1353	0	0	32.74628477
1329	1353	98.22616408	1	1329	0	1353	0	0	26.25136063
1325	1353	97.93052476	1	1325	0	1353	0	0	37.2906153
28	1353	2.06947524	1	28	0	1353	0	0	30.56976994
15	1367	1.097293343	1	15	0	1367	0	0	34.73333333
1350	1367	98.75640088	1	1350	0	1367	0	0	35.90444444
568	619	91.76090468	1	568	0	619	0	0	63.55985915
51	619	8.239095315	1	51	0	619	0	0	64
44	619	7.108239095	1	44	0	619	0	0	62.40909091
575	619	92.8917609	1	575	0	619	0	0	62.80173913
51	663	7.692307692	1	51	0	663	0	0	36.1372549
612	663	92.30769231	1	612	0	663	0	0	36.95098039
11	221	4.977375566	1	11	0	221	0	0	37.63636364
210	221	95.02262443	1	210	0	221	0	0	37.0952381
208	221	94.11764706	1	208	0	221	0	0	38.36538462
13	221	5.882352941	1	13	0	221	0	0	37.15384615
3	227	1.321585903	0.973978609	3	0	227	0	0	36.33333333
224	227	98.6784141	1	224	0	227	0	0	38.84821429
212	227	93.39207048	1	212	0	227	0	0	61.54716981
14	227	6.167400881	1	14	0	227	0	0	64
224	227	98.6784141	1	224	0	227	0	0	39.11607143
3	227	1.321585903	0.987944098	3	0	227	0	0	47.33333333
211	227	92.95154185	1	211	0	227	0	0	37.30331754
15	227	6.607929515	1	15	0	227	0	0	41.6
75	1054	7.115749526	1	75	0	1054	0	0	36.76
978	1054	92.78937381	1	978	0	1054	0	0	35.57668712
745	1050	70.95238095	1	745	0	1050	0	0	36.88187919
305	1050	29.04761905	1	305	0	1050	0	0	33.48852459
848	852	99.53051643	1	848	0	852	0	0	38.88207547

Read count	Read coverage	# unique start positions	# unique end positions	BaseQRankSum	Read position test probability	Read direction test probability	Homopolymer	Homopolymer length	QUAL
2814	2840	4	4	1.05	1	1	No	1	200
3092	4190	6	9	7.47	0	1	No	1	200
1090	4190	6	9		0	1	No	1	200
831	4190	3	8	-32.45	0	1	No	1	200
3357	4190	8	9		0	1	No	1	200
603	4190	5	6	-18.04	0	1	No	1	200
3579	4190	6	12		2.22045E-16	1	No	1	200
106	1354	2	3	0.95	1	1	No	1	200
1248	1354	4	8		1	1	No	1	200
106	1354	2	3	3.3	1	1	No	1	200
1248	1354	4	8		1	1	No	1	200
104	1354	2	3	1.77	0.377176864	1	No	1	200
1250	1354	4	8		0.877803149	1	No	1	200
1352	1354	4	9		1	1	No	1	200
1168	1354	4	7	14.76	0.340300664	1	Yes	7	200
92	1354	2	1	9.2	0.573145109	1	Yes	7	200
84	1354	1	6		2.52065E-05	1	Yes	7	200
24	1353	1	2	5.12	1	1	No	1	200
1329	1353	5	8		1	1	Yes	7	200
1325	1353	5	8	5.33	1	1	Yes	5	200
28	1353	1	2		1	1	Yes	5	200
15	1367	2	4	0.64	0	1	No	1	200
1350	1367	5	8		0.015137111	1	No	1	200
568	619	3	2	-0.35	1	1	No	1	200
51	619	1	2		1	1	No	1	200
44	619	1	2	0.36	0.468961351	1	No	1	200
575	619	3	2		0.956856069	1	No	1	200
51	663	3	4	-3.98	0.07335527	1	No	1	200
612	663	6	4		0.811597837	1	No	1	200
11	221	2	1	2.34	1	1	No	1	200
210	221	6	3		1	1	No	1	200
208	221	5	3	0.33	1	1	No	1	200
13	221	3	1		1	1	No	1	200
3	227	2	1	-1.61	0.0022396	1	No	1	15.84669497
224	227	8	4		0.831229348	1	No	1	200
212	227	8	4	-1.12	0.985285046	1	No	1	200
14	227	4	1		0.519960873	1	No	1	200
224	227	9	4	-1.35	0.856160992	1	No	1	200
3	227	2	1		0.00477381	1	No	1	19.18800287
211	227	8	4	-3.2	0.909068122	1	No	1	200
15	227	4	1		0.386428677	1	No	1	200
75	1054	3	2	8.52	6.98451E-05	1	No	1	200
978	1054	14	7		0.782467449	1	No	1	200
745	1050	4	3	14.36	0	1	No	1	200
305	1050	12	4		0	1	No	1	200
848	852	5	3		1	1	No	1	200

Supplementary Table S2 A10_154v Variant Table

Reference mitogenome	Region	Type	Reference	Allele	Reference allele	Length	Linkage	Zygosity
NC_012920_rCRS H2a_haplo	72	SNV	T	C	No	1		Homozygous
NC_012920_rCRS H2a_haplo	146	SNV	T	C	No	1		Heterozygous
NC_012920_rCRS H2a_haplo	146	SNV	T	T	Yes	1		Heterozygous
NC_012920_rCRS H2a_haplo	150	SNV	C	T	No	1		Heterozygous
NC_012920_rCRS H2a_haplo	150	SNV	C	C	Yes	1		Heterozygous
NC_012920_rCRS H2a_haplo	152	SNV	T	C	No	1		Heterozygous
NC_012920_rCRS H2a_haplo	152	SNV	T	T	Yes	1		Heterozygous
NC_012920_rCRS H2a_haplo	204	SNV	T	C	No	1		Homozygous
NC_012920_rCRS H2a_haplo	263	SNV	A	G	No	1		Homozygous
NC_012920_rCRS H2a_haplo	302^303	Insertion	-	C	No	1		Heterozygous
NC_012920_rCRS H2a_haplo	302^303	Insertion	-	CC	No	2		Heterozygous
NC_012920_rCRS H2a_haplo	302^303	Insertion	-	-	Yes	0		Heterozygous
NC_012920_rCRS H2a_haplo	310	SNV	T	C	No	1		Heterozygous
NC_012920_rCRS H2a_haplo	310	SNV	T	T	Yes	1		Heterozygous
NC_012920_rCRS H2a_haplo	310^311	Insertion	-	C	No	1		Heterozygous
NC_012920_rCRS H2a_haplo	310^311	Insertion	-	-	Yes	0		Heterozygous
NC_012920_rCRS H2a_haplo	351	SNV	A	G	No	1		Heterozygous
NC_012920_rCRS H2a_haplo	351	SNV	A	A	Yes	1		Heterozygous
NC_012920_rCRS H2a_haplo	16172	SNV	T	C	No	1		Heterozygous
NC_012920_rCRS H2a_haplo	16172	SNV	T	T	Yes	1		Heterozygous
NC_012920_rCRS H2a_haplo	16263	SNV	T	A	No	1		Heterozygous
NC_012920_rCRS H2a_haplo	16263	SNV	T	T	Yes	1		Heterozygous
NC_012920_rCRS H2a_haplo	16298	SNV	T	C	No	1		Homozygous

Supplementary Table S2 A10_154v Variant Table

**Count**	**Coverage**	**Frequency**	Probability	Forward read count	Reverse read count	Forward read coverage	Reverse read coverage	Forward/reverse balance	Average quality
4273	4305	99.25667828	1	4273	0	4305	0	0	62.97378891
563	5854	9.617355654	1	563	0	5854	0	0	32.9928952
5274	5854	90.09224462	1	5274	0	5854	0	0	37.19908987
969	5853	16.55561251	1	969	0	5853	0	0	36.31888545
4882	5853	83.41021698	1	4882	0	5853	0	0	37.86460467
659	5854	11.25725999	1	659	0	5854	0	0	36.90591806
5188	5854	88.62316365	1	5188	0	5854	0	0	37.69082498
1551	1553	99.871217	1	1551	0	1553	0	0	38.04642166
1553	1553	100	1	1553	0	1553	0	0	61.96394076
1253	1553	80.6825499	1	1253	0	1553	0	0	34.93695132
164	1553	10.56020605	1	164	0	1553	0	0	33.53658537
123	1553	7.92015454	1	123	0	1553	0	0	27.86178862
19	1551	1.225016119	1	19	0	1551	0	0	28.57808653
1511	1551	97.4210187	1	1511	0	1551	0	0	25.66116127
1506	1551	97.09864603	1	1506	0	1551	0	0	37.18791501
45	1551	2.901353965	1	45	0	1551	0	0	27.06666667
25	1575	1.587301587	1	25	0	1575	0	0	35.12
1549	1575	98.34920635	1	1549	0	1575	0	0	35.57779212
18	1276	1.410658307	1	18	0	1276	0	0	35
1254	1276	98.27586207	1	1254	0	1276	0	0	36.50558214
23	315	7.301587302	1	23	0	315	0	0	62.08695652
292	315	92.6984127	1	292	0	315	0	0	62.99657534
315	315	100	1	315	0	315	0	0	63.57777778

Supplementary Table S2 A10_154v Variant Table

Read count	Read coverage	# unique start positions	# unique end positions	BaseQRankSum	Read position test probability	Read direction test probability	Homopolymer	Homopolymer length	QUAL
4273	4305	3	4		1	1	No	1	200
563	5854	3	6	-34.09	0	1	No	1	200
5274	5854	6	13		9.99201E-16	1	No	1	200
969	5853	3	9	-35.32	0	1	No	1	200
4882	5853	6	9		0	1	No	1	200
659	5854	4	7	-20.75	0	1	No	1	200
5188	5854	5	11		0	1	No	1	200
1551	1553	3	9		1	1	No	1	200
1553	1553	3	9		1	1	No	1	200
1253	1553	3	7	21.89	0.695014441	1	Yes	7	200
164	1553	2	2	13.63	0.657352809	1	Yes	7	200
123	1553	2	4		0.060838934	1	Yes	7	200
19	1551	1	1	-6.05	1	1	No	1	200
1511	1551	3	7		1	1	Yes	7	200
1506	1551	3	7	8.66	1	1	Yes	5	200
45	1551	2	2		1	1	Yes	5	200
25	1575	2	2	1.64	0	1	No	1	200
1549	1575	3	8		1.19989E-05	1	No	1	200
18	1276	3	2	-2.33	0	1	No	1	200
1254	1276	7	7		0.495794341	1	No	1	200
23	315	1	2	-0.74	0	1	No	1	200
292	315	7	2		3.30854E-05	1	No	1	200
315	315	8	3		1	1	No	1	200

Supplementary Table S2 A11_155v Variant Table

Reference mitogenome	Region	Type	Reference	Allele	Reference allele	Length	Linkage	Zygosity
NC_012920_rCRS H2a_haplo	73	SNV	A	G	No	1		Homozygous
NC_012920_rCRS H2a_haplo	94	SNV	G	A	No	1		Homozygous
NC_012920_rCRS H2a_haplo	146	SNV	T	C	No	1		Heterozygous
NC_012920_rCRS H2a_haplo	146	SNV	T	T	Yes	1		Heterozygous
NC_012920_rCRS H2a_haplo	150	SNV	C	T	No	1		Heterozygous
NC_012920_rCRS H2a_haplo	150	SNV	C	C	Yes	1		Heterozygous
NC_012920_rCRS H2a_haplo	152	SNV	T	C	No	1		Heterozygous
NC_012920_rCRS H2a_haplo	152	SNV	T	T	Yes	1		Heterozygous
NC_012920_rCRS H2a_haplo	248	Deletion	A	-	No	1		Homozygous
NC_012920_rCRS H2a_haplo	263	SNV	A	G	No	1		Homozygous
NC_012920_rCRS H2a_haplo	286..287	Deletion	AA	-	No	2		Homozygous
NC_012920_rCRS H2a_haplo	302^303	Insertion	-	C	No	1		Heterozygous
NC_012920_rCRS H2a_haplo	302^303	Insertion	-	CC	No	2		Heterozygous
NC_012920_rCRS H2a_haplo	302^303	Insertion	-	-	Yes	0		Heterozygous
NC_012920_rCRS H2a_haplo	310^311	Insertion	-	TC	No	2		Homozygous
NC_012920_rCRS H2a_haplo	351	SNV	A	G	No	1		Heterozygous
NC_012920_rCRS H2a_haplo	351	SNV	A	A	Yes	1		Heterozygous
NC_012920_rCRS H2a_haplo	16172	SNV	T	C	No	1		Heterozygous
NC_012920_rCRS H2a_haplo	16172	SNV	T	T	Yes	1		Heterozygous
NC_012920_rCRS H2a_haplo	16223	SNV	C	T	No	1		Homozygous
NC_012920_rCRS H2a_haplo	16249	SNV	T	C	No	1		Heterozygous
NC_012920_rCRS H2a_haplo	16249	SNV	T	T	Yes	1		Heterozygous
NC_012920_rCRS H2a_haplo	16263	SNV	T	A	No	1		Heterozygous
NC_012920_rCRS H2a_haplo	16263	SNV	T	T	Yes	1		Heterozygous
NC_012920_rCRS H2a_haplo	16298	SNV	T	C	No	1		Homozygous
NC_012920_rCRS H2a_haplo	16325	SNV	T	C	No	1		Homozygous
NC_012920_rCRS H2a_haplo	16327	SNV	C	T	No	1		Homozygous
NC_012920_rCRS H2a_haplo	16519	SNV	T	C	No	1		Homozygous

Supplementary Table S2 A11_155v Variant Table

**Count**	**Coverage**	**Frequency**	Probability	Forward read count	Reverse read count	Forward read coverage	Reverse read coverage	Forward/reverse balance	Average quality
3320	3343	99.31199521	1	3320	0	3343	0	0	63.44096386
3306	3343	98.89320969	1	3306	0	3343	0	0	62.72262553
410	4376	9.36928702	1	410	0	4376	0	0	32.61463415
3953	4376	90.33363803	1	3953	0	4376	0	0	37.41386289
651	4377	14.87320082	1	651	0	4377	0	0	36.50537634
3718	4377	84.94402559	1	3718	0	4377	0	0	38.11161915
445	4375	10.17142857	1	445	0	4375	0	0	36.76404494
3928	4375	89.78285714	1	3928	0	4375	0	0	37.93380855
1035	1036	99.9034749	1	1035	0	1036	0	0	62.72850242
1036	1036	100	1	1036	0	1036	0	0	62.10907336
1033	1036	99.71042471	1	1033	0	1036	0	0	63.41529526
927	1036	89.47876448	1	927	0	1036	0	0	35.10463862
49	1036	4.72972973	1	49	0	1036	0	0	36.06122449
56	1036	5.405405405	1	56	0	1036	0	0	29.60714286
1018	1036	98.26254826	1	1018	0	1036	0	0	32.61051238
12	1044	1.149425287	1	12	0	1044	0	0	34.16666667
1032	1044	98.85057471	1	1032	0	1044	0	0	35.91182171
16	615	2.601626016	1	16	0	615	0	0	34.9375
599	615	97.39837398	1	599	0	615	0	0	37.28881469
352	356	98.87640449	1	352	0	356	0	0	38.28977273
4	359	1.114206128	0.991998371	4	0	359	0	0	37.5
355	359	98.88579387	1	355	0	359	0	0	39.07323944
35	395	8.860759494	1	35	0	395	0	0	62.77142857
360	395	91.13924051	1	360	0	395	0	0	62.925
393	396	99.24242424	1	393	0	396	0	0	63.61068702
393	396	99.24242424	1	393	0	396	0	0	39.99491094
393	396	99.24242424	1	393	0	396	0	0	37.4605598
2006	2009	99.85067198	1	2006	0	2009	0	0	37.96360917

Supplementary Table S2 A11_155v Variant Table

Read count	Read coverage	# unique start positions	# unique end positions	BaseQRankSum	Read position test probability	Read direction test probability	Homopolymer	Homopolymer length	QUAL
3320	3343	5	8		1	1	No	1	200
3306	3343	5	8	3.27	1	1	No	1	200
410	4376	3	6	-29.72	0	1	No	1	200
3953	4376	7	12		1.19138E-12	1	No	1	200
651	4377	3	5	-31.72	0	1	No	1	200
3718	4377	8	11		0	1	No	1	200
445	4375	3	3	-19.87	0	1	No	1	200
3928	4375	8	14		2.55684E-13	1	No	1	200
1035	1036	3	6		1	1	No	1	200
1036	1036	3	6		1	1	No	1	200
1033	1036	3	5	1.21	1	1	No	1	200
927	1036	3	5	10.79	1	1	Yes	7	200
49	1036	1	2	9.8	1	1	Yes	7	200
56	1036	1	2		1	1	Yes	7	200
1018	1036	3	5	2.1	1	1	No	1	200
12	1044	2	3	0.54	0	1	No	1	95.22878709
1032	1044	3	4		0.003469997	1	No	1	200
16	615	2	2	-2.69	1.2803E-09	1	No	1	200
599	615	6	5		0.718149539	1	No	1	200
352	356	8	2	1.46	1	1	No	1	200
4	359	3	1	-1.32	4.40035E-06	1	No	1	20.96821594
355	359	10	2		0.696556625	1	No	1	200
35	395	2	2	-0.68	0	1	No	1	200
360	395	11	2		2.29399E-06	1	No	1	200
393	396	13	3		1	1	No	1	200
393	396	13	3		1	1	No	1	200
393	396	13	3		1	1	No	1	200
2006	2009	6	6		1	1	No	1	200

Supplementary Table S2 A12_156v Variant Table

Reference mitogenome	Region	Type	Reference	Allele	Reference allele	Length	Linkage	Zygosity
NC_012920_rCRS H2a_haplo	72	SNV	T	C	No	1		Heterozygous
NC_012920_rCRS H2a_haplo	72	SNV	T	T	Yes	1		Heterozygous
NC_012920_rCRS H2a_haplo	146	SNV	T	C	No	1		Heterozygous
NC_012920_rCRS H2a_haplo	146	SNV	T	T	Yes	1		Heterozygous
NC_012920_rCRS H2a_haplo	150	SNV	C	T	No	1		Heterozygous
NC_012920_rCRS H2a_haplo	150	SNV	C	C	Yes	1		Heterozygous
NC_012920_rCRS H2a_haplo	152	SNV	T	C	No	1		Heterozygous
NC_012920_rCRS H2a_haplo	152	SNV	T	T	Yes	1		Heterozygous
NC_012920_rCRS H2a_haplo	204	SNV	T	C	No	1		Heterozygous
NC_012920_rCRS H2a_haplo	204	SNV	T	T	Yes	1		Heterozygous
NC_012920_rCRS H2a_haplo	263	SNV	A	G	No	1		Homozygous
NC_012920_rCRS H2a_haplo	286	Deletion	A	-	No	1		Heterozygous
NC_012920_rCRS H2a_haplo	286	SNV	A	A	Yes	1		Heterozygous
NC_012920_rCRS H2a_haplo	302	SNV	A	C	No	1		Heterozygous
NC_012920_rCRS H2a_haplo	302	SNV	A	A	Yes	1		Heterozygous
NC_012920_rCRS H2a_haplo	302^303	Insertion	-	C	No	1		Heterozygous
NC_012920_rCRS H2a_haplo	302^303	Insertion	-	-	Yes	0		Heterozygous
NC_012920_rCRS H2a_haplo	310	SNV	T	C	No	1		Heterozygous
NC_012920_rCRS H2a_haplo	310	SNV	T	T	Yes	1		Heterozygous
NC_012920_rCRS H2a_haplo	310^311	Insertion	-	C	No	1		Heterozygous
NC_012920_rCRS H2a_haplo	310^311	Insertion	-	-	Yes	0		Heterozygous
NC_012920_rCRS H2a_haplo	351	SNV	A	G	No	1		Heterozygous
NC_012920_rCRS H2a_haplo	351	SNV	A	A	Yes	1		Heterozygous
NC_012920_rCRS H2a_haplo	16249	SNV	T	C	No	1		Heterozygous
NC_012920_rCRS H2a_haplo	16249	SNV	T	T	Yes	1		Heterozygous
NC_012920_rCRS H2a_haplo	16256	SNV	C	A	No	1		Heterozygous
NC_012920_rCRS H2a_haplo	16256	SNV	C	C	Yes	1		Heterozygous
NC_012920_rCRS H2a_haplo	16263	SNV	T	A	No	1		Heterozygous
NC_012920_rCRS H2a_haplo	16263	SNV	T	T	Yes	1		Heterozygous
NC_012920_rCRS H2a_haplo	16298	SNV	T	C	No	1		Heterozygous
NC_012920_rCRS H2a_haplo	16298	SNV	T	T	Yes	1		Heterozygous
NC_012920_rCRS H2a_haplo	16375	Deletion	C	-	No	1		Heterozygous
NC_012920_rCRS H2a_haplo	16375	SNV	C	C	Yes	1		Heterozygous
NC_012920_rCRS H2a_haplo	16519	SNV	T	C	No	1		Heterozygous
NC_012920_rCRS H2a_haplo	16519	SNV	T	T	Yes	1		Heterozygous

Supplementary Table S2 A12_156v Variant Table

**Count**	**Coverage**	**Frequency**	Probability	Forward read count	Reverse read count	Forward read coverage	Reverse read coverage	Forward/reverse balance	Average quality
160	3444	4.645760743	1	160	0	3444	0	0	62.70625
3283	3444	95.32520325	1	3283	0	3444	0	0	63.43314042
151	3838	3.934340803	1	151	0	3838	0	0	31.65562914
3678	3838	95.83116206	1	3678	0	3838	0	0	37.42196846
238	3837	6.202762575	1	238	0	3837	0	0	36.10504202
3598	3837	93.7711754	1	3598	0	3837	0	0	38.06281267
187	3837	4.873599166	1	187	0	3837	0	0	36.1657754
3646	3837	95.02215272	1	3646	0	3837	0	0	37.82967636
18	394	4.568527919	1	18	0	394	0	0	37.61111111
376	394	95.43147208	1	376	0	394	0	0	37
394	394	100	1	394	0	394	0	0	62.8071066
4	394	1.015228426	0.999999904	4	0	394	0	0	64
390	394	98.98477157	1	390	0	394	0	0	63.73846154
7	394	1.776649746	0.999994438	7	0	394	0	0	23.28571429
387	394	98.22335025	1	387	0	394	0	0	30.45219638
24	394	6.091370558	1	24	0	394	0	0	34.04166667
369	394	93.65482234	1	369	0	394	0	0	30.37669377
11	394	2.791878173	1	11	0	394	0	0	35.2083247
378	394	95.93908629	1	378	0	394	0	0	26.17435862
375	394	95.17766497	1	375	0	394	0	0	37.2
18	394	4.568527919	1	18	0	394	0	0	32.05555556
9	403	2.23325062	1	9	0	403	0	0	34.22222222
394	403	97.76674938	1	394	0	403	0	0	35.56091371
3	153	1.960784314	0.993559669	3	0	153	0	0	38.33333333
150	153	98.03921569	1	150	0	153	0	0	39.02666667
2	153	1.307189542	0.901043001	2	0	153	0	0	64
151	153	98.69281046	1	151	0	153	0	0	63.28476821
25	179	13.96648045	1	25	0	179	0	0	63.28
154	179	86.03351955	1	154	0	179	0	0	63.17532468
8	179	4.469273743	1	8	0	179	0	0	64
170	179	94.97206704	1	170	0	179	0	0	63.94705882
38	3712	1.023706897	1	38	0	3712	0	0	37.02631579
3670	3712	98.86853448	1	3670	0	3712	0	0	38.22234332
3354	3534	94.90662139	1	3354	0	3534	0	0	37.89445438
179	3534	5.06508206	1	179	0	3534	0	0	38.15083799

Supplementary Table S2 A12_156v Variant Table

Read count	Read coverage	# unique start positions	# unique end positions	BaseQRankSum	Read position test probability	Read direction test probability	Homopolymer	Homopolymer length	QUAL
160	3444	1	2	-0.4	1	1	No	1	200
3283	3444	4	6		1	1	No	1	200
151	3838	2	5	-18.3	0	1	No	1	200
3678	3838	6	7		8.42066E-05	1	No	1	200
238	3837	3	4	-20.88	0	1	No	1	200
3598	3837	6	8		0	1	No	1	200
187	3837	3	2	-14.1	0	1	No	1	200
3646	3837	6	9		4.75586E-08	1	No	1	200
18	394	1	1	1.7	1	1	No	1	200
376	394	2	3		1	1	No	1	200
394	394	2	3		1	1	No	1	200
4	394	1	1	0.08	0.648712463	1	Yes	6	70.16373713
390	394	2	3		1	1	Yes	6	200
7	394	1	1	-1.93	0.896376452	1	No	1	52.54792441
387	394	2	3		1	1	Yes	7	200
24	394	1	1	4.31	0.810754585	1	Yes	7	200
369	394	2	3		0.976020173	1	Yes	7	200
11	394	1	1	1.74	1	1	No	1	200
378	394	2	3		1	1	Yes	7	200
375	394	2	3	5.21	1	1	Yes	5	200
18	394	2	1		1	1	Yes	5	200
9	403	3	2	-0.67	0	1	No	1	200
394	403	3	4		0.052081059	1	No	1	200
3	153	3	1	-1.55	1.49358E-12	1	No	1	21.91091778
150	153	6	3		0.170064301	1	No	1	200
2	153	2	1	0.16	0	1	No	1	10.04553482
151	153	6	3		0.387673704	1	No	1	200
25	179	1	2	-0.36	0	1	No	1	200
154	179	9	3		0.000235519	1	No	1	200
8	179	3	2	0.03	0.118207563	1	No	1	200
170	179	10	3		0.937070277	1	No	1	200
38	3712	3	2	-3.06	0.924006729	1	Yes	6	200
3670	3712	15	7		1	1	Yes	6	200
3354	3534	5	3	-2.24	1	1	No	1	200
179	3534	2	1		1	1	No	1	200

Supplementary Table S2 B01_500v Variant Table

Reference mitogenome	Region	Type	Reference	Allele	Reference allele	Length	Linkage	Zygosity
NC_012920_rCRS H2a_haplo	73	SNV	A	G	No	1		Homozygous
NC_012920_rCRS H2a_haplo	146	SNV	T	C	No	1		Heterozygous
NC_012920_rCRS H2a_haplo	146	SNV	T	T	Yes	1		Heterozygous
NC_012920_rCRS H2a_haplo	150	SNV	C	T	No	1		Heterozygous
NC_012920_rCRS H2a_haplo	150	SNV	C	C	Yes	1		Heterozygous
NC_012920_rCRS H2a_haplo	152	SNV	T	C	No	1		Heterozygous
NC_012920_rCRS H2a_haplo	152	SNV	T	T	Yes	1		Heterozygous
NC_012920_rCRS H2a_haplo	263	SNV	A	G	No	1		Homozygous
NC_012920_rCRS H2a_haplo	302^303	Insertion	-	C	No	1		Heterozygous
NC_012920_rCRS H2a_haplo	302^303	Insertion	-	CC	No	2		Heterozygous
NC_012920_rCRS H2a_haplo	302^303	Insertion	-	-	Yes	0		Heterozygous
NC_012920_rCRS H2a_haplo	310^311	Insertion	-	C	No	1		Heterozygous
NC_012920_rCRS H2a_haplo	310^311	Insertion	-	-	Yes	0		Heterozygous
NC_012920_rCRS H2a_haplo	351	SNV	A	G	No	1		Heterozygous
NC_012920_rCRS H2a_haplo	351	SNV	A	A	Yes	1		Heterozygous
NC_012920_rCRS H2a_haplo	497	SNV	C	T	No	1		Homozygous
NC_012920_rCRS H2a_haplo	16148	SNV	C	T	No	1		Heterozygous
NC_012920_rCRS H2a_haplo	16148	SNV	C	C	Yes	1		Heterozygous
NC_012920_rCRS H2a_haplo	16172	SNV	T	C	No	1		Heterozygous
NC_012920_rCRS H2a_haplo	16172	SNV	T	T	Yes	1		Heterozygous
NC_012920_rCRS H2a_haplo	16224	SNV	T	C	No	1		Homozygous
NC_012920_rCRS H2a_haplo	16249	SNV	T	C	No	1		Heterozygous
NC_012920_rCRS H2a_haplo	16249	SNV	T	T	Yes	1		Heterozygous
NC_012920_rCRS H2a_haplo	16256	SNV	C	A	No	1		Heterozygous
NC_012920_rCRS H2a_haplo	16256	SNV	C	C	Yes	1		Heterozygous
NC_012920_rCRS H2a_haplo	16263	SNV	T	A	No	1		Heterozygous
NC_012920_rCRS H2a_haplo	16263	SNV	T	T	Yes	1		Heterozygous
NC_012920_rCRS H2a_haplo	16311	SNV	T	C	No	1		Homozygous
NC_012920_rCRS H2a_haplo	16519	SNV	T	C	No	1		Homozygous

Supplementary Table S2 B01_500v Variant Table

**Count**	**Coverage**	**Frequency**	Probability	Forward read count	Reverse read count	Forward read coverage	Reverse read coverage	Forward/reverse balance
5115	5134	99.62991819	1	5115	0	5134	0	0
842	7320	11.50273224	1	842	0	7320	0	0
6465	7320	88.31967213	1	6465	0	7320	0	0
1315	7321	17.96202705	1	1315	0	7321	0	0
6003	7321	81.99699495	1	6003	0	7321	0	0
977	7321	13.34517142	1	977	0	7321	0	0
6339	7321	86.58653189	1	6339	0	7321	0	0
2189	2192	99.86313869	1	2189	0	2192	0	0
1939	2192	88.4580292	1	1939	0	2192	0	0
112	2192	5.109489051	1	112	0	2192	0	0
131	2192	5.976277372	1	131	0	2192	0	0
2156	2192	98.35766423	1	2156	0	2192	0	0
33	2192	1.505474453	1	33	0	2192	0	0
36	2225	1.617977528	1	36	0	2225	0	0
2188	2225	98.33707865	1	2188	0	2225	0	0
32	33	96.96969697	1	32	0	33	0	0
8	750	1.066666667	0.999999749	8	0	749	1	0
742	750	98.93333333	1	741	1	749	1	0.001347709
15	829	1.809408926	1	15	0	828	1	0
814	829	98.19059107	1	813	1	828	1	0.001228501
315	318	99.05660377	1	315	0	318	0	0
5	321	1.557632399	0.999908774	5	0	321	0	0
316	321	98.4423676	1	316	0	321	0	0
4	322	1.242236025	0.750438013	4	0	322	0	0
318	322	98.75776398	1	318	0	322	0	0
32	354	9.039548023	1	32	0	354	0	0
322	354	90.96045198	1	322	0	354	0	0
353	354	99.71751412	1	353	0	354	0	0
1542	1542	100	1	1542	0	1542	0	0

Supplementary Table S2 B01_500v Variant Table

Average quality	Read count	Read coverage	# unique start positions	# unique end positions	BaseQRankSum	Read position test probability	Read direction test probability	Homopolymer
63.43069404	5115	5134	8	5		1	1	No
32.98099762	842	7320	4	6	-42.95	0	1	No
37.57324053	6465	7320	13	10		0	1	No
36.62813688	1315	7321	4	6	-43.14	0	1	No
38.14209562	6003	7321	13	9		0	1	No
37.07574207	977	7321	6	5	-29.71	0	1	No
38.05111216	6339	7321	11	10		0	1	No
62.94655094	2189	2192	6	6		1	1	No
35.79834966	1939	2192	6	4	18.15	0.110833921	1	Yes
34.68303571	112	2192	2	1	8.88	0.705495452	1	Yes
30.80152672	131	2192	3	3		2.75741E-06	1	Yes
37.58395176	2156	2192	6	5	7.01	1	1	Yes
31.45454545	33	2192	1	3		1	1	Yes
36.80555556	36	2225	2	2	0.27	0	1	No
36.72760512	2188	2225	6	6		1.7166E-07	1	No
34.03125	32	33	1	1		1	1	No
35.625	8	750	1	1	-0.89	0.98991505	0.99524879	No
38.4083558	742	750	4	5		1	1	No
38	15	829	2	1	-2.44	0	0.992012227	No
37.43488943	814	829	8	6		0.663322716	1	No
37.06984127	315	318	7	3	-2.95	1	1	No
38.4	5	321	4	1	-1.02	0	1	No
38.09810127	316	321	7	3		0.242269031	1	No
52.5	4	322	2	1	-0.6	0	1	No
62.63522013	318	322	7	3		0.150099739	1	No
63.28125	32	354	2	1	0.2	0	1	No
61.95341615	322	354	9	3		1.66919E-05	1	No
42.04532578	353	354	11	3		1	1	No
38.53437095	1542	1542	6	5		1	1	No

Homopolymer length	QUAL
1	200
1	200
1	200
1	200
1	200
1	200
1	200
1	200
7	200
7	200
7	200
5	200
5	200
1	200
1	200
1	200
1	65.99807511
1	200
1	200
1	200
1	200
1	40.39880415
1	200
1	6.028215654
1	200
1	200
1	200
1	200
1	200

Supplementary Table S2 B02_503v Variant Table

Reference mitogenome	Region	Type	Reference	Allele	Reference allele	Length	Linkage	Zygosity
NC_012920_rCRS H2a_haplo	146	SNV	T	C	No	1		Heterozygous
NC_012920_rCRS H2a_haplo	146	SNV	T	T	Yes	1		Heterozygous
NC_012920_rCRS H2a_haplo	150	SNV	C	T	No	1		Heterozygous
NC_012920_rCRS H2a_haplo	150	SNV	C	C	Yes	1		Heterozygous
NC_012920_rCRS H2a_haplo	152	SNV	T	C	No	1		Heterozygous
NC_012920_rCRS H2a_haplo	152	SNV	T	T	Yes	1		Heterozygous
NC_012920_rCRS H2a_haplo	263	SNV	A	G	No	1		Homozygous
NC_012920_rCRS H2a_haplo	302^303	Insertion	-	C	No	1		Heterozygous
NC_012920_rCRS H2a_haplo	302^303	Insertion	-	CC	No	2		Heterozygous
NC_012920_rCRS H2a_haplo	302^303	Insertion	-	CCC	No	3		Heterozygous
NC_012920_rCRS H2a_haplo	302^303	Insertion	-	-	Yes	0		Heterozygous
NC_012920_rCRS H2a_haplo	310	SNV	T	C	No	1		Heterozygous
NC_012920_rCRS H2a_haplo	310	SNV	T	T	Yes	1		Heterozygous
NC_012920_rCRS H2a_haplo	310^311	Insertion	-	C	No	1		Heterozygous
NC_012920_rCRS H2a_haplo	310^311	Insertion	-	-	Yes	0		Heterozygous
NC_012920_rCRS H2a_haplo	16172	SNV	T	C	No	1		Heterozygous
NC_012920_rCRS H2a_haplo	16172	SNV	T	T	Yes	1		Heterozygous
NC_012920_rCRS H2a_haplo	16263	SNV	T	A	No	1		Heterozygous
NC_012920_rCRS H2a_haplo	16263	SNV	T	T	Yes	1		Heterozygous
NC_012920_rCRS H2a_haplo	16396	SNV	T	G	No	1		Heterozygous
NC_012920_rCRS H2a_haplo	16396	SNV	T	T	Yes	1		Heterozygous
NC_012920_rCRS H2a_haplo	16519	SNV	T	C	No	1		Homozygous

Supplementary Table S2 B02_503v Variant Table

**Count**	**Coverage**	**Frequency**	Probability	Forward read count	Reverse read count	Forward read coverage	Reverse read coverage	Forward/reverse balance	Average quality
738	6386	11.55652991	1	738	0	6386	0	0	33.80894309
5637	6386	88.27121829	1	5637	0	6386	0	0	37.08373248
772	6386	12.08894457	1	772	0	6386	0	0	36.28108808
5611	6386	87.86407767	1	5611	0	6386	0	0	37.7788273
5616	6386	87.94237394	1	5616	0	6386	0	0	37.78080484
758	6386	11.869715	1	758	0	6386	0	0	36.67810026
1263	1263	100	1	1263	0	1263	0	0	62.00079177
971	1263	76.88044339	1	971	0	1263	0	0	34.93820803
165	1263	13.06413302	1	165	0	1263	0	0	33.31818182
15	1263	1.187648456	1	15	0	1263	0	0	28.53333333
112	1263	8.867775139	1	112	0	1263	0	0	26.50892857
18	1263	1.425178147	1	18	0	1263	0	0	27.38888889
1214	1263	96.12034838	1	1214	0	1263	0	0	25.05321252
1212	1263	95.96199525	1	1212	0	1263	0	0	37.03712871
50	1263	3.958828187	1	50	0	1263	0	0	26.94
27	690	3.913043478	1	27	0	690	0	0	35.2962963
662	690	95.94202899	1	662	0	690	0	0	36.34441088
32	589	5.432937182	1	32	0	589	0	0	61.625
557	589	94.56706282	1	557	0	589	0	0	62.75942549
96	1905	5.039370079	1	96	0	1905	0	0	19.22916667
1804	1905	94.69816273	1	1804	0	1905	0	0	35.94955654
1329	1331	99.84973704	1	1329	0	1331	0	0	37.69977427

Supplementary Table S2 B02_503v Variant Table

Read count	Read coverage	# unique start positions	# unique end positions	BaseQRankSum	Read position test probability	Read direction test probability	Homopolymer	Homopolymer length	QUAL
738	6386	4	7	-30.5	0	1	No	1	200
5637	6386	7	9		6.57274E-12	1	No	1	200
772	6386	3	5	-33.49	0	1	No	1	200
5611	6386	7	9		0	1	No	1	200
5616	6386	7	8	26.48	0	1	No	1	200
758	6386	3	6		0	1	No	1	200
1263	1263	4	5		1	1	No	1	200
971	1263	4	4	21.33	1	1	Yes	7	200
165	1263	1	2	13.65	1	1	Yes	7	200
15	1263	1	1	1.15	1	1	Yes	7	200
112	1263	2	4		1	1	Yes	7	200
18	1263	1	1	-6.31	1	1	No	1	200
1214	1263	4	5		1	1	Yes	7	200
1212	1263	4	5	10.88	1	1	Yes	5	200
50	1263	1	2		1	1	Yes	5	200
27	690	3	2	-2.37	0	1	No	1	200
662	690	9	5		0.45032643	1	No	1	200
32	589	1	2	-1	0	1	No	1	200
557	589	11	3		2.82894E-06	1	No	1	200
96	1905	5	1	-14.67	0	1	No	1	200
1804	1905	14	6		0.276581727	1	No	1	200
1329	1331	4	4		1	1	No	1	200

Supplementary Table S2 B03_504v Variant Table

Reference mitogenome	Region	Type	Reference	Allele	Reference allele	Length	Linkage	Zygosity
NC_012920_rCRS H2a_haplo	66	Deletion	G	-	No	1		Heterozygous
NC_012920_rCRS H2a_haplo	66	SNV	G	G	Yes	1		Heterozygous
NC_012920_rCRS H2a_haplo	73	SNV	A	G	No	1		Heterozygous
NC_012920_rCRS H2a_haplo	73	SNV	A	A	Yes	1		Heterozygous
NC_012920_rCRS H2a_haplo	146	SNV	T	C	No	1		Heterozygous
NC_012920_rCRS H2a_haplo	146	SNV	T	T	Yes	1		Heterozygous
NC_012920_rCRS H2a_haplo	150	SNV	C	T	No	1		Heterozygous
NC_012920_rCRS H2a_haplo	150	SNV	C	C	Yes	1		Heterozygous
NC_012920_rCRS H2a_haplo	152	SNV	T	C	No	1		Heterozygous
NC_012920_rCRS H2a_haplo	152	SNV	T	T	Yes	1		Heterozygous
NC_012920_rCRS H2a_haplo	199	SNV	T	C	No	1		Heterozygous
NC_012920_rCRS H2a_haplo	199	SNV	T	T	Yes	1		Heterozygous
NC_012920_rCRS H2a_haplo	248	Deletion	A	-	No	1		Heterozygous
NC_012920_rCRS H2a_haplo	248	SNV	A	A	Yes	1		Heterozygous
NC_012920_rCRS H2a_haplo	263	SNV	A	G	No	1		Homozygous
NC_012920_rCRS H2a_haplo	302^303	Insertion	-	C	No	1		Heterozygous
NC_012920_rCRS H2a_haplo	302^303	Insertion	-	CC	No	2		Heterozygous
NC_012920_rCRS H2a_haplo	302^303	Insertion	-	CCC	No	3		Heterozygous
NC_012920_rCRS H2a_haplo	302^303	Insertion	-	-	Yes	0		Heterozygous
NC_012920_rCRS H2a_haplo	310	SNV	T	C	No	1		Heterozygous
NC_012920_rCRS H2a_haplo	310	SNV	T	T	Yes	1		Heterozygous
NC_012920_rCRS H2a_haplo	310^311	Insertion	-	C	No	1		Heterozygous
NC_012920_rCRS H2a_haplo	310^311	Insertion	-	-	Yes	0		Heterozygous
NC_012920_rCRS H2a_haplo	351	SNV	A	G	No	1		Heterozygous
NC_012920_rCRS H2a_haplo	351	SNV	A	A	Yes	1		Heterozygous
NC_012920_rCRS H2a_haplo	397	SNV	A	C	No	1		Heterozygous
NC_012920_rCRS H2a_haplo	397	SNV	A	A	Yes	1		Heterozygous
NC_012920_rCRS H2a_haplo	514..515	Deletion	CA	-	No	2		Heterozygous
NC_012920_rCRS H2a_haplo	514..515	MNV	CA	CA	Yes	2		Heterozygous
NC_012920_rCRS H2a_haplo	16140	SNV	T	A	No	1		Heterozygous
NC_012920_rCRS H2a_haplo	16140	SNV	T	T	Yes	1		Heterozygous
NC_012920_rCRS H2a_haplo	16143	SNV	T	C	No	1		Heterozygous
NC_012920_rCRS H2a_haplo	16143	SNV	T	T	Yes	1		Heterozygous
NC_012920_rCRS H2a_haplo	16149	SNV	A	C	No	1		Heterozygous
NC_012920_rCRS H2a_haplo	16149	SNV	A	A	Yes	1		Heterozygous
NC_012920_rCRS H2a_haplo	16152	SNV	T	C	No	1		Heterozygous
NC_012920_rCRS H2a_haplo	152..16153	MNV	TG	CT	No	2		Heterozygous
NC_012920_rCRS H2a_haplo	152..16153	MNV	TG	TG	Yes	2		Heterozygous
NC_012920_rCRS H2a_haplo	16153	SNV	G	T	No	1		Heterozygous
NC_012920_rCRS H2a_haplo	16159	SNV	C	A	No	1		Heterozygous
NC_012920_rCRS H2a_haplo	16159	SNV	C	C	Yes	1		Heterozygous
NC_012920_rCRS H2a_haplo	16161	SNV	T	A	No	1		Heterozygous
NC_012920_rCRS H2a_haplo	16161	SNV	T	T	Yes	1		Heterozygous
NC_012920_rCRS H2a_haplo	16166	SNV	A	C	No	1		Heterozygous
NC_012920_rCRS H2a_haplo	16166	SNV	A	A	Yes	1		Heterozygous
NC_012920_rCRS H2a_haplo	16172	SNV	T	A	No	1		Heterozygous
NC_012920_rCRS H2a_haplo	16172	SNV	T	C	No	1		Heterozygous
NC_012920_rCRS H2a_haplo	16172	SNV	T	T	Yes	1		Heterozygous
NC_012920_rCRS H2a_haplo	16175	SNV	A	C	No	1		Heterozygous
NC_012920_rCRS H2a_haplo	16175	SNV	A	A	Yes	1		Heterozygous
NC_012920_rCRS H2a_haplo	16178	SNV	T	A	No	1		Heterozygous
NC_012920_rCRS H2a_haplo	16178	Deletion	T	-	No	1		Heterozygous

NC_012920_rCRS H2a_haplo	16178	SNV	T	T	Yes	1		Heterozygous
NC_012920_rCRS H2a_haplo	16180	Deletion	A	-	No	1		Heterozygous
NC_012920_rCRS H2a_haplo	180..16181	Deletion	AA	-	No	2		Heterozygous
NC_012920_rCRS H2a_haplo	180..16183	MNV	AAAA	AAAA	Yes	4		Heterozygous
NC_012920_rCRS H2a_haplo	182..16183	Replacement	AA	CCC	No	3		Heterozygous
NC_012920_rCRS H2a_haplo	182..16183	Replacement	AA	CCCC	No	4		Heterozygous
NC_012920_rCRS H2a_haplo	182..16183	MNV	AA	CC	No	2		Heterozygous
NC_012920_rCRS H2a_haplo	16183	SNV	A	C	No	1		Heterozygous
NC_012920_rCRS H2a_haplo	16189	SNV	T	C	No	1		Heterozygous
NC_012920_rCRS H2a_haplo	16189	SNV	T	T	Yes	1		Heterozygous
NC_012920_rCRS H2a_haplo	16224	SNV	T	C	No	1		Heterozygous
NC_012920_rCRS H2a_haplo	16224	SNV	T	T	Yes	1		Heterozygous
NC_012920_rCRS H2a_haplo	237^16238	Insertion	-	C	No	1		Heterozygous
NC_012920_rCRS H2a_haplo	237^16238	Insertion	-	-	Yes	0		Heterozygous
NC_012920_rCRS H2a_haplo	16244	SNV	G	C	No	1		Heterozygous
NC_012920_rCRS H2a_haplo	16244	SNV	G	G	Yes	1		Heterozygous
NC_012920_rCRS H2a_haplo	16249	SNV	T	C	No	1		Heterozygous
NC_012920_rCRS H2a_haplo	16249	Deletion	T	-	No	1		Heterozygous
NC_012920_rCRS H2a_haplo	16249	SNV	T	T	Yes	1		Heterozygous
NC_012920_rCRS H2a_haplo	257^16258	Insertion	-	A	No	1		Heterozygous
NC_012920_rCRS H2a_haplo	257^16258	Insertion	-	-	Yes	0		Heterozygous
NC_012920_rCRS H2a_haplo	16263	SNV	T	A	No	1		Heterozygous
NC_012920_rCRS H2a_haplo	16263	SNV	T	T	Yes	1		Heterozygous
NC_012920_rCRS H2a_haplo	16264	Deletion	C	-	No	1		Heterozygous
NC_012920_rCRS H2a_haplo	16264	SNV	C	C	Yes	1		Heterozygous
NC_012920_rCRS H2a_haplo	16274	SNV	G	A	No	1		Heterozygous
NC_012920_rCRS H2a_haplo	16274	SNV	G	G	Yes	1		Heterozygous
NC_012920_rCRS H2a_haplo	16278	SNV	C	T	No	1		Heterozygous
NC_012920_rCRS H2a_haplo	16278	SNV	C	C	Yes	1		Heterozygous
NC_012920_rCRS H2a_haplo	16288	SNV	T	C	No	1		Heterozygous
NC_012920_rCRS H2a_haplo	16288	SNV	T	T	Yes	1		Heterozygous
NC_012920_rCRS H2a_haplo	16293	SNV	A	C	No	1		Heterozygous
NC_012920_rCRS H2a_haplo	16293	SNV	A	A	Yes	1		Heterozygous
NC_012920_rCRS H2a_haplo	16304	SNV	T	C	No	1		Heterozygous
NC_012920_rCRS H2a_haplo	16304	SNV	T	T	Yes	1		Heterozygous
NC_012920_rCRS H2a_haplo	16311	SNV	T	C	No	1		Heterozygous
NC_012920_rCRS H2a_haplo	16311	SNV	T	T	Yes	1		Heterozygous
NC_012920_rCRS H2a_haplo	16519	SNV	T	C	No	1		Heterozygous
NC_012920_rCRS H2a_haplo	16519	SNV	T	T	Yes	1		Heterozygous

**Count**	**Coverage**	**Frequency**	Probability	Forward read count	Reverse read count	Forward read coverage	Reverse read coverage	Forward/reverse balance	Average quality
42	4023	1.043997017	1	42	0	4023	0	0	61.33333333
3979	4023	98.90628884	1	3979	0	4023	0	0	63.79919578
3974	4023	98.78200348	1	3974	0	4023	0	0	63.27981882
48	4023	1.193139448	1	48	0	4023	0	0	52.375
860	6141	14.00423384	1	860	0	6141	0	0	32.88837209
5266	6141	85.75150627	1	5266	0	6141	0	0	37.40733004
1354	6141	22.0485263	1	1354	0	6141	0	0	36.50073855
4781	6141	77.85376974	1	4781	0	6141	0	0	37.96025936
954	6141	15.53492916	1	954	0	6141	0	0	37.03039832
5177	6141	84.30223091	1	5177	0	6141	0	0	37.87405833
2078	2126	97.74223895	1	2078	0	2126	0	0	37.88354187
48	2126	2.257761054	1	48	0	2126	0	0	37.3125
2077	2126	97.69520226	1	2077	0	2126	0	0	62.23639865
48	2126	2.257761054	1	48	0	2126	0	0	62.1875
2115	2126	99.48259643	1	2115	0	2126	0	0	60.73522459
1625	2126	76.434619	1	1625	0	2126	0	0	35.23507692
268	2126	12.60583255	1	268	0	2126	0	0	34.72761194
62	2126	2.916274694	1	62	0	2126	0	0	31.84408602
163	2126	7.666980245	1	163	0	2126	0	0	28.68711656
31	2124	1.459510358	1	31	0	2124	0	0	31.38709677
2069	2124	97.41054614	1	2069	0	2124	0	0	25.90865152
2061	2124	97.03389831	1	2061	0	2124	0	0	37.12227074
62	2124	2.919020716	1	62	0	2124	0	0	29.82258065
49	2166	2.262234534	1	49	0	2166	0	0	34.32653061
2116	2166	97.69159741	1	2116	0	2166	0	0	35.59310019
2	43	4.651162791	0.999537217	2	0	43	0	0	26.5
41	43	95.34883721	1	41	0	43	0	0	37.3902439
40	43	93.02325581	1	40	0	43	0	0	35.675
2	43	4.651162791	1	2	0	43	0	0	37.25
46	374	12.29946524	1	46	0	374	0	0	14.69565217
321	374	85.82887701	1	321	0	374	0	0	21.58255452
42	374	11.22994652	0.990265258	42	0	374	0	0	13.16666667
328	374	87.70053476	1	328	0	374	0	0	22.45121951
145	374	38.77005348	1	145	0	374	0	0	13.66206897
226	374	60.42780749	1	226	0	374	0	0	22.05752212
27	374	7.219251337	1	27	0	374	0	0	13.74023377
5	374	1.336898396	0.999996824	5	0	374	0	0	14.29813955
320	374	85.56149733	1	320	0	374	0	0	24.16261558
21	374	5.614973262	1	21	0	374	0	0	15.09807496
24	374	6.417112299	0.999992231	24	0	374	0	0	13.25
349	374	93.31550802	1	349	0	374	0	0	23.00286533
99	388	25.51546392	1	99	0	388	0	0	13.83838384
282	388	72.68041237	1	282	0	388	0	0	21.65248227
13	388	3.350515464	0.999967934	13	0	388	0	0	21.69230769
375	388	96.64948454	1	375	0	388	0	0	32.30133333
53	388	13.65979381	1	53	0	388	0	0	15.79245283
23	388	5.927835052	0.999575233	23	0	388	0	0	16.26086957
309	388	79.63917526	1	309	0	388	0	0	20.48543689
76	411	18.49148418	1	76	0	411	0	0	14.10464692
324	411	78.83211679	1	324	0	411	0	0	25.21494947
66	411	16.05839416	1	66	0	411	0	0	13.86363636
11	411	2.676399027	0.999999723	11	0	411	0	0	15.54545455

323	411	78.58880779	1	323	0	411	0	0	21.93498452
87	411	21.16788321	1	87	0	411	0	0	27.2083215
13	411	3.163017032	1	13	0	411	0	0	32.37986553
25	412	6.067961165	1	25	0	412	0	0	35.01052461
65	412	15.77669903	1	65	0	412	0	0	32.31773218
7	412	1.699029126	1	7	0	412	0	0	32.5306098
181	412	43.93203883	1	181	0	412	0	0	30.84722737
104	412	25.24271845	1	104	0	412	0	0	28.42104743
382	412	92.7184466	1	382	0	412	0	0	35.86387435
22	412	5.339805825	1	22	0	412	0	0	35.18181818
13	82	15.85365854	0.999864887	13	0	82	0	0	16.84615385
68	82	82.92682927	1	68	0	82	0	0	32.42647059
2	82	2.43902439	0.814261998	2	0	82	0	0	14
80	82	97.56097561	1	80	0	82	0	0	26.475
14	82	17.07317073	1	14	0	82	0	0	17.42857143
68	82	82.92682927	1	68	0	82	0	0	32.72058824
14	83	16.86746988	0.999999995	14	0	83	0	0	17.78571429
2	83	2.409638554	0.997186423	2	0	83	0	0	21
66	83	79.51807229	1	66	0	83	0	0	32.66666667
2	83	2.409638554	0.999999998	2	0	83	0	0	51.5
81	83	97.59036145	1	81	0	83	0	0	55.28395062
22	103	21.3592233	1	22	0	103	0	0	59.047338
81	103	78.6407767	1	81	0	103	0	0	47.24122046
2	103	1.941747573	0.980733624	2	0	103	0	0	38
100	103	97.08737864	1	100	0	103	0	0	60.9
2	103	1.941747573	0.828150684	2	0	103	0	0	58.5
101	103	98.05825243	1	101	0	103	0	0	56.9009901
2	103	1.941747573	0.841576342	2	0	103	0	0	47.5
101	103	98.05825243	1	101	0	103	0	0	60.13861386
2	103	1.941747573	0.873458617	2	0	103	0	0	64
101	103	98.05825243	1	101	0	103	0	0	53.57425743
2	103	1.941747573	0.999847637	2	0	103	0	0	64
101	103	98.05825243	1	101	0	103	0	0	52.01980198
97	103	94.17475728	1	97	0	103	0	0	60.88659794
6	103	5.825242718	1	6	0	103	0	0	57
2	103	1.941747573	0.86021547	2	0	103	0	0	36
101	103	98.05825243	1	101	0	103	0	0	53.88118812
1000	1016	98.42519685	1	1000	0	1016	0	0	38.149
15	1016	1.476377953	1	15	0	1016	0	0	36.06666667

Supplementary Table S2 B03_504v Variant Table

Read count	Read coverage	# unique start positions	# unique end positions	BaseQRankSum	Read position test probability	Read direction test probability	Homopolymer length	Homopolymer	QUAL
42	4023	1	1	-1.17	1	1	6	Yes	200
3979	4023	4	4		1	1	6	Yes	200
3974	4023	4	4	3.09	1	1	1	No	200
48	4023	1	2		1	1	1	No	200
860	6141	3	6	-41.71	0	1	1	No	200
5266	6141	8	10		0	1	1	No	200
1354	6141	4	7	-39.78	0	1	1	No	200
4781	6141	7	9		0	1	1	No	200
954	6141	5	4	-24.65	0	1	1	No	200
5177	6141	6	11		0	1	1	No	200
2078	2126	4	7	-0.3	1	1	1	No	200
48	2126	1	1		1	1	1	No	200
2077	2126	4	7	-0.43	1	1	1	No	200
48	2126	1	1		0.884204853	1	1	No	200
2115	2126	4	7		1	1	1	No	200
1625	2126	4	6	25.58	0.870826049	1	7	Yes	200
268	2126	3	2	18.79	0.731759063	1	7	Yes	200
62	2126	1	1	5.84	0.86877514	1	7	Yes	200
163	2126	1	2		0.789348096	1	7	Yes	200
31	2124	1	1	-10.43	0.906095906	1	1	No	200
2069	2124	5	7		1	1	7	Yes	200
2061	2124	5	6	12.13	0.337771639	1	5	Yes	200
62	2124	1	2		3.63273E-06	1	5	Yes	200
49	2166	3	3	0.2	0	1	1	No	200
2116	2166	5	5		7.26809E-09	1	1	No	200
2	43	1	1	-2.16	1	1	1	No	33.34622791
41	43	2	1		1	1	1	No	200
40	43	2	1	0.21	1	1	1	No	200
2	43	1	1		1	1	1	No	200
46	374	1	2	-3.68	0.079993266	1	1	No	200
321	374	3	2		0.364672175	1	1	No	200
42	374	2	2	-8.49	0.746351378	1	1	No	20.11675541
328	374	3	3		0.948435205	1	1	No	200
145	374	2	2	-14.57	0.545330122	1	1	No	200
226	374	3	3		0.739417436	1	1	No	200
27	374	2	2	-8.39	0.796613512	1	1	No	99.99999964
5	374	1	1	-2.36	0.911045537	1	1	No	54.98064813
320	374	3	3		0.974617836	1	1	No	200
21	374	1	2	-4.97	0.967841974	1	1	No	200
24	374	1	1	-5.22	1	1	1	No	51.0965724
349	374	3	3		1	1	1	No	200
99	388	2	2	-11.08	0.066840929	1	1	No	200
282	388	4	5		0.457159381	1	1	No	200
13	388	1	1	-3.21	0.506329224	1	1	No	44.93951149
375	388	4	5		1	1	1	No	200
53	388	2	2	-5.67	0.180712043	1	1	No	200
23	388	2	3	-4.76	0.031356341	1	1	No	33.71849538
309	388	4	4		1	1	1	No	200
76	411	2	2	-8.37	0.027568879	1	1	No	200
324	411	4	5		0.600186741	1	1	No	200
66	411	2	2	-10.72	0.046524464	1	1	No	200
11	411	2	3	-3.98	0.003813524	1	1	No	65.57520231

323	411	4	4		0.38955518	1	1	No	200
87	411	4	3	-15.1	0.030215981	1	4	Yes	200
13	411	3	2	-7.35	0.773582379	1	4	Yes	200
25	412	4	2		0.364024873	1	5	Yes	200
65	412	3	2	-14.53	0.049086425	1	5	Yes	200
7	412	1	2	-8.58	0.715661104	1	5	Yes	200
181	412	3	3	-16.17	0.657533223	1	1	No	200
104	412	4	4	-16.58	0.053546216	1	1	No	200
382	412	5	5	0.88	0.993974802	1	1	No	200
22	412	3	2		0.975435365	1	1	No	200
13	82	2	1	-4.88	1	1	1	No	38.69303184
68	82	9	2		1	1	1	No	200
2	82	2	1	-1.47	1	1	1	No	7.310992314
80	82	9	2		1	1	1	No	200
14	82	2	1	-4.57	1	1	1	No	200
68	82	9	2		1	1	1	No	200
14	83	2	1	-4.65	1	1	1	No	83.18758767
2	83	2	1	-1.78	1	1	1	No	25.50741149
66	83	9	2		1	1	1	No	200
2	83	1	1	-0.43	0.150575259	1	1	No	86.57577327
81	83	10	2		1	1	1	No	200
22	103	2	1	3.02	6.70897E-12	1	1	No	200
81	103	10	2		0.000241482	1	1	No	200
2	103	1	1	-1.93	0.440158768	1	1	No	17.15199968
100	103	10	2		1	1	1	No	200
2	103	1	1	0.01	0.506682578	1	1	No	7.648521909
101	103	11	2		1	1	1	No	200
2	103	1	1	-0.8	0.506682578	1	1	No	8.001799639
101	103	11	2		1	1	1	No	200
2	103	1	1	1.05	0.506682578	1	1	No	8.97767423
101	103	11	2		1	1	1	No	200
2	103	1	1	1.08	0.506682578	1	1	No	38.1712077
101	103	11	2		1	1	1	No	200
97	103	11	2	0.3	0.995286712	1	1	No	200
6	103	3	1		0.687652585	1	1	No	200
2	103	1	1	-1.65	0.506682578	1	1	No	8.545408888
101	103	11	2		1	1	1	No	200
1000	1016	6	7	0.87	1	1	1	No	200
15	1016	1	2		1	1	1	No	200

Supplementary Table S2 B04_505v Variant Table

Reference mitogenome	Region	Type	Reference	Allele	Reference allele	Length	Linkage	Zygosity
NC_012920_rCRS H2a_haplo	64	SNV	C	T	No	1		Heterozygous
NC_012920_rCRS H2a_haplo	64	SNV	C	C	Yes	1		Heterozygous
NC_012920_rCRS H2a_haplo	73	SNV	A	G	No	1		Homozygous
NC_012920_rCRS H2a_haplo	146	SNV	T	C	No	1		Heterozygous
NC_012920_rCRS H2a_haplo	146	SNV	T	T	Yes	1		Heterozygous
NC_012920_rCRS H2a_haplo	150	SNV	C	T	No	1		Heterozygous
NC_012920_rCRS H2a_haplo	150	SNV	C	C	Yes	1		Heterozygous
NC_012920_rCRS H2a_haplo	152	SNV	T	C	No	1		Heterozygous
NC_012920_rCRS H2a_haplo	152	SNV	T	T	Yes	1		Heterozygous
NC_012920_rCRS H2a_haplo	235	SNV	A	G	No	1		Homozygous
NC_012920_rCRS H2a_haplo	263	SNV	A	G	No	1		Homozygous
NC_012920_rCRS H2a_haplo	302^303	Insertion	-	C	No	1		Heterozygous
NC_012920_rCRS H2a_haplo	302^303	Insertion	-	CC	No	2		Heterozygous
NC_012920_rCRS H2a_haplo	302^303	Insertion	-	-	Yes	0		Heterozygous
NC_012920_rCRS H2a_haplo	310^311	Insertion	-	C	No	1		Heterozygous
NC_012920_rCRS H2a_haplo	310^311	Insertion	-	-	Yes	0		Heterozygous
NC_012920_rCRS H2a_haplo	351	SNV	A	G	No	1		Heterozygous
NC_012920_rCRS H2a_haplo	351	SNV	A	A	Yes	1		Heterozygous
NC_012920_rCRS H2a_haplo	514..515	Deletion	CA	-	No	2		Homozygous
NC_012920_rCRS H2a_haplo	16111	SNV	C	T	No	1		Homozygous
NC_012920_rCRS H2a_haplo	16172	SNV	T	C	No	1		Heterozygous
NC_012920_rCRS H2a_haplo	16172	SNV	T	T	Yes	1		Heterozygous
NC_012920_rCRS H2a_haplo	16223	SNV	C	T	No	1		Homozygous
NC_012920_rCRS H2a_haplo	16263	SNV	T	A	No	1		Heterozygous
NC_012920_rCRS H2a_haplo	16263	SNV	T	T	Yes	1		Heterozygous
NC_012920_rCRS H2a_haplo	16290	SNV	C	T	No	1		Homozygous
NC_012920_rCRS H2a_haplo	16319	SNV	G	A	No	1		Homozygous
NC_012920_rCRS H2a_haplo	16362	SNV	T	C	No	1		Heterozygous
NC_012920_rCRS H2a_haplo	16362	SNV	T	T	Yes	1		Heterozygous
NC_012920_rCRS H2a_haplo	16396	SNV	T	G	No	1		Heterozygous
NC_012920_rCRS H2a_haplo	16396	SNV	T	T	Yes	1		Heterozygous

Supplementary Table S2 B04_505v Variant Table

**Count**	**Coverage**	**Frequency**	Probability	Forward read count	Reverse read count	Forward read coverage	Reverse read coverage	Forward/reverse balance	Average quality
3933	4003	98.25131152	1	3933	0	4003	0	0	62.93643529
54	4003	1.348988259	1	54	0	4003	0	0	46.07407407
3979	4003	99.40044966	1	3979	0	4003	0	0	63.37245539
4644	5872	79.08719346	1	4644	0	5872	0	0	36.15719208
1210	5872	20.60626703	1	1210	0	5872	0	0	36.30413223
1178	5874	20.05447736	1	1178	0	5874	0	0	36.41341256
4693	5874	79.89445012	1	4693	0	5874	0	0	38.09673983
838	5875	14.26382979	1	838	0	5875	0	0	36.87589499
5031	5875	85.63404255	1	5031	0	5875	0	0	37.95646989
1881	1885	99.78779841	1	1881	0	1885	0	0	63.19883041
1883	1885	99.8938992	1	1883	0	1885	0	0	62.82421668
1623	1884	86.14649682	1	1623	0	1884	0	0	35.76586568
92	1884	4.883227176	1	92	0	1884	0	0	34.30978261
163	1884	8.651804671	1	163	0	1884	0	0	31.06134969
1852	1884	98.3014862	1	1852	0	1884	0	0	37.48704104
31	1884	1.645435244	1	31	0	1884	0	0	31.51612903
20	1901	1.052077854	1	20	0	1901	0	0	36.1
1878	1901	98.79011047	1	1878	0	1901	0	0	36.69062833
19	19	100	1	19	0	19	0	0	33.26315789
688	690	99.71014493	1	688	0	690	0	0	63.50726744
13	745	1.744966443	1	13	0	745	0	0	37.53846154
732	745	98.25503356	1	732	0	745	0	0	37.67759563
288	289	99.65397924	1	288	0	289	0	0	38.26388889
27	318	8.490566038	1	27	0	318	0	0	61.96296296
291	318	91.50943396	1	291	0	318	0	0	63.24742268
317	318	99.68553459	1	317	0	318	0	0	62.86435331
317	318	99.68553459	1	317	0	318	0	0	40.50473186
316	1112	28.41726619	1	316	0	1112	0	0	39.72151899
795	1112	71.49280576	1	795	0	1112	0	0	36.95974843
25	1114	2.244165171	0.970937696	25	0	1114	0	0	19
1089	1114	97.75583483	1	1089	0	1114	0	0	36.30486685

Supplementary Table S2 B04_505v Variant Table

Read count	Read coverage	# unique start positions	# unique end positions	BaseQRankSum	Read position test probability	Read direction test probability	Homopolymer	Homopolymer length	QUAL
3933	4003	4	5	4.68	1	1	No	1	200
54	4003	2	1		1	1	No	1	200
3979	4003	4	5		1	1	No	1	200
4644	5872	6	9	20.55	0	1	No	1	200
1210	5872	5	7		0	1	No	1	200
1178	5874	5	6	-40.1	0	1	No	1	200
4693	5874	7	11		0	1	No	1	200
838	5875	6	5	-25.87	0	1	No	1	200
5031	5875	7	12		0	1	No	1	200
1881	1885	5	8		1	1	No	1	200
1883	1885	5	8		1	1	No	1	200
1623	1884	5	8	20.64	0.912876884	1	Yes	7	200
92	1884	1	2	8.31	0.762890547	1	Yes	7	200
163	1884	3	2		0.688149915	1	Yes	7	200
1852	1884	4	6	6.4	0.331313385	1	Yes	5	200
31	1884	2	2		6.12759E-08	1	Yes	5	200
20	1901	3	2	-0.21	0	1	No	1	200
1878	1901	5	6		0.000122039	1	No	1	200
19	19	2	1		1	1	No	1	200
688	690	3	2		1	1	No	1	200
13	745	2	1	-3.11	0	1	No	1	200
732	745	5	4		0.300041798	1	No	1	200
288	289	6	5		1	1	No	1	200
27	318	1	1	-1.7	0	1	No	1	200
291	318	7	5		3.4371E-05	1	No	1	200
317	318	8	5		1	1	No	1	200
317	318	8	5		1	1	No	1	200
316	1112	8	4	6.79	0	1	No	1	200
795	1112	4	5		0	1	No	1	200
25	1114	3	1	-7.8	2.92877E-13	1	No	1	15.36669954
1089	1114	11	8		0.97032052	1	No	1	200

Supplementary Table S2 B05_507v Variant Table

Reference mitogenome	Region	Type	Reference	Allele	Reference allele	Length	Linkage	Zygosity
NC_012920_rCRS H2a_haplo	146	SNV	T	C	No	1		Heterozygous
NC_012920_rCRS H2a_haplo	146	SNV	T	T	Yes	1		Heterozygous
NC_012920_rCRS H2a_haplo	150	SNV	C	T	No	1		Heterozygous
NC_012920_rCRS H2a_haplo	150	SNV	C	C	Yes	1		Heterozygous
NC_012920_rCRS H2a_haplo	152	SNV	T	C	No	1		Heterozygous
NC_012920_rCRS H2a_haplo	152	SNV	T	T	Yes	1		Heterozygous
NC_012920_rCRS H2a_haplo	199	SNV	T	C	No	1		Homozygous
NC_012920_rCRS H2a_haplo	263	SNV	A	G	No	1		Homozygous
NC_012920_rCRS H2a_haplo	302^303	Insertion	-	C	No	1		Heterozygous
NC_012920_rCRS H2a_haplo	302^303	Insertion	-	-	Yes	0		Heterozygous
NC_012920_rCRS H2a_haplo	310	SNV	T	C	No	1		Heterozygous
NC_012920_rCRS H2a_haplo	310	SNV	T	T	Yes	1		Heterozygous
NC_012920_rCRS H2a_haplo	310^311	Insertion	-	C	No	1		Heterozygous
NC_012920_rCRS H2a_haplo	310^311	Insertion	-	-	Yes	0		Heterozygous
NC_012920_rCRS H2a_haplo	16172	SNV	T	C	No	1		Heterozygous
NC_012920_rCRS H2a_haplo	16172	SNV	T	T	Yes	1		Heterozygous
NC_012920_rCRS H2a_haplo	16263	SNV	T	A	No	1		Heterozygous
NC_012920_rCRS H2a_haplo	16263	SNV	T	T	Yes	1		Heterozygous
NC_012920_rCRS H2a_haplo	16519	SNV	T	C	No	1		Homozygous

Supplementary Table S2 B05_507v Variant Table

**Count**	**Coverage**	**Frequency**	Probability	Forward read count	Reverse read count	Forward read coverage	Reverse read coverage	Forward/reverse balance	Average quality
905	7795	11.61000641	1	905	0	7795	0	0	32.78121547
6867	7795	88.09493265	1	6867	0	7795	0	0	37.22120285
1571	7795	20.15394484	1	1571	0	7795	0	0	36.4665818
6220	7795	79.79474022	1	6220	0	7795	0	0	37.7511254
6281	7795	80.57729314	1	6281	0	7795	0	0	37.85416335
1504	7795	19.2944195	1	1504	0	7795	0	0	37.15425532
2510	2520	99.6031746	1	2510	0	2520	0	0	37.64701195
2516	2520	99.84126984	1	2516	0	2520	0	0	62.91096979
54	2520	2.142857143	1	54	0	2520	0	0	33.77777778
2466	2520	97.85714286	1	2466	0	2520	0	0	30.2757502
53	2520	2.103174603	1	53	0	2520	0	0	34.2235354
2437	2520	96.70634921	1	2437	0	2520	0	0	25.64820165
2427	2520	96.30952381	1	2427	0	2520	0	0	37.01194988
93	2520	3.69047619	1	93	0	2520	0	0	31.6345177
19	856	2.219626168	1	19	0	856	0	0	35.36842105
836	856	97.6635514	1	836	0	856	0	0	36.74043062
45	361	12.46537396	1	45	0	361	0	0	62.75555556
316	361	87.53462604	1	316	0	361	0	0	62.64556962
1230	1234	99.67585089	1	1230	0	1234	0	0	37.61382114

Supplementary Table S2 B05_507v Variant Table

Read count	Read coverage	# unique start positions	# unique end positions	BaseQRankSum	Read position test probability	Read direction test probability	Homopolymer	Homopolymer length	QUAL
905	7795	3	6	-43.15	0	1	No	1	200
6867	7795	9	10		0	1	No	1	200
1571	7795	3	6	-42.75	0	1	No	1	200
6220	7795	9	10		0	1	No	1	200
6281	7795	9	11	34.14	0	1	No	1	200
1504	7795	3	5		0	1	No	1	200
2510	2520	4	7		1	1	No	1	200
2516	2520	4	7		1	1	No	1	200
54	2520	2	2	3.38	0.00016639	1	Yes	7	200
2466	2520	4	6		0.600288619	1	Yes	7	200
53	2520	2	2	-1.31	1	1	No	1	200
2437	2520	4	6		1	1	Yes	7	200
2427	2520	4	5	7.54	1	1	Yes	5	200
93	2520	2	3		1	1	Yes	5	200
19	856	4	2	-2.91	0	1	No	1	200
836	856	5	6		0.297780138	1	No	1	200
45	361	1	2	-0.3	0	1	No	1	200
316	361	11	2		2.01827E-08	1	No	1	200
1230	1234	7	2		1	1	No	1	200

Supplementary Table S2 B06_508v Variant Table

Reference mitogenome	Region	Type	Reference	Allele	Reference allele	Length	Linkage	Zygosity
NC_012920_rCRS H2a_haplo	64	SNV	C	T	No	1		Homozygous
NC_012920_rCRS H2a_haplo	73	SNV	A	G	No	1		Homozygous
NC_012920_rCRS H2a_haplo	146	SNV	T	C	No	1		Heterozygous
NC_012920_rCRS H2a_haplo	146	SNV	T	T	Yes	1		Heterozygous
NC_012920_rCRS H2a_haplo	150	SNV	C	T	No	1		Heterozygous
NC_012920_rCRS H2a_haplo	150	SNV	C	C	Yes	1		Heterozygous
NC_012920_rCRS H2a_haplo	152	SNV	T	C	No	1		Heterozygous
NC_012920_rCRS H2a_haplo	152..153	MNV	TA	TA	Yes	2		Heterozygous
NC_012920_rCRS H2a_haplo	153	SNV	A	G	No	1		Heterozygous
NC_012920_rCRS H2a_haplo	235	SNV	A	G	No	1		Homozygous
NC_012920_rCRS H2a_haplo	263	SNV	A	G	No	1		Homozygous
NC_012920_rCRS H2a_haplo	302^303	Insertion	-	C	No	1		Heterozygous
NC_012920_rCRS H2a_haplo	302^303	Insertion	-	CC	No	2		Heterozygous
NC_012920_rCRS H2a_haplo	302^303	Insertion	-	-	Yes	0		Heterozygous
NC_012920_rCRS H2a_haplo	310	SNV	T	C	No	1		Heterozygous
NC_012920_rCRS H2a_haplo	310	SNV	T	T	Yes	1		Heterozygous
NC_012920_rCRS H2a_haplo	310^311	Insertion	-	C	No	1		Heterozygous
NC_012920_rCRS H2a_haplo	310^311	Insertion	-	-	Yes	0		Heterozygous
NC_012920_rCRS H2a_haplo	351	SNV	A	G	No	1		Heterozygous
NC_012920_rCRS H2a_haplo	351	SNV	A	A	Yes	1		Heterozygous
NC_012920_rCRS H2a_haplo	514..515	Deletion	CA	-	No	2		Homozygous
NC_012920_rCRS H2a_haplo	16095	SNV	C	T	No	1		Homozygous
NC_012920_rCRS H2a_haplo	16111	SNV	C	T	No	1		Homozygous
NC_012920_rCRS H2a_haplo	16172	SNV	T	C	No	1		Heterozygous
NC_012920_rCRS H2a_haplo	16172	SNV	T	T	Yes	1		Heterozygous
NC_012920_rCRS H2a_haplo	16223	SNV	C	T	No	1		Homozygous
NC_012920_rCRS H2a_haplo	16263	SNV	T	A	No	1		Heterozygous
NC_012920_rCRS H2a_haplo	16263	SNV	T	T	Yes	1		Heterozygous
NC_012920_rCRS H2a_haplo	16290	SNV	C	T	No	1		Homozygous
NC_012920_rCRS H2a_haplo	16319	SNV	G	A	No	1		Homozygous
NC_012920_rCRS H2a_haplo	16362	SNV	T	C	No	1		Heterozygous
NC_012920_rCRS H2a_haplo	16362	SNV	T	T	Yes	1		Heterozygous
NC_012920_rCRS H2a_haplo	16396	SNV	T	G	No	1		Heterozygous
NC_012920_rCRS H2a_haplo	16396	SNV	T	T	Yes	1		Heterozygous

**Count**	**Coverage**	**Frequency**	Probability	Forward read count	Reverse read count	Forward read coverage	Reverse read coverage	Forward/reverse balance	Average quality
5699	5775	98.68398268	1	5699	0	5775	0	0	62.7908405
5745	5773	99.51498354	1	5745	0	5773	0	0	63.44995648
6231	7001	89.0015712	1	6231	0	7001	0	0	36.23832451
761	7001	10.86987573	1	761	0	7001	0	0	35.99211564
735	7002	10.49700086	1	735	0	7002	0	0	36.25986395
6263	7002	89.44587261	1	6263	0	7002	0	0	38.07616158
526	7002	7.512139389	1	526	0	7002	0	0	36.77743693
779	7002	11.12539274	1	779	0	7002	0	0	36.45603921
5691	7002	81.27677806	1	5691	0	7002	0	0	37.58558679
1232	1234	99.83792545	1	1232	0	1234	0	0	63.15422078
1233	1234	99.91896272	1	1233	0	1234	0	0	62.65044607
1061	1234	85.98055105	1	1061	0	1234	0	0	35.65409991
73	1234	5.915721232	1	73	0	1234	0	0	34.57534247
88	1234	7.131280389	1	88	0	1234	0	0	30.84090909
15	1234	1.215559157	1	15	0	1234	0	0	32.8
1219	1234	98.78444084	1	1219	0	1234	0	0	26.84003281
1214	1234	98.37925446	1	1214	0	1234	0	0	37.50576606
20	1234	1.620745543	1	20	0	1234	0	0	32.6
39	1276	3.056426332	1	39	0	1276	0	0	35.76923077
1236	1276	96.86520376	1	1236	0	1276	0	0	36.29368932
39	41	95.12195122	1	39	0	41	0	0	35.05128205
902	908	99.33920705	1	902	0	908	0	0	63.77383592
903	908	99.44933921	1	903	0	908	0	0	63.58803987
23	1027	2.239532619	1	23	0	1027	0	0	37.7826087
1003	1027	97.6630964	1	1003	0	1027	0	0	37.35094716
550	554	99.27797834	1	550	0	554	0	0	37.90545455
43	601	7.154742097	1	43	0	601	0	0	63.25581395
558	601	92.8452579	1	558	0	601	0	0	63.39964158
599	601	99.6672213	1	599	0	601	0	0	62.60267112
595	601	99.00166389	1	595	0	601	0	0	40.42352941
595	1691	35.18628031	1	595	0	1691	0	0	39.02689076
1096	1691	64.81371969	1	1096	0	1691	0	0	36.72080292
48	1692	2.836879433	0.999778192	48	0	1692	0	0	18.1875
1641	1692	96.9858156	1	1641	0	1692	0	0	35.6812919

Supplementary Table S2 B06_508v Variant Table

Read count	Read coverage	# unique start positions	# unique end positions	BaseQRankSum	Read position test probability	Read direction test probability	Homopolymer	Homopolymer length	QUAL
5699	5775	7	8		1	1	No	1	200
5745	5773	7	6		1	1	No	1	200
6231	7001	8	10	21.11	0	1	No	1	200
761	7001	5	3		0	1	No	1	200
735	7002	4	6	-34.51	0	1	No	1	200
6263	7002	11	7		0	1	No	1	200
526	7002	5	3	1.43	0	1	No	1	200
779	7002	4	5		0	1	No	1	200
5691	7002	7	6	48.01	0	1	No	1	200
1232	1234	5	4		1	1	No	1	200
1233	1234	5	4		1	1	No	1	200
1061	1234	4	4	16.51	0.937243833	1	Yes	7	200
73	1234	2	1	7.99	0.814433527	1	Yes	7	200
88	1234	2	2		0.796671021	1	Yes	7	200
15	1234	1	1	4.1	1	1	No	1	200
1219	1234	5	5		1	1	Yes	7	200
1214	1234	5	5	4.8	1	1	Yes	5	200
20	1234	1	1		1	1	Yes	5	200
39	1276	2	3	0.68	0	1	No	1	200
1236	1276	6	5		1.79669E-07	1	No	1	200
39	41	2	1		1	1	No	1	200
902	908	3	3		1	1	No	1	200
903	908	3	3		1	1	No	1	200
23	1027	3	2	-3.06	0	1	No	1	200
1003	1027	6	5		0.588743989	1	No	1	200
550	554	11	2		1	1	No	1	200
43	601	3	1	-0.82	0	1	No	1	200
558	601	13	2		1.19356E-07	1	No	1	200
599	601	16	2		1	1	No	1	200
595	601	16	2	-0.93	1	1	No	1	200
595	1691	16	2	8.55	0	1	No	1	200
1096	1691	8	3		0	1	No	1	200
48	1692	7	1	-10.51	0	1	No	1	36.54023186
1641	1692	20	4		0.950051911	1	No	1	200

Supplementary Table S2 B07_509v Variant Table

Reference mitogenome	Region	Type	Reference	Allele	Reference allele	Length	Linkage	Zygosity
NC_012920_rCRS H2a_haplo	64	SNV	C	T	No	1		Homozygous
NC_012920_rCRS H2a_haplo	93	SNV	A	G	No	1		Heterozygous
NC_012920_rCRS H2a_haplo	93	SNV	A	A	Yes	1		Heterozygous
NC_012920_rCRS H2a_haplo	95	SNV	A	C	No	1		Heterozygous
NC_012920_rCRS H2a_haplo	95	SNV	A	A	Yes	1		Heterozygous
NC_012920_rCRS H2a_haplo	146	SNV	T	C	No	1		Heterozygous
NC_012920_rCRS H2a_haplo	146	SNV	T	T	Yes	1		Heterozygous
NC_012920_rCRS H2a_haplo	150	SNV	C	T	No	1		Heterozygous
NC_012920_rCRS H2a_haplo	150	SNV	C	C	Yes	1		Heterozygous
NC_012920_rCRS H2a_haplo	152	SNV	T	C	No	1		Heterozygous
NC_012920_rCRS H2a_haplo	152	SNV	T	T	Yes	1		Heterozygous
NC_012920_rCRS H2a_haplo	185	SNV	G	A	No	1		Heterozygous
NC_012920_rCRS H2a_haplo	185	SNV	G	G	Yes	1		Heterozygous
NC_012920_rCRS H2a_haplo	189	SNV	A	G	No	1		Homozygous
NC_012920_rCRS H2a_haplo	200	SNV	A	G	No	1		Homozygous
NC_012920_rCRS H2a_haplo	236	SNV	T	C	No	1		Homozygous
NC_012920_rCRS H2a_haplo	247	SNV	G	A	No	1		Homozygous
NC_012920_rCRS H2a_haplo	263	SNV	A	G	No	1		Homozygous
NC_012920_rCRS H2a_haplo	302^303	Insertion	-	C	No	1		Heterozygous
NC_012920_rCRS H2a_haplo	302^303	Insertion	-	-	Yes	0		Heterozygous
NC_012920_rCRS H2a_haplo	310	SNV	T	C	No	1		Heterozygous
NC_012920_rCRS H2a_haplo	310	SNV	T	T	Yes	1		Heterozygous
NC_012920_rCRS H2a_haplo	310^311	Insertion	-	C	No	1		Heterozygous
NC_012920_rCRS H2a_haplo	310^311	Insertion	-	-	Yes	0		Heterozygous
NC_012920_rCRS H2a_haplo	351	SNV	A	G	No	1		Heterozygous
NC_012920_rCRS H2a_haplo	351	SNV	A	A	Yes	1		Heterozygous
NC_012920_rCRS H2a_haplo	410	SNV	G	T	No	1		Heterozygous
NC_012920_rCRS H2a_haplo	410	SNV	G	G	Yes	1		Heterozygous
NC_012920_rCRS H2a_haplo	514..515	Deletion	CA	-	No	2		Homozygous
NC_012920_rCRS H2a_haplo	16129	SNV	G	A	No	1		Homozygous
NC_012920_rCRS H2a_haplo	16148	SNV	C	T	No	1		Homozygous
NC_012920_rCRS H2a_haplo	16168	SNV	C	T	No	1		Heterozygous
NC_012920_rCRS H2a_haplo	16168	SNV	C	C	Yes	1		Heterozygous
NC_012920_rCRS H2a_haplo	16172	SNV	T	C	No	1		Heterozygous
NC_012920_rCRS H2a_haplo	16172	SNV	T	T	Yes	1		Heterozygous
NC_012920_rCRS H2a_haplo	16187..16189	MNV	CCT	TGC	No	3		Homozygous
NC_012920_rCRS H2a_haplo	16223	SNV	C	T	No	1		Homozygous
NC_012920_rCRS H2a_haplo	16230	SNV	A	G	No	1		Homozygous
NC_012920_rCRS H2a_haplo	16263	SNV	T	A	No	1		Heterozygous
NC_012920_rCRS H2a_haplo	16263	SNV	T	T	Yes	1		Heterozygous
NC_012920_rCRS H2a_haplo	16311	SNV	T	C	No	1		Homozygous
NC_012920_rCRS H2a_haplo	16320	SNV	C	T	No	1		Homozygous
NC_012920_rCRS H2a_haplo	16396	SNV	T	G	No	1		Heterozygous
NC_012920_rCRS H2a_haplo	16396	SNV	T	T	Yes	1		Heterozygous

Supplementary Table S2 B07_509v Variant Table

**Count**	**Coverage**	**Frequency**	Probability	Forward read count	Reverse read count	Forward read coverage	Reverse read coverage	Forward/reverse balance	Average quality
4243	4303	98.60562398	1	4243	0	4303	0	0	62.69172755
4236	4303	98.44294678	1	4236	0	4303	0	0	62.32932011
63	4303	1.464094818	1	63	0	4303	0	0	42.84126984
4233	4303	98.37322798	1	4233	0	4303	0	0	62.35270494
69	4303	1.603532419	1	69	0	4303	0	0	40.92753623
865	6570	13.16590563	1	865	0	6570	0	0	32.7017341
5687	6570	86.56012177	1	5687	0	6570	0	0	37.41164058
1419	6570	21.59817352	1	1419	0	6570	0	0	36.40239605
5143	6570	78.28006088	1	5143	0	6570	0	0	38.07310908
1022	6572	15.55082167	1	1022	0	6572	0	0	37.2407045
5546	6572	84.38831406	1	5546	0	6572	0	0	37.88892896
2245	6544	34.30623472	1	2245	0	6544	0	0	35.90868597
4282	6544	65.43398533	1	4282	0	6544	0	0	32.84002802
2231	2277	97.97979798	1	2231	0	2277	0	0	34.07799193
2260	2277	99.2534036	1	2260	0	2277	0	0	37.64690265
2269	2277	99.64866052	1	2269	0	2277	0	0	63.38607316
2268	2277	99.60474308	1	2268	0	2277	0	0	63.80070547
2277	2277	100	1	2277	0	2277	0	0	63.12209047
121	2277	5.314009662	1	121	0	2277	0	0	35.38016529
2156	2277	94.68599034	1	2156	0	2277	0	0	30.9025974
50	2277	2.195871761	1	50	0	2277	0	0	33.2
2227	2277	97.80412824	1	2227	0	2277	0	0	26.79838348
2218	2277	97.40887132	1	2218	0	2277	0	0	37.45085663
59	2277	2.591128678	1	59	0	2277	0	0	32.16949153
37	2313	1.599654129	1	37	0	2313	0	0	35.72972973
2274	2313	98.31387808	1	2274	0	2313	0	0	36.44195251
2	36	5.555555556	0.937169347	2	0	36	0	0	24.5
33	36	91.66666667	1	33	0	36	0	0	33.3030303
35	36	97.22222222	1	35	0	36	0	0	34.94285714
728	728	100	1	728	0	728	0	0	63.71016484
725	728	99.58791209	1	725	0	728	0	0	37.59448276
723	779	92.81129653	1	723	0	779	0	0	36.71922545
55	779	7.060333761	1	55	0	779	0	0	31.10909091
737	779	94.6084724	1	737	0	779	0	0	36.85888738
42	779	5.391527599	1	42	0	779	0	0	36.5
1116	1125	99.2	1	1116	0	1125	0	0	36.48718701
580	585	99.14529915	1	580	0	585	0	0	38.19655172
575	585	98.29059829	1	575	0	585	0	0	36.36695652
21	609	3.448275862	1	21	0	609	0	0	64
588	609	96.55172414	1	588	0	609	0	0	63.1207483
609	610	99.83606557	1	609	0	610	0	0	47.1954023
597	610	97.86885246	1	597	0	610	0	0	43.40033501
49	1902	2.576235542	0.999996713	49	0	1902	0	0	18.24489796
1841	1902	96.79284963	1	1841	0	1902	0	0	35.74144487

Read count	Read coverage	# unique start positions	# unique end positions	BaseQRankSum	Read position test probability	Read direction test probability	Homopolymer	Homopolymer length	QUAL
4243	4303	5	7	4.06	1	1	No	1	200
4236	4303	5	7	4.84	1	1	No	1	200
63	4303	1	1		0.90628861	1	No	1	200
4233	4303	5	6	6.04	0.733648269	1	No	1	200
69	4303	1	1		6.2385E-06	1	No	1	200
865	6570	3	7	-42.68	0	1	No	1	200
5687	6570	9	10		0	1	No	1	200
1419	6570	4	5	-42.56	0	1	No	1	200
5143	6570	8	11		0	1	No	1	200
1022	6572	5	5	-26.52	0	1	No	1	200
5546	6572	7	12		0	1	No	1	200
2245	6544	4	5	48	0	1	No	1	200
4282	6544	6	7		0	1	No	1	200
2231	2277	4	5		1	1	No	1	200
2260	2277	4	5		1	1	No	1	200
2269	2277	4	5		1	1	No	1	200
2268	2277	4	5		1	1	No	1	200
2277	2277	4	5		1	1	No	1	200
121	2277	2	3	9.29	0.753024785	1	Yes	7	200
2156	2277	4	4		0.988298233	1	Yes	7	200
50	2277	1	3	7.33	1.13483E-08	1	No	1	200
2227	2277	5	4		0.337413308	1	Yes	7	200
2218	2277	5	4	6.26	0.338405568	1	Yes	5	200
59	2277	1	3		1.66402E-07	1	Yes	5	200
37	2313	3	5	0.91	0	1	No	1	200
2274	2313	5	6		1.50084E-07	1	No	1	200
2	36	1	1	-1.46	1	1	No	1	12.01828424
33	36	2	3		1	1	No	1	200
35	36	2	3		1	1	No	1	200
728	728	3	6		1	1	No	1	200
725	728	3	6		1	1	No	1	200
723	779	3	6	9.7	1.21002E-10	1	No	1	200
55	779	4	2		0	1	No	1	200
737	779	4	7	0.1	2.70951E-06	1	No	1	200
42	779	3	1		0	1	No	1	200
1116	1125	10	8		1	1	No	1	200
580	585	11	3		1	1	No	1	200
575	585	11	3		1	1	No	1	200
21	609	1	1	0.5	0	1	No	1	200
588	609	13	3		0.00061145	1	No	1	200
609	610	16	3		1	1	No	1	200
597	610	16	3		1	1	No	1	200
49	1902	6	1	-10.73	0	1	No	1	54.83173872
1841	1902	19	7		0.864902125	1	No	1	200

Supplementary Table S2 B08_511v Variant Table

Reference mitogenome	Region	Type	Reference	Allele	Reference allele	Length	Linkage	Zygosity
NC_012920_rCRS H2a_haplo	146	SNV	T	C	No	1		Heterozygous
NC_012920_rCRS H2a_haplo	146	SNV	T	T	Yes	1		Heterozygous
NC_012920_rCRS H2a_haplo	150	SNV	C	T	No	1		Heterozygous
NC_012920_rCRS H2a_haplo	150	SNV	C	C	Yes	1		Heterozygous
NC_012920_rCRS H2a_haplo	152	SNV	T	C	No	1		Heterozygous
NC_012920_rCRS H2a_haplo	152	SNV	T	T	Yes	1		Heterozygous
NC_012920_rCRS H2a_haplo	263	SNV	A	G	No	1		Homozygous
NC_012920_rCRS H2a_haplo	302^303	Insertion	-	C	No	1		Heterozygous
NC_012920_rCRS H2a_haplo	302^303	Insertion	-	-	Yes	0		Heterozygous
NC_012920_rCRS H2a_haplo	310	SNV	T	C	No	1		Heterozygous
NC_012920_rCRS H2a_haplo	310	SNV	T	T	Yes	1		Heterozygous
NC_012920_rCRS H2a_haplo	310^311	Insertion	-	C	No	1		Heterozygous
NC_012920_rCRS H2a_haplo	310^311	Insertion	-	-	Yes	0		Heterozygous
NC_012920_rCRS H2a_haplo	16037	SNV	A	G	No	1		Homozygous
NC_012920_rCRS H2a_haplo	16172	SNV	T	C	No	1		Heterozygous
NC_012920_rCRS H2a_haplo	16172	SNV	T	T	Yes	1		Heterozygous
NC_012920_rCRS H2a_haplo	16263	SNV	T	A	No	1		Heterozygous
NC_012920_rCRS H2a_haplo	16263	SNV	T	T	Yes	1		Heterozygous

Supplementary Table S2 B08_511v Variant Table

**Count**	**Coverage**	**Frequency**	Probability	Forward read count	Reverse read count	Forward read coverage	Reverse read coverage	Forward/reverse balance	Average quality
760	6170	12.31766613	1	760	0	6170	0	0	32.76447368
5398	6170	87.48784441	1	5398	0	6170	0	0	37.45942942
1297	6168	21.02788586	1	1297	0	6168	0	0	36.37856592
4864	6168	78.85862516	1	4864	0	6168	0	0	38.10176809
4877	6168	79.0693904	1	4877	0	6168	0	0	37.98421161
1286	6168	20.84954604	1	1286	0	6168	0	0	37.062986
2049	2049	100	1	2049	0	2049	0	0	63.01903367
50	2048	2.44140625	1	50	0	2048	0	0	34.48
1998	2048	97.55859375	1	1998	0	2048	0	0	31.03303303
38	2047	1.856375183	1	38	0	2047	0	0	33.34210526
2009	2047	98.14362482	1	2009	0	2047	0	0	27.38825286
1999	2047	97.65510503	1	1999	0	2047	0	0	37.47138301
48	2047	2.344894968	1	48	0	2047	0	0	32.32843451
684	692	98.84393064	1	684	0	692	0	0	35.6374269
24	791	3.034134008	1	24	0	791	0	0	34.91666667
766	791	96.83944374	1	766	0	791	0	0	37.46344648
51	550	9.272727273	1	51	0	550	0	0	62.64705882
499	550	90.72727273	1	499	0	550	0	0	62.99398798

Supplementary Table S2 B08_511v Variant Table

Read count	Read coverage	# unique start positions	# unique end positions	BaseQRankSum	Read position test probability	Read direction test probability	Homopolymer	Homopolymer length	QUAL
760	6170	3	5	-40.1	0	1	No	1	200
5398	6170	9	15		0	1	No	1	200
1297	6168	5	9	-41.77	0	1	No	1	200
4864	6168	10	11		0	1	No	1	200
4877	6168	10	12	33.49	0	1	No	1	200
1286	6168	4	8		0	1	No	1	200
2049	2049	6	9		1	1	No	1	200
50	2048	1	2	4.53	0.830512498	1	Yes	7	200
1998	2048	6	8		1	1	Yes	7	200
38	2047	2	3	5.83	1	1	No	1	200
2009	2047	6	5		1	1	Yes	7	200
1999	2047	6	5	6.72	1	1	Yes	5	200
48	2047	2	3		1	1	Yes	5	200
684	692	3	4	0.16	1	1	No	1	200
24	791	3	3	-4	0	1	No	1	200
766	791	8	7		0.308574138	1	No	1	200
51	550	2	3	-0.67	0	1	No	1	200
499	550	12	4		1.55217E-09	1	No	1	200

Supplementary Table S2 B09_512v Variant Table

Reference mitogenome	Region	Type	Reference	Allele	Reference allele	Length	Linkage	Zygosity
NC_012920_rCRS H2a_haplo	72	SNV	T	C	No	1		Homozygous
NC_012920_rCRS H2a_haplo	93	SNV	A	G	No	1		Homozygous
NC_012920_rCRS H2a_haplo	146	SNV	T	C	No	1		Heterozygous
NC_012920_rCRS H2a_haplo	146	SNV	T	T	Yes	1		Heterozygous
NC_012920_rCRS H2a_haplo	150	SNV	C	T	No	1		Heterozygous
NC_012920_rCRS H2a_haplo	150	SNV	C	C	Yes	1		Heterozygous
NC_012920_rCRS H2a_haplo	152	SNV	T	C	No	1		Heterozygous
NC_012920_rCRS H2a_haplo	152	SNV	T	T	Yes	1		Heterozygous
NC_012920_rCRS H2a_haplo	195	SNV	T	C	No	1		Homozygous
NC_012920_rCRS H2a_haplo	263	SNV	A	G	No	1		Homozygous
NC_012920_rCRS H2a_haplo	302^303	Insertion	-	C	No	1		Heterozygous
NC_012920_rCRS H2a_haplo	302^303	Insertion	-	CC	No	2		Heterozygous
NC_012920_rCRS H2a_haplo	302^303	Insertion	-	CCC	No	3		Heterozygous
NC_012920_rCRS H2a_haplo	302^303	Insertion	-	-	Yes	0		Heterozygous
NC_012920_rCRS H2a_haplo	310	SNV	T	C	No	1		Heterozygous
NC_012920_rCRS H2a_haplo	310	SNV	T	T	Yes	1		Heterozygous
NC_012920_rCRS H2a_haplo	310^311	Insertion	-	C	No	1		Heterozygous
NC_012920_rCRS H2a_haplo	310^311	Insertion	-	-	Yes	0		Heterozygous
NC_012920_rCRS H2a_haplo	351	SNV	A	G	No	1		Heterozygous
NC_012920_rCRS H2a_haplo	351	SNV	A	A	Yes	1		Heterozygous
NC_012920_rCRS H2a_haplo	16153	SNV	G	A	No	1		Homozygous
NC_012920_rCRS H2a_haplo	16172	SNV	T	C	No	1		Heterozygous
NC_012920_rCRS H2a_haplo	16172	SNV	T	T	Yes	1		Heterozygous
NC_012920_rCRS H2a_haplo	16263	SNV	T	A	No	1		Heterozygous
NC_012920_rCRS H2a_haplo	16263	SNV	T	T	Yes	1		Heterozygous
NC_012920_rCRS H2a_haplo	16298	SNV	T	C	No	1		Homozygous

Supplementary Table S2 B09_512v Variant Table

**Count**	**Coverage**	**Frequency**	Probability	Forward read count	Reverse read count	Forward read coverage	Reverse read coverage	Forward/reverse balance	Average quality
4226	4256	99.29511278	1	4226	0	4256	0	0	62.90416469
4221	4256	99.17763158	1	4221	0	4256	0	0	62.71120588
660	6011	10.97987024	1	660	0	6011	0	0	32.74393939
5332	6011	88.70404259	1	5332	0	6011	0	0	37.35408852
1104	6011	18.3663284	1	1104	0	6011	0	0	36.24003623
4905	6011	81.60039927	1	4905	0	6011	0	0	38.06381244
723	6011	12.02794876	1	723	0	6011	0	0	36.99170124
5285	6011	87.92214274	1	5285	0	6011	0	0	37.88552507
1755	1760	99.71590909	1	1755	0	1760	0	0	38.03988604
1758	1760	99.88636364	1	1758	0	1760	0	0	62.06598407
1431	1759	81.3530415	1	1431	0	1759	0	0	34.98742138
214	1759	12.16600341	1	214	0	1759	0	0	34.54205607
25	1759	1.421262081	1	25	0	1759	0	0	31.32
87	1759	4.945992041	1	87	0	1759	0	0	28.10344828
26	1758	1.478953356	1	26	0	1758	0	0	31.80696994
1731	1758	98.46416382	1	1731	0	1758	0	0	25.86020396
1722	1758	97.95221843	1	1722	0	1758	0	0	37.24332172
33	1758	1.877133106	1	33	0	1758	0	0	29.63636364
19	1775	1.070422535	1	19	0	1775	0	0	36.42105263
1754	1775	98.81690141	1	1754	0	1775	0	0	35.76054732
771	773	99.74126779	1	771	0	773	0	0	38.19455253
19	862	2.204176334	1	19	0	862	0	0	34.21052632
842	862	97.67981439	1	842	0	862	0	0	36.93586698
33	431	7.656612529	1	33	0	431	0	0	63.3030303
398	431	92.34338747	1	398	0	431	0	0	63.37437186
429	432	99.30555556	1	429	0	432	0	0	63.74592075

Read count	Read coverage	# unique start positions	# unique end positions	BaseQRankSum	Read position test probability	Read direction test probability	Homopolymer	Homopolymer length	QUAL
4226	4256	4	5		1	1	No	1	200
4221	4256	4	5		1	1	No	1	200
660	6011	3	5	-37.44	0	1	No	1	200
5332	6011	8	11		0	1	No	1	200
1104	6011	4	5	-39.78	0	1	No	1	200
4905	6011	8	10		0	1	No	1	200
723	6011	5	5	-24.6	0	1	No	1	200
5285	6011	7	10		0	1	No	1	200
1755	1760	4	6		1	1	No	1	200
1758	1760	4	6		1	1	No	1	200
1431	1759	4	4	14.79	1	1	Yes	7	200
214	1759	3	3	11.66	1	1	Yes	7	200
25	1759	1	2	1.34	1	1	Yes	7	200
87	1759	1	2		1	1	Yes	7	200
26	1758	1	1	4.18	1	1	No	1	200
1731	1758	4	4		1	1	Yes	7	200
1722	1758	4	4	10.29	1	1	Yes	5	200
33	1758	1	1		1	1	Yes	5	200
19	1775	2	2	1.74	0	1	No	1	200
1754	1775	4	4		3.58655E-05	1	No	1	200
771	773	3	4		1	1	No	1	200
19	862	3	2	-2.42	0	1	No	1	200
842	862	6	6		0.694890344	1	No	1	200
33	431	1	1	-0.54	0	1	No	1	200
398	431	14	3		9.58999E-06	1	No	1	200
429	432	15	3		1	1	No	1	200

Supplementary Table S2 B10_513v Variant Table

Reference mitogenome	Region	Type	Reference	Allele	Reference allele	Length	Linkage	Zygosity
NC_012920_rCRS H2a_haplo	73	SNV	A	G	No	1		Homozygous
NC_012920_rCRS H2a_haplo	146	SNV	T	C	No	1		Heterozygous
NC_012920_rCRS H2a_haplo	146	SNV	T	T	Yes	1		Heterozygous
NC_012920_rCRS H2a_haplo	150	SNV	C	T	No	1		Heterozygous
NC_012920_rCRS H2a_haplo	150	SNV	C	C	Yes	1		Heterozygous
NC_012920_rCRS H2a_haplo	152	SNV	T	C	No	1		Heterozygous
NC_012920_rCRS H2a_haplo	152	SNV	T	T	Yes	1		Heterozygous
NC_012920_rCRS H2a_haplo	189	SNV	A	G	No	1		Homozygous
NC_012920_rCRS H2a_haplo	200	SNV	A	G	No	1		Homozygous
NC_012920_rCRS H2a_haplo	204	SNV	T	C	No	1		Heterozygous
NC_012920_rCRS H2a_haplo	204	SNV	T	T	Yes	1		Heterozygous
NC_012920_rCRS H2a_haplo	214	SNV	A	G	No	1		Heterozygous
NC_012920_rCRS H2a_haplo	214	SNV	A	A	Yes	1		Heterozygous
NC_012920_rCRS H2a_haplo	215	SNV	A	G	No	1		Heterozygous
NC_012920_rCRS H2a_haplo	215	SNV	A	A	Yes	1		Heterozygous
NC_012920_rCRS H2a_haplo	263	SNV	A	G	No	1		Homozygous
NC_012920_rCRS H2a_haplo	302^303	Insertion	-	C	No	1		Heterozygous
NC_012920_rCRS H2a_haplo	302^303	Insertion	-	CC	No	2		Heterozygous
NC_012920_rCRS H2a_haplo	302^303	Insertion	-	-	Yes	0		Heterozygous
NC_012920_rCRS H2a_haplo	310	SNV	T	C	No	1		Heterozygous
NC_012920_rCRS H2a_haplo	310	SNV	T	T	Yes	1		Heterozygous
NC_012920_rCRS H2a_haplo	310^311	Insertion	-	C	No	1		Heterozygous
NC_012920_rCRS H2a_haplo	310^311	Insertion	-	-	Yes	0		Heterozygous
NC_012920_rCRS H2a_haplo	351	SNV	A	G	No	1		Heterozygous
NC_012920_rCRS H2a_haplo	351	SNV	A	A	Yes	1		Heterozygous
NC_012920_rCRS H2a_haplo	16129	SNV	G	A	No	1		Heterozygous
NC_012920_rCRS H2a_haplo	16129	SNV	G	G	Yes	1		Heterozygous
NC_012920_rCRS H2a_haplo	16152	SNV	T	C	No	1		Heterozygous
NC_012920_rCRS H2a_haplo	16152	SNV	T	T	Yes	1		Heterozygous
NC_012920_rCRS H2a_haplo	16176	SNV	C	T	No	1		Heterozygous
NC_012920_rCRS H2a_haplo	16176	SNV	C	C	Yes	1		Heterozygous
NC_012920_rCRS H2a_haplo	16189	SNV	T	C	No	1		Heterozygous
NC_012920_rCRS H2a_haplo	16189	SNV	T	T	Yes	1		Heterozygous
NC_012920_rCRS H2a_haplo	16223	SNV	C	T	No	1		Homozygous
NC_012920_rCRS H2a_haplo	16263	SNV	T	A	No	1		Heterozygous
NC_012920_rCRS H2a_haplo	16263	SNV	T	T	Yes	1		Heterozygous
NC_012920_rCRS H2a_haplo	16327	SNV	C	T	No	1		Homozygous

Supplementary Table S2 B10_513v Variant Table

**Count**	**Coverage**	**Frequency**	Probability	Forward read count	Reverse read count	Forward read coverage	Reverse read coverage	Forward/reverse balance	Average quality
5630	5666	99.36463113	1	5630	0	5666	0	0	63.20586146
919	8012	11.47029456	1	919	0	8012	0	0	32.67791077
7073	8012	88.28007988	1	7073	0	8012	0	0	36.93666054
7022	8013	87.63259703	1	7022	0	8013	0	0	36.96995158
983	8013	12.26756521	1	983	0	8013	0	0	35.43438454
6595	8014	82.2934864	1	6595	0	8014	0	0	37.69673995
1403	8014	17.50686299	1	1403	0	8014	0	0	36.94939416
2323	2357	98.55748833	1	2323	0	2357	0	0	34.44597503
2335	2357	99.0666101	1	2335	0	2357	0	0	37.89122056
44	2357	1.866779805	1	44	0	2357	0	0	35
2308	2357	97.92108613	1	2308	0	2357	0	0	37.60745234
181	2357	7.679253288	1	181	0	2357	0	0	37.8442556
2176	2357	92.32074671	1	2176	0	2357	0	0	38.45603273
31	2357	1.315231226	1	31	0	2357	0	0	37.77419355
2326	2357	98.68476877	1	2326	0	2357	0	0	38.13843508
2354	2357	99.87271956	1	2354	0	2357	0	0	62.33135089
2001	2355	84.96815287	1	2001	0	2355	0	0	34.77281091
161	2355	6.836518047	1	161	0	2355	0	0	33.22974357
181	2355	7.685774947	1	181	0	2355	0	0	28.78354749
32	2355	1.35881104	1	32	0	2355	0	0	29.65496882
2292	2355	97.32484076	1	2292	0	2355	0	0	25.1189045
2265	2355	96.17834395	1	2265	0	2355	0	0	37.12939212
86	2355	3.651804671	1	86	0	2355	0	0	28.19715254
38	2383	1.594628619	1	38	0	2383	0	0	30.5
2339	2383	98.15358791	1	2339	0	2383	0	0	35.46045319
11	955	1.151832461	0.999998937	11	0	955	0	0	58.63636364
943	955	98.7434555	1	943	0	955	0	0	63.06892895
10	955	1.047120419	0.99999997	10	0	955	0	0	34.9
945	955	98.95287958	1	945	0	955	0	0	37.63809524
974	991	98.28456105	1	974	0	991	0	0	36.61806982
17	991	1.715438951	1	17	0	991	0	0	35.76470588
13	991	1.311806256	0.999997658	13	0	991	0	0	29.15384615
970	991	97.88092836	1	970	0	991	0	0	34.51237113
54	54	100	1	54	0	54	0	0	38.38888889
20	75	26.66666667	1	20	0	75	0	0	62.2
55	75	73.33333333	1	55	0	75	0	0	62.78181818
75	75	100	1	75	0	75	0	0	42.82666667

Supplementary Table S2 B10_513v Variant Table

Read count	Read coverage	# unique start positions	# unique end positions	BaseQRankSum	Read position test probability	Read direction test probability	Homopolymer	Homopolymer length	QUAL
5630	5666	6	6		1	1	No	1	200
919	8012	4	6	-41.6	0	1	No	1	200
7073	8012	10	13		0	1	No	1	200
7022	8013	10	13	26.49	0	1	No	1	200
983	8013	5	6		0	1	No	1	200
6595	8014	10	10	32	0	1	No	1	200
1403	8014	5	9		0	1	No	1	200
2323	2357	7	9		1	1	No	1	200
2335	2357	7	9	0.25	1	1	No	1	200
44	2357	3	2	-0.59	0.894458331	1	No	1	200
2308	2357	6	9		1	1	No	1	200
181	2357	2	3	-1.89	0.788784688	1	No	1	200
2176	2357	7	9		0.971673942	1	No	1	200
31	2357	1	1	-0.91	0.910805863	1	No	1	200
2326	2357	7	9		1	1	No	1	200
2354	2357	7	9		1	1	No	1	200
2001	2355	7	7	20.8	0.918307358	1	Yes	7	200
161	2355	2	2	11.05	0.800469992	1	Yes	7	200
181	2355	2	2		0.788616937	1	Yes	7	200
32	2355	1	3	-7.6	1	1	No	1	200
2292	2355	7	7		1	1	Yes	7	200
2265	2355	7	7	14.84	1	1	Yes	5	200
86	2355	2	3		1	1	Yes	5	200
38	2383	3	2	-1.67	0	1	No	1	200
2339	2383	8	8		4.73862E-06	1	No	1	200
11	955	1	1	-0.15	0.470720402	1	No	1	59.73466735
943	955	4	7		1	1	No	1	200
10	955	1	1	-0.97	0.920533775	1	No	1	75.2724355
945	955	4	7		1	1	No	1	200
974	991	7	7	-0.21	0.832888043	1	No	1	200
17	991	3	2		1.97231E-12	1	No	1	200
13	991	2	3	0.22	0.80014296	1	No	1	56.30338941
970	991	9	5		1	1	No	1	200
54	54	6	2		1	1	No	1	200
20	75	1	2	0.24	1.38492E-07	1	No	1	200
55	75	7	2		0.000827649	1	No	1	200
75	75	8	3		1	1	No	1	200

Supplementary Table S2 B11_515v Variant Table

Reference mitogenome	Region	Type	Reference	Allele	Reference allele	Length	Linkage	Zygosity
NC_012920_rCRS H2a_haplo	72	SNV	T	C	No	1		Heterozygous
NC_012920_rCRS H2a_haplo	72	SNV	T	T	Yes	1		Heterozygous
NC_012920_rCRS H2a_haplo	146	SNV	T	C	No	1		Heterozygous
NC_012920_rCRS H2a_haplo	146	SNV	T	T	Yes	1		Heterozygous
NC_012920_rCRS H2a_haplo	150	SNV	C	T	No	1		Heterozygous
NC_012920_rCRS H2a_haplo	150	SNV	C	C	Yes	1		Heterozygous
NC_012920_rCRS H2a_haplo	152	SNV	T	C	No	1		Heterozygous
NC_012920_rCRS H2a_haplo	152	SNV	T	T	Yes	1		Heterozygous
NC_012920_rCRS H2a_haplo	189	SNV	A	G	No	1		Heterozygous
NC_012920_rCRS H2a_haplo	189	SNV	A	A	Yes	1		Heterozygous
NC_012920_rCRS H2a_haplo	195	SNV	T	C	No	1		Homozygous
NC_012920_rCRS H2a_haplo	240	SNV	A	G	No	1		Homozygous
NC_012920_rCRS H2a_haplo	263	SNV	A	G	No	1		Homozygous
NC_012920_rCRS H2a_haplo	302^303	Insertion	-	C	No	1		Heterozygous
NC_012920_rCRS H2a_haplo	302^303	Insertion	-	-	Yes	0		Heterozygous
NC_012920_rCRS H2a_haplo	310	SNV	T	C	No	1		Heterozygous
NC_012920_rCRS H2a_haplo	310	SNV	T	T	Yes	1		Heterozygous
NC_012920_rCRS H2a_haplo	310^311	Insertion	-	C	No	1		Heterozygous
NC_012920_rCRS H2a_haplo	310^311	Insertion	-	-	Yes	0		Heterozygous
NC_012920_rCRS H2a_haplo	456	SNV	C	T	No	1		Homozygous
NC_012920_rCRS H2a_haplo	514..515	Deletion	CA	-	No	2		Homozygous
NC_012920_rCRS H2a_haplo	16172	SNV	T	C	No	1		Heterozygous
NC_012920_rCRS H2a_haplo	16172	SNV	T	T	Yes	1		Heterozygous
NC_012920_rCRS H2a_haplo	16192	SNV	C	T	No	1		Homozygous
NC_012920_rCRS H2a_haplo	16249	SNV	T	C	No	1		Heterozygous
NC_012920_rCRS H2a_haplo	16249	SNV	T	T	Yes	1		Heterozygous
NC_012920_rCRS H2a_haplo	16256	SNV	C	A	No	1		Heterozygous
NC_012920_rCRS H2a_haplo	16256	SNV	C	C	Yes	1		Heterozygous
NC_012920_rCRS H2a_haplo	16263	SNV	T	A	No	1		Heterozygous
NC_012920_rCRS H2a_haplo	16263	SNV	T	T	Yes	1		Heterozygous
NC_012920_rCRS H2a_haplo	16304	SNV	T	C	No	1		Homozygous

Supplementary Table S2 B11_515v Variant Table

**Count**	**Coverage**	**Frequency**	Probability	Forward read count	Reverse read count	Forward read coverage	Reverse read coverage	Forward/reverse balance	Average quality
403	3730	10.80428954	1	403	0	3730	0	0	62.5707196
3326	3730	89.1689008	1	3326	0	3730	0	0	63.43956705
653	5415	12.05909511	1	653	0	5415	0	0	32.8085758
4749	5415	87.70083102	1	4749	0	5415	0	0	37.49947357
1031	5416	19.03618907	1	1031	0	5416	0	0	36.27546072
4382	5416	80.9084195	1	4382	0	5416	0	0	37.98585121
737	5417	13.6053166	1	737	0	5417	0	0	36.95658073
4676	5417	86.32084179	1	4676	0	5417	0	0	37.88451668
50	1689	2.960331557	1	50	0	1689	0	0	33.52
1637	1689	96.92125518	1	1637	0	1689	0	0	36.62858888
1678	1688	99.40758294	1	1678	0	1688	0	0	37.77056019
1673	1688	99.11137441	1	1673	0	1688	0	0	62.02630006
1688	1688	100	1	1688	0	1688	0	0	62.82049763
72	1688	4.265402844	1	72	0	1688	0	0	34.16666667
1615	1688	95.67535545	1	1615	0	1688	0	0	30.61052632
37	1688	2.191943128	1	37	0	1688	0	0	34.97933438
1640	1688	97.1563981	1	1640	0	1688	0	0	25.71096332
1630	1688	96.56398104	1	1630	0	1688	0	0	37.18711656
58	1688	3.436018957	1	58	0	1688	0	0	32.36206897
13	13	100	1	13	0	13	0	0	64
13	13	100	1	13	0	13	0	0	37.46153846
10	684	1.461988304	0.999997326	10	0	684	0	0	34.7
672	684	98.24561404	1	672	0	684	0	0	37.09821429
877	891	98.42873176	1	877	0	891	0	0	36.49714937
3	297	1.01010101	0.745790791	3	0	297	0	0	36
294	297	98.98989899	1	294	0	297	0	0	38.87414966
4	298	1.342281879	0.966120782	4	0	298	0	0	63
294	298	98.65771812	1	294	0	298	0	0	62.77891156
35	333	10.51051051	1	35	0	333	0	0	63.37142857
298	333	89.48948949	1	298	0	333	0	0	62.95302013
331	333	99.3993994	1	331	0	333	0	0	63.58912387

Supplementary Table S2 B11_515v Variant Table

Read count	Read coverage	# unique start positions	# unique end positions	BaseQRankSum	Read position test probability	Read direction test probability	Homopolymer	Homopolymer length	QUAL
403	3730	3	2	-1.05	1	1	No	1	200
3326	3730	4	5		1	1	No	1	200
653	5415	3	4	-37.58	0	1	No	1	200
4749	5415	9	9		0	1	No	1	200
1031	5416	4	5	-36.99	0	1	No	1	200
4382	5416	8	8		0	1	No	1	200
737	5417	5	4	-23.94	0	1	No	1	200
4676	5417	7	9		0	1	No	1	200
50	1689	2	1	-2.92	0.520744722	1	No	1	200
1637	1689	5	5		1	1	No	1	200
1678	1688	4	4		1	1	No	1	200
1673	1688	4	4		1	1	No	1	200
1688	1688	4	4		1	1	No	1	200
72	1688	1	1	6.01	0.729747171	1	Yes	7	200
1615	1688	4	4		0.99578401	1	Yes	7	200
37	1688	1	1	3.16	1	1	No	1	200
1640	1688	5	5		1	1	Yes	7	200
1630	1688	5	5	5.8	1	1	Yes	5	200
58	1688	1	1		1	1	Yes	5	200
13	13	1	1		1	1	No	1	200
13	13	1	1		1	1	No	1	200
10	684	3	2	-2.6	1.81688E-12	1	No	1	55.72854839
672	684	5	5		0.670333357	1	No	1	200
877	891	8	5	3.81	0.388677993	1	No	1	200
3	297	2	1	-2.51	0	1	No	1	5.948087211
294	297	5	2		0.09128838	1	No	1	200
4	298	3	1	-0.55	0	1	No	1	14.70066617
294	298	6	2		0.439745244	1	No	1	200
35	333	2	1	-0.26	0	1	No	1	200
298	333	8	2		5.36254E-06	1	No	1	200
331	333	10	2		1	1	No	1	200

Supplementary Table S2 B12_516v Variant Table

Reference mitogenome	Region	Type	Reference	Allele	Reference allele	Length	Linkage	Zygosity
NC_012920_rCRS H2a_haplo	72	SNV	T	C	No	1		Homozygous
NC_012920_rCRS H2a_haplo	146	SNV	T	C	No	1		Heterozygous
NC_012920_rCRS H2a_haplo	146	SNV	T	T	Yes	1		Heterozygous
NC_012920_rCRS H2a_haplo	150	SNV	C	T	No	1		Heterozygous
NC_012920_rCRS H2a_haplo	150	SNV	C	C	Yes	1		Heterozygous
NC_012920_rCRS H2a_haplo	152	SNV	T	C	No	1		Heterozygous
NC_012920_rCRS H2a_haplo	152	SNV	T	T	Yes	1		Heterozygous
NC_012920_rCRS H2a_haplo	263	SNV	A	G	No	1		Homozygous
NC_012920_rCRS H2a_haplo	302	Replacement	A	CCC	No	3		Heterozygous
NC_012920_rCRS H2a_haplo	302	SNV	A	A	Yes	1		Heterozygous
NC_012920_rCRS H2a_haplo	302^303	Insertion	-	C	No	1		Heterozygous
NC_012920_rCRS H2a_haplo	302^303	Insertion	-	CC	No	2		Heterozygous
NC_012920_rCRS H2a_haplo	302^303	Insertion	-	CCC	No	3		Heterozygous
NC_012920_rCRS H2a_haplo	302^303	Insertion	-	-	Yes	0		Heterozygous
NC_012920_rCRS H2a_haplo	310	SNV	T	C	No	1		Heterozygous
NC_012920_rCRS H2a_haplo	310	SNV	T	T	Yes	1		Heterozygous
NC_012920_rCRS H2a_haplo	310^311	Insertion	-	C	No	1		Heterozygous
NC_012920_rCRS H2a_haplo	310^311	Insertion	-	-	Yes	0		Heterozygous
NC_012920_rCRS H2a_haplo	351	SNV	A	G	No	1		Heterozygous
NC_012920_rCRS H2a_haplo	351	SNV	A	A	Yes	1		Heterozygous
NC_012920_rCRS H2a_haplo	16093	SNV	T	C	No	1		Heterozygous
NC_012920_rCRS H2a_haplo	16093	SNV	T	T	Yes	1		Heterozygous
NC_012920_rCRS H2a_haplo	16129	SNV	G	A	No	1		Homozygous
NC_012920_rCRS H2a_haplo	16148	SNV	C	T	No	1		Heterozygous
NC_012920_rCRS H2a_haplo	16148	SNV	C	C	Yes	1		Heterozygous
NC_012920_rCRS H2a_haplo	16172	SNV	T	C	No	1		Heterozygous
NC_012920_rCRS H2a_haplo	16172	SNV	T	T	Yes	1		Heterozygous
NC_012920_rCRS H2a_haplo	16263	SNV	T	A	No	1		Heterozygous
NC_012920_rCRS H2a_haplo	16263	SNV	T	T	Yes	1		Heterozygous
NC_012920_rCRS H2a_haplo	16298	SNV	T	C	No	1		Homozygous

Supplementary Table S2 B12_516v Variant Table

**Count**	**Coverage**	**Frequency**	Probability	Forward read count	Reverse read count	Forward read coverage	Reverse read coverage	Forward/reverse balance	Average quality
4367	4385	99.58950969	1	4367	0	4385	0	0	62.91893749
496	5672	8.74471086	1	496	0	5672	0	0	32.80645161
5166	5672	91.07898449	1	5166	0	5672	0	0	37.57781649
759	5671	13.38388291	1	759	0	5671	0	0	36.48748353
4909	5671	86.56321636	1	4909	0	5671	0	0	38.10531677
572	5671	10.08640451	1	572	0	5671	0	0	37.01223776
5094	5671	89.82542761	1	5094	0	5671	0	0	38.04711425
1291	1291	100	1	1291	0	1291	0	0	60.20836561
27	1290	2.093023256	1	27	0	1290	0	0	29.95012987
1247	1290	96.66666667	1	1247	0	1290	0	0	27.82912812
188	1290	14.57364341	1	188	0	1290	0	0	35.77659368
908	1290	70.3875969	1	908	0	1290	0	0	34.88931778
120	1290	9.302325581	1	120	0	1290	0	0	32.77222077
71	1290	5.503875969	1	71	0	1290	0	0	28.07042253
18	1290	1.395348837	1	18	0	1290	0	0	32.94444444
1255	1290	97.28682171	1	1255	0	1290	0	0	25.22262948
1247	1290	96.66666667	1	1247	0	1290	0	0	37.04009623
39	1290	3.023255814	1	39	0	1290	0	0	29.94871795
23	1310	1.755725191	1	23	0	1310	0	0	32.7826087
1285	1310	98.09160305	1	1285	0	1310	0	0	35.73385214
844	898	93.98663697	1	844	0	898	0	0	63.21682464
53	898	5.902004454	1	53	0	898	0	0	62.13207547
895	898	99.66592428	1	895	0	898	0	0	63.46145251
11	898	1.224944321	1	11	0	898	0	0	36
887	898	98.77505568	1	887	0	898	0	0	38.36640361
15	975	1.538461538	0.999999996	15	0	975	0	0	31.66666667
957	975	98.15384615	1	957	0	975	0	0	37.22361546
23	275	8.363636364	1	23	0	275	0	0	62.82608696
252	275	91.63636364	1	252	0	275	0	0	63.1031746
275	276	99.63768116	1	275	0	276	0	0	63.57818182

Read count	Read coverage	# unique start positions	# unique end positions	BaseQRankSum	Read position test probability	Read direction test probability	Homopolymer	Homopolymer length	QUAL
4367	4385	5	5		1	1	No	1	200
496	5672	4	3	-33.26	0	1	No	1	200
5166	5672	8	9		4.44089E-15	1	No	1	200
759	5671	4	4	-34.78	0	1	No	1	200
4909	5671	8	8		0	1	No	1	200
572	5671	4	3	-24.58	0	1	No	1	200
5094	5671	8	9		0	1	No	1	200
1291	1291	3	4		1	1	No	1	200
27	1290	1	1	4.28	1	1	No	1	200
1247	1290	3	3		1	1	Yes	7	200
188	1290	3	2	19.12	1	1	Yes	7	200
908	1290	3	2	19.26	1	1	Yes	7	200
120	1290	2	3	10.43	1	1	Yes	7	200
71	1290	2	1		1	1	Yes	7	200
18	1290	1	1	-3.99	1	1	No	1	200
1255	1290	3	3		1	1	Yes	7	200
1247	1290	3	3	10.95	1	1	Yes	5	200
39	1290	1	1		1	1	Yes	5	200
23	1310	2	4	0.14	0	1	No	1	200
1285	1310	3	3		1.426E-05	1	No	1	200
844	898	2	4	0.14	0.982382882	1	No	1	200
53	898	1	1		0.740231635	1	No	1	200
895	898	2	4		1	1	No	1	200
11	898	1	1	-1.91	0.878824259	1	No	1	200
887	898	2	4		1	1	No	1	200
15	975	2	2	-3.65	0	1	No	1	84.43697503
957	975	4	5		0.569126471	1	No	1	200
23	275	1	1	-0.19	0	1	No	1	200
252	275	8	3		3.55474E-05	1	No	1	200
275	276	10	3		1	1	No	1	200

Supplementary Table S2 C01_519v Variant Table

Reference mitogenome	Region	Type	Reference	Allele	Reference allele	Length	Linkage	Zygosity
NC_012920_rCRS H2a_haplo	73	SNV	A	G	No	1		Homozygous
NC_012920_rCRS H2a_haplo	146	SNV	T	C	No	1		Heterozygous
NC_012920_rCRS H2a_haplo	146	SNV	T	T	Yes	1		Heterozygous
NC_012920_rCRS H2a_haplo	150	SNV	C	T	No	1		Heterozygous
NC_012920_rCRS H2a_haplo	150	SNV	C	C	Yes	1		Heterozygous
NC_012920_rCRS H2a_haplo	152	SNV	T	C	No	1		Heterozygous
NC_012920_rCRS H2a_haplo	152	SNV	T	T	Yes	1		Heterozygous
NC_012920_rCRS H2a_haplo	204	SNV	T	C	No	1		Heterozygous
NC_012920_rCRS H2a_haplo	204	SNV	T	T	Yes	1		Heterozygous
NC_012920_rCRS H2a_haplo	248	Deletion	A	-	No	1		Homozygous
NC_012920_rCRS H2a_haplo	263	SNV	A	G	No	1		Homozygous
NC_012920_rCRS H2a_haplo	302^303	Insertion	-	C	No	1		Heterozygous
NC_012920_rCRS H2a_haplo	302^303	Insertion	-	CC	No	2		Heterozygous
NC_012920_rCRS H2a_haplo	302^303	Insertion	-	-	Yes	0		Heterozygous
NC_012920_rCRS H2a_haplo	310	SNV	T	C	No	1		Heterozygous
NC_012920_rCRS H2a_haplo	310	SNV	T	T	Yes	1		Heterozygous
NC_012920_rCRS H2a_haplo	310^311	Insertion	-	C	No	1		Heterozygous
NC_012920_rCRS H2a_haplo	310^311	Insertion	-	-	Yes	0		Heterozygous
NC_012920_rCRS H2a_haplo	351	SNV	A	G	No	1		Heterozygous
NC_012920_rCRS H2a_haplo	351	SNV	A	A	Yes	1		Heterozygous
NC_012920_rCRS H2a_haplo	16129	SNV	G	A	No	1		Homozygous
NC_012920_rCRS H2a_haplo	16172	SNV	T	C	No	1		Heterozygous
NC_012920_rCRS H2a_haplo	16172	SNV	T	T	Yes	1		Heterozygous
NC_012920_rCRS H2a_haplo	16263	SNV	T	A	No	1		Heterozygous
NC_012920_rCRS H2a_haplo	16263	SNV	T	T	Yes	1		Heterozygous
NC_012920_rCRS H2a_haplo	16304	SNV	T	C	No	1		Homozygous
NC_012920_rCRS H2a_haplo	16311	SNV	T	C	No	1		Homozygous
NC_012920_rCRS H2a_haplo	16361	SNV	G	A	No	1		Heterozygous
NC_012920_rCRS H2a_haplo	16361	SNV	G	G	Yes	1		Heterozygous
NC_012920_rCRS H2a_haplo	16519	SNV	T	C	No	1		Homozygous

Supplementary Table S2 C01_519v Variant Table

**Count**	**Coverage**	**Frequency**	Probability	Forward read count	Reverse read count	Forward read coverage	Reverse read coverage	Forward/reverse balance	Average quality
1038	1038	100	1	1038	0	1038	0	0	63.54142582
132	1407	9.381663113	1	132	0	1407	0	0	32.97727273
1272	1407	90.40511727	1	1272	0	1407	0	0	37.38443396
221	1407	15.70717839	1	221	0	1407	0	0	36.23076923
1186	1407	84.29282161	1	1186	0	1407	0	0	38.20994941
171	1407	12.15351812	1	171	0	1407	0	0	36.85380117
1235	1407	87.77540867	1	1235	0	1407	0	0	38.07773279
159	370	42.97297297	1	159	0	370	0	0	37.71698113
211	370	57.02702703	1	211	0	370	0	0	36.78672986
367	370	99.18918919	1	367	0	370	0	0	62.46049046
369	370	99.72972973	1	369	0	370	0	0	61.94850949
281	370	75.94594595	1	281	0	370	0	0	35.07117438
30	370	8.108108108	1	30	0	370	0	0	35.15
59	370	15.94594595	1	59	0	370	0	0	31.83050847
4	370	1.081081081	0.985042357	4	0	370	0	0	34.25
362	370	97.83783784	1	362	0	370	0	0	27.29742173
363	370	98.10810811	1	363	0	370	0	0	37.24242424
7	370	1.891891892	1	7	0	370	0	0	28.85714286
5	374	1.336898396	0.986960466	5	0	374	0	0	33.2
368	374	98.39572193	1	368	0	374	0	0	36.2173913
137	138	99.27536232	1	137	0	138	0	0	63.87591241
139	154	90.25974026	1	139	0	154	0	0	37.51079137
15	154	9.74025974	1	15	0	154	0	0	38.06666667
15	160	9.375	1	15	0	160	0	0	63.86666667
145	160	90.625	1	145	0	160	0	0	63.0137931
159	160	99.375	1	159	0	160	0	0	63.6918239
160	160	100	1	160	0	160	0	0	42.2875
7	436	1.605504587	1	7	0	436	0	0	37
428	436	98.16513761	1	428	0	436	0	0	38.28971963
280	280	100	1	280	0	280	0	0	38.1

Supplementary Table S2 C01_519v Variant Table

Read count	Read coverage	# unique start positions	# unique end positions	BaseQRankSum	Read position test probability	Read direction test probability	Homopolymer	Homopolymer length	QUAL
1038	1038	5	4		1	1	No	1	200
132	1407	2	2	-16.72	0	1	No	1	200
1272	1407	9	7		0.000715539	1	No	1	200
221	1407	3	4	-18.49	0	1	No	1	200
1186	1407	9	6		3.86358E-14	1	No	1	200
171	1407	4	3	-13.35	0	1	No	1	200
1235	1407	8	6		2.91024E-07	1	No	1	200
159	370	2	3	2.55	1	1	No	1	200
211	370	3	2		1	1	No	1	200
367	370	4	3		1	1	No	1	200
369	370	4	3		1	1	No	1	200
281	370	4	2	8.24	1	1	Yes	7	200
30	370	1	2	4.7	1	1	Yes	7	200
59	370	1	1		1	1	Yes	7	200
4	370	1	1	-2.08	1	1	No	1	18.25136828
362	370	5	4		1	1	Yes	7	200
363	370	5	4	2.84	1	1	Yes	5	200
7	370	1	1		1	1	Yes	5	200
5	374	2	2	-0.17	0	1	No	1	18.84737912
368	374	4	3		0.051908272	1	No	1	200
137	138	2	2		1	1	No	1	200
139	154	4	3	-1.02	0.002391069	1	No	1	200
15	154	3	3		2.44249E-15	1	No	1	200
15	160	1	1	0.17	1.11022E-16	1	No	1	200
145	160	8	3		0.003156196	1	No	1	200
159	160	9	3		1	1	No	1	200
160	160	9	3		1	1	No	1	200
7	436	1	1	-3.12	0.152732093	1	No	1	200
428	436	12	3		1	1	No	1	200
280	280	5	1		1	1	No	1	200

Supplementary Table S2 C02_520v Variant Table

Reference mitogenome	Region	Type	Reference	Allele	Reference allele	Length	Linkage	Zygosity
NC_012920_rCRS H2a_haplo	73	SNV	A	G	No	1		Homozygous
NC_012920_rCRS H2a_haplo	146	SNV	T	C	No	1		Heterozygous
NC_012920_rCRS H2a_haplo	146	SNV	T	T	Yes	1		Heterozygous
NC_012920_rCRS H2a_haplo	150	SNV	C	T	No	1		Heterozygous
NC_012920_rCRS H2a_haplo	150	SNV	C	C	Yes	1		Heterozygous
NC_012920_rCRS H2a_haplo	152	SNV	T	C	No	1		Heterozygous
NC_012920_rCRS H2a_haplo	152	SNV	T	T	Yes	1		Heterozygous
NC_012920_rCRS H2a_haplo	195	SNV	T	C	No	1		Homozygous
NC_012920_rCRS H2a_haplo	263	SNV	A	G	No	1		Homozygous
NC_012920_rCRS H2a_haplo	302^303	Insertion	-	C	No	1		Heterozygous
NC_012920_rCRS H2a_haplo	302^303	Insertion	-	-	Yes	0		Heterozygous
NC_012920_rCRS H2a_haplo	309..310	MNV	CT	TC	No	2		Heterozygous
NC_012920_rCRS H2a_haplo	309..310	MNV	CT	CT	Yes	2		Heterozygous
NC_012920_rCRS H2a_haplo	310^311	Insertion	-	C	No	1		Heterozygous
NC_012920_rCRS H2a_haplo	310^311	Insertion	-	-	Yes	0		Heterozygous
NC_012920_rCRS H2a_haplo	351	SNV	A	G	No	1		Heterozygous
NC_012920_rCRS H2a_haplo	351	SNV	A	A	Yes	1		Heterozygous
NC_012920_rCRS H2a_haplo	16140	SNV	T	A	No	1		Heterozygous
NC_012920_rCRS H2a_haplo	16140	SNV	T	T	Yes	1		Heterozygous
NC_012920_rCRS H2a_haplo	16143	SNV	T	C	No	1		Heterozygous
NC_012920_rCRS H2a_haplo	16143	SNV	T	T	Yes	1		Heterozygous
NC_012920_rCRS H2a_haplo	16146	SNV	A	G	No	1		Heterozygous
NC_012920_rCRS H2a_haplo	16146	SNV	A	A	Yes	1		Heterozygous
NC_012920_rCRS H2a_haplo	16149	SNV	A	C	No	1		Heterozygous
NC_012920_rCRS H2a_haplo	16149	SNV	A	A	Yes	1		Heterozygous
NC_012920_rCRS H2a_haplo	16161	SNV	T	A	No	1		Heterozygous
NC_012920_rCRS H2a_haplo	16161	SNV	T	T	Yes	1		Heterozygous
NC_012920_rCRS H2a_haplo	16172	SNV	T	C	No	1		Heterozygous
NC_012920_rCRS H2a_haplo	16172	SNV	T	T	Yes	1		Heterozygous
NC_012920_rCRS H2a_haplo	16175	SNV	A	C	No	1		Heterozygous
NC_012920_rCRS H2a_haplo	16175	SNV	A	A	Yes	1		Heterozygous
NC_012920_rCRS H2a_haplo	16178	SNV	T	A	No	1		Heterozygous
NC_012920_rCRS H2a_haplo	16178	Deletion	T	-	No	1		Heterozygous
NC_012920_rCRS H2a_haplo	16178	SNV	T	T	Yes	1		Heterozygous
NC_012920_rCRS H2a_haplo	16180	Deletion	A	-	No	1		Heterozygous
NC_012920_rCRS H2a_haplo	16180	SNV	A	A	Yes	1		Heterozygous
NC_012920_rCRS H2a_haplo	5182..16183	MNV	AA	CC	No	2		Heterozygous
NC_012920_rCRS H2a_haplo	5182..16183	MNV	AA	AA	Yes	2		Heterozygous
NC_012920_rCRS H2a_haplo	16183	SNV	A	C	No	1		Heterozygous
NC_012920_rCRS H2a_haplo	16183	Replacement	A	CC	No	2		Heterozygous
NC_012920_rCRS H2a_haplo	16183	Replacement	A	CCC	No	3		Heterozygous
NC_012920_rCRS H2a_haplo	5183^16184	Insertion	-	C	No	1		Heterozygous
NC_012920_rCRS H2a_haplo	5183^16184	Insertion	-	-	Yes	0		Heterozygous
NC_012920_rCRS H2a_haplo	16189	SNV	T	C	No	1		Heterozygous
NC_012920_rCRS H2a_haplo	16189	Deletion	T	-	No	1		Heterozygous
NC_012920_rCRS H2a_haplo	16189	SNV	T	T	Yes	1		Heterozygous
NC_012920_rCRS H2a_haplo	16194	SNV	A	C	No	1		Heterozygous
NC_012920_rCRS H2a_haplo	16194	SNV	A	A	Yes	1		Heterozygous
NC_012920_rCRS H2a_haplo	16223	SNV	C	T	No	1		Homozygous
NC_012920_rCRS H2a_haplo	16233	SNV	A	C	No	1		Heterozygous
NC_012920_rCRS H2a_haplo	16233	SNV	A	A	Yes	1		Heterozygous
NC_012920_rCRS H2a_haplo	16234	SNV	C	A	No	1		Heterozygous

NC_012920_rCRS	H2a_haplo	16234	SNV	C	C	Yes	1		Heterozygous
NC_012920_rCRS	H2a_haplo	16238	SNV	T	A	No	1		Heterozygous
NC_012920_rCRS	H2a_haplo	16238	SNV	T	T	Yes	1		Heterozygous
NC_012920_rCRS	H2a_haplo	16244	SNV	G	C	No	1		Heterozygous
NC_012920_rCRS	H2a_haplo	16244	SNV	G	T	No	1		Heterozygous
NC_012920_rCRS	H2a_haplo	16244	SNV	G	G	Yes	1		Heterozygous
NC_012920_rCRS	H2a_haplo	16263	SNV	T	A	No	1		Heterozygous
NC_012920_rCRS	H2a_haplo	16263	SNV	T	T	Yes	1		Heterozygous
NC_012920_rCRS	H2a_haplo	16320	SNV	C	T	No	1		Homozygous
NC_012920_rCRS	H2a_haplo	16375	Deletion	C	-	No	1		Heterozygous
NC_012920_rCRS	H2a_haplo	16375	SNV	C	C	Yes	1		Heterozygous
NC_012920_rCRS	H2a_haplo	16519	SNV	T	C	No	1		Homozygous

Supplementary Table S2 C02_520v Variant Table

**Count**	**Coverage**	**Frequency**	Probability	Forward read count	Reverse read count	Forward read coverage	Reverse read coverage	Forward/reverse balance	Average quality
1128	1132	99.64664311	1	1128	0	1132	0	0	63.3785461
243	1775	13.69014085	1	243	0	1775	0	0	33.02057613
1530	1775	86.1971831	1	1530	0	1775	0	0	37.37712418
1509	1775	85.01408451	1	1509	0	1775	0	0	37.33001988
265	1775	14.92957746	1	265	0	1775	0	0	36.37358491
1385	1777	77.9403489	1	1385	0	1777	0	0	37.89025271
387	1777	21.778278	1	387	0	1777	0	0	37.39018088
642	646	99.38080495	1	642	0	646	0	0	38.13862928
646	646	100	1	646	0	646	0	0	62.90557276
54	646	8.359133127	1	54	0	646	0	0	34.24074074
592	646	91.64086687	1	592	0	646	0	0	30.6722973
10	645	1.550387597	1	10	0	645	0	0	27.68
619	645	95.96899225	1	619	0	645	0	0	26.62746365
617	645	95.65891473	1	617	0	645	0	0	37.31442464
28	645	4.341085271	1	28	0	645	0	0	34.39285715
7	652	1.073619632	0.99998199	7	0	652	0	0	34.57142857
645	652	98.92638037	1	645	0	652	0	0	36.26666667
11	157	7.006369427	0.999797179	11	0	157	0	0	14.63636364
144	157	91.71974522	1	144	0	157	0	0	22.79166667
14	157	8.917197452	0.999787391	14	0	157	0	0	14.57142857
141	157	89.8089172	1	141	0	157	0	0	21.77304965
9	157	5.732484076	0.882298484	9	0	157	0	0	12.11111111
145	157	92.3566879	1	145	0	157	0	0	26.64137931
41	157	26.11464968	0.999999848	41	0	157	0	0	13.24390244
114	157	72.61146497	1	114	0	157	0	0	22.1754386
34	162	20.98765432	1	34	0	162	0	0	13
127	162	78.39506173	1	127	0	162	0	0	22.26771654
151	162	93.20987654	1	151	0	162	0	0	27.68874172
10	162	6.172839506	1	10	0	162	0	0	32.7
34	188	18.08510638	1	34	0	188	0	0	15.29411765
152	188	80.85106383	1	152	0	188	0	0	27.86184211
25	188	13.29787234	0.999999903	25	0	188	0	0	14.04
4	188	2.127659574	0.999988232	4	0	188	0	0	13.5
158	188	84.04255319	1	158	0	188	0	0	24.02531646
42	188	22.34042553	1	42	0	188	0	0	30.76190476
143	188	76.06382979	1	143	0	188	0	0	30.87412587
4	188	2.127659574	0.999995354	4	0	188	0	0	18.54914778
52	188	27.65957447	1	52	0	188	0	0	31.11858965
68	188	36.17021277	1	68	0	188	0	0	29.04749111
49	188	26.06382979	1	49	0	188	0	0	29.44897281
13	188	6.914893617	1	13	0	188	0	0	32.25000002
2	188	1.063829787	0.99989602	2	0	188	0	0	32
185	188	98.40425532	1	185	0	188	0	0	30.24324324
179	187	95.72192513	1	179	0	187	0	0	35.66480447
4	187	2.139037433	0.999999958	4	0	187	0	0	37
4	187	2.139037433	0.999999996	4	0	187	0	0	37.25
14	193	7.25388601	0.99969646	14	0	193	0	0	17.71428571
179	193	92.74611399	1	179	0	193	0	0	35.89385475
33	37	89.18918919	1	33	0	37	0	0	31.36363636
3	37	8.108108108	0.999994858	3	0	37	0	0	23
34	37	91.89189189	1	34	0	37	0	0	24.47058824
5	37	13.51351351	0.999496731	5	0	37	0	0	22.4

32	37	86.48648649	1	32	0	37	0	0	27.71875
4	37	10.81081081	0.82019225	4	0	37	0	0	14
30	37	81.08108108	1	30	0	37	0	0	23.9
10	37	27.02702703	0.999999999	10	0	37	0	0	21.8
3	37	8.108108108	0.88313231	3	0	37	0	0	17.33333333
24	37	64.86486486	1	24	0	37	0	0	27.66666667
5	42	11.9047619	1	5	0	42	0	0	64
35	42	83.33333333	1	35	0	42	0	0	37.48571429
41	42	97.61904762	1	41	0	42	0	0	40.82926829
7	390	1.794871795	1	7	0	390	0	0	38.71428571
382	390	97.94871795	1	382	0	390	0	0	38.57329843
351	352	99.71590909	1	351	0	352	0	0	38.03703704

Supplementary Table S2 C02_520v Variant Table

Read count	Read coverage	# unique start positions	# unique end positions	BaseQRankSum	Read position test probability	Read direction test probability	Homopolymer	Homopolymer length	QUAL
1128	1132	3	4		1	1	No	1	200
243	1775	3	4	-22.57	0	1	No	1	200
1530	1775	6	7		1.94463E-06	1	No	1	200
1509	1775	5	7	13.76	3.53197E-07	1	No	1	200
265	1775	4	3		0	1	No	1	200
1385	1777	5	6	17.3	0	1	No	1	200
387	1777	4	5		0	1	No	1	200
642	646	4	3		1	1	No	1	200
646	646	4	3		1	1	No	1	200
54	646	1	1	4.65	1	1	Yes	7	200
592	646	4	3		1	1	Yes	7	200
10	645	1	1	0.79	1	1	No	1	200
619	645	5	4		1	1	Yes	7	200
617	645	5	4	3.97	1	1	Yes	5	200
28	645	1	1		1	1	Yes	5	200
7	652	2	2	0.56	0	1	No	1	47.44486287
645	652	5	4		0.04041148	1	No	1	200
11	157	1	3	-2.78	1	1	No	1	36.92887723
144	157	2	2		1	1	No	1	200
14	157	1	2	-4	1	1	No	1	36.72419172
141	157	2	3		1	1	No	1	200
9	157	1	2	-4.24	1	1	No	1	9.292179423
145	157	2	3		1	1	No	1	200
41	157	2	2	-8.57	1	1	No	1	68.19300799
114	157	2	3		1	1	No	1	200
34	162	1	3	-6.88	0.321395396	1	No	1	200
127	162	3	3		0.746600286	1	No	1	200
151	162	2	3	-3.13	0.034504632	1	No	1	200
10	162	3	3		1.05687E-09	1	No	1	200
34	188	2	2	-6.79	0.025891219	1	No	1	200
152	188	4	5		0.738544997	1	No	1	200
25	188	2	3	-6.61	0.105826862	1	No	1	70.15022874
4	188	1	1	-2.13	0.687867625	1	No	1	49.29286269
158	188	4	5		0.865573648	1	No	1	200
42	188	2	5	0.23	0.53185653	1	Yes	4	200
143	188	4	5		0.915249914	1	Yes	4	200
4	188	1	2	-5.8	0.393068869	1	No	1	53.329021
52	188	3	5		0.71212778	1	Yes	5	200
68	188	4	2	-3.29	0.810386604	1	Yes	5	200
49	188	4	3	-1.18	0.580703423	1	Yes	5	200
13	188	2	3	0.64	0.435054106	1	Yes	5	200
2	188	1	1	-0.42	0.54466509	1	Yes	5	39.83050605
185	188	4	6		1	1	Yes	5	200
179	187	4	6	-0.04	1	1	No	1	200
4	187	2	2	0.29	0.679907255	1	Yes	5	73.79863945
4	187	3	2		0.679907255	1	No	1	83.87216143
14	193	3	4	-5.5	1.04974E-09	1	No	1	35.17783927
179	193	8	4		0.214250787	1	No	1	200
33	37	6	3	3.11	1	1	No	1	200
3	37	1	1	-0.61	1	1	No	1	52.88893267
34	37	6	3		1	1	No	1	200
5	37	1	2	-1.02	1	1	No	1	32.98200079

32	37	6	2		1	1	No	1	200
4	37	1	1	-3.21	1	1	No	1	7.451915927
30	37	6	2		1	1	No	1	200
10	37	2	1	-1.76	1	1	No	1	89.20818758
3	37	1	2	-1.31	1	1	No	1	9.323055383
24	37	6	2		1	1	No	1	200
5	42	1	1	2.76	4.3372E-05	1	No	1	200
35	42	6	3		0.126540492	1	No	1	200
41	42	7	3		1	1	No	1	200
7	390	1	1	0.46	0.379684037	1	Yes	6	200
382	390	11	5		1	1	Yes	6	200
351	352	5	2		1	1	No	1	200

Supplementary Table S2 C03_521v Variant Table

Reference mitogenome	Region	Type	Reference	Allele	Reference allele	Length	Linkage	Zygosity
NC_012920_rCRS H2a_haplo	73	SNV	A	G	No	1		Homozygous
NC_012920_rCRS H2a_haplo	146	SNV	T	C	No	1		Heterozygous
NC_012920_rCRS H2a_haplo	146	SNV	T	T	Yes	1		Heterozygous
NC_012920_rCRS H2a_haplo	150	SNV	C	T	No	1		Heterozygous
NC_012920_rCRS H2a_haplo	150	SNV	C	C	Yes	1		Heterozygous
NC_012920_rCRS H2a_haplo	152	SNV	T	C	No	1		Heterozygous
NC_012920_rCRS H2a_haplo	152	SNV	T	T	Yes	1		Heterozygous
NC_012920_rCRS H2a_haplo	189	SNV	A	G	No	1		Homozygous
NC_012920_rCRS H2a_haplo	200	SNV	A	G	No	1		Homozygous
NC_012920_rCRS H2a_haplo	263	SNV	A	G	No	1		Homozygous
NC_012920_rCRS H2a_haplo	302^303	Insertion	-	C	No	1		Heterozygous
NC_012920_rCRS H2a_haplo	302^303	Insertion	-	-	Yes	0		Heterozygous
NC_012920_rCRS H2a_haplo	310	SNV	T	C	No	1		Heterozygous
NC_012920_rCRS H2a_haplo	310	SNV	T	T	Yes	1		Heterozygous
NC_012920_rCRS H2a_haplo	310^311	Insertion	-	C	No	1		Heterozygous
NC_012920_rCRS H2a_haplo	310^311	Insertion	-	-	Yes	0		Heterozygous
NC_012920_rCRS H2a_haplo	351	SNV	A	G	No	1		Heterozygous
NC_012920_rCRS H2a_haplo	351	SNV	A	A	Yes	1		Heterozygous
NC_012920_rCRS H2a_haplo	440	SNV	A	T	No	1		Heterozygous
NC_012920_rCRS H2a_haplo	440	SNV	A	A	Yes	1		Heterozygous
NC_012920_rCRS H2a_haplo	16176	SNV	C	T	No	1		Heterozygous
NC_012920_rCRS H2a_haplo	16176	SNV	C	C	Yes	1		Heterozygous
NC_012920_rCRS H2a_haplo	16223	SNV	C	T	No	1		Heterozygous
NC_012920_rCRS H2a_haplo	16223	SNV	C	C	Yes	1		Heterozygous
NC_012920_rCRS H2a_haplo	16240..16241	Replacement	AA	T	No	2		Heterozygous
NC_012920_rCRS H2a_haplo	16240..16241	MNV	AA	AA	Yes	2		Heterozygous
NC_012920_rCRS H2a_haplo	16249	SNV	T	C	No	1		Heterozygous
NC_012920_rCRS H2a_haplo	16249	SNV	T	T	Yes	1		Heterozygous
NC_012920_rCRS H2a_haplo	16256	SNV	C	A	No	1		Heterozygous
NC_012920_rCRS H2a_haplo	16256	SNV	C	C	Yes	1		Heterozygous
NC_012920_rCRS H2a_haplo	16263	SNV	T	A	No	1		Heterozygous
NC_012920_rCRS H2a_haplo	16263	SNV	T	T	Yes	1		Heterozygous
NC_012920_rCRS H2a_haplo	16311	SNV	T	C	No	1		Heterozygous
NC_012920_rCRS H2a_haplo	16311	SNV	T	T	Yes	1		Heterozygous
NC_012920_rCRS H2a_haplo	16327	SNV	C	T	No	1		Heterozygous
NC_012920_rCRS H2a_haplo	16327	SNV	C	C	Yes	1		Heterozygous
NC_012920_rCRS H2a_haplo	16519	SNV	T	C	No	1		Homozygous

Supplementary Table S2 C03_521v Variant Table

**Count**	**Coverage**	**Frequency**	Probability	Forward read count	Reverse read count	Forward read coverage	Reverse read coverage	Forward/reverse balance	Average quality
24350	24405	99.77463635	1	24350	0	24405	0	0	63.44919918
4291	35768	11.99675688	1	4291	0	35768	0	0	32.97273363
31388	35768	87.75441736	1	31388	0	35768	0	0	37.60468969
31218	35768	87.27913219	1	31218	0	35768	0	0	37.56320072
4525	35768	12.65097294	1	4525	0	35768	0	0	36.23955801
29048	35772	81.20317567	1	29048	0	35772	0	0	38.13825392
6691	35772	18.70457341	1	6691	0	35772	0	0	37.1812883
11210	11400	98.33333333	1	11210	0	11400	0	0	34.03336307
11351	11400	99.57017544	1	11351	0	11400	0	0	37.74566118
11397	11399	99.9824546	1	11397	0	11399	0	0	63.12617355
234	11398	2.052991753	1	234	0	11398	0	0	34.0042735
11164	11398	97.94700825	1	11164	0	11398	0	0	31.18335722
193	11395	1.693725318	1	193	0	11395	0	0	34.49191591
11117	11395	97.56033348	1	11117	0	11395	0	0	26.58905617
11073	11395	97.17419921	1	11073	0	11395	0	0	37.36756554
319	11395	2.799473453	1	319	0	11395	0	0	31.93726847
146	11529	1.266371758	1	146	0	11529	0	0	34.70547945
11373	11529	98.64689045	1	11373	0	11529	0	0	36.22096193
2	138	1.449275362	0.999865822	2	0	138	0	0	59
136	138	98.55072464	1	136	0	138	0	0	61.61029412
4640	4723	98.24264239	1	4640	0	4723	0	0	37.33469828
78	4723	1.651492695	1	78	0	4723	0	0	34.79487179
281	288	97.56944444	1	281	0	288	0	0	38.15658363
7	288	2.430555556	0.999997004	7	0	288	0	0	34.57142857
3	292	1.02739726	1	3	0	292	0	0	35.66666666
289	292	98.97260274	1	289	0	292	0	0	38.31314879
4	294	1.360544218	0.999580743	4	0	294	0	0	44.5
289	294	98.29931973	1	289	0	294	0	0	39.05536332
3	294	1.020408163	0.996386568	3	0	294	0	0	62.66666667
289	294	98.29931973	1	289	0	294	0	0	63.31487889
161	455	35.38461538	1	161	0	455	0	0	63.06832298
294	455	64.61538462	1	294	0	455	0	0	63.46258503
6	457	1.312910284	0.999999196	6	0	457	0	0	46.66666667
451	457	98.68708972	1	451	0	457	0	0	52.20399113
446	457	97.59299781	1	446	0	457	0	0	47.00672646
11	457	2.407002188	1	11	0	457	0	0	39.18181818
8580	8593	99.84871407	1	8580	0	8593	0	0	38.3455711

Supplementary Table S2 C03_521v Variant Table

Read count	Read coverage	# unique start positions	# unique end positions	BaseQRankSum	Read position test probability	Read direction test probability	Homopolymer	Homopolymer length	QUAL
24350	24405	10	10		1	1	No	1	200
4291	35768	4	7	-97.52	0	1	No	1	200
31388	35768	16	22		0	1	No	1	200
31218	35768	14	19	66.16	0	1	No	1	200
4525	35768	6	10		0	1	No	1	200
29048	35772	16	17	83.02	0	1	No	1	200
6691	35772	6	11		0	1	No	1	200
11210	11400	9	11		0.623731848	1	No	1	200
11351	11400	9	11		0.328149205	1	No	1	200
11397	11399	9	11		1	1	No	1	200
234	11398	2	3	7.7	0.943686097	1	Yes	7	200
11164	11398	9	10		1	1	Yes	7	200
193	11395	2	4	6.25	7.2153E-10	1	No	1	200
11117	11395	9	8		0.336441455	1	Yes	7	200
11073	11395	9	8	15.54	0.337416492	1	Yes	5	200
319	11395	2	4		2.18648E-06	1	Yes	5	200
146	11529	3	6	1.76	0	1	No	1	200
11373	11529	10	9		0	1	No	1	200
2	138	1	1	-0.73	0.814494494	1	No	1	38.72317715
136	138	3	4		1	1	No	1	200
4640	4723	10	11	4.65	0.036146217	1	No	1	200
78	4723	4	7		0	1	No	1	200
281	288	11	3	3.47	1	1	No	1	200
7	288	3	2		1	1	No	1	55.23443695
3	292	1	1	-3.74	0	1	No	1	200
289	292	12	3		0.1986554	1	No	1	200
4	294	4	1	-0.45	0	1	No	1	33.77519885
289	294	11	3		0.182387195	1	No	1	200
3	294	3	1	-0.85	0	1	No	1	24.42080066
289	294	12	3		0.658374966	1	No	1	200
161	455	2	2	-1.39	0	1	No	1	200
294	455	14	3		0	1	No	1	200
6	457	5	2	-0.83	0.312325765	1	No	1	60.94743951
451	457	15	3		1	1	No	1	200
446	457	15	3	1.74	1	1	No	1	200
11	457	5	2		0.069617859	1	No	1	200
8580	8593	9	10		1	1	No	1	200

Supplementary Table S2 C04_523v Variant Table

Reference mitogenome	Region	Type	Reference	Allele	Reference allele	Length	Linkage	Zygosity
NC_012920_rCRS H2a_haplo	73	SNV	A	G	No	1		Homozygous
NC_012920_rCRS H2a_haplo	146	SNV	T	C	No	1		Heterozygous
NC_012920_rCRS H2a_haplo	146	SNV	T	T	Yes	1		Heterozygous
NC_012920_rCRS H2a_haplo	150	SNV	C	T	No	1		Heterozygous
NC_012920_rCRS H2a_haplo	150	SNV	C	C	Yes	1		Heterozygous
NC_012920_rCRS H2a_haplo	152	SNV	T	C	No	1		Heterozygous
NC_012920_rCRS H2a_haplo	152	SNV	T	T	Yes	1		Heterozygous
NC_012920_rCRS H2a_haplo	195	SNV	T	C	No	1		Homozygous
NC_012920_rCRS H2a_haplo	263	SNV	A	G	No	1		Homozygous
NC_012920_rCRS H2a_haplo	302^303	Insertion	-	C	No	1		Heterozygous
NC_012920_rCRS H2a_haplo	302^303	Insertion	-	-	Yes	0		Heterozygous
NC_012920_rCRS H2a_haplo	310	SNV	T	C	No	1		Heterozygous
NC_012920_rCRS H2a_haplo	310	SNV	T	T	Yes	1		Heterozygous
NC_012920_rCRS H2a_haplo	310^311	Insertion	-	C	No	1		Heterozygous
NC_012920_rCRS H2a_haplo	310^311	Insertion	-	-	Yes	0		Heterozygous
NC_012920_rCRS H2a_haplo	16093	SNV	T	C	No	1		Heterozygous
NC_012920_rCRS H2a_haplo	16093	SNV	T	T	Yes	1		Heterozygous
NC_012920_rCRS H2a_haplo	16172	SNV	T	C	No	1		Heterozygous
NC_012920_rCRS H2a_haplo	16172	SNV	T	T	Yes	1		Heterozygous
NC_012920_rCRS H2a_haplo	16223	SNV	C	T	No	1		Homozygous
NC_012920_rCRS H2a_haplo	16265	SNV	A	T	No	1		Homozygous
NC_012920_rCRS H2a_haplo	16519	SNV	T	C	No	1		Homozygous

Supplementary Table S2 C04_523v Variant Table

**Count**	**Coverage**	**Frequency**	Probability	Forward read count	Reverse read count	Forward read coverage	Reverse read coverage	Forward/reverse balance	Average quality
1099	1101	99.81834696	1	1099	0	1101	0	0	63.52684258
173	1577	10.97019658	1	173	0	1577	0	0	32.83815029
1399	1577	88.71274572	1	1399	0	1577	0	0	37.79413867
1382	1577	87.63474952	1	1382	0	1577	0	0	37.89580318
195	1577	12.36525048	1	195	0	1577	0	0	36.57435897
197	1577	12.49207356	1	197	0	1577	0	0	37.48730964
1379	1577	87.4445149	1	1379	0	1577	0	0	38.31254532
475	478	99.37238494	1	475	0	478	0	0	38.05473684
478	478	100	1	478	0	478	0	0	63.29079498
16	478	3.347280335	1	16	0	478	0	0	35.6875
462	478	96.65271967	1	462	0	478	0	0	32.37878788
6	478	1.255230126	0.999996061	6	0	478	0	0	36.66666667
469	478	98.11715481	1	469	0	478	0	0	27.69722814
467	478	97.69874477	1	467	0	478	0	0	37.50321199
11	478	2.30125523	1	11	0	478	0	0	26.72727273
216	224	96.42857143	1	216	0	224	0	0	63.27314815
8	224	3.571428571	1	8	0	224	0	0	60.875
8	262	3.053435115	0.999999999	8	0	262	0	0	37.5
252	262	96.18320611	1	252	0	262	0	0	37.15873016
147	147	100	1	147	0	147	0	0	38.06802721
146	147	99.31972789	1	146	0	147	0	0	62.60273973
425	425	100	1	425	0	425	0	0	38.18352941

Supplementary Table S2 C04_523v Variant Table

Read count	Read coverage	# unique start positions	# unique end positions	BaseQRankSum	Read position test probability	Read direction test probability	Homopolymer	Homopolymer length	QUAL
1099	1101	5	4		1	1	No	1	200
173	1577	2	3	-19.94	0	1	No	1	200
1399	1577	6	5		0.00011392	1	No	1	200
1382	1577	6	5	15.07	2.69985E-06	1	No	1	200
195	1577	2	3		0	1	No	1	200
197	1577	2	3	-14.07	0	1	No	1	200
1379	1577	6	5		2.50265E-06	1	No	1	200
475	478	1	2		1	1	No	1	200
478	478	1	2		1	1	No	1	200
16	478	1	1	2.96	1	1	Yes	7	200
462	478	1	2		1	1	Yes	7	200
6	478	1	1	1.89	1	1	No	1	54.04569919
469	478	2	3		1	1	Yes	7	200
467	478	2	3	2.82	1	1	Yes	5	200
11	478	1	1		1	1	Yes	5	200
216	224	2	2	0.41	0.336057701	1	No	1	200
8	224	1	2		0.00030828	1	No	1	200
8	262	2	2	-1.7	3.8409E-10	1	No	1	88.53871963
252	262	4	3		0.518941034	1	No	1	200
147	147	5	4		1	1	No	1	200
146	147	5	4		1	1	No	1	200
425	425	4	3		1	1	No	1	200

Supplementary Table S2 C05_524v Variant Table

Reference mitogenome	Region	Type	Reference	Allele	Reference allele	Length	Linkage	Zygosity
NC_012920_rCRS H2a_haplo	146	SNV	T	C	No	1		Heterozygous
NC_012920_rCRS H2a_haplo	146	SNV	T	T	Yes	1		Heterozygous
NC_012920_rCRS H2a_haplo	150	SNV	C	T	No	1		Heterozygous
NC_012920_rCRS H2a_haplo	150	SNV	C	C	Yes	1		Heterozygous
NC_012920_rCRS H2a_haplo	152	SNV	T	C	No	1		Heterozygous
NC_012920_rCRS H2a_haplo	152	SNV	T	T	Yes	1		Heterozygous
NC_012920_rCRS H2a_haplo	263	SNV	A	G	No	1		Homozygous
NC_012920_rCRS H2a_haplo	302^303	Insertion	-	C	No	1		Heterozygous
NC_012920_rCRS H2a_haplo	302^303	Insertion	-	-	Yes	0		Heterozygous
NC_012920_rCRS H2a_haplo	310	SNV	T	C	No	1		Heterozygous
NC_012920_rCRS H2a_haplo	310	SNV	T	T	Yes	1		Heterozygous
NC_012920_rCRS H2a_haplo	310^311	Insertion	-	C	No	1		Heterozygous
NC_012920_rCRS H2a_haplo	310^311	Insertion	-	-	Yes	0		Heterozygous
NC_012920_rCRS H2a_haplo	16172	SNV	T	C	No	1		Heterozygous
NC_012920_rCRS H2a_haplo	16172	SNV	T	T	Yes	1		Heterozygous
NC_012920_rCRS H2a_haplo	16263	SNV	T	A	No	1		Heterozygous
NC_012920_rCRS H2a_haplo	16263	SNV	T	T	Yes	1		Heterozygous
NC_012920_rCRS H2a_haplo	16519	SNV	T	C	No	1		Homozygous

Supplementary Table S2 C05_524v Variant Table

**Count**	**Coverage**	**Frequency**	Probability	Forward read count	Reverse read count	Forward read coverage	Reverse read coverage	Forward/reverse balance	Average quality
146	1361	10.72740632	1	146	0	1361	0	0	32.98630137
1211	1361	88.97869214	1	1211	0	1361	0	0	37.34682081
291	1361	21.38133725	1	291	0	1361	0	0	36.48797251
1069	1361	78.54518736	1	1069	0	1361	0	0	38.05799813
169	1361	12.41734019	1	169	0	1361	0	0	36.47337278
1192	1361	87.58265981	1	1192	0	1361	0	0	37.97147651
420	420	100	1	420	0	420	0	0	62.50952381
10	419	2.386634845	1	10	0	419	0	0	31.5
409	419	97.61336516	1	409	0	419	0	0	30.67726161
7	418	1.674641148	0.999995206	7	0	418	0	0	29.42857143
404	418	96.6507177	1	404	0	418	0	0	25.8019802
403	418	96.41148325	1	403	0	418	0	0	36.89826303
15	418	3.588516746	1	15	0	418	0	0	27.4
4	202	1.98019802	0.999815996	4	0	202	0	0	31.5
198	202	98.01980198	1	198	0	202	0	0	36.52020202
6	96	6.25	1	6	0	96	0	0	61.66666667
90	96	93.75	1	90	0	96	0	0	62.86666667
303	305	99.3442623	1	303	0	305	0	0	38.07260726

Supplementary Table S2 C05_524v Variant Table

Read count	Read coverage	# unique start positions	# unique end positions	BaseQRankSum	Read position test probability	Read direction test probability	Homopolymer	Homopolymer length	QUAL
146	1361	2	5	-17.4	0	1	No	1	200
1211	1361	7	7		0.001381862	1	No	1	200
291	1361	3	5	-19.65	0	1	No	1	200
1069	1361	6	7		0	1	No	1	200
169	1361	4	4	-11.22	0	1	No	1	200
1192	1361	6	7		0.000187141	1	No	1	200
420	420	3	4		1	1	No	1	200
10	419	1	1	0.72	1	1	Yes	7	200
409	419	3	4		1	1	Yes	7	200
7	418	1	1	0.41	1	1	No	1	53.1931103
404	418	3	3		1	1	Yes	7	200
403	418	3	3	4.38	1	1	Yes	5	200
15	418	1	1		1	1	Yes	5	200
4	202	2	2	-1.39	0.000350257	1	No	1	37.3517368
198	202	4	4		0.8165732	1	No	1	200
6	96	1	1	-0.42	6.20475E-11	1	No	1	200
90	96	6	1		0.066457546	1	No	1	200
303	305	6	1		1	1	No	1	200

Supplementary Table S2 C06_525v Variant Table

Reference mitogenome	Region	Type	Reference	Allele	Reference allele	Length	Linkage	Zygosity
NC_012920_rCRS H2a_haplo	73	SNV	A	G	No	1		Homozygous
NC_012920_rCRS H2a_haplo	146	SNV	T	C	No	1		Heterozygous
NC_012920_rCRS H2a_haplo	146	SNV	T	T	Yes	1		Heterozygous
NC_012920_rCRS H2a_haplo	150	SNV	C	T	No	1		Heterozygous
NC_012920_rCRS H2a_haplo	150..151	MNV	CC	TT	No	2		Heterozygous
NC_012920_rCRS H2a_haplo	150..151	MNV	CC	CC	Yes	2		Heterozygous
NC_012920_rCRS H2a_haplo	152	SNV	T	C	No	1		Heterozygous
NC_012920_rCRS H2a_haplo	152	SNV	T	T	Yes	1		Heterozygous
NC_012920_rCRS H2a_haplo	207	SNV	G	A	No	1		Heterozygous
NC_012920_rCRS H2a_haplo	207	SNV	G	G	Yes	1		Heterozygous
NC_012920_rCRS H2a_haplo	217	SNV	T	C	No	1		Heterozygous
NC_012920_rCRS H2a_haplo	217	SNV	T	T	Yes	1		Heterozygous
NC_012920_rCRS H2a_haplo	263	SNV	A	G	No	1		Homozygous
NC_012920_rCRS H2a_haplo	302^303	Insertion	-	C	No	1		Heterozygous
NC_012920_rCRS H2a_haplo	302^303	Insertion	-	CC	No	2		Heterozygous
NC_012920_rCRS H2a_haplo	302^303	Insertion	-	-	Yes	0		Heterozygous
NC_012920_rCRS H2a_haplo	310^311	Insertion	-	C	No	1		Homozygous
NC_012920_rCRS H2a_haplo	340	SNV	C	T	No	1		Heterozygous
NC_012920_rCRS H2a_haplo	340	SNV	C	C	Yes	1		Heterozygous
NC_012920_rCRS H2a_haplo	351	SNV	A	G	No	1		Heterozygous
NC_012920_rCRS H2a_haplo	351	SNV	A	A	Yes	1		Heterozygous
NC_012920_rCRS H2a_haplo	16067	SNV	C	T	No	1		Heterozygous
NC_012920_rCRS H2a_haplo	16067	SNV	C	C	Yes	1		Heterozygous
NC_012920_rCRS H2a_haplo	16172	SNV	T	C	No	1		Heterozygous
NC_012920_rCRS H2a_haplo	16172	SNV	T	T	Yes	1		Heterozygous
NC_012920_rCRS H2a_haplo	16223	SNV	C	T	No	1		Heterozygous
NC_012920_rCRS H2a_haplo	16223	SNV	C	C	Yes	1		Heterozygous
NC_012920_rCRS H2a_haplo	16249	SNV	T	C	No	1		Heterozygous
NC_012920_rCRS H2a_haplo	16249	SNV	T	T	Yes	1		Heterozygous
NC_012920_rCRS H2a_haplo	16256	SNV	C	A	No	1		Heterozygous
NC_012920_rCRS H2a_haplo	16256	SNV	C	C	Yes	1		Heterozygous
NC_012920_rCRS H2a_haplo	16263	SNV	T	A	No	1		Heterozygous
NC_012920_rCRS H2a_haplo	16263	SNV	T	T	Yes	1		Heterozygous
NC_012920_rCRS H2a_haplo	16269	SNV	A	G	No	1		Heterozygous
NC_012920_rCRS H2a_haplo	16269	SNV	A	A	Yes	1		Heterozygous
NC_012920_rCRS H2a_haplo	16271	SNV	T	C	No	1		Homozygous
NC_012920_rCRS H2a_haplo	16301	SNV	C	T	No	1		Heterozygous
NC_012920_rCRS H2a_haplo	16301	SNV	C	C	Yes	1		Heterozygous

Supplementary Table S2 C06_525v Variant Table

**Count**	**Coverage**	**Frequency**	Probability	Forward read count	Reverse read count	Forward read coverage	Reverse read coverage	Forward/reverse balance	Average quality
762	763	99.8689384	1	762	0	763	0	0	63.5144357
26	842	3.087885986	1	26	0	842	0	0	32.46153846
814	842	96.67458432	1	814	0	842	0	0	37.92997543
53	842	6.294536817	1	53	0	842	0	0	36.33838324
750	842	89.0736342	1	750	0	842	0	0	38.13720681
38	842	4.513064133	1	38	0	842	0	0	36.1310809
40	842	4.750593824	1	40	0	842	0	0	34.525
802	842	95.24940618	1	802	0	842	0	0	38.24314214
75	79	94.93670886	1	75	0	79	0	0	38.33333333
4	79	5.063291139	0.999999997	4	0	79	0	0	37
2	79	2.53164557	0.998529922	2	0	79	0	0	39
77	79	97.46835443	1	77	0	79	0	0	38.02597403
79	79	100	1	79	0	79	0	0	63.2278481
63	79	79.74683544	1	63	0	79	0	0	35.47619048
6	79	7.594936709	1	6	0	79	0	0	36.16666667
10	79	12.65822785	1	10	0	79	0	0	31.2
77	79	97.46835443	1	77	0	79	0	0	37.72727273
3	83	3.614457831	0.99999999	3	0	83	0	0	31.33333333
80	83	96.38554217	1	80	0	83	0	0	38.3125
4	83	4.819277108	0.999999995	4	0	83	0	0	36.75
79	83	95.18072289	1	79	0	83	0	0	36.24050633
6	124	4.838709677	1	6	0	124	0	0	38.66666667
118	124	95.16129032	1	118	0	124	0	0	37.98305085
3	139	2.158273381	0.914107933	3	0	139	0	0	38
136	139	97.84172662	1	136	0	139	0	0	36.64705882
98	100	98	1	98	0	100	0	0	38.17346939
2	100	2	0.960186272	2	0	100	0	0	38.5
3	102	2.941176471	0.997835876	3	0	102	0	0	38.33333333
99	102	97.05882353	1	99	0	102	0	0	38.68686869
2	103	1.941747573	0.927910285	2	0	103	0	0	64
101	103	98.05825243	1	101	0	103	0	0	63.32673267
13	116	11.20689655	1	13	0	116	0	0	56.15384615
103	116	88.79310345	1	103	0	116	0	0	59.00970874
115	117	98.29059829	1	115	0	117	0	0	62.63478261
2	117	1.709401709	0.935492622	2	0	117	0	0	64
115	117	98.29059829	1	115	0	117	0	0	63.33913043
4	117	3.418803419	0.999492703	4	0	117	0	0	61.5
113	117	96.58119658	1	113	0	117	0	0	63.26548673

Supplementary Table S2 C06_525v Variant Table

Read count	Read coverage	# unique start positions	# unique end positions	BaseQRankSum	Read position test probability	Read direction test probability	Homopolymer	Homopolymer length	QUAL
762	763	3	3		1	1	No	1	200
26	842	2	3	-8.12	0	1	No	1	200
814	842	4	4		0.099692345	1	No	1	200
53	842	2	2	-0.85	0	1	No	1	200
750	842	3	3	10.6	1.11022E-16	1	No	1	200
38	842	2	2		0	1	No	1	200
40	842	2	2	-7.04	0	1	No	1	200
802	842	4	4		0.012831561	1	No	1	200
75	79	1	1	0.65	1	1	No	1	200
4	79	1	1		1	1	No	1	85.85026653
2	79	1	1	0.47	1	1	No	1	28.3265971
77	79	1	1		1	1	No	1	200
79	79	1	1		1	1	No	1	200
63	79	1	1	3.87	1	1	Yes	7	200
6	79	1	1	2.96	1	1	Yes	7	200
10	79	1	1		1	1	Yes	7	200
77	79	1	1		1	1	No	1	200
3	83	1	1	-2.34	0.710894748	1	No	1	80.04364804
80	83	2	2		1	1	No	1	200
4	83	1	1	0.28	1.63763E-10	1	No	1	83.18758767
79	83	1	1		0.055857022	1	No	1	200
6	124	1	2	-0.14	1	1	No	1	200
118	124	2	2		1	1	No	1	200
3	139	1	1	-0.67	5.23219E-06	1	No	1	10.66046943
136	139	4	4		0.67683887	1	No	1	200
98	100	6	3	0.48	1	1	No	1	200
2	100	1	1		1	1	No	1	13.99967152
3	102	3	1	-1.45	9.03691E-09	1	No	1	26.64717879
99	102	6	3		0.172259325	1	No	1	200
2	103	2	1	0.1	1.59411E-09	1	No	1	11.4212669
101	103	7	3		0.642611523	1	No	1	200
13	116	1	1	-1.26	3.26466E-11	1	No	1	200
103	116	9	3		0.026126634	1	No	1	200
115	117	11	3	-0.17	1	1	No	1	200
2	117	1	1		0.855868877	1	No	1	11.90390613
115	117	11	3	1.06	1	1	No	1	200
4	117	1	1	-0.69	0.449495345	1	No	1	32.94737449
113	117	11	3		1	1	No	1	200

Supplementary Table S2 C07_528v Variant Table

Reference mitogenome	Region	Type	Reference	Allele	Reference allele	Length	Linkage	Zygosity
NC_012920_rCRS H2a_haplo	73	SNV	A	G	No	1		Heterozygous
NC_012920_rCRS H2a_haplo	73	SNV	A	A	Yes	1		Heterozygous
NC_012920_rCRS H2a_haplo	146	SNV	T	C	No	1		Heterozygous
NC_012920_rCRS H2a_haplo	146	SNV	T	T	Yes	1		Heterozygous
NC_012920_rCRS H2a_haplo	150	SNV	C	T	No	1		Heterozygous
NC_012920_rCRS H2a_haplo	150	SNV	C	C	Yes	1		Heterozygous
NC_012920_rCRS H2a_haplo	152	SNV	T	C	No	1		Heterozygous
NC_012920_rCRS H2a_haplo	152	SNV	T	T	Yes	1		Heterozygous
NC_012920_rCRS H2a_haplo	195	SNV	T	C	No	1		Heterozygous
NC_012920_rCRS H2a_haplo	195	SNV	T	T	Yes	1		Heterozygous
NC_012920_rCRS H2a_haplo	204	SNV	T	C	No	1		Heterozygous
NC_012920_rCRS H2a_haplo	204	SNV	T	T	Yes	1		Heterozygous
NC_012920_rCRS H2a_haplo	263	SNV	A	G	No	1		Homozygous
NC_012920_rCRS H2a_haplo	297	SNV	A	C	No	1		Heterozygous
NC_012920_rCRS H2a_haplo	297	SNV	A	A	Yes	1		Heterozygous
NC_012920_rCRS H2a_haplo	302	Replacement	A	CCC	No	3		Heterozygous
NC_012920_rCRS H2a_haplo	302	SNV	A	A	Yes	1		Heterozygous
NC_012920_rCRS H2a_haplo	302^303	Insertion	-	C	No	1		Heterozygous
NC_012920_rCRS H2a_haplo	302^303	Insertion	-	CC	No	2		Heterozygous
NC_012920_rCRS H2a_haplo	302^303	Insertion	-	CCC	No	3		Heterozygous
NC_012920_rCRS H2a_haplo	302^303	Insertion	-	CCCC	No	4		Heterozygous
NC_012920_rCRS H2a_haplo	302^303	Insertion	-	-	Yes	0		Heterozygous
NC_012920_rCRS H2a_haplo	310	SNV	T	C	No	1		Heterozygous
NC_012920_rCRS H2a_haplo	310	Replacement	T	CCGC	No	4		Heterozygous
NC_012920_rCRS H2a_haplo	310	SNV	T	T	Yes	1		Heterozygous
NC_012920_rCRS H2a_haplo	310^311	Insertion	-	C	No	1		Heterozygous
NC_012920_rCRS H2a_haplo	310^311	Insertion	-	-	Yes	0		Heterozygous
NC_012920_rCRS H2a_haplo	351	SNV	A	G	No	1		Heterozygous
NC_012920_rCRS H2a_haplo	351	SNV	A	A	Yes	1		Heterozygous
NC_012920_rCRS H2a_haplo	16143	SNV	T	C	No	1		Heterozygous
NC_012920_rCRS H2a_haplo	16143	SNV	T	T	Yes	1		Heterozygous
NC_012920_rCRS H2a_haplo	16149	SNV	A	C	No	1		Heterozygous
NC_012920_rCRS H2a_haplo	16149	SNV	A	A	Yes	1		Heterozygous
NC_012920_rCRS H2a_haplo	16152	SNV	T	C	No	1		Heterozygous
NC_012920_rCRS H2a_haplo	16152	SNV	T	T	Yes	1		Heterozygous
NC_012920_rCRS H2a_haplo	16161	SNV	T	A	No	1		Heterozygous
NC_012920_rCRS H2a_haplo	16161	SNV	T	T	Yes	1		Heterozygous
NC_012920_rCRS H2a_haplo	16172	SNV	T	A	No	1		Heterozygous
NC_012920_rCRS H2a_haplo	16172	SNV	T	T	Yes	1		Heterozygous
NC_012920_rCRS H2a_haplo	16176	SNV	C	T	No	1		Heterozygous
NC_012920_rCRS H2a_haplo	16176	SNV	C	C	Yes	1		Heterozygous
NC_012920_rCRS H2a_haplo	16178	SNV	T	A	No	1		Heterozygous
NC_012920_rCRS H2a_haplo	16178	SNV	T	T	Yes	1		Heterozygous
NC_012920_rCRS H2a_haplo	16180	Deletion	A	-	No	1		Heterozygous
NC_012920_rCRS H2a_haplo	16180	SNV	A	A	Yes	1		Heterozygous
NC_012920_rCRS H2a_haplo	.6182..16183	Replacement	AA	CCC	No	3		Heterozygous
NC_012920_rCRS H2a_haplo	.6182..16183	MNV	AA	CC	No	2		Heterozygous
NC_012920_rCRS H2a_haplo	.6182..16183	MNV	AA	AA	Yes	2		Heterozygous
NC_012920_rCRS H2a_haplo	16183	SNV	A	C	No	1		Heterozygous
NC_012920_rCRS H2a_haplo	16189	SNV	T	C	No	1		Homozygous
NC_012920_rCRS H2a_haplo	16211	SNV	C	A	No	1		Heterozygous
NC_012920_rCRS H2a_haplo	16211	SNV	C	C	Yes	1		Heterozygous

NC_012920_rCRS H2a_haplo	16217	SNV	T	C	No	1		Heterozygous
NC_012920_rCRS H2a_haplo	16217	SNV	T	T	Yes	1		Heterozygous
NC_012920_rCRS H2a_haplo	16229	SNV	T	A	No	1		Heterozygous
NC_012920_rCRS H2a_haplo	16229	SNV	T	T	Yes	1		Heterozygous
NC_012920_rCRS H2a_haplo	16249	SNV	T	C	No	1		Heterozygous
NC_012920_rCRS H2a_haplo	16249	SNV	T	T	Yes	1		Heterozygous
NC_012920_rCRS H2a_haplo	16263	SNV	T	A	No	1		Heterozygous
NC_012920_rCRS H2a_haplo	16263	SNV	T	T	Yes	1		Heterozygous
NC_012920_rCRS H2a_haplo	16360	SNV	C	T	No	1		Heterozygous
NC_012920_rCRS H2a_haplo	16360	SNV	C	C	Yes	1		Heterozygous
NC_012920_rCRS H2a_haplo	16519	SNV	T	C	No	1		Homozygous
NC_012920_rCRS H2a_haplo	16536	Deletion	C	-	No	1		Heterozygous
NC_012920_rCRS H2a_haplo	16536	SNV	C	C	Yes	1		Heterozygous

Supplementary Table S2 C07_528v Variant Table

**Count**	**Coverage**	**Frequency**	Probability	Forward read count	Reverse read count	Forward read coverage	Reverse read coverage	Forward/reverse balance	Average quality
453	468	96.79487179	1	453	0	468	0	0	63.52759382
8	468	1.709401709	0.999823733	8	0	468	0	0	52.8125
103	732	14.07103825	1	103	0	732	0	0	33.12621359
625	732	85.38251366	1	625	0	732	0	0	37.4928
169	733	23.05593452	1	169	0	733	0	0	36.20118343
563	733	76.80763984	1	563	0	733	0	0	37.75843694
119	733	16.23465211	1	119	0	733	0	0	36.79831933
613	733	83.62892224	1	613	0	733	0	0	37.69983687
3	265	1.132075472	0.98583152	3	0	265	0	0	32
262	265	98.86792453	1	262	0	265	0	0	38.44656489
35	265	13.20754717	1	35	0	265	0	0	38.22857143
230	265	86.79245283	1	230	0	265	0	0	36.83043478
264	264	100	1	264	0	264	0	0	58.35227273
20	264	7.575757576	0.991770675	20	0	264	0	0	14.1
242	264	91.66666667	1	242	0	264	0	0	24.41322314
12	264	4.545454545	1	12	0	264	0	0	28.15
246	264	93.18181818	1	246	0	264	0	0	26.64227642
14	264	5.303030303	1	14	0	264	0	0	35.42857143
145	264	54.92424242	1	145	0	264	0	0	34.62413793
69	264	26.13636364	1	69	0	264	0	0	33.14492754
4	264	1.515151515	0.999996959	4	0	264	0	0	31.375
31	264	11.74242424	1	31	0	264	0	0	27.06451613
4	264	1.515151515	0.970249178	4	0	264	0	0	25
3	264	1.136363636	0.99999649	3	0	264	0	0	19.46666667
253	264	95.83333333	1	253	0	264	0	0	24.49011858
250	264	94.6969697	1	250	0	264	0	0	36.864
14	264	5.303030303	1	14	0	264	0	0	25.85714286
4	267	1.498127341	0.9999991	4	0	267	0	0	32
262	267	98.12734082	1	262	0	267	0	0	35.04198473
5	57	8.771929825	0.903022828	5	0	57	0	0	15
51	57	89.47368421	1	51	0	57	0	0	20.54901961
21	57	36.84210526	0.999999051	21	0	57	0	0	12.66666667
35	57	61.40350877	1	35	0	57	0	0	20.11428571
4	57	7.01754386	0.791219735	4	0	57	0	0	19.25
53	57	92.98245614	1	53	0	57	0	0	22
8	59	13.55932203	0.774019703	8	0	59	0	0	12
48	59	81.3559322	1	48	0	59	0	0	17.97916667
8	59	13.55932203	0.994996105	8	0	59	0	0	14.25
50	59	84.74576271	1	50	0	59	0	0	18.84
2	65	3.076923077	0.878085642	2	0	65	0	0	28
63	65	96.92307692	1	63	0	65	0	0	24.58730159
13	65	20	1	13	0	65	0	0	17.23076923
50	65	76.92307692	1	50	0	65	0	0	21.92
14	65	21.53846154	1	14	0	65	0	0	28.78571429
49	65	75.38461538	1	49	0	65	0	0	27.14285714
22	65	33.84615385	1	22	0	65	0	0	32.09090909
24	65	36.92307692	1	24	0	65	0	0	31.5
3	65	4.615384615	1	3	0	65	0	0	36.44444444
15	65	23.07692308	1	15	0	65	0	0	32.86666667
65	66	98.48484848	1	65	0	66	0	0	36.30769231
3	73	4.109589041	0.999955862	3	0	73	0	0	24
69	73	94.52054795	1	69	0	73	0	0	34.76811594

16	73	21.91780822	1	16	0	73	0	0	32.5
53	73	72.60273973	1	53	0	73	0	0	32.26415094
2	16	12.5	0.99640169	2	0	16	0	0	20.5
13	16	81.25	1	13	0	16	0	0	29.92307692
4	17	23.52941176	0.974083055	4	0	17	0	0	14.75
13	17	76.47058824	1	13	0	17	0	0	32.61538462
8	25	32	1	8	0	25	0	0	64
17	25	68	1	17	0	25	0	0	40.58823529
24	57	42.10526316	1	24	0	57	0	0	41.41666667
32	57	56.14035088	1	32	0	57	0	0	37
32	32	100	1	32	0	32	0	0	38.53125
5	500	1	0.999350156	5	0	500	0	0	35.8
495	500	99	1	495	0	500	0	0	37.31313131

Supplementary Table S2 C07_528v Variant Table

Read count	Read coverage	# unique start positions	# unique end positions	Read position test probability	Read direction test probability	BaseQRankSum	Homopolymer	Homopolymer length	QUAL
453	468	3	4	1	1	3.28	No	1	200
8	468	1	1	1	1		No	1	37.5382948
103	732	3	2	0	1	-14.6	No	1	200
625	732	4	5	0.000649058	1		No	1	200
169	733	4	2	0	1	-14.12	No	1	200
563	733	3	6	9.34142E-12	1		No	1	200
119	733	4	4	0	1	-8.85	No	1	200
613	733	4	4	0.000632139	1		No	1	200
3	265	1	1	0.917418691	1	-0.67	No	1	18.48676751
262	265	3	3	1	1		No	1	200
35	265	1	1	0.725651261	1	2.42	No	1	200
230	265	3	3	0.942554096	1		No	1	200
264	264	3	2	1	1		No	1	200
20	264	2	1	1	1	-4.89	No	1	20.84635807
242	264	3	2	1	1		No	1	200
12	264	1	1	1	1	2.56	No	1	200
246	264	3	2	1	1		Yes	7	200
14	264	1	1	1	1	7.72	Yes	7	200
145	264	3	2	1	1	11.68	Yes	7	200
69	264	1	1	1	1	8.13	Yes	7	200
4	264	1	1	1	1	1.45	Yes	7	55.16926458
31	264	2	1	1	1		Yes	7	200
4	264	1	1	1	1	-1.37	No	1	15.26501033
3	264	2	1	1	1	-1.98	No	1	54.54668138
253	264	4	3	1	1		Yes	7	200
250	264	4	3	1	1	4.1	Yes	5	200
14	264	2	1	1	1		Yes	5	200
4	267	2	2	0	1	0	No	1	60.45516283
262	267	3	2	0.092740187	1		No	1	200
5	57	1	1	1	1	-2.66	No	1	10.13330483
51	57	1	2	1	1		No	1	200
21	57	1	1	1	1	-6.22	No	1	60.22779553
35	57	1	2	1	1		No	1	200
4	57	1	1	1	1	-1.66	No	1	6.803105559
53	57	1	2	1	1		No	1	200
8	59	1	1	0.614424501	1	-3.75	No	1	6.459294257
48	59	2	3	0.867397401	1		No	1	200
8	59	1	1	0.614424501	1	-2.89	No	1	23.00691812
50	59	2	3	0.899824262	1		No	1	200
2	65	1	1	0.481530385	1	0.8	No	1	9.139451433
63	65	3	3	1	1		No	1	200
13	65	1	1	0.205800667	1	-3.01	No	1	200
50	65	3	3	0.654221252	1		No	1	200
14	65	3	3	0.147591112	1	0.55	Yes	4	200
49	65	3	3	0.543082173	1		Yes	4	200
22	65	2	2	0.343193006	1	-1.19	Yes	5	200
24	65	3	3	1	1	-0.87	No	1	200
3	65	2	2	0.310208622	1		No	1	200
15	65	3	3	0.484156987	1	-1.58	No	1	200
65	66	4	3	1	1		No	1	200
3	73	1	1	0.000118828	1	-1.61	No	1	43.55188334
69	73	6	3	0.804544312	1		No	1	200

16	73	5	1	6.3558E-08	1	0.65	No	1	200
53	73	1	2	0.002155272	1		No	1	200
2	16	2	1	1	1	-1.36	No	1	24.43901436
13	16	5	1	1	1		No	1	200
4	17	3	1	0.636095861	1	-2.94	No	1	15.86416196
13	17	6	1	0.871091114	1		No	1	200
8	25	1	1	0.009412575	1	2.33	No	1	200
17	25	6	1	0.100814526	1		No	1	200
24	57	7	1	3.0396E-05	1	0.23	No	1	200
32	57	2	1	0.000116517	1		No	1	200
32	32	2	1	1	1		No	1	200
5	500	1	1	0.574676744	1	-3.23	Yes	3	31.8719082
495	500	4	4	1	1		Yes	3	200

Supplementary Table S2 C08_529v Variant Table

Reference mitogenome	Region	Type	Reference	Allele	Reference allele	Length	Linkage	Zygosity
NC_012920_rCRS H2a_haplo	73	SNV	A	G	No	1		Homozygous
NC_012920_rCRS H2a_haplo	146	SNV	T	C	No	1		Heterozygous
NC_012920_rCRS H2a_haplo	146	SNV	T	T	Yes	1		Heterozygous
NC_012920_rCRS H2a_haplo	150	SNV	C	T	No	1		Heterozygous
NC_012920_rCRS H2a_haplo	150	SNV	C	C	Yes	1		Heterozygous
NC_012920_rCRS H2a_haplo	152	SNV	T	C	No	1		Heterozygous
NC_012920_rCRS H2a_haplo	152	SNV	T	T	Yes	1		Heterozygous
NC_012920_rCRS H2a_haplo	263	SNV	A	G	No	1		Homozygous
NC_012920_rCRS H2a_haplo	297	SNV	A	G	No	1		Heterozygous
NC_012920_rCRS H2a_haplo	297	SNV	A	A	Yes	1		Heterozygous
NC_012920_rCRS H2a_haplo	302	Replacement	A	CCC	No	3		Heterozygous
NC_012920_rCRS H2a_haplo	302	SNV	A	A	Yes	1		Heterozygous
NC_012920_rCRS H2a_haplo	302^303	Insertion	-	C	No	1		Heterozygous
NC_012920_rCRS H2a_haplo	302^303	Insertion	-	CC	No	2		Heterozygous
NC_012920_rCRS H2a_haplo	302^303	Insertion	-	CCC	No	3		Heterozygous
NC_012920_rCRS H2a_haplo	302^303	Insertion	-	-	Yes	0		Heterozygous
NC_012920_rCRS H2a_haplo	310	SNV	T	C	No	1		Heterozygous
NC_012920_rCRS H2a_haplo	310	SNV	T	T	Yes	1		Heterozygous
NC_012920_rCRS H2a_haplo	310^311	Insertion	-	C	No	1		Heterozygous
NC_012920_rCRS H2a_haplo	310^311	Insertion	-	-	Yes	0		Heterozygous
NC_012920_rCRS H2a_haplo	351	SNV	A	G	No	1		Heterozygous
NC_012920_rCRS H2a_haplo	351	SNV	A	A	Yes	1		Heterozygous
NC_012920_rCRS H2a_haplo	16172	SNV	T	C	No	1		Heterozygous
NC_012920_rCRS H2a_haplo	16172	SNV	T	T	Yes	1		Heterozygous
NC_012920_rCRS H2a_haplo	16219	SNV	A	G	No	1		Heterozygous
NC_012920_rCRS H2a_haplo	16219	SNV	A	A	Yes	1		Heterozygous
NC_012920_rCRS H2a_haplo	16223	SNV	C	T	No	1		Homozygous
NC_012920_rCRS H2a_haplo	16263	SNV	T	A	No	1		Heterozygous
NC_012920_rCRS H2a_haplo	16263	SNV	T	T	Yes	1		Heterozygous
NC_012920_rCRS H2a_haplo	16290	SNV	C	T	No	1		Homozygous
NC_012920_rCRS H2a_haplo	16519	SNV	T	C	No	1		Homozygous

Supplementary Table S2 C08_529v Variant Table

**Count**	**Coverage**	**Frequency**	Probability	Forward read count	Reverse read count	Forward read coverage	Reverse read coverage	Forward/reverse balance	Average quality
1154	1155	99.91341991	1	1154	0	1155	0	0	63.29896014
154	1560	9.871794872	1	154	0	1560	0	0	32.93506494
1403	1560	89.93589744	1	1403	0	1560	0	0	37.50748396
245	1560	15.70512821	1	245	0	1560	0	0	36.33877551
1313	1560	84.16666667	1	1313	0	1560	0	0	38.00609292
181	1560	11.6025641	1	181	0	1560	0	0	36.71823204
1378	1560	88.33333333	1	1378	0	1560	0	0	37.86865022
407	407	100	1	407	0	407	0	0	60.91891892
10	406	2.463054187	0.723717281	10	0	406	0	0	18.2
380	406	93.59605911	1	380	0	406	0	0	26.52631579
6	406	1.477832512	0.999999944	6	0	406	0	0	28.45833333
393	406	96.79802956	1	393	0	406	0	0	28.30852417
120	406	29.55665025	1	120	0	406	0	0	34.925
235	406	57.8817734	1	235	0	406	0	0	35.4212766
23	406	5.665024631	1	23	0	406	0	0	32.55072464
27	406	6.650246305	1	27	0	406	0	0	29.51851852
9	406	2.216748768	0.999999993	9	0	406	0	0	32.66666667
397	406	97.78325123	1	397	0	406	0	0	25.25692695
397	406	97.78325123	1	397	0	406	0	0	37.10831234
9	406	2.216748768	1	9	0	406	0	0	32.66666667
9	413	2.179176755	0.999999994	9	0	413	0	0	30.44444444
404	413	97.82082324	1	404	0	413	0	0	35.93316832
217	243	89.30041152	1	217	0	243	0	0	37.23041475
26	243	10.69958848	1	26	0	243	0	0	37.80769231
110	321	34.26791277	1	110	0	321	0	0	36.3
211	321	65.73208723	1	211	0	321	0	0	31.81990521
110	110	100	1	110	0	110	0	0	37.55454545
9	120	7.5	1	9	0	120	0	0	64
111	120	92.5	1	111	0	120	0	0	62.81081081
120	120	100	1	120	0	120	0	0	62.16666667
369	369	100	1	369	0	369	0	0	38.33333333

Supplementary Table S2 C08_529v Variant Table

Read count	Read coverage	# unique start positions	# unique end positions	BaseQRankSum	Read position test probability	Read direction test probability	Homopolymer	Homopolymer length	QUAL
1154	1155	4	5		1	1	No	1	200
154	1560	2	4	-18.16	0	1	No	1	200
1403	1560	6	7		7.39108E-05	1	No	1	200
245	1560	3	3	-18.78	0	1	No	1	200
1313	1560	5	8		1.19904E-14	1	No	1	200
181	1560	2	3	-12.31	0	1	No	1	200
1378	1560	6	8		1.17006E-05	1	No	1	200
407	407	2	3		1	1	No	1	200
10	406	1	1	-1.94	1	1	No	1	5.586462792
380	406	2	2		1	1	No	1	200
6	406	1	1	1.11	1	1	No	1	72.53365801
393	406	2	2		1	1	Yes	7	200
120	406	2	2	7.23	1	1	Yes	7	200
235	406	1	1	8.62	1	1	Yes	7	200
23	406	1	1	3.14	1	1	Yes	7	200
27	406	1	1		1	1	Yes	7	200
9	406	1	1	2.83	1	1	No	1	81.80456065
397	406	2	2		1	1	Yes	7	200
397	406	2	2	1.77	1	1	Yes	5	200
9	406	1	1		1	1	Yes	5	200
9	413	3	3	-1.25	0	1	No	1	82.14670169
404	413	2	2		0.010351738	1	No	1	200
217	243	3	5	0.07	2.24753E-05	1	No	1	200
26	243	2	2		0	1	No	1	200
110	321	3	3	12.38	0	1	No	1	200
211	321	2	3		0	1	No	1	200
110	110	3	3		1	1	No	1	200
9	120	1	1	0.36	1.2369E-12	1	No	1	200
111	120	4	3		0.040148621	1	No	1	200
120	120	5	3		1	1	No	1	200
369	369	4	3		1	1	No	1	200

Reference mitogenome	Region	Type	Reference	Allele	Reference allele	Length	Linkage	Zygosity
NC_012920_rCRS H2a_haplo	73	SNV	A	G	No	1		Homozygous
NC_012920_rCRS H2a_haplo	143	SNV	G	A	No	1		Heterozygous
NC_012920_rCRS H2a_haplo	143	SNV	G	G	Yes	1		Heterozygous
NC_012920_rCRS H2a_haplo	146	SNV	T	C	No	1		Heterozygous
NC_012920_rCRS H2a_haplo	146	SNV	T	T	Yes	1		Heterozygous
NC_012920_rCRS H2a_haplo	150	SNV	C	T	No	1		Heterozygous
NC_012920_rCRS H2a_haplo	150	SNV	C	C	Yes	1		Heterozygous
NC_012920_rCRS H2a_haplo	152	SNV	T	C	No	1		Heterozygous
NC_012920_rCRS H2a_haplo	152	SNV	T	T	Yes	1		Heterozygous
NC_012920_rCRS H2a_haplo	189	SNV	A	G	No	1		Heterozygous
NC_012920_rCRS H2a_haplo	189	SNV	A	A	Yes	1		Heterozygous
NC_012920_rCRS H2a_haplo	200	SNV	A	G	No	1		Heterozygous
NC_012920_rCRS H2a_haplo	200	SNV	A	A	Yes	1		Heterozygous
NC_012920_rCRS H2a_haplo	248	Deletion	A	-	No	1		Heterozygous
NC_012920_rCRS H2a_haplo	248	SNV	A	A	Yes	1		Heterozygous
NC_012920_rCRS H2a_haplo	263	SNV	A	G	No	1		Homozygous
NC_012920_rCRS H2a_haplo	286..287	Deletion	AA	-	No	2		Heterozygous
NC_012920_rCRS H2a_haplo	286..287	MNV	AA	AA	Yes	2		Heterozygous
NC_012920_rCRS H2a_haplo	302^303	Insertion	-	C	No	1		Heterozygous
NC_012920_rCRS H2a_haplo	302^303	Insertion	-	CC	No	2		Heterozygous
NC_012920_rCRS H2a_haplo	302^303	Insertion	-	CCC	No	3		Heterozygous
NC_012920_rCRS H2a_haplo	302^303	Insertion	-	-	Yes	0		Heterozygous
NC_012920_rCRS H2a_haplo	308..310	Deletion	CCT	-	No	3		Heterozygous
NC_012920_rCRS H2a_haplo	308..310	MNV	CCT	CCT	Yes	3		Heterozygous
NC_012920_rCRS H2a_haplo	309..310	Deletion	CT	-	No	2		Heterozygous
NC_012920_rCRS H2a_haplo	310	SNV	T	C	No	1		Heterozygous
NC_012920_rCRS H2a_haplo	310^311	Insertion	-	C	No	1		Heterozygous
NC_012920_rCRS H2a_haplo	310^311	Insertion	-	-	Yes	0		Heterozygous
NC_012920_rCRS H2a_haplo	351	SNV	A	G	No	1		Heterozygous
NC_012920_rCRS H2a_haplo	351	SNV	A	A	Yes	1		Heterozygous
NC_012920_rCRS H2a_haplo	16036	SNV	G	T	No	1		Heterozygous
NC_012920_rCRS H2a_haplo	16036	SNV	G	G	Yes	1		Heterozygous
NC_012920_rCRS H2a_haplo	16086	SNV	T	C	No	1		Heterozygous
NC_012920_rCRS H2a_haplo	16086	SNV	T	T	Yes	1		Heterozygous
NC_012920_rCRS H2a_haplo	16149	SNV	A	C	No	1		Heterozygous
NC_012920_rCRS H2a_haplo	16149	SNV	A	A	Yes	1		Heterozygous
NC_012920_rCRS H2a_haplo	16172	SNV	T	A	No	1		Heterozygous
NC_012920_rCRS H2a_haplo	16172	SNV	T	T	Yes	1		Heterozygous
NC_012920_rCRS H2a_haplo	16176	SNV	C	T	No	1		Heterozygous
NC_012920_rCRS H2a_haplo	16176	SNV	C	C	Yes	1		Heterozygous
NC_012920_rCRS H2a_haplo	16180	SNV	A	C	No	1		Heterozygous
NC_012920_rCRS H2a_haplo	16180	Deletion	A	-	No	1		Heterozygous
NC_012920_rCRS H2a_haplo	16180	SNV	A	A	Yes	1		Heterozygous
NC_012920_rCRS H2a_haplo	16183	SNV	A	C	No	1		Heterozygous
NC_012920_rCRS H2a_haplo	16183	Replacement	A	CC	No	2		Heterozygous
NC_012920_rCRS H2a_haplo	16183	Replacement	A	CCC	No	3		Heterozygous
NC_012920_rCRS H2a_haplo	16183	SNV	A	A	Yes	1		Heterozygous
NC_012920_rCRS H2a_haplo	6183^16184	Insertion	-	C	No	1		Heterozygous
NC_012920_rCRS H2a_haplo	6183^16184	Insertion	-	-	Yes	0		Heterozygous
NC_012920_rCRS H2a_haplo	16189	SNV	T	C	No	1		Heterozygous
NC_012920_rCRS H2a_haplo	16189	SNV	T	T	Yes	1		Heterozygous
NC_012920_rCRS H2a_haplo	16196	SNV	G	C	No	1		Heterozygous

NC_012920_rCRS H2a_haplo	16196	SNV	G	G	Yes	1		Heterozygous
NC_012920_rCRS H2a_haplo	16223	SNV	C	T	No	1		Homozygous
NC_012920_rCRS H2a_haplo	16244	SNV	G	C	No	1		Heterozygous
NC_012920_rCRS H2a_haplo	16244	SNV	G	G	Yes	1		Heterozygous
NC_012920_rCRS H2a_haplo	16263	SNV	T	A	No	1		Heterozygous
NC_012920_rCRS H2a_haplo	16263	SNV	T	T	Yes	1		Heterozygous
NC_012920_rCRS H2a_haplo	16278	SNV	C	T	No	1		Homozygous
NC_012920_rCRS H2a_haplo	16298	SNV	T	C	No	1		Homozygous
NC_012920_rCRS H2a_haplo	16325	SNV	T	C	No	1		Homozygous
NC_012920_rCRS H2a_haplo	16327	SNV	C	T	No	1		Homozygous

**Count**	**Coverage**	**Frequency**	Probability	Forward read count	Reverse read count	Forward read coverage	Reverse read coverage	Forward/reverse balance	Average quality
608	610	99.67213115	1	608	0	610	0	0	63.40789474
586	727	80.60522696	1	586	0	727	0	0	36.58532423
137	727	18.84456671	1	137	0	727	0	0	31.80291971
46	727	6.327372765	1	46	0	727	0	0	31.73913043
680	727	93.53507565	1	680	0	727	0	0	37.21029412
61	726	8.402203857	1	61	0	726	0	0	36.81967213
665	726	91.59779614	1	665	0	726	0	0	38.1593985
55	726	7.575757576	1	55	0	726	0	0	35.96363636
670	726	92.28650138	1	670	0	726	0	0	38
3	119	2.521008403	0.799443837	3	0	119	0	0	28.66666667
116	119	97.4789916	1	116	0	119	0	0	36.76724138
2	118	1.694915254	0.999999987	2	0	118	0	0	37.5
116	118	98.30508475	1	116	0	118	0	0	38.62068966
116	118	98.30508475	1	116	0	118	0	0	61.60344828
2	118	1.694915254	1	2	0	118	0	0	64
118	118	100	1	118	0	118	0	0	60.43220339
115	118	97.45762712	1	115	0	118	0	0	62.53043478
3	118	2.542372881	1	3	0	118	0	0	62.16666667
88	118	74.57627119	1	88	0	118	0	0	34.95454545
7	118	5.93220339	1	7	0	118	0	0	33.35714286
2	118	1.694915254	1	2	0	118	0	0	27.66666667
21	118	17.79661017	1	21	0	118	0	0	28.19047619
4	118	3.389830508	1	4	0	118	0	0	37
106	118	89.83050847	1	106	0	118	0	0	31.71069182
4	118	3.389830508	1	4	0	118	0	0	38
3	118	2.542372881	0.999926764	3	0	118	0	0	38
104	118	88.13559322	1	104	0	118	0	0	37.29807692
14	118	11.86440678	1	14	0	118	0	0	34.78571429
3	121	2.479338843	0.999649743	3	0	121	0	0	38
118	121	97.52066116	1	118	0	121	0	0	36.03389831
2	64	3.125	0.999986801	2	0	64	0	0	27
62	64	96.875	1	62	0	64	0	0	36.56451613
59	64	92.1875	1	59	0	64	0	0	61.57627119
5	64	7.8125	1	5	0	64	0	0	53.6
19	64	29.6875	0.998966886	19	0	64	0	0	13.57894737
45	64	70.3125	1	45	0	64	0	0	23.11111111
5	68	7.352941176	0.750928218	5	0	68	0	0	15
60	68	88.23529412	1	60	0	68	0	0	21.61666667
2	96	2.083333333	0.999824306	2	0	96	0	0	35.5
94	96	97.91666667	1	94	0	96	0	0	29.91489362
2	97	2.06185567	0.999857887	2	0	97	0	0	24.5
13	97	13.40206186	1	13	0	97	0	0	36
82	97	84.53608247	1	82	0	97	0	0	32.02439024
46	98	46.93877551	1	46	0	98	0	0	30.19565217
23	98	23.46938776	1	23	0	98	0	0	31.01449275
5	98	5.102040816	1	5	0	98	0	0	31.33333333
22	98	22.44897959	1	22	0	98	0	0	31.20754717
3	98	3.06122449	1	3	0	98	0	0	35
94	98	95.91836735	1	94	0	98	0	0	31.40425532
95	98	96.93877551	1	95	0	98	0	0	36.35789474
3	98	3.06122449	0.903439792	3	0	98	0	0	30.33333333
9	106	8.490566038	0.808564246	9	0	106	0	0	18.22222222

95	106	89.62264151	1	95	0	106	0	0	33.25263158
41	42	97.61904762	1	41	0	42	0	0	27.80487805
7	44	15.90909091	0.999699059	7	0	44	0	0	17.42857143
35	44	79.54545455	1	35	0	44	0	0	29.22857143
7	51	13.7254902	1	7	0	51	0	0	63.85714286
44	51	86.2745098	1	44	0	51	0	0	40.22727273
51	51	100	1	51	0	51	0	0	56.94117647
51	51	100	1	51	0	51	0	0	60.43137255
51	51	100	1	51	0	51	0	0	42.29411765
51	51	100	1	51	0	51	0	0	40.62745098

Read count	Read coverage	# unique start positions	# unique end positions	BaseQRankSum	Read position test probability	Read direction test probability	Homopolymer	Homopolymer length	QUAL
608	610	3	4		1	1	No	1	200
586	727	3	4	13.77	0	1	No	1	200
137	727	3	4		0	1	No	1	200
46	727	2	4	-9.68	0	1	No	1	200
680	727	5	4		0.03536428	1	No	1	200
61	726	3	3	-10.18	0	1	No	1	200
665	726	4	5		0.001494785	1	No	1	200
55	726	3	3	-7.14	0	1	No	1	200
670	726	4	5		0.046258377	1	No	1	200
3	119	1	1	-1.55	0.000129874	1	No	1	6.977639882
116	119	3	3		0.869475872	1	No	1	200
2	118	1	1	-0.9	1	1	No	1	78.72895202
116	118	2	2		1	1	No	1	200
116	118	2	2	-0.5	1	1	No	1	200
2	118	1	1		1	1	No	1	200
118	118	2	2		1	1	No	1	200
115	118	2	2	0.16	1	1	Yes	6	200
3	118	1	1		0.827149653	1	Yes	6	200
88	118	2	2	5.46	1	1	Yes	7	200
7	118	1	1	1.49	1	1	Yes	7	200
2	118	1	1	0.29	1	1	Yes	7	200
21	118	1	1		1	1	Yes	7	200
4	118	1	1	3.45	1	1	No	1	200
106	118	2	2		1	1	Yes	7	200
4	118	1	1	4.55	1	1	Yes	7	200
3	118	2	2	3.02	1	1	Yes	7	41.35274791
104	118	2	2	1.19	1	1	Yes	5	200
14	118	2	2		1	1	Yes	5	200
3	121	1	1	1.4	9.21485E-15	1	No	1	34.55613548
118	121	2	2		0.093776177	1	No	1	200
2	64	1	1	0	1	1	No	1	48.79455681
62	64	1	2		1	1	No	1	200
59	64	1	2	0.78	1	1	No	1	200
5	64	1	1		1	1	No	1	200
19	64	1	1	-5.83	1	1	No	1	29.85851711
45	64	1	2		1	1	No	1	200
5	68	1	1	-2.41	0.596299182	1	No	1	6.03675472
60	68	2	3		0.903915341	1	No	1	200
2	96	1	1	0.68	0.245338974	1	No	1	37.5524307
94	96	3	4		1	1	No	1	200
2	97	2	2	-0.92	0.660290992	1	No	1	38.47365887
13	97	2	2	0.38	0.494450514	1	Yes	4	200
82	97	4	4		0.995550408	1	Yes	4	200
46	98	4	3	0.01	0.242206647	1	Yes	5	200
23	98	3	2	0.07	0.767420044	1	Yes	5	200
5	98	1	1	-0.13	0.119407079	1	Yes	5	200
22	98	3	3		0.566571813	1	Yes	5	200
3	98	2	2	-0.12	0.984815565	1	Yes	5	200
94	98	5	3		1	1	Yes	5	200
95	98	5	4	1.89	1	1	No	1	200
3	98	1	1		0.229088567	1	No	1	10.15201806
9	106	2	2	-3.72	0.000696838	1	No	1	7.179769473

95	106	9	4		0.446815651	1	No		1	200
41	42	8	2		1	1	No		1	200
7	44	1	1	-2.97	0.583922972	1	No		1	35.21519073
35	44	10	2		0.849578379	1	No		1	200
7	51	1	1	3.23	1.65871E-05	1	No		1	200
44	51	10	2		0.106107073	1	No		1	200
51	51	11	2		1	1	No		1	200
51	51	11	2		1	1	No		1	200
51	51	11	2		1	1	No		1	200
51	51	11	2		1	1	No		1	200

Supplementary Table S2 C10_533v Variant Table

Reference mitogenome	Region	Type	Reference	Allele	Reference allele	Length	Linkage	Zygosity
NC_012920_rCRS H2a_haplo	66	Deletion	G	-	No	1		Heterozygous
NC_012920_rCRS H2a_haplo	66	SNV	G	G	Yes	1		Heterozygous
NC_012920_rCRS H2a_haplo	73	SNV	A	G	No	1		Homozygous
NC_012920_rCRS H2a_haplo	146	SNV	T	C	No	1		Heterozygous
NC_012920_rCRS H2a_haplo	146	SNV	T	T	Yes	1		Heterozygous
NC_012920_rCRS H2a_haplo	150	SNV	C	T	No	1		Heterozygous
NC_012920_rCRS H2a_haplo	150	SNV	C	C	Yes	1		Heterozygous
NC_012920_rCRS H2a_haplo	152	SNV	T	C	No	1		Heterozygous
NC_012920_rCRS H2a_haplo	152	SNV	T	T	Yes	1		Heterozygous
NC_012920_rCRS H2a_haplo	195	SNV	T	C	No	1		Heterozygous
NC_012920_rCRS H2a_haplo	195	SNV	T	T	Yes	1		Heterozygous
NC_012920_rCRS H2a_haplo	204	SNV	T	C	No	1		Heterozygous
NC_012920_rCRS H2a_haplo	204	SNV	T	T	Yes	1		Heterozygous
NC_012920_rCRS H2a_haplo	259	SNV	A	G	No	1		Heterozygous
NC_012920_rCRS H2a_haplo	259	SNV	A	A	Yes	1		Heterozygous
NC_012920_rCRS H2a_haplo	263	SNV	A	G	No	1		Homozygous
NC_012920_rCRS H2a_haplo	302	SNV	A	C	No	1		Heterozygous
NC_012920_rCRS H2a_haplo	302	SNV	A	A	Yes	1		Heterozygous
NC_012920_rCRS H2a_haplo	302^303	Insertion	-	C	No	1		Heterozygous
NC_012920_rCRS H2a_haplo	302^303	Insertion	-	CC	No	2		Heterozygous
NC_012920_rCRS H2a_haplo	302^303	Insertion	-	-	Yes	0		Heterozygous
NC_012920_rCRS H2a_haplo	310	SNV	T	C	No	1		Heterozygous
NC_012920_rCRS H2a_haplo	310	SNV	T	T	Yes	1		Heterozygous
NC_012920_rCRS H2a_haplo	310^311	Insertion	-	C	No	1		Heterozygous
NC_012920_rCRS H2a_haplo	310^311	Insertion	-	-	Yes	0		Heterozygous
NC_012920_rCRS H2a_haplo	16172	SNV	T	C	No	1		Heterozygous
NC_012920_rCRS H2a_haplo	16172	SNV	T	T	Yes	1		Heterozygous
NC_012920_rCRS H2a_haplo	16189	SNV	T	C	No	1		Heterozygous
NC_012920_rCRS H2a_haplo	16189	Deletion	T	-	No	1		Heterozygous
NC_012920_rCRS H2a_haplo	16189	SNV	T	T	Yes	1		Heterozygous
NC_012920_rCRS H2a_haplo	16192	SNV	C	T	No	1		Heterozygous
NC_012920_rCRS H2a_haplo	16192	SNV	C	C	Yes	1		Heterozygous
NC_012920_rCRS H2a_haplo	16192^16193	Insertion	-	T	No	1		Heterozygous
NC_012920_rCRS H2a_haplo	16192^16193	Insertion	-	-	Yes	0		Heterozygous
NC_012920_rCRS H2a_haplo	16223..16224	MNV	CT	TC	No	2		Homozygous
NC_012920_rCRS H2a_haplo	16263	SNV	T	A	No	1		Heterozygous
NC_012920_rCRS H2a_haplo	16263	SNV	T	T	Yes	1		Heterozygous
NC_012920_rCRS H2a_haplo	16274	SNV	G	A	No	1		Homozygous
NC_012920_rCRS H2a_haplo	16278	SNV	C	T	No	1		Homozygous
NC_012920_rCRS H2a_haplo	16294	SNV	C	T	No	1		Homozygous
NC_012920_rCRS H2a_haplo	16309	SNV	A	G	No	1		Homozygous
NC_012920_rCRS H2a_haplo	16361	SNV	G	A	No	1		Heterozygous
NC_012920_rCRS H2a_haplo	16361	SNV	G	G	Yes	1		Heterozygous
NC_012920_rCRS H2a_haplo	16390	SNV	G	A	No	1		Heterozygous
NC_012920_rCRS H2a_haplo	16390	SNV	G	G	Yes	1		Heterozygous
NC_012920_rCRS H2a_haplo	16519	SNV	T	C	No	1		Homozygous

**Count**	**Coverage**	**Frequency**	Probability	Forward read count	Reverse read count	Forward read coverage	Reverse read coverage	Forward/reverse balance	Average quality
8	730	1.095890411	1	8	0	730	0	0	62.625
722	730	98.90410959	1	722	0	730	0	0	63.81717452
726	730	99.45205479	1	726	0	730	0	0	62.75619835
796	957	83.17659352	1	796	0	957	0	0	35.57286432
159	957	16.61442006	1	159	0	957	0	0	36.11320755
160	957	16.71891327	1	160	0	957	0	0	35.98125
796	957	83.17659352	1	796	0	957	0	0	37.76633166
820	957	85.68443051	1	820	0	957	0	0	37.69146341
136	957	14.21107628	1	136	0	957	0	0	37.25735294
224	229	97.81659389	1	224	0	229	0	0	38.27232143
5	229	2.183406114	0.999824269	5	0	229	0	0	35
7	229	3.056768559	0.999999917	7	0	229	0	0	29.14285714
222	229	96.94323144	1	222	0	229	0	0	37.67117117
222	228	97.36842105	1	222	0	228	0	0	61.24774775
6	228	2.631578947	0.999999566	6	0	228	0	0	64
228	228	100	1	228	0	228	0	0	62.46491228
5	228	2.192982456	0.992619655	5	0	228	0	0	24.4
223	228	97.80701754	1	223	0	228	0	0	30.4573991
191	228	83.77192982	1	191	0	228	0	0	34.25131226
14	228	6.140350877	1	14	0	228	0	0	31.57140282
23	228	10.0877193	1	23	0	228	0	0	29.26086947
3	228	1.315789474	0.849872909	3	0	228	0	0	26.33333333
222	228	97.36842105	1	222	0	228	0	0	24.01238739
221	228	96.92982456	1	221	0	228	0	0	36.91402715
7	228	3.070175439	1	7	0	228	0	0	31.14285714
4	114	3.50877193	0.999990642	4	0	114	0	0	30.25
110	114	96.49122807	1	110	0	114	0	0	34.54545455
156	163	95.70552147	1	156	0	163	0	0	36.93589744
5	163	3.067484663	0.999999945	5	0	163	0	0	37.8
2	163	1.226993865	0.989289974	2	0	163	0	0	36.5
144	163	88.34355828	1	144	0	163	0	0	36.07057309
19	163	11.65644172	1	19	0	163	0	0	36.46496218
15	163	9.202453988	1	15	0	163	0	0	33.13445179
147	163	90.18404908	1	147	0	163	0	0	35.68694371
65	65	100	1	65	0	65	0	0	37.57692308
6	72	8.333333333	1	6	0	72	0	0	63.66666667
66	72	91.66666667	1	66	0	72	0	0	62.33333333
72	72	100	1	72	0	72	0	0	63.44444444
72	72	100	1	72	0	72	0	0	62.88888889
72	72	100	1	72	0	72	0	0	62.93055556
72	72	100	1	72	0	72	0	0	62.23611111
4	353	1.133144476	0.99999998	4	0	353	0	0	37
349	353	98.86685552	1	349	0	353	0	0	37.747851
281	354	79.37853107	1	281	0	354	0	0	37.75088968
73	354	20.62146893	1	73	0	354	0	0	34.67123288
282	284	99.29577465	1	282	0	284	0	0	37.72695035

Supplementary Table S2 C10_533v Variant Table

Read count	Read coverage	# unique start positions	# unique end positions	BaseQRankSum	Read position test probability	Read direction test probability	Homopolymer length	Homopolymer	QUAL
8	730	2	2	-0.5	1	1	6	Yes	200
722	730	2	3		1	1	6	Yes	200
726	730	2	3		1	1	1	No	200
796	957	3	6	5.74	1.55762E-11	1	1	No	200
159	957	3	4		0	1	1	No	200
160	957	3	3	-14.08	0	1	1	No	200
796	957	3	6		2.9543E-13	1	1	No	200
820	957	4	5	10.16	4.39326E-09	1	1	No	200
136	957	3	5		0	1	1	No	200
224	229	2	4	0.71	1	1	1	No	200
5	229	1	1		1	1	1	No	37.55151125
7	229	1	1	-0.98	0.865717998	1	1	No	70.78833949
222	229	2	4		1	1	1	No	200
222	228	2	3	-0.55	1	1	1	No	200
6	228	1	1		1	1	1	No	63.6251027
228	228	2	3		1	1	1	No	200
5	228	1	1	-1.07	1	1	1	No	21.31923342
223	228	2	3		1	1	7	Yes	200
191	228	2	2	3.96	1	1	7	Yes	200
14	228	1	1	-0.5	1	1	7	Yes	200
23	228	1	2		1	1	7	Yes	200
3	228	1	1	-1.93	1	1	1	No	8.2354093
222	228	3	3		1	1	7	Yes	200
221	228	3	3	2.64	1	1	5	Yes	200
7	228	1	2		1	1	5	Yes	200
4	114	2	2	-0.12	0.000864502	1	1	No	50.28812318
110	114	4	4		0.703398296	1	1	No	200
156	163	4	5	0.72	0.999995991	1	1	No	200
5	163	2	2	0.77	0.700304872	1	5	Yes	72.55707017
2	163	1	1		0.538544662	1	1	No	19.70209483
144	163	4	5	-0.69	0.997507254	1	1	No	200
19	163	3	3		0.797066093	1	3	Yes	200
15	163	3	3	-1.6	0.958016359	1	1	No	200
147	163	4	5		0.998546135	1	3	Yes	200
65	65	4	2		1	1	1	No	200
6	72	2	2	0.03	1.28723E-07	1	1	No	200
66	72	5	2		0.145862251	1	1	No	200
72	72	7	3		1	1	1	No	200
72	72	7	3		1	1	1	No	200
72	72	7	3		1	1	1	No	200
72	72	7	3		1	1	1	No	200
4	353	1	1	-2.28	0.622635346	1	1	No	77.05533775
349	353	9	5		1	1	1	No	200
281	354	3	2	9.36	1.15463E-14	1	1	No	200
73	354	8	4		0	1	1	No	200
282	284	4	2		1	1	1	No	200

Supplementary Table S2 C11_534v Variant Table

Reference mitogenome	Region	Type	Reference	Allele	Reference allele	Length	Linkage	Zygosity
NC_012920_rCRS H2a_haplo	66	Deletion	G	-	No	1		Heterozygous
NC_012920_rCRS H2a_haplo	66	SNV	G	G	Yes	1		Heterozygous
NC_012920_rCRS H2a_haplo	73	SNV	A	G	No	1		Homozygous
NC_012920_rCRS H2a_haplo	146	SNV	T	C	No	1		Heterozygous
NC_012920_rCRS H2a_haplo	146	SNV	T	T	Yes	1		Heterozygous
NC_012920_rCRS H2a_haplo	150	SNV	C	T	No	1		Heterozygous
NC_012920_rCRS H2a_haplo	150	SNV	C	C	Yes	1		Heterozygous
NC_012920_rCRS H2a_haplo	152	SNV	T	C	No	1		Heterozygous
NC_012920_rCRS H2a_haplo	152	SNV	T	T	Yes	1		Heterozygous
NC_012920_rCRS H2a_haplo	153	SNV	A	G	No	1		Heterozygous
NC_012920_rCRS H2a_haplo	153	SNV	A	A	Yes	1		Heterozygous
NC_012920_rCRS H2a_haplo	235	SNV	A	G	No	1		Homozygous
NC_012920_rCRS H2a_haplo	263	SNV	A	G	No	1		Homozygous
NC_012920_rCRS H2a_haplo	302^303	Insertion	-	C	No	1		Heterozygous
NC_012920_rCRS H2a_haplo	302^303	Insertion	-	CC	No	2		Heterozygous
NC_012920_rCRS H2a_haplo	302^303	Insertion	-	-	Yes	0		Heterozygous
NC_012920_rCRS H2a_haplo	310^311	Insertion	-	C	No	1		Heterozygous
NC_012920_rCRS H2a_haplo	310^311	Insertion	-	-	Yes	0		Heterozygous
NC_012920_rCRS H2a_haplo	16111	SNV	C	T	No	1		Heterozygous
NC_012920_rCRS H2a_haplo	16111	SNV	C	C	Yes	1		Heterozygous
NC_012920_rCRS H2a_haplo	16180	Deletion	A	-	No	1		Heterozygous
NC_012920_rCRS H2a_haplo	16180	SNV	A	A	Yes	1		Heterozygous
NC_012920_rCRS H2a_haplo	16223	SNV	C	T	No	1		Homozygous
NC_012920_rCRS H2a_haplo	16265..16266	MNV	AC	GT	No	2		Homozygous
NC_012920_rCRS H2a_haplo	16290	SNV	C	T	No	1		Homozygous
NC_012920_rCRS H2a_haplo	16311	SNV	T	C	No	1		Homozygous
NC_012920_rCRS H2a_haplo	16319	SNV	G	A	No	1		Homozygous
NC_012920_rCRS H2a_haplo	16362	SNV	T	C	No	1		Heterozygous
NC_012920_rCRS H2a_haplo	16362	SNV	T	T	Yes	1		Heterozygous
NC_012920_rCRS H2a_haplo	16412	SNV	G	T	No	1		Heterozygous
NC_012920_rCRS H2a_haplo	16412	SNV	G	G	Yes	1		Heterozygous
NC_012920_rCRS H2a_haplo	16519	SNV	T	C	No	1		Homozygous

Supplementary Table S2 C11_534v Variant Table

**Count**	**Coverage**	**Frequency**	Probability	Forward read count	Reverse read count	Forward read coverage	Reverse read coverage	Forward/reverse balance	Average quality
8	506	1.581027668	1	8	0	506	0	0	57.125
498	506	98.41897233	1	498	0	506	0	0	63.7248996
503	506	99.40711462	1	503	0	506	0	0	63.00994036
544	619	87.88368336	1	544	0	619	0	0	36.26286765
74	619	11.95476575	1	74	0	619	0	0	36.32432432
76	619	12.27786753	1	76	0	619	0	0	35.89473684
543	619	87.72213247	1	543	0	619	0	0	37.95395948
53	619	8.562197092	1	53	0	619	0	0	35.73584906
566	619	91.43780291	1	566	0	619	0	0	37.72438163
491	619	79.32148627	1	491	0	619	0	0	37.57637475
128	619	20.67851373	1	128	0	619	0	0	36.671875
114	115	99.13043478	1	114	0	115	0	0	62.63157895
115	115	100	1	115	0	115	0	0	62.64347826
101	115	87.82608696	1	101	0	115	0	0	35.07920792
10	115	8.695652174	1	10	0	115	0	0	33.9
4	115	3.47826087	0.889161759	4	0	115	0	0	24.25
112	115	97.39130435	1	112	0	115	0	0	37.08035714
2	115	1.739130435	0.999996265	2	0	115	0	0	36
110	113	97.34513274	1	110	0	113	0	0	63.44545455
3	113	2.654867257	0.999553726	3	0	113	0	0	64
2	167	1.19760479	0.999999983	2	0	167	0	0	38
165	167	98.80239521	1	165	0	167	0	0	37.04848485
54	54	100	1	54	0	54	0	0	37.11111111
54	54	100	1	54	0	54	0	0	61.7037037
54	54	100	1	54	0	54	0	0	61.92592593
54	54	100	1	54	0	54	0	0	37.44444444
53	54	98.14814815	1	53	0	54	0	0	34.67924528
54	243	22.22222222	1	54	0	243	0	0	35.7037037
189	243	77.77777778	1	189	0	243	0	0	37.37037037
2	191	1.047120419	0.757000682	2	0	191	0	0	39
189	191	98.95287958	1	189	0	191	0	0	37.5978836
191	191	100	1	191	0	191	0	0	38.13089005

Supplementary Table S2 C11_534v Variant Table

Read count	Read coverage	# unique start positions	# unique end positions	BaseQRankSum	Read position test probability	Read direction test probability	Homopolymer length	Homopolymer	QUAL
8	506	1	1	-1.15	1	1	6	Yes	200
498	506	3	2		1	1	6	Yes	200
503	506	3	2		1	1	1	No	200
544	619	5	3	4.82	8.51286E-05	1	1	No	200
74	619	3	2		0	1	1	No	200
76	619	3	2	-10.69	0	1	1	No	200
543	619	5	3		1.24492E-06	1	1	No	200
53	619	3	2	-7.19	0	1	1	No	200
566	619	4	3		0.003047568	1	1	No	200
491	619	3	2	10.2	0	1	1	No	200
128	619	3	2		0	1	1	No	200
114	115	2	1		1	1	1	No	200
115	115	2	1		1	1	1	No	200
101	115	2	1	4.53	1	1	7	Yes	200
10	115	2	1	3.71	1	1	7	Yes	200
4	115	1	1		1	1	7	Yes	9.553103743
112	115	3	2	1.17	1	1	5	Yes	200
2	115	1	1		1	1	5	Yes	54.27686139
110	113	2	4	-0.08	1	1	1	No	200
3	113	1	1		1	1	1	No	33.50398804
2	167	1	1	0.47	0.633293919	1	4	Yes	77.7469072
165	167	6	5		1	1	4	Yes	200
54	54	4	1		1	1	1	No	200
54	54	4	1		1	1	1	No	200
54	54	4	1		1	1	1	No	200
54	54	4	1		1	1	1	No	200
53	54	4	1		1	1	1	No	200
54	243	4	1	-0.35	0	1	1	No	200
189	243	2	1		2.68673E-11	1	1	No	200
2	191	1	1	2.43	0.920533775	1	1	No	6.143949453
189	191	3	1		1	1	1	No	200
191	191	3	1		1	1	1	No	200

Supplementary Table S2 C12_536v Variant Table

Reference mitogenome	Region	Type	Reference	Allele	Reference allele	Length	Linkage	Zygosity
NC_012920_rCRS H2a_haplo	66	Deletion	G	-	No	1		Heterozygous
NC_012920_rCRS H2a_haplo	66	SNV	G	G	Yes	1		Heterozygous
NC_012920_rCRS H2a_haplo	73	SNV	A	G	No	1		Homozygous
NC_012920_rCRS H2a_haplo	146	SNV	T	C	No	1		Heterozygous
NC_012920_rCRS H2a_haplo	146	SNV	T	T	Yes	1		Heterozygous
NC_012920_rCRS H2a_haplo	150	SNV	C	T	No	1		Heterozygous
NC_012920_rCRS H2a_haplo	150	SNV	C	C	Yes	1		Heterozygous
NC_012920_rCRS H2a_haplo	152	SNV	T	C	No	1		Heterozygous
NC_012920_rCRS H2a_haplo	152	SNV	T	T	Yes	1		Heterozygous
NC_012920_rCRS H2a_haplo	187	SNV	G	T	No	1		Heterozygous
NC_012920_rCRS H2a_haplo	187	SNV	G	G	Yes	1		Heterozygous
NC_012920_rCRS H2a_haplo	195	SNV	T	C	No	1		Homozygous
NC_012920_rCRS H2a_haplo	263	SNV	A	G	No	1		Homozygous
NC_012920_rCRS H2a_haplo	310	SNV	T	C	No	1		Heterozygous
NC_012920_rCRS H2a_haplo	310	SNV	T	T	Yes	1		Heterozygous
NC_012920_rCRS H2a_haplo	310^311	Insertion	-	C	No	1		Heterozygous
NC_012920_rCRS H2a_haplo	310^311	Insertion	-	-	Yes	0		Heterozygous
NC_012920_rCRS H2a_haplo	16093	SNV	T	C	No	1		Homozygous
NC_012920_rCRS H2a_haplo	16129	SNV	G	A	No	1		Heterozygous
NC_012920_rCRS H2a_haplo	16129	SNV	G	G	Yes	1		Heterozygous
NC_012920_rCRS H2a_haplo	16173	SNV	C	T	No	1		Heterozygous
NC_012920_rCRS H2a_haplo	16173	SNV	C	C	Yes	1		Heterozygous
NC_012920_rCRS H2a_haplo	16189	SNV	T	C	No	1		Heterozygous
NC_012920_rCRS H2a_haplo	16189	Deletion	T	-	No	1		Heterozygous
NC_012920_rCRS H2a_haplo	16192	SNV	C	T	No	1		Heterozygous
NC_012920_rCRS H2a_haplo	16192	SNV	C	C	Yes	1		Heterozygous
NC_012920_rCRS H2a_haplo	16192^16193	Insertion	-	T	No	1		Heterozygous
NC_012920_rCRS H2a_haplo	16192^16193	Insertion	-	-	Yes	0		Heterozygous
NC_012920_rCRS H2a_haplo	16361	SNV	G	A	No	1		Heterozygous
NC_012920_rCRS H2a_haplo	16361	SNV	G	G	Yes	1		Heterozygous
NC_012920_rCRS H2a_haplo	16390	SNV	G	A	No	1		Homozygous

Supplementary Table S2 C12_536v Variant Table

**Count**	**Coverage**	**Frequency**	Probability	Forward read count	Reverse read count	Forward read coverage	Reverse read coverage	Forward/reverse balance	Average quality
5	184	2.717391304	1	0	5	0	184	0	64
177	184	96.19565217	1	0	177	0	184	0	63.60451977
182	184	98.91304348	1	0	182	0	184	0	63.76373626
226	302	74.83443709	1	0	226	0	302	0	35.77433628
75	302	24.83443709	1	0	75	0	302	0	35.57333333
70	302	23.17880795	1	0	70	0	302	0	36.25714286
231	302	76.49006623	1	0	231	0	302	0	37.77489177
234	302	77.48344371	1	0	234	0	302	0	37.67094017
68	302	22.51655629	1	0	68	0	302	0	37.36764706
2	118	1.694915254	0.999888127	0	2	0	118	0	37
116	118	98.30508475	1	0	116	0	118	0	36.17241379
118	118	100	1	0	118	0	118	0	37.77966102
118	118	100	1	0	118	0	118	0	63.00847458
4	118	3.389830508	0.999971746	0	4	0	118	0	38
113	118	95.76271186	1	0	113	0	118	0	26.60619469
111	118	94.06779661	1	0	111	0	118	0	37.0990991
7	118	5.93220339	1	0	7	0	118	0	29.71428571
60	62	96.77419355	1	0	60	0	62	0	62.21666667
2	62	3.225806452	0.857425343	0	2	0	62	0	64
60	62	96.77419355	1	0	60	0	62	0	60.33333333
60	62	96.77419355	1	0	60	0	62	0	33.65
2	62	3.225806452	0.99969634	0	2	0	62	0	34
60	62	96.77419355	1	0	60	0	62	0	36.43333333
2	62	3.225806452	0.797098768	0	2	0	62	0	27.5
35	62	56.4516129	1	0	35	0	62	0	35.17142857
27	62	43.5483871	1	0	27	0	62	0	37.48148148
19	62	30.64516129	1	0	19	0	62	0	37.73684211
41	62	66.12903226	1	0	41	0	62	0	34.24390244
2	174	1.149425287	0.994444268	0	2	0	174	0	37.5
172	174	98.85057471	1	0	172	0	174	0	37.5
173	175	98.85714286	1	0	173	0	175	0	37.41040462

Read count	Read coverage	# unique start positions	# unique end positions	BaseQRankSum	Read position test probability	Read direction test probability	Homopolymer length	Homopolymer	QUAL
5	184	1	1	0.09	1	1	6	Yes	200
177	184	3	1		1	1	6	Yes	200
182	184	3	1	1.19	1	1	1	No	200
226	302	4	3	4.15	9.67099E-05	1	1	No	200
75	302	3	4		2.22045E-16	1	1	No	200
70	302	2	2	-8.99	0	1	1	No	200
231	302	4	3		0.000224007	1	1	No	200
234	302	4	3	7.18	0.001416658	1	1	No	200
68	302	3	3		4.55191E-15	1	1	No	200
2	118	1	1	-1.33	0.899322332	1	1	No	39.51275104
116	118	2	3		1	1	1	No	200
118	118	2	3		1	1	1	No	200
118	118	2	3		1	1	1	No	200
4	118	1	2	1.71	1	1	1	No	45.48916985
113	118	2	3		1	1	7	Yes	200
111	118	2	3	1.52	1	1	5	Yes	200
7	118	1	2		1	1	5	Yes	200
60	62	1	1		1	1	1	No	200
2	62	1	1	0.44	0.761527553	1	1	No	8.459576639
60	62	1	1		1	1	1	No	200
60	62	1	1	0.54	1	1	1	No	200
2	62	1	1		1	1	1	No	35.17612984
60	62	1	1		1	1	1	No	200
2	62	1	1		1	1	5	Yes	6.927153149
35	62	1	1	-2.32	1	1	1	No	200
27	62	1	1		1	1	3	Yes	200
19	62	1	1	2.29	1	1	1	No	200
41	62	1	1		1	1	3	Yes	200
2	174	1	2	-0.58	0.916797683	1	1	No	22.5525872
172	174	3	5		1	1	1	No	200
173	175	2	5		0.329922679	1	1	No	200

Supplementary Table S2 D01_537v Variant Table

Reference mitogenome	Region	Type	Reference	Allele	Reference allele	Length	Linkage	Zygosity
NC_012920_rCRS H2a_haplo	146	SNV	T	C	No	1		Heterozygous
NC_012920_rCRS H2a_haplo	146	SNV	T	T	Yes	1		Heterozygous
NC_012920_rCRS H2a_haplo	150	SNV	C	T	No	1		Heterozygous
NC_012920_rCRS H2a_haplo	150	SNV	C	C	Yes	1		Heterozygous
NC_012920_rCRS H2a_haplo	152	SNV	T	C	No	1		Heterozygous
NC_012920_rCRS H2a_haplo	152	SNV	T	T	Yes	1		Heterozygous
NC_012920_rCRS H2a_haplo	263	SNV	A	G	No	1		Homozygous
NC_012920_rCRS H2a_haplo	302^303	Insertion	-	C	No	1		Heterozygous
NC_012920_rCRS H2a_haplo	302^303	Insertion	-	-	Yes	0		Heterozygous
NC_012920_rCRS H2a_haplo	310	SNV	T	C	No	1		Heterozygous
NC_012920_rCRS H2a_haplo	310	SNV	T	T	Yes	1		Heterozygous
NC_012920_rCRS H2a_haplo	310^311	Insertion	-	C	No	1		Heterozygous
NC_012920_rCRS H2a_haplo	310^311	Insertion	-	-	Yes	0		Heterozygous
NC_012920_rCRS H2a_haplo	16172	SNV	T	C	No	1		Heterozygous
NC_012920_rCRS H2a_haplo	16172	SNV	T	T	Yes	1		Heterozygous
NC_012920_rCRS H2a_haplo	16263	SNV	T	A	No	1		Heterozygous
NC_012920_rCRS H2a_haplo	16263	SNV	T	T	Yes	1		Heterozygous
NC_012920_rCRS H2a_haplo	16519	SNV	T	C	No	1		Homozygous

**Count**	**Coverage**	**Frequency**	Probability	Forward read count	Reverse read count	Forward read coverage	Reverse read coverage	Forward/reverse balance	Average quality
75	692	10.83815029	1	75	0	692	0	0	33.09333333
616	692	89.01734104	1	616	0	692	0	0	37.3961039
117	692	16.90751445	1	117	0	692	0	0	36.53846154
575	692	83.09248555	1	575	0	692	0	0	38.27652174
82	692	11.84971098	1	82	0	692	0	0	36.8902439
610	692	88.15028902	1	610	0	692	0	0	38
187	187	100	1	187	0	187	0	0	63.50802139
2	187	1.069518717	0.999999638	2	0	187	0	0	34
185	187	98.93048128	1	185	0	187	0	0	31.94054054
2	187	1.069518717	0.981489377	2	0	187	0	0	38
185	187	98.93048128	1	185	0	187	0	0	26.50810811
182	187	97.32620321	1	182	0	187	0	0	37.65384615
5	187	2.673796791	1	5	0	187	0	0	35.4
2	95	2.105263158	0.836264712	2	0	95	0	0	38
93	95	97.89473684	1	93	0	95	0	0	37.47311828
6	51	11.76470588	1	6	0	51	0	0	64
45	51	88.23529412	1	45	0	51	0	0	61.91111111
135	135	100	1	135	0	135	0	0	38.55555556

Supplementary Table S2 D01_537v Variant Table

Read count	Read coverage	# unique start positions	# unique end positions	BaseQRankSum	Read position test probability	Read direction test probability	Homopolymer	Homopolymer length	QUAL
75	692	2	2	-12.62	0	1	No	1	200
616	692	5	4		0.001288019	1	No	1	200
117	692	3	2	-12.96	0	1	No	1	200
575	692	4	4		3.57905E-09	1	No	1	200
82	692	3	3	-8.72	0	1	No	1	200
610	692	5	3		0.000343891	1	No	1	200
187	187	2	2		1	1	No	1	200
2	187	1	1	0.47	1	1	Yes	7	64.40691989
185	187	2	2		1	1	Yes	7	200
2	187	1	1	2.42	1	1	No	1	17.32578974
185	187	2	2		1	1	Yes	7	200
182	187	2	2	2.6	1	1	Yes	5	200
5	187	1	1		1	1	Yes	5	200
2	95	1	1	-0.67	0.000318757	1	No	1	7.858577117
93	95	2	3		0.756390546	1	No	1	200
6	51	2	1	0.53	5.00045E-06	1	No	1	200
45	51	4	2		0.072968356	1	No	1	200
135	135	4	1		1	1	No	1	200

Supplementary Table S2 D02_539v Variant Table

Reference mitogenome	Region	Type	Reference	Allele	Reference allele	Length	Linkage	Zygosity
NC_012920_rCRS H2a_haplo	146	SNV	T	C	No	1		Heterozygous
NC_012920_rCRS H2a_haplo	146	SNV	T	T	Yes	1		Heterozygous
NC_012920_rCRS H2a_haplo	150	SNV	C	T	No	1		Heterozygous
NC_012920_rCRS H2a_haplo	150	SNV	C	C	Yes	1		Heterozygous
NC_012920_rCRS H2a_haplo	152	SNV	T	C	No	1		Heterozygous
NC_012920_rCRS H2a_haplo	152	SNV	T	T	Yes	1		Heterozygous
NC_012920_rCRS H2a_haplo	263	SNV	A	G	No	1		Homozygous
NC_012920_rCRS H2a_haplo	285	SNV	C	A	No	1		Heterozygous
NC_012920_rCRS H2a_haplo	285	SNV	C	C	Yes	1		Heterozygous
NC_012920_rCRS H2a_haplo	302^303	Insertion	-	C	No	1		Heterozygous
NC_012920_rCRS H2a_haplo	302^303	Insertion	-	CC	No	2		Heterozygous
NC_012920_rCRS H2a_haplo	302^303	Insertion	-	-	Yes	0		Heterozygous
NC_012920_rCRS H2a_haplo	310^311	Insertion	-	C	No	1		Heterozygous
NC_012920_rCRS H2a_haplo	310^311	Insertion	-	-	Yes	0		Heterozygous
NC_012920_rCRS H2a_haplo	16162	SNV	A	G	No	1		Heterozygous
NC_012920_rCRS H2a_haplo	16162	SNV	A	A	Yes	1		Heterozygous
NC_012920_rCRS H2a_haplo	16242	SNV	C	T	No	1		Heterozygous
NC_012920_rCRS H2a_haplo	16242	SNV	C	C	Yes	1		Heterozygous
NC_012920_rCRS H2a_haplo	16263	SNV	T	A	No	1		Heterozygous
NC_012920_rCRS H2a_haplo	16263	SNV	T	T	Yes	1		Heterozygous
NC_012920_rCRS H2a_haplo	16298	SNV	T	C	No	1		Homozygous
NC_012920_rCRS H2a_haplo	16375	SNV	C	A	No	1		Heterozygous
NC_012920_rCRS H2a_haplo	16375	SNV	C	C	Yes	1		Heterozygous
NC_012920_rCRS H2a_haplo	16390	SNV	G	A	No	1		Heterozygous
NC_012920_rCRS H2a_haplo	16390	SNV	G	G	Yes	1		Heterozygous
NC_012920_rCRS H2a_haplo	16519	SNV	T	C	No	1		Heterozygous
NC_012920_rCRS H2a_haplo	16519	SNV	T	T	Yes	1		Heterozygous

Supplementary Table S2 D02_539v Variant Table

**Count**	**Coverage**	**Frequency**	Probability	Forward read count	Reverse read count	Forward read coverage	Reverse read coverage	Forward/reverse balance	Average quality
61	599	10.1836394	1	61	0	599	0	0	33.19672131
536	599	89.48247078	1	536	0	599	0	0	37.11940299
104	599	17.36227045	1	104	0	599	0	0	36.44230769
495	599	82.63772955	1	495	0	599	0	0	37.78181818
66	599	11.01836394	1	66	0	599	0	0	37.46969697
531	599	88.64774624	1	531	0	599	0	0	37.68549906
162	162	100	1	162	0	162	0	0	62.01234568
3	162	1.851851852	0.998093174	3	0	162	0	0	64
159	162	98.14814815	1	159	0	162	0	0	63.57232704
135	162	83.33333333	1	135	0	162	0	0	35.02222222
18	162	11.11111111	1	18	0	162	0	0	35.41666667
9	162	5.555555556	1	9	0	162	0	0	26.88888889
160	162	98.7654321	1	160	0	162	0	0	37.225
2	162	1.234567901	0.999943334	2	0	162	0	0	26
61	68	89.70588235	1	61	0	68	0	0	38.70491803
7	68	10.29411765	1	7	0	68	0	0	33.14285714
2	34	5.882352941	0.982409061	2	0	34	0	0	39
32	34	94.11764706	1	32	0	34	0	0	37.28125
3	37	8.108108108	0.999996857	3	0	37	0	0	64
34	37	91.89189189	1	34	0	37	0	0	62.11764706
37	37	100	1	37	0	37	0	0	63.32432432
2	171	1.169590643	0.932876468	2	0	171	0	0	38.5
169	171	98.83040936	1	169	0	171	0	0	38.0887574
5	171	2.923976608	1	5	0	171	0	0	33.4
166	171	97.07602339	1	166	0	171	0	0	36.27108434
2	136	1.470588235	0.999999997	2	0	136	0	0	39
134	136	98.52941176	1	134	0	136	0	0	38.49253731

Supplementary Table S2 D02_539v Variant Table

Read count	Read coverage	# unique start positions	# unique end positions	BaseQRankSum	Read position test probability	Read direction test probability	Homopolymer	Homopolymer length	QUAL
61	599	2	3	-11.08	0	1	No	1	200
536	599	3	5		0.008117131	1	No	1	200
104	599	2	3	-11.37	0	1	No	1	200
495	599	3	4		2.2268E-08	1	No	1	200
66	599	2	3	-6.23	0	1	No	1	200
531	599	3	5		0.002346184	1	No	1	200
162	162	1	2		1	1	No	1	200
3	162	1	1	0.13	0.894871048	1	No	1	27.19688958
159	162	1	2		1	1	No	1	200
135	162	1	2	5.29	1	1	Yes	7	200
18	162	1	1	4.81	1	1	Yes	7	200
9	162	1	1		1	1	Yes	7	200
160	162	1	2	1.04	1	1	Yes	5	200
2	162	1	1		1	1	Yes	5	42.46678209
61	68	1	2	4.09	0.013054617	1	No	1	200
7	68	1	2		1.04334E-08	1	No	1	200
2	34	2	1	0.44	0.814709927	1	No	1	17.54710982
32	34	7	4		0.988328566	1	No	1	200
3	37	1	1	0.17	0.000578665	1	No	1	55.02641801
34	37	7	4		0.476748223	1	No	1	200
37	37	8	4		1	1	No	1	200
2	171	2	1	0.01	0.774646102	1	No	1	11.73125197
169	171	11	6		1	1	No	1	200
5	171	2	1	-0.45	0.523195547	1	No	1	200
166	171	11	6		1	1	No	1	200
2	136	2	1	0.74	0.986177579	1	No	1	85.85026653
134	136	4	3		1	1	No	1	200

Supplementary Table S2 D03_542v Variant Table

Reference mitogenome	Region	Type	Reference	Allele	Reference allele	Length	Linkage	Zygosity
NC_012920_rCRS H2a_haplo	146	SNV	T	C	No	1		Heterozygous
NC_012920_rCRS H2a_haplo	146	SNV	T	T	Yes	1		Heterozygous
NC_012920_rCRS H2a_haplo	150	SNV	C	T	No	1		Heterozygous
NC_012920_rCRS H2a_haplo	150	SNV	C	C	Yes	1		Heterozygous
NC_012920_rCRS H2a_haplo	152	SNV	T	C	No	1		Heterozygous
NC_012920_rCRS H2a_haplo	152	SNV	T	T	Yes	1		Heterozygous
NC_012920_rCRS H2a_haplo	263	SNV	A	G	No	1		Homozygous
NC_012920_rCRS H2a_haplo	302^303	Insertion	-	C	No	1		Heterozygous
NC_012920_rCRS H2a_haplo	302^303	Insertion	-	CC	No	2		Heterozygous
NC_012920_rCRS H2a_haplo	302^303	Insertion	-	CCC	No	3		Heterozygous
NC_012920_rCRS H2a_haplo	302^303	Insertion	-	-	Yes	0		Heterozygous
NC_012920_rCRS H2a_haplo	310	SNV	T	C	No	1		Heterozygous
NC_012920_rCRS H2a_haplo	310	SNV	T	T	Yes	1		Heterozygous
NC_012920_rCRS H2a_haplo	310^311	Insertion	-	C	No	1		Heterozygous
NC_012920_rCRS H2a_haplo	310^311	Insertion	-	-	Yes	0		Heterozygous
NC_012920_rCRS H2a_haplo	351	SNV	A	G	No	1		Heterozygous
NC_012920_rCRS H2a_haplo	351	SNV	A	A	Yes	1		Heterozygous
NC_012920_rCRS H2a_haplo	514..515	Deletion	CA	-	No	2		Homozygous
NC_012920_rCRS H2a_haplo	16263	SNV	T	A	No	1		Heterozygous
NC_012920_rCRS H2a_haplo	16263	SNV	T	T	Yes	1		Heterozygous
NC_012920_rCRS H2a_haplo	16311	SNV	T	C	No	1		Homozygous
NC_012920_rCRS H2a_haplo	16313	SNV	C	A	No	1		Heterozygous
NC_012920_rCRS H2a_haplo	16313	SNV	C	C	Yes	1		Heterozygous
NC_012920_rCRS H2a_haplo	16519	SNV	T	C	No	1		Heterozygous
NC_012920_rCRS H2a_haplo	16519	SNV	T	T	Yes	1		Heterozygous

**Count**	**Coverage**	**Frequency**	Probability	Forward read count	Reverse read count	Forward read coverage	Reverse read coverage	Forward/reverse balance	Average quality
155	1488	10.41666667	1	155	0	1488	0	0	32.46451613
1330	1488	89.38172043	1	1330	0	1488	0	0	37.63909774
235	1490	15.77181208	1	235	0	1490	0	0	36.2893617
1254	1490	84.16107383	1	1254	0	1490	0	0	38.1539075
178	1491	11.93829645	1	178	0	1491	0	0	37.2247191
1313	1491	88.06170355	1	1313	0	1491	0	0	37.99619193
397	398	99.74874372	1	397	0	398	0	0	62.12846348
331	397	83.37531486	1	331	0	397	0	0	35.3836858
35	397	8.816120907	1	35	0	397	0	0	34.12857143
4	397	1.007556675	1	4	0	397	0	0	33.91666667
27	397	6.801007557	1	27	0	397	0	0	26.55555556
6	397	1.511335013	0.999990772	6	0	397	0	0	34.83333333
391	397	98.48866499	1	391	0	397	0	0	26.20716113
390	397	98.23677582	1	390	0	397	0	0	37.36153846
6	397	1.511335013	1	6	0	397	0	0	34.83333333
11	409	2.689486553	1	11	0	409	0	0	37.90909091
398	409	97.31051345	1	398	0	409	0	0	35.73869347
12	12	100	1	12	0	12	0	0	36.16666667
9	72	12.5	1	9	0	72	0	0	62.55555556
63	72	87.5	1	63	0	72	0	0	61.84126984
72	72	100	1	72	0	72	0	0	42.16666667
2	72	2.777777778	0.999946358	2	0	72	0	0	37
70	72	97.22222222	1	70	0	72	0	0	41.68571429
3	298	1.006711409	0.999456618	3	0	298	0	0	37.66666667
295	298	98.99328859	1	295	0	298	0	0	38.37966102

Supplementary Table S2 D03_542v Variant Table

Read count	Read coverage	# unique start positions	# unique end positions	BaseQRankSum	Read position test probability	Read direction test probability	Homopolymer	Homopolymer length	QUAL
155	1488	3	4	-18.6	0	1	No	1	200
1330	1488	6	6		5.84999E-06	1	No	1	200
235	1490	3	3	-18.82	0	1	No	1	200
1254	1490	6	7		1.9984E-15	1	No	1	200
178	1491	4	4	-12.08	0	1	No	1	200
1313	1491	5	6		2.42338E-07	1	No	1	200
397	398	3	3		1	1	No	1	200
331	397	3	2	8.42	1	1	Yes	7	200
35	397	1	1	5.09	1	1	Yes	7	200
4	397	1	1	2.06	1	1	Yes	7	200
27	397	3	1		1	1	Yes	7	200
6	397	2	1	2.69	1	1	No	1	50.3489712
391	397	4	3		1	1	Yes	7	200
390	397	4	3	2.37	1	1	Yes	5	200
6	397	2	1		1	1	Yes	5	200
11	409	1	1	2.05	0	1	No	1	99.99999964
398	409	4	3		0.003390935	1	No	1	200
12	12	1	1		1	1	No	1	200
9	72	1	1	0.18	1.14256E-07	1	No	1	200
63	72	7	3		0.045354362	1	No	1	200
72	72	8	3		1	1	No	1	200
2	72	2	1	-0.94	0.567507976	1	No	1	42.70495038
70	72	8	3		1	1	No	1	200
3	298	1	1	-1.48	1	1	No	1	32.64894832
295	298	5	1		1	1	No	1	200

Supplementary Table S2 D04_543v Variant Table

Reference mitogenome	Region	Type	Reference	Allele	Reference allele	Length	Linkage	Zygosity
NC_012920_rCRS H2a_haplo	73	SNV	A	G	No	1		Homozygous
NC_012920_rCRS H2a_haplo	146	SNV	T	C	No	1		Heterozygous
NC_012920_rCRS H2a_haplo	146	SNV	T	T	Yes	1		Heterozygous
NC_012920_rCRS H2a_haplo	150	SNV	C	T	No	1		Heterozygous
NC_012920_rCRS H2a_haplo	150	SNV	C	C	Yes	1		Heterozygous
NC_012920_rCRS H2a_haplo	152	SNV	T	C	No	1		Heterozygous
NC_012920_rCRS H2a_haplo	152	SNV	T	T	Yes	1		Heterozygous
NC_012920_rCRS H2a_haplo	200	SNV	A	G	No	1		Homozygous
NC_012920_rCRS H2a_haplo	263	SNV	A	G	No	1		Homozygous
NC_012920_rCRS H2a_haplo	295	SNV	C	T	No	1		Homozygous
NC_012920_rCRS H2a_haplo	302^303	Insertion	-	C	No	1		Heterozygous
NC_012920_rCRS H2a_haplo	302^303	Insertion	-	CC	No	2		Heterozygous
NC_012920_rCRS H2a_haplo	302^303	Insertion	-	-	Yes	0		Heterozygous
NC_012920_rCRS H2a_haplo	310^311	Insertion	-	C	No	1		Heterozygous
NC_012920_rCRS H2a_haplo	310^311	Insertion	-	-	Yes	0		Heterozygous
NC_012920_rCRS H2a_haplo	351	SNV	A	G	No	1		Heterozygous
NC_012920_rCRS H2a_haplo	351	SNV	A	A	Yes	1		Heterozygous
NC_012920_rCRS H2a_haplo	16069	SNV	C	T	No	1		Homozygous
NC_012920_rCRS H2a_haplo	16126	SNV	T	C	No	1		Homozygous
NC_012920_rCRS H2a_haplo	16145	SNV	G	A	No	1		Homozygous
NC_012920_rCRS H2a_haplo	16158	SNV	A	G	No	1		Homozygous
NC_012920_rCRS H2a_haplo	16162	Deletion	A	-	No	1		Heterozygous
NC_012920_rCRS H2a_haplo	16162	SNV	A	A	Yes	1		Heterozygous
NC_012920_rCRS H2a_haplo	16172	SNV	T	C	No	1		Heterozygous
NC_012920_rCRS H2a_haplo	16172	SNV	T	T	Yes	1		Heterozygous
NC_012920_rCRS H2a_haplo	16222	SNV	C	T	No	1		Homozygous
NC_012920_rCRS H2a_haplo	16261	SNV	C	T	No	1		Homozygous
NC_012920_rCRS H2a_haplo	16287	SNV	C	T	No	1		Homozygous

Supplementary Table S2 D04_543v Variant Table

**Count**	**Coverage**	**Frequency**	Probability	Forward read count	Reverse read count	Forward read coverage	Reverse read coverage	Forward/reverse balance	Average quality
629	632	99.52531646	1	629	0	632	0	0	63.44674086
125	954	13.10272537	1	125	0	954	0	0	32.92
825	954	86.47798742	1	825	0	954	0	0	37.36121212
193	954	20.23060797	1	193	0	954	0	0	36.61139896
759	954	79.55974843	1	759	0	954	0	0	38.06324111
124	954	12.99790356	1	124	0	954	0	0	37.58064516
830	954	87.00209644	1	830	0	954	0	0	37.8746988
321	323	99.38080495	1	321	0	323	0	0	37.81931464
323	323	100	1	323	0	323	0	0	62.37770898
321	323	99.38080495	1	321	0	323	0	0	33.06853583
280	323	86.6873065	1	280	0	323	0	0	35.49642857
20	323	6.191950464	1	20	0	323	0	0	34.7
21	323	6.501547988	1	21	0	323	0	0	27.57142857
316	323	97.83281734	1	316	0	323	0	0	37.55696203
7	323	2.167182663	1	7	0	323	0	0	28.42857143
4	326	1.226993865	0.786683055	4	0	326	0	0	32.75
322	326	98.77300613	1	322	0	326	0	0	36.53416149
137	137	100	1	137	0	137	0	0	38.16788321
137	137	100	1	137	0	137	0	0	63.27737226
136	136	100	1	136	0	136	0	0	37.50735294
136	136	100	1	136	0	136	0	0	37.97794118
2	165	1.212121212	0.906645865	2	0	165	0	0	37.5
163	165	98.78787879	1	163	0	165	0	0	36.47239264
9	168	5.357142857	1	9	0	168	0	0	37.55555556
158	168	94.04761905	1	158	0	168	0	0	37.48101266
119	120	99.16666667	1	119	0	120	0	0	38.05882353
120	120	100	1	120	0	120	0	0	62.95833333
120	120	100	1	120	0	120	0	0	63.55

Supplementary Table S2 D04_543v Variant Table

Read count	Read coverage	# unique start positions	# unique end positions	BaseQRankSum	Read position test probability	Read direction test probability	Homopolymer	Homopolymer length	QUAL
629	632	3	2		1	1	No	1	200
125	954	2	3	-16.32	0	1	No	1	200
825	954	6	4		0.000140751	1	No	1	200
193	954	2	2	-15.72	0	1	No	1	200
759	954	6	4		5.21361E-13	1	No	1	200
124	954	3	3	-9.99	0	1	No	1	200
830	954	5	4		0.000165277	1	No	1	200
321	323	3	2		1	1	No	1	200
323	323	3	2		1	1	No	1	200
321	323	3	2		1	1	No	1	200
280	323	3	2	8.96	1	1	Yes	7	200
20	323	1	1	5.68	1	1	Yes	7	200
21	323	1	1		1	1	Yes	7	200
316	323	3	2	3.51	1	1	Yes	5	200
7	323	1	1		1	1	Yes	5	99.99999964
4	326	2	2	0.14	0	1	No	1	6.709746445
322	326	3	2		0.091651128	1	No	1	200
137	137	3	4		1	1	No	1	200
137	137	3	4		1	1	No	1	200
136	136	3	3		1	1	No	1	200
136	136	3	3		1	1	No	1	200
2	165	1	1	-0.26	0.530567028	1	Yes	5	10.29866443
163	165	4	4		1	1	Yes	5	200
9	168	2	1	-1.68	3.48403E-08	1	No	1	200
158	168	5	4		0.397413051	1	No	1	200
119	120	4	2		0.329151295	1	No	1	200
120	120	5	2		1	1	No	1	200
120	120	5	2		1	1	No	1	200

Supplementary Table S2 D05_544v Variant Table

Reference mitogenome	Region	Type	Reference	Allele	Reference allele	Length	Linkage	Zygosity
NC_012920_rCRS H2a_haplo	73	SNV	A	G	No	1		Homozygous
NC_012920_rCRS H2a_haplo	146	SNV	T	C	No	1		Heterozygous
NC_012920_rCRS H2a_haplo	146	SNV	T	T	Yes	1		Heterozygous
NC_012920_rCRS H2a_haplo	150	SNV	C	T	No	1		Heterozygous
NC_012920_rCRS H2a_haplo	150	SNV	C	C	Yes	1		Heterozygous
NC_012920_rCRS H2a_haplo	152	SNV	T	C	No	1		Heterozygous
NC_012920_rCRS H2a_haplo	152	SNV	T	T	Yes	1		Heterozygous
NC_012920_rCRS H2a_haplo	185	SNV	G	A	No	1		Heterozygous
NC_012920_rCRS H2a_haplo	185	SNV	G	G	Yes	1		Heterozygous
NC_012920_rCRS H2a_haplo	228	SNV	G	A	No	1		Homozygous
NC_012920_rCRS H2a_haplo	263	SNV	A	G	No	1		Homozygous
NC_012920_rCRS H2a_haplo	295	SNV	C	T	No	1		Homozygous
NC_012920_rCRS H2a_haplo	302^303	Insertion	-	C	No	1		Heterozygous
NC_012920_rCRS H2a_haplo	302^303	Insertion	-	-	Yes	0		Heterozygous
NC_012920_rCRS H2a_haplo	310	SNV	T	C	No	1		Heterozygous
NC_012920_rCRS H2a_haplo	310	SNV	T	T	Yes	1		Heterozygous
NC_012920_rCRS H2a_haplo	310^311	Insertion	-	C	No	1		Heterozygous
NC_012920_rCRS H2a_haplo	310^311	Insertion	-	-	Yes	0		Heterozygous
NC_012920_rCRS H2a_haplo	351	SNV	A	G	No	1		Heterozygous
NC_012920_rCRS H2a_haplo	351	SNV	A	A	Yes	1		Heterozygous
NC_012920_rCRS H2a_haplo	462	SNV	C	T	No	1		Homozygous
NC_012920_rCRS H2a_haplo	489	SNV	T	C	No	1		Homozygous
NC_012920_rCRS H2a_haplo	16069	SNV	C	T	No	1		Homozygous
NC_012920_rCRS H2a_haplo	16126	SNV	T	C	No	1		Homozygous
NC_012920_rCRS H2a_haplo	16249	SNV	T	C	No	1		Heterozygous
NC_012920_rCRS H2a_haplo	16249	SNV	T	T	Yes	1		Heterozygous
NC_012920_rCRS H2a_haplo	16256	SNV	C	A	No	1		Heterozygous
NC_012920_rCRS H2a_haplo	16256	SNV	C	C	Yes	1		Heterozygous
NC_012920_rCRS H2a_haplo	16261	SNV	C	T	No	1		Homozygous

Supplementary Table S2 D05_544v Variant Table

**Count**	**Coverage**	**Frequency**	Probability	Forward read count	Reverse read count	Forward read coverage	Reverse read coverage	Forward/reverse balance	Average quality
1086	1089	99.72451791	1	1086	0	1089	0	0	63.46040516
410	2107	19.45894637	1	410	0	2107	0	0	32.8195122
1693	2107	80.35121025	1	1693	0	2107	0	0	37.01240402
622	2108	29.50664137	1	622	0	2108	0	0	36.36173633
1484	2108	70.39848197	1	1484	0	2108	0	0	37.71024259
449	2109	21.28971076	1	449	0	2109	0	0	37.27839644
1657	2109	78.56804173	1	1657	0	2109	0	0	37.57634279
1000	2104	47.52851711	1	1000	0	2104	0	0	35.818
1086	2104	51.61596958	1	1086	0	2104	0	0	32.26427256
1021	1023	99.80449658	1	1021	0	1023	0	0	37.48383937
1023	1023	100	1	1023	0	1023	0	0	63.13294233
992	1023	96.96969697	1	992	0	1023	0	0	31.04334677
28	1023	2.737047898	1	28	0	1023	0	0	34.60714286
995	1023	97.2629521	1	995	0	1023	0	0	30.61105528
17	1022	1.663405088	1	17	0	1022	0	0	34.52941176
994	1022	97.26027397	1	994	0	1022	0	0	26.02917505
982	1022	96.08610568	1	982	0	1022	0	0	37.07433809
40	1022	3.913894325	1	40	0	1022	0	0	31.7
14	1036	1.351351351	1	14	0	1036	0	0	36.78571429
1022	1036	98.64864865	1	1022	0	1036	0	0	35.71624266
14	14	100	1	14	0	14	0	0	60.35714286
14	14	100	1	14	0	14	0	0	36
552	553	99.81916817	1	552	0	553	0	0	38.4384058
553	553	100	1	553	0	553	0	0	63.72332731
2	109	1.834862385	0.815858014	2	0	109	0	0	38
107	109	98.16513761	1	107	0	109	0	0	38.91588785
2	109	1.834862385	0.773939102	2	0	109	0	0	64
107	109	98.16513761	1	107	0	109	0	0	63.60747664
109	109	100	1	109	0	109	0	0	62.40366972

Read count	Read coverage	# unique start positions	# unique end positions	BaseQRankSum	Read position test probability	Read direction test probability	Homopolymer	Homopolymer length	QUAL
1086	1089	4	3		1	1	No	1	200
410	2107	3	5	-27.78	0	1	No	1	200
1693	2107	7	7		8.03398E-11	1	No	1	200
622	2108	3	5	-22.6	0	1	No	1	200
1484	2108	7	5		0	1	No	1	200
449	2109	4	4	-13.03	0	1	No	1	200
1657	2109	6	7		2.34923E-13	1	No	1	200
1000	2104	3	4	28.31	0	1	No	1	200
1086	2104	5	3		0	1	No	1	200
1021	1023	3	4		1	1	No	1	200
1023	1023	3	4		1	1	No	1	200
992	1023	3	4	4.78	1	1	No	1	200
28	1023	1	1	3.54	1	1	Yes	7	200
995	1023	3	4		1	1	Yes	7	200
17	1022	1	1	2.74	1	1	No	1	200
994	1022	3	4		1	1	Yes	7	200
982	1022	3	4	4.79	1	1	Yes	5	200
40	1022	1	1		1	1	Yes	5	200
14	1036	1	1	0.26	0	1	No	1	200
1022	1036	3	4		0.000271098	1	No	1	200
14	14	1	1		1	1	No	1	200
14	14	1	1		1	1	No	1	200
552	553	2	6		1	1	No	1	200
553	553	2	6		1	1	No	1	200
2	109	2	1	-2.03	5.71264E-11	1	No	1	7.348471768
107	109	5	1		0.346435707	1	No	1	200
2	109	2	1	0.07	4.7951E-10	1	No	1	6.457745523
107	109	5	1		0.641956338	1	No	1	200
109	109	7	1		1	1	No	1	200

Supplementary Table S2 D06_546v Variant Table

Reference mitogenome	Region	Type	Reference	Allele	Reference allele	Length	Linkage	Zygosity
NC_012920_rCRS H2a_haplo	73	SNV	A	G	No	1		Homozygous
NC_012920_rCRS H2a_haplo	146	SNV	T	C	No	1		Heterozygous
NC_012920_rCRS H2a_haplo	146	SNV	T	T	Yes	1		Heterozygous
NC_012920_rCRS H2a_haplo	150	SNV	C	T	No	1		Heterozygous
NC_012920_rCRS H2a_haplo	150	SNV	C	C	Yes	1		Heterozygous
NC_012920_rCRS H2a_haplo	152	SNV	T	C	No	1		Heterozygous
NC_012920_rCRS H2a_haplo	152	SNV	T	T	Yes	1		Heterozygous
NC_012920_rCRS H2a_haplo	228	SNV	G	A	No	1		Heterozygous
NC_012920_rCRS H2a_haplo	228	SNV	G	G	Yes	1		Heterozygous
NC_012920_rCRS H2a_haplo	263	SNV	A	G	No	1		Homozygous
NC_012920_rCRS H2a_haplo	302^303	Insertion	-	C	No	1		Heterozygous
NC_012920_rCRS H2a_haplo	302^303	Insertion	-	-	Yes	0		Heterozygous
NC_012920_rCRS H2a_haplo	310	SNV	T	C	No	1		Heterozygous
NC_012920_rCRS H2a_haplo	310	SNV	T	T	Yes	1		Heterozygous
NC_012920_rCRS H2a_haplo	310^311	Insertion	-	C	No	1		Heterozygous
NC_012920_rCRS H2a_haplo	310^311	Insertion	-	-	Yes	0		Heterozygous
NC_012920_rCRS H2a_haplo	351	SNV	A	G	No	1		Heterozygous
NC_012920_rCRS H2a_haplo	351	SNV	A	A	Yes	1		Heterozygous
NC_012920_rCRS H2a_haplo	16172	SNV	T	C	No	1		Heterozygous
NC_012920_rCRS H2a_haplo	16172	SNV	T	T	Yes	1		Heterozygous
NC_012920_rCRS H2a_haplo	16239	SNV	C	T	No	1		Heterozygous
NC_012920_rCRS H2a_haplo	16239	SNV	C	C	Yes	1		Heterozygous
NC_012920_rCRS H2a_haplo	16249	SNV	T	C	No	1		Heterozygous
NC_012920_rCRS H2a_haplo	16249	SNV	T	T	Yes	1		Heterozygous
NC_012920_rCRS H2a_haplo	16256	SNV	C	T	No	1		Homozygous
NC_012920_rCRS H2a_haplo	16263	SNV	T	A	No	1		Heterozygous
NC_012920_rCRS H2a_haplo	16263	SNV	T	T	Yes	1		Heterozygous
NC_012920_rCRS H2a_haplo	16270	SNV	C	T	No	1		Homozygous
NC_012920_rCRS H2a_haplo	16399	SNV	A	G	No	1		Heterozygous
NC_012920_rCRS H2a_haplo	16399	SNV	A	A	Yes	1		Heterozygous

Supplementary Table S2 D06_546v Variant Table

**Count**	**Coverage**	**Frequency**	Probability	Forward read count	Reverse read count	Forward read coverage	Reverse read coverage	Forward/reverse balance	Average quality
1358	1361	99.77957384	1	1358	0	1361	0	0	63.45139912
249	1996	12.4749499	1	249	0	1996	0	0	33.00401606
1746	1996	87.4749499	1	1746	0	1996	0	0	37.44959908
405	1995	20.30075188	1	405	0	1995	0	0	36.40987654
1590	1995	79.69924812	1	1590	0	1995	0	0	38.17295597
247	1995	12.38095238	1	247	0	1995	0	0	36.97165992
1745	1995	87.46867168	1	1745	0	1995	0	0	37.80573066
22	636	3.459119497	1	22	0	636	0	0	38.18181818
612	636	96.22641509	1	612	0	636	0	0	38.04411765
636	636	100	1	636	0	636	0	0	62.98427673
13	636	2.044025157	1	13	0	636	0	0	35.46153846
623	636	97.95597484	1	623	0	636	0	0	31.11075441
11	636	1.729559748	0.999999999	11	0	636	0	0	35.90909091
625	636	98.27044025	1	625	0	636	0	0	27.2016
623	636	97.95597484	1	623	0	636	0	0	37.39325843
13	636	2.044025157	1	13	0	636	0	0	35.15384615
16	653	2.450229709	1	16	0	653	0	0	37.625
635	653	97.24349158	1	635	0	653	0	0	36.70708661
7	285	2.456140351	0.999964035	7	0	285	0	0	34.85714286
278	285	97.54385965	1	278	0	285	0	0	37.07553957
2	162	1.234567901	0.962911412	2	0	162	0	0	37.5
160	162	98.7654321	1	160	0	162	0	0	38.2625
2	163	1.226993865	0.9310204	2	0	163	0	0	37
161	163	98.77300613	1	161	0	163	0	0	39.24223602
161	163	98.77300613	1	161	0	163	0	0	62.61490683
9	172	5.23255814	1	9	0	172	0	0	64
163	172	94.76744186	1	163	0	172	0	0	63.1595092
171	172	99.41860465	1	171	0	172	0	0	63.53216374
394	564	69.85815603	1	394	0	564	0	0	36.84771574
170	564	30.14184397	1	170	0	564	0	0	31.90588235

Supplementary Table S2 D06_546v Variant Table

Read count	Read coverage	# unique start positions	# unique end positions	BaseQRankSum	Read position test probability	Read direction test probability	Homopolymer	Homopolymer length	QUAL
1358	1361	3	3		1	1	No	1	200
249	1996	2	2	-22.98	0	1	No	1	200
1746	1996	5	7		1.88387E-08	1	No	1	200
405	1995	3	5	-23.32	0	1	No	1	200
1590	1995	5	5		0	1	No	1	200
247	1995	2	4	-14.12	0	1	No	1	200
1745	1995	5	6		6.13509E-08	1	No	1	200
22	636	1	2	-0.65	1	1	No	1	200
612	636	2	4		1	1	No	1	200
636	636	2	4		1	1	No	1	200
13	636	1	2	3.15	1	1	Yes	7	200
623	636	2	4		1	1	Yes	7	200
11	636	1	1	4.38	1	1	No	1	88.86056649
625	636	2	4		1	1	Yes	7	200
623	636	2	4	2.71	1	1	Yes	5	200
13	636	1	1		1	1	Yes	5	200
16	653	2	2	0.97	0	1	No	1	200
635	653	3	4		0.000989552	1	No	1	200
7	285	3	3	-1.3	2.90067E-08	1	No	1	44.44116313
278	285	4	6		0.612374266	1	No	1	200
2	162	1	1	-1.96	0.851309741	1	No	1	14.30759702
160	162	7	3		1	1	No	1	200
2	163	2	1	-2.16	0.000102813	1	No	1	11.61279326
161	163	7	3		0.757530178	1	No	1	200
161	163	7	3		0.953461927	1	No	1	200
9	172	1	1	0.31	4.44089E-16	1	No	1	200
163	172	8	3		0.099849949	1	No	1	200
171	172	9	3		1	1	No	1	200
394	564	3	2	15.69	0	1	No	1	200
170	564	10	3		0	1	No	1	200

Supplementary Table S2 D07_548v Variant Table

Reference mitogenome	Region	Type	Reference	Allele	Reference allele	Length	Linkage	Zygosity
NC_012920_rCRS H2a_haplo	16	SNV	A	T	No	1		Homozygous
NC_012920_rCRS H2a_haplo	66	Deletion	G	-	No	1		Heterozygous
NC_012920_rCRS H2a_haplo	66	SNV	G	G	Yes	1		Heterozygous
NC_012920_rCRS H2a_haplo	73	SNV	A	G	No	1		Homozygous
NC_012920_rCRS H2a_haplo	146	SNV	T	C	No	1		Heterozygous
NC_012920_rCRS H2a_haplo	146	SNV	T	T	Yes	1		Heterozygous
NC_012920_rCRS H2a_haplo	150	SNV	C	T	No	1		Heterozygous
NC_012920_rCRS H2a_haplo	150	SNV	C	C	Yes	1		Heterozygous
NC_012920_rCRS H2a_haplo	152	SNV	T	C	No	1		Heterozygous
NC_012920_rCRS H2a_haplo	152	SNV	T	T	Yes	1		Heterozygous
NC_012920_rCRS H2a_haplo	199	SNV	T	C	No	1		Heterozygous
NC_012920_rCRS H2a_haplo	199	SNV	T	T	Yes	1		Heterozygous
NC_012920_rCRS H2a_haplo	263	SNV	A	G	No	1		Homozygous
NC_012920_rCRS H2a_haplo	302^303	Insertion	-	C	No	1		Heterozygous
NC_012920_rCRS H2a_haplo	302^303	Insertion	-	CC	No	2		Heterozygous
NC_012920_rCRS H2a_haplo	302^303	Insertion	-	-	Yes	0		Heterozygous
NC_012920_rCRS H2a_haplo	310	Replacement	T	CGC	No	3		Heterozygous
NC_012920_rCRS H2a_haplo	310	SNV	T	T	Yes	1		Heterozygous
NC_012920_rCRS H2a_haplo	310^311	Insertion	-	C	No	1		Heterozygous
NC_012920_rCRS H2a_haplo	310^311	Insertion	-	-	Yes	0		Heterozygous
NC_012920_rCRS H2a_haplo	351	SNV	A	G	No	1		Heterozygous
NC_012920_rCRS H2a_haplo	351	SNV	A	A	Yes	1		Heterozygous
NC_012920_rCRS H2a_haplo	16129	SNV	G	A	No	1		Homozygous
NC_012920_rCRS H2a_haplo	16172	SNV	T	C	No	1		Heterozygous
NC_012920_rCRS H2a_haplo	16172	SNV	T	T	Yes	1		Heterozygous
NC_012920_rCRS H2a_haplo	16224	SNV	T	C	No	1		Homozygous
NC_012920_rCRS H2a_haplo	16260	SNV	C	A	No	1		Heterozygous
NC_012920_rCRS H2a_haplo	16260	SNV	C	C	Yes	1		Heterozygous
NC_012920_rCRS H2a_haplo	16263	SNV	T	A	No	1		Heterozygous
NC_012920_rCRS H2a_haplo	16263	SNV	T	T	Yes	1		Heterozygous
NC_012920_rCRS H2a_haplo	16311	SNV	T	C	No	1		Homozygous
NC_012920_rCRS H2a_haplo	16396	SNV	T	G	No	1		Heterozygous
NC_012920_rCRS H2a_haplo	16396	SNV	T	T	Yes	1		Heterozygous
NC_012920_rCRS H2a_haplo	16519	SNV	T	C	No	1		Homozygous

Supplementary Table S2 D07_548v Variant Table

**Count**	**Coverage**	**Frequency**	Probability	Forward read count	Reverse read count	Forward read coverage	Reverse read coverage	Forward/reverse balance	Average quality
872	876	99.543379	1	872	0	876	0	0	37.89220183
20	876	2.283105023	1	20	0	876	0	0	63.35
856	876	97.71689498	1	856	0	876	0	0	63.79906542
872	876	99.543379	1	872	0	876	0	0	63.52866972
123	1186	10.37099494	1	123	0	1186	0	0	33.41463415
1060	1186	89.37605396	1	1060	0	1186	0	0	37.26603774
1059	1186	89.29173693	1	1059	0	1186	0	0	37.39754485
127	1186	10.70826307	1	127	0	1186	0	0	36.51181102
129	1186	10.87689713	1	129	0	1186	0	0	37.39534884
1057	1186	89.12310287	1	1057	0	1186	0	0	38.00473037
306	310	98.70967742	1	306	0	310	0	0	37.67973856
4	310	1.290322581	0.999999999	4	0	310	0	0	37.75
310	310	100	1	310	0	310	0	0	62.89677419
272	310	87.74193548	1	272	0	310	0	0	35.55514706
12	310	3.870967742	1	12	0	310	0	0	34.20833333
24	310	7.741935484	1	24	0	310	0	0	30.20833333
4	310	1.290322581	1	4	0	310	0	0	17.8125
301	310	97.09677419	1	301	0	310	0	0	26.77325581
299	310	96.4516129	1	299	0	310	0	0	37.42474916
11	310	3.548387097	1	11	0	310	0	0	26.90909091
6	316	1.898734177	0.999999677	6	0	316	0	0	38
310	316	98.10126582	1	310	0	316	0	0	36.18709677
158	159	99.37106918	1	158	0	159	0	0	63.93670886
5	174	2.873563218	0.999999987	5	0	174	0	0	37.6
168	174	96.55172414	1	168	0	174	0	0	36.91071429
81	82	98.7804878	1	81	0	82	0	0	36.95061728
2	92	2.173913043	0.981785682	2	0	92	0	0	64
90	92	97.82608696	1	90	0	92	0	0	63.65555556
8	92	8.695652174	1	8	0	92	0	0	64
84	92	91.30434783	1	84	0	92	0	0	62.76190476
92	92	100	1	92	0	92	0	0	41.52173913
11	327	3.363914373	0.999973259	11	0	327	0	0	19.45454545
316	327	96.63608563	1	316	0	327	0	0	36.12341772
234	235	99.57446809	1	234	0	235	0	0	37.93589744

Supplementary Table S2 D07_548v Variant Table

Read count	Read coverage	# unique start positions	# unique end positions	BaseQRankSum	Read position test probability	Read direction test probability	Homopolymer	Homopolymer length	QUAL
872	876	3	4		1	1	No	1	200
20	876	1	1	-0.27	1	1	Yes	6	200
856	876	3	4		1	1	Yes	6	200
872	876	3	4		1	1	No	1	200
123	1186	2	3	-15.72	0	1	No	1	200
1060	1186	5	7		5.48218E-05	1	No	1	200
1059	1186	5	8	10.74	4.50141E-05	1	No	1	200
127	1186	3	3		0	1	No	1	200
129	1186	3	2	-10.84	0	1	No	1	200
1057	1186	5	8		1.38544E-05	1	No	1	200
306	310	2	4	1.08	1	1	No	1	200
4	310	1	1		1	1	No	1	88.23908737
310	310	2	4		1	1	No	1	200
272	310	2	3	8.28	0.948701885	1	Yes	7	200
12	310	1	1	3.06	0.849250761	1	Yes	7	200
24	310	1	3		0.788345145	1	Yes	7	200
4	310	1	1	-3.53	1	1	No	1	200
301	310	2	4		1	1	Yes	7	200
299	310	2	4	3.78	1	1	Yes	5	200
11	310	1	1		1	1	Yes	5	200
6	316	1	1	1.5	0	1	No	1	64.91335637
310	316	2	4		0.059285642	1	No	1	200
158	159	1	2		1	1	No	1	200
5	174	1	1	-1.53	2.23087E-10	1	No	1	78.72895202
168	174	3	3		0.416540112	1	No	1	200
81	82	6	2		1	1	No	1	200
2	92	1	1	0.08	0.001047751	1	No	1	17.39587074
90	92	7	2		0.943851104	1	No	1	200
8	92	1	1	0.5	4.14251E-09	1	No	1	200
84	92	7	2		0.107850283	1	No	1	200
92	92	8	2		1	1	No	1	200
11	327	3	1	-5.05	4.01115E-06	1	No	1	45.72825604
316	327	10	4		0.941113377	1	No	1	200
234	235	4	1		1	1	No	1	200

Reference mitogenome	Region	Type	Reference	Allele	Reference allele	Length	Linkage	Zygosity
NC_012920_rCRS H2a_haplo	73	SNV	A	G	No	1		Homozygous
NC_012920_rCRS H2a_haplo	146	SNV	T	C	No	1		Heterozygous
NC_012920_rCRS H2a_haplo	146	SNV	T	T	Yes	1		Heterozygous
NC_012920_rCRS H2a_haplo	150	SNV	C	T	No	1		Heterozygous
NC_012920_rCRS H2a_haplo	150	SNV	C	C	Yes	1		Heterozygous
NC_012920_rCRS H2a_haplo	152	SNV	T	C	No	1		Heterozygous
NC_012920_rCRS H2a_haplo	152	SNV	T	T	Yes	1		Heterozygous
NC_012920_rCRS H2a_haplo	242	SNV	C	T	No	1		Homozygous
NC_012920_rCRS H2a_haplo	263	SNV	A	G	No	1		Homozygous
NC_012920_rCRS H2a_haplo	295	SNV	C	T	No	1		Homozygous
NC_012920_rCRS H2a_haplo	302^303	Insertion	-	C	No	1		Heterozygous
NC_012920_rCRS H2a_haplo	302^303	Insertion	-	-	Yes	0		Heterozygous
NC_012920_rCRS H2a_haplo	310	SNV	T	C	No	1		Heterozygous
NC_012920_rCRS H2a_haplo	310	SNV	T	T	Yes	1		Heterozygous
NC_012920_rCRS H2a_haplo	310^311	Insertion	-	C	No	1		Heterozygous
NC_012920_rCRS H2a_haplo	310^311	Insertion	-	-	Yes	0		Heterozygous
NC_012920_rCRS H2a_haplo	340	SNV	C	T	No	1		Homozygous
NC_012920_rCRS H2a_haplo	16069	SNV	C	T	No	1		Homozygous
NC_012920_rCRS H2a_haplo	16126	SNV	T	C	No	1		Homozygous
NC_012920_rCRS H2a_haplo	16145	SNV	G	A	No	1		Homozygous
NC_012920_rCRS H2a_haplo	16172	SNV	T	C	No	1		Heterozygous
NC_012920_rCRS H2a_haplo	16172	SNV	T	T	Yes	1		Heterozygous
NC_012920_rCRS H2a_haplo	16222	SNV	C	T	No	1		Homozygous
NC_012920_rCRS H2a_haplo	16241	SNV	A	T	No	1		Heterozygous
NC_012920_rCRS H2a_haplo	16241	SNV	A	A	Yes	1		Heterozygous
NC_012920_rCRS H2a_haplo	16261	SNV	C	T	No	1		Heterozygous
NC_012920_rCRS H2a_haplo	16261	SNV	C	C	Yes	1		Heterozygous

Supplementary Table S2 D08_549v Variant Table

**Count**	**Coverage**	**Frequency**	Probability	Forward read count	Reverse read count	Forward read coverage	Reverse read coverage	Forward/reverse balance	Average quality
1500	1501	99.93337775	1	1500	0	1501	0	0	63.40066667
1823	2391	76.24424927	1	1823	0	2391	0	0	35.94075699
563	2391	23.54663321	1	563	0	2391	0	0	36.15630551
508	2393	21.22858337	1	508	0	2393	0	0	36.48228346
1883	2393	78.68783953	1	1883	0	2393	0	0	37.9070632
377	2393	15.75428333	1	377	0	2393	0	0	36.81697613
2015	2393	84.20392812	1	2015	0	2393	0	0	37.64019851
891	893	99.77603583	1	891	0	893	0	0	62.48484848
893	893	100	1	893	0	893	0	0	63.06047032
865	893	96.86450168	1	865	0	893	0	0	31.79190751
15	893	1.679731243	1	15	0	893	0	0	33
878	893	98.32026876	1	878	0	893	0	0	31.30296128
14	893	1.56774916	1	14	0	893	0	0	35.04630894
873	893	97.76035834	1	873	0	893	0	0	27.51438615
866	893	96.97648376	1	866	0	893	0	0	37.47575058
27	893	3.023516237	1	27	0	893	0	0	29.96296296
885	893	99.10414334	1	885	0	893	0	0	37.32542373
421	421	100	1	421	0	421	0	0	37.9976247
420	421	99.76247031	1	420	0	421	0	0	63.76904762
420	421	99.76247031	1	420	0	421	0	0	37.42142857
426	462	92.20779221	1	426	0	462	0	0	37.27699531
35	462	7.575757576	1	35	0	462	0	0	36.2
193	195	98.97435897	1	193	0	195	0	0	37.79792746
2	197	1.015228426	0.99999972	2	0	197	0	0	38
195	197	98.98477157	1	195	0	197	0	0	38.41538462
195	198	98.48484848	1	195	0	198	0	0	61.6974359
3	198	1.515151515	0.991468112	3	0	198	0	0	64

Supplementary Table S2 D08_549v Variant Table

Read count	Read coverage	# unique start positions	# unique end positions	BaseQRankSum	Read position test probability	Read direction test probability	Homopolymer	Homopolymer length	QUAL
1500	1501	6	4		1	1	No	1	200
1823	2391	8	6	10.98	0	1	No	1	200
563	2391	4	6		0	1	No	1	200
508	2393	2	4	-24.14	0	1	No	1	200
1883	2393	9	7		0	1	No	1	200
377	2393	4	4	-14.84	0	1	No	1	200
2015	2393	8	8		1.94954E-11	1	No	1	200
891	893	3	5		1	1	No	1	200
893	893	3	5		1	1	No	1	200
865	893	3	4	5.69	1	1	No	1	200
15	893	1	1	0.64	1	1	Yes	7	200
878	893	3	5		1	1	Yes	7	200
14	893	1	2	2.63	1	1	No	1	200
873	893	3	5		1	1	Yes	7	200
866	893	3	4	4.59	1	1	Yes	5	200
27	893	2	3		1	1	Yes	5	200
885	893	3	4		0.329306924	1	No	1	200
421	421	3	2		1	1	No	1	200
420	421	3	2		1	1	No	1	200
420	421	3	2		1	1	No	1	200
426	462	4	3	0.87	2.26776E-06	1	No	1	200
35	462	2	2		0	1	No	1	200
193	195	5	3	0.94	1	1	No	1	200
2	197	2	2	-0.87	1.64281E-08	1	No	1	65.53307534
195	197	6	3		0.593424464	1	No	1	200
195	198	6	3	-0.58	0.928410921	1	No	1	200
3	198	3	1		0.000489128	1	No	1	20.68954859

Supplementary Table S2 D09_550v Variant Table

Reference mitogenome	Region	Type	Reference	Allele	Reference allele	Length	Linkage	Zygosity
NC_012920_rCRS H2a_haplo	146	SNV	T	C	No	1		Heterozygous
NC_012920_rCRS H2a_haplo	146	SNV	T	T	Yes	1		Heterozygous
NC_012920_rCRS H2a_haplo	150	SNV	C	T	No	1		Heterozygous
NC_012920_rCRS H2a_haplo	150	SNV	C	C	Yes	1		Heterozygous
NC_012920_rCRS H2a_haplo	152	SNV	T	C	No	1		Heterozygous
NC_012920_rCRS H2a_haplo	152	SNV	T	T	Yes	1		Heterozygous
NC_012920_rCRS H2a_haplo	263	SNV	A	G	No	1		Homozygous
NC_012920_rCRS H2a_haplo	302^303	Insertion	-	C	No	1		Heterozygous
NC_012920_rCRS H2a_haplo	302^303	Insertion	-	-	Yes	0		Heterozygous
NC_012920_rCRS H2a_haplo	309..310	MNV	CT	TC	No	2		Heterozygous
NC_012920_rCRS H2a_haplo	309..310	MNV	CT	CT	Yes	2		Heterozygous
NC_012920_rCRS H2a_haplo	310	SNV	T	C	No	1		Heterozygous
NC_012920_rCRS H2a_haplo	310^311	Insertion	-	C	No	1		Heterozygous
NC_012920_rCRS H2a_haplo	310^311	Insertion	-	-	Yes	0		Heterozygous
NC_012920_rCRS H2a_haplo	351	SNV	A	G	No	1		Heterozygous
NC_012920_rCRS H2a_haplo	351	SNV	A	A	Yes	1		Heterozygous
NC_012920_rCRS H2a_haplo	16263	SNV	T	A	No	1		Heterozygous
NC_012920_rCRS H2a_haplo	16263	SNV	T	T	Yes	1		Heterozygous
NC_012920_rCRS H2a_haplo	16278	SNV	C	T	No	1		Heterozygous
NC_012920_rCRS H2a_haplo	16278	SNV	C	C	Yes	1		Heterozygous

Supplementary Table S2 D09_550v Variant Table

**Count**	**Coverage**	**Frequency**	Probability	Forward read count	Reverse read count	Forward read coverage	Reverse read coverage	Forward/reverse balance	Average quality
140	1336	10.47904192	1	140	0	1336	0	0	32.95
1196	1336	89.52095808	1	1196	0	1336	0	0	37.34949833
266	1336	19.91017964	1	266	0	1336	0	0	36.58646617
1070	1336	80.08982036	1	1070	0	1336	0	0	37.96728972
211	1336	15.79341317	1	211	0	1336	0	0	37.22274882
1125	1336	84.20658683	1	1125	0	1336	0	0	37.76888889
448	448	100	1	448	0	448	0	0	63.03125
11	448	2.455357143	1	11	0	448	0	0	36.27272727
437	448	97.54464286	1	437	0	448	0	0	31.13501144
6	448	1.339285714	1	6	0	448	0	0	31.77777778
432	448	96.42857143	1	432	0	448	0	0	28.9058642
6	448	1.339285714	0.997559278	6	0	448	0	0	29.33333333
432	448	96.42857143	1	432	0	448	0	0	37.25462963
16	448	3.571428571	1	16	0	448	0	0	30.75
6	454	1.321585903	0.99997315	6	0	454	0	0	34.33333333
446	454	98.23788546	1	446	0	454	0	0	36.1793722
14	83	16.86746988	1	14	0	83	0	0	63.92857143
69	83	83.13253012	1	69	0	83	0	0	63.31884058
2	83	2.409638554	0.929567248	2	0	83	0	0	64
81	83	97.59036145	1	81	0	83	0	0	63.60493827

Supplementary Table S2 D09_550v Variant Table

Read count	Read coverage	# unique start positions	# unique end positions	BaseQRankSum	Read position test probability	Read direction test probability	Homopolymer	Homopolymer length	QUAL
140	1336	2	4	-17.32	0	1	No	1	200
1196	1336	5	7		0.001186219	1	No	1	200
266	1336	3	4	-17.91	0	1	No	1	200
1070	1336	5	7		1.11022E-16	1	No	1	200
211	1336	3	4	-11.44	0	1	No	1	200
1125	1336	5	6		8.53158E-09	1	No	1	200
448	448	2	4		1	1	No	1	200
11	448	1	1	3.8	1	1	Yes	7	200
437	448	2	4		1	1	Yes	7	200
6	448	1	2	2.41	1	1	No	1	200
432	448	2	4		1	1	Yes	7	200
6	448	1	1	-1.8	1	1	No	1	26.12481666
432	448	2	4	2.03	1	1	Yes	5	200
16	448	1	2		1	1	Yes	5	200
6	454	1	1	0.64	0	1	No	1	45.7106218
446	454	2	4		0.017367938	1	No	1	200
14	83	2	1	-0.15	3.82259E-09	1	No	1	200
69	83	5	2		0.002654185	1	No	1	200
2	83	2	1	0.06	0.543526567	1	No	1	11.5222534
81	83	6	2		1	1	No	1	200

Supplementary Table S2 D10_551v Variant Table

Reference mitogenome	Region	Type	Reference	Allele	Reference allele	Length	Linkage	Zygosity
NC_012920_rCRS H2a_haplo	146	SNV	T	C	No	1		Heterozygous
NC_012920_rCRS H2a_haplo	146	SNV	T	T	Yes	1		Heterozygous
NC_012920_rCRS H2a_haplo	150	SNV	C	T	No	1		Heterozygous
NC_012920_rCRS H2a_haplo	150	SNV	C	C	Yes	1		Heterozygous
NC_012920_rCRS H2a_haplo	152	SNV	T	C	No	1		Heterozygous
NC_012920_rCRS H2a_haplo	152	SNV	T	T	Yes	1		Heterozygous
NC_012920_rCRS H2a_haplo	195	SNV	T	C	No	1		Homozygous
NC_012920_rCRS H2a_haplo	204	SNV	T	C	No	1		Heterozygous
NC_012920_rCRS H2a_haplo	204	SNV	T	T	Yes	1		Heterozygous
NC_012920_rCRS H2a_haplo	263	SNV	A	G	No	1		Homozygous
NC_012920_rCRS H2a_haplo	297	SNV	A	C	No	1		Heterozygous
NC_012920_rCRS H2a_haplo	297	SNV	A	A	Yes	1		Heterozygous
NC_012920_rCRS H2a_haplo	302	Replacement	A	CCC	No	3		Heterozygous
NC_012920_rCRS H2a_haplo	302	SNV	A	A	Yes	1		Heterozygous
NC_012920_rCRS H2a_haplo	302^303	Insertion	-	C	No	1		Heterozygous
NC_012920_rCRS H2a_haplo	302^303	Insertion	-	CC	No	2		Heterozygous
NC_012920_rCRS H2a_haplo	302^303	Insertion	-	CCC	No	3		Heterozygous
NC_012920_rCRS H2a_haplo	302^303	Insertion	-	-	Yes	0		Heterozygous
NC_012920_rCRS H2a_haplo	310	SNV	T	C	No	1		Heterozygous
NC_012920_rCRS H2a_haplo	310	Replacement	T	CCGC	No	4		Heterozygous
NC_012920_rCRS H2a_haplo	310	SNV	T	T	Yes	1		Heterozygous
NC_012920_rCRS H2a_haplo	310^311	Insertion	-	C	No	1		Heterozygous
NC_012920_rCRS H2a_haplo	310^311	Insertion	-	-	Yes	0		Heterozygous
NC_012920_rCRS H2a_haplo	16172	SNV	T	C	No	1		Heterozygous
NC_012920_rCRS H2a_haplo	16172	SNV	T	T	Yes	1		Heterozygous
NC_012920_rCRS H2a_haplo	16263	SNV	T	A	No	1		Heterozygous
NC_012920_rCRS H2a_haplo	16263	SNV	T	T	Yes	1		Heterozygous
NC_012920_rCRS H2a_haplo	16293	SNV	A	G	No	1		Homozygous
NC_012920_rCRS H2a_haplo	16311	SNV	T	C	No	1		Homozygous
NC_012920_rCRS H2a_haplo	16396	SNV	T	G	No	1		Heterozygous
NC_012920_rCRS H2a_haplo	16396	SNV	T	T	Yes	1		Heterozygous

Supplementary Table S2 D10_551v Variant Table

**Count**	**Coverage**	**Frequency**	Probability	Forward read count	Reverse read count	Forward read coverage	Reverse read coverage	Forward/reverse balance	Average quality
130	1500	8.666666667	1	130	0	1500	0	0	32.93076923
1367	1500	91.13333333	1	1367	0	1500	0	0	37.13021214
201	1500	13.4	1	201	0	1500	0	0	36.12935323
1299	1500	86.6	1	1299	0	1500	0	0	37.82525019
142	1500	9.466666667	1	142	0	1500	0	0	36.95774648
1356	1500	90.4	1	1356	0	1500	0	0	37.58849558
327	330	99.09090909	1	327	0	330	0	0	38.28134557
4	330	1.212121212	0.849625887	4	0	330	0	0	22.75
325	330	98.48484848	1	325	0	330	0	0	37.82153846
329	330	99.6969697	1	329	0	330	0	0	59.2674772
20	330	6.060606061	0.729366554	20	0	330	0	0	13.95
303	330	91.81818182	1	303	0	330	0	0	25.0330033
9	330	2.727272727	1	9	0	330	0	0	25.61102591
318	330	96.36363636	1	318	0	330	0	0	27.04795631
28	330	8.484848485	1	28	0	330	0	0	35.03568937
241	330	73.03030303	1	241	0	330	0	0	33.96588159
34	330	10.3030303	1	34	0	330	0	0	30.47086797
27	330	8.181818182	1	27	0	330	0	0	24.44443919
5	330	1.515151515	0.999941163	5	0	330	0	0	33.4
5	330	1.515151515	0.999999998	5	0	330	0	0	23.27999696
315	330	95.45454545	1	315	0	330	0	0	24.12507937
314	330	95.15151515	1	314	0	330	0	0	36.1433121
16	330	4.848484848	1	16	0	330	0	0	28.25
3	219	1.369863014	0.999764978	3	0	219	0	0	37.33333333
215	219	98.17351598	1	215	0	219	0	0	36.75348837
10	143	6.993006993	1	10	0	143	0	0	61.2
133	143	93.00699301	1	133	0	143	0	0	59.90977444
143	143	100	1	143	0	143	0	0	62.72727273
143	143	100	1	143	0	143	0	0	41.34965035
27	493	5.476673428	1	27	0	493	0	0	21.44444444
465	493	94.32048682	1	465	0	493	0	0	36.43870968

Supplementary Table S2 D10_551v Variant Table

Read count	Read coverage	# unique start positions	# unique end positions	BaseQRankSum	Read position test probability	Read direction test probability	Homopolymer	Homopolymer length	QUAL
130	1500	3	2	-16.2	0	1	No	1	200
1367	1500	4	5		3.31167E-05	1	No	1	200
201	1500	2	4	-16.86	0	1	No	1	200
1299	1500	4	3		2.45359E-14	1	No	1	200
142	1500	3	2	-9.72	0	1	No	1	200
1356	1500	4	5		1.19435E-05	1	No	1	200
327	330	2	3	0.48	1	1	No	1	200
4	330	1	1	-2.26	1	1	No	1	8.228269222
325	330	2	3		1	1	No	1	200
329	330	2	3		1	1	No	1	200
20	330	1	1	-5.43	1	1	No	1	5.676185324
303	330	2	3		1	1	No	1	200
9	330	1	1	-0.75	1	1	No	1	200
318	330	2	3		1	1	Yes	7	200
28	330	1	1	8.55	1	1	Yes	7	200
241	330	2	3	9.77	1	1	Yes	7	200
34	330	2	1	5.27	1	1	Yes	7	200
27	330	1	1		1	1	Yes	7	200
5	330	1	1	-3.39	1	1	No	1	42.30348003
5	330	1	1	-0.76	1	1	No	1	86.57577327
315	330	2	3		1	1	Yes	7	200
314	330	2	3	3.75	1	1	Yes	5	200
16	330	1	1		1	1	Yes	5	200
3	219	2	1	-1.06	0.001054272	1	No	1	36.28891667
215	219	5	4		0.960785766	1	No	1	200
10	143	2	2	0.63	4.77396E-15	1	No	1	200
133	143	10	3		0.025646776	1	No	1	200
143	143	12	3		1	1	No	1	200
143	143	12	3		1	1	No	1	200
27	493	2	1	-7.8	5.54889E-13	1	No	1	93.97939973
465	493	16	6		0.610233427	1	No	1	200

Supplementary Table S2 D11_553v Variant Table

Reference mitogenome	Region	Type	Reference	Allele	Reference allele	Length	Linkage	Zygosity
NC_012920_rCRS H2a_haplo	73	SNV	A	G	No	1		Homozygous
NC_012920_rCRS H2a_haplo	114	SNV	C	T	No	1		Homozygous
NC_012920_rCRS H2a_haplo	146	SNV	T	C	No	1		Heterozygous
NC_012920_rCRS H2a_haplo	146	SNV	T	T	Yes	1		Heterozygous
NC_012920_rCRS H2a_haplo	150	SNV	C	T	No	1		Heterozygous
NC_012920_rCRS H2a_haplo	150	SNV	C	C	Yes	1		Heterozygous
NC_012920_rCRS H2a_haplo	152	SNV	T	C	No	1		Heterozygous
NC_012920_rCRS H2a_haplo	152	SNV	T	T	Yes	1		Heterozygous
NC_012920_rCRS H2a_haplo	263	SNV	A	G	No	1		Homozygous
NC_012920_rCRS H2a_haplo	302^303	Insertion	-	C	No	1		Heterozygous
NC_012920_rCRS H2a_haplo	302^303	Insertion	-	-	Yes	0		Heterozygous
NC_012920_rCRS H2a_haplo	310	SNV	T	C	No	1		Heterozygous
NC_012920_rCRS H2a_haplo	310	SNV	T	T	Yes	1		Heterozygous
NC_012920_rCRS H2a_haplo	310^311	Insertion	-	C	No	1		Heterozygous
NC_012920_rCRS H2a_haplo	310^311	Insertion	-	-	Yes	0		Heterozygous
NC_012920_rCRS H2a_haplo	351	SNV	A	G	No	1		Heterozygous
NC_012920_rCRS H2a_haplo	351	SNV	A	A	Yes	1		Heterozygous
NC_012920_rCRS H2a_haplo	16093	SNV	T	C	No	1		Heterozygous
NC_012920_rCRS H2a_haplo	16093	SNV	T	T	Yes	1		Heterozygous
NC_012920_rCRS H2a_haplo	16172	SNV	T	C	No	1		Heterozygous
NC_012920_rCRS H2a_haplo	16172	SNV	T	T	Yes	1		Heterozygous
NC_012920_rCRS H2a_haplo	16224	SNV	T	C	No	1		Homozygous
NC_012920_rCRS H2a_haplo	16263	SNV	T	A	No	1		Heterozygous
NC_012920_rCRS H2a_haplo	16263	SNV	T	T	Yes	1		Heterozygous
NC_012920_rCRS H2a_haplo	16311	SNV	T	C	No	1		Homozygous
NC_012920_rCRS H2a_haplo	16519	SNV	T	C	No	1		Homozygous

Supplementary Table S2 D11_553v Variant Table

**Count**	**Coverage**	**Frequency**	Probability	Forward read count	Reverse read count	Forward read coverage	Reverse read coverage	Forward/reverse balance	Average quality
1419	1427	99.43938332	1	1419	0	1427	0	0	63.44326991
1414	1427	99.0889979	1	1414	0	1427	0	0	38.04314003
215	2019	10.64883606	1	215	0	2019	0	0	32.94883721
1802	2019	89.252105	1	1802	0	2019	0	0	37.25027747
366	2020	18.11881188	1	366	0	2020	0	0	36.53825137
1653	2020	81.83168317	1	1653	0	2020	0	0	38.05747126
1689	2020	83.61386139	1	1689	0	2020	0	0	38.04026051
330	2020	16.33663366	1	330	0	2020	0	0	37.21818182
593	594	99.83164983	1	593	0	594	0	0	62.77571669
11	594	1.851851852	1	11	0	594	0	0	33.54545455
583	594	98.14814815	1	583	0	594	0	0	30.28987993
16	594	2.693602694	1	16	0	594	0	0	31.75
578	594	97.30639731	1	578	0	594	0	0	26.62629758
574	594	96.63299663	1	574	0	594	0	0	37.2804878
20	594	3.367003367	1	20	0	594	0	0	30.85
8	602	1.328903654	0.999999989	8	0	602	0	0	38
594	602	98.67109635	1	594	0	602	0	0	35.75589226
306	344	88.95348837	1	306	0	344	0	0	63.60784314
37	344	10.75581395	1	37	0	344	0	0	63.02702703
14	408	3.431372549	1	14	0	408	0	0	34.07142857
393	408	96.32352941	1	393	0	408	0	0	37.40966921
122	123	99.18699187	1	122	0	123	0	0	36.87704918
12	135	8.888888889	1	12	0	135	0	0	60.83333333
123	135	91.11111111	1	123	0	135	0	0	62.69105691
135	135	100	1	135	0	135	0	0	41.82962963
837	838	99.88066826	1	837	0	838	0	0	37.70250896

Supplementary Table S2 D11_553v Variant Table

Read count	Read coverage	# unique start positions	# unique end positions	BaseQRankSum	Read position test probability	Read direction test probability	Homopolymer	Homopolymer length	QUAL
1419	1427	4	3		1	1	No	1	200
1414	1427	4	3	-0.05	1	1	No	1	200
215	2019	3	3	-20.93	0	1	No	1	200
1802	2019	6	6		1.3041E-06	1	No	1	200
366	2020	2	2	-22.43	0	1	No	1	200
1653	2020	7	5		0	1	No	1	200
1689	2020	7	5	17.04	0	1	No	1	200
330	2020	3	4		0	1	No	1	200
593	594	3	3		1	1	No	1	200
11	594	1	1	1.49	0.894871048	1	Yes	7	200
583	594	3	3		1	1	Yes	7	200
16	594	2	2	2.36	1	1	No	1	200
578	594	4	3		1	1	Yes	7	200
574	594	4	3	5.38	1	1	Yes	5	200
20	594	2	2		1	1	Yes	5	200
8	602	1	1	2.11	0	1	No	1	79.39302161
594	602	3	3		0.022606584	1	No	1	200
306	344	2	4	0.79	0.938442785	1	No	1	200
37	344	1	2		0.65441573	1	No	1	200
14	408	2	3	-2.67	4.01855E-11	1	No	1	200
393	408	3	3		0.425343629	1	No	1	200
122	123	5	2		0.329100724	1	No	1	200
12	135	1	2	0.03	6.21725E-15	1	No	1	200
123	135	6	2		0.004857366	1	No	1	200
135	135	7	3		1	1	No	1	200
837	838	4	4		1	1	No	1	200

Supplementary Table S2 D12_554v Variant Table

Reference mitogenome	Region	Type	Reference	Allele	Reference allele	Length	Linkage	Zygosity
NC_012920_rCRS H2a_haplo	66	Deletion	G	-	No	1		Heterozygous
NC_012920_rCRS H2a_haplo	66	SNV	G	G	Yes	1		Heterozygous
NC_012920_rCRS H2a_haplo	73	SNV	A	G	No	1		Homozygous
NC_012920_rCRS H2a_haplo	146	SNV	T	C	No	1		Heterozygous
NC_012920_rCRS H2a_haplo	146	SNV	T	T	Yes	1		Heterozygous
NC_012920_rCRS H2a_haplo	150	SNV	C	T	No	1		Heterozygous
NC_012920_rCRS H2a_haplo	150	SNV	C	C	Yes	1		Heterozygous
NC_012920_rCRS H2a_haplo	152	SNV	T	C	No	1		Heterozygous
NC_012920_rCRS H2a_haplo	152	SNV	T	T	Yes	1		Heterozygous
NC_012920_rCRS H2a_haplo	263	SNV	A	G	No	1		Homozygous
NC_012920_rCRS H2a_haplo	302^303	Insertion	-	C	No	1		Heterozygous
NC_012920_rCRS H2a_haplo	302^303	Insertion	-	CC	No	2		Heterozygous
NC_012920_rCRS H2a_haplo	302^303	Insertion	-	-	Yes	0		Heterozygous
NC_012920_rCRS H2a_haplo	310	SNV	T	C	No	1		Heterozygous
NC_012920_rCRS H2a_haplo	310	SNV	T	T	Yes	1		Heterozygous
NC_012920_rCRS H2a_haplo	310^311	Insertion	-	C	No	1		Heterozygous
NC_012920_rCRS H2a_haplo	310^311	Insertion	-	-	Yes	0		Heterozygous
NC_012920_rCRS H2a_haplo	351	SNV	A	G	No	1		Heterozygous
NC_012920_rCRS H2a_haplo	351	SNV	A	A	Yes	1		Heterozygous
NC_012920_rCRS H2a_haplo	16169	SNV	C	T	No	1		Heterozygous
NC_012920_rCRS H2a_haplo	16169	SNV	C	C	Yes	1		Heterozygous
NC_012920_rCRS H2a_haplo	16192	SNV	C	A	No	1		Heterozygous
NC_012920_rCRS H2a_haplo	16192	SNV	C	C	Yes	1		Heterozygous
NC_012920_rCRS H2a_haplo	16224	SNV	T	C	No	1		Homozygous
NC_012920_rCRS H2a_haplo	16263	SNV	T	A	No	1		Heterozygous
NC_012920_rCRS H2a_haplo	16263	SNV	T	T	Yes	1		Heterozygous
NC_012920_rCRS H2a_haplo	16311	SNV	T	C	No	1		Homozygous
NC_012920_rCRS H2a_haplo	16396	SNV	T	G	No	1		Heterozygous
NC_012920_rCRS H2a_haplo	16396	SNV	T	T	Yes	1		Heterozygous
NC_012920_rCRS H2a_haplo	16438	SNV	G	A	No	1		Homozygous
NC_012920_rCRS H2a_haplo	16519	SNV	T	C	No	1		Homozygous

Supplementary Table S2 D12_554v Variant Table

**Count**	**Coverage**	**Frequency**	Probability	Forward read count	Reverse read count	Forward read coverage	Reverse read coverage	Forward/reverse balance	Average quality
13	1123	1.157613535	1	13	0	1123	0	0	61
1109	1123	98.75333927	1	1109	0	1123	0	0	63.82055906
1119	1123	99.64381122	1	1119	0	1123	0	0	63.49597855
97	1402	6.918687589	1	97	0	1402	0	0	33.17525773
1300	1402	92.72467903	1	1300	0	1402	0	0	37.43153846
157	1402	11.19828816	1	157	0	1402	0	0	36.36942675
1245	1402	88.80171184	1	1245	0	1402	0	0	38.1253012
114	1402	8.131241084	1	114	0	1402	0	0	36.68421053
1287	1402	91.79743224	1	1287	0	1402	0	0	37.90598291
279	279	100	1	279	0	279	0	0	63.07168459
248	278	89.20863309	1	248	0	278	0	0	35.47177419
19	278	6.834532374	1	19	0	278	0	0	34.5
10	278	3.597122302	1	10	0	278	0	0	30.5
3	278	1.079136691	0.99659901	3	0	278	0	0	29.33333333
275	278	98.92086331	1	275	0	278	0	0	26.84727273
273	278	98.20143885	1	273	0	278	0	0	37.53113553
5	278	1.798561151	0.999978439	5	0	278	0	0	26.6
7	285	2.456140351	1	7	0	285	0	0	34.57142857
277	285	97.19298246	1	277	0	285	0	0	36.1299639
171	207	82.60869565	1	171	0	207	0	0	38.22222222
36	207	17.39130435	1	36	0	207	0	0	32.5
3	215	1.395348837	0.939357603	3	0	215	0	0	30.33333333
212	215	98.60465116	1	212	0	215	0	0	36.66509434
43	43	100	1	43	0	43	0	0	37.20930233
2	45	4.444444444	0.998543724	2	0	45	0	0	64
43	45	95.55555556	1	43	0	45	0	0	63.3255814
45	45	100	1	45	0	45	0	0	40.22222222
8	401	1.995012469	0.999840433	8	0	401	0	0	27.375
393	401	98.00498753	1	393	0	401	0	0	37.38167939
356	358	99.44134078	1	356	0	358	0	0	63.29775281
357	358	99.72067039	1	357	0	358	0	0	38.05882353

Supplementary Table S2 D12_554v Variant Table

Read count	Read coverage	# unique start positions	# unique end positions	BaseQRankSum	Read position test probability	Read direction test probability	Homopolymer length	Homopolymer	QUAL
13	1123	1	2	-0.4	1	1	6	Yes	200
1109	1123	3	4		1	1	6	Yes	200
1119	1123	3	4		1	1	1	No	200
97	1402	3	3	-14.28	0	1	1	No	200
1300	1402	6	7		0.001400593	1	1	No	200
157	1402	3	3	-16.09	0	1	1	No	200
1245	1402	6	6		1.481E-10	1	1	No	200
114	1402	4	3	-10.08	0	1	1	No	200
1287	1402	5	6		0.0001054	1	1	No	200
279	279	3	3		1	1	1	No	200
248	278	3	2	4.5	1	1	7	Yes	200
19	278	1	1	3.13	1	1	7	Yes	200
10	278	2	1		1	1	7	Yes	200
3	278	1	1	1.15	1	1	1	No	24.68394594
275	278	4	3		1	1	7	Yes	200
273	278	4	3	2.14	1	1	5	Yes	200
5	278	1	1		1	1	5	Yes	46.66335129
7	285	1	1	-0.22	0	1	1	No	200
277	285	3	2		0.010605239	1	1	No	200
171	207	2	2	8.97	5.64611E-08	1	1	No	200
36	207	2	3		0	1	1	No	200
3	215	2	1	-1.36	0.00087443	1	1	No	12.1722364
212	215	4	4		0.933774185	1	1	No	200
43	43	3	2		1	1	1	No	200
2	45	1	1	0.17	1.52521E-05	1	1	No	28.36756188
43	45	3	2		0.399638073	1	1	No	200
45	45	4	2		1	1	1	No	200
8	401	2	1	-4.43	2.0185E-12	1	1	No	37.97057464
393	401	7	7		0.82964502	1	1	No	200
356	358	4	5		1	1	1	No	200
357	358	4	5		1	1	1	No	200

aThe variant table was auto-generated by the CLC Microbial Genomics workflow. Definitions of variant metrics are provided in the CLC online manual [25].
