# Supplementary material for: A Customized Human Mitochondrial DNA Database (hMITO DB v1.0) for Rapid Sequence Analysis, Haplotyping and Geo-Mapping
Source: Int J Mol Sci. 2023 Aug 31;24(17):13505. doi: 10.3390/ijms241713505 (PMC10488239; doi:10.3390/ijms241713505)
Supplement: Supplementary file 1 [file ijms-24-13505-s001.zip › ijms-2550861-supplementary/Table S3.pdf]

Supplementary Table S3 BLAST results and statistics[a] for human mtDNA haplotyping and geo-mapping

Query	Number of HSPs	Lowest E-value	Accession (E-value)	Description (E-value)
A01_HeLa	500	0	JQ705753.1	JQ705753.1; L3b1a; Africa_E; 38; A73G; A263G; C315CC
A03_Caski	500	0	KC911472_HV	KC911472_HV; HV; Asia_W; 11; A263G; C315CC; A750G
A04_SiHa	500	0	JQ703841.1	JQ703841.1; X2b+226; Asia_W_America_N; 32; A73G; T195C; A200G
A05_C33-A	500	0	JQ706015.1	JQ706015.1; U5a1b1a1; Asia_W_Europe_C; 29; A73G; A263G; C315CC
A06_Doc2C	500	0	JQ702663.1	JQ702663.1; U2e1b1; Asia_S_W_Europe; 38; A73G; T152C; T217C
A07_151v	500	0	JQ706012.1	JQ706012.1; H3i1; Asia_W_Europe; 12; T152C; A263G; C315CC
A08_152v	500	0	JQ705889.1	JQ705889.1; I1a1; Asia_W_SW; 42; A73G; T199C; G203A
A09_153v	500	0	JQ702841.1	JQ702841.1; H49a; Asia_W_Europe; 13; A263G; C309CCCT; T310C
A10_154v	500	0	JQ705798.1	JQ705798.1; V7; Europe_S; 18; T72C; A93G; T152C
A11_155v	500	0	JQ701820.1	JQ701820.1; C1b2; Asia_NE_America_N_S; 43; A73G; A249d; AA290d
A12_156v	500	0	JQ706012.1	JQ706012.1; H3i1; Asia_W_Europe; 12; T152C; A263G; C315CC
B01_500v	500	0	JQ706056.1	JQ706056.1; K1a31; Asia_W; 30; A73G; C150T; A263G
B02_503v	500	0	JQ706012.1	JQ706012.1; H3i1; Asia_W_Europe; 12; T152C; A263G; C315CC
B03_504v	500	0	JQ704786.1	JQ704786.1; T2j; Asia_W; 39; A73G; A249d; A263G
B04_505v	250	0	JQ705434.1	JQ705434.1; A2j; Asia_NE_America_N; 33; C64T; A73G; T146C
B05_507v	250	0	JQ706012.1	JQ706012.1; H3i1; Asia_W_Europe; 12; T152C; A263G; C315CC
B06_508v	500	0	JQ704350.1	JQ704350.1; A2+(64); Asia_NE_America_N; 35; C64T; A73G; T146C
B07_509v	500	0	JQ703481.1	JQ703481.1; L0a1a2; Africa_S_SE; 83; C64T; A93G; G185A
B08_511v	500	0	JQ703650.1	JQ703650.1; H13a1a1a; Asia_W_Europe; 19; T152C; A263G; C309CCT
B09_512v	500	0	JQ705099.1	JQ705099.1; V7a; Europe_S; 21; T72C; A93G; T195C
B10_513v	500	0	JQ705521.1	JQ705521.1; L3e1f2; Africa_E; 37; A73G; C150T; A189G
B11_515v	500	0	JQ704225.1	JQ704225.1; H5a1; Asia_W_Europe; 16; T195C; A240G; A263G
B12_516v	500	0	JQ704767.1	JQ704767.1; V+@72; Europe_S; 14; A263G; C309CCT; T310C
C01_519v	500	0	JQ702074.1	JQ702074.1; F1a3a; Asia_SE_E; 37; A73G; A249d; A263G
C02_520v	750	0	JQ703138.1	JQ703138.1; L3e2b1a; Africa_E; 33; A73G; C150T; T195C
C03_521v	749	0	JQ705902.1	JQ705902.1; L3e1a3a; Africa_E; 34; A73G; C150T; A200G
C04_523v	250	3.2383E-180	JQ704931.1	JQ704931.1; L3e3b1; Africa_E; 41; A73G; C150T; T195C
C05_524v	250	8.8262E-142	JQ706012.1	JQ706012.1; H3i1; Asia_W_Europe; 12; T152C; A263G; C315CC
C06_525v	500	0	JQ705272.1	JQ705272.1; U5a1h; Asia_W_Europe_C; 37; A73G; C150T; G207A
C07_528v	500	0	JQ705362.1	JQ705362.1; H1av; Asia_W_Europe; 14; A263G; C309CCCT; T310C
C08_529v	500	0	JQ705975.1	JQ705975.1; H66a; Asia_W_Europe; 11; A263G; C315CC; A750G
C09_532v	495	0	JQ705585.1	JQ705585.1; C1b4; Asia_NE_America_N_S; 47; A73G; G143A; T152C
C10_533v	500	0	JQ705150.1	JQ705150.1; L2a1f; Africa_W_C; 54; A73G; T146C; T152C
C11_534v	495	1.7504E-177	JQ705378.1	JQ705378.1; I1a1a3; Asia_W_SW; 49; A73G; A189G; T199C
C12_536v	996	0	JQ705455.1	JQ705455.1; L2a1e1; Africa_W_C; 56; A73G; T146C; T152C
D01_537v	500	0	KC911472_HV	KC911472_HV; HV; Asia_W; 11; A263G; C315CC; A750G
D02_539v	500	1.679E-158	KC911472_HV	KC911472_HV; HV; Asia_W; 11; A263G; C315CC; A750G
D03_542v	500	0	JQ706018.1	JQ706018.1; NA; NA; 2; C315CC; T16311C;
D04_543v	500	0	JQ703786.1	JQ703786.1; J1c3; Asia_W; 27; A73G; A263G; C295T
D05_544v	500	0	JQ705489.1	JQ705489.1; J1c+16261; Asia_W; 29; A73G; G185A; A263G
D06_546v	500	0	JQ705886.1	JQ705886.1; U5a1a2b1; Asia_W_Europe_C; 33; A73G; A263G; C309CCT
D07_548v	500	0	JQ702675.1	JQ702675.1; K1a11a; Asia_W; 39; A16T; A73G; C150T
D08_549v	500	0	JQ705964.1	JQ705964.1; J1b1a1c; Asia_W; 39; T10C; A73G; T146C
D09_550v	500	0	NC_012920_rCRS	NC_012920_rCRS;H2a2a1;Asia_W_Europe;0;REF;REF;REF
D10_551v	500	0	JQ705928.1	JQ705928.1; H11a; Asia_W_Europe; 16; T195C; A263G; C309CCT
D11_553v	500	0	JQ706065.1	JQ706065.1; K1a12a1a; Asia_W; 37; A73G; T195C; A263G
D12_554v	1000	0	JQ704761.1	JQ704761.1; K1a4e; Asia_W; 35; A73G; A263G; C309CCT

Supplementary Table S3 BLAST results and statistics[a] for human mtDNA haplotyping and geo-mapping

Query	Greatest identity %	Accession (identity %)	Description (identity %)
A01_HeLa	99.82758621	JQ705753.1	JQ705753.1; L3b1a; Africa_E; 38; A73G; A263G; C315CC
A03_Caski	100	KC911472_HV	KC911472_HV; HV; Asia_W; 11; A263G; C315CC; A750G
A04_SiHa	99.82758621	JQ703841.1	JQ703841.1; X2b+226; Asia_W_America_N; 32; A73G; T195C; A200G
A05_C33-A	99.82425308	JQ706015.1	JQ706015.1; U5a1b1a1; Asia_W_Europe_C; 29; A73G; A263G; C315CC
A06_Doc2C	100	JQ702663.1	JQ702663.1; U2e1b1; Asia_S_W_Europe; 38; A73G; T152C; T217C
A07_151v	100	JQ706012.1	JQ706012.1; H3i1; Asia_W_Europe; 12; T152C; A263G; C315CC
A08_152v	100	JQ705889.1	JQ705889.1; I1a1; Asia_W_SW; 42; A73G; T199C; G203A
A09_153v	100	EU007853_Y	EU007853_Y;Y1a+16189;Asia_NE;24;A73G;T146C;A263G
A10_154v	100	JQ705798.1	JQ705798.1; V7; Europe_S; 18; T72C; A93G; T152C
A11_155v	100	JQ701820.1	JQ701820.1; C1b2; Asia_NE_America_N_S; 43; A73G; A249d; AA290d
A12_156v	100	JQ706012.1	JQ706012.1; H3i1; Asia_W_Europe; 12; T152C; A263G; C315CC
B01_500v	100	JQ706056.1	JQ706056.1; K1a31; Asia_W; 30; A73G; C150T; A263G
B02_503v	100	JQ706012.1	JQ706012.1; H3i1; Asia_W_Europe; 12; T152C; A263G; C315CC
B03_504v	100	JQ705572.1	JQ705572.1; F1f; Asia_SE_E; 32; A73G; A249d; A263G
B04_505v	100	JQ705434.1	JQ705434.1; A2j; Asia_NE_America_N; 33; C64T; A73G; T146C
B05_507v	100	JQ706012.1	JQ706012.1; H3i1; Asia_W_Europe; 12; T152C; A263G; C315CC
B06_508v	100	JQ704350.1	JQ704350.1; A2+(64); Asia_NE_America_N; 35; C64T; A73G; T146C
B07_509v	100	JQ703481.1	JQ703481.1; L0a1a2; Africa_S_SE; 83; C64T; A93G; G185A
B08_511v	100	JQ703650.1	JQ703650.1; H13a1a1a; Asia_W_Europe; 19; T152C; A263G; C309CCT
B09_512v	100	JQ705099.1	JQ705099.1; V7a; Europe_S; 21; T72C; A93G; T195C
B10_513v	99.73890339	JQ704728.1	JQ704728.1; L3e1f; Africa_E; 39; A73G; C150T; A189G
B11_515v	100	JQ704225.1	JQ704225.1; H5a1; Asia_W_Europe; 16; T195C; A240G; A263G
B12_516v	100	JQ705599.1	JQ705599.1; HV0; Asia_W; 14; T72C; A263G; C309CCCT
C01_519v	100	JQ702074.1	JQ702074.1; F1a3a; Asia_SE_E; 37; A73G; A249d; A263G
C02_520v	100	JQ703138.1	JQ703138.1; L3e2b1a; Africa_E; 33; A73G; C150T; T195C
C03_521v	100	JQ705902.1	JQ705902.1; L3e1a3a; Africa_E; 34; A73G; C150T; A200G
C04_523v	100	JQ704931.1	JQ704931.1; L3e3b1; Africa_E; 41; A73G; C150T; T195C
C05_524v	100	JQ706012.1	JQ706012.1; H3i1; Asia_W_Europe; 12; T152C; A263G; C315CC
C06_525v	99.64912281	JQ705272.1	JQ705272.1; U5a1h; Asia_W_Europe_C; 37; A73G; C150T; G207A
C07_528v	100	JQ705362.1	JQ705362.1; H1av; Asia_W_Europe; 14; A263G; C309CCCT; T310C
C08_529v	99.48364888	JQ705975.1	JQ705975.1; H66a; Asia_W_Europe; 11; A263G; C315CC; A750G
C09_532v	100	JQ705585.1	JQ705585.1; C1b4; Asia_NE_America_N_S; 47; A73G; G143A; T152C
C10_533v	99.65517241	JQ705150.1	JQ705150.1; L2a1f; Africa_W_C; 54; A73G; T146C; T152C
C11_534v	100	JQ705378.1	JQ705378.1; I1a1a3; Asia_W_SW; 49; A73G; A189G; T199C
C12_536v	100	JQ705455.1	JQ705455.1; L2a1e1; Africa_W_C; 56; A73G; T146C; T152C
D01_537v	100	KC911472_HV	KC911472_HV; HV; Asia_W; 11; A263G; C315CC; A750G
D02_539v	100	KC911472_HV	KC911472_HV; HV; Asia_W; 11; A263G; C315CC; A750G
D03_542v	100	JQ706018.1	JQ706018.1; NA; NA; 2; C315CC; T16311C;
D04_543v	100	JQ703786.1	JQ703786.1; J1c3; Asia_W; 27; A73G; A263G; C295T
D05_544v	100	JQ705489.1	JQ705489.1; J1c+16261; Asia_W; 29; A73G; G185A; A263G
D06_546v	100	JQ705886.1	JQ705886.1; U5a1a2b1; Asia_W_Europe_C; 33; A73G; A263G; C309CCT
D07_548v	100	JQ702675.1	JQ702675.1; K1a11a; Asia_W; 39; A16T; A73G; C150T
D08_549v	100	JQ705964.1	JQ705964.1; J1b1a1c; Asia_W; 39; T10C; A73G; T146C
D09_550v	100	NC_012920_rCRS	NC_012920_rCRS;H2a2a1;Asia_W_Europe;0;REF;REF;REF
D10_551v	100	JQ705928.1	JQ705928.1; H11a; Asia_W_Europe; 16; T195C; A263G; C309CCT
D11_553v	100	JQ706065.1	JQ706065.1; K1a12a1a; Asia_W; 37; A73G; T195C; A263G
D12_554v	100	JQ704761.1	JQ704761.1; K1a4e; Asia_W; 35; A73G; A263G; C309CCT

Supplementary Table S3 BLAST results and statistics[a] for human mtDNA haplotyping and geo-mapping

Query	Greatest positive %	Accession (positive %)	Description (positive %)
A01_HeLa	99.82758621	JQ705753.1	JQ705753.1; L3b1a; Africa_E; 38; A73G; A263G; C315CC
A03_Caski	100	KC911472_HV	KC911472_HV; HV; Asia_W; 11; A263G; C315CC; A750G
A04_SiHa	99.82758621	JQ703841.1	JQ703841.1; X2b+226; Asia_W_America_N; 32; A73G; T195C; A200G
A05_C33-A	99.82425308	JQ706015.1	JQ706015.1; U5a1b1a1; Asia_W_Europe_C; 29; A73G; A263G; C315CC
A06_Doc2C	100	JQ702663.1	JQ702663.1; U2e1b1; Asia_S_W_Europe; 38; A73G; T152C; T217C
A07_151v	100	JQ706012.1	JQ706012.1; H3i1; Asia_W_Europe; 12; T152C; A263G; C315CC
A08_152v	100	JQ705889.1	JQ705889.1; I1a1; Asia_W_SW; 42; A73G; T199C; G203A
A09_153v	100	EU007853_Y	EU007853_Y;Y1a+16189;Asia_NE;24;A73G;T146C;A263G
A10_154v	100	JQ705798.1	JQ705798.1; V7; Europe_S; 18; T72C; A93G; T152C
A11_155v	100	JQ701820.1	JQ701820.1; C1b2; Asia_NE_America_N_S; 43; A73G; A249d; AA290d
A12_156v	100	JQ706012.1	JQ706012.1; H3i1; Asia_W_Europe; 12; T152C; A263G; C315CC
B01_500v	100	JQ706056.1	JQ706056.1; K1a31; Asia_W; 30; A73G; C150T; A263G
B02_503v	100	JQ706012.1	JQ706012.1; H3i1; Asia_W_Europe; 12; T152C; A263G; C315CC
B03_504v	100	JQ705572.1	JQ705572.1; F1f; Asia_SE_E; 32; A73G; A249d; A263G
B04_505v	100	JQ705434.1	JQ705434.1; A2j; Asia_NE_America_N; 33; C64T; A73G; T146C
B05_507v	100	JQ706012.1	JQ706012.1; H3i1; Asia_W_Europe; 12; T152C; A263G; C315CC
B06_508v	100	JQ704350.1	JQ704350.1; A2+(64); Asia_NE_America_N; 35; C64T; A73G; T146C
B07_509v	100	JQ703481.1	JQ703481.1; L0a1a2; Africa_S_SE; 83; C64T; A93G; G185A
B08_511v	100	JQ703650.1	JQ703650.1; H13a1a1a; Asia_W_Europe; 19; T152C; A263G; C309CCT
B09_512v	100	JQ705099.1	JQ705099.1; V7a; Europe_S; 21; T72C; A93G; T195C
B10_513v	99.73890339	JQ704728.1	JQ704728.1; L3e1f; Africa_E; 39; A73G; C150T; A189G
B11_515v	100	JQ704225.1	JQ704225.1; H5a1; Asia_W_Europe; 16; T195C; A240G; A263G
B12_516v	100	JQ705599.1	JQ705599.1; HV0; Asia_W; 14; T72C; A263G; C309CCCT
C01_519v	100	JQ702074.1	JQ702074.1; F1a3a; Asia_SE_E; 37; A73G; A249d; A263G
C02_520v	100	JQ703138.1	JQ703138.1; L3e2b1a; Africa_E; 33; A73G; C150T; T195C
C03_521v	100	JQ705902.1	JQ705902.1; L3e1a3a; Africa_E; 34; A73G; C150T; A200G
C04_523v	100	JQ704931.1	JQ704931.1; L3e3b1; Africa_E; 41; A73G; C150T; T195C
C05_524v	100	JQ706012.1	JQ706012.1; H3i1; Asia_W_Europe; 12; T152C; A263G; C315CC
C06_525v	99.64912281	JQ705272.1	JQ705272.1; U5a1h; Asia_W_Europe_C; 37; A73G; C150T; G207A
C07_528v	100	JQ705362.1	JQ705362.1; H1av; Asia_W_Europe; 14; A263G; C309CCCT; T310C
C08_529v	99.48364888	JQ705975.1	JQ705975.1; H66a; Asia_W_Europe; 11; A263G; C315CC; A750G
C09_532v	100	JQ705585.1	JQ705585.1; C1b4; Asia_NE_America_N_S; 47; A73G; G143A; T152C
C10_533v	99.65517241	JQ705150.1	JQ705150.1; L2a1f; Africa_W_C; 54; A73G; T146C; T152C
C11_534v	100	JQ705378.1	JQ705378.1; I1a1a3; Asia_W_SW; 49; A73G; A189G; T199C
C12_536v	100	JQ705455.1	JQ705455.1; L2a1e1; Africa_W_C; 56; A73G; T146C; T152C
D01_537v	100	KC911472_HV	KC911472_HV; HV; Asia_W; 11; A263G; C315CC; A750G
D02_539v	100	KC911472_HV	KC911472_HV; HV; Asia_W; 11; A263G; C315CC; A750G
D03_542v	100	JQ706018.1	JQ706018.1; NA; NA; 2; C315CC; T16311C;
D04_543v	100	JQ703786.1	JQ703786.1; J1c3; Asia_W; 27; A73G; A263G; C295T
D05_544v	100	JQ705489.1	JQ705489.1; J1c+16261; Asia_W; 29; A73G; G185A; A263G
D06_546v	100	JQ705886.1	JQ705886.1; U5a1a2b1; Asia_W_Europe_C; 33; A73G; A263G; C309CCT
D07_548v	100	JQ702675.1	JQ702675.1; K1a11a; Asia_W; 39; A16T; A73G; C150T
D08_549v	100	JQ705964.1	JQ705964.1; J1b1a1c; Asia_W; 39; T10C; A73G; T146C
D09_550v	100	NC_012920_rCRS	NC_012920_rCRS;H2a2a1;Asia_W_Europe;0;REF;REF;REF
D10_551v	100	JQ705928.1	JQ705928.1; H11a; Asia_W_Europe; 16; T195C; A263G; C309CCT
D11_553v	100	JQ706065.1	JQ706065.1; K1a12a1a; Asia_W; 37; A73G; T195C; A263G
D12_554v	100	JQ704761.1	JQ704761.1; K1a4e; Asia_W; 35; A73G; A263G; C309CCT

Supplementary Table S3 BLAST results and statistics[a] for human mtDNA haplotyping and geo-mapping

Query	Greatest HSP length	Accession (HSP length)	Description (HSP length)
A01_HeLa	580	JQ705753.1	JQ705753.1; L3b1a; Africa_E; 38; A73G; A263G; C315CC
A03_Caski	568	KC911472_HV	KC911472_HV; HV; Asia_W; 11; A263G; C315CC; A750G
A04_SiHa	580	JQ703841.1	JQ703841.1; X2b+226; Asia_W_America_N; 32; A73G; T195C; A200G
A05_C33-A	569	JQ706015.1	JQ706015.1; U5a1b1a1; Asia_W_Europe_C; 29; A73G; A263G; C315CC
A06_Doc2C	579	JQ702663.1	JQ702663.1; U2e1b1; Asia_S_W_Europe; 38; A73G; T152C; T217C
A07_151v	580	JQ706012.1	JQ706012.1; H3i1; Asia_W_Europe; 12; T152C; A263G; C315CC
A08_152v	578	JQ705889.1	JQ705889.1; I1a1; Asia_W_SW; 42; A73G; T199C; G203A
A09_153v	580	JQ702841.1	JQ702841.1; H49a; Asia_W_Europe; 13; A263G; C309CCCT; T310C
A10_154v	581	JQ705798.1	JQ705798.1; V7; Europe_S; 18; T72C; A93G; T152C
A11_155v	580	JQ701820.1	JQ701820.1; C1b2; Asia_NE_America_N_S; 43; A73G; A249d; AA290d
A12_156v	580	JQ706012.1	JQ706012.1; H3i1; Asia_W_Europe; 12; T152C; A263G; C315CC
B01_500v	580	JQ706056.1	JQ706056.1; K1a31; Asia_W; 30; A73G; C150T; A263G
B02_503v	581	JQ706012.1	JQ706012.1; H3i1; Asia_W_Europe; 12; T152C; A263G; C315CC
B03_504v	383	JQ704528.1	JQ704528.1; H3ap; Asia_W_Europe; 15; A73G; T199C; A263G
B04_505v	411	JQ705434.1	JQ705434.1; A2j; Asia_NE_America_N; 33; C64T; A73G; T146C
B05_507v	368	JQ706012.1	JQ706012.1; H3i1; Asia_W_Europe; 12; T152C; A263G; C315CC
B06_508v	580	JQ704350.1	JQ704350.1; A2+(64); Asia_NE_America_N; 35; C64T; A73G; T146C
B07_509v	581	JQ703481.1	JQ703481.1; L0a1a2; Africa_S_SE; 83; C64T; A93G; G185A
B08_511v	580	JQ703650.1	JQ703650.1; H13a1a1a; Asia_W_Europe; 19; T152C; A263G; C309CCT
B09_512v	567	JQ705942.1	JQ705942.1; V5; Europe_S; 17; T72C; A93G; A263G
B10_513v	581	JQ705521.1	JQ705521.1; L3e1f2; Africa_E; 37; A73G; C150T; A189G
B11_515v	580	JQ704225.1	JQ704225.1; H5a1; Asia_W_Europe; 16; T195C; A240G; A263G
B12_516v	580	JQ704767.1	JQ704767.1; V+@72; Europe_S; 14; A263G; C309CCT; T310C
C01_519v	580	JQ702074.1	JQ702074.1; F1a3a; Asia_SE_E; 37; A73G; A249d; A263G
C02_520v	581	JQ703138.1	JQ703138.1; L3e2b1a; Africa_E; 33; A73G; C150T; T195C
C03_521v	581	JQ705902.1	JQ705902.1; L3e1a3a; Africa_E; 34; A73G; C150T; A200G
C04_523v	349	JQ704931.1	JQ704931.1; L3e3b1; Africa_E; 41; A73G; C150T; T195C
C05_524v	278	JQ706012.1	JQ706012.1; H3i1; Asia_W_Europe; 12; T152C; A263G; C315CC
C06_525v	572	JQ705180.1	JQ705180.1; U5b2b4; Asia_W_Europe_C; 33; A73G; C150T; A263G
C07_528v	580	JQ705362.1	JQ705362.1; H1av; Asia_W_Europe; 14; A263G; C309CCCT; T310C
C08_529v	581	JQ705975.1	JQ705975.1; H66a; Asia_W_Europe; 11; A263G; C315CC; A750G
C09_532v	354	JQ705222.1	JQ705222.1; C4a2a1; Asia_NE_America_N_S; 51; A73G; G207A; A249d
C10_533v	580	JQ705150.1	JQ705150.1; L2a1f; Africa_W_C; 54; A73G; T146C; T152C
C11_534v	354	JQ704077.1	JQ704077.1; I1f; Asia_W_SW; 41; A73G; T199C; T204C
C12_536v	384	JQ702959.1	JQ702959.1; T1a1k1; Asia_W; 38; A73G; T146C; T152C
D01_537v	377	KC911472_HV	KC911472_HV; HV; Asia_W; 11; A263G; C315CC; A750G
D02_539v	309	KC911472_HV	KC911472_HV; HV; Asia_W; 11; A263G; C315CC; A750G
D03_542v	580	JQ706018.1	JQ706018.1; NA; NA; 2; C315CC; T16311C;
D04_543v	571	JQ701995.1	JQ701995.1; J1c3f; Asia_W; 30; A73G; A263G; C295T
D05_544v	580	JQ705489.1	JQ705489.1; J1c+16261; Asia_W; 29; A73G; G185A; A263G
D06_546v	580	JQ705886.1	JQ705886.1; U5a1a2b1; Asia_W_Europe_C; 33; A73G; A263G; C309CCT
D07_548v	568	JQ705775.1	JQ705775.1; K1a4d; Asia_W; 37; A73G; A263G; C309CCT
D08_549v	580	JQ705964.1	JQ705964.1; J1b1a1c; Asia_W; 39; T10C; A73G; T146C
D09_550v	580	NC_012920_rCRS	NC_012920_rCRS;H2a2a1;Asia_W_Europe;0;REF;REF;REF
D10_551v	580	JQ705928.1	JQ705928.1; H11a; Asia_W_Europe; 16; T195C; A263G; C309CCT
D11_553v	580	JQ706065.1	JQ706065.1; K1a12a1a; Asia_W; 37; A73G; T195C; A263G
D12_554v	581	JQ704761.1	JQ704761.1; K1a4e; Asia_W; 35; A73G; A263G; C309CCT

Supplementary Table S3 BLAST results and statistics[a] for human mtDNA haplotyping and geo-mapping

Query	Greatest bit score	Accession (bit score)	Description (bit score)
A01_HeLa	1042.73	JQ705753.1	JQ705753.1; L3b1a; Africa_E; 38; A73G; A263G; C315CC
A03_Caski	1025.6	KC911472_HV	KC911472_HV; HV; Asia_W; 11; A263G; C315CC; A750G
A04_SiHa	1044.54	JQ703841.1	JQ703841.1; X2b+226; Asia_W_America_N; 32; A73G; T195C; A200G
A05_C33-A	1022.89	JQ706015.1	JQ706015.1; U5a1b1a1; Asia_W_Europe_C; 29; A73G; A263G; C315CC
A06_Doc2C	1045.44	JQ702663.1	JQ702663.1; U2e1b1; Asia_S_W_Europe; 38; A73G; T152C; T217C
A07_151v	1047.24	JQ706012.1	JQ706012.1; H3i1; Asia_W_Europe; 12; T152C; A263G; C315CC
A08_152v	1043.63	JQ705889.1	JQ705889.1; I1a1; Asia_W_SW; 42; A73G; T199C; G203A
A09_153v	1029.21	JQ702841.1	JQ702841.1; H49a; Asia_W_Europe; 13; A263G; C309CCCT; T310C
A10_154v	1049.04	JQ705798.1	JQ705798.1; V7; Europe_S; 18; T72C; A93G; T152C
A11_155v	1047.24	JQ701820.1	JQ701820.1; C1b2; Asia_NE_America_N_S; 43; A73G; A249d; AA290d
A12_156v	1047.24	JQ706012.1	JQ706012.1; H3i1; Asia_W_Europe; 12; T152C; A263G; C315CC
B01_500v	1047.24	JQ706056.1	JQ706056.1; K1a31; Asia_W; 30; A73G; C150T; A263G
B02_503v	1049.04	JQ706012.1	JQ706012.1; H3i1; Asia_W_Europe; 12; T152C; A263G; C315CC
B03_504v	685.665	JQ704786.1	JQ704786.1; T2j; Asia_W; 39; A73G; A249d; A263G
B04_505v	742.471	JQ705434.1	JQ705434.1; A2j; Asia_NE_America_N; 33; C64T; A73G; T146C
B05_507v	664.926	JQ706012.1	JQ706012.1; H3i1; Asia_W_Europe; 12; T152C; A263G; C315CC
B06_508v	1038.22	JQ704350.1	JQ704350.1; A2+(64); Asia_NE_America_N; 35; C64T; A73G; T146C
B07_509v	1049.04	JQ703481.1	JQ703481.1; L0a1a2; Africa_S_SE; 83; C64T; A93G; G185A
B08_511v	1047.24	JQ703650.1	JQ703650.1; H13a1a1a; Asia_W_Europe; 19; T152C; A263G; C309CCT
B09_512v	1021.99	JQ705099.1	JQ705099.1; V7a; Europe_S; 21; T72C; A93G; T195C
B10_513v	1040.03	JQ705521.1	JQ705521.1; L3e1f2; Africa_E; 37; A73G; C150T; A189G
B11_515v	1047.24	JQ704225.1	JQ704225.1; H5a1; Asia_W_Europe; 16; T195C; A240G; A263G
B12_516v	1042.73	JQ704767.1	JQ704767.1; V+@72; Europe_S; 14; A263G; C309CCT; T310C
C01_519v	1047.24	JQ702074.1	JQ702074.1; F1a3a; Asia_SE_E; 37; A73G; A249d; A263G
C02_520v	1044.54	JQ703138.1	JQ703138.1; L3e2b1a; Africa_E; 33; A73G; C150T; T195C
C03_521v	1040.03	JQ705902.1	JQ705902.1; L3e1a3a; Africa_E; 34; A73G; C150T; A200G
C04_523v	630.662	JQ704931.1	JQ704931.1; L3e3b1; Africa_E; 41; A73G; C150T; T195C
C05_524v	502.623	JQ706012.1	JQ706012.1; H3i1; Asia_W_Europe; 12; T152C; A263G; C315CC
C06_525v	1020.19	JQ705272.1	JQ705272.1; U5a1h; Asia_W_Europe_C; 37; A73G; C150T; G207A
C07_528v	1047.24	JQ705362.1	JQ705362.1; H1av; Asia_W_Europe; 14; A263G; C309CCCT; T310C
C08_529v	1035.52	JQ705975.1	JQ705975.1; H66a; Asia_W_Europe; 11; A263G; C315CC; A750G
C09_532v	634.269	JQ705585.1	JQ705585.1; C1b4; Asia_NE_America_N_S; 47; A73G; G143A; T152C
C10_533v	1038.22	JQ705150.1	JQ705150.1; L2a1f; Africa_W_C; 54; A73G; T146C; T152C
C11_534v	622.547	JQ705378.1	JQ705378.1; I1a1a3; Asia_W_SW; 49; A73G; A189G; T199C
C12_536v	690.173	JQ705455.1	JQ705455.1; L2a1e1; Africa_W_C; 56; A73G; T146C; T152C
D01_537v	681.156	KC911472_HV	KC911472_HV; HV; Asia_W; 11; A263G; C315CC; A750G
D02_539v	558.527	KC911472_HV	KC911472_HV; HV; Asia_W; 11; A263G; C315CC; A750G
D03_542v	1047.24	JQ706018.1	JQ706018.1; NA; NA; 2; C315CC; T16311C;
D04_543v	1024.7	JQ703786.1	JQ703786.1; J1c3; Asia_W; 27; A73G; A263G; C295T
D05_544v	1047.24	JQ705489.1	JQ705489.1; J1c+16261; Asia_W; 29; A73G; G185A; A263G
D06_546v	1047.24	JQ705886.1	JQ705886.1; U5a1a2b1; Asia_W_Europe_C; 33; A73G; A263G; C309CCT
D07_548v	1017.48	JQ702675.1	JQ702675.1; K1a11a; Asia_W; 39; A16T; A73G; C150T
D08_549v	1047.24	JQ705964.1	JQ705964.1; J1b1a1c; Asia_W; 39; T10C; A73G; T146C
D09_550v	1047.24	NC_012920_rCRS	NC_012920_rCRS;H2a2a1;Asia_W_Europe;0;REF;REF;REF
D10_551v	1047.24	JQ705928.1	JQ705928.1; H11a; Asia_W_Europe; 16; T195C; A263G; C309CCT
D11_553v	1047.24	JQ706065.1	JQ706065.1; K1a12a1a; Asia_W; 37; A73G; T195C; A263G
D12_554v	1049.04	JQ704761.1	JQ704761.1; K1a4e; Asia_W; 35; A73G; A263G; C309CCT

[a] BLAST statistics nomenclature and definitions from left to right of table columns [55].

HSP (n), Number of high scoring pairs from the source database.

Lowest E-val, Lowest Expect value.

Accession (E-val), Accession name of the matched database sequence with the lowest E-value.

Hit, Name of the sequence found in the BLAST search with the max score.

Total score, Total alignment score for all HSPs.

Max score, Maximum (best) score or highest alignment score of all HSPs.

Min E-value, Minimum (best) e-value of all HSPs.

Max bit score, Maximum (best) bit score of all HSPs.

Max id, Maximum number of identical residues in the query and Hit sequence.

Max %id, Percentage of maximum identical residues in the query and Hit sequence.

Max pos, Maximum number of similar but not necessarily identical residues in the query and Hit sequence.

Max %pos, Percentage of maximum similar but not necessarily identical residues in the query and Hit sequence.
