# Supplementary material for: A Customized Human Mitochondrial DNA Database (hMITO DB v1.0) for Rapid Sequence Analysis, Haplotyping and Geo-Mapping
Source: Int J Mol Sci. 2023 Aug 31;24(17):13505. doi: 10.3390/ijms241713505 (PMC10488239; doi:10.3390/ijms241713505)
Supplement: Supplementary file 1 [file ijms-24-13505-s001.zip › ijms-2550861-supplementary/Table S4.pdf]

Supplementary Table S4 Human mitochondrial database (hMITO DB v1.0) metadata[a]

Row	Name (accession no.)	Description	Size	Geo_Region	Haplogroup	Macro_ Haplo	Macro_ Haplo2	Total Variants
1	JQ701803.1	JQ701803.1; U5b1b1e; Asia_W_Europe_C; 32; A73G; C150T; T152C	16570	Asia_W_Europe_C	U5b1b1e	U	U5	32
2	JQ701804.1	JQ701804.1; T1a1; Asia_W; 33; A73G; T152C; A263G	16569	Asia_W	T1a1	T	T	33
3	JQ701805.1	JQ701805.1; N1b1a5; Africa_NE_Asia_W; 39; A73G; T152C; A263G	16570	Africa_NE_Asia_W	N1b1a5	N	N1	39
4	JQ701806.1	JQ701806.1; H1j; Asia_W_Europe; 15; A263G; C309CCT; T310C	16570	Asia_W_Europe	H1j	H	H	15
5	JQ701807.1	JQ701807.1; J2a1a1; Asia_W; 41; A73G; G75A; C150T	16568	Asia_W	J2a1a1	J	J	41
6	JQ701808.1	JQ701808.1; K2a9; Asia_W; 36; A73G; T146C; A263G	16569	Asia_W	K2a9	K	K	36
7	JQ701809.1	JQ701809.1; H10e; Asia_W_Europe; 12; A263G; C309CCT; T310C	16570	Asia_W_Europe	H10e	H	H	12
8	JQ701810.1	JQ701810.1; K1a4a1a2b; Asia_W; 42; A73G; T146C; A263G	16572	Asia_W	K1a4a1a2b	K	K	42
9	JQ701811.1	JQ701811.1; K2a2a1; Asia_W; 36; A73G; T146C; T152C	16569	Asia_W	K2a2a1	K	K	36
10	JQ701812.1	JQ701812.1; H2a5b1; Asia_W_Europe; 11; A249d; A263G; C309CCCT	16570	Asia_W_Europe	H2a5b1	H	H	11
11	JQ701813.1	JQ701813.1; H1bt1; Asia_W_Europe; 13; A263G; C315CC; A750G	16569	Asia_W_Europe	H1bt1	H	H	13
12	JQ701814.1	JQ701814.1; L2a1f1; Africa_W_C; 54; A73G; T146C; T152C	16569	Africa_W_C	L2a1f1	L2	L2	54
13	JQ701815.1	JQ701815.1; H60a; Asia_W_Europe; 15; A73G; C150T; A263G	16570	Asia_W_Europe	H60a	H	H	15
14	JQ701816.1	JQ701816.1; H13a2b1; Asia_W_Europe; 15; A263G; C315CC; G709A	16570	Asia_W_Europe	H13a2b1	H	H	15
15	JQ701817.1	JQ701817.1; X2d2; Asia_W_America_N; 33; A73G; T195C; A263G	16571	Asia_W_America_N	X2d2	X	X	33
16	JQ701818.1	JQ701818.1; H13a1a1; Asia_W_Europe; 15; C150T; A263G; T310C	16569	Asia_W_Europe	H13a1a1	H	H	15
17	JQ701819.1	JQ701819.1; H2a1j; Asia_W_Europe; 11; A263G; C309CCT; T310C	16570	Asia_W_Europe	H2a1j	H	H	11
18	JQ701820.1	JQ701820.1; C1b2; Asia_NE_America_N_S; 43; A73G; A249d; AA290d	16564	Asia_NE_America_N_S	C1b2	C	C	43
19	JQ701821.1	JQ701821.1; J1c7; Asia_W; 33; A73G; G185A; G228A	16569	Asia_W	J1c7	J	J	33
20	JQ701822.1	JQ701822.1; J1c3e1; Asia_W; 34; A73G; C150T; G185A	16569	Asia_W	J1c3e1	J	J	34
21	JQ701823.1	JQ701823.1; L1b1a3b; Africa_C; 81; A73G; T152C; C182T	16567	Africa_C	L1b1a3b	L1	L1	81
22	JQ701824.1	JQ701824.1; W3b1; Asia_W; 36; A73G; C194T; T195C	16570	Asia_W	W3b1	W	W	36
23	JQ701825.1	JQ701825.1; H1n+146; Asia_W_Europe; 13; T146C; A263G; C309CCCT	16571	Asia_W_Europe	H1n+146	H	H	13
24	JQ701826.1	JQ701826.1; H2a5b; Asia_W_Europe; 9; A263G; C309CCT; T310C	16570	Asia_W_Europe	H2a5b	H	H	9
25	JQ701827.1	JQ701827.1; W5a; Asia_W; 37; A73G; A189G; C194T	16569	Asia_W	W5a	W	W	37
26	JQ701828.1	JQ701828.1; T2b5a1; Asia_W; 39; A73G; A263G; C309CCT	16571	Asia_W	T2b5a1	T	T	39
27	JQ701829.1	JQ701829.1; L3e2b1a2; Africa_E; 35; A73G; C150T; T195C	16569	Africa_E	L3e2b1a2	L3	L3	35
28	JQ701830.1	JQ701830.1; H1a3; Asia_W_Europe; 15; A73G; A263G; C309CCT	16570	Asia_W_Europe	H1a3	H	H	15
29	JQ701831.1	JQ701831.1; H1c1; Asia_W_Europe; 13; A263G; C315CC; T477C	16571	Asia_W_Europe	H1c1	H	H	13
30	JQ701832.1	JQ701832.1; T2b2b1; Asia_W; 34; A73G; A263G; C315CC	16569	Asia_W	T2b2b1	T	T	34
31	JQ701833.1	JQ701833.1; L2b1a3; Africa_W_C; 69; A73G; C150T; T152C	16567	Africa_W_C	L2b1a3	L2	L2	69
32	JQ701834.1	JQ701834.1; U5a1a1a; Asia_W_Europe_C; 29; A73G; T152C; A263G	16570	Asia_W_Europe_C	U5a1a1a	U	U5	29
33	JQ701835.1	JQ701835.1; K1a4a1; Asia_W; 38; A73G; A153G; A263G	16569	Asia_W	K1a4a1	K	K	38
34	JQ701836.1	JQ701836.1; H2a2b1a1; Asia_W_Europe; 9; A263G; C309CCCT; T310C	16571	Asia_W_Europe	H2a2b1a1	H	H	9
35	JQ701837.1	JQ701837.1; W1c1; Asia_W; 36; A73G; A189G; T195C	16569	Asia_W	W1c1	W	W	36
36	JQ701838.1	JQ701838.1; K1a4d; Asia_W; 34; A73G; A263G; C315CC	16567	Asia_W	K1a4d	K	K	34
37	JQ701839.1	JQ701839.1; H4a1a1a; Asia_W_Europe; 19; A73G; A263G; C309CCT	16568	Asia_W_Europe	H4a1a1a	H	H	19
38	JQ701840.1	JQ701840.1; T2b; Asia_W; 36; A73G; A263G; C309CCCT	16569	Asia_W	T2b	T	T	36
39	JQ701841.1	JQ701841.1; U4a2; Asia_N_Europe_N; 30; A73G; T195C; A263G	16568	Asia_N_Europe_N	U4a2	U	U4	30
40	JQ701842.1	JQ701842.1; H13a1a3; Asia_W_Europe; 14; A263G; C309CCT; T310C	16570	Asia_W_Europe	H13a1a3	H	H	14
41	JQ701843.1	JQ701843.1; H4; Asia_W_Europe; 16; T152C; A263G; C309CCT	16568	Asia_W_Europe	H4	H	H	16

Supplementary Table S4 Human mitochondrial database (hMITO DB v1.0) metadata[a]

Row	Name (accession no.)	Description	Size	Geo_Region	Haplogroup	Macro_ Haplo	Macro_ Haplo2	Total Variants
42	JQ701844.1	JQ701844.1; W3a1; Asia_W; 38; T10C; A73G; T152C	16570	Asia_W	W3a1	W	W	38
43	JQ701845.1	JQ701845.1; K1c2; Asia_W; 36; T55C; A73G; T146C	16568	Asia_W	K1c2	K	K	36
44	JQ701846.1	JQ701846.1; H1bv1; Asia_W_Europe; 15; A263G; C309CCCT; T310C	16571	Asia_W_Europe	H1bv1	H	H	15
45	JQ701847.1	JQ701847.1; J1b1a1a; Asia_W; 37; A73G; C242T; A263G	16569	Asia_W	J1b1a1a	J	J	37
46	JQ701848.1	JQ701848.1; H2a1a2; Asia_W_Europe; 13; A263G; C315CC; A750G	16569	Asia_W_Europe	H2a1a2	H	H	13
47	JQ701849.1	JQ701849.1; H10; Asia_W_Europe; 10; A263G; C309CCT; T310C	16570	Asia_W_Europe	H10	H	H	10
48	JQ701850.1	JQ701850.1; H7; Asia_W_Europe; 12; T152C; A263G; C309CCT	16570	Asia_W_Europe	H7	H	H	12
49	JQ701851.1	JQ701851.1; J1c2h; Asia_W; 34; A73G; G185A; A188G	16569	Asia_W	J1c2h	J	J	34
50	JQ701852.1	JQ701852.1; J1b1a1; Asia_W; 37; A73G; G185A; G228A	16569	Asia_W	J1b1a1	J	J	37
51	JQ701853.1	JQ701853.1; T2e; Asia_W; 34; A73G; C150T; A263G	16570	Asia_W	T2e	T	T	34
52	JQ701854.1	JQ701854.1; J1c3f; Asia_W; 32; A73G; G228A; A263G	16569	Asia_W	J1c3f	J	J	32
53	JQ701855.1	JQ701855.1; A2ak; Asia_NE_America_N; 41; C64T; A73G; T146C	16569	Asia_NE_America_N	A2ak	A	A	41
54	JQ701856.1	JQ701856.1; K1a2a1; Asia_W; 37; A73G; A263G; C309CCT	16570	Asia_W	K1a2a1	K	K	37
55	JQ701857.1	JQ701857.1; U2e1c1; Asia_S_W_Europe; 44; A73G; T152C; A200G	16571	Asia_S_W_Europe	U2e1c1	U	U2	44
56	JQ701858.1	JQ701858.1; K1a1b2a1a; Asia_W; 41; A73G; C114T; T152C	16569	Asia_W	K1a1b2a1a	K	K	41
57	JQ701859.1	JQ701859.1; J1c7a; Asia_W; 34; A73G; G185A; G228A	16570	Asia_W	J1c7a	J	J	34
58	JQ701860.1	JQ701860.1; H10b; Asia_W_Europe; 11; A263G; C309CCT; T310C	16570	Asia_W_Europe	H10b	H	H	11
59	JQ701861.1	JQ701861.1; H1+16189; Asia_W_Europe; 13; A263G; C309CCCT; T310C	16571	Asia_W_Europe	H1+16189	H	H	13
60	JQ701862.1	JQ701862.1; B2; Asia_SE_E_America_N_S; 36; A73G; A263G; C309CCT	16563	Asia_SE_E_America_N_S	B2	B	B	36
61	JQ701863.1	JQ701863.1; H1ag1; Asia_W_Europe; 12; A263G; C309CCT; T310C	16570	Asia_W_Europe	H1ag1	H	H	12
62	JQ701864.1	JQ701864.1; H3h6; Asia_W_Europe; 16; A263G; C309CCT; T310C	16570	Asia_W_Europe	H3h6	H	H	16
63	JQ701865.1	JQ701865.1; H5; Asia_W_Europe; 15; A263G; C309CCT; T310C	16570	Asia_W_Europe	H5	H	H	15
64	JQ701866.1	JQ701866.1; U5a1a1d1; Asia_W_Europe_C; 32; A73G; G185A; A189G	16570	Asia_W_Europe_C	U5a1a1d1	U	U5	32
65	JQ701867.1	JQ701867.1; T2b37; Asia_W; 36; A73G; T152C; A263G	16569	Asia_W	T2b37	T	T	36
66	JQ701868.1	JQ701868.1; D1f3; Asia_E_America_N_S; 40; A73G; T146C; A263G	16570	Asia_E_America_N_S	D1f3	D	D	40
67	JQ701869.1	JQ701869.1; U5a1a1; Asia_N_Europe_N; 30; A73G; T146C; A263G	16570	Asia_N_Europe_N	U5a1a1	U	U4	30
68	JQ701870.1	JQ701870.1; J1c15; Asia_W; 34; A73G; G185A; G228A	16568	Asia_W	J1c15	J	J	34
69	JQ701871.1	JQ701871.1; X2b5; Asia_W_America_N; 34; A73G; A153G; T195C	16569	Asia_W_America_N	X2b5	X	X	34
70	JQ701872.1	JQ701872.1; H72; Asia_W_Europe; 12; A263G; C315CC; A750G	16569	Asia_W_Europe	H72	H	H	12
71	JQ701873.1	JQ701873.1; H1b1+16362; Asia_W_Europe; 16; T152C; A263G; C315CC	16567	Asia_W_Europe	H1b1+16362	H	H	16
72	JQ701874.1	JQ701874.1; H1e2; Asia_W_Europe; 12; A263G; C315CC; A750G	16569	Asia_W_Europe	H1e2	H	H	12
73	JQ701875.1	JQ701875.1; H5a4a1a; Asia_W_Europe; 20; A263G; T282C; C315CC	16569	Asia_W_Europe	H5a4a1a	H	H	20
74	JQ701876.1	JQ701876.1; U4b1a2b; Asia_N_Europe_N; 36; A73G; T195C; A263G	16570	Asia_N_Europe_N	U4b1a2b	U	U4	36
75	JQ701877.1	JQ701877.1; H3; Asia_W_Europe; 9; A263G; C315CC; A750G	16569	Asia_W_Europe	H3	H	H	9
76	JQ701878.1	JQ701878.1; J1c2; Asia_W; 32; A73G; G185A; A188G	16569	Asia_W	J1c2	J	J	32
77	JQ701879.1	JQ701879.1; H1h1; Asia_W_Europe; 15; A263G; C309CCT; T310C	16570	Asia_W_Europe	H1h1	H	H	15
78	JQ701880.1	JQ701880.1; T2a1a7; Asia_W; 35; A73G; A263G; C315CC	16569	Asia_W	T2a1a7	T	T	35
79	JQ701881.1	JQ701881.1; H1ax; Asia_W_Europe; 11; A263G; C315CC; A750G	16569	Asia_W_Europe	H1ax	H	H	11
80	JQ701882.1	JQ701882.1; H5; Asia_W_Europe; 17; A263G; C315CC; C456T	16569	Asia_W_Europe	H5	H	H	17
81	JQ701883.1	JQ701883.1; K2b1a; Asia_W; 35; A73G; T146C; A263G	16570	Asia_W	K2b1a	K	K	35
82	JQ701884.1	JQ701884.1; K2a; Asia_W; 33; A73G; T146C; T152C	16569	Asia_W	K2a	K	K	33

Supplementary Table S4 Human mitochondrial database (hMITO DB v1.0) metadata[a]

Row	Name (accession no.)	Description	Size	Geo_Region	Haplogroup	Macro_ Haplo	Macro_ Haplo2	Total Variants
83	JQ701885.1	JQ701885.1; H2c; Asia_W_Europe; 13; T152C; A263G; C309CCT	16568	Asia_W_Europe	H2c	H	H	13
84	JQ701886.1	JQ701886.1; X2b5; Asia_W_America_N; 34; A73G; A153G; T195C	16569	Asia_W_America_N	X2b5	X	X	34
85	JQ701887.1	JQ701887.1; U2e1; Asia_S_W_Europe; 42; A73G; T152C; T217C	16572	Asia_S_W_Europe	U2e1	U	U2	42
86	JQ701888.1	JQ701888.1; H1a; Asia_W_Europe; 15; A73G; A153G; A263G	16570	Asia_W_Europe	H1a	H	H	15
87	JQ701889.1	JQ701889.1; H; Asia_W_Europe; 15; A263G; C309CCCT; T310C	16571	Asia_W_Europe	H	H	H	15
88	JQ701890.1	JQ701890.1; U2e2a1d; Asia_S_W_Europe; 42; A73G; T152C; T217C	16570	Asia_S_W_Europe	U2e2a1d	U	U2	42
89	JQ701891.1	JQ701891.1; C1b11; Asia_NE_America_N_S; 48; A73G; A249d; A263G	16566	Asia_NE_America_N_S	C1b11	C	C	48
90	JQ701892.1	JQ701892.1; H15a1a1; Asia_W_Europe; 15; T55C; A56AC; A263G	16571	Asia_W_Europe	H15a1a1	H	H	15
91	JQ701893.1	JQ701893.1; H; Asia_W_Europe; 12; A200G; A263G; C309CCCT	16571	Asia_W_Europe	H	H	H	12
92	JQ701894.1	JQ701894.1; I5a2+16086; Asia_W_SW; 36; A73G; T152C; T199C	16571	Asia_W_SW	I5a2+16086	I	I	36
93	JQ701895.1	JQ701895.1; K1b1a1a; Asia_W; 44; A73G; T152C; T199C	16572	Asia_W	K1b1a1a	K	K	44
94	JQ701896.1	JQ701896.1; H5a3a2; Asia_W_Europe; 17; T152C; A263G; C309CCT	16570	Asia_W_Europe	H5a3a2	H	H	17
95	JQ701897.1	JQ701897.1; T2b4f; Asia_W; 36; A73G; T152C; A263G	16570	Asia_W	T2b4f	T	T	36
96	JQ701898.1	JQ701898.1; H3; Asia_W_Europe; 11; A263G; C315CC; A523ACA	16571	Asia_W_Europe	H3	H	H	11
97	JQ701899.1	JQ701899.1; H1a(H1ar); Asia_W_Europe; 4; A183G; C315CC; A1555G	16569	Asia_W_Europe	H1a(H1ar)	H	H	4
98	JQ701900.1	JQ701900.1; I1a1e; Asia_W_SW; 43; A73G; T199C; G203A	16570	Asia_W_SW	I1a1e	I	I	43
99	JQ701901.1	JQ701901.1; L1c1c; Africa_C; 86; A73G; T152C; C182T	16566	Africa_C	L1c1c	L1	L1	86
100	JQ701902.1	JQ701902.1; J1b1a1; Asia_W; 39; A73G; C242T; A263G	16569	Asia_W	J1b1a1	J	J	39
101	JQ701903.1	JQ701903.1; U5a1a1d1; Asia_W_Europe_C; 35; A73G; G185A; A189G	16572	Asia_W_Europe_C	U5a1a1d1	U	U5	35
102	JQ701904.1	JQ701904.1; H2a2a; Asia_W_Europe; 7; T195C; A263G; C309CCCT	16571	Asia_W_Europe	H2a2a	H	H	7
103	JQ701905.1	JQ701905.1; X2k; Asia_W_America_N; 32; A73G; A153G; T195C	16568	Asia_W_America_N	X2k	X	X	32
104	JQ701906.1	JQ701906.1; A2d1; Asia_NE_America_N; 36; C64T; A73G; T146C	16559	Asia_NE_America_N	A2d1	A	A	36
105	JQ701907.1	JQ701907.1; J1b1a1b; Asia_W; 38; A73G; C242T; A263G	16569	Asia_W	J1b1a1b	J	J	38
106	JQ701908.1	JQ701908.1; HV7; Asia_W; 17; A263G; C315CC; A523ACACA	16573	Asia_W	HV7	HV	HV	17
107	JQ701909.1	JQ701909.1; I4a; Asia_W_SW; 36; A73G; T199C; T204C	16570	Asia_W_SW	I4a	I	I	36
108	JQ701910.1	JQ701910.1; H59; Asia_W_Europe; 12; A263G; C309CCT; T310C	16572	Asia_W_Europe	H59	H	H	12
109	JQ701911.1	JQ701911.1; U4b1b1; Asia_N_Europe_N; 32; A73G; T146C; T152C	16569	Asia_N_Europe_N	U4b1b1	U	U4	32
110	JQ701912.1	JQ701912.1; W5b1a; Asia_W; 38; A56ATC; TG65-; A73G	16569	Asia_W	W5b1a	W	W	38
111	JQ701913.1	JQ701913.1; U5a1a1; Asia_W_Europe_C; 32; A73G; A263G; C315CC	16569	Asia_W_Europe_C	U5a1a1	U	U5	32
112	JQ701914.1	JQ701914.1; L2a1m1a; Africa_W_C; 55; A73G; G143A; T146C	16561	Africa_W_C	L2a1m1a	L2	L2	55
113	JQ701915.1	JQ701915.1; U5b2a1a1a; Asia_W_Europe_C; 29; A73G; C150T; A263G	16569	Asia_W_Europe_C	U5b2a1a1a	U	U5	29
114	JQ701916.1	JQ701916.1; J1b1a1; Asia_W; 37; A73G; C242T; A263G	16569	Asia_W	J1b1a1	J	J	37
115	JQ701917.1	JQ701917.1; H7d4; Asia_W_Europe; 16; A263G; C309CCCT; T310C	16571	Asia_W_Europe	H7d4	H	H	16
116	JQ701918.1	JQ701918.1; H18; Asia_W_Europe; 12; A263G; C315CC; A523ACA	16571	Asia_W_Europe	H18	H	H	12
117	JQ701919.1	JQ701919.1; V18a; Europe_S; 20; T72C; A263G; C309CCCT	16571	Europe_S	V18a	V	V	20
118	JQ701920.1	JQ701920.1; J1c3; Asia_W; 30; A73G; G228A; A263G	16570	Asia_W	J1c3	J	J	30
119	JQ701921.1	JQ701921.1; J1c5a1; Asia_W; 31; A73G; G185A; G228A	16569	Asia_W	J1c5a1	J	J	31
120	JQ701922.1	JQ701922.1; U5a2c3; Asia_W_Europe_C; 31; A73G; A263G; C309CCT	16570	Asia_W_Europe_C	U5a2c3	U	U5	31
121	JQ701923.1	JQ701923.1; U7a3b; Asia_W; 37; A73G; C151T; T152C	16568	Asia_W	U7a3b	U	U7	37
122	JQ701924.1	JQ701924.1; N1a1a2; Africa_NE_Asia_W; 35; A73G; T152C; T199C	16571	Africa_NE_Asia_W	N1a1a2	N	N1	35
123	JQ701925.1	JQ701925.1; H6a1a1; Asia_W_Europe; 18; T239C; A263G; C309CCT	16571	Asia_W_Europe	H6a1a1	H	H	18

Supplementary Table S4 Human mitochondrial database (hMITO DB v1.0) metadata[a]

Row	Name (accession no.)	Description	Size	Geo_Region	Haplogroup	Macro_ Haplo	Macro_ Haplo2	Total Variants
124	JQ701926.1	JQ701926.1; L2a1c4a; Africa_W_C; 52; A73G; G143A; T146C	16569	Africa_W_C	L2a1c4a	L2	L2	52
125	JQ701927.1	JQ701927.1; T2b3d; Asia_W; 39; A73G; C151T; T199C	16570	Asia_W	T2b3d	T	T	39
126	JQ701928.1	JQ701928.1; X2c1; Asia_W_America_N; 30; A73G; A153G; T195C	16569	Asia_W_America_N	X2c1	X	X	30
127	JQ701929.1	JQ701929.1; V3c; Europe_S; 17; A263G; C309CCCT; T310C	16571	Europe_S	V3c	V	V	17
128	JQ701930.1	JQ701930.1; HV0a1a; Asia_W; 20; T72C; A200G; A263G	16568	Asia_W	HV0a1a	HV	HV	20
129	JQ701931.1	JQ701931.1; H1; Asia_W_Europe; 16; A263G; C315CC; A750G	16569	Asia_W_Europe	H1	H	H	16
130	JQ701932.1	JQ701932.1; H27e; Asia_W_Europe; 15; C64T; A263G; C309CCT	16561	Asia_W_Europe	H27e	H	H	15
131	JQ701933.1	JQ701933.1; H5a2; Asia_W_Europe; 15; A263G; C309CCT; T310C	16570	Asia_W_Europe	H5a2	H	H	15
132	JQ701934.1	JQ701934.1; T2c1a2; Asia_W; 39; A73G; A263G; C309CCT	16572	Asia_W	T2c1a2	T	T	39
133	JQ701935.1	JQ701935.1; H3h; Asia_W_Europe; 12; A263G; C315CC; A750G	16569	Asia_W_Europe	H3h	H	H	12
134	JQ701936.1	JQ701936.1; H60a; Asia_W_Europe; 15; G185A; A263G; C309CCCT	16571	Asia_W_Europe	H60a	H	H	15
135	JQ701937.1	JQ701937.1; U4b2a; Asia_S_W_Europe; 32; A73G; A263G; C309CCT	16572	Asia_S_W_Europe	U4b2a	U	U4	32
136	JQ701938.1	JQ701938.1; V+@72; Europe_S; 14; A263G; C309CCT; T310C	16570	Europe_S	V+@72	V	V	14
137	JQ701939.1	JQ701939.1; T2b28; Asia_W; 36; A73G; A263G; C315CC	16569	Asia_W	T2b28	T	T	36
138	JQ701940.1	JQ701940.1; T1a1; Asia_W; 36; A73G; T152C; T195C	16570	Asia_W	T1a1	T	T	36
139	JQ701941.1	JQ701941.1; H5a2; Asia_W_Europe; 14; A263G; C309CCCT; T310C	16571	Asia_W_Europe	H5a2	H	H	14
140	JQ701942.1	JQ701942.1; I2; Asia_W_SW; 37; A73G; T152C; T199C	16572	Asia_W_SW	I2	I	I	37
141	JQ701943.1	JQ701943.1; H8c1; Asia_W_Europe; 19; T146C; T152C; T195C	16569	Asia_W_Europe	H8c1	H	H	19
142	JQ701944.1	JQ701944.1; H1a8; Asia_W_Europe; 13; A73G; A263G; C309CCT	16570	Asia_W_Europe	H1a8	H	H	13
143	JQ701945.1	JQ701945.1; H1g1; Asia_W_Europe; 12; A263G; C315CC; A750G	16569	Asia_W_Europe	H1g1	H	H	12
144	JQ701946.1	JQ701946.1; X2b4; Asia_W_America_N; 32; A73G; A153G; T195C	16571	Asia_W_America_N	X2b4	X	X	32
145	JQ701947.1	JQ701947.1; U2e2a1d; Asia_S_W_Europe; 40; A73G; T152C; T217C	16571	Asia_S_W_Europe	U2e2a1d	U	U2	40
146	JQ701948.1	JQ701948.1; H13a1a; Asia_W_Europe; 16; A153G; A263G; C309CCT	16570	Asia_W_Europe	H13a1a	H	H	16
147	JQ701949.1	JQ701949.1; H5a1c1a; Asia_W_Europe; 17; A263G; C315CC; C456T	16567	Asia_W_Europe	H5a1c1a	H	H	17
148	JQ701950.1	JQ701950.1; H1a; Asia_W_Europe; 13; A73G; A263G; C309CCT	16570	Asia_W_Europe	H1a	H	H	13
149	JQ701951.1	JQ701951.1; H4a1; Asia_W_Europe; 19; T152C; C182T; A263G	16567	Asia_W_Europe	H4a1	H	H	19
150	JQ701952.1	JQ701952.1; H1e1a; Asia_W_Europe; 13; A263G; C315CC; A750G	16569	Asia_W_Europe	H1e1a	H	H	13
151	JQ701953.1	JQ701953.1; H26c; Asia_W_Europe; 13; T146C; T152C; A263G	16569	Asia_W_Europe	H26c	H	H	13
152	JQ701954.1	JQ701954.1; L2c; Africa_W_C; 65; A73G; A93G; A95C	16568	Africa_W_C	L2c	L2	L2	65
153	JQ701955.1	JQ701955.1; H6a1b; Asia_W_Europe; 19; A200G; T239C; A263G	16570	Asia_W_Europe	H6a1b	H	H	19
154	JQ701956.1	JQ701956.1; H23; Asia_W_Europe; 10; A263G; C315CC; A750G	16569	Asia_W_Europe	H23	H	H	10
155	JQ701957.1	JQ701957.1; I4a; Asia_W_SW; 35; A73G; T199C; T204C	16569	Asia_W_SW	I4a	I	I	35
156	JQ701958.1	JQ701958.1; U5a1a1d; Asia_W_Europe_C; 31; A73G; G185A; T204C	16570	Asia_W_Europe_C	U5a1a1d	U	U5	31
157	JQ701959.1	JQ701959.1; H106; Asia_W_Europe; 14; T146C; A263G; C315CC	16569	Asia_W_Europe	H106	H	H	14
158	JQ701960.1	JQ701960.1; H; Asia_W_Europe; 14; A73G; A263G; C309CCT	16570	Asia_W_Europe	H	H	H	14
159	JQ701961.1	JQ701961.1; J1c3j; Asia_W; 33; A73G; C150T; G185A	16569	Asia_W	J1c3j	J	J	33
160	JQ701962.1	JQ701962.1; K1a3a3; Asia_W; 35; A73G; A263G; C309CCT	16570	Asia_W	K1a3a3	K	K	35
161	JQ701963.1	JQ701963.1; H6a1a2b1; Asia_W_Europe; 20; T239C; A263G; C309CCT	16570	Asia_W_Europe	H6a1a2b1	H	H	20
162	JQ701964.1	JQ701964.1; K1a3a; Asia_W; 34; A73G; A263G; C315CC	16569	Asia_W	K1a3a	K	K	34
163	JQ701965.1	JQ701965.1; HV6; Asia_W; 12; A263G; C315CC; A750G	16569	Asia_W	HV6	HV	HV	12
164	JQ701966.1	JQ701966.1; H1b1b; Asia_W_Europe; 17; A263G; C309CCCT; T310C	16569	Asia_W_Europe	H1b1b	H	H	17

Supplementary Table S4 Human mitochondrial database (hMITO DB v1.0) metadata[a]

Row	Name (accession no.)	Description	Size	Geo_Region	Haplogroup	Macro_ Haplo	Macro_ Haplo2	Total Variants
165	JQ701967.1	JQ701967.1; H3g1; Asia_W_Europe; 16; T152C; A263G; C315CC	16569	Asia_W_Europe	H3g1	H	H	16
166	JQ701968.1	JQ701968.1; J1c2b3; Asia_W; 34; A73G; G185A; A188G	16570	Asia_W	J1c2b3	J	J	34
167	JQ701969.1	JQ701969.1; H1ai1; Asia_W_Europe; 12; A263G; C309CCCT; T310C	16571	Asia_W_Europe	H1ai1	H	H	12
168	JQ701970.1	JQ701970.1; U5a1b1g; Asia_W_Europe_C; 29; A73G; A263G; C315CC	16569	Asia_W_Europe_C	U5a1b1g	U	U5	29
169	JQ701971.1	JQ701971.1; V1a; Europe_S; 17; A263G; C315CC; A750G	16569	Europe_S	V1a	V	V	17
170	JQ701972.1	JQ701972.1; T2a1a8; Asia_W; 37; A73G; A263G; C309CCT	16570	Asia_W	T2a1a8	T	T	37
171	JQ701973.1	JQ701973.1; K1a4e; Asia_W; 36; A73G; A263G; C309CCT	16570	Asia_W	K1a4e	K	K	36
172	JQ701974.1	JQ701974.1; H5a7; Asia_W_Europe; 14; A263G; C315CC; C456T	16569	Asia_W_Europe	H5a7	H	H	14
173	JQ701975.1	JQ701975.1; H31; Asia_W_Europe; 13; T146C; T195C; A263G	16571	Asia_W_Europe	H31	H	H	13
174	JQ701976.1	JQ701976.1; A2al; Asia_NE_America_N; 38; C64T; A73G; T146C	16567	Asia_NE_America_N	A2al	A	A	38
175	JQ701977.1	JQ701977.1; H; Asia_W_Europe; 9; A263G; C315CC; A750G	16569	Asia_W_Europe	H	H	H	9
176	JQ701978.1	JQ701978.1; H1i2; Asia_W_Europe; 13; T152C; A263G; C315CC	16569	Asia_W_Europe	H1i2	H	H	13
177	JQ701979.1	JQ701979.1; H6a1b; Asia_W_Europe; 19; T239C; A263G; C309CCT	16570	Asia_W_Europe	H6a1b	H	H	19
178	JQ701980.1	JQ701980.1; H1e1a; Asia_W_Europe; 17; T204C; A263G; C315CC	16569	Asia_W_Europe	H1e1a	H	H	17
179	JQ701981.1	JQ701981.1; J2b1e; Asia_W; 35; A73G; C150T; T152C	16570	Asia_W	J2b1e	J	J	35
180	JQ701982.1	JQ701982.1; T2b13; Asia_W; 35; A73G; A263G; C309CCT	16570	Asia_W	T2b13	T	T	35
181	JQ701983.1	JQ701983.1; H3aj; Asia_W_Europe; 10; A263G; C315CC; A750G	16569	Asia_W_Europe	H3aj	H	H	10
182	JQ701984.1	JQ701984.1; H1c4b1; Asia_W_Europe; 13; A263G; C315CC; T477C	16569	Asia_W_Europe	H1c4b1	H	H	13
183	JQ701985.1	JQ701985.1; T2e5; Asia_W; 37; A73G; C150T; A200G	16570	Asia_W	T2e5	T	T	37
184	JQ701986.1	JQ701986.1; T2b; Asia_W; 35; A73G; A263G; C315CC	16569	Asia_W	T2b	T	T	35
185	JQ701987.1	JQ701987.1; H1e2c; Asia_W_Europe; 15; A73G; A263G; C315CC	16569	Asia_W_Europe	H1e2c	H	H	15
186	JQ701988.1	JQ701988.1; HV+16311; Asia_W_Europe; 16; A263G; C309CCT; T310C	16570	Asia_W_Europe	HV+16311	H	H	16
187	JQ701989.1	JQ701989.1; V12; Europe_S; 14; A263G; C309CCT; T310C	16570	Europe_S	V12	V	V	14
188	JQ701990.1	JQ701990.1; U4b1a2b; Asia_N_Europe_N; 39; A73G; T195C; A263G	16570	Asia_N_Europe_N	U4b1a2b	U	U4	39
189	JQ701991.1	JQ701991.1; X2i1; Asia_W_America_N; 29; A73G; A153G; T195C	16571	Asia_W_America_N	X2i1	X	X	29
190	JQ701992.1	JQ701992.1; J1c8b; Asia_W; 26; A73G; G185A; A263G	16569	Asia_W	J1c8b	J	J	26
191	JQ701993.1	JQ701993.1; H1c+152; Asia_W_Europe; 11; T152C; A263G; C315CC	16569	Asia_W_Europe	H1c+152	H	H	11
192	JQ701994.1	JQ701994.1; K1a4a1h; Asia_W; 41; A73G; T152C; A263G	16574	Asia_W	K1a4a1h	K	K	41
193	JQ701995.1	JQ701995.1; J1c3f; Asia_W; 30; A73G; A263G; C295T	16571	Asia_W	J1c3f	J	J	30
194	JQ701996.1	JQ701996.1; H77; Asia_W_Europe; 10; A263G; A291AA; C315CC	16570	Asia_W_Europe	H77	H	H	10
195	JQ701997.1	JQ701997.1; T2a1a2; Asia_W; 36; A73G; A263G; C315CC	16569	Asia_W	T2a1a2	T	T	36
196	JQ701998.1	JQ701998.1; H79; Asia_W_Europe; 13; A263G; C309CCCT; T310C	16574	Asia_W_Europe	H79	H	H	13
197	JQ701999.1	JQ701999.1; T2b4; Asia_W; 35; A73G; A263G; C309CCT	16570	Asia_W	T2b4	T	T	35
198	JQ702000.1	JQ702000.1; H6a1b3; Asia_W_Europe; 20; A93G; T204C; T239C	16571	Asia_W_Europe	H6a1b3	H	H	20
199	JQ702001.1	JQ702001.1; J1b2a; Asia_W; 34; A73G; A263G; C295T	16569	Asia_W	J1b2a	J	J	34
200	JQ702002.1	JQ702002.1; H1ah1; Asia_W_Europe; 17; A200G; A263G; C309CCT	16570	Asia_W_Europe	H1ah1	H	H	17
201	JQ702003.1	JQ702003.1; M33c; Asia_S; 37; A73G; A263G; C315CC	16569	Asia_S	M33c	M	M33	37
202	JQ702004.1	JQ702004.1; U2e2a1d; Asia_S_W_Europe; 44; A73G; T152C; T217C	16570	Asia_S_W_Europe	U2e2a1d	U	U2	44
203	JQ702005.1	JQ702005.1; H1c; Asia_W_Europe; 11; A263G; C315CC; T477C	16569	Asia_W_Europe	H1c	H	H	11
204	JQ702006.1	JQ702006.1; H1c5a; Asia_W_Europe; 14; T152C; A249d; A263G	16568	Asia_W_Europe	H1c5a	H	H	14
205	JQ702007.1	JQ702007.1; H1r; Asia_W_Europe; 15; T146C; T152C; A263G	16571	Asia_W_Europe	H1r	H	H	15

Supplementary Table S4 Human mitochondrial database (hMITO DB v1.0) metadata[a]

Row	Name (accession no.)	Description	Size	Geo_Region	Haplogroup	Macro_ Haplo	Macro_ Haplo2	Total Variants
206	JQ702008.1	JQ702008.1; H1a3; Asia_W_Europe; 12; A73G; T152C; A263G	16569	Asia_W_Europe	H1a3	H	H	12
207	JQ702009.1	JQ702009.1; H1n1b; Asia_W_Europe; 16; T146C; A263G; C315CC	16571	Asia_W_Europe	H1n1b	H	H	16
208	JQ702010.1	JQ702010.1; H10e3a; Asia_W_Europe; 15; T152C; A263G; C309CCT	16570	Asia_W_Europe	H10e3a	H	H	15
209	JQ702011.1	JQ702011.1; M1a8a; Africa_E_Nile Valley; 43; A73G; T195C; A263G	16571	Africa_E_Nile Valley	M1a8a	M	M1	43
210	JQ702012.1	JQ702012.1; T2b4; Asia_W; 34; A73G; A263G; C315CC	16569	Asia_W	T2b4	T	T	34
211	JQ702013.1	JQ702013.1; HV0+195; Asia_W; 17; C64T; T72C; T195C	16570	Asia_W	HV0+195	HV	HV	17
212	JQ702014.1	JQ702014.1; H56; Asia_W_Europe; 11; T152C; A263G; C309CCT	16570	Asia_W_Europe	H56	H	H	11
213	JQ702015.1	JQ702015.1; L2a1l2a1; Africa_W_C; 55; A73G; T146C; T152C	16570	Africa_W_C	L2a1l2a1	L2	L2	55
214	JQ702016.1	JQ702016.1; H1c1; Asia_W_Europe; 12; A263G; C315CC; T477C	16569	Asia_W_Europe	H1c1	H	H	12
215	JQ702017.1	JQ702017.1; J1c3; Asia_W; 30; A73G; G185A; T195C	16569	Asia_W	J1c3	J	J	30
216	JQ702018.1	JQ702018.1; J1c2c; Asia_W; 34; A73G; T146C; G185A	16570	Asia_W	J1c2c	J	J	34
217	JQ702019.1	JQ702019.1; H5g; Asia_W_Europe; 13; T146C; A263G; C309CCCT	16571	Asia_W_Europe	H5g	H	H	13
218	JQ702020.1	JQ702020.1; J1c1b2a; Asia_W; 35; A73G; G228A; A263G	16567	Asia_W	J1c1b2a	J	J	35
219	JQ702021.1	JQ702021.1; U5b2a1a1a; Asia_W_Europe_C; 31; A73G; C150T; A263G	16570	Asia_W_Europe_C	U5b2a1a1a	U	U5	31
220	JQ702022.1	JQ702022.1; H13a1a1e; Asia_W_Europe; 16; A263G; C309CCT; T310C	16568	Asia_W_Europe	H13a1a1e	H	H	16
221	JQ702023.1	JQ702023.1; I1a1c; Asia_W_SW; 43; A73G; A189G; T199C	16572	Asia_W_SW	I1a1c	I	I	43
222	JQ702024.1	JQ702024.1; H1e1a6; Asia_W_Europe; 15; C150T; A263G; C309CCT	16570	Asia_W_Europe	H1e1a6	H	H	15
223	JQ702025.1	JQ702025.1; V8; Europe_S; 17; T72C; A263G; C309CCT	16570	Europe_S	V8	V	V	17
224	JQ702026.1	JQ702026.1; V1a; Europe_S; 18; T72C; A263G; C309CCCT	16571	Europe_S	V1a	V	V	18
225	JQ702027.1	JQ702027.1; J2a1a1a2; Asia_W; 42; A73G; C150T; T152C	16571	Asia_W	J2a1a1a2	J	J	42
226	JQ702028.1	JQ702028.1; H1b1a; Asia_W_Europe; 17; A263G; C315CC; A374G	16568	Asia_W_Europe	H1b1a	H	H	17
227	JQ702029.1	JQ702029.1; H2a5b; Asia_W_Europe; 11; A263G; C309CCCT; T310C	16571	Asia_W_Europe	H2a5b	H	H	11
228	JQ702030.1	JQ702030.1; U5a1a2b1; Asia_W_Europe_C; 33; A73G; A263G; C309CCT	16570	Asia_W_Europe_C	U5a1a2b1	U	U5	33
229	JQ702031.1	JQ702031.1; K1a2a; Asia_W; 33; A73G; T152C; A263G	16569	Asia_W	K1a2a	K	K	33
230	JQ702032.1	JQ702032.1; H3+16311; Asia_W_Europe; 14; T152C; A263G; C309CCCT	16571	Asia_W_Europe	H3+16311	H	H	14
231	JQ702033.1	JQ702033.1; K2b1a1; Asia_W; 35; A73G; T146C; A263G	16569	Asia_W	K2b1a1	K	K	35
232	JQ702034.1	JQ702034.1; H1ao; Asia_W_Europe; 14; A93G; T146C; A263G	16569	Asia_W_Europe	H1ao	H	H	14
233	JQ702035.1	JQ702035.1; T1a1; Asia_W; 36; A73G; T152C; T195C	16570	Asia_W	T1a1	T	T	36
234	JQ702036.1	JQ702036.1; T2a1a6; Asia_W; 37; A73G; A263G; C309CCT	16570	Asia_W	T2a1a6	T	T	37
235	JQ702037.1	JQ702037.1; H26a1a; Asia_W_Europe; 14; A263G; C315CC; G709A	16569	Asia_W_Europe	H26a1a	H	H	14
236	JQ702038.1	JQ702038.1; U5a1a1; Asia_W_Europe_C; 27; A73G; A263G; C309CCT	16570	Asia_W_Europe_C	U5a1a1	U	U5	27
237	JQ702039.1	JQ702039.1; H1ai1; Asia_W_Europe; 13; A263G; C309CCT; T310C	16570	Asia_W_Europe	H1ai1	H	H	13
238	JQ702040.1	JQ702040.1; T1a4; Asia_W; 38; A73G; T152C; A263G	16570	Asia_W	T1a4	T	T	38
239	JQ702041.1	JQ702041.1; I3a; Asia_W_SW; 37; A73G; T152C; T199C	16574	Asia_W_SW	I3a	I	I	37
240	JQ702042.1	JQ702042.1; J2b1b1; Asia_W; 37; A73G; C150T; T152C	16570	Asia_W	J2b1b1	J	J	37
241	JQ702043.1	JQ702043.1; H1n4; Asia_W_Europe; 16; T146C; A263G; C309CCCT	16571	Asia_W_Europe	H1n4	H	H	16
242	JQ702044.1	JQ702044.1; H11a1; Asia_W_Europe; 17; T146C; T195C; A263G	16569	Asia_W_Europe	H11a1	H	H	17
243	JQ702045.1	JQ702045.1; V1a1b; Europe_S; 17; T72C; A263G; C315CC	16569	Europe_S	V1a1b	V	V	17
244	JQ702046.1	JQ702046.1; U5b2b3a1; Asia_W_Europe_C; 37; A73G; C150T; A263G	16569	Asia_W_Europe_C	U5b2b3a1	U	U5	37
245	JQ702047.1	JQ702047.1; L3k1; Africa_E; 41; A73G; C150T; T152C	16569	Africa_E	L3k1	L3	L3	41
246	JQ702048.1	JQ702048.1; HV11; Asia_W; 16; T146C; C150T; A263G	16570	Asia_W	HV11	HV	HV	16

Supplementary Table S4 Human mitochondrial database (hMITO DB v1.0) metadata[a]

Row	Name (accession no.)	Description	Size	Geo_Region	Haplogroup	Macro_ Haplo	Macro_ Haplo2	Total Variants
247	JQ702049.1	JQ702049.1; J1c8a1; Asia_W; 32; A73G; G185A; G228A	16574	Asia_W	J1c8a1	J	J	32
248	JQ702050.1	JQ702050.1; W1+119; Asia_W; 36; A73G; T119C; A189G	16569	Asia_W	W1+119	W	W	36
249	JQ702051.1	JQ702051.1; H11a2; Asia_W_Europe; 20; T146C; T195C; A263G	16569	Asia_W_Europe	H11a2	H	H	20
250	JQ702052.1	JQ702052.1; H47a; Asia_W_Europe; 14; T152C; A263G; C315CC	16567	Asia_W_Europe	H47a	H	H	14
251	JQ702053.1	JQ702053.1; U5a1a2b1; Asia_W_Europe_C; 33; A73G; A263G; C309CCT	16570	Asia_W_Europe_C	U5a1a2b1	U	U5	33
252	JQ702054.1	JQ702054.1; U5b1+16189+@16192; Asia_W_Europe_C; 30; A73G; C150T; A263	16570	Asia_W_Europe_C	U5b1+16189+@	U	U5	30
253	JQ702055.1	JQ702055.1; H3b+16129; Asia_W_Europe; 13; A263G; C309CCCT; T310C	16571	Asia_W_Europe	H3b+16129	H	H	13
254	JQ702056.1	JQ702056.1; H7b2; Asia_W_Europe; 11; A263G; C315CC; A750G	16569	Asia_W_Europe	H7b2	H	H	11
255	JQ702057.1	JQ702057.1; H1aa1; Asia_W_Europe; 12; A263G; C315CC; A750G	16569	Asia_W_Europe	H1aa1	H	H	12
256	JQ702058.1	JQ702058.1; T2b4+152; Asia_W; 38; A73G; T152C; A263G	16570	Asia_W	T2b4+152	T	T	38
257	JQ702059.1	JQ702059.1; H2a1a; Asia_W_Europe; 14; G94A; T152C; A263G	16568	Asia_W_Europe	H2a1a	H	H	14
258	JQ702060.1	JQ702060.1; H41a; Asia_W_Europe; 14; C262T; A263G; C309CCT	16570	Asia_W_Europe	H41a	H	H	14
259	JQ702061.1	JQ702061.1; W5a1a; Asia_W; 42; A73G; T152C; A189G	16570	Asia_W	W5a1a	W	W	42
260	JQ702062.1	JQ702062.1; I3a; Asia_W_SW; 39; A73G; T152C; A189G	16576	Asia_W_SW	I3a	I	I	39
261	JQ702063.1	JQ702063.1; J1c3+189; Asia_W; 30; A73G; G185A; A189G	16570	Asia_W	J1c3+189	J	J	30
262	JQ702064.1	JQ702064.1; B5a1b1; Asia_SE_E_America_N_S; 35; A73G; A210G; A263G	16560	Asia_SE_E_America_N_S	B5a1b1	B	B	35
263	JQ702065.1	JQ702065.1; X2b+226; Asia_W_America_N; 33; A73G; T195C; G225A	16570	Asia_W_America_N	X2b+226	X	X	33
264	JQ702066.1	JQ702066.1; K1a9; Asia_W; 33; A73G; T195C; A263G	16569	Asia_W	K1a9	K	K	33
265	JQ702067.1	JQ702067.1; H; Asia_W_Europe; 11; A73G; G207A; A263G	16570	Asia_W_Europe	H	H	H	11
266	JQ702068.1	JQ702068.1; J1c8a1a; Asia_W; 35; A73G; G185A; G228A	16570	Asia_W	J1c8a1a	J	J	35
267	JQ702069.1	JQ702069.1; M7a1a9; Asia_E_SE; 45; A73G; T146C; A263G	16568	Asia_E_SE	M7a1a9	M	M7	45
268	JQ702070.1	JQ702070.1; K1a+195; Asia_W; 35; A73G; T195C; A263G	16573	Asia_W	K1a+195	K	K	35
269	JQ702071.1	JQ702071.1; H1ae1; Asia_W_Europe; 11; A263G; C315CC; A750G	16569	Asia_W_Europe	H1ae1	H	H	11
270	JQ702072.1	JQ702072.1; U5a2a1; Asia_W_Europe_C; 28; A73G; A263G; C309CCT	16570	Asia_W_Europe_C	U5a2a1	U	U5	28
271	JQ702073.1	JQ702073.1; H56; Asia_W_Europe; 9; A263G; C315CC; A750G	16569	Asia_W_Europe	H56	H	H	9
272	JQ702074.1	JQ702074.1; F1a3a; Asia_SE_E; 37; A73G; A249d; A263G	16566	Asia_SE_E	F1a3a	F	F	37
273	JQ702075.1	JQ702075.1; K2b1a4; Asia_W; 38; A73G; A93G; T146C	16569	Asia_W	K2b1a4	K	K	38
274	JQ702076.1	JQ702076.1; K1a4d; Asia_W; 35; A73G; A263G; C315CC	16571	Asia_W	K1a4d	K	K	35
275	JQ702077.1	JQ702077.1; H1n1b; Asia_W_Europe; 17; T146C; A263G; C309CCT	16574	Asia_W_Europe	H1n1b	H	H	17
276	JQ702078.1	JQ702078.1; U5a1a1; Asia_W_Europe_C; 30; A73G; G207A; A263G	16570	Asia_W_Europe_C	U5a1a1	U	U5	30
277	JQ702079.1	JQ702079.1; C1b11; Asia_NE_America_N_S; 48; T72C; A73G; C194T	16565	Asia_NE_America_N_S	C1b11	C	C	48
278	JQ702080.1	JQ702080.1; H82; Asia_W_Europe; 11; T195C; A263G; C315CC	16567	Asia_W_Europe	H82	H	H	11
279	JQ702081.1	JQ702081.1; J1c1b2; Asia_W; 35; A73G; G228A; A263G	16567	Asia_W	J1c1b2	J	J	35
280	JQ702082.1	JQ702082.1; A2; Asia_NE_America_N; 32; A73G; T146C; A153G	16569	Asia_NE_America_N	A2	A	A	32
281	JQ702083.1	JQ702083.1; H5a4a1a; Asia_W_Europe; 18; A263G; T282C; C315CC	16569	Asia_W_Europe	H5a4a1a	H	H	18
282	JQ702084.1	JQ702084.1; T2b13a; Asia_W; 38; A73G; A263G; C309CCT	16570	Asia_W	T2b13a	T	T	38
283	JQ702085.1	JQ702085.1; U5a1a1a; Asia_W_Europe_C; 31; A73G; T152C; A263G	16570	Asia_W_Europe_C	U5a1a1a	U	U5	31
284	JQ702086.1	JQ702086.1; K1a1c; Asia_W; 36; A73G; C114T; A263G	16569	Asia_W	K1a1c	K	K	36
285	JQ702087.1	JQ702087.1; H3+152; Asia_W_Europe; 10; T152C; A263G; C315CC	16569	Asia_W_Europe	H3+152	H	H	10
286	JQ702088.1	JQ702088.1; J1c12b; Asia_W; 35; A73G; G185A; A189G	16572	Asia_W	J1c12b	J	J	35
287	JQ702089.1	JQ702089.1; H1c2; Asia_W_Europe; 14; T195C; A263G; C315CC	16569	Asia_W_Europe	H1c2	H	H	14

Supplementary Table S4 Human mitochondrial database (hMITO DB v1.0) metadata[a]

Row	Name (accession no.)	Description	Size	Geo_Region	Haplogroup	Macro_ Haplo	Macro_ Haplo2	Total Variants
288	JQ702090.1	JQ702090.1; H6a1b2; Asia_W_Europe; 17; T239C; A263G; C309CCT	16570	Asia_W_Europe	H6a1b2	H	H	17
289	JQ702091.1	JQ702091.1; J2a1a1c; Asia_W; 41; A73G; C150T; T152C	16570	Asia_W	J2a1a1c	J	J	41
290	JQ702092.1	JQ702092.1; H3m; Asia_W_Europe; 11; A263G; C315CC; A750G	16569	Asia_W_Europe	H3m	H	H	11
291	JQ702093.1	JQ702093.1; A2v1+152; Asia_NE_America_N; 38; C64T; A73G; T146C	16569	Asia_NE_America_N	A2v1+152	A	A	38
292	JQ702094.1	JQ702094.1; U4a2a1; Asia_N_Europe_N; 29; A73G; T195C; A263G	16570	Asia_N_Europe_N	U4a2a1	U	U4	29
293	JQ702095.1	JQ702095.1; H3ap; Asia_W_Europe; 15; T146C; A263G; C309CCT	16570	Asia_W_Europe	H3ap	H	H	15
294	JQ702096.1	JQ702096.1; X2b9; Asia_W_America_N; 32; A73G; A153G; T195C	16570	Asia_W_America_N	X2b9	X	X	32
295	JQ702097.1	JQ702097.1; J1c3e1; Asia_W; 35; A73G; G185A; G228A	16569	Asia_W	J1c3e1	J	J	35
296	JQ702098.1	JQ702098.1; W5a1a; Asia_W; 39; A73G; A189G; C194T	16570	Asia_W	W5a1a	W	W	39
297	JQ702099.1	JQ702099.1; H1c1b; Asia_W_Europe; 16; A263G; C309CCT; T310C	16570	Asia_W_Europe	H1c1b	H	H	16
298	JQ702100.1	JQ702100.1; K1a9; Asia_W; 32; A73G; T195C; A263G	16569	Asia_W	K1a9	K	K	32
299	JQ702101.1	JQ702101.1; H1a3a; Asia_W_Europe; 15; A73G; A263G; C309CCT	16570	Asia_W_Europe	H1a3a	H	H	15
300	JQ702102.1	JQ702102.1; H1; Asia_W_Europe; 10; A263G; C315CC; A750G	16569	Asia_W_Europe	H1	H	H	10
301	JQ702103.1	JQ702103.1; H16c; Asia_W_Europe; 14; T152C; T195C; A263G	16571	Asia_W_Europe	H16c	H	H	14
302	JQ702104.1	JQ702104.1; H1e1b1b; Asia_W_Europe; 17; T152C; A263G; C315CC	16569	Asia_W_Europe	H1e1b1b	H	H	17
303	JQ702105.1	JQ702105.1; H1ae3a; Asia_W_Europe; 15; A263G; C309CCT; T310C	16570	Asia_W_Europe	H1ae3a	H	H	15
304	JQ702106.1	JQ702106.1; U2e1a1; Asia_S_W_Europe; 42; A73G; T152C; T217C	16572	Asia_S_W_Europe	U2e1a1	U	U2	42
305	JQ702107.1	JQ702107.1; H6a1a2a; Asia_W_Europe; 17; C41T; T239C; A263G	16569	Asia_W_Europe	H6a1a2a	H	H	17
306	JQ702108.1	JQ702108.1; T2g2a; Asia_W; 40; A73G; T195C; A263G	16570	Asia_W	T2g2a	T	T	40
307	JQ702109.1	JQ702109.1; I3a; Asia_W_SW; 38; A73G; T152C; T199C	16582	Asia_W_SW	I3a	I	I	38
308	JQ702110.1	JQ702110.1; J1c2; Asia_W; 32; A73G; G185A; A188G	16570	Asia_W	J1c2	J	J	32
309	JQ702111.1	JQ702111.1; K2a; Asia_W; 31; A73G; T146C; T152C	16569	Asia_W	K2a	K	K	31
310	JQ702112.1	JQ702112.1; H13a1a; Asia_W_Europe; 14; A263G; C309CCT; T310C	16570	Asia_W_Europe	H13a1a	H	H	14
311	JQ702113.1	JQ702113.1; H1c1; Asia_W_Europe; 14; A263G; C315CC; T477C	16569	Asia_W_Europe	H1c1	H	H	14
312	JQ702114.1	JQ702114.1; H1b1; Asia_W_Europe; 15; A263G; C309CCCT; T310C	16571	Asia_W_Europe	H1b1	H	H	15
313	JQ702115.1	JQ702115.1; L2c4; Africa_W_C; 64; A73G; T89C; A93G	16567	Africa_W_C	L2c4	L2	L2	64
314	JQ702116.1	JQ702116.1; HV0d; Asia_W; 17; T72C; T195C; A263G	16570	Asia_W	HV0d	HV	HV	17
315	JQ702117.1	JQ702117.1; K1a9; Asia_W; 32; A73G; T195C; A263G	16569	Asia_W	K1a9	K	K	32
316	JQ702118.1	JQ702118.1; U6a7c; Asia_W_Europe_C; 36; A73G; C151T; T152C	16570	Asia_W_Europe_C	U6a7c	U	U6	36
317	JQ702119.1	JQ702119.1; H17a; Asia_W_Europe; 14; A263G; C309CCCT; T310C	16571	Asia_W_Europe	H17a	H	H	14
318	JQ702120.1	JQ702120.1; H3u1; Asia_W_Europe; 14; A263G; C315CC; A750G	16570	Asia_W_Europe	H3u1	H	H	14
319	JQ702121.1	JQ702121.1; H8c; Asia_W_Europe; 22; A93G; C114T; T146C	16571	Asia_W_Europe	H8c	H	H	22
320	JQ702122.1	JQ702122.1; U5a1a1h; Asia_W_Europe_C; 29; A73G; T152C; T195C	16569	Asia_W_Europe_C	U5a1a1h	U	U5	29
321	JQ702123.1	JQ702123.1; L2b3a; Africa_W_C; 70; A73G; T146C; C150T	16570	Africa_W_C	L2b3a	L2	L2	70
322	JQ702124.1	JQ702124.1; H3i; Asia_W_Europe; 12; T152C; A263G; C315CC	16569	Asia_W_Europe	H3i	H	H	12
323	JQ702125.1	JQ702125.1; U4a1e; Asia_W_Europe_C; 35; A73G; T152C; T195C	16571	Asia_W_Europe_C	U4a1e	U	U4	35
324	JQ702126.1	JQ702126.1; M7b1a1+(16192); Asia_E_SE; 48; A73G; T146C; C150T	16570	Asia_E_SE	M7b1a1+(1619	M	M7	48
325	JQ702127.1	JQ702127.1; H3ac; Asia_W_Europe; 10; A263G; C315CC; A750G	16569	Asia_W_Europe	H3ac	H	H	10
326	JQ702128.1	JQ702128.1; H5a1g1a; Asia_W_Europe; 20; A263G; C315CC; A444G	16567	Asia_W_Europe	H5a1g1a	H	H	20
327	JQ702129.1	JQ702129.1; K1c2; Asia_W; 36; A73G; T146C; T152C	16568	Asia_W	K1c2	K	K	36
328	JQ702130.1	JQ702130.1; H1e1a; Asia_W_Europe; 13; A263G; C315CC; A750G	16569	Asia_W_Europe	H1e1a	H	H	13

Supplementary Table S4 Human mitochondrial database (hMITO DB v1.0) metadata[a]

Row	Name (accession no.)	Description	Size	Geo_Region	Haplogroup	Macro_ Haplo	Macro_ Haplo2	Total Variants
329	JQ702131.1	JQ702131.1; K2b1a1a; Asia_W; 37; A73G; T146C; T152C	16569	Asia_W	K2b1a1a	K	K	37
330	JQ702132.1	JQ702132.1; H1+152; Asia_W_Europe; 13; T152C; A263G; C309CCT	16570	Asia_W_Europe	H1+152	H	H	13
331	JQ702133.1	JQ702133.1; K1c1; Asia_W; 34; A73G; T146C; T152C	16570	Asia_W	K1c1	K	K	34
332	JQ702134.1	JQ702134.1; K2a10; Asia_W; 34; A73G; T146C; T152C	16570	Asia_W	K2a10	K	K	34
333	JQ702135.1	JQ702135.1; H3h7; Asia_W_Europe; 13; A93G; A263G; C315CC	16569	Asia_W_Europe	H3h7	H	H	13
334	JQ702136.1	JQ702136.1; U5b2b3b; Asia_W_Europe_C; 35; A73G; C150T; A263G	16570	Asia_W_Europe_C	U5b2b3b	U	U5	35
335	JQ702137.1	JQ702137.1; D1f2; Asia_E_America_N_S; 40; A73G; T152C; A263G	16570	Asia_E_America_N_S	D1f2	D	D	40
336	JQ702138.1	JQ702138.1; H+152; Asia_W_Europe; 13; A73G; T146C; T152C	16567	Asia_W_Europe	H+152	H	H	13
337	JQ702139.1	JQ702139.1; T1a6; Asia_W; 37; A73G; A263G; C309CCCT	16570	Asia_W	T1a6	T	T	37
338	JQ702140.1	JQ702140.1; H1h1; Asia_W_Europe; 18; T146C; T152C; A234G	16571	Asia_W_Europe	H1h1	H	H	18
339	JQ702141.1	JQ702141.1; U8a2; Asia_W_Europe ; 29; A73G; A263G; T282C	16570	Asia_W_Europe	U8a2	U	U8	29
340	JQ702142.1	JQ702142.1; A2q; Asia_NE_America_N; 34; C64T; A73G; T146C	16568	Asia_NE_America_N	A2q	A	A	34
341	JQ702143.1	JQ702143.1; HV5a; Asia_W; 11; A263G; C315CC; A750G	16569	Asia_W	HV5a	HV	HV	11
342	JQ702144.1	JQ702144.1; U5a2d; Asia_W_Europe_C; 36; A73G; A200G; A263G	16571	Asia_W_Europe_C	U5a2d	U	U5	36
343	JQ702145.1	JQ702145.1; V9a2; Europe_S; 22; T72C; T204C; G207A	16570	Europe_S	V9a2	V	V	22
344	JQ702146.1	JQ702146.1; U5b3b1; Asia_W_Europe_C; 29; A73G; C150T; A189G	16569	Asia_W_Europe_C	U5b3b1	U	U5	29
345	JQ702147.1	JQ702147.1; W7; Asia_W; 36; A73G; T119C; G185A	16570	Asia_W	W7	W	W	36
346	JQ702148.1	JQ702148.1; K1c1; Asia_W; 35; A73G; T146C; T152C	16568	Asia_W	K1c1	K	K	35
347	JQ702149.1	JQ702149.1; HV0a1a; Asia_W; 21; T72C; A200G; A263G	16568	Asia_W	HV0a1a	HV	HV	21
348	JQ702150.1	JQ702150.1; U3b2; Africa_NE_Asia_W; 32; A73G; C150T; T208C	16567	Africa_NE_Asia_W	U3b2	U	U3	32
349	JQ702151.1	JQ702151.1; H5a2; Asia_W_Europe; 13; A263G; C309CCT; T310C	16570	Asia_W_Europe	H5a2	H	H	13
350	JQ702152.1	JQ702152.1; U5b1b2; Asia_W_Europe_C; 29; A73G; C150T; T217C	16570	Asia_W_Europe_C	U5b1b2	U	U5	29
351	JQ702153.1	JQ702153.1; D5c1a; Asia_E_America_N_S; 44; A73G; T146C; C150T	16571	Asia_E_America_N_S	D5c1a	D	D	44
352	JQ702154.1	JQ702154.1; U4d1a1; Asia_W_Europe_C; 34; A73G; T195C; A263G	16573	Asia_W_Europe_C	U4d1a1	U	U4	34
353	JQ702155.1	JQ702155.1; K1a1b1a; Asia_W; 37; A73G; A263G; C315CC	16571	Asia_W	K1a1b1a	K	K	37
354	JQ702156.1	JQ702156.1; H5+16192; Asia_W_Europe; 16; A263G; C315CC; C456T	16569	Asia_W_Europe	H5+16192	H	H	16
355	JQ702157.1	JQ702157.1; H14a2; Asia_W_Europe; 21; A95C; T152C; A263G	16570	Asia_W_Europe	H14a2	H	H	21
356	JQ702158.1	JQ702158.1; T2b4d; Asia_W; 35; A73G; T152C; A263G	16569	Asia_W	T2b4d	T	T	35
357	JQ702159.1	JQ702159.1; V15a; Europe_S; 16; T72C; A263G; C309CCCT	16571	Europe_S	V15a	V	V	16
358	JQ702160.1	JQ702160.1; U5a1f1; Asia_W_Europe_C; 32; A73G; T195C; A263G	16569	Asia_W_Europe_C	U5a1f1	U	U5	32
359	JQ702161.1	JQ702161.1; H1n2; Asia_W_Europe; 19; T146C; T152C; A200G	16571	Asia_W_Europe	H1n2	H	H	19
360	JQ702162.1	JQ702162.1; H3a1; Asia_W_Europe; 14; T152C; A263G; C309CCT	16570	Asia_W_Europe	H3a1	H	H	14
361	JQ702163.1	JQ702163.1; I2c; Asia_W_SW; 36; A73G; T199C; T204C	16571	Asia_W_SW	I2c	I	I	36
362	JQ702164.1	JQ702164.1; G1b+16129; Asia_E; 38; A73G; G207A; A263G	16569	Asia_E	G1b+16129	G	G	38
363	JQ702165.1	JQ702165.1; U5a2c1; Asia_W_Europe_C; 28; A73G; A263G; C309CCT	16568	Asia_W_Europe_C	U5a2c1	U	U5	28
364	JQ702166.1	JQ702166.1; K1a1a; Asia_W; 32; A73G; C114T; A263G	16569	Asia_W	K1a1a	K	K	32
365	JQ702167.1	JQ702167.1; U5b2a2a2; Asia_W_Europe_C; 34; A73G; C150T; A263G	16569	Asia_W_Europe_C	U5b2a2a2	U	U5	34
366	JQ702168.1	JQ702168.1; K1b1a1a; Asia_W; 41; A73G; T152C; T199C	16569	Asia_W	K1b1a1a	K	K	41
367	JQ702169.1	JQ702169.1; L2c; Africa_W_C; 59; A73G; A93G; C150T	16568	Africa_W_C	L2c	L2	L2	59
368	JQ702170.1	JQ702170.1; T2h2; Asia_W; 37; A73G; A263G; C315CC	16569	Asia_W	T2h2	T	T	37
369	JQ702171.1	JQ702171.1; H2a5b; Asia_W_Europe; 11; A263G; C309CCT; T310C	16570	Asia_W_Europe	H2a5b	H	H	11

Supplementary Table S4 Human mitochondrial database (hMITO DB v1.0) metadata[a]

Row	Name (accession no.)	Description	Size	Geo_Region	Haplogroup	Macro_ Haplo	Macro_ Haplo2	Total Variants
370	JQ702172.1	JQ702172.1; A2k1a; Asia_NE_America_N; 35; C64T; A73G; T146C	16568	Asia_NE_America_N	A2k1a	A	A	35
371	JQ702173.1	JQ702173.1; T1a1q; Asia_W; 37; A73G; T152C; T195C	16570	Asia_W	T1a1q	T	T	37
372	JQ702174.1	JQ702174.1; H1a3; Asia_W_Europe; 13; A73G; A263G; C315CC	16569	Asia_W_Europe	H1a3	H	H	13
373	JQ702175.1	JQ702175.1; K1a4a1a; Asia_W; 40; A73G; T131C; A263G	16573	Asia_W	K1a4a1a	K	K	40
374	JQ702176.1	JQ702176.1; J1c14; Asia_W; 36; A73G; G185A; T195C	16572	Asia_W	J1c14	J	J	36
375	JQ702177.1	JQ702177.1; A2ao1; Asia_NE_America_N; 37; C64T; A73G; T146C	16568	Asia_NE_America_N	A2ao1	A	A	37
376	JQ702178.1	JQ702178.1; H1ba; Asia_W_Europe; 11; A263G; C315CC; T482C	16569	Asia_W_Europe	H1ba	H	H	11
377	JQ702179.1	JQ702179.1; L3e3b; Africa_E; 41; A73G; C150T; A189G	16567	Africa_E	L3e3b	L3	L3	41
378	JQ702180.1	JQ702180.1; H4a1a4b; Asia_W_Europe; 22; T195C; A263G; C309CCCT	16569	Asia_W_Europe	H4a1a4b	H	H	22
379	JQ702181.1	JQ702181.1; R0a2a1; Africa_NE_Asia_W; 22; T57TC; C64T; A263G	16570	Africa_NE_Asia_W	R0a2a1	R	R0	22
380	JQ702182.1	JQ702182.1; H1a3; Asia_W_Europe; 14; A73G; A263G; C315CC	16569	Asia_W_Europe	H1a3	H	H	14
381	JQ702183.1	JQ702183.1; H3m; Asia_W_Europe; 12; A263G; C315CC; A750G	16569	Asia_W_Europe	H3m	H	H	12
382	JQ702184.1	JQ702184.1; U5a2b2a; Asia_W_Europe_C; 31; A73G; C182T; A263G	16570	Asia_W_Europe_C	U5a2b2a	U	U5	31
383	JQ702185.1	JQ702185.1; C1b; Asia_NE_America_N_S; 47; A73G; G143A; T195C	16564	Asia_NE_America_N_S	C1b	C	C	47
384	JQ702186.1	JQ702186.1; H1c1; Asia_W_Europe; 14; A263G; C315CC; T477C	16569	Asia_W_Europe	H1c1	H	H	14
385	JQ702187.1	JQ702187.1; H1a1; Asia_W_Europe; 15; A73G; A263G; C315CC	16569	Asia_W_Europe	H1a1	H	H	15
386	JQ702188.1	JQ702188.1; T2b+152; Asia_W; 35; A73G; T152C; A263G	16569	Asia_W	T2b+152	T	T	35
387	JQ702189.1	JQ702189.1; H6a1a6; Asia_W_Europe; 18; T239C; A263G; A288G	16570	Asia_W_Europe	H6a1a6	H	H	18
388	JQ702190.1	JQ702190.1; U5b2a1a2; Asia_W_Europe_C; 32; A73G; C150T; A263G	16572	Asia_W_Europe_C	U5b2a1a2	U	U5	32
389	JQ702191.1	JQ702191.1; I2; Asia_W_SW; 38; A73G; T152C; T199C	16571	Asia_W_SW	I2	I	I	38
390	JQ702192.1	JQ702192.1; K1a9; Asia_W; 33; A73G; T195C; A263G	16569	Asia_W	K1a9	K	K	33
391	JQ702193.1	JQ702193.1; K1a13; Asia_W; 39; A73G; T146C; T152C	16572	Asia_W	K1a13	K	K	39
392	JQ702194.1	JQ702194.1; HV0d; Asia_W; 17; T72C; T195C; A263G	16571	Asia_W	HV0d	HV	HV	17
393	JQ702195.1	JQ702195.1; U5a1b1a; Asia_W_Europe_C; 29; A73G; A263G; C315CC	16569	Asia_W_Europe_C	U5a1b1a	U	U5	29
394	JQ702196.1	JQ702196.1; J2b1a; Asia_W; 32; A73G; C150T; T152C	16569	Asia_W	J2b1a	J	J	32
395	JQ702197.1	JQ702197.1; N9a2a; Africa_NE_Asia_E; 27; A73G; C150T; A263G	16571	Africa_NE_Asia_E	N9a2a	N	N9	27
396	JQ702198.1	JQ702198.1; H1a1b; Asia_W_Europe; 15; A73G; A263G; C315CC	16569	Asia_W_Europe	H1a1b	H	H	15
397	JQ702199.1	JQ702199.1; J2b1a; Asia_W; 38; A73G; C150T; T152C	16569	Asia_W	J2b1a	J	J	38
398	JQ702200.1	JQ702200.1; A2+(64)+@153; Asia_NE_America_N; 34; C64T; A73G; A111C	16568	Asia_NE_America_N	A2+(64)+@153	A	A	34
399	JQ702201.1	JQ702201.1; J1c5d; Asia_W; 34; A73G; G185A; A188G	16568	Asia_W	J1c5d	J	J	34
400	JQ702202.1	JQ702202.1; H26a1; Asia_W_Europe; 14; T152C; A263G; C315CC	16570	Asia_W_Europe	H26a1	H	H	14
401	JQ702203.1	JQ702203.1; U8a1a1b1; Asia_W_Europe ; 32; A73G; A263G; T282C	16570	Asia_W_Europe	U8a1a1b1	U	U8	32
402	JQ702204.1	JQ702204.1; H1c; Asia_W_Europe; 11; T146C; A263G; C315CC	16569	Asia_W_Europe	H1c	H	H	11
403	JQ702205.1	JQ702205.1; H2a5b1; Asia_W_Europe; 11; A249d; A263G; C309CCT	16569	Asia_W_Europe	H2a5b1	H	H	11
404	JQ702206.1	JQ702206.1; V14; Europe_S; 15; T72C; A263G; C309CCT	16570	Europe_S	V14	V	V	15
405	JQ702207.1	JQ702207.1; D4a1f1; Asia_E_America_N_S; 41; A73G; T152C; A263G	16570	Asia_E_America_N_S	D4a1f1	D	D	41
406	JQ702208.1	JQ702208.1; J1c12b; Asia_W; 35; A73G; G185A; A189G	16574	Asia_W	J1c12b	J	J	35
407	JQ702209.1	JQ702209.1; U5b2b4a; Asia_W_Europe_C; 35; A73G; C150T; T152C	16570	Asia_W_Europe_C	U5b2b4a	U	U5	35
408	JQ702210.1	JQ702210.1; T2e+152; Asia_W; 39; A73G; C150T; T152C	16570	Asia_W	T2e+152	T	T	39
409	JQ702211.1	JQ702211.1; U4b; Asia_W_Europe_C; 29; A73G; T195C; A263G	16571	Asia_W_Europe_C	U4b	U	U4	29
410	JQ702212.1	JQ702212.1; H6a1a; Asia_W_Europe; 18; T239C; A263G; C309CCCT	16571	Asia_W_Europe	H6a1a	H	H	18

Supplementary Table S4 Human mitochondrial database (hMITO DB v1.0) metadata[a]

Row	Name (accession no.)	Description	Size	Geo_Region	Haplogroup	Macro_ Haplo	Macro_ Haplo2	Total Variants
411	JQ702213.1	JQ702213.1; K1a4a1a+195; Asia_W; 36; A73G; T195C; A263G	16569	Asia_W	K1a4a1a+195	K	K	36
412	JQ702214.1	JQ702214.1; H6a1a; Asia_W_Europe; 16; T239C; A263G; C315CC	16571	Asia_W_Europe	H6a1a	H	H	16
413	JQ702215.1	JQ702215.1; H1c+152; Asia_W_Europe; 13; T152C; A263G; C309CCCT	16571	Asia_W_Europe	H1c+152	H	H	13
414	JQ702216.1	JQ702216.1; J2a1a1; Asia_W; 38; A73G; C150T; T152C	16570	Asia_W	J2a1a1	J	J	38
415	JQ702217.1	JQ702217.1; H26; Asia_W_Europe; 11; A263G; C309CCCT; T310C	16571	Asia_W_Europe	H26	H	H	11
416	JQ702218.1	JQ702218.1; H79; Asia_W_Europe; 12; A263G; C309CCCT; T310C	16571	Asia_W_Europe	H79	H	H	12
417	JQ702219.1	JQ702219.1; J1c2; Asia_W; 31; A73G; G185A; A188G	16570	Asia_W	J1c2	J	J	31
418	JQ702220.1	JQ702220.1; H3t; Asia_W_Europe; 12; A263G; C315CC; A750G	16569	Asia_W_Europe	H3t	H	H	12
419	JQ702221.1	JQ702221.1; H1c; Asia_W_Europe; 13; A263G; C315CC; T477C	16567	Asia_W_Europe	H1c	H	H	13
420	JQ702222.1	JQ702222.1; HV0d; Asia_W; 17; C64T; T72C; T195C	16570	Asia_W	HV0d	HV	HV	17
421	JQ702223.1	JQ702223.1; H1i2a; Asia_W_Europe; 14; T152C; A263G; C315CC	16569	Asia_W_Europe	H1i2a	H	H	14
422	JQ702224.1	JQ702224.1; J1c1b; Asia_W; 32; A73G; G185A; A263G	16569	Asia_W	J1c1b	J	J	32
423	JQ702225.1	JQ702225.1; U5a1f1a1; Asia_W_Europe_C; 37; A73G; A189G; T199C	16574	Asia_W_Europe_C	U5a1f1a1	U	U5	37
424	JQ702226.1	JQ702226.1; H1a1; Asia_W_Europe; 13; A73G; A263G; C315CC	16569	Asia_W_Europe	H1a1	H	H	13
425	JQ702227.1	JQ702227.1; L0a1a2; Africa_S_SE; 82; C64T; A93G; G185A	16567	Africa_S_SE	L0a1a2	L0	L0	82
426	JQ702228.1	JQ702228.1; J1c8a; Asia_W; 29; A73G; G185A; G228A	16569	Asia_W	J1c8a	J	J	29
427	JQ702229.1	JQ702229.1; H33; Asia_W_Europe; 10; A263G; C315CC; A750G	16569	Asia_W_Europe	H33	H	H	10
428	JQ702230.1	JQ702230.1; U5a2b3a1; Asia_W_Europe_C; 34; A73G; T152C; A263G	16571	Asia_W_Europe_C	U5a2b3a1	U	U5	34
429	JQ702231.1	JQ702231.1; U5a1e; Asia_W_Europe_C; 27; A73G; A263G; C315CC	16569	Asia_W_Europe_C	U5a1e	U	U5	27
430	JQ702232.1	JQ702232.1; M74b2; Asia_S; 42; A73G; A263G; C309CCT	16570	Asia_S	M74b2	M	M74	42
431	JQ702233.1	JQ702233.1; H5a1j; Asia_W_Europe; 17; A257G; A263G; C309CCT	16568	Asia_W_Europe	H5a1j	H	H	17
432	JQ702234.1	JQ702234.1; H1a3b; Asia_W_Europe; 17; A73G; T195C; A263G	16568	Asia_W_Europe	H1a3b	H	H	17
433	JQ702235.1	JQ702235.1; U5b2a1b; Asia_W_Europe_C; 30; A73G; C150T; T152C	16569	Asia_W_Europe_C	U5b2a1b	U	U5	30
434	JQ702236.1	JQ702236.1; H1r; Asia_W_Europe; 13; T152C; A263G; C309CCCT	16571	Asia_W_Europe	H1r	H	H	13
435	JQ702237.1	JQ702237.1; H1c1b; Asia_W_Europe; 16; A263G; C309CCT; T310C	16570	Asia_W_Europe	H1c1b	H	H	16
436	JQ702238.1	JQ702238.1; K1a4a1a2b; Asia_W; 40; A73G; T146C; A263G	16572	Asia_W	K1a4a1a2b	K	K	40
437	JQ702239.1	JQ702239.1; T2b; Asia_W; 37; A73G; A237G; A263G	16569	Asia_W	T2b	T	T	37
438	JQ702240.1	JQ702240.1; H27a; Asia_W_Europe; 12; A263G; C315CC; A750G	16569	Asia_W_Europe	H27a	H	H	12
439	JQ702241.1	JQ702241.1; L3b1a10; Africa_E; 39; A73G; A263G; C315CC	16566	Africa_E	L3b1a10	L3	L3	39
440	JQ702242.1	JQ702242.1; H53; Asia_W_Europe; 10; A263G; C315CC; A750G	16569	Asia_W_Europe	H53	H	H	10
441	JQ702243.1	JQ702243.1; J1c7a; Asia_W; 35; A73G; G185A; G228A	16570	Asia_W	J1c7a	J	J	35
442	JQ702244.1	JQ702244.1; U5a2a1; Asia_W_Europe_C; 29; A73G; C150T; A263G	16569	Asia_W_Europe_C	U5a2a1	U	U5	29
443	JQ702245.1	JQ702245.1; K1a1b1a; Asia_W; 37; A73G; C114T; A263G	16569	Asia_W	K1a1b1a	K	K	37
444	JQ702246.1	JQ702246.1; G2a2; Asia_E; 43; A73G; T152C; A263G	16568	Asia_E	G2a2	G	G	43
445	JQ702247.1	JQ702247.1; M59; Asia_S; 42; A73G; A249G; A263G	16568	Asia_S	M59	M	M59	42
446	JQ702248.1	JQ702248.1; X1c; Asia_W_America_N; 32; A73G; T146C; A153G	16567	Asia_W_America_N	X1c	X	X	32
447	JQ702249.1	JQ702249.1; W1g; Asia_W; 34; A73G; A189G; T195C	16570	Asia_W	W1g	W	W	34
448	JQ702250.1	JQ702250.1; U2e2a1a; Asia_S_W_Europe; 40; A73G; T152C; T217C	16569	Asia_S_W_Europe	U2e2a1a	U	U2	40
449	JQ702251.1	JQ702251.1; A2f2; Asia_NE_America_N; 37; C64T; A73G; A153G	16568	Asia_NE_America_N	A2f2	A	A	37
450	JQ702252.1	JQ702252.1; H; Asia_W_Europe; 14; A73G; A263G; C309CCT	16570	Asia_W_Europe	H	H	H	14
451	JQ702253.1	JQ702253.1; I2c; Asia_W_SW; 35; A73G; T199C; T204C	16571	Asia_W_SW	I2c	I	I	35

Supplementary Table S4 Human mitochondrial database (hMITO DB v1.0) metadata[a]

Row	Name (accession no.)	Description	Size	Geo_Region	Haplogroup	Macro_ Haplo	Macro_ Haplo2	Total Variants
452	JQ702254.1	JQ702254.1; K1d1; Asia_W; 38; A73G; T195C; A263G	16573	Asia_W	K1d1	K	K	38
453	JQ702255.1	JQ702255.1; A12a; Asia_NE_America_N; 39; A73G; T152C; A235G	16568	Asia_NE_America_N	A12a	A	A	39
454	JQ702256.1	JQ702256.1; H35a; Asia_W_Europe; 12; A263G; C309CCT; T310C	16570	Asia_W_Europe	H35a	H	H	12
455	JQ702257.1	JQ702257.1; V2; Europe_S; 19; T72C; A263G; C309CCT	16570	Europe_S	V2	V	V	19
456	JQ702258.1	JQ702258.1; F1a1; Asia_SE_E; 35; A73G; A249d; A263G	16567	Asia_SE_E	F1a1	F	F	35
457	JQ702259.1	JQ702259.1; J1b1a1; Asia_W; 36; A73G; C242T; A263G	16569	Asia_W	J1b1a1	J	J	36
458	JQ702260.1	JQ702260.1; H5; Asia_W_Europe; 16; A263G; C315CC; C456T	16569	Asia_W_Europe	H5	H	H	16
459	JQ702261.1	JQ702261.1; L2a1c1a2; Africa_W_C; 61; A73G; G143A; T146C	16569	Africa_W_C	L2a1c1a2	L2	L2	61
460	JQ702262.1	JQ702262.1; H1bb; Asia_W_Europe; 13; A73G; T152C; A263G	16571	Asia_W_Europe	H1bb	H	H	13
461	JQ702263.1	JQ702263.1; H1e2c; Asia_W_Europe; 14; A73G; A263G; C315CC	16570	Asia_W_Europe	H1e2c	H	H	14
462	JQ702264.1	JQ702264.1; D5a3a1a; Asia_E_America_N_S; 44; A73G; T146C; C150T	16570	Asia_E_America_N_S	D5a3a1a	D	D	44
463	JQ702265.1	JQ702265.1; H6a1a2b1; Asia_W_Europe; 21; T239C; A263G; C309CCCT	16571	Asia_W_Europe	H6a1a2b1	H	H	21
464	JQ702266.1	JQ702266.1; K1a; Asia_W; 33; A73G; G228A; A263G	16569	Asia_W	K1a	K	K	33
465	JQ702267.1	JQ702267.1; U4d1a1; Asia_W_Europe_C; 35; A73G; T195C; A263G	16573	Asia_W_Europe_C	U4d1a1	U	U4	35
466	JQ702268.1	JQ702268.1; D4h1c1; Asia_E_America_N_S; 42; A73G; T152C; A263G	16569	Asia_E_America_N_S	D4h1c1	D	D	42
467	JQ702269.1	JQ702269.1; H1az; Asia_W_Europe; 11; A263G; C309CCT; T310C	16570	Asia_W_Europe	H1az	H	H	11
468	JQ702270.1	JQ702270.1; U4a1b; Asia_N_Europe_N; 33; A73G; T152C; T195C	16570	Asia_N_Europe_N	U4a1b	U	U4	33
469	JQ702271.1	JQ702271.1; K1a16; Asia_W; 33; A73G; T195C; A263G	16573	Asia_W	K1a16	K	K	33
470	JQ702272.1	JQ702272.1; H10h; Asia_W_Europe; 13; A263G; C315CC; A750G	16569	Asia_W_Europe	H10h	H	H	13
471	JQ702273.1	JQ702273.1; K1a+150; Asia_W; 32; A73G; C150T; T152C	16569	Asia_W	K1a+150	K	K	32
472	JQ702274.1	JQ702274.1; T1a1; Asia_W; 35; A73G; T152C; T195C	16569	Asia_W	T1a1	T	T	35
473	JQ702275.1	JQ702275.1; H1at1a; Asia_W_Europe; 13; T152C; A263G; C315CC	16569	Asia_W_Europe	H1at1a	H	H	13
474	JQ702276.1	JQ702276.1; K1a10a; Asia_W; 36; A73G; T195C; A230T	16571	Asia_W	K1a10a	K	K	36
475	JQ702277.1	JQ702277.1; H7c4; Asia_W_Europe; 19; G75A; A263G; C309CCCT	16571	Asia_W_Europe	H7c4	H	H	19
476	JQ702278.1	JQ702278.1; T2b4i; Asia_W; 37; A73G; A263G; C309CCT	16570	Asia_W	T2b4i	T	T	37
477	JQ702279.1	JQ702279.1; H34; Asia_W_Europe; 16; T152C; A263G; C309CCCT	16571	Asia_W_Europe	H34	H	H	16
478	JQ702280.1	JQ702280.1; U4a3a; Asia_N_Europe_N; 35; A73G; T195C; G247A	16571	Asia_N_Europe_N	U4a3a	U	U4	35
479	JQ702281.1	JQ702281.1; K1a4a1; Asia_W; 38; A73G; T152C; T204C	16569	Asia_W	K1a4a1	K	K	38
480	JQ702282.1	JQ702282.1; K2a9; Asia_W; 34; A73G; T146C; A263G	16569	Asia_W	K2a9	K	K	34
481	JQ702283.1	JQ702283.1; H14a2b; Asia_W_Europe; 14; A263G; C315CC; A750G	16569	Asia_W_Europe	H14a2b	H	H	14
482	JQ702284.1	JQ702284.1; I2; Asia_W_SW; 35; A73G; T152C; T199C	16570	Asia_W_SW	I2	I	I	35
483	JQ702285.1	JQ702285.1; H6b; Asia_W_Europe; 16; T239C; A263G; C309CCCT	16573	Asia_W_Europe	H6b	H	H	16
484	JQ702286.1	JQ702286.1; T2e1; Asia_W; 35; C41T; A73G; C150T	16570	Asia_W	T2e1	T	T	35
485	JQ702287.1	JQ702287.1; H2a2a1b; Asia_W_Europe; 1; A9299G; ;	16568	Asia_W_Europe	H2a2a1b	H	H	1
486	JQ702288.1	JQ702288.1; H1j3; Asia_W_Europe; 13; A263G; C315CC; A750G	16570	Asia_W_Europe	H1j3	H	H	13
487	JQ702289.1	JQ702289.1; D4q; Asia_E_America_N_S; 40; A73G; T146C; A153G	16569	Asia_E_America_N_S	D4q	D	D	40
488	JQ702290.1	JQ702290.1; A2d1a; Asia_NE_America_N; 35; A73G; T146C; A153G	16558	Asia_NE_America_N	A2d1a	A	A	35
489	JQ702291.1	JQ702291.1; H4a1a1; Asia_W_Europe; 17; A263G; C315CC; A750G	16569	Asia_W_Europe	H4a1a1	H	H	17
490	JQ702292.1	JQ702292.1; H100; Asia_W_Europe; 14; A93G; A200G; A263G	16569	Asia_W_Europe	H100	H	H	14
491	JQ702293.1	JQ702293.1; B2l; Asia_SE_E_America_N_S; 32; A73G; T159C; A263G	16562	Asia_SE_E_America_N_S	B2l	B	B	32
492	JQ702294.1	JQ702294.1; T2f5; Asia_W; 38; A73G; A263G; C315CC	16560	Asia_W	T2f5	T	T	38

Supplementary Table S4 Human mitochondrial database (hMITO DB v1.0) metadata[a]

Row	Name (accession no.)	Description	Size	Geo_Region	Haplogroup	Macro_ Haplo	Macro_ Haplo2	Total Variants
493	JQ702295.1	JQ702295.1; U5b3e; Asia_W_Europe_C; 28; A73G; C150T; G228A	16569	Asia_W_Europe_C	U5b3e	U	U5	28
494	JQ702296.1	JQ702296.1; H1c1; Asia_W_Europe; 12; A263G; C315CC; T477C	16569	Asia_W_Europe	H1c1	H	H	12
495	JQ702297.1	JQ702297.1; H33a; Asia_W_Europe; 9; A263G; C315CC; A750G	16569	Asia_W_Europe	H33a	H	H	9
496	JQ702298.1	JQ702298.1; W3b1; Asia_W; 37; A73G; C194T; T195C	16570	Asia_W	W3b1	W	W	37
497	JQ702299.1	JQ702299.1; HV+16311; Asia_W_Europe; 16; T195C; A263G; C309CCT	16570	Asia_W_Europe	HV+16311	H	H	16
498	JQ702300.1	JQ702300.1; V16; Europe_S; 15; T72C; A263G; C315CC	16569	Europe_S	V16	V	V	15
499	JQ702301.1	JQ702301.1; H5a1g1a; Asia_W_Europe; 20; T152C; A200G; A263G	16567	Asia_W_Europe	H5a1g1a	H	H	20
500	JQ702302.1	JQ702302.1; K1a4a1f1; Asia_W; 38; A73G; T152C; T217C	16569	Asia_W	K1a4a1f1	K	K	38
501	JQ702303.1	JQ702303.1; H13a1a1a; Asia_W_Europe; 17; T152C; A263G; C309CCT	16570	Asia_W_Europe	H13a1a1a	H	H	17
502	JQ702304.1	JQ702304.1; K1b1a1c; Asia_W; 44; A73G; T152C; A263G	16572	Asia_W	K1b1a1c	K	K	44
503	JQ702305.1	JQ702305.1; H18; Asia_W_Europe; 12; A263G; C315CC; A750G	16569	Asia_W_Europe	H18	H	H	12
504	JQ702306.1	JQ702306.1; K1a2c; Asia_W; 37; A73G; T146C; T152C	16574	Asia_W	K1a2c	K	K	37
505	JQ702307.1	JQ702307.1; L2a1c; Africa_W_C; 57; A73G; G143A; T146C	16566	Africa_W_C	L2a1c	L2	L2	57
506	JQ702308.1	JQ702308.1; H1+16189; Asia_W_Europe; 14; A263G; C309CCCT; T310C	16571	Asia_W_Europe	H1+16189	H	H	14
507	JQ702309.1	JQ702309.1; H2a2b3; Asia_W_Europe; 9; A263G; C309CCT; T310C	16570	Asia_W_Europe	H2a2b3	H	H	9
508	JQ702310.1	JQ702310.1; U5a2+16294; Asia_W_Europe_C; 35; C64T; A73G; T152C	16569	Asia_W_Europe_C	U5a2+16294	U	U5	35
509	JQ702311.1	JQ702311.1; J1c2e; Asia_W; 37; A73G; A93G; G185A	16568	Asia_W	J1c2e	J	J	37
510	JQ702312.1	JQ702312.1; T2b26; Asia_W; 36; A73G; C309CCT; T310C	16572	Asia_W	T2b26	T	T	36
511	JQ702313.1	JQ702313.1; H1e7; Asia_W_Europe; 14; A263G; C309CCT; T310C	16572	Asia_W_Europe	H1e7	H	H	14
512	JQ702314.1	JQ702314.1; H36; Asia_W_Europe; 16; T152C; A263G; C309CCT	16570	Asia_W_Europe	H36	H	H	16
513	JQ702315.1	JQ702315.1; K1a4a1i; Asia_W; 33; A73G; A263G; C315CC	16569	Asia_W	K1a4a1i	K	K	33
514	JQ702316.1	JQ702316.1; U2e1e; Asia_S_W_Europe; 42; A73G; T152C; T217C	16569	Asia_S_W_Europe	U2e1e	U	U2	42
515	JQ702317.1	JQ702317.1; H5a1g1a; Asia_W_Europe; 20; A263G; C309CCCT; T310C	16569	Asia_W_Europe	H5a1g1a	H	H	20
516	JQ702318.1	JQ702318.1; H6a1a; Asia_W_Europe; 17; T239C; A263G; C309CCCT	16571	Asia_W_Europe	H6a1a	H	H	17
517	JQ702319.1	JQ702319.1; H6a1a2a; Asia_W_Europe; 20; C41T; T239C; A263G	16570	Asia_W_Europe	H6a1a2a	H	H	20
518	JQ702320.1	JQ702320.1; U5a2b4; Asia_W_Europe_C; 31; A73G; A263G; C309CCT	16572	Asia_W_Europe_C	U5a2b4	U	U5	31
519	JQ702321.1	JQ702321.1; C5c1a; Asia_NE_America_N_S; 46; A73G; T152C; A249d	16569	Asia_NE_America_N_S	C5c1a	C	C	46
520	JQ702322.1	JQ702322.1; B2; Asia_SE_E_America_N_S; 35; A73G; G185A; A189G	16559	Asia_SE_E_America_N_S	B2	B	B	35
521	JQ702323.1	JQ702323.1; T2b4a1; Asia_W; 37; A73G; A263G; C309CCT	16570	Asia_W	T2b4a1	T	T	37
522	JQ702324.1	JQ702324.1; A2ac1; Asia_NE_America_N; 38; C64T; A73G; T146C	16568	Asia_NE_America_N	A2ac1	A	A	38
523	JQ702325.1	JQ702325.1; K1b2a2a; Asia_W; 37; A73G; T146C; T195C	16569	Asia_W	K1b2a2a	K	K	37
524	JQ702326.1	JQ702326.1; L0a1b1a1; Africa_S_SE; 88; A93G; A95C; G185A	16569	Africa_S_SE	L0a1b1a1	L0	L0	88
525	JQ702327.1	JQ702327.1; W1c1; Asia_W; 36; A73G; T119C; A189G	16569	Asia_W	W1c1	W	W	36
526	JQ702328.1	JQ702328.1; H1e; Asia_W_Europe; 11; A263G; C315CC; A750G	16569	Asia_W_Europe	H1e	H	H	11
527	JQ702329.1	JQ702329.1; H3g1b; Asia_W_Europe; 16; T152C; A263G; C309CCT	16570	Asia_W_Europe	H3g1b	H	H	16
528	JQ702330.1	JQ702330.1; H6a1b4; Asia_W_Europe; 19; T239C; A263G; C309CCCT	16571	Asia_W_Europe	H6a1b4	H	H	19
529	JQ702331.1	JQ702331.1; U5a1b; Asia_W_Europe_C; 25; A73G; A263G; C309CCT	16570	Asia_W_Europe_C	U5a1b	U	U5	25
530	JQ702332.1	JQ702332.1; W1; Asia_W; 34; A73G; G143A; A189G	16569	Asia_W	W1	W	W	34
531	JQ702333.1	JQ702333.1; K1c1b; Asia_W; 35; A73G; T146C; T152C	16568	Asia_W	K1c1b	K	K	35
532	JQ702334.1	JQ702334.1; H1i; Asia_W_Europe; 15; T152C; A263G; C309CCT	16570	Asia_W_Europe	H1i	H	H	15
533	JQ702335.1	JQ702335.1; H1an2; Asia_W_Europe; 14; C151T; T152C; A263G	16569	Asia_W_Europe	H1an2	H	H	14

Supplementary Table S4 Human mitochondrial database (hMITO DB v1.0) metadata[a]

Row	Name (accession no.)	Description	Size	Geo_Region	Haplogroup	Macro_ Haplo	Macro_ Haplo2	Total Variants
534	JQ702336.1	JQ702336.1; H1c15; Asia_W_Europe; 14; A263G; C309CCT; T310C	16573	Asia_W_Europe	H1c15	H	H	14
535	JQ702337.1	JQ702337.1; H7d2a; Asia_W_Europe; 13; A263G; A291AA; C315CC	16570	Asia_W_Europe	H7d2a	H	H	13
536	JQ702338.1	JQ702338.1; J1c7a; Asia_W; 35; A73G; G185A; T195C	16571	Asia_W	J1c7a	J	J	35
537	JQ702339.1	JQ702339.1; U5a1a1; Asia_W_Europe_C; 29; A73G; A263G; C309CCT	16572	Asia_W_Europe_C	U5a1a1	U	U5	29
538	JQ702340.1	JQ702340.1; T1a1; Asia_W; 37; A73G; T152C; T195C	16570	Asia_W	T1a1	T	T	37
539	JQ702341.1	JQ702341.1; K1a4a1e; Asia_W; 37; A73G; T152C; T204C	16571	Asia_W	K1a4a1e	K	K	37
540	JQ702342.1	JQ702342.1; I1a1d; Asia_W_SW; 48; A73G; T199C; G203A	16570	Asia_W_SW	I1a1d	I	I	48
541	JQ702343.1	JQ702343.1; T1a1+@152; Asia_W; 35; A73G; T195C; A263G	16570	Asia_W	T1a1+@152	T	T	35
542	JQ702344.1	JQ702344.1; T2a1b; Asia_W; 36; A73G; A263G; C315CC	16569	Asia_W	T2a1b	T	T	36
543	JQ702345.1	JQ702345.1; H5b3; Asia_W_Europe; 15; T199C; A263G; C309CCT	16570	Asia_W_Europe	H5b3	H	H	15
544	JQ702346.1	JQ702346.1; J2b1a4; Asia_W; 36; A73G; C150T; T152C	16573	Asia_W	J2b1a4	J	J	36
545	JQ702347.1	JQ702347.1; U5a1b; Asia_W_Europe_C; 26; A73G; A263G; C315CC	16569	Asia_W_Europe_C	U5a1b	U	U5	26
546	JQ702348.1	JQ702348.1; K1a3a1b; Asia_W; 36; A73G; A263G; C309CCT	16572	Asia_W	K1a3a1b	K	K	36
547	JQ702349.1	JQ702349.1; H1e1c; Asia_W_Europe; 13; A263G; C315CC; A750G	16569	Asia_W_Europe	H1e1c	H	H	13
548	JQ702350.1	JQ702350.1; T2f1a1; Asia_W; 42; A73G; T195C; A263G	16561	Asia_W	T2f1a1	T	T	42
549	JQ702351.1	JQ702351.1; U4a2d; Asia_N_Europe_N; 29; A73G; T195C; A263G	16568	Asia_N_Europe_N	U4a2d	U	U4	29
550	JQ702352.1	JQ702352.1; H3a1a; Asia_W_Europe; 16; T152C; G228A; A263G	16570	Asia_W_Europe	H3a1a	H	H	16
551	JQ702353.1	JQ702353.1; K2a3; Asia_W; 37; A73G; T146C; T152C	16570	Asia_W	K2a3	K	K	37
552	JQ702354.1	JQ702354.1; H24; Asia_W_Europe; 10; A263G; C315CC; A750G	16569	Asia_W_Europe	H24	H	H	10
553	JQ702355.1	JQ702355.1; U5a2a1b1; Asia_W_Europe_C; 31; A73G; T204C; T252C	16570	Asia_W_Europe_C	U5a2a1b1	U	U5	31
554	JQ702356.1	JQ702356.1; T2a1b1a; Asia_W; 40; A73G; T152C; A263G	16569	Asia_W	T2a1b1a	T	T	40
555	JQ702357.1	JQ702357.1; H61a; Asia_W_Europe; 13; A263G; C315CC; A750G	16569	Asia_W_Europe	H61a	H	H	13
556	JQ702358.1	JQ702358.1; W9; Asia_W; 36; A73G; G143A; A189G	16570	Asia_W	W9	W	W	36
557	JQ702359.1	JQ702359.1; H1b1+16362; Asia_W_Europe; 16; A263G; C315CC; CA522d	16567	Asia_W_Europe	H1b1+16362	H	H	16
558	JQ702360.1	JQ702360.1; H1o; Asia_W_Europe; 13; C150T; A263G; C315CC	16569	Asia_W_Europe	H1o	H	H	13
559	JQ702361.1	JQ702361.1; H4a1a4b; Asia_W_Europe; 19; T195C; A263G; C315CC	16567	Asia_W_Europe	H4a1a4b	H	H	19
560	JQ702362.1	JQ702362.1; H1n+146; Asia_W_Europe; 14; T146C; A263G; C309CCCT	16571	Asia_W_Europe	H1n+146	H	H	14
561	JQ702363.1	JQ702363.1; H24a; Asia_W_Europe; 12; A263G; C315CC; A750G	16569	Asia_W_Europe	H24a	H	H	12
562	JQ702364.1	JQ702364.1; J2a1a2a1a; Asia_W; 43; A73G; C150T; T152C	16570	Asia_W	J2a1a2a1a	J	J	43
563	JQ702365.1	JQ702365.1; K1a+195; Asia_W; 33; A73G; T195C; A263G	16572	Asia_W	K1a+195	K	K	33
564	JQ702366.1	JQ702366.1; J1c2m1; Asia_W; 35; A73G; G185A; A188G	16570	Asia_W	J1c2m1	J	J	35
565	JQ702367.1	JQ702367.1; H47; Asia_W_Europe; 11; A263G; C315CC; A750G	16569	Asia_W_Europe	H47	H	H	11
566	JQ702368.1	JQ702368.1; H2a2b1a; Asia_W_Europe; 10; A263G; C309CCT; T310C	16570	Asia_W_Europe	H2a2b1a	H	H	10
567	JQ702369.1	JQ702369.1; I4a; Asia_W_SW; 34; A73G; T199C; T204C	16569	Asia_W_SW	I4a	I	I	34
568	JQ702370.1	JQ702370.1; H1ae2a; Asia_W_Europe; 14; A263G; C309CCT; T310C	16570	Asia_W_Europe	H1ae2a	H	H	14
569	JQ702371.1	JQ702371.1; H1ba; Asia_W_Europe; 12; A263G; C315CC; A750G	16569	Asia_W_Europe	H1ba	H	H	12
570	JQ702372.1	JQ702372.1; K1a3a; Asia_W; 38; A73G; T199C; A263G	16570	Asia_W	K1a3a	K	K	38
571	JQ702373.1	JQ702373.1; K2a6; Asia_W; 33; A73G; T146C; T152C	16569	Asia_W	K2a6	K	K	33
572	JQ702374.1	JQ702374.1; H23; Asia_W_Europe; 10; A263G; C315CC; A750G	16569	Asia_W_Europe	H23	H	H	10
573	JQ702375.1	JQ702375.1; H58a; Asia_W_Europe; 16; T152C; A263G; C309CCT	16570	Asia_W_Europe	H58a	H	H	16
574	JQ702376.1	JQ702376.1; U5b1d1a; Asia_W_Europe_C; 29; A73G; C150T; A263G	16572	Asia_W_Europe_C	U5b1d1a	U	U5	29

Supplementary Table S4 Human mitochondrial database (hMITO DB v1.0) metadata[a]

Row	Name (accession no.)	Description	Size	Geo_Region	Haplogroup	Macro_ Haplo	Macro_ Haplo2	Total Variants
575	JQ702377.1	JQ702377.1; H1a1; Asia_W_Europe; 14; A73G; A263G; C315CC	16569	Asia_W_Europe	H1a1	H	H	14
576	JQ702378.1	JQ702378.1; H7b2a; Asia_W_Europe; 15; A189G; A263G; C309CCT	16570	Asia_W_Europe	H7b2a	H	H	15
577	JQ702379.1	JQ702379.1; T2a1a; Asia_W; 37; A73G; A263G; C309CCT	16570	Asia_W	T2a1a	T	T	37
578	JQ702380.1	JQ702380.1; K2a7; Asia_W; 34; A73G; T146C; T152C	16568	Asia_W	K2a7	K	K	34
579	JQ702381.1	JQ702381.1; L3b1a; Africa_E; 42; A73G; T195C; A263G	16565	Africa_E	L3b1a	L3	L3	42
580	JQ702382.1	JQ702382.1; H1c; Asia_W_Europe; 12; A263G; C309CCT; T310C	16570	Asia_W_Europe	H1c	H	H	12
581	JQ702383.1	JQ702383.1; H1bs; Asia_W_Europe; 15; A263G; C309CCT; T310C	16570	Asia_W_Europe	H1bs	H	H	15
582	JQ702384.1	JQ702384.1; U5b1b1a; Asia_W_Europe_C; 28; A73G; C150T; A263G	16569	Asia_W_Europe_C	U5b1b1a	U	U5	28
583	JQ702385.1	JQ702385.1; H6a1b4; Asia_W_Europe; 18; T152C; T239C; A263G	16569	Asia_W_Europe	H6a1b4	H	H	18
584	JQ702386.1	JQ702386.1; J1c5a1; Asia_W; 33; A73G; G185A; G228A	16569	Asia_W	J1c5a1	J	J	33
585	JQ702387.1	JQ702387.1; U5b2a1a1b; Asia_W_Europe_C; 33; A73G; C150T; A249d	16569	Asia_W_Europe_C	U5b2a1a1b	U	U5	33
586	JQ702388.1	JQ702388.1; H11a2a1; Asia_W_Europe; 18; T195C; A263G; C309CCT	16570	Asia_W_Europe	H11a2a1	H	H	18
587	JQ702389.1	JQ702389.1; U2e3a; Asia_S_W_Europe; 45; A73G; T146C; T152C	16572	Asia_S_W_Europe	U2e3a	U	U2	45
588	JQ702390.1	JQ702390.1; J1c2a1; Asia_W; 37; A73G; T146C; G185A	16570	Asia_W	J1c2a1	J	J	37
589	JQ702391.1	JQ702391.1; V15a; Europe_S; 19; T72C; A263G; C309CCT	16570	Europe_S	V15a	V	V	19
590	JQ702392.1	JQ702392.1; U5a2b; Asia_W_Europe_C; 27; A73G; A263G; C309CCT	16570	Asia_W_Europe_C	U5a2b	U	U5	27
591	JQ702393.1	JQ702393.1; A2r1; Asia_NE_America_N; 34; A73G; G103A; T146C	16568	Asia_NE_America_N	A2r1	A	A	34
592	JQ702394.1	JQ702394.1; K2b1a1a; Asia_W; 36; A73G; T146C; T199C	16569	Asia_W	K2b1a1a	K	K	36
593	JQ702395.1	JQ702395.1; J1c3c1; Asia_W; 31; A73G; G185A; G228A	16569	Asia_W	J1c3c1	J	J	31
594	JQ702396.1	JQ702396.1; H11a2a3; Asia_W_Europe; 18; T195C; A263G; C315CC	16569	Asia_W_Europe	H11a2a3	H	H	18
595	JQ702397.1	JQ702397.1; H2a1a; Asia_W_Europe; 12; A263G; C315CC; A750G	16569	Asia_W_Europe	H2a1a	H	H	12
596	JQ702398.1	JQ702398.1; U2e1a1; Asia_S_W_Europe; 41; A73G; T152C; T217C	16573	Asia_S_W_Europe	U2e1a1	U	U2	41
597	JQ702399.1	JQ702399.1; H13a1a1e; Asia_W_Europe; 18; A263G; C309CCT; T310C	16568	Asia_W_Europe	H13a1a1e	H	H	18
598	JQ702400.1	JQ702400.1; U5a1b1d; Asia_W_Europe_C; 29; A73G; A263G; C309CCT	16570	Asia_W_Europe_C	U5a1b1d	U	U5	29
599	JQ702401.1	JQ702401.1; H1+16189; Asia_W_Europe; 14; A263G; C309CCT; T310C	16570	Asia_W_Europe	H1+16189	H	H	14
600	JQ702402.1	JQ702402.1; H5c; Asia_W_Europe; 10; A263G; C315CC; C456T	16569	Asia_W_Europe	H5c	H	H	10
601	JQ702403.1	JQ702403.1; U4a2a; Asia_N_Europe_N; 31; A73G; T195C; G203A	16568	Asia_N_Europe_N	U4a2a	U	U4	31
602	JQ702404.1	JQ702404.1; U5a1d1; Asia_W_Europe_C; 30; A73G; A263G; C315CC	16569	Asia_W_Europe_C	U5a1d1	U	U5	30
603	JQ702405.1	JQ702405.1; H1a2; Asia_W_Europe; 12; A73G; A263G; C315CC	16569	Asia_W_Europe	H1a2	H	H	12
604	JQ702406.1	JQ702406.1; U5b2c1; Asia_W_Europe_C; 31; A73G; C150T; G185A	16571	Asia_W_Europe_C	U5b2c1	U	U5	31
605	JQ702407.1	JQ702407.1; M49c1; Asia_S; 33; A73G; T204C; A263G	16569	Asia_S	M49c1	M	M49	33
606	JQ702408.1	JQ702408.1; N3a; Africa_NE_Europe_E; 35; A73G; T146C; A210G	16570	Africa_NE_Europe_E	N3a	N	N3	35
607	JQ702409.1	JQ702409.1; H1b; Asia_W_Europe; 15; T152C; A263G; C309CCT	16572	Asia_W_Europe	H1b	H	H	15
608	JQ702410.1	JQ702410.1; H4a1a1a1a1; Asia_W_Europe; 23; A73G; A263G; C309CCT	16568	Asia_W_Europe	H4a1a1a1a1	H	H	23
609	JQ702411.1	JQ702411.1; H3ap; Asia_W_Europe; 12; T199C; A263G; C315CC	16569	Asia_W_Europe	H3ap	H	H	12
610	JQ702412.1	JQ702412.1; H5a1c1a; Asia_W_Europe; 18; A263G; C309CCT; T310C	16568	Asia_W_Europe	H5a1c1a	H	H	18
611	JQ702413.1	JQ702413.1; I3a; Asia_W_SW; 40; A73G; T152C; T199C	16576	Asia_W_SW	I3a	I	I	40
612	JQ702414.1	JQ702414.1; V2c; Europe_S; 18; A200G; A263G; C315CC	16569	Europe_S	V2c	V	V	18
613	JQ702415.1	JQ702415.1; H1b3; Asia_W_Europe; 15; A263G; C315CC; A750G	16569	Asia_W_Europe	H1b3	H	H	15
614	JQ702416.1	JQ702416.1; H3h; Asia_W_Europe; 12; A263G; C315CC; A750G	16569	Asia_W_Europe	H3h	H	H	12
615	JQ702417.1	JQ702417.1; K2a6; Asia_W; 34; A73G; T146C; T152C	16569	Asia_W	K2a6	K	K	34

Supplementary Table S4 Human mitochondrial database (hMITO DB v1.0) metadata[a]

Row	Name (accession no.)	Description	Size	Geo_Region	Haplogroup	Macro_ Haplo	Macro_ Haplo2	Total Variants
616	JQ702418.1	JQ702418.1; H6a1a; Asia_W_Europe; 17; T239C; A263G; C309CCT	16570	Asia_W_Europe	H6a1a	H	H	17
617	JQ702419.1	JQ702419.1; H73; Asia_W_Europe; 11; A263G; C309CCCT; T310C	16571	Asia_W_Europe	H73	H	H	11
618	JQ702420.1	JQ702420.1; L3d1b2; Africa_E; 35; A73G; C150T; T152C	16570	Africa_E	L3d1b2	L3	L3	35
619	JQ702421.1	JQ702421.1; W3a1; Asia_W; 39; A73G; G143A; A189G	16569	Asia_W	W3a1	W	W	39
620	JQ702422.1	JQ702422.1; H24a; Asia_W_Europe; 10; A263G; C315CC; A750G	16569	Asia_W_Europe	H24a	H	H	10
621	JQ702423.1	JQ702423.1; H49; Asia_W_Europe; 13; A263G; C309CCT; T310C	16570	Asia_W_Europe	H49	H	H	13
622	JQ702424.1	JQ702424.1; J2b1h; Asia_W; 39; A73G; C150T; T152C	16570	Asia_W	J2b1h	J	J	39
623	JQ702425.1	JQ702425.1; A2ad; Asia_NE_America_N; 37; C64T; A73G; T146C	16568	Asia_NE_America_N	A2ad	A	A	37
624	JQ702426.1	JQ702426.1; H2a1; Asia_W_Europe; 10; A263G; C315CC; A750G	16569	Asia_W_Europe	H2a1	H	H	10
625	JQ702427.1	JQ702427.1; H3; Asia_W_Europe; 11; A263G; C309CCC; A750G	16570	Asia_W_Europe	H3	H	H	11
626	JQ702428.1	JQ702428.1; L0a1a+200; Africa_S_SE; 87; C64T; A93G; T152C	16568	Africa_S_SE	L0a1a+200	L0	L0	87
627	JQ702429.1	JQ702429.1; K2a2a1; Asia_W; 37; A73G; T146C; T152C	16569	Asia_W	K2a2a1	K	K	37
628	JQ702430.1	JQ702430.1; L2a1+143+16189 (16192)+@16309; Africa_W_C; 57; A73G; G143A	16571	Africa_W_C	L2a1+143+1618	L2	L2	57
629	JQ702431.1	JQ702431.1; U5b2a2c; Asia_W_Europe_C; 32; A73G; C150T; A263G	16569	Asia_W_Europe_C	U5b2a2c	U	U5	32
630	JQ702432.1	JQ702432.1; U2e2a1a1; Asia_S_W_Europe; 46; A73G; T146C; T152C	16573	Asia_S_W_Europe	U2e2a1a1	U	U2	46
631	JQ702433.1	JQ702433.1; H1e4; Asia_W_Europe; 12; T146C; A263G; C315CC	16569	Asia_W_Europe	H1e4	H	H	12
632	JQ702434.1	JQ702434.1; J1b1a1; Asia_W; 38; A73G; T152C; C242T	16569	Asia_W	J1b1a1	J	J	38
633	JQ702435.1	JQ702435.1; T2b13; Asia_W; 36; A73G; A263G; C309CCCT	16571	Asia_W	T2b13	T	T	36
634	JQ702436.1	JQ702436.1; H1c1a1; Asia_W_Europe; 14; A263G; C315CC; T477C	16569	Asia_W_Europe	H1c1a1	H	H	14
635	JQ702437.1	JQ702437.1; U5a1a1c; Asia_W_Europe_C; 28; A73G; A263G; C315CC	16569	Asia_W_Europe_C	U5a1a1c	U	U5	28
636	JQ702438.1	JQ702438.1; V9; Europe_S; 16; T72C; T204C; A263G	16570	Europe_S	V9	V	V	16
637	JQ702439.1	JQ702439.1; M3a2; Asia_S; 34; A73G; A263G; C315CC	16570	Asia_S	M3a2	M	M3	34
638	JQ702440.1	JQ702440.1; J1c1; Asia_W; 35; A73G; G228A; A263G	16569	Asia_W	J1c1	J	J	35
639	JQ702441.1	JQ702441.1; L1c3b1a; Africa_C; 90; A73G; C151T; T152C	16566	Africa_C	L1c3b1a	L1	L1	90
640	JQ702442.1	JQ702442.1; J2b1a; Asia_W; 34; A73G; C150T; T152C	16569	Asia_W	J2b1a	J	J	34
641	JQ702443.1	JQ702443.1; H73a; Asia_W_Europe; 14; A263G; C309CCT; T310C	16571	Asia_W_Europe	H73a	H	H	14
642	JQ702444.1	JQ702444.1; HV+16311; Asia_W_Europe; 14; T195C; A263G; C309CCT	16570	Asia_W_Europe	HV+16311	H	H	14
643	JQ702445.1	JQ702445.1; J1c2; Asia_W; 32; A73G; G185A; A188G	16568	Asia_W	J1c2	J	J	32
644	JQ702446.1	JQ702446.1; U4b1a2; Asia_N_Europe_N; 34; A73G; T195C; A263G	16570	Asia_N_Europe_N	U4b1a2	U	U4	34
645	JQ702447.1	JQ702447.1; M3a1+204; Asia_S; 31; A73G; T204C; A263G	16571	Asia_S	M3a1+204	M	M3	31
646	JQ702448.1	JQ702448.1; H67a; Asia_W_Europe; 13; A263G; C309CCCT; T310C	16571	Asia_W_Europe	H67a	H	H	13
647	JQ702449.1	JQ702449.1; H17b; Asia_W_Europe; 12; C150T; A263G; C315CC	16569	Asia_W_Europe	H17b	H	H	12
648	JQ702450.1	JQ702450.1; W1c; Asia_W; 36; A73G; T119C; A189G	16569	Asia_W	W1c	W	W	36
649	JQ702451.1	JQ702451.1; A2i; Asia_NE_America_N; 41; C64T; A73G; G94A	16569	Asia_NE_America_N	A2i	A	A	41
650	JQ702452.1	JQ702452.1; K1c1b; Asia_W; 36; A73G; T146C; T152C	16568	Asia_W	K1c1b	K	K	36
651	JQ702453.1	JQ702453.1; H+152; Asia_W_Europe; 12; T152C; A263G; C309CCCT	16569	Asia_W_Europe	H+152	H	H	12
652	JQ702454.1	JQ702454.1; A2d1; Asia_NE_America_N; 38; C64T; A73G; T146C	16559	Asia_NE_America_N	A2d1	A	A	38
653	JQ702455.1	JQ702455.1; H1b1h; Asia_W_Europe; 17; A263G; C309CCCT; T310C	16569	Asia_W_Europe	H1b1h	H	H	17
654	JQ702456.1	JQ702456.1; V13; Europe_S; 17; T72C; A263G; C309CCT	16570	Europe_S	V13	V	V	17
655	JQ702457.1	JQ702457.1; I1a1c; Asia_W_SW; 43; A73G; A189G; T199C	16572	Asia_W_SW	I1a1c	I	I	43
656	JQ702458.1	JQ702458.1; H1b1+16362; Asia_W_Europe; 17; C151T; A263G; C309CCCT	16569	Asia_W_Europe	H1b1+16362	H	H	17

Supplementary Table S4 Human mitochondrial database (hMITO DB v1.0) metadata[a]

Row	Name (accession no.)	Description	Size	Geo_Region	Haplogroup	Macro_ Haplo	Macro_ Haplo2	Total Variants
657	JQ702459.1	JQ702459.1; J2b1g; Asia_W; 32; A73G; C150T; T152C	16567	Asia_W	J2b1g	J	J	32
658	JQ702460.1	JQ702460.1; T1a2; Asia_W; 38; A73G; T152C; A200G	16570	Asia_W	T1a2	T	T	38
659	JQ702461.1	JQ702461.1; T2b; Asia_W; 36; A73G; A263G; C309CCT	16568	Asia_W	T2b	T	T	36
660	JQ702462.1	JQ702462.1; H4a1a4b; Asia_W_Europe; 19; T195C; A263G; C315CC	16567	Asia_W_Europe	H4a1a4b	H	H	19
661	JQ702463.1	JQ702463.1; H5; Asia_W_Europe; 14; C150T; A263G; C309CCCT	16571	Asia_W_Europe	H5	H	H	14
662	JQ702464.1	JQ702464.1; T2b4+152; Asia_W; 39; A73G; T152C; A263G	16570	Asia_W	T2b4+152	T	T	39
663	JQ702465.1	JQ702465.1; H6a1b2; Asia_W_Europe; 20; T152C; T239C; A263G	16570	Asia_W_Europe	H6a1b2	H	H	20
664	JQ702466.1	JQ702466.1; H3ap; Asia_W_Europe; 13; A263G; C306T; CCT308-	16565	Asia_W_Europe	H3ap	H	H	13
665	JQ702467.1	JQ702467.1; H23; Asia_W_Europe; 11; A263G; C315CC; A750G	16569	Asia_W_Europe	H23	H	H	11
666	JQ702468.1	JQ702468.1; U5b2c1; Asia_W_Europe_C; 33; A73G; C150T; T152C	16571	Asia_W_Europe_C	U5b2c1	U	U5	33
667	JQ702469.1	JQ702469.1; R1a1a2; Asia_S_SE; 38; A73G; A263G; C295A	16570	Asia_S_SE	R1a1a2	R	R1	38
668	JQ702470.1	JQ702470.1; I1a1b; Asia_W_SW; 42; A73G; T199C; G203A	16572	Asia_W_SW	I1a1b	I	I	42
669	JQ702471.1	JQ702471.1; K1a4a1b2; Asia_W; 39; A73G; A263G; C309CCT	16570	Asia_W	K1a4a1b2	K	K	39
670	JQ702472.1	JQ702472.1; I1f; Asia_W_SW; 39; A73G; T152C; T199C	16572	Asia_W_SW	I1f	I	I	39
671	JQ702473.1	JQ702473.1; H2a1; Asia_W_Europe; 9; A263G; C309CCT; T310C	16570	Asia_W_Europe	H2a1	H	H	9
672	JQ702474.1	JQ702474.1; H1a6; Asia_W_Europe; 14; A73G; C151T; A263G	16569	Asia_W_Europe	H1a6	H	H	14
673	JQ702475.1	JQ702475.1; H6a1b2; Asia_W_Europe; 18; T239C; A263G; C315CC	16569	Asia_W_Europe	H6a1b2	H	H	18
674	JQ702476.1	JQ702476.1; T2b; Asia_W; 38; A73G; A263G; C309CCT	16570	Asia_W	T2b	T	T	38
675	JQ702477.1	JQ702477.1; J2b1a; Asia_W; 35; A73G; C150T; T152C	16570	Asia_W	J2b1a	J	J	35
676	JQ702478.1	JQ702478.1; H1bs; Asia_W_Europe; 14; A263G; C309CCCT; T310C	16571	Asia_W_Europe	H1bs	H	H	14
677	JQ702479.1	JQ702479.1; H3m; Asia_W_Europe; 12; A263G; C315CC; A750G	16569	Asia_W_Europe	H3m	H	H	12
678	JQ702480.1	JQ702480.1; H1e1a; Asia_W_Europe; 19; A56AC; A263G; C309CCT	16571	Asia_W_Europe	H1e1a	H	H	19
679	JQ702481.1	JQ702481.1; L3d1a2; Africa_E; 41; A73G; T152C; T195C	16568	Africa_E	L3d1a2	L3	L3	41
680	JQ702482.1	JQ702482.1; X2b10; Asia_W_America_N; 34; A73G; T146C; A153G	16570	Asia_W_America_N	X2b10	X	X	34
681	JQ702483.1	JQ702483.1; H1bw; Asia_W_Europe; 11; A263G; C315CC; A750G	16569	Asia_W_Europe	H1bw	H	H	11
682	JQ702484.1	JQ702484.1; R1a1a2; Asia_S_SE; 39; A73G; A263G; C295A	16570	Asia_S_SE	R1a1a2	R	R1	39
683	JQ702485.1	JQ702485.1; U2e1; Asia_S_W_Europe; 38; A73G; T152C; T217C	16573	Asia_S_W_Europe	U2e1	U	U2	38
684	JQ702486.1	JQ702486.1; J1c7; Asia_W; 34; A73G; T146C; G185A	16569	Asia_W	J1c7	J	J	34
685	JQ702487.1	JQ702487.1; K2b1a1; Asia_W; 37; A73G; T146C; A263G	16569	Asia_W	K2b1a1	K	K	37
686	JQ702488.1	JQ702488.1; J2b1g; Asia_W; 35; A73G; C150T; T152C	16567	Asia_W	J2b1g	J	J	35
687	JQ702489.1	JQ702489.1; H5; Asia_W_Europe; 12; A73G; A263G; C315CC	16569	Asia_W_Europe	H5	H	H	12
688	JQ702490.1	JQ702490.1; HV7; Asia_W; 19; T146C; A263G; C315CC	16567	Asia_W	HV7	HV	HV	19
689	JQ702491.1	JQ702491.1; H1e2d; Asia_W_Europe; 14; A263G; C315CC; CA522d	16567	Asia_W_Europe	H1e2d	H	H	14
690	JQ702492.1	JQ702492.1; T2b21b; Asia_W; 38; A73G; T152C; A263G	16570	Asia_W	T2b21b	T	T	38
691	JQ702493.1	JQ702493.1; I3c; Asia_W_SW; 36; A73G; T152C; T199C	16576	Asia_W_SW	I3c	I	I	36
692	JQ702494.1	JQ702494.1; H1ap1; Asia_W_Europe; 16; T152C; A191AA; A263G	16571	Asia_W_Europe	H1ap1	H	H	16
693	JQ702495.1	JQ702495.1; H1as2; Asia_W_Europe; 13; A263G; C309CCT; T310C	16570	Asia_W_Europe	H1as2	H	H	13
694	JQ702496.1	JQ702496.1; U5a1a2b1; Asia_W_Europe_C; 33; A73G; A263G; C315CC	16569	Asia_W_Europe_C	U5a1a2b1	U	U5	33
695	JQ702497.1	JQ702497.1; H1bc; Asia_W_Europe; 16; T152C; A263G; C315CC	16571	Asia_W_Europe	H1bc	H	H	16
696	JQ702498.1	JQ702498.1; V1a1; Europe_S; 20; T72C; A263G; C309CCT	16570	Europe_S	V1a1	V	V	20
697	JQ702499.1	JQ702499.1; J1c2c1; Asia_W; 36; A73G; T146C; G185A	16569	Asia_W	J1c2c1	J	J	36

Supplementary Table S4 Human mitochondrial database (hMITO DB v1.0) metadata[a]

Row	Name (accession no.)	Description	Size	Geo_Region	Haplogroup	Macro_ Haplo	Macro_ Haplo2	Total Variants
698	JQ702500.1	JQ702500.1; U5b1b1a1a; Asia_W_Europe_C; 31; A73G; C150T; A263G	16570	Asia_W_Europe_C	U5b1b1a1a	U	U5	31
699	JQ702501.1	JQ702501.1; H1c13; Asia_W_Europe; 11; A263G; C315CC; T477C	16569	Asia_W_Europe	H1c13	H	H	11
700	JQ702502.1	JQ702502.1; H5a1h; Asia_W_Europe; 13; A263G; C315CC; C456T	16567	Asia_W_Europe	H5a1h	H	H	13
701	JQ702503.1	JQ702503.1; L2a1c; Africa_W_C; 59; A73G; G143A; T146C	16570	Africa_W_C	L2a1c	L2	L2	59
702	JQ702504.1	JQ702504.1; L4b2b1; Africa_E; 56; A73G; T146C; T195C	16571	Africa_E	L4b2b1	L3	L3	56
703	JQ702505.1	JQ702505.1; K1a4; Asia_W; 33; A73G; A263G; C315CC	16571	Asia_W	K1a4	K	K	33
704	JQ702506.1	JQ702506.1; H6a1a; Asia_W_Europe; 17; T239C; A263G; C309CCT	16570	Asia_W_Europe	H6a1a	H	H	17
705	JQ702507.1	JQ702507.1; H3h7; Asia_W_Europe; 14; A93G; A263G; C309CCT	16570	Asia_W_Europe	H3h7	H	H	14
706	JQ702508.1	JQ702508.1; K1a9; Asia_W; 32; A73G; T195C; A263G	16569	Asia_W	K1a9	K	K	32
707	JQ702509.1	JQ702509.1; H6a1a1a; Asia_W_Europe; 21; T239C; A263G; C309CCT	16570	Asia_W_Europe	H6a1a1a	H	H	21
708	JQ702510.1	JQ702510.1; U5b2a1a1a; Asia_W_Europe_C; 29; A73G; C150T; A263G	16569	Asia_W_Europe_C	U5b2a1a1a	U	U5	29
709	JQ702511.1	JQ702511.1; H10a1; Asia_W_Europe; 14; A263G; C309CCT; T310C	16570	Asia_W_Europe	H10a1	H	H	14
710	JQ702512.1	JQ702512.1; K1a9; Asia_W; 33; A73G; T195C; A263G	16569	Asia_W	K1a9	K	K	33
711	JQ702513.1	JQ702513.1; H; Asia_W_Europe; 10; A263G; C309CCT; T310C	16570	Asia_W_Europe	H	H	H	10
712	JQ702514.1	JQ702514.1; H2a2b4; Asia_W_Europe; 8; A263G; C309CCT; T310C	16570	Asia_W_Europe	H2a2b4	H	H	8
713	JQ702515.1	JQ702515.1; J1c2; Asia_W; 31; A73G; G185A; A188G	16570	Asia_W	J1c2	J	J	31
714	JQ702516.1	JQ702516.1; T2b4; Asia_W; 34; A73G; A263G; C315CC	16569	Asia_W	T2b4	T	T	34
715	JQ702517.1	JQ702517.1; K1a+195; Asia_W; 33; A73G; T195C; A263G	16571	Asia_W	K1a+195	K	K	33
716	JQ702518.1	JQ702518.1; H7a1b; Asia_W_Europe; 17; A263G; C309CCT; T310C	16570	Asia_W_Europe	H7a1b	H	H	17
717	JQ702519.1	JQ702519.1; I1a1; Asia_W_SW; 43; A73G; T199C; G203A	16573	Asia_W_SW	I1a1	I	I	43
718	JQ702520.1	JQ702520.1; U2e1d; Asia_S_W_Europe; 40; A73G; T152C; T217C	16573	Asia_S_W_Europe	U2e1d	U	U2	40
719	JQ702521.1	JQ702521.1; K1a4a; Asia_W; 38; A73G; T146C; A263G	16569	Asia_W	K1a4a	K	K	38
720	JQ702522.1	JQ702522.1; H41a; Asia_W_Europe; 15; C262T; A263G; C309CCCT	16572	Asia_W_Europe	H41a	H	H	15
721	JQ702523.1	JQ702523.1; H1bv1; Asia_W_Europe; 13; A263G; C315CC; A750G	16569	Asia_W_Europe	H1bv1	H	H	13
722	JQ702524.1	JQ702524.1; U5b1b1a; Asia_W_Europe_C; 30; A73G; C150T; A263G	16569	Asia_W_Europe_C	U5b1b1a	U	U5	30
723	JQ702525.1	JQ702525.1; J1c3b; Asia_W; 27; A73G; A263G; C295T	16569	Asia_W	J1c3b	J	J	27
724	JQ702526.1	JQ702526.1; HV0d; Asia_W; 17; T72C; T195C; A263G	16570	Asia_W	HV0d	HV	HV	17
725	JQ702527.1	JQ702527.1; H1b1c; Asia_W_Europe; 16; A263G; C315CC; CA522d	16567	Asia_W_Europe	H1b1c	H	H	16
726	JQ702528.1	JQ702528.1; H1c; Asia_W_Europe; 12; A263G; C309CCT; T310C	16570	Asia_W_Europe	H1c	H	H	12
727	JQ702529.1	JQ702529.1; H1b2; Asia_W_Europe; 14; T146C; A263G; C309CCCT	16571	Asia_W_Europe	H1b2	H	H	14
728	JQ702530.1	JQ702530.1; H3h2; Asia_W_Europe; 12; A263G; C315CC; A750G	16569	Asia_W_Europe	H3h2	H	H	12
729	JQ702531.1	JQ702531.1; U5b2b4; Asia_W_Europe_C; 33; A73G; C150T; A263G	16572	Asia_W_Europe_C	U5b2b4	U	U5	33
730	JQ702532.1	JQ702532.1; H1c; Asia_W_Europe; 12; A263G; C309CCT; T310C	16570	Asia_W_Europe	H1c	H	H	12
731	JQ702533.1	JQ702533.1; L3e2a1b; Africa_E; 36; A73G; C150T; T195C	16569	Africa_E	L3e2a1b	L3	L3	36
732	JQ702534.1	JQ702534.1; C1b5b; Asia_NE_America_N_S; 48; A73G; A249d; G255A	16564	Asia_NE_America_N_S	C1b5b	C	C	48
733	JQ702535.1	JQ702535.1; H1a5; Asia_W_Europe; 13; A73G; A263G; C315CC	16569	Asia_W_Europe	H1a5	H	H	13
734	JQ702536.1	JQ702536.1; HV16; Asia_W; 17; A263G; C309CCT; T310C	16570	Asia_W	HV16	HV	HV	17
735	JQ702537.1	JQ702537.1; H4a1a1a; Asia_W_Europe; 19; A73G; A263G; C315CC	16567	Asia_W_Europe	H4a1a1a	H	H	19
736	JQ702538.1	JQ702538.1; J1b6a; Asia_W; 32; A73G; A263G; C295T	16570	Asia_W	J1b6a	J	J	32
737	JQ702539.1	JQ702539.1; H; Asia_W_Europe; 9; A263G; C315CC; A750G	16569	Asia_W_Europe	H	H	H	9
738	JQ702540.1	JQ702540.1; K1a9; Asia_W; 33; A73G; T195C; A263G	16569	Asia_W	K1a9	K	K	33

Supplementary Table S4 Human mitochondrial database (hMITO DB v1.0) metadata[a]

Row	Name (accession no.)	Description	Size	Geo_Region	Haplogroup	Macro_ Haplo	Macro_ Haplo2	Total Variants
739	JQ702541.1	JQ702541.1; K1a; Asia_W; 36; A73G; T146C; A263G	16569	Asia_W	K1a	K	K	36
740	JQ702542.1	JQ702542.1; H1j; Asia_W_Europe; 14; A93G; A263G; C315CC	16567	Asia_W_Europe	H1j	H	H	14
741	JQ702543.1	JQ702543.1; U5b2b1a1; Asia_W_Europe_C; 35; A73G; C150T; T204C	16570	Asia_W_Europe_C	U5b2b1a1	U	U5	35
742	JQ702544.1	JQ702544.1; T2b7a1; Asia_W; 39; A73G; T146C; T152C	16568	Asia_W	T2b7a1	T	T	39
743	JQ702545.1	JQ702545.1; W1b1; Asia_W; 37; A73G; A189G; T195C	16570	Asia_W	W1b1	W	W	37
744	JQ702546.1	JQ702546.1; H4a1a4b2; Asia_W_Europe; 20; T195C; A263G; C315CC	16567	Asia_W_Europe	H4a1a4b2	H	H	20
745	JQ702547.1	JQ702547.1; T2b24a; Asia_W; 38; A73G; A263G; C309CCT	16570	Asia_W	T2b24a	T	T	38
746	JQ702548.1	JQ702548.1; H7a1b; Asia_W_Europe; 13; A263G; C309CCCT; T310C	16571	Asia_W_Europe	H7a1b	H	H	13
747	JQ702549.1	JQ702549.1; U4a2h1; Asia_N_Europe_N; 29; A73G; T195C; A263G	16568	Asia_N_Europe_N	U4a2h1	U	U4	29
748	JQ702550.1	JQ702550.1; H5a1; Asia_W_Europe; 13; A263G; C315CC; C456T	16567	Asia_W_Europe	H5a1	H	H	13
749	JQ702551.1	JQ702551.1; Z1a1a; Asia_C_E; 47; A73G; C151T; T152C	16569	Asia_C_E	Z1a1a	Z	Z	47
750	JQ702552.1	JQ702552.1; U5a1b1f; Asia_W_Europe_C; 31; A73G; A263G; C315CC	16569	Asia_W_Europe_C	U5a1b1f	U	U5	31
751	JQ702553.1	JQ702553.1; J2b1a; Asia_W; 33; A73G; C150T; T152C	16570	Asia_W	J2b1a	J	J	33
752	JQ702554.1	JQ702554.1; J1c8a1; Asia_W; 35; A73G; C150T; G185A	16574	Asia_W	J1c8a1	J	J	35
753	JQ702555.1	JQ702555.1; K1c2; Asia_W; 36; A73G; T146C; T152C	16568	Asia_W	K1c2	K	K	36
754	JQ702556.1	JQ702556.1; T1a1; Asia_W; 36; A73G; T152C; T195C	16570	Asia_W	T1a1	T	T	36
755	JQ702557.1	JQ702557.1; H1ag1b; Asia_W_Europe; 15; A235G; A263G; C309CCT	16570	Asia_W_Europe	H1ag1b	H	H	15
756	JQ702558.1	JQ702558.1; W1c1; Asia_W; 37; A73G; A189G; T195C	16569	Asia_W	W1c1	W	W	37
757	JQ702559.1	JQ702559.1; H1y; Asia_W_Europe; 12; A263G; C315CC; A750G	16569	Asia_W_Europe	H1y	H	H	12
758	JQ702560.1	JQ702560.1; H27; Asia_W_Europe; 13; A215G; A263G; C309CCCT	16571	Asia_W_Europe	H27	H	H	13
759	JQ702561.1	JQ702561.1; J2a1a1; Asia_W; 40; A73G; C150T; T152C	16570	Asia_W	J2a1a1	J	J	40
760	JQ702562.1	JQ702562.1; H; Asia_W_Europe; 10; A73G; A263G; C315CC	16569	Asia_W_Europe	H	H	H	10
761	JQ702563.1	JQ702563.1; J2b1; Asia_W; 32; A73G; C150T; T152C	16570	Asia_W	J2b1	J	J	32
762	JQ702564.1	JQ702564.1; T2b4a; Asia_W; 35; A73G; A263G; C315CC	16569	Asia_W	T2b4a	T	T	35
763	JQ702565.1	JQ702565.1; H1aa1; Asia_W_Europe; 15; A263G; C309CCT; T310C	16571	Asia_W_Europe	H1aa1	H	H	15
764	JQ702566.1	JQ702566.1; H1b2a; Asia_W_Europe; 17; A183G; A263G; C309CCCT	16571	Asia_W_Europe	H1b2a	H	H	17
765	JQ702567.1	JQ702567.1; I1; Asia_W_SW; 40; A73G; T152C; T199C	16573	Asia_W_SW	I1	I	I	40
766	JQ702568.1	JQ702568.1; U5a1a2a1a; Asia_W_Europe_C; 32; A73G; A263G; C309CCT	16572	Asia_W_Europe_C	U5a1a2a1a	U	U5	32
767	JQ702569.1	JQ702569.1; V7a1; Europe_S; 20; T72C; A93G; A263G	16570	Europe_S	V7a1	V	V	20
768	JQ702570.1	JQ702570.1; H1bo; Asia_W_Europe; 19; A263G; T267C; C315CC	16569	Asia_W_Europe	H1bo	H	H	19
769	JQ702571.1	JQ702571.1; H5a5; Asia_W_Europe; 16; T146C; A263G; C309CCT	16570	Asia_W_Europe	H5a5	H	H	16
770	JQ702572.1	JQ702572.1; G2a1; Asia_E; 41; A73G; T152C; A263G	16570	Asia_E	G2a1	G	G	41
771	JQ702573.1	JQ702573.1; X2b+226; Asia_W_America_N; 31; A73G; A153G; T195C	16570	Asia_W_America_N	X2b+226	X	X	31
772	JQ702574.1	JQ702574.1; T2g2a; Asia_W; 40; A73G; A235G; A263G	16570	Asia_W	T2g2a	T	T	40
773	JQ702575.1	JQ702575.1; H1+16189; Asia_W_Europe; 13; A263G; C309CCCT; T310C	16571	Asia_W_Europe	H1+16189	H	H	13
774	JQ702576.1	JQ702576.1; X2b+226; Asia_W_America_N; 29; A73G; A153G; T195C	16569	Asia_W_America_N	X2b+226	X	X	29
775	JQ702577.1	JQ702577.1; H1u; Asia_W_Europe; 13; C151T; T152C; A263G	16569	Asia_W_Europe	H1u	H	H	13
776	JQ702578.1	JQ702578.1; I2e; Asia_W_SW; 36; A73G; T152C; T199C	16573	Asia_W_SW	I2e	I	I	36
777	JQ702579.1	JQ702579.1; H10e1; Asia_W_Europe; 13; A263G; C315CC; A750G	16569	Asia_W_Europe	H10e1	H	H	13
778	JQ702580.1	JQ702580.1; H4b; Asia_W_Europe; 15; A263G; C309CCCT; T310C	16569	Asia_W_Europe	H4b	H	H	15
779	JQ702581.1	JQ702581.1; J1c2; Asia_W; 32; A73G; G185A; A188G	16571	Asia_W	J1c2	J	J	32

Supplementary Table S4 Human mitochondrial database (hMITO DB v1.0) metadata[a]

Row	Name (accession no.)	Description	Size	Geo_Region	Haplogroup	Macro_ Haplo	Macro_ Haplo2	Total Variants
780	JQ702582.1	JQ702582.1; H13a1a1a; Asia_W_Europe; 18; A263G; C309CCCT; T310C	16570	Asia_W_Europe	H13a1a1a	H	H	18
781	JQ702583.1	JQ702583.1; H5m; Asia_W_Europe; 13; T146C; A263G; C315CC	16571	Asia_W_Europe	H5m	H	H	13
782	JQ702584.1	JQ702584.1; H1bb; Asia_W_Europe; 13; T152C; A263G; C315CC	16571	Asia_W_Europe	H1bb	H	H	13
783	JQ702585.1	JQ702585.1; U5b2b4; Asia_W_Europe_C; 31; A73G; C150T; A263G	16571	Asia_W_Europe_C	U5b2b4	U	U5	31
784	JQ702586.1	JQ702586.1; K1a9; Asia_W; 34; A73G; T152C; T195C	16569	Asia_W	K1a9	K	K	34
785	JQ702587.1	JQ702587.1; U5b1b1+@16192; Asia_W_Europe_C; 32; A73G; C150T; T152C	16570	Asia_W_Europe_C	U5b1b1+@1619	U	U5	32
786	JQ702588.1	JQ702588.1; U5b2a1a1; Asia_W_Europe_C; 29; A73G; C150T; A263G	16569	Asia_W_Europe_C	U5b2a1a1	U	U5	29
787	JQ702589.1	JQ702589.1; H79a; Asia_W_Europe; 11; A263G; C315CC; A750G	16569	Asia_W_Europe	H79a	H	H	11
788	JQ702590.1	JQ702590.1; H1bs; Asia_W_Europe; 16; A93G; A263G; C309CCT	16570	Asia_W_Europe	H1bs	H	H	16
789	JQ702591.1	JQ702591.1; H1aj1a; Asia_W_Europe; 19; T204C; G207A; A263G	16569	Asia_W_Europe	H1aj1a	H	H	19
790	JQ702592.1	JQ702592.1; A2ab; Asia_NE_America_N; 37; C64T; A73G; T146C	16568	Asia_NE_America_N	A2ab	A	A	37
791	JQ702593.1	JQ702593.1; J2b1a; Asia_W; 34; A73G; C150T; T152C	16571	Asia_W	J2b1a	J	J	34
792	JQ702594.1	JQ702594.1; T2a1a; Asia_W; 38; A73G; A263G; A297G	16569	Asia_W	T2a1a	T	T	38
793	JQ702595.1	JQ702595.1; C1b5b; Asia_NE_America_N_S; 45; A73G; A249d; A263G	16564	Asia_NE_America_N_S	C1b5b	C	C	45
794	JQ702596.1	JQ702596.1; HV17a; Asia_W; 16; A263G; C315CC; C549T	16569	Asia_W	HV17a	HV	HV	16
795	JQ702597.1	JQ702597.1; H1e1a; Asia_W_Europe; 17; A263G; C315CC; C520T	16570	Asia_W_Europe	H1e1a	H	H	17
796	JQ702598.1	JQ702598.1; H1a; Asia_W_Europe; 13; A73G; T146C; A263G	16569	Asia_W_Europe	H1a	H	H	13
797	JQ702599.1	JQ702599.1; H10e3a; Asia_W_Europe; 13; A263G; C309CCT; T310C	16570	Asia_W_Europe	H10e3a	H	H	13
798	JQ702600.1	JQ702600.1; L1c3b1b; Africa_C; 86; A73G; C151T; T152C	16566	Africa_C	L1c3b1b	L1	L1	86
799	JQ702601.1	JQ702601.1; H7b; Asia_W_Europe; 12; T152C; A263G; C315CC	16569	Asia_W_Europe	H7b	H	H	12
800	JQ702602.1	JQ702602.1; H1a1a; Asia_W_Europe; 15; A73G; A263G; C315CC	16569	Asia_W_Europe	H1a1a	H	H	15
801	JQ702603.1	JQ702603.1; H3c3; Asia_W_Europe; 15; G260A; A263G; C315CC	16569	Asia_W_Europe	H3c3	H	H	15
802	JQ702604.1	JQ702604.1; T2b4c; Asia_W; 38; A73G; T152C; A263G	16570	Asia_W	T2b4c	T	T	38
803	JQ702605.1	JQ702605.1; J2a1a1e; Asia_W; 42; A73G; C150T; T152C	16567	Asia_W	J2a1a1e	J	J	42
804	JQ702606.1	JQ702606.1; H6a1a10; Asia_W_Europe; 19; T239C; A263G; C309CCCT	16571	Asia_W_Europe	H6a1a10	H	H	19
805	JQ702607.1	JQ702607.1; H1c20; Asia_W_Europe; 14; A263G; C309CCT; T310C	16570	Asia_W_Europe	H1c20	H	H	14
806	JQ702608.1	JQ702608.1; H1e4a; Asia_W_Europe; 13; A263G; C315CC; A750G	16569	Asia_W_Europe	H1e4a	H	H	13
807	JQ702609.1	JQ702609.1; J1c13; Asia_W; 32; A73G; G185A; A189G	16569	Asia_W	J1c13	J	J	32
808	JQ702610.1	JQ702610.1; K1a4a1b; Asia_W; 44; A73G; T152C; A263G	16574	Asia_W	K1a4a1b	K	K	44
809	JQ702611.1	JQ702611.1; T2a1a; Asia_W; 36; A73G; A263G; C309CCT	16570	Asia_W	T2a1a	T	T	36
810	JQ702612.1	JQ702612.1; U6a1a1; Asia_SW_Africa_N; 31; A73G; A263G; C309CCT	16570	Asia_SW_Africa_N	U6a1a1	U	U6	31
811	JQ702613.1	JQ702613.1; H4a1a2a1; Asia_W_Europe; 20; A263G; C315CC; CA522d	16567	Asia_W_Europe	H4a1a2a1	H	H	20
812	JQ702614.1	JQ702614.1; H5c1; Asia_W_Europe; 12; A263G; C309CCT; T310C	16570	Asia_W_Europe	H5c1	H	H	12
813	JQ702615.1	JQ702615.1; H5a3a; Asia_W_Europe; 16; A263G; C309CCT; T310C	16570	Asia_W_Europe	H5a3a	H	H	16
814	JQ702616.1	JQ702616.1; X2c1a; Asia_W_America_N; 33; A73G; A153G; T195C	16570	Asia_W_America_N	X2c1a	X	X	33
815	JQ702617.1	JQ702617.1; L1c5; Africa_C; 85; A73G; C151T; T152C	16558	Africa_C	L1c5	L1	L1	85
816	JQ702618.1	JQ702618.1; K1c1; Asia_W; 35; A73G; T146C; T152C	16570	Asia_W	K1c1	K	K	35
817	JQ702619.1	JQ702619.1; HV0a1a; Asia_W; 23; T72C; A200G; A263G	16569	Asia_W	HV0a1a	HV	HV	23
818	JQ702620.1	JQ702620.1; J1c1a; Asia_W; 34; A73G; A93G; A210G	16569	Asia_W	J1c1a	J	J	34
819	JQ702621.1	JQ702621.1; H2; Asia_W_Europe; 14; A263G; C309CCT; T310C	16570	Asia_W_Europe	H2	H	H	14
820	JQ702622.1	JQ702622.1; J2a1a1a2; Asia_W; 43; A73G; C150T; T152C	16570	Asia_W	J2a1a1a2	J	J	43

Supplementary Table S4 Human mitochondrial database (hMITO DB v1.0) metadata[a]

Row	Name (accession no.)	Description	Size	Geo_Region	Haplogroup	Macro_ Haplo	Macro_ Haplo2	Total Variants
821	JQ702623.1	JQ702623.1; H1ak; Asia_W_Europe; 11; A263G; C309CCCT; T310C	16571	Asia_W_Europe	H1ak	H	H	11
822	JQ702624.1	JQ702624.1; K1a1b2b; Asia_W; 39; A73G; A189G; T199C	16569	Asia_W	K1a1b2b	K	K	39
823	JQ702625.1	JQ702625.1; U5b2c2a; Asia_W_Europe_C; 34; A73G; C150T; T195C	16571	Asia_W_Europe_C	U5b2c2a	U	U5	34
824	JQ702626.1	JQ702626.1; L2b3a; Africa_W_C; 73; A73G; T146C; C150T	16570	Africa_W_C	L2b3a	L2	L2	73
825	JQ702627.1	JQ702627.1; H14b; Asia_W_Europe; 14; G143A; T152C; A263G	16569	Asia_W_Europe	H14b	H	H	14
826	JQ702628.1	JQ702628.1; H1bb; Asia_W_Europe; 13; T152C; A263G; C315CC	16569	Asia_W_Europe	H1bb	H	H	13
827	JQ702629.1	JQ702629.1; H3; Asia_W_Europe; 16; T195C; A263G; C315CC	16570	Asia_W_Europe	H3	H	H	16
828	JQ702630.1	JQ702630.1; J1c3b2; Asia_W; 29; A73G; G185A; A263G	16569	Asia_W	J1c3b2	J	J	29
829	JQ702631.1	JQ702631.1; H4a1c1a; Asia_W_Europe; 17; A263G; C315CC; CA522d	16567	Asia_W_Europe	H4a1c1a	H	H	17
830	JQ702632.1	JQ702632.1; K1c2; Asia_W; 36; A73G; T146C; T152C	16568	Asia_W	K1c2	K	K	36
831	JQ702633.1	JQ702633.1; X2c1e; Asia_W_America_N; 31; A73G; A153G; T195C	16569	Asia_W_America_N	X2c1e	X	X	31
832	JQ702634.1	JQ702634.1; H1bc; Asia_W_Europe; 16; T152C; A263G; C315CC	16571	Asia_W_Europe	H1bc	H	H	16
833	JQ702635.1	JQ702635.1; H3h7; Asia_W_Europe; 15; A93G; A263G; C309CCCT	16571	Asia_W_Europe	H3h7	H	H	15
834	JQ702636.1	JQ702636.1; K1b2a2a; Asia_W; 37; A73G; T146C; T195C	16573	Asia_W	K1b2a2a	K	K	37
835	JQ702637.1	JQ702637.1; V1a1; Europe_S; 17; A263G; C309CCCT; T310C	16571	Europe_S	V1a1	V	V	17
836	JQ702638.1	JQ702638.1; W1e1a; Asia_W; 37; A73G; A189G; T195C	16569	Asia_W	W1e1a	W	W	37
837	JQ702639.1	JQ702639.1; H1a3a; Asia_W_Europe; 15; A73G; A263G; C315CC	16569	Asia_W_Europe	H1a3a	H	H	15
838	JQ702640.1	JQ702640.1; K2a7; Asia_W; 36; A73G; T146C; T152C	16569	Asia_W	K2a7	K	K	36
839	JQ702641.1	JQ702641.1; H10e1; Asia_W_Europe; 12; A263G; C315CC; A750G	16569	Asia_W_Europe	H10e1	H	H	12
840	JQ702642.1	JQ702642.1; T2f1a1; Asia_W; 42; A73G; T195C; A263G	16560	Asia_W	T2f1a1	T	T	42
841	JQ702643.1	JQ702643.1; T1a1f; Asia_W; 36; A73G; T152C; T195C	16570	Asia_W	T1a1f	T	T	36
842	JQ702644.1	JQ702644.1; H15a1; Asia_W_Europe; 13; T55C; T57C; A263G	16569	Asia_W_Europe	H15a1	H	H	13
843	JQ702645.1	JQ702645.1; T2b; Asia_W; 34; A73G; A263G; C315CC	16569	Asia_W	T2b	T	T	34
844	JQ702646.1	JQ702646.1; J1c3a1; Asia_W; 32; A73G; G228A; A263G	16579	Asia_W	J1c3a1	J	J	32
845	JQ702647.1	JQ702647.1; I3d1; Asia_W_SW; 42; A73G; T152C; A183G	16579	Asia_W_SW	I3d1	I	I	42
846	JQ702648.1	JQ702648.1; B2d; Asia_SE_E_America_N_S; 35; A73G; A263G; C309CCT	16560	Asia_SE_E_America_N_S	B2d	B	B	35
847	JQ702649.1	JQ702649.1; H1e1b1a; Asia_W_Europe; 15; A263G; C309CCCT; T310C	16571	Asia_W_Europe	H1e1b1a	H	H	15
848	JQ702650.1	JQ702650.1; H1a3a; Asia_W_Europe; 15; A73G; T146C; A263G	16570	Asia_W_Europe	H1a3a	H	H	15
849	JQ702651.1	JQ702651.1; C1b8; Asia_NE_America_N_S; 52; A73G; G143A; T195C	16565	Asia_NE_America_N_S	C1b8	C	C	52
850	JQ702652.1	JQ702652.1; H3+16311; Asia_W_Europe; 12; A263G; C315CC; A750G	16569	Asia_W_Europe	H3+16311	H	H	12
851	JQ702653.1	JQ702653.1; H5a1c1a; Asia_W_Europe; 18; A263G; C315CC; C456T	16567	Asia_W_Europe	H5a1c1a	H	H	18
852	JQ702654.1	JQ702654.1; U2e1; Asia_S_W_Europe; 41; A73G; T152C; A200G	16573	Asia_S_W_Europe	U2e1	U	U2	41
853	JQ702655.1	JQ702655.1; I1c1a; Asia_W_SW; 44; A73G; T199C; T204C	16572	Asia_W_SW	I1c1a	I	I	44
854	JQ702656.1	JQ702656.1; H5a6a; Asia_W_Europe; 17; T152C; A263G; C315CC	16569	Asia_W_Europe	H5a6a	H	H	17
855	JQ702657.1	JQ702657.1; H1q1a; Asia_W_Europe; 13; A263G; C315CC; A750G	16569	Asia_W_Europe	H1q1a	H	H	13
856	JQ702658.1	JQ702658.1; H13a1a; Asia_W_Europe; 13; A263G; C309CCCT; T310C	16571	Asia_W_Europe	H13a1a	H	H	13
857	JQ702659.1	JQ702659.1; L2a1c6; Africa_W_C; 58; A73G; G143A; T146C	16568	Africa_W_C	L2a1c6	L2	L2	58
858	JQ702660.1	JQ702660.1; H4a1a4b; Asia_W_Europe; 20; T195C; A263G; C315CC	16568	Asia_W_Europe	H4a1a4b	H	H	20
859	JQ702661.1	JQ702661.1; B2g2; Asia_SE_E_America_N_S; 37; G9A; A73G; C114G	16560	Asia_SE_E_America_N_S	B2g2	B	B	37
860	JQ702662.1	JQ702662.1; H34; Asia_W_Europe; 13; T152C; A263G; C315CC	16569	Asia_W_Europe	H34	H	H	13
861	JQ702663.1	JQ702663.1; U2e1b1; Asia_S_W_Europe; 38; A73G; T152C; T217C	16569	Asia_S_W_Europe	U2e1b1	U	U2	38

Supplementary Table S4 Human mitochondrial database (hMITO DB v1.0) metadata[a]

Row	Name (accession no.)	Description	Size	Geo_Region	Haplogroup	Macro_ Haplo	Macro_ Haplo2	Total Variants
862	JQ702664.1	JQ702664.1; M7c1c; Asia_E_SE; 43; A73G; T146C; T152C	16568	Asia_E_SE	M7c1c	M	M7	43
863	JQ702665.1	JQ702665.1; K1a1b2a1a; Asia_W; 42; A73G; T152C; A263G	16571	Asia_W	K1a1b2a1a	K	K	42
864	JQ702666.1	JQ702666.1; T2b5; Asia_W; 38; A73G; A93G; A263G	16570	Asia_W	T2b5	T	T	38
865	JQ702667.1	JQ702667.1; V3a1; Europe_S; 17; T72C; A263G; C315CC	16569	Europe_S	V3a1	V	V	17
866	JQ702668.1	JQ702668.1; B2a1a1; Asia_SE_E_America_N_S; 31; A73G; A263G; C309CCCT	16562	Asia_SE_E_America_N_S	B2a1a1	B	B	31
867	JQ702669.1	JQ702669.1; V13; Europe_S; 18; T72C; A263G; C309CCCT	16571	Europe_S	V13	V	V	18
868	JQ702670.1	JQ702670.1; H5a1h; Asia_W_Europe; 13; A263G; C315CC; C456T	16567	Asia_W_Europe	H5a1h	H	H	13
869	JQ702671.1	JQ702671.1; K1a1b1a; Asia_W; 39; A73G; C114T; A263G	16571	Asia_W	K1a1b1a	K	K	39
870	JQ702672.1	JQ702672.1; K2b1b; Asia_W; 39; A73G; T146C; T195C	16570	Asia_W	K2b1b	K	K	39
871	JQ702673.1	JQ702673.1; R1a1a2; Asia_S_SE; 38; A73G; A263G; C295A	16573	Asia_S_SE	R1a1a2	R	R1	38
872	JQ702674.1	JQ702674.1; K1a15; Asia_W; 34; A73G; A93G; T195C	16573	Asia_W	K1a15	K	K	34
873	JQ702675.1	JQ702675.1; K1a11a; Asia_W; 39; A16T; A73G; C150T	16561	Asia_W	K1a11a	K	K	39
874	JQ702676.1	JQ702676.1; K1a1b1a; Asia_W; 36; A73G; A263G; C315CC	16569	Asia_W	K1a1b1a	K	K	36
875	JQ702677.1	JQ702677.1; U3a1b; Africa_NE_Asia_W; 31; A73G; C150T; A263G	16570	Africa_NE_Asia_W	U3a1b	U	U3	31
876	JQ702678.1	JQ702678.1; R0a1; Africa_NE_Asia_W; 30; C64T; A189G; T204C	16569	Africa_NE_Asia_W	R0a1	R	R0	30
877	JQ702679.1	JQ702679.1; H1c7; Asia_W_Europe; 12; A263G; C315CC; T477C	16573	Asia_W_Europe	H1c7	H	H	12
878	JQ702680.1	JQ702680.1; T1a1h; Asia_W; 37; A73G; T152C; T195C	16571	Asia_W	T1a1h	T	T	37
879	JQ702681.1	JQ702681.1; U3a1b; Africa_NE_Asia_W; 30; A73G; C150T; A263G	16569	Africa_NE_Asia_W	U3a1b	U	U3	30
880	JQ702682.1	JQ702682.1; H; Asia_W_Europe; 11; A263G; C315CC; A750G	16569	Asia_W_Europe	H	H	H	11
881	JQ702683.1	JQ702683.1; H1c; Asia_W_Europe; 13; T195C; A263G; C315CC	16569	Asia_W_Europe	H1c	H	H	13
882	JQ702684.1	JQ702684.1; J1b2; Asia_W; 32; A73G; C150T; T152C	16569	Asia_W	J1b2	J	J	32
883	JQ702685.1	JQ702685.1; J1c1; Asia_W; 32; A73G; G185A; G228A	16572	Asia_W	J1c1	J	J	32
884	JQ702686.1	JQ702686.1; J1c3e2; Asia_W; 33; A73G; G185A; G228A	16570	Asia_W	J1c3e2	J	J	33
885	JQ702687.1	JQ702687.1; H3ar; Asia_W_Europe; 13; A263G; C309CCT; T310C	16570	Asia_W_Europe	H3ar	H	H	13
886	JQ702688.1	JQ702688.1; U5b2c2b; Asia_W_Europe_C; 36; A73G; C150T; T195C	16571	Asia_W_Europe_C	U5b2c2b	U	U5	36
887	JQ702689.1	JQ702689.1; V21; Europe_S; 16; A263G; C309CCT; T310C	16572	Europe_S	V21	V	V	16
888	JQ702690.1	JQ702690.1; U4c2a; Asia_N_Europe_N; 32; A73G; C150T; T195C	16569	Asia_N_Europe_N	U4c2a	U	U4	32
889	JQ702691.1	JQ702691.1; J1c3; Asia_W; 28; A73G; G185A; G228A	16569	Asia_W	J1c3	J	J	28
890	JQ702692.1	JQ702692.1; H4a1c1a; Asia_W_Europe; 19; A263G; C315CC; CA522d	16567	Asia_W_Europe	H4a1c1a	H	H	19
891	JQ702693.1	JQ702693.1; K1a3a2; Asia_W; 38; A73G; A263G; C309CCT	16570	Asia_W	K1a3a2	K	K	38
892	JQ702694.1	JQ702694.1; L2b1a3; Africa_W_C; 73; A73G; C150T; T152C	16568	Africa_W_C	L2b1a3	L2	L2	73
893	JQ702695.1	JQ702695.1; J1b1a1a; Asia_W; 39; A73G; C242T; A263G	16569	Asia_W	J1b1a1a	J	J	39
894	JQ702696.1	JQ702696.1; U5a1a1c; Asia_W_Europe_C; 28; A73G; A263G; C315CC	16569	Asia_W_Europe_C	U5a1a1c	U	U5	28
895	JQ702697.1	JQ702697.1; H31b; Asia_W_Europe; 15; T195C; A263G; C315CC	16569	Asia_W_Europe	H31b	H	H	15
896	JQ702698.1	JQ702698.1; H1a3c; Asia_W_Europe; 13; A73G; A263G; C315CC	16569	Asia_W_Europe	H1a3c	H	H	13
897	JQ702699.1	JQ702699.1; HV0e; Asia_W; 18; T72C; T195C; T204C	16570	Asia_W	HV0e	HV	HV	18
898	JQ702700.1	JQ702700.1; K1a4a1a+195; Asia_W; 37; A73G; T195C; A263G	16573	Asia_W	K1a4a1a+195	K	K	37
899	JQ702701.1	JQ702701.1; H1as; Asia_W_Europe; 11; A263G; C309CCT; T310C	16570	Asia_W_Europe	H1as	H	H	11
900	JQ702702.1	JQ702702.1; J2b1a3; Asia_W; 35; A73G; C150T; T152C	16570	Asia_W	J2b1a3	J	J	35
901	JQ702703.1	JQ702703.1; H1b; Asia_W_Europe; 15; A263G; C309CCCT; T310C	16571	Asia_W_Europe	H1b	H	H	15
902	JQ702704.1	JQ702704.1; T2; Asia_W; 32; A73G; A263G; C315CC	16569	Asia_W	T2	T	T	32

Supplementary Table S4 Human mitochondrial database (hMITO DB v1.0) metadata[a]

Row	Name (accession no.)	Description	Size	Geo_Region	Haplogroup	Macro_ Haplo	Macro_ Haplo2	Total Variants
903	JQ702705.1	JQ702705.1; K1a3; Asia_W; 33; A73G; A263G; C315CC	16571	Asia_W	K1a3	K	K	33
904	JQ702706.1	JQ702706.1; J1c2b3; Asia_W; 34; T59C; A73G; G185A	16569	Asia_W	J1c2b3	J	J	34
905	JQ702707.1	JQ702707.1; H28a; Asia_W_Europe; 13; C186A; A263G; C315CC	16569	Asia_W_Europe	H28a	H	H	13
906	JQ702708.1	JQ702708.1; H6a1b2; Asia_W_Europe; 16; T239C; A263G; C315CC	16569	Asia_W_Europe	H6a1b2	H	H	16
907	JQ702709.1	JQ702709.1; H1b1+16362; Asia_W_Europe; 14; A263G; C315CC; CA522d	16567	Asia_W_Europe	H1b1+16362	H	H	14
908	JQ702710.1	JQ702710.1; T2g2a; Asia_W; 39; A73G; A235G; A263G	16570	Asia_W	T2g2a	T	T	39
909	JQ702711.1	JQ702711.1; U5b2a1b; Asia_W_Europe_C; 31; A73G; C150T; T152C	16569	Asia_W_Europe_C	U5b2a1b	U	U5	31
910	JQ702712.1	JQ702712.1; U5b2a1a2; Asia_W_Europe_C; 33; A73G; C150T; A263G	16572	Asia_W_Europe_C	U5b2a1a2	U	U5	33
911	JQ702713.1	JQ702713.1; U5b2b; Asia_W_Europe_C; 28; A73G; C150T; A263G	16569	Asia_W_Europe_C	U5b2b	U	U5	28
912	JQ702714.1	JQ702714.1; U5a1b1h; Asia_W_Europe_C; 28; A73G; A263G; C315CC	16567	Asia_W_Europe_C	U5a1b1h	U	U5	28
913	JQ702715.1	JQ702715.1; J1c2; Asia_W; 32; A73G; G185A; A188G	16570	Asia_W	J1c2	J	J	32
914	JQ702716.1	JQ702716.1; T1a1; Asia_W; 37; A73G; T152C; T195C	16570	Asia_W	T1a1	T	T	37
915	JQ702717.1	JQ702717.1; K1a24a; Asia_W; 37; A73G; C150T; T195C	16568	Asia_W	K1a24a	K	K	37
916	JQ702718.1	JQ702718.1; N1b1a6; Africa_NE_Asia_W; 38; A73G; T152C; A263G	16567	Africa_NE_Asia_W	N1b1a6	N	N1	38
917	JQ702719.1	JQ702719.1; H1ae2; Asia_W_Europe; 16; A263G; C315CC; A750G	16569	Asia_W_Europe	H1ae2	H	H	16
918	JQ702720.1	JQ702720.1; U5b2b1a1; Asia_W_Europe_C; 34; A73G; C150T; A263G	16570	Asia_W_Europe_C	U5b2b1a1	U	U5	34
919	JQ702721.1	JQ702721.1; V19; Europe_S; 16; T72C; C150T; A263G	16570	Europe_S	V19	V	V	16
920	JQ702722.1	JQ702722.1; K1c1g; Asia_W; 35; A73G; T146C; T152C	16568	Asia_W	K1c1g	K	K	35
921	JQ702723.1	JQ702723.1; V10a; Europe_S; 18; T72C; A263G; C309CCT	16570	Europe_S	V10a	V	V	18
922	JQ702724.1	JQ702724.1; J1c2m1; Asia_W; 34; A73G; G185A; A188G	16570	Asia_W	J1c2m1	J	J	34
923	JQ702725.1	JQ702725.1; H6a1b3a; Asia_W_Europe; 19; T204C; T239C; A263G	16570	Asia_W_Europe	H6a1b3a	H	H	19
924	JQ702726.1	JQ702726.1; H1u; Asia_W_Europe; 13; A263G; C315CC; A750G	16569	Asia_W_Europe	H1u	H	H	13
925	JQ702727.1	JQ702727.1; J1c3c2; Asia_W; 30; A73G; G185A; G228A	16569	Asia_W	J1c3c2	J	J	30
926	JQ702728.1	JQ702728.1; T1a1k; Asia_W; 36; A73G; T152C; T195C	16570	Asia_W	T1a1k	T	T	36
927	JQ702729.1	JQ702729.1; H+152; Asia_W_Europe; 13; T152C; A263G; C315CC	16569	Asia_W_Europe	H+152	H	H	13
928	JQ702730.1	JQ702730.1; U5a1a1; Asia_W_Europe_C; 27; A73G; A263G; C315CC	16569	Asia_W_Europe_C	U5a1a1	U	U5	27
929	JQ702731.1	JQ702731.1; T2e1; Asia_W; 40; C41T; A73G; C150T	16570	Asia_W	T2e1	T	T	40
930	JQ702732.1	JQ702732.1; K1a1b1; Asia_W; 34; A73G; A263G; C315CC	16569	Asia_W	K1a1b1	K	K	34
931	JQ702733.1	JQ702733.1; H3+152; Asia_W_Europe; 14; T152C; A263G; C315CC	16571	Asia_W_Europe	H3+152	H	H	14
932	JQ702734.1	JQ702734.1; X2c1a; Asia_W_America_N; 31; A73G; A153G; T195C	16569	Asia_W_America_N	X2c1a	X	X	31
933	JQ702735.1	JQ702735.1; U5a1f1a1; Asia_W_Europe_C; 32; A73G; T199C; A263G	16569	Asia_W_Europe_C	U5a1f1a1	U	U5	32
934	JQ702736.1	JQ702736.1; H4a1a; Asia_W_Europe; 18; A263G; C309CCT; T310C	16568	Asia_W_Europe	H4a1a	H	H	18
935	JQ702737.1	JQ702737.1; T2b9; Asia_W; 37; A73G; C150T; A263G	16569	Asia_W	T2b9	T	T	37
936	JQ702738.1	JQ702738.1; H1c3; Asia_W_Europe; 15; T195C; A257G; A263G	16570	Asia_W_Europe	H1c3	H	H	15
937	JQ702739.1	JQ702739.1; X2d1; Asia_W_America_N; 34; A73G; T195C; T204C	16567	Asia_W_America_N	X2d1	X	X	34
938	JQ702740.1	JQ702740.1; K1a10; Asia_W; 35; A73G; T195C; A263G	16573	Asia_W	K1a10	K	K	35
939	JQ702741.1	JQ702741.1; J1c2c2; Asia_W; 34; A73G; T146C; G185A	16569	Asia_W	J1c2c2	J	J	34
940	JQ702742.1	JQ702742.1; J1c2a3; Asia_W; 33; A73G; G185A; A188G	16570	Asia_W	J1c2a3	J	J	33
941	JQ702743.1	JQ702743.1; U5b1+16189+@16192; Asia_W_Europe_C; 31; T60TT; G71d; A73G	16571	Asia_W_Europe_C	U5b1+16189+@	U	U5	31
942	JQ702744.1	JQ702744.1; K1a4a1; Asia_W; 34; A73G; C150T; A263G	16569	Asia_W	K1a4a1	K	K	34
943	JQ702745.1	JQ702745.1; K1b1a1; Asia_W; 39; A73G; T152C; A263G	16570	Asia_W	K1b1a1	K	K	39

Supplementary Table S4 Human mitochondrial database (hMITO DB v1.0) metadata[a]

Row	Name (accession no.)	Description	Size	Geo_Region	Haplogroup	Macro_ Haplo	Macro_ Haplo2	Total Variants
944	JQ702746.1	JQ702746.1; U5a2b1; Asia_W_Europe_C; 26; A73G; A263G; C315CC	16570	Asia_W_Europe_C	U5a2b1	U	U5	26
945	JQ702747.1	JQ702747.1; J1c3a1; Asia_W; 32; A73G; G228A; A263G	16570	Asia_W	J1c3a1	J	J	32
946	JQ702748.1	JQ702748.1; K1a2a1; Asia_W; 35; A73G; A263G; C309CCCT	16571	Asia_W	K1a2a1	K	K	35
947	JQ702749.1	JQ702749.1; W5a1a; Asia_W; 42; A73G; T152C; A189G	16570	Asia_W	W5a1a	W	W	42
948	JQ702750.1	JQ702750.1; A2aa; Asia_NE_America_N; 38; C64T; A73G; T146C	16568	Asia_NE_America_N	A2aa	A	A	38
949	JQ702751.1	JQ702751.1; H1a; Asia_W_Europe; 14; A73G; T146C; A263G	16569	Asia_W_Europe	H1a	H	H	14
950	JQ702752.1	JQ702752.1; K1b1a1b; Asia_W; 44; A73G; T152C; T199C	16570	Asia_W	K1b1a1b	K	K	44
951	JQ702753.1	JQ702753.1; H1a3a; Asia_W_Europe; 15; A73G; A263G; C309CCT	16570	Asia_W_Europe	H1a3a	H	H	15
952	JQ702754.1	JQ702754.1; K2b1a1; Asia_W; 36; A73G; T146C; A263G	16569	Asia_W	K2b1a1	K	K	36
953	JQ702755.1	JQ702755.1; K1a1b1a; Asia_W; 37; A73G; C114T; A263G	16569	Asia_W	K1a1b1a	K	K	37
954	JQ702756.1	JQ702756.1; H4a1a1a; Asia_W_Europe; 19; A73G; T146C; A263G	16568	Asia_W_Europe	H4a1a1a	H	H	19
955	JQ702757.1	JQ702757.1; H1; Asia_W_Europe; 11; A263G; C315CC; A750G	16569	Asia_W_Europe	H1	H	H	11
956	JQ702758.1	JQ702758.1; J2b1a; Asia_W; 34; A73G; C150T; T152C	16570	Asia_W	J2b1a	J	J	34
957	JQ702759.1	JQ702759.1; U8a; Asia_W_Europe ; 31; A73G; A263G; T282C	16572	Asia_W_Europe	U8a	U	U8	31
958	JQ702760.1	JQ702760.1; K1a4a1f1; Asia_W; 40; A73G; T152C; T217C	16569	Asia_W	K1a4a1f1	K	K	40
959	JQ702761.1	JQ702761.1; U8a1a2; Asia_W_Europe ; 30; A73G; A263G; T282C	16570	Asia_W_Europe	U8a1a2	U	U8	30
960	JQ702762.1	JQ702762.1; H3g4; Asia_W_Europe; 17; T152C; A263G; C309CCT	16570	Asia_W_Europe	H3g4	H	H	17
961	JQ702763.1	JQ702763.1; H1b1e1; Asia_W_Europe; 17; A263G; C315CC; A750G	16569	Asia_W_Europe	H1b1e1	H	H	17
962	JQ702764.1	JQ702764.1; H3; Asia_W_Europe; 10; A263G; C315CC; A750G	16569	Asia_W_Europe	H3	H	H	10
963	JQ702765.1	JQ702765.1; A2v1b; Asia_NE_America_N; 40; C64T; A73G; T146C	16568	Asia_NE_America_N	A2v1b	A	A	40
964	JQ702766.1	JQ702766.1; H7h; Asia_W_Europe; 12; A263G; C315CC; A750G	16569	Asia_W_Europe	H7h	H	H	12
965	JQ702767.1	JQ702767.1; H8b; Asia_W_Europe; 13; T146C; A263G; C315CC	16569	Asia_W_Europe	H8b	H	H	13
966	JQ702768.1	JQ702768.1; H61; Asia_W_Europe; 12; A263G; C315CC; A750G	16569	Asia_W_Europe	H61	H	H	12
967	JQ702769.1	JQ702769.1; H3as; Asia_W_Europe; 11; A263G; C315CC; A750G	16569	Asia_W_Europe	H3as	H	H	11
968	JQ702770.1	JQ702770.1; H1aj1a; Asia_W_Europe; 17; G207A; A263G; C315CC	16569	Asia_W_Europe	H1aj1a	H	H	17
969	JQ702771.1	JQ702771.1; K2a11; Asia_W; 38; A73G; G143A; T146C	16569	Asia_W	K2a11	K	K	38
970	JQ702772.1	JQ702772.1; J2a1a1a2; Asia_W; 41; A73G; C150T; T152C	16570	Asia_W	J2a1a1a2	J	J	41
971	JQ702773.1	JQ702773.1; K1a4a1a1; Asia_W; 39; A73G; T195C; A263G	16571	Asia_W	K1a4a1a1	K	K	39
972	JQ702774.1	JQ702774.1; H3ad; Asia_W_Europe; 11; A263G; C315CC; A750G	16569	Asia_W_Europe	H3ad	H	H	11
973	JQ702775.1	JQ702775.1; U5a1b1; Asia_W_Europe_C; 28; A73G; A263G; C315CC	16569	Asia_W_Europe_C	U5a1b1	U	U5	28
974	JQ702776.1	JQ702776.1; J1c5c1; Asia_W; 35; A73G; G185A; G228A	16567	Asia_W	J1c5c1	J	J	35
975	JQ702777.1	JQ702777.1; U4c1; Asia_N_Europe_N; 37; A73G; T195C; A263G	16571	Asia_N_Europe_N	U4c1	U	U4	37
976	JQ702778.1	JQ702778.1; HV15; Asia_W; 18; A200G; A263G; C309CCT	16570	Asia_W	HV15	HV	HV	18
977	JQ702779.1	JQ702779.1; K1a4a1b1; Asia_W; 38; A73G; A263G; C309CCT	16570	Asia_W	K1a4a1b1	K	K	38
978	JQ702780.1	JQ702780.1; K1a1b1a; Asia_W; 38; A73G; C114T; A263G	16569	Asia_W	K1a1b1a	K	K	38
979	JQ702781.1	JQ702781.1; K1a1b1; Asia_W; 36; A73G; C114T; A263G	16569	Asia_W	K1a1b1	K	K	36
980	JQ702782.1	JQ702782.1; K1a1b1d; Asia_W; 35; A73G; A263G; C315CC	16569	Asia_W	K1a1b1d	K	K	35
981	JQ702783.1	JQ702783.1; U4b2a; Asia_N_Europe_N; 32; A73G; T195C; A263G	16569	Asia_N_Europe_N	U4b2a	U	U4	32
982	JQ702784.1	JQ702784.1; T1a1; Asia_W; 37; A73G; C151T; T152C	16570	Asia_W	T1a1	T	T	37
983	JQ702785.1	JQ702785.1; K1a1b1b; Asia_W; 38; A73G; C114T; A189G	16569	Asia_W	K1a1b1b	K	K	38
984	JQ702786.1	JQ702786.1; H31; Asia_W_Europe; 14; T146C; T195C; A263G	16570	Asia_W_Europe	H31	H	H	14

Supplementary Table S4 Human mitochondrial database (hMITO DB v1.0) metadata[a]

Row	Name (accession no.)	Description	Size	Geo_Region	Haplogroup	Macro_ Haplo	Macro_ Haplo2	Total Variants
985	JQ702787.1	JQ702787.1; H16; Asia_W_Europe; 11; A263G; C315CC; A750G	16569	Asia_W_Europe	H16	H	H	11
986	JQ702788.1	JQ702788.1; J1c3g; Asia_W; 32; A73G; G185A; T195C	16569	Asia_W	J1c3g	J	J	32
987	JQ702789.1	JQ702789.1; H3b+16129; Asia_W_Europe; 14; A263G; C309CCCT; T310C	16573	Asia_W_Europe	H3b+16129	H	H	14
988	JQ702790.1	JQ702790.1; U5a1b1; Asia_W_Europe_C; 27; A73G; A263G; C309CCT	16570	Asia_W_Europe_C	U5a1b1	U	U5	27
989	JQ702791.1	JQ702791.1; U5b2c2; Asia_W_Europe_C; 32; A73G; C150T; A263G	16571	Asia_W_Europe_C	U5b2c2	U	U5	32
990	JQ702792.1	JQ702792.1; U5b3a2; Asia_W_Europe_C; 31; A73G; C150T; A263G	16572	Asia_W_Europe_C	U5b3a2	U	U5	31
991	JQ702793.1	JQ702793.1; W1; Asia_W; 33; A73G; A189G; T195C	16570	Asia_W	W1	W	W	33
992	JQ702794.1	JQ702794.1; V1a1; Europe_S; 18; T72C; A263G; C309CCT	16570	Europe_S	V1a1	V	V	18
993	JQ702795.1	JQ702795.1; T2c1c2; Asia_W; 38; A73G; A263G; C315CC	16569	Asia_W	T2c1c2	T	T	38
994	JQ702796.1	JQ702796.1; K1a13; Asia_W; 35; A73G; T146C; T195C	16570	Asia_W	K1a13	K	K	35
995	JQ702797.1	JQ702797.1; K1a4a1a2b; Asia_W; 39; A73G; T146C; A263G	16571	Asia_W	K1a4a1a2b	K	K	39
996	JQ702798.1	JQ702798.1; K1a1b1b; Asia_W; 37; A73G; C114T; A189G	16569	Asia_W	K1a1b1b	K	K	37
997	JQ702799.1	JQ702799.1; H1ba; Asia_W_Europe; 12; A93G; A263G; C309CCCT	16571	Asia_W_Europe	H1ba	H	H	12
998	JQ702800.1	JQ702800.1; T2b4+152; Asia_W; 38; A73G; T152C; A263G	16568	Asia_W	T2b4+152	T	T	38
999	JQ702801.1	JQ702801.1; V; Europe_S; 15; T72C; A263G; C309CCT	16570	Europe_S	V	V	V	15
1000	JQ702802.1	JQ702802.1; K1a1a; Asia_W; 35; A73G; A263G; C315CC	16569	Asia_W	K1a1a	K	K	35
1001	JQ702803.1	JQ702803.1; V7; Europe_S; 19; T72C; A93G; A263G	16568	Europe_S	V7	V	V	19
1002	JQ702804.1	JQ702804.1; W3a1c; Asia_W; 40; A73G; A189G; C194T	16570	Asia_W	W3a1c	W	W	40
1003	JQ702805.1	JQ702805.1; K2b1a1a; Asia_W; 39; A73G; T146C; A263G	16569	Asia_W	K2b1a1a	K	K	39
1004	JQ702806.1	JQ702806.1; U5a1a2a; Asia_W_Europe_C; 30; A73G; A263G; C309CCT	16572	Asia_W_Europe_C	U5a1a2a	U	U5	30
1005	JQ702807.1	JQ702807.1; U5b1d1; Asia_W_Europe_C; 29; A73G; C150T; A263G	16572	Asia_W_Europe_C	U5b1d1	U	U5	29
1006	JQ702808.1	JQ702808.1; H3; Asia_W_Europe; 11; A263G; C309CCT; T310C	16570	Asia_W_Europe	H3	H	H	11
1007	JQ702809.1	JQ702809.1; U5a1a1d1; Asia_W_Europe_C; 31; A73G; G185A; A189G	16570	Asia_W_Europe_C	U5a1a1d1	U	U5	31
1008	JQ702810.1	JQ702810.1; K1b2b; Asia_W; 39; A73G; T146C; T195C	16570	Asia_W	K1b2b	K	K	39
1009	JQ702811.1	JQ702811.1; K1a4a1a1; Asia_W; 38; A73G; T195C; A263G	16571	Asia_W	K1a4a1a1	K	K	38
1010	JQ702812.1	JQ702812.1; U8a1a1b1; Asia_W_Europe ; 33; A73G; A263G; T282C	16570	Asia_W_Europe	U8a1a1b1	U	U8	33
1011	JQ702813.1	JQ702813.1; H6a1b3a; Asia_W_Europe; 21; T204C; T239C; A263G	16573	Asia_W_Europe	H6a1b3a	H	H	21
1012	JQ702814.1	JQ702814.1; H3m; Asia_W_Europe; 12; A73G; A263G; C315CC	16569	Asia_W_Europe	H3m	H	H	12
1013	JQ702815.1	JQ702815.1; U5b2a3; Asia_W_Europe_C; 32; A73G; C150T; A263G	16572	Asia_W_Europe_C	U5b2a3	U	U5	32
1014	JQ702816.1	JQ702816.1; U6a1b2; Asia_SW_Africa_N; 28; A73G; T195C; A263G	16570	Asia_SW_Africa_N	U6a1b2	U	U6	28
1015	JQ702817.1	JQ702817.1; X2c1a; Asia_W_America_N; 30; A73G; A153G; T195C	16569	Asia_W_America_N	X2c1a	X	X	30
1016	JQ702818.1	JQ702818.1; H10h; Asia_W_Europe; 12; A263G; C315CC; A750G	16569	Asia_W_Europe	H10h	H	H	12
1017	JQ702819.1	JQ702819.1; N1a1a1a3; Africa_NE_Asia_W; 40; A73G; T152C; T199C	16571	Africa_NE_Asia_W	N1a1a1a3	N	N1	40
1018	JQ702820.1	JQ702820.1; I1a1e; Asia_W_SW; 47; A73G; T146C; T199C	16570	Asia_W_SW	I1a1e	I	I	47
1019	JQ702821.1	JQ702821.1; H48; Asia_W_Europe; 11; A263G; C309CCT; T310C	16570	Asia_W_Europe	H48	H	H	11
1020	JQ702822.1	JQ702822.1; H6a1a; Asia_W_Europe; 18; T239C; A263G; C309CCT	16570	Asia_W_Europe	H6a1a	H	H	18
1021	JQ702823.1	JQ702823.1; H3a1a; Asia_W_Europe; 15; T152C; A263G; C309CCCT	16571	Asia_W_Europe	H3a1a	H	H	15
1022	JQ702824.1	JQ702824.1; T2b; Asia_W; 35; A73G; A263G; C309CCT	16570	Asia_W	T2b	T	T	35
1023	JQ702825.1	JQ702825.1; T1a1n; Asia_W; 40; A73G; T152C; T195C	16570	Asia_W	T1a1n	T	T	40
1024	JQ702826.1	JQ702826.1; J1c2; Asia_W; 33; A73G; G185A; A188G	16567	Asia_W	J1c2	J	J	33
1025	JQ702827.1	JQ702827.1; J1c14; Asia_W; 35; A73G; T195C; G228A	16570	Asia_W	J1c14	J	J	35

Supplementary Table S4 Human mitochondrial database (hMITO DB v1.0) metadata[a]

Row	Name (accession no.)	Description	Size	Geo_Region	Haplogroup	Macro_ Haplo	Macro_ Haplo2	Total Variants
1026	JQ702828.1	JQ702828.1; H1q; Asia_W_Europe; 14; A263G; C315CC; A750G	16569	Asia_W_Europe	H1q	H	H	14
1027	JQ702829.1	JQ702829.1; H13a1a1d1; Asia_W_Europe; 20; A263G; C309CCCT; T310C	16562	Asia_W_Europe	H13a1a1d1	H	H	20
1028	JQ702830.1	JQ702830.1; H6a1b3a; Asia_W_Europe; 20; T204C; T239C; A263G	16570	Asia_W_Europe	H6a1b3a	H	H	20
1029	JQ702831.1	JQ702831.1; H59; Asia_W_Europe; 12; A73G; A263G; C309CCT	16571	Asia_W_Europe	H59	H	H	12
1030	JQ702832.1	JQ702832.1; H1c1; Asia_W_Europe; 14; A234G; A263G; C309CCT	16570	Asia_W_Europe	H1c1	H	H	14
1031	JQ702833.1	JQ702833.1; H1e1b; Asia_W_Europe; 13; A263G; C315CC; T453C	16569	Asia_W_Europe	H1e1b	H	H	13
1032	JQ702834.1	JQ702834.1; H39a; Asia_W_Europe; 12; A263G; C309CCCT; T310C	16571	Asia_W_Europe	H39a	H	H	12
1033	JQ702835.1	JQ702835.1; H13a1a1d1; Asia_W_Europe; 20; T152C; A263G; C309CCCT	16571	Asia_W_Europe	H13a1a1d1	H	H	20
1034	JQ702836.1	JQ702836.1; U5a1c1a; Asia_W_Europe_C; 29; A73G; A153G; T195C	16570	Asia_W_Europe_C	U5a1c1a	U	U5	29
1035	JQ702837.1	JQ702837.1; U5b1b1a; Asia_W_Europe_C; 30; A73G; C150T; A263G	16569	Asia_W_Europe_C	U5b1b1a	U	U5	30
1036	JQ702838.1	JQ702838.1; H24a; Asia_W_Europe; 12; T146C; A263G; C315CC	16569	Asia_W_Europe	H24a	H	H	12
1037	JQ702839.1	JQ702839.1; V2a1a; Europe_S; 20; T72C; A263G; C309CCCT	16571	Europe_S	V2a1a	V	V	20
1038	JQ702840.1	JQ702840.1; H14b3; Asia_W_Europe; 15; A189G; G225A; A263G	16569	Asia_W_Europe	H14b3	H	H	15
1039	JQ702841.1	JQ702841.1; H49a; Asia_W_Europe; 13; A263G; C309CCCT; T310C	16571	Asia_W_Europe	H49a	H	H	13
1040	JQ702842.1	JQ702842.1; V1a; Europe_S; 19; T72C; A263G; C309CCT	16570	Europe_S	V1a	V	V	19
1041	JQ702843.1	JQ702843.1; U5b2b3a; Asia_W_Europe_C; 37; A73G; C150T; A263G	16570	Asia_W_Europe_C	U5b2b3a	U	U5	37
1042	JQ702844.1	JQ702844.1; Z1a1a; Asia_C_E; 45; A73G; C151T; T152C	16569	Asia_C_E	Z1a1a	Z	Z	45
1043	JQ702845.1	JQ702845.1; U5b1b1a1a1; Asia_W_Europe_C; 31; A73G; C150T; A263G	16569	Asia_W_Europe_C	U5b1b1a1a1	U	U5	31
1044	JQ702846.1	JQ702846.1; H1+152; Asia_W_Europe; 11; T152C; A263G; C315CC	16569	Asia_W_Europe	H1+152	H	H	11
1045	JQ702847.1	JQ702847.1; U5a2b3a; Asia_W_Europe_C; 31; A73G; C150T; A263G	16569	Asia_W_Europe_C	U5a2b3a	U	U5	31
1046	JQ702848.1	JQ702848.1; H1b1a; Asia_W_Europe; 16; A263G; C315CC; CA522d	16568	Asia_W_Europe	H1b1a	H	H	16
1047	JQ702849.1	JQ702849.1; K1b2a2; Asia_W; 36; A73G; T146C; T195C	16573	Asia_W	K1b2a2	K	K	36
1048	JQ702850.1	JQ702850.1; H1c7; Asia_W_Europe; 11; A263G; C315CC; T477C	16569	Asia_W_Europe	H1c7	H	H	11
1049	JQ702851.1	JQ702851.1; K1c1b; Asia_W; 35; A73G; T146C; T152C	16568	Asia_W	K1c1b	K	K	35
1050	JQ702852.1	JQ702852.1; K1+16362; Asia_W; 40; A73G; C194T; T246C	16569	Asia_W	K1+16362	K	K	40
1051	JQ702853.1	JQ702853.1; T1a1; Asia_W; 38; A73G; C151T; T152C	16571	Asia_W	T1a1	T	T	38
1052	JQ702854.1	JQ702854.1; K1a+195; Asia_W; 31; A73G; T195C; A263G	16569	Asia_W	K1a+195	K	K	31
1053	JQ702855.1	JQ702855.1; B2a1; Asia_SE_E_America_N_S; 34; A73G; A263G; C309CCT	16561	Asia_SE_E_America_N_S	B2a1	B	B	34
1054	JQ702856.1	JQ702856.1; H1n5; Asia_W_Europe; 13; T146C; T195C; A263G	16569	Asia_W_Europe	H1n5	H	H	13
1055	JQ702857.1	JQ702857.1; J1c7a; Asia_W; 32; A73G; G185A; A263G	16570	Asia_W	J1c7a	J	J	32
1056	JQ702858.1	JQ702858.1; J2b1a3; Asia_W; 33; A73G; C150T; T152C	16570	Asia_W	J2b1a3	J	J	33
1057	JQ702859.1	JQ702859.1; K1a1b1a; Asia_W; 36; A73G; C114T; A263G	16569	Asia_W	K1a1b1a	K	K	36
1058	JQ702860.1	JQ702860.1; H33a; Asia_W_Europe; 10; A263G; C315CC; A750G	16569	Asia_W_Europe	H33a	H	H	10
1059	JQ702861.1	JQ702861.1; K1c2; Asia_W; 36; A73G; T146C; T152C	16568	Asia_W	K1c2	K	K	36
1060	JQ702862.1	JQ702862.1; U4a2e; Asia_N_Europe_N; 32; A73G; T195C; A263G	16572	Asia_N_Europe_N	U4a2e	U	U4	32
1061	JQ702863.1	JQ702863.1; J2b1a1a; Asia_W; 33; A73G; C150T; T152C	16569	Asia_W	J2b1a1a	J	J	33
1062	JQ702864.1	JQ702864.1; U4b1b1b; Asia_N_Europe_N; 39; A73G; T152C; T195C	16569	Asia_N_Europe_N	U4b1b1b	U	U4	39
1063	JQ702865.1	JQ702865.1; H8c; Asia_W_Europe; 21; C114T; T146C; T152C	16569	Asia_W_Europe	H8c	H	H	21
1064	JQ702866.1	JQ702866.1; J1b1a1b; Asia_W; 39; A73G; A153G; C242T	16570	Asia_W	J1b1a1b	J	J	39
1065	JQ702867.1	JQ702867.1; H1c8; Asia_W_Europe; 15; T152C; A263G; C309CCT	16570	Asia_W_Europe	H1c8	H	H	15
1066	JQ702868.1	JQ702868.1; J1c8a; Asia_W; 30; A73G; G185A; G228A	16569	Asia_W	J1c8a	J	J	30

Supplementary Table S4 Human mitochondrial database (hMITO DB v1.0) metadata[a]

Row	Name (accession no.)	Description	Size	Geo_Region	Haplogroup	Macro_ Haplo	Macro_ Haplo2	Total Variants
1067	JQ702869.1	JQ702869.1; K1a1a2a1; Asia_W; 37; A73G; A263G; C309CCT	16571	Asia_W	K1a1a2a1	K	K	37
1068	JQ702870.1	JQ702870.1; H1j; Asia_W_Europe; 13; A263G; C309CCT; T310C	16570	Asia_W_Europe	H1j	H	H	13
1069	JQ702871.1	JQ702871.1; U5a1a2a; Asia_W_Europe_C; 31; A73G; A263G; C309CCT	16572	Asia_W_Europe_C	U5a1a2a	U	U5	31
1070	JQ702872.1	JQ702872.1; K1a10a; Asia_W; 34; A73G; T195C; A263G	16571	Asia_W	K1a10a	K	K	34
1071	JQ702873.1	JQ702873.1; H1c7; Asia_W_Europe; 12; A263G; C315CC; T477C	16569	Asia_W_Europe	H1c7	H	H	12
1072	JQ702874.1	JQ702874.1; H4a1a1a; Asia_W_Europe; 18; A73G; A263G; C315CC	16567	Asia_W_Europe	H4a1a1a	H	H	18
1073	JQ702875.1	JQ702875.1; H51a; Asia_W_Europe; 13; A263G; C309CCT; T310C	16570	Asia_W_Europe	H51a	H	H	13
1074	JQ702876.1	JQ702876.1; J1c8a; Asia_W; 29; A73G; G185A; G228A	16570	Asia_W	J1c8a	J	J	29
1075	JQ702877.1	JQ702877.1; H10e; Asia_W_Europe; 13; A263G; C315CC; G709A	16569	Asia_W_Europe	H10e	H	H	13
1076	JQ702878.1	JQ702878.1; U5a1h; Asia_W_Europe_C; 36; A73G; C150T; A263G	16570	Asia_W_Europe_C	U5a1h	U	U5	36
1077	JQ702879.1	JQ702879.1; H10e; Asia_W_Europe; 12; A263G; C315CC; A750G	16569	Asia_W_Europe	H10e	H	H	12
1078	JQ702880.1	JQ702880.1; T2a1a5; Asia_W; 36; A73G; A263G; C315CC	16569	Asia_W	T2a1a5	T	T	36
1079	JQ702881.1	JQ702881.1; H1b1+16362; Asia_W_Europe; 20; A263G; C309CCCT; T310C	16569	Asia_W_Europe	H1b1+16362	H	H	20
1080	JQ702882.1	JQ702882.1; I1a1; Asia_W_SW; 42; A73G; C150T; T199C	16572	Asia_W_SW	I1a1	I	I	42
1081	JQ702883.1	JQ702883.1; K1d1; Asia_W; 38; A73G; T195C; A200G	16572	Asia_W	K1d1	K	K	38
1082	JQ702884.1	JQ702884.1; T2b5; Asia_W; 34; A73G; A263G; C315CC	16569	Asia_W	T2b5	T	T	34
1083	JQ702885.1	JQ702885.1; T2b5a1; Asia_W; 39; A73G; T152C; A263G	16571	Asia_W	T2b5a1	T	T	39
1084	JQ702886.1	JQ702886.1; H4a1a3a; Asia_W_Europe; 18; T195C; A263G; C315CC	16567	Asia_W_Europe	H4a1a3a	H	H	18
1085	JQ702887.1	JQ702887.1; K2a; Asia_W; 37; A73G; T152C; T199C	16571	Asia_W	K2a	K	K	37
1086	JQ702888.1	JQ702888.1; X2b; Asia_W_America_N; 32; A73G; A153G; T195C	16569	Asia_W_America_N	X2b	X	X	32
1087	JQ702889.1	JQ702889.1; H49a2; Asia_W_Europe; 14; A93G; A263G; C309CCT	16570	Asia_W_Europe	H49a2	H	H	14
1088	JQ702890.1	JQ702890.1; H2a1e; Asia_W_Europe; 9; A263G; C315CC; C575T	16569	Asia_W_Europe	H2a1e	H	H	9
1089	JQ702891.1	JQ702891.1; T2e5; Asia_W; 35; A73G; C150T; A200G	16571	Asia_W	T2e5	T	T	35
1090	JQ702892.1	JQ702892.1; H5a1; Asia_W_Europe; 14; A263G; C315CC; C456T	16567	Asia_W_Europe	H5a1	H	H	14
1091	JQ702893.1	JQ702893.1; X2b6a; Asia_W_America_N; 37; A73G; A153G; T195C	16569	Asia_W_America_N	X2b6a	X	X	37
1092	JQ702894.1	JQ702894.1; T2b36; Asia_W; 36; A73G; A263G; C315CC	16569	Asia_W	T2b36	T	T	36
1093	JQ702895.1	JQ702895.1; H5m; Asia_W_Europe; 16; T146C; A263G; C309CCT	16570	Asia_W_Europe	H5m	H	H	16
1094	JQ702896.1	JQ702896.1; K1a4a1a2b; Asia_W; 39; A73G; T146C; A263G	16570	Asia_W	K1a4a1a2b	K	K	39
1095	JQ702897.1	JQ702897.1; H; Asia_W_Europe; 12; A93G; C151T; A263G	16569	Asia_W_Europe	H	H	H	12
1096	JQ702898.1	JQ702898.1; K1a1b1b; Asia_W; 37; A73G; C114T; T195C	16570	Asia_W	K1a1b1b	K	K	37
1097	JQ702899.1	JQ702899.1; H1c3; Asia_W_Europe; 16; T195C; A257G; A263G	16570	Asia_W_Europe	H1c3	H	H	16
1098	JQ702900.1	JQ702900.1; H2a2b4; Asia_W_Europe; 10; A263G; C309CCT; T310C	16570	Asia_W_Europe	H2a2b4	H	H	10
1099	JQ702901.1	JQ702901.1; K1c1e; Asia_W; 35; A73G; T146C; T195C	16568	Asia_W	K1c1e	K	K	35
1100	JQ702902.1	JQ702902.1; K1a8; Asia_W; 37; A73G; A263G; C295A	16575	Asia_W	K1a8	K	K	37
1101	JQ702903.1	JQ702903.1; L1c2b1c; Africa_C; 93; A73G; T152C; C182T	16568	Africa_C	L1c2b1c	L1	L1	93
1102	JQ702904.1	JQ702904.1; L2a1l2a1; Africa_W_C; 55; A73G; T146C; T152C	16570	Africa_W_C	L2a1l2a1	L2	L2	55
1103	JQ702905.1	JQ702905.1; K1c1e; Asia_W; 36; A73G; T146C; T152C	16568	Asia_W	K1c1e	K	K	36
1104	JQ702906.1	JQ702906.1; H55+153; Asia_W_Europe; 12; A153G; A263G; C309CCCT	16571	Asia_W_Europe	H55+153	H	H	12
1105	JQ702907.1	JQ702907.1; H1aq1; Asia_W_Europe; 14; A263G; C315CC; CA522d	16567	Asia_W_Europe	H1aq1	H	H	14
1106	JQ702908.1	JQ702908.1; K1c1b; Asia_W; 36; A73G; T146C; T152C	16568	Asia_W	K1c1b	K	K	36
1107	JQ702909.1	JQ702909.1; H3z; Asia_W_Europe; 12; A263G; T293C; C309CCCT	16571	Asia_W_Europe	H3z	H	H	12

Supplementary Table S4 Human mitochondrial database (hMITO DB v1.0) metadata[a]

Row	Name (accession no.)	Description	Size	Geo_Region	Haplogroup	Macro_ Haplo	Macro_ Haplo2	Total Variants
1108	JQ702910.1	JQ702910.1; H4a1a4b; Asia_W_Europe; 20; T195C; A263G; C315CC	16567	Asia_W_Europe	H4a1a4b	H	H	20
1109	JQ702911.1	JQ702911.1; K1a4h1; Asia_W; 38; A73G; G207A; A263G	16572	Asia_W	K1a4h1	K	K	38
1110	JQ702912.1	JQ702912.1; J1c3k; Asia_W; 31; A73G; G185A; A263G	16570	Asia_W	J1c3k	J	J	31
1111	JQ702913.1	JQ702913.1; U5a1g1; Asia_W_Europe_C; 28; A73G; A263G; C315CC	16569	Asia_W_Europe_C	U5a1g1	U	U5	28
1112	JQ702914.1	JQ702914.1; C1b; Asia_NE_America_N_S; 41; A73G; A249d; A263G	16563	Asia_NE_America_N_S	C1b	C	C	41
1113	JQ702915.1	JQ702915.1; H7a1b; Asia_W_Europe; 16; T89C; A93G; A263G	16571	Asia_W_Europe	H7a1b	H	H	16
1114	JQ702916.1	JQ702916.1; H28a; Asia_W_Europe; 13; C186A; A263G; C309CCT	16570	Asia_W_Europe	H28a	H	H	13
1115	JQ702917.1	JQ702917.1; U5a2b; Asia_W_Europe_C; 28; A73G; T152C; A263G	16572	Asia_W_Europe_C	U5a2b	U	U5	28
1116	JQ702918.1	JQ702918.1; H64; Asia_W_Europe; 13; A263G; C315CC; A750G	16569	Asia_W_Europe	H64	H	H	13
1117	JQ702919.1	JQ702919.1; K1b1a1a; Asia_W; 42; A73G; T152C; T199C	16572	Asia_W	K1b1a1a	K	K	42
1118	JQ702920.1	JQ702920.1; K2a4; Asia_W; 33; A73G; T146C; T152C	16569	Asia_W	K2a4	K	K	33
1119	JQ702921.1	JQ702921.1; H3b2; Asia_W_Europe; 14; A263G; C309CCT; T310C	16570	Asia_W_Europe	H3b2	H	H	14
1120	JQ702922.1	JQ702922.1; U5a1a1c; Asia_W_Europe_C; 29; A73G; A263G; C315CC	16569	Asia_W_Europe_C	U5a1a1c	U	U5	29
1121	JQ702923.1	JQ702923.1; T1a1c; Asia_W; 39; A73G; T152C; T195C	16570	Asia_W	T1a1c	T	T	39
1122	JQ702924.1	JQ702924.1; H40b; Asia_W_Europe; 13; T152C; A263G; C309CCT	16570	Asia_W_Europe	H40b	H	H	13
1123	JQ702925.1	JQ702925.1; T1a1j; Asia_W; 37; A73G; T152C; T195C	16570	Asia_W	T1a1j	T	T	37
1124	JQ702926.1	JQ702926.1; K1b2a1a1; Asia_W; 39; A73G; T146C; T195C	16573	Asia_W	K1b2a1a1	K	K	39
1125	JQ702927.1	JQ702927.1; U5b2b1b; Asia_W_Europe_C; 31; A73G; C150T; A263G	16569	Asia_W_Europe_C	U5b2b1b	U	U5	31
1126	JQ702928.1	JQ702928.1; T2c1d+152; Asia_W; 39; A73G; T146C; T152C	16570	Asia_W	T2c1d+152	T	T	39
1127	JQ702929.1	JQ702929.1; J1c7a; Asia_W; 34; A73G; G228A; A263G	16569	Asia_W	J1c7a	J	J	34
1128	JQ702930.1	JQ702930.1; U8a1a; Asia_W_Europe ; 28; A73G; A263G; T282C	16570	Asia_W_Europe	U8a1a	U	U8	28
1129	JQ702931.1	JQ702931.1; K1a16; Asia_W; 32; A73G; T195C; A263G	16573	Asia_W	K1a16	K	K	32
1130	JQ702932.1	JQ702932.1; T2b3c; Asia_W; 40; A73G; C151T; A263G	16569	Asia_W	T2b3c	T	T	40
1131	JQ702933.1	JQ702933.1; H40b; Asia_W_Europe; 12; A263G; C309CCT; T310C	16570	Asia_W_Europe	H40b	H	H	12
1132	JQ702934.1	JQ702934.1; U2e2a1a; Asia_S_W_Europe; 42; A73G; T152C; T217C	16571	Asia_S_W_Europe	U2e2a1a	U	U2	42
1133	JQ702935.1	JQ702935.1; V10a; Europe_S; 20; T72C; A263G; C309CCT	16570	Europe_S	V10a	V	V	20
1134	JQ702936.1	JQ702936.1; H4a2; Asia_W_Europe; 17; A263G; C309CCT; T310C	16568	Asia_W_Europe	H4a2	H	H	17
1135	JQ702937.1	JQ702937.1; T2a1b1a1; Asia_W; 41; A73G; A263G; C309CCT	16570	Asia_W	T2a1b1a1	T	T	41
1136	JQ702938.1	JQ702938.1; V1b; Europe_S; 18; T72C; A263G; C309CCCT	16571	Europe_S	V1b	V	V	18
1137	JQ702939.1	JQ702939.1; I1a1a; Asia_W_SW; 44; A73G; T199C; G203A	16574	Asia_W_SW	I1a1a	I	I	44
1138	JQ702940.1	JQ702940.1; R0a4; Africa_NE_Asia_W; 20; T57TC; C64T; C150T	16570	Africa_NE_Asia_W	R0a4	R	R0	20
1139	JQ702941.1	JQ702941.1; U2e3a; Asia_S_W_Europe; 45; A73G; T152C; T217C	16572	Asia_S_W_Europe	U2e3a	U	U2	45
1140	JQ702942.1	JQ702942.1; U4a2a; Asia_N_Europe_N; 29; A73G; T195C; A263G	16568	Asia_N_Europe_N	U4a2a	U	U4	29
1141	JQ702943.1	JQ702943.1; H2b; Asia_W_Europe; 12; T152C; A263G; C309CCT	16570	Asia_W_Europe	H2b	H	H	12
1142	JQ702944.1	JQ702944.1; K1c2; Asia_W; 36; A73G; T146C; T152C	16568	Asia_W	K1c2	K	K	36
1143	JQ702945.1	JQ702945.1; K1a1b1a; Asia_W; 37; A73G; C114T; A263G	16569	Asia_W	K1a1b1a	K	K	37
1144	JQ702946.1	JQ702946.1; H5a1; Asia_W_Europe; 15; G66T; A263G; C315CC	16567	Asia_W_Europe	H5a1	H	H	15
1145	JQ702947.1	JQ702947.1; K1a15; Asia_W; 33; A73G; T195C; A263G	16573	Asia_W	K1a15	K	K	33
1146	JQ702948.1	JQ702948.1; H7; Asia_W_Europe; 11; A263G; C315CC; A750G	16569	Asia_W_Europe	H7	H	H	11
1147	JQ702949.1	JQ702949.1; H3; Asia_W_Europe; 11; T146C; A263G; C309CCT	16570	Asia_W_Europe	H3	H	H	11
1148	JQ702950.1	JQ702950.1; J2a1a1; Asia_W; 40; A73G; C150T; T152C	16570	Asia_W	J2a1a1	J	J	40

Supplementary Table S4 Human mitochondrial database (hMITO DB v1.0) metadata[a]

Row	Name (accession no.)	Description	Size	Geo_Region	Haplogroup	Macro_ Haplo	Macro_ Haplo2	Total Variants
1149	JQ702951.1	JQ702951.1; T2b3e; Asia_W; 39; A73G; C151T; A263G	16570	Asia_W	T2b3e	T	T	39
1150	JQ702952.1	JQ702952.1; T1a4; Asia_W; 41; A73G; T152C; A263G	16570	Asia_W	T1a4	T	T	41
1151	JQ702953.1	JQ702953.1; T2b26; Asia_W; 37; A73G; T152C; T195C	16569	Asia_W	T2b26	T	T	37
1152	JQ702954.1	JQ702954.1; T2e; Asia_W; 35; A73G; C150T; A263G	16571	Asia_W	T2e	T	T	35
1153	JQ702955.1	JQ702955.1; M1a; Africa_E_Nile Valley; 42; A73G; G143A; T146C	16569	Africa_E_Nile Valley	M1a	M	M1	42
1154	JQ702956.1	JQ702956.1; H4a1; Asia_W_Europe; 17; A263G; C315CC; CA522d	16567	Asia_W_Europe	H4a1	H	H	17
1155	JQ702957.1	JQ702957.1; T2f5; Asia_W; 36; A73G; A263G; C315CC	16560	Asia_W	T2f5	T	T	36
1156	JQ702958.1	JQ702958.1; T1a1b; Asia_W; 39; A73G; T152C; T195C	16570	Asia_W	T1a1b	T	T	39
1157	JQ702959.1	JQ702959.1; T1a1k1; Asia_W; 38; A73G; T146C; T152C	16571	Asia_W	T1a1k1	T	T	38
1158	JQ702960.1	JQ702960.1; H4c1; Asia_W_Europe; 15; A73G; A263G; C315CC	16567	Asia_W_Europe	H4c1	H	H	15
1159	JQ702961.1	JQ702961.1; H27d; Asia_W_Europe; 12; A263G; C315CC; G380A	16569	Asia_W_Europe	H27d	H	H	12
1160	JQ702962.1	JQ702962.1; T1a1; Asia_W; 36; A73G; T152C; T195C	16570	Asia_W	T1a1	T	T	36
1161	JQ702963.1	JQ702963.1; T2b13; Asia_W; 38; A73G; T195C; A263G	16570	Asia_W	T2b13	T	T	38
1162	JQ702964.1	JQ702964.1; U5b2a1a1; Asia_W_Europe_C; 29; A73G; C150T; A263G	16569	Asia_W_Europe_C	U5b2a1a1	U	U5	29
1163	JQ702965.1	JQ702965.1; T2b+150; Asia_W; 35; A73G; C150T; A263G	16569	Asia_W	T2b+150	T	T	35
1164	JQ702966.1	JQ702966.1; H11a; Asia_W_Europe; 14; T195C; A263G; C315CC	16569	Asia_W_Europe	H11a	H	H	14
1165	JQ702967.1	JQ702967.1; J1c11; Asia_W; 28; A73G; G185A; G228A	16569	Asia_W	J1c11	J	J	28
1166	JQ702968.1	JQ702968.1; L2a1c+16129; Africa_W_C; 58; A73G; G143A; T146C	16568	Africa_W_C	L2a1c+16129	L2	L2	58
1167	JQ702969.1	JQ702969.1; T2a1a; Asia_W; 34; A73G; A263G; C309CCT	16570	Asia_W	T2a1a	T	T	34
1168	JQ702970.1	JQ702970.1; U5a1h; Asia_W_Europe_C; 34; A73G; C150T; A263G	16569	Asia_W_Europe_C	U5a1h	U	U5	34
1169	JQ702971.1	JQ702971.1; H1u1; Asia_W_Europe; 13; A263G; C315CC; A750G	16569	Asia_W_Europe	H1u1	H	H	13
1170	JQ702972.1	JQ702972.1; T2a1b1a; Asia_W; 38; A73G; T146C; A263G	16569	Asia_W	T2a1b1a	T	T	38
1171	JQ702973.1	JQ702973.1; N1b1a8a; Africa_NE_Asia_W; 38; A73G; T152C; T195C	16569	Africa_NE_Asia_W	N1b1a8a	N	N1	38
1172	JQ702974.1	JQ702974.1; H2a2a2; Asia_W_Europe; 8; T152C; A200G; A263G	16570	Asia_W_Europe	H2a2a2	H	H	8
1173	JQ702975.1	JQ702975.1; T2f1a1; Asia_W; 43; A73G; T195C; A263G	16561	Asia_W	T2f1a1	T	T	43
1174	JQ702976.1	JQ702976.1; J1b1a1a; Asia_W; 40; A73G; C242T; A263G	16569	Asia_W	J1b1a1a	J	J	40
1175	JQ702977.1	JQ702977.1; K1c2; Asia_W; 38; A73G; T146C; T152C	16570	Asia_W	K1c2	K	K	38
1176	JQ702978.1	JQ702978.1; T2b5; Asia_W; 35; A73G; A263G; C309CCT	16570	Asia_W	T2b5	T	T	35
1177	JQ702979.1	JQ702979.1; T2b; Asia_W; 36; A73G; A263G; C315CC	16567	Asia_W	T2b	T	T	36
1178	JQ702980.1	JQ702980.1; T2b; Asia_W; 33; A73G; A263G; C315CC	16571	Asia_W	T2b	T	T	33
1179	JQ702981.1	JQ702981.1; J1c1b2a; Asia_W; 34; A73G; G228A; A263G	16567	Asia_W	J1c1b2a	J	J	34
1180	JQ702982.1	JQ702982.1; T2b5a1; Asia_W; 37; A73G; A263G; C309CCT	16572	Asia_W	T2b5a1	T	T	37
1181	JQ702983.1	JQ702983.1; H1aq1; Asia_W_Europe; 13; A263G; C315CC; CA522d	16567	Asia_W_Europe	H1aq1	H	H	13
1182	JQ702984.1	JQ702984.1; C1b9; Asia_NE_America_N_S; 45; A73G; T146C; A249d	16565	Asia_NE_America_N_S	C1b9	C	C	45
1183	JQ702985.1	JQ702985.1; H49a1; Asia_W_Europe; 12; A263G; C309CCT; T310C	16570	Asia_W_Europe	H49a1	H	H	12
1184	JQ702986.1	JQ702986.1; T1a1; Asia_W; 34; A73G; T152C; T195C	16569	Asia_W	T1a1	T	T	34
1185	JQ702987.1	JQ702987.1; M30b; Asia_S; 40; C44CC; A73G; T152C	16570	Asia_S	M30b	M	M30	40
1186	JQ702988.1	JQ702988.1; T1a1+@152; Asia_W; 34; A73G; T195C; A263G	16569	Asia_W	T1a1+@152	T	T	34
1187	JQ702989.1	JQ702989.1; W+194; Asia_W; 36; A73G; A189G; C194T	16568	Asia_W	W+194	W	W	36
1188	JQ702990.1	JQ702990.1; H7b; Asia_W_Europe; 11; A263G; C315CC; A750G	16569	Asia_W_Europe	H7b	H	H	11
1189	JQ702991.1	JQ702991.1; K1a13a; Asia_W; 37; A73G; T146C; T195C	16571	Asia_W	K1a13a	K	K	37

Supplementary Table S4 Human mitochondrial database (hMITO DB v1.0) metadata[a]

Row	Name (accession no.)	Description	Size	Geo_Region	Haplogroup	Macro_ Haplo	Macro_ Haplo2	Total Variants
1190	JQ702992.1	JQ702992.1; H1e2b; Asia_W_Europe; 13; A263G; C309CCT; T310C	16570	Asia_W_Europe	H1e2b	H	H	13
1191	JQ702993.1	JQ702993.1; X2c1a; Asia_W_America_N; 32; A73G; A153G; T195C	16569	Asia_W_America_N	X2c1a	X	X	32
1192	JQ702994.1	JQ702994.1; H16a1; Asia_W_Europe; 14; T152C; A263G; C315CC	16569	Asia_W_Europe	H16a1	H	H	14
1193	JQ702995.1	JQ702995.1; X2b+226+16192; Asia_W_America_N; 34; A73G; T152C; A153G	16570	Asia_W_America_N	X2b+226+1619:	X	X	34
1194	JQ702996.1	JQ702996.1; J1c5a1; Asia_W; 33; A73G; G185A; G228A	16569	Asia_W	J1c5a1	J	J	33
1195	JQ702997.1	JQ702997.1; H26a1; Asia_W_Europe; 15; T152C; C194T; A263G	16570	Asia_W_Europe	H26a1	H	H	15
1196	JQ702998.1	JQ702998.1; H5a1; Asia_W_Europe; 14; A263G; C315CC; C456T	16567	Asia_W_Europe	H5a1	H	H	14
1197	JQ702999.1	JQ702999.1; H3+152; Asia_W_Europe; 11; T152C; A263G; C315CC	16571	Asia_W_Europe	H3+152	H	H	11
1198	JQ703000.1	JQ703000.1; H4a1a4b1; Asia_W_Europe; 23; T195C; A263G; C315CC	16567	Asia_W_Europe	H4a1a4b1	H	H	23
1199	JQ703001.1	JQ703001.1; K2b1a1; Asia_W; 37; A73G; T146C; T152C	16569	Asia_W	K2b1a1	K	K	37
1200	JQ703002.1	JQ703002.1; H10e1; Asia_W_Europe; 14; A263G; C309CCCT; T310C	16571	Asia_W_Europe	H10e1	H	H	14
1201	JQ703003.1	JQ703003.1; K1b1c; Asia_W; 37; A73G; G94A; A263G	16570	Asia_W	K1b1c	K	K	37
1202	JQ703004.1	JQ703004.1; H30a; Asia_W_Europe; 15; A263G; C309CCT; T310C	16570	Asia_W_Europe	H30a	H	H	15
1203	JQ703005.1	JQ703005.1; K1a10; Asia_W; 35; A73G; T195C; A263G	16573	Asia_W	K1a10	K	K	35
1204	JQ703006.1	JQ703006.1; K1a+195; Asia_W; 33; A73G; T152C; T195C	16573	Asia_W	K1a+195	K	K	33
1205	JQ703007.1	JQ703007.1; K1a4a1a1; Asia_W; 39; A73G; T195C; A263G	16571	Asia_W	K1a4a1a1	K	K	39
1206	JQ703008.1	JQ703008.1; K1b1a1; Asia_W; 42; A73G; T152C; A263G	16572	Asia_W	K1b1a1	K	K	42
1207	JQ703009.1	JQ703009.1; T2b19b; Asia_W; 38; A73G; A263G; C315CC	16567	Asia_W	T2b19b	T	T	38
1208	JQ703010.1	JQ703010.1; H10f; Asia_W_Europe; 12; A263G; C315CC; A750G	16569	Asia_W_Europe	H10f	H	H	12
1209	JQ703011.1	JQ703011.1; T2b9; Asia_W; 37; A73G; C150T; A263G	16569	Asia_W	T2b9	T	T	37
1210	JQ703012.1	JQ703012.1; K1a1b1a; Asia_W; 35; A73G; A263G; C315CC	16569	Asia_W	K1a1b1a	K	K	35
1211	JQ703013.1	JQ703013.1; W1+119; Asia_W; 36; A73G; T119C; A189G	16561	Asia_W	W1+119	W	W	36
1212	JQ703014.1	JQ703014.1; H4a1a1a; Asia_W_Europe; 20; A73G; A263G; C309CCT	16568	Asia_W_Europe	H4a1a1a	H	H	20
1213	JQ703015.1	JQ703015.1; C7a; Asia_NE_America_N_S; 42; C44CC; A73G; A249d	16569	Asia_NE_America_N_S	C7a	C	C	42
1214	JQ703016.1	JQ703016.1; H23; Asia_W_Europe; 10; T72C; A263G; C315CC	16569	Asia_W_Europe	H23	H	H	10
1215	JQ703017.1	JQ703017.1; H2a1a; Asia_W_Europe; 12; T152C; A263G; C309CCT	16572	Asia_W_Europe	H2a1a	H	H	12
1216	JQ703018.1	JQ703018.1; K2a5; Asia_W; 35; A73G; T146C; T152C	16569	Asia_W	K2a5	K	K	35
1217	JQ703019.1	JQ703019.1; K1c2; Asia_W; 38; A73G; T146C; T152C	16568	Asia_W	K1c2	K	K	38
1218	JQ703020.1	JQ703020.1; H2a2a1; Asia_W_Europe; 3; A183G; C315CC; A16207G	16569	Asia_W_Europe	H2a2a1	H	H	3
1219	JQ703021.1	JQ703021.1; K1b2a3; Asia_W; 35; A73G; T146C; T195C	16575	Asia_W	K1b2a3	K	K	35
1220	JQ703022.1	JQ703022.1; H3; Asia_W_Europe; 10; A263G; C315CC; A750G	16569	Asia_W_Europe	H3	H	H	10
1221	JQ703023.1	JQ703023.1; T2b5a1; Asia_W; 36; A73G; A263G; C315CC	16569	Asia_W	T2b5a1	T	T	36
1222	JQ703024.1	JQ703024.1; I2c; Asia_W_SW; 39; A73G; T152C; T199C	16571	Asia_W_SW	I2c	I	I	39
1223	JQ703025.1	JQ703025.1; K1a4d; Asia_W; 37; A73G; A153G; A263G	16572	Asia_W	K1a4d	K	K	37
1224	JQ703026.1	JQ703026.1; K1a2a; Asia_W; 36; A73G; A263G; C309CCT	16572	Asia_W	K1a2a	K	K	36
1225	JQ703027.1	JQ703027.1; U5b2a2c; Asia_W_Europe_C; 35; A73G; C150T; A263G	16569	Asia_W_Europe_C	U5b2a2c	U	U5	35
1226	JQ703028.1	JQ703028.1; H6a1a3; Asia_W_Europe; 17; T239C; A263G; C309CCCT	16571	Asia_W_Europe	H6a1a3	H	H	17
1227	JQ703029.1	JQ703029.1; J1c1b1; Asia_W; 36; C64T; A73G; G185A	16569	Asia_W	J1c1b1	J	J	36
1228	JQ703030.1	JQ703030.1; T2c1a1; Asia_W; 41; A73G; T152C; A263G	16570	Asia_W	T2c1a1	T	T	41
1229	JQ703031.1	JQ703031.1; T2b; Asia_W; 36; A73G; C140T; A263G	16570	Asia_W	T2b	T	T	36
1230	JQ703032.1	JQ703032.1; T1a1a1; Asia_W; 37; A73G; T152C; T195C	16570	Asia_W	T1a1a1	T	T	37

Supplementary Table S4 Human mitochondrial database (hMITO DB v1.0) metadata[a]

Row	Name (accession no.)	Description	Size	Geo_Region	Haplogroup	Macro_ Haplo	Macro_ Haplo2	Total Variants
1231	JQ703033.1	JQ703033.1; K1b2a; Asia_W; 35; A73G; T146C; T195C	16573	Asia_W	K1b2a	K	K	35
1232	JQ703034.1	JQ703034.1; K1a4d; Asia_W; 34; A73G; A263G; C315CC	16569	Asia_W	K1a4d	K	K	34
1233	JQ703035.1	JQ703035.1; H13a1a1; Asia_W_Europe; 15; A263G; C309CCT; T310C	16570	Asia_W_Europe	H13a1a1	H	H	15
1234	JQ703036.1	JQ703036.1; L1b1a3b; Africa_C; 82; A73G; T152C; C182T	16567	Africa_C	L1b1a3b	L1	L1	82
1235	JQ703037.1	JQ703037.1; K1a2c; Asia_W; 39; A73G; T152C; A263G	16573	Asia_W	K1a2c	K	K	39
1236	JQ703038.1	JQ703038.1; K2b1a; Asia_W; 36; A73G; T146C; A263G	16569	Asia_W	K2b1a	K	K	36
1237	JQ703039.1	JQ703039.1; K2a3; Asia_W; 33; A73G; T146C; T152C	16570	Asia_W	K2a3	K	K	33
1238	JQ703040.1	JQ703040.1; N1a3a; Africa_NE_Asia_W; 33; A73G; A189G; T195C	16569	Africa_NE_Asia_W	N1a3a	N	N1	33
1239	JQ703041.1	JQ703041.1; K1a2a; Asia_W; 36; A73G; A263G; C315CC	16571	Asia_W	K1a2a	K	K	36
1240	JQ703042.1	JQ703042.1; T2b13b; Asia_W; 36; A73G; A263G; C309CCT	16570	Asia_W	T2b13b	T	T	36
1241	JQ703043.1	JQ703043.1; H3g; Asia_W_Europe; 12; T152C; A263G; C315CC	16569	Asia_W_Europe	H3g	H	H	12
1242	JQ703044.1	JQ703044.1; K1c2; Asia_W; 35; A73G; T146C; T152C	16569	Asia_W	K1c2	K	K	35
1243	JQ703045.1	JQ703045.1; N9a3; Africa_NE_Asia_E; 28; A73G; T146C; C150T	16570	Africa_NE_Asia_E	N9a3	N	N9	28
1244	JQ703046.1	JQ703046.1; U8a1a2; Asia_W_Europe ; 31; A73G; C150T; A263G	16570	Asia_W_Europe	U8a1a2	U	U8	31
1245	JQ703047.1	JQ703047.1; J1c3; Asia_W; 29; A73G; G185A; G228A	16569	Asia_W	J1c3	J	J	29
1246	JQ703048.1	JQ703048.1; K1a4d; Asia_W; 36; A73G; A263G; C315CC	16571	Asia_W	K1a4d	K	K	36
1247	JQ703049.1	JQ703049.1; K2a6; Asia_W; 33; A73G; T146C; T152C	16569	Asia_W	K2a6	K	K	33
1248	JQ703050.1	JQ703050.1; K2a; Asia_W; 32; A73G; T146C; T152C	16570	Asia_W	K2a	K	K	32
1249	JQ703051.1	JQ703051.1; K1a+195; Asia_W; 32; A73G; T195C; A263G	16571	Asia_W	K1a+195	K	K	32
1250	JQ703052.1	JQ703052.1; H1a; Asia_W_Europe; 13; A73G; A263G; C309CCT	16570	Asia_W_Europe	H1a	H	H	13
1251	JQ703053.1	JQ703053.1; H13a1a1; Asia_W_Europe; 20; A263G; C309CCT; T310C	16568	Asia_W_Europe	H13a1a1	H	H	20
1252	JQ703054.1	JQ703054.1; K1c1b; Asia_W; 36; A73G; T146C; T152C	16568	Asia_W	K1c1b	K	K	36
1253	JQ703055.1	JQ703055.1; A2d1; Asia_NE_America_N; 37; C64T; A73G; T146C	16559	Asia_NE_America_N	A2d1	A	A	37
1254	JQ703056.1	JQ703056.1; H2a2b; Asia_W_Europe; 9; A263G; C309CCT; T310C	16570	Asia_W_Europe	H2a2b	H	H	9
1255	JQ703057.1	JQ703057.1; H5+16311; Asia_W_Europe; 12; C150T; A263G; C309CCT	16570	Asia_W_Europe	H5+16311	H	H	12
1256	JQ703058.1	JQ703058.1; W3a1c; Asia_W; 38; A73G; A189G; C194T	16570	Asia_W	W3a1c	W	W	38
1257	JQ703059.1	JQ703059.1; HV; Asia_W; 16; T195C; A263G; C309CCT	16570	Asia_W	HV	HV	HV	16
1258	JQ703060.1	JQ703060.1; A2f1a; Asia_NE_America_N; 39; C64T; A73G; T146C	16567	Asia_NE_America_N	A2f1a	A	A	39
1259	JQ703061.1	JQ703061.1; H6a1a5; Asia_W_Europe; 18; T239C; A263G; C309CCCT	16573	Asia_W_Europe	H6a1a5	H	H	18
1260	JQ703062.1	JQ703062.1; T2b7a1; Asia_W; 40; A73G; T152C; A263G	16568	Asia_W	T2b7a1	T	T	40
1261	JQ703063.1	JQ703063.1; M1a1i; Africa_E_Nile Valley; 48; T63C; G66T; A73G	16569	Africa_E_Nile Valley	M1a1i	M	M1	48
1262	JQ703064.1	JQ703064.1; K2b1b; Asia_W; 38; A73G; T146C; T195C	16569	Asia_W	K2b1b	K	K	38
1263	JQ703065.1	JQ703065.1; L2a1n; Africa_W_C; 54; A73G; G143A; T146C	16568	Africa_W_C	L2a1n	L2	L2	54
1264	JQ703066.1	JQ703066.1; K2b1a1; Asia_W; 36; A73G; T146C; A263G	16569	Asia_W	K2b1a1	K	K	36
1265	JQ703067.1	JQ703067.1; K1c2; Asia_W; 35; A73G; T146C; T152C	16568	Asia_W	K1c2	K	K	35
1266	JQ703068.1	JQ703068.1; H2a2b; Asia_W_Europe; 11; A263G; C309CCT; T310C	16570	Asia_W_Europe	H2a2b	H	H	11
1267	JQ703069.1	JQ703069.1; K1a1b1a; Asia_W; 38; A73G; C114T; A263G	16569	Asia_W	K1a1b1a	K	K	38
1268	JQ703070.1	JQ703070.1; H46a; Asia_W_Europe; 14; T152C; A263G; C309CCT	16568	Asia_W_Europe	H46a	H	H	14
1269	JQ703071.1	JQ703071.1; H11a2a2; Asia_W_Europe; 19; T195C; A263G; C315CC	16569	Asia_W_Europe	H11a2a2	H	H	19
1270	JQ703072.1	JQ703072.1; K2a2a1; Asia_W; 37; A73G; T146C; T152C	16569	Asia_W	K2a2a1	K	K	37
1271	JQ703073.1	JQ703073.1; K1a1b2b; Asia_W; 39; A73G; A189G; T195C	16570	Asia_W	K1a1b2b	K	K	39

Supplementary Table S4 Human mitochondrial database (hMITO DB v1.0) metadata[a]

Row	Name (accession no.)	Description	Size	Geo_Region	Haplogroup	Macro_ Haplo	Macro_ Haplo2	Total Variants
1272	JQ703074.1	JQ703074.1; U5a1b1a2; Asia_W_Europe_C; 32; A73G; A263G; C315CC	16569	Asia_W_Europe_C	U5a1b1a2	U	U5	32
1273	JQ703075.1	JQ703075.1; K1a9; Asia_W; 34; A73G; T152C; T195C	16570	Asia_W	K1a9	K	K	34
1274	JQ703076.1	JQ703076.1; W5; Asia_W; 40; A56ATC; TC63-; G66T	16569	Asia_W	W5	W	W	40
1275	JQ703077.1	JQ703077.1; H52; Asia_W_Europe; 12; T152C; A263G; C309CCT	16570	Asia_W_Europe	H52	H	H	12
1276	JQ703078.1	JQ703078.1; H7d2a; Asia_W_Europe; 14; A263G; A291AA; C315CC	16570	Asia_W_Europe	H7d2a	H	H	14
1277	JQ703079.1	JQ703079.1; H15a1; Asia_W_Europe; 13; T55C; T57C; A263G	16571	Asia_W_Europe	H15a1	H	H	13
1278	JQ703080.1	JQ703080.1; H2a1a2; Asia_W_Europe; 16; T195C; A263G; C309CCT	16570	Asia_W_Europe	H2a1a2	H	H	16
1279	JQ703081.1	JQ703081.1; H2a5b; Asia_W_Europe; 9; A263G; C315CC; A512C	16569	Asia_W_Europe	H2a5b	H	H	9
1280	JQ703082.1	JQ703082.1; H10e; Asia_W_Europe; 13; A263G; C309CCT; T310C	16570	Asia_W_Europe	H10e	H	H	13
1281	JQ703083.1	JQ703083.1; T2b; Asia_W; 34; A73G; T195C; A263G	16569	Asia_W	T2b	T	T	34
1282	JQ703084.1	JQ703084.1; W6a; Asia_W; 40; A73G; A189G; C194T	16573	Asia_W	W6a	W	W	40
1283	JQ703085.1	JQ703085.1; K1a4d; Asia_W; 34; A73G; A263G; C315CC	16571	Asia_W	K1a4d	K	K	34
1284	JQ703086.1	JQ703086.1; M1b1a; Africa_E_Nile Valley; 45; A73G; T195C; A200G	16569	Africa_E_Nile Valley	M1b1a	M	M1	45
1285	JQ703087.1	JQ703087.1; U5a2b1d; Asia_W_Europe_C; 28; A73G; A263G; C315CC	16570	Asia_W_Europe_C	U5a2b1d	U	U5	28
1286	JQ703088.1	JQ703088.1; T2a1a; Asia_W; 34; A73G; A263G; C309CCT	16570	Asia_W	T2a1a	T	T	34
1287	JQ703089.1	JQ703089.1; H1af1a; Asia_W_Europe; 13; A263G; C315CC; A750G	16569	Asia_W_Europe	H1af1a	H	H	13
1288	JQ703090.1	JQ703090.1; HV1b2; Asia_W; 21; T152C; A263G; C309CCT	16570	Asia_W	HV1b2	HV	HV	21
1289	JQ703091.1	JQ703091.1; W1+119; Asia_W; 34; A73G; T119C; T152C	16569	Asia_W	W1+119	W	W	34
1290	JQ703092.1	JQ703092.1; H27c; Asia_W_Europe; 13; A263G; C309CCT; T310C	16570	Asia_W_Europe	H27c	H	H	13
1291	JQ703093.1	JQ703093.1; X2b4; Asia_W_America_N; 32; A73G; T195C; G225A	16570	Asia_W_America_N	X2b4	X	X	32
1292	JQ703094.1	JQ703094.1; H3y; Asia_W_Europe; 13; A263G; C309CCT; T310C	16570	Asia_W_Europe	H3y	H	H	13
1293	JQ703095.1	JQ703095.1; K1c2; Asia_W; 40; A73G; T146C; T152C	16568	Asia_W	K1c2	K	K	40
1294	JQ703096.1	JQ703096.1; K1a3a1; Asia_W; 36; A73G; T245C; A263G	16572	Asia_W	K1a3a1	K	K	36
1295	JQ703097.1	JQ703097.1; H26c; Asia_W_Europe; 12; T146C; T152C; A263G	16569	Asia_W_Europe	H26c	H	H	12
1296	JQ703098.1	JQ703098.1; H1ba; Asia_W_Europe; 12; C261T; A263G; C315CC	16570	Asia_W_Europe	H1ba	H	H	12
1297	JQ703099.1	JQ703099.1; T2b3c; Asia_W; 41; A73G; C151T; A263G	16569	Asia_W	T2b3c	T	T	41
1298	JQ703100.1	JQ703100.1; K1a+195; Asia_W; 35; A73G; T152C; T195C	16569	Asia_W	K1a+195	K	K	35
1299	JQ703101.1	JQ703101.1; H5a1; Asia_W_Europe; 15; A263G; C309CCT; T310C	16568	Asia_W_Europe	H5a1	H	H	15
1300	JQ703102.1	JQ703102.1; H3h7; Asia_W_Europe; 13; A93G; A263G; C315CC	16569	Asia_W_Europe	H3h7	H	H	13
1301	JQ703103.1	JQ703103.1; H6a1a; Asia_W_Europe; 21; A93G; T239C; A263G	16571	Asia_W_Europe	H6a1a	H	H	21
1302	JQ703104.1	JQ703104.1; H1c1; Asia_W_Europe; 14; A200G; A263G; C315CC	16569	Asia_W_Europe	H1c1	H	H	14
1303	JQ703105.1	JQ703105.1; H5a1; Asia_W_Europe; 16; A93G; A263G; C315CC	16567	Asia_W_Europe	H5a1	H	H	16
1304	JQ703106.1	JQ703106.1; I2e; Asia_W_SW; 35; A73G; T152C; T199C	16570	Asia_W_SW	I2e	I	I	35
1305	JQ703107.1	JQ703107.1; H27d; Asia_W_Europe; 14; A263G; C315CC; G380A	16569	Asia_W_Europe	H27d	H	H	14
1306	JQ703108.1	JQ703108.1; H16; Asia_W_Europe; 10; A263G; C315CC; A750G	16569	Asia_W_Europe	H16	H	H	10
1307	JQ703109.1	JQ703109.1; H1e1a; Asia_W_Europe; 15; A263G; C309CCT; T310C	16570	Asia_W_Europe	H1e1a	H	H	15
1308	JQ703110.1	JQ703110.1; H5s; Asia_W_Europe; 14; A263G; C309CCCT; T310C	16571	Asia_W_Europe	H5s	H	H	14
1309	JQ703111.1	JQ703111.1; H55a; Asia_W_Europe; 13; A263G; C309CCT; T310C	16570	Asia_W_Europe	H55a	H	H	13
1310	JQ703112.1	JQ703112.1; H1+16189; Asia_W_Europe; 13; T152C; A263G; T310C	16568	Asia_W_Europe	H1+16189	H	H	13
1311	JQ703113.1	JQ703113.1; HV0+195; Asia_W; 13; T72C; T195C; A263G	16570	Asia_W	HV0+195	HV	HV	13
1312	JQ703114.1	JQ703114.1; U5a1b; Asia_W_Europe_C; 29; A73G; A201G; A263G	16579	Asia_W_Europe_C	U5a1b	U	U5	29

Supplementary Table S4 Human mitochondrial database (hMITO DB v1.0) metadata[a]

Row	Name (accession no.)	Description	Size	Geo_Region	Haplogroup	Macro_ Haplo	Macro_ Haplo2	Total Variants
1313	JQ703115.1	JQ703115.1; H1e2c; Asia_W_Europe; 13; A73G; A263G; C315CC	16570	Asia_W_Europe	H1e2c	H	H	13
1314	JQ703116.1	JQ703116.1; V2b; Europe_S; 17; T72C; T195C; A263G	16568	Europe_S	V2b	V	V	17
1315	JQ703117.1	JQ703117.1; H10a1; Asia_W_Europe; 12; T199C; A263G; C315CC	16569	Asia_W_Europe	H10a1	H	H	12
1316	JQ703118.1	JQ703118.1; H3i; Asia_W_Europe; 11; T152C; A263G; C315CC	16569	Asia_W_Europe	H3i	H	H	11
1317	JQ703119.1	JQ703119.1; H3z1; Asia_W_Europe; 14; A73G; A263G; T293C	16569	Asia_W_Europe	H3z1	H	H	14
1318	JQ703120.1	JQ703120.1; H7; Asia_W_Europe; 14; T152C; A263G; C315CC	16569	Asia_W_Europe	H7	H	H	14
1319	JQ703121.1	JQ703121.1; H1c2; Asia_W_Europe; 14; A263G; C315CC; T477C	16569	Asia_W_Europe	H1c2	H	H	14
1320	JQ703122.1	JQ703122.1; K1c2a; Asia_W; 40; A73G; T146C; T152C	16567	Asia_W	K1c2a	K	K	40
1321	JQ703123.1	JQ703123.1; T2g1a1; Asia_W; 40; A73G; T152C; A200G	16569	Asia_W	T2g1a1	T	T	40
1322	JQ703124.1	JQ703124.1; K1c2; Asia_W; 37; A73G; T146C; T152C	16568	Asia_W	K1c2	K	K	37
1323	JQ703125.1	JQ703125.1; H1e2c; Asia_W_Europe; 15; A73G; A200G; A263G	16569	Asia_W_Europe	H1e2c	H	H	15
1324	JQ703126.1	JQ703126.1; H1c19; Asia_W_Europe; 11; A263G; C315CC; T477C	16569	Asia_W_Europe	H1c19	H	H	11
1325	JQ703127.1	JQ703127.1; H4a1c1a; Asia_W_Europe; 19; A263G; C309CCT; T310C	16568	Asia_W_Europe	H4a1c1a	H	H	19
1326	JQ703128.1	JQ703128.1; H24a; Asia_W_Europe; 10; A263G; C315CC; A750G	16569	Asia_W_Europe	H24a	H	H	10
1327	JQ703129.1	JQ703129.1; H1af1b; Asia_W_Europe; 14; A263G; C309CCCT; T310C	16571	Asia_W_Europe	H1af1b	H	H	14
1328	JQ703130.1	JQ703130.1; K1b1a1; Asia_W; 42; A73G; T152C; A263G	16571	Asia_W	K1b1a1	K	K	42
1329	JQ703131.1	JQ703131.1; H4a1a4b; Asia_W_Europe; 20; T195C; A263G; C315CC	16567	Asia_W_Europe	H4a1a4b	H	H	20
1330	JQ703132.1	JQ703132.1; H1bk; Asia_W_Europe; 12; A263G; C309CCT; T310C	16568	Asia_W_Europe	H1bk	H	H	12
1331	JQ703133.1	JQ703133.1; K1a; Asia_W; 39; A73G; T152C; A263G	16572	Asia_W	K1a	K	K	39
1332	JQ703134.1	JQ703134.1; H4a1a4a; Asia_W_Europe; 20; T195C; A263G; C309CCT	16568	Asia_W_Europe	H4a1a4a	H	H	20
1333	JQ703135.1	JQ703135.1; H1a3b; Asia_W_Europe; 16; A73G; T195C; A263G	16567	Asia_W_Europe	H1a3b	H	H	16
1334	JQ703136.1	JQ703136.1; K2a7; Asia_W; 34; A73G; T146C; T152C	16567	Asia_W	K2a7	K	K	34
1335	JQ703137.1	JQ703137.1; H1e4a; Asia_W_Europe; 13; A263G; C315CC; A750G	16569	Asia_W_Europe	H1e4a	H	H	13
1336	JQ703138.1	JQ703138.1; L3e2b1a; Africa_E; 33; A73G; C150T; T195C	16569	Africa_E	L3e2b1a	L3	L3	33
1337	JQ703139.1	JQ703139.1; HV0+195; Asia_W; 17; T72C; T195C; A263G	16570	Asia_W	HV0+195	HV	HV	17
1338	JQ703140.1	JQ703140.1; H3ap; Asia_W_Europe; 11; A263G; C309CCT; T310C	16570	Asia_W_Europe	H3ap	H	H	11
1339	JQ703141.1	JQ703141.1; H3b+16129; Asia_W_Europe; 12; A263G; C315CC; A523ACA	16571	Asia_W_Europe	H3b+16129	H	H	12
1340	JQ703142.1	JQ703142.1; H1c3a; Asia_W_Europe; 17; T146C; A257G; A263G	16570	Asia_W_Europe	H1c3a	H	H	17
1341	JQ703143.1	JQ703143.1; H1; Asia_W_Europe; 14; A263G; C309CCT; T310C	16570	Asia_W_Europe	H1	H	H	14
1342	JQ703144.1	JQ703144.1; H2a1a; Asia_W_Europe; 10; A263G; T293C; C315CC	16569	Asia_W_Europe	H2a1a	H	H	10
1343	JQ703145.1	JQ703145.1; H2a1e; Asia_W_Europe; 9; A263G; C309CCT; T310C	16570	Asia_W_Europe	H2a1e	H	H	9
1344	JQ703146.1	JQ703146.1; K2a3; Asia_W; 34; A73G; T146C; T152C	16569	Asia_W	K2a3	K	K	34
1345	JQ703147.1	JQ703147.1; H1j6; Asia_W_Europe; 13; A263G; C315CC; G709A	16569	Asia_W_Europe	H1j6	H	H	13
1346	JQ703148.1	JQ703148.1; H1bg; Asia_W_Europe; 13; A263G; C315CC; A750G	16569	Asia_W_Europe	H1bg	H	H	13
1347	JQ703149.1	JQ703149.1; T2a1b1a1; Asia_W; 40; A73G; A263G; C315CC	16569	Asia_W	T2a1b1a1	T	T	40
1348	JQ703150.1	JQ703150.1; H5b1; Asia_W_Europe; 15; T146C; T195C; A263G	16570	Asia_W_Europe	H5b1	H	H	15
1349	JQ703151.1	JQ703151.1; H1ae1; Asia_W_Europe; 13; A263G; C315CC; A750G	16569	Asia_W_Europe	H1ae1	H	H	13
1350	JQ703152.1	JQ703152.1; H5a1; Asia_W_Europe; 14; C186T; A263G; C315CC	16567	Asia_W_Europe	H5a1	H	H	14
1351	JQ703153.1	JQ703153.1; H1c1+16093; Asia_W_Europe; 13; A263G; C315CC; T477C	16569	Asia_W_Europe	H1c1+16093	H	H	13
1352	JQ703154.1	JQ703154.1; H27; Asia_W_Europe; 13; A263G; C315CC; A750G	16569	Asia_W_Europe	H27	H	H	13
1353	JQ703155.1	JQ703155.1; H71; Asia_W_Europe; 15; A263G; C309CCCT; T310C	16571	Asia_W_Europe	H71	H	H	15

Supplementary Table S4 Human mitochondrial database (hMITO DB v1.0) metadata[a]

Row	Name (accession no.)	Description	Size	Geo_Region	Haplogroup	Macro_ Haplo	Macro_ Haplo2	Total Variants
1354	JQ703156.1	JQ703156.1; H2a2a1; Asia_W_Europe; 3; C315CC; G7805A; A8679G	16569	Asia_W_Europe	H2a2a1	H	H	3
1355	JQ703157.1	JQ703157.1; H35; Asia_W_Europe; 11; A263G; C309CCT; T310C	16571	Asia_W_Europe	H35	H	H	11
1356	JQ703158.1	JQ703158.1; H4a1c1a; Asia_W_Europe; 20; A263G; C309CCCT; T310C	16568	Asia_W_Europe	H4a1c1a	H	H	20
1357	JQ703159.1	JQ703159.1; K1c2; Asia_W; 37; A73G; T146C; T152C	16568	Asia_W	K1c2	K	K	37
1358	JQ703160.1	JQ703160.1; K1a1a2a; Asia_W; 36; A73G; A263G; C315CC	16570	Asia_W	K1a1a2a	K	K	36
1359	JQ703161.1	JQ703161.1; H6a1a; Asia_W_Europe; 14; T239C; A263G; C309CCT	16570	Asia_W_Europe	H6a1a	H	H	14
1360	JQ703162.1	JQ703162.1; X2b+226; Asia_W_America_N; 35; A73G; T195C; C198T	16570	Asia_W_America_N	X2b+226	X	X	35
1361	JQ703163.1	JQ703163.1; H11a; Asia_W_Europe; 15; G185A; T195C; A263G	16571	Asia_W_Europe	H11a	H	H	15
1362	JQ703164.1	JQ703164.1; HV+16311; Asia_W_Europe; 14; A263G; C309CCT; T310C	16570	Asia_W_Europe	HV+16311	H	H	14
1363	JQ703165.1	JQ703165.1; K1a1b1a; Asia_W; 37; A73G; C114T; T152C	16569	Asia_W	K1a1b1a	K	K	37
1364	JQ703166.1	JQ703166.1; H1c2; Asia_W_Europe; 13; T195C; A263G; C315CC	16569	Asia_W_Europe	H1c2	H	H	13
1365	JQ703167.1	JQ703167.1; H11a; Asia_W_Europe; 16; T195C; A263G; C309CCT	16570	Asia_W_Europe	H11a	H	H	16
1366	JQ703168.1	JQ703168.1; K1a4a1a2; Asia_W; 39; A73G; A263G; C315CC	16571	Asia_W	K1a4a1a2	K	K	39
1367	JQ703169.1	JQ703169.1; H13a1a1a; Asia_W_Europe; 17; T152C; A263G; C309CCT	16570	Asia_W_Europe	H13a1a1a	H	H	17
1368	JQ703170.1	JQ703170.1; H1bb; Asia_W_Europe; 13; T152C; T199C; A263G	16569	Asia_W_Europe	H1bb	H	H	13
1369	JQ703171.1	JQ703171.1; K1a4a1b2; Asia_W; 35; A73G; A263G; C309CCT	16570	Asia_W	K1a4a1b2	K	K	35
1370	JQ703172.1	JQ703172.1; H11a3; Asia_W_Europe; 15; T195C; A263G; C309CCT	16570	Asia_W_Europe	H11a3	H	H	15
1371	JQ703173.1	JQ703173.1; H2a2a2; Asia_W_Europe; 6; T152C; A263G; C309CCT	16570	Asia_W_Europe	H2a2a2	H	H	6
1372	JQ703174.1	JQ703174.1; H1c6; Asia_W_Europe; 12; A263G; C309CCT; T310C	16570	Asia_W_Europe	H1c6	H	H	12
1373	JQ703175.1	JQ703175.1; H11a2; Asia_W_Europe; 18; T195C; A263G; C315CC	16569	Asia_W_Europe	H11a2	H	H	18
1374	JQ703176.1	JQ703176.1; H1bk; Asia_W_Europe; 13; A93G; A95C; A263G	16567	Asia_W_Europe	H1bk	H	H	13
1375	JQ703177.1	JQ703177.1; H7c3; Asia_W_Europe; 14; A263G; C309CCT; T310C	16570	Asia_W_Europe	H7c3	H	H	14
1376	JQ703178.1	JQ703178.1; H72; Asia_W_Europe; 13; A263G; C315CC; A750G	16569	Asia_W_Europe	H72	H	H	13
1377	JQ703179.1	JQ703179.1; H3p; Asia_W_Europe; 17; T146C; A200G; A263G	16570	Asia_W_Europe	H3p	H	H	17
1378	JQ703180.1	JQ703180.1; HV6; Asia_W; 15; A263G; C309CCT; T310C	16570	Asia_W	HV6	HV	HV	15
1379	JQ703181.1	JQ703181.1; H66a1; Asia_W_Europe; 12; A263G; C315CC; A750G	16569	Asia_W_Europe	H66a1	H	H	12
1380	JQ703182.1	JQ703182.1; J1b1a1e; Asia_W; 40; A73G; T146C; C242T	16570	Asia_W	J1b1a1e	J	J	40
1381	JQ703183.1	JQ703183.1; H11a2a1; Asia_W_Europe; 17; T195C; A263G; C309CCT	16570	Asia_W_Europe	H11a2a1	H	H	17
1382	JQ703184.1	JQ703184.1; K2b1a; Asia_W; 35; A73G; T146C; A263G	16569	Asia_W	K2b1a	K	K	35
1383	JQ703185.1	JQ703185.1; H3v1; Asia_W_Europe; 15; A263G; C315CC; T408A	16569	Asia_W_Europe	H3v1	H	H	15
1384	JQ703186.1	JQ703186.1; H5c2; Asia_W_Europe; 12; A263G; C315CC; C456T	16569	Asia_W_Europe	H5c2	H	H	12
1385	JQ703187.1	JQ703187.1; U5a2c; Asia_W_Europe_C; 25; A73G; A263G; C315CC	16569	Asia_W_Europe_C	U5a2c	U	U5	25
1386	JQ703188.1	JQ703188.1; H17a1; Asia_W_Europe; 14; A263G; C309CCT; T310C	16570	Asia_W_Europe	H17a1	H	H	14
1387	JQ703189.1	JQ703189.1; K1c1g; Asia_W; 36; A73G; T146C; T152C	16569	Asia_W	K1c1g	K	K	36
1388	JQ703190.1	JQ703190.1; H1e2c; Asia_W_Europe; 14; A73G; A263G; C309CCT	16572	Asia_W_Europe	H1e2c	H	H	14
1389	JQ703191.1	JQ703191.1; H2a2; Asia_W_Europe; 8; A263G; C309CCCT; T310C	16571	Asia_W_Europe	H2a2	H	H	8
1390	JQ703192.1	JQ703192.1; H2a3b; Asia_W_Europe; 10; A263G; C315CC; A750G	16569	Asia_W_Europe	H2a3b	H	H	10
1391	JQ703193.1	JQ703193.1; H13a1a3; Asia_W_Europe; 16; A263G; C309CCCT; T310C	16571	Asia_W_Europe	H13a1a3	H	H	16
1392	JQ703194.1	JQ703194.1; K1b1a2; Asia_W; 45; A73G; T152C; T195C	16571	Asia_W	K1b1a2	K	K	45
1393	JQ703195.1	JQ703195.1; H4a1a4b; Asia_W_Europe; 20; T195C; A263G; C315CC	16567	Asia_W_Europe	H4a1a4b	H	H	20
1394	JQ703196.1	JQ703196.1; H1e4; Asia_W_Europe; 11; A263G; C315CC; A750G	16569	Asia_W_Europe	H1e4	H	H	11

Supplementary Table S4 Human mitochondrial database (hMITO DB v1.0) metadata[a]

Row	Name (accession no.)	Description	Size	Geo_Region	Haplogroup	Macro_ Haplo	Macro_ Haplo2	Total Variants
1395	JQ703197.1	JQ703197.1; K2b1b; Asia_W; 40; A73G; T146C; T195C	16569	Asia_W	K2b1b	K	K	40
1396	JQ703198.1	JQ703198.1; H1bb; Asia_W_Europe; 13; T152C; A263G; C315CC	16571	Asia_W_Europe	H1bb	H	H	13
1397	JQ703199.1	JQ703199.1; H1+16239; Asia_W_Europe; 11; A263G; C315CC; A750G	16569	Asia_W_Europe	H1+16239	H	H	11
1398	JQ703200.1	JQ703200.1; K1c2; Asia_W; 38; A73G; T146C; T152C	16569	Asia_W	K1c2	K	K	38
1399	JQ703201.1	JQ703201.1; H27; Asia_W_Europe; 12; A263G; C309CCCT; T310C	16571	Asia_W_Europe	H27	H	H	12
1400	JQ703202.1	JQ703202.1; HV9; Asia_W; 13; A263G; C309CCT; T310C	16570	Asia_W	HV9	HV	HV	13
1401	JQ703203.1	JQ703203.1; U2e1a1b; Asia_S_W_Europe; 42; A73G; A108G; T152C	16570	Asia_S_W_Europe	U2e1a1b	U	U2	42
1402	JQ703204.1	JQ703204.1; H2a1b1; Asia_W_Europe; 11; A210G; A263G; C309CCCT	16571	Asia_W_Europe	H2a1b1	H	H	11
1403	JQ703205.1	JQ703205.1; H6a1a3a; Asia_W_Europe; 18; T195C; T239C; A263G	16571	Asia_W_Europe	H6a1a3a	H	H	18
1404	JQ703206.1	JQ703206.1; K1b2b; Asia_W; 39; A73G; T146C; T195C	16572	Asia_W	K1b2b	K	K	39
1405	JQ703207.1	JQ703207.1; H1e2b; Asia_W_Europe; 14; A263G; C309CCCT; T310C	16571	Asia_W_Europe	H1e2b	H	H	14
1406	JQ703208.1	JQ703208.1; H13a1a2a; Asia_W_Europe; 20; A263G; C309CCT; T310C	16570	Asia_W_Europe	H13a1a2a	H	H	20
1407	JQ703209.1	JQ703209.1; H2a2b1a1; Asia_W_Europe; 11; A263G; C309CCT; T310C	16570	Asia_W_Europe	H2a2b1a1	H	H	11
1408	JQ703210.1	JQ703210.1; H4a1a4b2; Asia_W_Europe; 21; T195C; A263G; C315CC	16567	Asia_W_Europe	H4a1a4b2	H	H	21
1409	JQ703211.1	JQ703211.1; J1b2; Asia_W; 32; A73G; C150T; T152C	16569	Asia_W	J1b2	J	J	32
1410	JQ703212.1	JQ703212.1; K1a4a1a2; Asia_W; 40; A73G; A263G; C309CCT	16573	Asia_W	K1a4a1a2	K	K	40
1411	JQ703213.1	JQ703213.1; H2a1a; Asia_W_Europe; 12; T152C; A263G; C309CCT	16568	Asia_W_Europe	H2a1a	H	H	12
1412	JQ703214.1	JQ703214.1; H2a1e1a1; Asia_W_Europe; 12; A263G; C315CC; C575T	16569	Asia_W_Europe	H2a1e1a1	H	H	12
1413	JQ703215.1	JQ703215.1; V14; Europe_S; 17; T72C; A263G; C315CC	16569	Europe_S	V14	V	V	17
1414	JQ703216.1	JQ703216.1; H1q; Asia_W_Europe; 12; A263G; C315CC; A523ACACA	16573	Asia_W_Europe	H1q	H	H	12
1415	JQ703217.1	JQ703217.1; H7a1b; Asia_W_Europe; 13; A263G; C309CCT; T310C	16570	Asia_W_Europe	H7a1b	H	H	13
1416	JQ703218.1	JQ703218.1; H1j; Asia_W_Europe; 13; A263G; C309CCT; T310C	16570	Asia_W_Europe	H1j	H	H	13
1417	JQ703219.1	JQ703219.1; H1h1; Asia_W_Europe; 15; A263G; C309CCT; T310C	16570	Asia_W_Europe	H1h1	H	H	15
1418	JQ703220.1	JQ703220.1; H1y; Asia_W_Europe; 13; A263G; C309CCT; T310C	16570	Asia_W_Europe	H1y	H	H	13
1419	JQ703221.1	JQ703221.1; H3; Asia_W_Europe; 13; A263G; C309CCT; T310C	16570	Asia_W_Europe	H3	H	H	13
1420	JQ703222.1	JQ703222.1; H1+16355; Asia_W_Europe; 13; A263G; C315CC; A750G	16569	Asia_W_Europe	H1+16355	H	H	13
1421	JQ703223.1	JQ703223.1; H2a1b2; Asia_W_Europe; 13; T72G; A263G; C309CCT	16570	Asia_W_Europe	H2a1b2	H	H	13
1422	JQ703224.1	JQ703224.1; H1at1; Asia_W_Europe; 12; A263G; C309CCT; T310C	16570	Asia_W_Europe	H1at1	H	H	12
1423	JQ703225.1	JQ703225.1; A2+(64); Asia_NE_America_N; 35; C64T; A73G; T146C	16567	Asia_NE_America_N	A2+(64)	A	A	35
1424	JQ703226.1	JQ703226.1; H1e1a; Asia_W_Europe; 14; A263G; C309CCCT; T310C	16571	Asia_W_Europe	H1e1a	H	H	14
1425	JQ703227.1	JQ703227.1; U5a1b1d1; Asia_W_Europe_C; 29; A73G; C198T; A263G	16569	Asia_W_Europe_C	U5a1b1d1	U	U5	29
1426	JQ703228.1	JQ703228.1; H5+16192; Asia_W_Europe; 12; A263G; C315CC; C456T	16569	Asia_W_Europe	H5+16192	H	H	12
1427	JQ703229.1	JQ703229.1; A2f2; Asia_NE_America_N; 34; C64T; A73G; A153G	16567	Asia_NE_America_N	A2f2	A	A	34
1428	JQ703230.1	JQ703230.1; H3q; Asia_W_Europe; 11; A263G; C315CC; A750G	16569	Asia_W_Europe	H3q	H	H	11
1429	JQ703231.1	JQ703231.1; H1g1; Asia_W_Europe; 14; T152C; A263G; C315CC	16569	Asia_W_Europe	H1g1	H	H	14
1430	JQ703232.1	JQ703232.1; H6a2; Asia_W_Europe; 16; T239C; A263G; C309CCCT	16571	Asia_W_Europe	H6a2	H	H	16
1431	JQ703233.1	JQ703233.1; H7a2; Asia_W_Europe; 15; A263G; C309CCT; T310C	16579	Asia_W_Europe	H7a2	H	H	15
1432	JQ703234.1	JQ703234.1; H41a; Asia_W_Europe; 14; C262T; A263G; C309CCT	16570	Asia_W_Europe	H41a	H	H	14
1433	JQ703235.1	JQ703235.1; H11a3; Asia_W_Europe; 15; T195C; A263G; C309CCT	16570	Asia_W_Europe	H11a3	H	H	15
1434	JQ703236.1	JQ703236.1; H5a1; Asia_W_Europe; 13; C150T; A263G; C315CC	16567	Asia_W_Europe	H5a1	H	H	13
1435	JQ703237.1	JQ703237.1; H10a1b; Asia_W_Europe; 16; A263G; C309CCT; T310C	16570	Asia_W_Europe	H10a1b	H	H	16

Supplementary Table S4 Human mitochondrial database (hMITO DB v1.0) metadata[a]

Row	Name (accession no.)	Description	Size	Geo_Region	Haplogroup	Macro_ Haplo	Macro_ Haplo2	Total Variants
1436	JQ703238.1	JQ703238.1; H1a4; Asia_W_Europe; 15; A73G; A263G; C309CCT	16570	Asia_W_Europe	H1a4	H	H	15
1437	JQ703239.1	JQ703239.1; H26; Asia_W_Europe; 11; A263G; C309CCT; T310C	16570	Asia_W_Europe	H26	H	H	11
1438	JQ703240.1	JQ703240.1; T1a1; Asia_W; 37; A73G; T152C; T195C	16570	Asia_W	T1a1	T	T	37
1439	JQ703241.1	JQ703241.1; H5b2; Asia_W_Europe; 13; A263G; C309CCT; T310C	16570	Asia_W_Europe	H5b2	H	H	13
1440	JQ703242.1	JQ703242.1; W1; Asia_W; 34; A73G; A189G; T195C	16570	Asia_W	W1	W	W	34
1441	JQ703243.1	JQ703243.1; H1j1a; Asia_W_Europe; 16; A93G; A263G; C309CCCT	16571	Asia_W_Europe	H1j1a	H	H	16
1442	JQ703244.1	JQ703244.1; H1bt1; Asia_W_Europe; 12; A263G; C315CC; A750G	16569	Asia_W_Europe	H1bt1	H	H	12
1443	JQ703245.1	JQ703245.1; H1y; Asia_W_Europe; 14; A263G; C309CCT; T310C	16570	Asia_W_Europe	H1y	H	H	14
1444	JQ703246.1	JQ703246.1; H3g2; Asia_W_Europe; 14; T152C; T204C; A263G	16569	Asia_W_Europe	H3g2	H	H	14
1445	JQ703247.1	JQ703247.1; H8c; Asia_W_Europe; 19; T146C; T152C; T195C	16571	Asia_W_Europe	H8c	H	H	19
1446	JQ703248.1	JQ703248.1; H95a; Asia_W_Europe; 14; A263G; C315CC; A750G	16569	Asia_W_Europe	H95a	H	H	14
1447	JQ703249.1	JQ703249.1; J1b1a1a; Asia_W; 40; A73G; A215G; C242T	16570	Asia_W	J1b1a1a	J	J	40
1448	JQ703250.1	JQ703250.1; HV1b2; Asia_W; 21; T152C; A263G; C309CCT	16570	Asia_W	HV1b2	HV	HV	21
1449	JQ703251.1	JQ703251.1; H1i2; Asia_W_Europe; 14; T152C; A263G; C315CC	16569	Asia_W_Europe	H1i2	H	H	14
1450	JQ703252.1	JQ703252.1; H14a; Asia_W_Europe; 16; A263G; C309CCT; T310C	16569	Asia_W_Europe	H14a	H	H	16
1451	JQ703253.1	JQ703253.1; H11a+152; Asia_W_Europe; 14; T152C; T195C; A263G	16569	Asia_W_Europe	H11a+152	H	H	14
1452	JQ703254.1	JQ703254.1; H1b1f; Asia_W_Europe; 15; A263G; C315CC; CA522d	16567	Asia_W_Europe	H1b1f	H	H	15
1453	JQ703255.1	JQ703255.1; K1a; Asia_W; 32; A73G; A263G; C309CCT	16572	Asia_W	K1a	K	K	32
1454	JQ703256.1	JQ703256.1; H1au1a; Asia_W_Europe; 12; A263G; C315CC; A750G	16569	Asia_W_Europe	H1au1a	H	H	12
1455	JQ703257.1	JQ703257.1; H5; Asia_W_Europe; 11; A263G; C315CC; C456T	16569	Asia_W_Europe	H5	H	H	11
1456	JQ703258.1	JQ703258.1; H3k; Asia_W_Europe; 13; T152C; T195C; A263G	16570	Asia_W_Europe	H3k	H	H	13
1457	JQ703259.1	JQ703259.1; H11a1; Asia_W_Europe; 15; T195C; A263G; C315CC	16569	Asia_W_Europe	H11a1	H	H	15
1458	JQ703260.1	JQ703260.1; H3v2; Asia_W_Europe; 17; A73G; A263G; C315CC	16569	Asia_W_Europe	H3v2	H	H	17
1459	JQ703261.1	JQ703261.1; K1c1b; Asia_W; 35; A73G; T146C; T152C	16569	Asia_W	K1c1b	K	K	35
1460	JQ703262.1	JQ703262.1; H1aj; Asia_W_Europe; 13; A263G; C309CCT; T310C	16570	Asia_W_Europe	H1aj	H	H	13
1461	JQ703263.1	JQ703263.1; T2b22; Asia_W; 38; A73G; T152C; A263G	16570	Asia_W	T2b22	T	T	38
1462	JQ703264.1	JQ703264.1; H58; Asia_W_Europe; 12; T146C; A263G; C315CC	16569	Asia_W_Europe	H58	H	H	12
1463	JQ703265.1	JQ703265.1; H3j; Asia_W_Europe; 12; T152C; A263G; C315CC	16569	Asia_W_Europe	H3j	H	H	12
1464	JQ703266.1	JQ703266.1; H5s; Asia_W_Europe; 14; A263G; C309CCT; T310C	16570	Asia_W_Europe	H5s	H	H	14
1465	JQ703267.1	JQ703267.1; K1a+195; Asia_W; 36; A73G; T195C; A263G	16571	Asia_W	K1a+195	K	K	36
1466	JQ703268.1	JQ703268.1; H1b2a1; Asia_W_Europe; 19; A183G; A263G; C309CCCT	16573	Asia_W_Europe	H1b2a1	H	H	19
1467	JQ703269.1	JQ703269.1; H5a1; Asia_W_Europe; 13; A263G; C315CC; C456T	16567	Asia_W_Europe	H5a1	H	H	13
1468	JQ703270.1	JQ703270.1; H1i; Asia_W_Europe; 15; T152C; T252C; A263G	16570	Asia_W_Europe	H1i	H	H	15
1469	JQ703271.1	JQ703271.1; H6a1a; Asia_W_Europe; 15; T239C; A263G; C315CC	16569	Asia_W_Europe	H6a1a	H	H	15
1470	JQ703272.1	JQ703272.1; H3ad; Asia_W_Europe; 10; A263G; C315CC; A750G	16569	Asia_W_Europe	H3ad	H	H	10
1471	JQ703273.1	JQ703273.1; H27a; Asia_W_Europe; 14; T152C; A263G; C315CC	16569	Asia_W_Europe	H27a	H	H	14
1472	JQ703274.1	JQ703274.1; H1a3; Asia_W_Europe; 14; A73G; A263G; C315CC	16567	Asia_W_Europe	H1a3	H	H	14
1473	JQ703275.1	JQ703275.1; H7b2a; Asia_W_Europe; 15; A189G; C194T; A263G	16569	Asia_W_Europe	H7b2a	H	H	15
1474	JQ703276.1	JQ703276.1; H5b2; Asia_W_Europe; 14; A263G; C309CCCT; T310C	16572	Asia_W_Europe	H5b2	H	H	14
1475	JQ703277.1	JQ703277.1; H1+152; Asia_W_Europe; 14; T152C; A263G; C309CCT	16570	Asia_W_Europe	H1+152	H	H	14
1476	JQ703278.1	JQ703278.1; H5q; Asia_W_Europe; 11; A263G; C315CC; C456T	16569	Asia_W_Europe	H5q	H	H	11

Supplementary Table S4 Human mitochondrial database (hMITO DB v1.0) metadata[a]

Row	Name (accession no.)	Description	Size	Geo_Region	Haplogroup	Macro_ Haplo	Macro_ Haplo2	Total Variants
1477	JQ703279.1	JQ703279.1; H3; Asia_W_Europe; 11; A263G; C309CCT; T310C	16570	Asia_W_Europe	H3	H	H	11
1478	JQ703280.1	JQ703280.1; U5a2d; Asia_W_Europe_C; 31; A73G; A263G; C315CC	16569	Asia_W_Europe_C	U5a2d	U	U5	31
1479	JQ703281.1	JQ703281.1; K1a10a; Asia_W; 32; A73G; T195C; A263G	16568	Asia_W	K1a10a	K	K	32
1480	JQ703282.1	JQ703282.1; H1as2; Asia_W_Europe; 13; A263G; C309CCT; T310C	16570	Asia_W_Europe	H1as2	H	H	13
1481	JQ703283.1	JQ703283.1; W5b; Asia_W; 42; C61T; G62T; T63C	16569	Asia_W	W5b	W	W	42
1482	JQ703284.1	JQ703284.1; H; Asia_W_Europe; 12; C151T; A263G; C309CCT	16570	Asia_W_Europe	H	H	H	12
1483	JQ703285.1	JQ703285.1; K1a15; Asia_W; 33; A73G; T195C; A263G	16573	Asia_W	K1a15	K	K	33
1484	JQ703286.1	JQ703286.1; H1ba; Asia_W_Europe; 11; A183G; A263G; C315CC	16569	Asia_W_Europe	H1ba	H	H	11
1485	JQ703287.1	JQ703287.1; H35; Asia_W_Europe; 16; T146C; A263G; C309CCCT	16571	Asia_W_Europe	H35	H	H	16
1486	JQ703288.1	JQ703288.1; H7d3a; Asia_W_Europe; 14; C150T; A263G; C309CCT	16570	Asia_W_Europe	H7d3a	H	H	14
1487	JQ703289.1	JQ703289.1; H3v1; Asia_W_Europe; 14; A263G; C315CC; T408A	16569	Asia_W_Europe	H3v1	H	H	14
1488	JQ703290.1	JQ703290.1; H2a2; Asia_W_Europe; 5; A263G; C309CCT; T310C	16570	Asia_W_Europe	H2a2	H	H	5
1489	JQ703291.1	JQ703291.1; H3b3; Asia_W_Europe; 15; A263G; C309CCCT; T310C	16571	Asia_W_Europe	H3b3	H	H	15
1490	JQ703292.1	JQ703292.1; K1c1d; Asia_W; 37; A73G; T146C; T152C	16566	Asia_W	K1c1d	K	K	37
1491	JQ703293.1	JQ703293.1; T2b1; Asia_W; 39; A73G; T195C; A263G	16571	Asia_W	T2b1	T	T	39
1492	JQ703294.1	JQ703294.1; T2b7a1; Asia_W; 39; A73G; T152C; A263G	16568	Asia_W	T2b7a1	T	T	39
1493	JQ703295.1	JQ703295.1; H20a2; Asia_W_Europe; 12; A263G; C315CC; A750G	16569	Asia_W_Europe	H20a2	H	H	12
1494	JQ703296.1	JQ703296.1; K1a4; Asia_W; 34; A73G; A263G; C309CCT	16572	Asia_W	K1a4	K	K	34
1495	JQ703297.1	JQ703297.1; U5a1a1d; Asia_W_Europe_C; 31; A73G; G185A; T204C	16569	Asia_W_Europe_C	U5a1a1d	U	U5	31
1496	JQ703298.1	JQ703298.1; H3z1; Asia_W_Europe; 16; A263G; T293C; C315CC	16569	Asia_W_Europe	H3z1	H	H	16
1497	JQ703299.1	JQ703299.1; H8c1; Asia_W_Europe; 21; C114T; T146C; T152C	16570	Asia_W_Europe	H8c1	H	H	21
1498	JQ703300.1	JQ703300.1; K2a6; Asia_W; 34; A73G; T146C; T152C	16569	Asia_W	K2a6	K	K	34
1499	JQ703301.1	JQ703301.1; H3ac; Asia_W_Europe; 10; A263G; C315CC; A750G	16569	Asia_W_Europe	H3ac	H	H	10
1500	JQ703302.1	JQ703302.1; H5a1a; Asia_W_Europe; 15; A93G; A263G; C315CC	16567	Asia_W_Europe	H5a1a	H	H	15
1501	JQ703303.1	JQ703303.1; H91; Asia_W_Europe; 11; T195C; A263G; C315CC	16569	Asia_W_Europe	H91	H	H	11
1502	JQ703304.1	JQ703304.1; H3an; Asia_W_Europe; 12; G251A; A263G; C315CC	16569	Asia_W_Europe	H3an	H	H	12
1503	JQ703305.1	JQ703305.1; H1+152; Asia_W_Europe; 10; T152C; A263G; C315CC	16569	Asia_W_Europe	H1+152	H	H	10
1504	JQ703306.1	JQ703306.1; H62; Asia_W_Europe; 12; T152C; A263G; C315CC	16569	Asia_W_Europe	H62	H	H	12
1505	JQ703307.1	JQ703307.1; H5; Asia_W_Europe; 14; A263G; C315CC; C456T	16569	Asia_W_Europe	H5	H	H	14
1506	JQ703308.1	JQ703308.1; K1a1b1a; Asia_W; 37; A73G; C114T; A263G	16570	Asia_W	K1a1b1a	K	K	37
1507	JQ703309.1	JQ703309.1; K1a2; Asia_W; 37; A73G; A263G; C309CCT	16570	Asia_W	K1a2	K	K	37
1508	JQ703310.1	JQ703310.1; H83; Asia_W_Europe; 11; A263G; C315CC; A750G	16569	Asia_W_Europe	H83	H	H	11
1509	JQ703311.1	JQ703311.1; H1u1; Asia_W_Europe; 11; A263G; A750G; A1438G	16568	Asia_W_Europe	H1u1	H	H	11
1510	JQ703312.1	JQ703312.1; H1n5; Asia_W_Europe; 13; T146C; T195C; A263G	16569	Asia_W_Europe	H1n5	H	H	13
1511	JQ703313.1	JQ703313.1; H7; Asia_W_Europe; 14; T152C; A263G; C309CCT	16572	Asia_W_Europe	H7	H	H	14
1512	JQ703314.1	JQ703314.1; H6a1b4; Asia_W_Europe; 16; T239C; A263G; C315CC	16569	Asia_W_Europe	H6a1b4	H	H	16
1513	JQ703315.1	JQ703315.1; H1a1; Asia_W_Europe; 14; A73G; A263G; C315CC	16569	Asia_W_Europe	H1a1	H	H	14
1514	JQ703316.1	JQ703316.1; H1ag1; Asia_W_Europe; 12; A263G; C315CC; CA522d	16567	Asia_W_Europe	H1ag1	H	H	12
1515	JQ703317.1	JQ703317.1; H1bn; Asia_W_Europe; 14; A263G; C309CCT; T310C	16570	Asia_W_Europe	H1bn	H	H	14
1516	JQ703318.1	JQ703318.1; K2a5; Asia_W; 34; A73G; T146C; T152C	16570	Asia_W	K2a5	K	K	34
1517	JQ703319.1	JQ703319.1; H1n1a; Asia_W_Europe; 18; T146C; A263G; C309CCT	16570	Asia_W_Europe	H1n1a	H	H	18

Supplementary Table S4 Human mitochondrial database (hMITO DB v1.0) metadata[a]

Row	Name (accession no.)	Description	Size	Geo_Region	Haplogroup	Macro_ Haplo	Macro_ Haplo2	Total Variants
1518	JQ703320.1	JQ703320.1; H10e; Asia_W_Europe; 14; A263G; C309CCT; T310C	16570	Asia_W_Europe	H10e	H	H	14
1519	JQ703321.1	JQ703321.1; H6a1b4; Asia_W_Europe; 15; T239C; A263G; C315CC	16569	Asia_W_Europe	H6a1b4	H	H	15
1520	JQ703322.1	JQ703322.1; H1f+16093; Asia_W_Europe; 14; A263G; C315CC; A750G	16569	Asia_W_Europe	H1f+16093	H	H	14
1521	JQ703323.1	JQ703323.1; K1a12; Asia_W; 34; A73G; T195C; A263G	16573	Asia_W	K1a12	K	K	34
1522	JQ703324.1	JQ703324.1; H1ak; Asia_W_Europe; 13; T195C; A263G; C309CCT	16570	Asia_W_Europe	H1ak	H	H	13
1523	JQ703325.1	JQ703325.1; H39b; Asia_W_Europe; 11; A263G; C309CCT; T310C	16570	Asia_W_Europe	H39b	H	H	11
1524	JQ703326.1	JQ703326.1; H5; Asia_W_Europe; 12; A263G; C309CCT; T310C	16570	Asia_W_Europe	H5	H	H	12
1525	JQ703327.1	JQ703327.1; H50; Asia_W_Europe; 10; A263G; C309CCT; T310C	16570	Asia_W_Europe	H50	H	H	10
1526	JQ703328.1	JQ703328.1; H2a2a1; Asia_W_Europe; 5; C309CCT; T310C; C12858T	16570	Asia_W_Europe	H2a2a1	H	H	5
1527	JQ703329.1	JQ703329.1; H17b; Asia_W_Europe; 10; A263G; A750G; A1438G	16568	Asia_W_Europe	H17b	H	H	10
1528	JQ703330.1	JQ703330.1; J2a1a1a2; Asia_W; 41; A73G; C150T; T152C	16568	Asia_W	J2a1a1a2	J	J	41
1529	JQ703331.1	JQ703331.1; U5a1b3; Asia_W_Europe_C; 27; A73G; A263G; C315CC	16569	Asia_W_Europe_C	U5a1b3	U	U5	27
1530	JQ703332.1	JQ703332.1; A2+(64); Asia_NE_America_N; 40; C64T; A73G; T146C	16568	Asia_NE_America_N	A2+(64)	A	A	40
1531	JQ703333.1	JQ703333.1; H1; Asia_W_Europe; 12; A263G; C309CCT; T310C	16570	Asia_W_Europe	H1	H	H	12
1532	JQ703334.1	JQ703334.1; H33a; Asia_W_Europe; 10; A263G; A750G; A1374T	16568	Asia_W_Europe	H33a	H	H	10
1533	JQ703335.1	JQ703335.1; U5a2a1+152; Asia_W_Europe_C; 30; A73G; T152C; A263G	16570	Asia_W_Europe_C	U5a2a1+152	U	U5	30
1534	JQ703336.1	JQ703336.1; H1; Asia_W_Europe; 12; A263G; C309CCT; T310C	16570	Asia_W_Europe	H1	H	H	12
1535	JQ703337.1	JQ703337.1; H1b; Asia_W_Europe; 13; A263G; C315CC; A750G	16569	Asia_W_Europe	H1b	H	H	13
1536	JQ703338.1	JQ703338.1; H31; Asia_W_Europe; 13; T146C; T195C; A263G	16569	Asia_W_Europe	H31	H	H	13
1537	JQ703339.1	JQ703339.1; H4a1a+195; Asia_W_Europe; 22; T195C; A263G; C309CCT	16568	Asia_W_Europe	H4a1a+195	H	H	22
1538	JQ703340.1	JQ703340.1; H3n; Asia_W_Europe; 15; T146C; A263G; C315CC	16569	Asia_W_Europe	H3n	H	H	15
1539	JQ703341.1	JQ703341.1; H; Asia_W_Europe; 14; A263G; C309CCT; T310C	16570	Asia_W_Europe	H	H	H	14
1540	JQ703342.1	JQ703342.1; H1c14; Asia_W_Europe; 16; A263G; C309CCCT; T310C	16571	Asia_W_Europe	H1c14	H	H	16
1541	JQ703343.1	JQ703343.1; H11b1; Asia_W_Europe; 18; T152C; T195C; A263G	16570	Asia_W_Europe	H11b1	H	H	18
1542	JQ703344.1	JQ703344.1; H2a1; Asia_W_Europe; 9; A263G; C315CC; A750G	16569	Asia_W_Europe	H2a1	H	H	9
1543	JQ703345.1	JQ703345.1; H6a1a2b1; Asia_W_Europe; 19; T239C; A263G; C309CCT	16570	Asia_W_Europe	H6a1a2b1	H	H	19
1544	JQ703346.1	JQ703346.1; H1bq; Asia_W_Europe; 16; A263G; C309CCCT; T310C	16571	Asia_W_Europe	H1bq	H	H	16
1545	JQ703347.1	JQ703347.1; K1a3; Asia_W; 34; A73G; C151T; T152C	16570	Asia_W	K1a3	K	K	34
1546	JQ703348.1	JQ703348.1; H3q; Asia_W_Europe; 16; A263G; C315CC; C356CC	16570	Asia_W_Europe	H3q	H	H	16
1547	JQ703349.1	JQ703349.1; H52; Asia_W_Europe; 11; T152C; A263G; C309CCT	16570	Asia_W_Europe	H52	H	H	11
1548	JQ703350.1	JQ703350.1; H56b; Asia_W_Europe; 13; A263G; C309CCT; T310C	16570	Asia_W_Europe	H56b	H	H	13
1549	JQ703351.1	JQ703351.1; H1; Asia_W_Europe; 14; C150T; A263G; C309CCCT	16569	Asia_W_Europe	H1	H	H	14
1550	JQ703352.1	JQ703352.1; H24a1; Asia_W_Europe; 13; A263G; C315CCC; A750G	16570	Asia_W_Europe	H24a1	H	H	13
1551	JQ703353.1	JQ703353.1; H6b1; Asia_W_Europe; 18; C44CC; T146C; T152C	16571	Asia_W_Europe	H6b1	H	H	18
1552	JQ703354.1	JQ703354.1; H7; Asia_W_Europe; 12; A263G; C315CC; A750G	16569	Asia_W_Europe	H7	H	H	12
1553	JQ703355.1	JQ703355.1; H2a5; Asia_W_Europe; 12; A263G; C309CCCT; T310C	16571	Asia_W_Europe	H2a5	H	H	12
1554	JQ703356.1	JQ703356.1; U2e1; Asia_S_W_Europe; 39; A73G; T152C; T217C	16573	Asia_S_W_Europe	U2e1	U	U2	39
1555	JQ703357.1	JQ703357.1; H1b1g; Asia_W_Europe; 18; C64T; T195C; A263G	16569	Asia_W_Europe	H1b1g	H	H	18
1556	JQ703358.1	JQ703358.1; H24a; Asia_W_Europe; 12; T152C; A263G; C315CC	16569	Asia_W_Europe	H24a	H	H	12
1557	JQ703359.1	JQ703359.1; H7b; Asia_W_Europe; 13; A263G; C309CCCT; T310C	16571	Asia_W_Europe	H7b	H	H	13
1558	JQ703360.1	JQ703360.1; H+152; Asia_W_Europe; 13; T152C; A263G; C315CC	16567	Asia_W_Europe	H+152	H	H	13

Supplementary Table S4 Human mitochondrial database (hMITO DB v1.0) metadata[a]

Row	Name (accession no.)	Description	Size	Geo_Region	Haplogroup	Macro_ Haplo	Macro_ Haplo2	Total Variants
1559	JQ703361.1	JQ703361.1; K1a4h; Asia_W; 36; A73G; A263G; C309CCT	16570	Asia_W	K1a4h	K	K	36
1560	JQ703362.1	JQ703362.1; H2a1b1; Asia_W_Europe; 13; A210G; A263G; C309CCCT	16571	Asia_W_Europe	H2a1b1	H	H	13
1561	JQ703363.1	JQ703363.1; HV6; Asia_W; 13; A263G; C309CCCT; T310C	16571	Asia_W	HV6	HV	HV	13
1562	JQ703364.1	JQ703364.1; H1b1+16362; Asia_W_Europe; 16; A263G; C315CC; CA522d	16567	Asia_W_Europe	H1b1+16362	H	H	16
1563	JQ703365.1	JQ703365.1; K1a16; Asia_W; 32; A73G; T195C; A263G	16571	Asia_W	K1a16	K	K	32
1564	JQ703366.1	JQ703366.1; H2a1e1a; Asia_W_Europe; 12; A263G; C309CCT; T310C	16570	Asia_W_Europe	H2a1e1a	H	H	12
1565	JQ703367.1	JQ703367.1; HV9; Asia_W; 12; A263G; C309CCCT; T310C	16571	Asia_W	HV9	HV	HV	12
1566	JQ703368.1	JQ703368.1; H4a1a1; Asia_W_Europe; 16; A263G; C315CC; A750G	16569	Asia_W_Europe	H4a1a1	H	H	16
1567	JQ703369.1	JQ703369.1; H51a; Asia_W_Europe; 12; A263G; C309CCT; T310C	16570	Asia_W_Europe	H51a	H	H	12
1568	JQ703370.1	JQ703370.1; HV+16311; Asia_W_Europe; 16; A263G; C309CCT; T310C	16571	Asia_W_Europe	HV+16311	H	H	16
1569	JQ703371.1	JQ703371.1; H1aq1; Asia_W_Europe; 13; A263G; C315CC; CA522d	16567	Asia_W_Europe	H1aq1	H	H	13
1570	JQ703372.1	JQ703372.1; H7b; Asia_W_Europe; 10; A263G; C315CC; A750G	16569	Asia_W_Europe	H7b	H	H	10
1571	JQ703373.1	JQ703373.1; H13a1a1a; Asia_W_Europe; 18; T152C; A263G; C309CCT	16570	Asia_W_Europe	H13a1a1a	H	H	18
1572	JQ703374.1	JQ703374.1; H5a(H5a1i); Asia_W_Europe; 2; C315CC; G16153A;	16569	Asia_W_Europe	H5a(H5a1i)	H	H	2
1573	JQ703375.1	JQ703375.1; K2b1a1a; Asia_W; 37; A73G; T146C; T152C	16569	Asia_W	K2b1a1a	K	K	37
1574	JQ703376.1	JQ703376.1; V; Europe_S; 15; T72C; A263G; C309CCT	16570	Europe_S	V	V	V	15
1575	JQ703377.1	JQ703377.1; H7d1; Asia_W_Europe; 13; A263G; C315CC; A750G	16569	Asia_W_Europe	H7d1	H	H	13
1576	JQ703378.1	JQ703378.1; K1b2a; Asia_W; 35; A73G; T146C; T195C	16573	Asia_W	K1b2a	K	K	35
1577	JQ703379.1	JQ703379.1; K1a15; Asia_W; 32; A73G; T195C; A263G	16571	Asia_W	K1a15	K	K	32
1578	JQ703380.1	JQ703380.1; H1ba; Asia_W_Europe; 11; A263G; C315CC; A750G	16569	Asia_W_Europe	H1ba	H	H	11
1579	JQ703381.1	JQ703381.1; H3n; Asia_W_Europe; 16; A263G; C309CCT; T310C	16570	Asia_W_Europe	H3n	H	H	16
1580	JQ703382.1	JQ703382.1; H3b1b1; Asia_W_Europe; 18; A153G; A263G; C309CCT	16568	Asia_W_Europe	H3b1b1	H	H	18
1581	JQ703383.1	JQ703383.1; H1c20; Asia_W_Europe; 14; A263G; C309CCCT; T310C	16571	Asia_W_Europe	H1c20	H	H	14
1582	JQ703384.1	JQ703384.1; H1a; Asia_W_Europe; 13; A73G; G143A; A263G	16569	Asia_W_Europe	H1a	H	H	13
1583	JQ703385.1	JQ703385.1; U4a2b; Asia_N_Europe_N; 30; A73G; T195C; A263G	16570	Asia_N_Europe_N	U4a2b	U	U4	30
1584	JQ703386.1	JQ703386.1; H13a1a1b; Asia_W_Europe; 16; A263G; C309CCT; T310C	16570	Asia_W_Europe	H13a1a1b	H	H	16
1585	JQ703387.1	JQ703387.1; H3v+16093; Asia_W_Europe; 14; A263G; C309CCCT; T310C	16571	Asia_W_Europe	H3v+16093	H	H	14
1586	JQ703388.1	JQ703388.1; H1cf; Asia_W_Europe; 12; A263G; C315CC; A750G	16569	Asia_W_Europe	H1cf	H	H	12
1587	JQ703389.1	JQ703389.1; H5s; Asia_W_Europe; 14; A73G; A263G; C309CCT	16570	Asia_W_Europe	H5s	H	H	14
1588	JQ703390.1	JQ703390.1; H1+16239; Asia_W_Europe; 13; A263G; C309CCT; T310C	16570	Asia_W_Europe	H1+16239	H	H	13
1589	JQ703391.1	JQ703391.1; H6a1b2b; Asia_W_Europe; 17; T239C; A263G; C309CCT	16570	Asia_W_Europe	H6a1b2b	H	H	17
1590	JQ703392.1	JQ703392.1; H3; Asia_W_Europe; 13; A263G; C315CC; A503G	16569	Asia_W_Europe	H3	H	H	13
1591	JQ703393.1	JQ703393.1; H3ao; Asia_W_Europe; 13; A263G; C315CC; A750G	16569	Asia_W_Europe	H3ao	H	H	13
1592	JQ703394.1	JQ703394.1; H5; Asia_W_Europe; 14; C150T; T204C; A263G	16571	Asia_W_Europe	H5	H	H	14
1593	JQ703395.1	JQ703395.1; T1a1; Asia_W; 34; A73G; T152C; T195C	16570	Asia_W	T1a1	T	T	34
1594	JQ703396.1	JQ703396.1; H24a1; Asia_W_Europe; 13; A263G; C315CCC; A750G	16570	Asia_W_Europe	H24a1	H	H	13
1595	JQ703397.1	JQ703397.1; H2a1a1; Asia_W_Europe; 13; A263G; C315CC; A750G	16569	Asia_W_Europe	H2a1a1	H	H	13
1596	JQ703398.1	JQ703398.1; H4a1a3; Asia_W_Europe; 21; C150T; T195C; A263G	16568	Asia_W_Europe	H4a1a3	H	H	21
1597	JQ703399.1	JQ703399.1; H1; Asia_W_Europe; 12; T195C; A263G; T292C	16569	Asia_W_Europe	H1	H	H	12
1598	JQ703400.1	JQ703400.1; H1; Asia_W_Europe; 11; A263G; C315CC; A750G	16569	Asia_W_Europe	H1	H	H	11
1599	JQ703401.1	JQ703401.1; H4a1a4b1; Asia_W_Europe; 24; T195C; A263G; C315CC	16567	Asia_W_Europe	H4a1a4b1	H	H	24

Supplementary Table S4 Human mitochondrial database (hMITO DB v1.0) metadata[a]

Row	Name (accession no.)	Description	Size	Geo_Region	Haplogroup	Macro_ Haplo	Macro_ Haplo2	Total Variants
1600	JQ703402.1	JQ703402.1; J1c8a; Asia_W; 29; A73G; G185A; G228A	16571	Asia_W	J1c8a	J	J	29
1601	JQ703403.1	JQ703403.1; H1an1; Asia_W_Europe; 14; T152C; A263G; C315CC	16569	Asia_W_Europe	H1an1	H	H	14
1602	JQ703404.1	JQ703404.1; H6a1b2; Asia_W_Europe; 22; T146C; T152C; T239C	16570	Asia_W_Europe	H6a1b2	H	H	22
1603	JQ703405.1	JQ703405.1; H5h; Asia_W_Europe; 12; A263G; C315CC; C456T	16569	Asia_W_Europe	H5h	H	H	12
1604	JQ703406.1	JQ703406.1; W1e1a; Asia_W; 41; A73G; A189G; T195C	16571	Asia_W	W1e1a	W	W	41
1605	JQ703407.1	JQ703407.1; K1a4a1b; Asia_W; 37; A73G; T146C; A263G	16570	Asia_W	K1a4a1b	K	K	37
1606	JQ703408.1	JQ703408.1; H39; Asia_W_Europe; 10; A263G; C315CC; A750G	16569	Asia_W_Europe	H39	H	H	10
1607	JQ703409.1	JQ703409.1; D4a1c; Asia_E_America_N_S; 39; A73G; T152C; A263G	16570	Asia_E_America_N_S	D4a1c	D	D	39
1608	JQ703410.1	JQ703410.1; H3c1; Asia_W_Europe; 15; A189G; A263G; C315CC	16569	Asia_W_Europe	H3c1	H	H	15
1609	JQ703411.1	JQ703411.1; H1+16189; Asia_W_Europe; 11; A263G; C315CC; A750G	16569	Asia_W_Europe	H1+16189	H	H	11
1610	JQ703412.1	JQ703412.1; K1b1b1; Asia_W; 43; A73G; T152C; A263G	16574	Asia_W	K1b1b1	K	K	43
1611	JQ703413.1	JQ703413.1; H2a2b1; Asia_W_Europe; 7; A263G; C309CCT; T310C	16570	Asia_W_Europe	H2a2b1	H	H	7
1612	JQ703414.1	JQ703414.1; H2a2b2; Asia_W_Europe; 8; A263G; C309CCT; T310C	16570	Asia_W_Europe	H2a2b2	H	H	8
1613	JQ703415.1	JQ703415.1; HV1b2; Asia_W; 22; T131C; T152C; A263G	16571	Asia_W	HV1b2	HV	HV	22
1614	JQ703416.1	JQ703416.1; H3b; Asia_W_Europe; 14; A263G; C309CCCT; T310C	16571	Asia_W_Europe	H3b	H	H	14
1615	JQ703417.1	JQ703417.1; H26a1a1; Asia_W_Europe; 15; T196C; A263G; C309CCT	16570	Asia_W_Europe	H26a1a1	H	H	15
1616	JQ703418.1	JQ703418.1; H1c1; Asia_W_Europe; 13; A263G; C315CC; T477C	16569	Asia_W_Europe	H1c1	H	H	13
1617	JQ703419.1	JQ703419.1; H1a3c1; Asia_W_Europe; 15; A73G; A263G; C315CC	16569	Asia_W_Europe	H1a3c1	H	H	15
1618	JQ703420.1	JQ703420.1; H1j; Asia_W_Europe; 13; A263G; C309CCCT; T310C	16571	Asia_W_Europe	H1j	H	H	13
1619	JQ703421.1	JQ703421.1; H13a1a1e; Asia_W_Europe; 18; T131C; A263G; C309CCT	16568	Asia_W_Europe	H13a1a1e	H	H	18
1620	JQ703422.1	JQ703422.1; H7c1; Asia_W_Europe; 15; A263G; C309CCT; T310C	16570	Asia_W_Europe	H7c1	H	H	15
1621	JQ703423.1	JQ703423.1; H; Asia_W_Europe; 11; A263G; C309CCT; T310C	16570	Asia_W_Europe	H	H	H	11
1622	JQ703424.1	JQ703424.1; H1a1b; Asia_W_Europe; 14; A73G; A263G; C315CC	16569	Asia_W_Europe	H1a1b	H	H	14
1623	JQ703425.1	JQ703425.1; H3ap; Asia_W_Europe; 15; A263G; C309CCT; T310C	16570	Asia_W_Europe	H3ap	H	H	15
1624	JQ703426.1	JQ703426.1; H1; Asia_W_Europe; 11; A93G; A263G; C315CC	16569	Asia_W_Europe	H1	H	H	11
1625	JQ703427.1	JQ703427.1; K1a3a4; Asia_W; 34; A73G; A263G; C309CCT	16570	Asia_W	K1a3a4	K	K	34
1626	JQ703428.1	JQ703428.1; H1a3c; Asia_W_Europe; 14; A73G; A93G; A263G	16569	Asia_W_Europe	H1a3c	H	H	14
1627	JQ703429.1	JQ703429.1; H27; Asia_W_Europe; 13; A263G; C309CCT; T310C	16570	Asia_W_Europe	H27	H	H	13
1628	JQ703430.1	JQ703430.1; H1c7; Asia_W_Europe; 13; A263G; C309CCT; T310C	16570	Asia_W_Europe	H1c7	H	H	13
1629	JQ703431.1	JQ703431.1; V1a1; Europe_S; 19; T72C; A263G; C309CCT	16570	Europe_S	V1a1	V	V	19
1630	JQ703432.1	JQ703432.1; H6a1a2a; Asia_W_Europe; 20; C41T; T239C; A263G	16570	Asia_W_Europe	H6a1a2a	H	H	20
1631	JQ703433.1	JQ703433.1; H2a2a1; Asia_W_Europe; 4; T195C; C315CC; A9494G	16569	Asia_W_Europe	H2a2a1	H	H	4
1632	JQ703434.1	JQ703434.1; H3k; Asia_W_Europe; 13; T152C; T195C; A263G	16570	Asia_W_Europe	H3k	H	H	13
1633	JQ703435.1	JQ703435.1; H; Asia_W_Europe; 15; A263G; C309CCT; T310C	16570	Asia_W_Europe	H	H	H	15
1634	JQ703436.1	JQ703436.1; H24a; Asia_W_Europe; 12; A263G; C315CC; A750G	16569	Asia_W_Europe	H24a	H	H	12
1635	JQ703437.1	JQ703437.1; H7e; Asia_W_Europe; 13; A263G; C309CCT; T310C	16570	Asia_W_Europe	H7e	H	H	13
1636	JQ703438.1	JQ703438.1; H1e1a; Asia_W_Europe; 13; A263G; C315CC; A750G	16569	Asia_W_Europe	H1e1a	H	H	13
1637	JQ703439.1	JQ703439.1; H3j; Asia_W_Europe; 14; T152C; A263G; C315CC	16569	Asia_W_Europe	H3j	H	H	14
1638	JQ703440.1	JQ703440.1; H27; Asia_W_Europe; 13; A263G; C309CCT; T310C	16570	Asia_W_Europe	H27	H	H	13
1639	JQ703441.1	JQ703441.1; H1c1; Asia_W_Europe; 15; A263G; C309CCT; T310C	16570	Asia_W_Europe	H1c1	H	H	15
1640	JQ703442.1	JQ703442.1; H3g1; Asia_W_Europe; 15; T152C; A263G; C315CC	16569	Asia_W_Europe	H3g1	H	H	15

Supplementary Table S4 Human mitochondrial database (hMITO DB v1.0) metadata[a]

Row	Name (accession no.)	Description	Size	Geo_Region	Haplogroup	Macro_ Haplo	Macro_ Haplo2	Total Variants
1641	JQ703443.1	JQ703443.1; H1b1+16362; Asia_W_Europe; 17; A263G; C315CC; T482C	16567	Asia_W_Europe	H1b1+16362	H	H	17
1642	JQ703444.1	JQ703444.1; K1a4a1a+195; Asia_W; 37; A73G; T195C; A263G	16569	Asia_W	K1a4a1a+195	K	K	37
1643	JQ703445.1	JQ703445.1; M52a; Asia_S; 41; A73G; A263G; C315CC	16570	Asia_S	M52a	M	M52	41
1644	JQ703446.1	JQ703446.1; T1a1j; Asia_W; 42; A73G; T152C; T195C	16571	Asia_W	T1a1j	T	T	42
1645	JQ703447.1	JQ703447.1; H5a1; Asia_W_Europe; 16; A263G; C315CC; C456T	16567	Asia_W_Europe	H5a1	H	H	16
1646	JQ703448.1	JQ703448.1; J1c5; Asia_W; 31; A73G; T152C; G185A	16569	Asia_W	J1c5	J	J	31
1647	JQ703449.1	JQ703449.1; H2a2a1; Asia_W_Europe; 4; C309CCT; T310C; G5252A	16570	Asia_W_Europe	H2a2a1	H	H	4
1648	JQ703450.1	JQ703450.1; H1a; Asia_W_Europe; 13; A73G; A263G; C309CCT	16570	Asia_W_Europe	H1a	H	H	13
1649	JQ703451.1	JQ703451.1; H11a2a1; Asia_W_Europe; 19; T195C; A263G; C309CCCT	16571	Asia_W_Europe	H11a2a1	H	H	19
1650	JQ703452.1	JQ703452.1; H10e2; Asia_W_Europe; 13; A263G; C309CCT; T310C	16570	Asia_W_Europe	H10e2	H	H	13
1651	JQ703453.1	JQ703453.1; H11a1; Asia_W_Europe; 18; T195C; A263G; C315CC	16569	Asia_W_Europe	H11a1	H	H	18
1652	JQ703454.1	JQ703454.1; H89; Asia_W_Europe; 15; A263G; C309CCT; T310C	16570	Asia_W_Europe	H89	H	H	15
1653	JQ703455.1	JQ703455.1; H66a1; Asia_W_Europe; 11; A263G; C315CC; A750G	16569	Asia_W_Europe	H66a1	H	H	11
1654	JQ703456.1	JQ703456.1; H7c6; Asia_W_Europe; 16; T146C; T152C; A263G	16570	Asia_W_Europe	H7c6	H	H	16
1655	JQ703457.1	JQ703457.1; H1b; Asia_W_Europe; 15; A263G; C309CCT; T310C	16570	Asia_W_Europe	H1b	H	H	15
1656	JQ703458.1	JQ703458.1; T2b23; Asia_W; 37; A73G; A263G; C309CCCT	16571	Asia_W	T2b23	T	T	37
1657	JQ703459.1	JQ703459.1; H1c9a; Asia_W_Europe; 16; T152C; A263G; C309CCCT	16571	Asia_W_Europe	H1c9a	H	H	16
1658	JQ703460.1	JQ703460.1; H49a; Asia_W_Europe; 13; A263G; C309CCT; T310C	16572	Asia_W_Europe	H49a	H	H	13
1659	JQ703461.1	JQ703461.1; J1c2; Asia_W; 31; A73G; G185A; A188G	16569	Asia_W	J1c2	J	J	31
1660	JQ703462.1	JQ703462.1; H3ag; Asia_W_Europe; 11; A263G; C315CC; A750G	16569	Asia_W_Europe	H3ag	H	H	11
1661	JQ703463.1	JQ703463.1; K1a; Asia_W; 36; A73G; T146C; A263G	16571	Asia_W	K1a	K	K	36
1662	JQ703464.1	JQ703464.1; J1c1c; Asia_W; 36; A73G; C150T; G185A	16567	Asia_W	J1c1c	J	J	36
1663	JQ703465.1	JQ703465.1; H1v; Asia_W_Europe; 11; A263G; C315CC; A745G	16569	Asia_W_Europe	H1v	H	H	11
1664	JQ703466.1	JQ703466.1; H1; Asia_W_Europe; 11; A263G; C309CCT; T310C	16570	Asia_W_Europe	H1	H	H	11
1665	JQ703467.1	JQ703467.1; H3b+16129; Asia_W_Europe; 14; A263G; C309CCT; T310C	16570	Asia_W_Europe	H3b+16129	H	H	14
1666	JQ703468.1	JQ703468.1; H5a1+16093; Asia_W_Europe; 14; A263G; C315CC; C456T	16567	Asia_W_Europe	H5a1+16093	H	H	14
1667	JQ703469.1	JQ703469.1; J1c3a2; Asia_W; 33; A73G; G228A; A263G	16570	Asia_W	J1c3a2	J	J	33
1668	JQ703470.1	JQ703470.1; H3a; Asia_W_Europe; 14; T152C; A263G; C309CCCT	16571	Asia_W_Europe	H3a	H	H	14
1669	JQ703471.1	JQ703471.1; H2a2a1d; Asia_W_Europe; 4; C315CC; C12135A; A14357G	16569	Asia_W_Europe	H2a2a1d	H	H	4
1670	JQ703472.1	JQ703472.1; H1a1; Asia_W_Europe; 13; A73G; A263G; C315CC	16569	Asia_W_Europe	H1a1	H	H	13
1671	JQ703473.1	JQ703473.1; H1ad; Asia_W_Europe; 14; A263G; C309CCCT; T310C	16569	Asia_W_Europe	H1ad	H	H	14
1672	JQ703474.1	JQ703474.1; H31a; Asia_W_Europe; 16; T72G; T146C; T195C	16570	Asia_W_Europe	H31a	H	H	16
1673	JQ703475.1	JQ703475.1; HV1b2; Asia_W; 21; T152C; A263G; C309CCCT	16571	Asia_W	HV1b2	HV	HV	21
1674	JQ703476.1	JQ703476.1; H1g1; Asia_W_Europe; 13; A263G; C315CC; A750G	16569	Asia_W_Europe	H1g1	H	H	13
1675	JQ703477.1	JQ703477.1; H1bc; Asia_W_Europe; 15; T152C; A263G; C309CCT	16572	Asia_W_Europe	H1bc	H	H	15
1676	JQ703478.1	JQ703478.1; H56; Asia_W_Europe; 10; A263G; C309CCT; T310C	16570	Asia_W_Europe	H56	H	H	10
1677	JQ703479.1	JQ703479.1; H7g; Asia_W_Europe; 11; A263G; C309CCT; T310C	16570	Asia_W_Europe	H7g	H	H	11
1678	JQ703480.1	JQ703480.1; H26a1; Asia_W_Europe; 14; T152C; A263G; C315CC	16569	Asia_W_Europe	H26a1	H	H	14
1679	JQ703481.1	JQ703481.1; L0a1a2; Africa_S_SE; 83; C64T; A93G; G185A	16567	Africa_S_SE	L0a1a2	L0	L0	83
1680	JQ703482.1	JQ703482.1; H75; Asia_W_Europe; 12; T152C; A263G; C309CCT	16570	Asia_W_Europe	H75	H	H	12
1681	JQ703483.1	JQ703483.1; H10e2; Asia_W_Europe; 15; A263G; C309CCT; T310C	16570	Asia_W_Europe	H10e2	H	H	15

Supplementary Table S4 Human mitochondrial database (hMITO DB v1.0) metadata[a]

Row	Name (accession no.)	Description	Size	Geo_Region	Haplogroup	Macro_ Haplo	Macro_ Haplo2	Total Variants
1682	JQ703484.1	JQ703484.1; H1; Asia_W_Europe; 12; A263G; C309CCT; T310C	16570	Asia_W_Europe	H1	H	H	12
1683	JQ703485.1	JQ703485.1; K1a1b1a; Asia_W; 37; A73G; A263G; C315CC	16569	Asia_W	K1a1b1a	K	K	37
1684	JQ703486.1	JQ703486.1; K1a+195; Asia_W; 34; A73G; T195C; A263G	16569	Asia_W	K1a+195	K	K	34
1685	JQ703487.1	JQ703487.1; K1c1b; Asia_W; 36; A73G; T146C; T152C	16568	Asia_W	K1c1b	K	K	36
1686	JQ703488.1	JQ703488.1; U2e1d; Asia_S_W_Europe; 41; A73G; T152C; T217C	16573	Asia_S_W_Europe	U2e1d	U	U2	41
1687	JQ703489.1	JQ703489.1; H1c; Asia_W_Europe; 11; A263G; C315CC; T477C	16570	Asia_W_Europe	H1c	H	H	11
1688	JQ703490.1	JQ703490.1; V; Europe_S; 15; T72C; A263G; C309CCT	16570	Europe_S	V	V	V	15
1689	JQ703491.1	JQ703491.1; U2e1g; Asia_S_W_Europe; 39; A73G; T152C; T217C	16571	Asia_S_W_Europe	U2e1g	U	U2	39
1690	JQ703492.1	JQ703492.1; H3g; Asia_W_Europe; 12; T152C; A263G; C315CC	16569	Asia_W_Europe	H3g	H	H	12
1691	JQ703493.1	JQ703493.1; K1a4a1; Asia_W; 40; A73G; T146C; A257G	16569	Asia_W	K1a4a1	K	K	40
1692	JQ703494.1	JQ703494.1; H5a5; Asia_W_Europe; 14; T146C; A263G; C309CCCT	16571	Asia_W_Europe	H5a5	H	H	14
1693	JQ703495.1	JQ703495.1; H7h; Asia_W_Europe; 13; T146C; A263G; C299A	16569	Asia_W_Europe	H7h	H	H	13
1694	JQ703496.1	JQ703496.1; H1c3; Asia_W_Europe; 14; A257G; A263G; C309CCT	16570	Asia_W_Europe	H1c3	H	H	14
1695	JQ703497.1	JQ703497.1; W6b; Asia_W; 44; A73G; A189G; C194T	16572	Asia_W	W6b	W	W	44
1696	JQ703498.1	JQ703498.1; H5d; Asia_W_Europe; 13; A263G; C309CCCT; T310C	16571	Asia_W_Europe	H5d	H	H	13
1697	JQ703499.1	JQ703499.1; H1c4b; Asia_W_Europe; 16; A263G; C315CC; T477C	16567	Asia_W_Europe	H1c4b	H	H	16
1698	JQ703500.1	JQ703500.1; H1b1h; Asia_W_Europe; 17; A263G; C309CCCT; T310C	16569	Asia_W_Europe	H1b1h	H	H	17
1699	JQ703501.1	JQ703501.1; T2b4; Asia_W; 34; A73G; A263G; C309CCCT	16571	Asia_W	T2b4	T	T	34
1700	JQ703502.1	JQ703502.1; H49a; Asia_W_Europe; 13; A263G; C309CCT; T310C	16573	Asia_W_Europe	H49a	H	H	13
1701	JQ703503.1	JQ703503.1; H31; Asia_W_Europe; 15; T146C; T195C; A263G	16570	Asia_W_Europe	H31	H	H	15
1702	JQ703504.1	JQ703504.1; H10d; Asia_W_Europe; 13; A263G; C309CCT; T310C	16570	Asia_W_Europe	H10d	H	H	13
1703	JQ703505.1	JQ703505.1; R0a2m; Africa_NE_Asia_W; 22; T57TC; C64T; A263G	16572	Africa_NE_Asia_W	R0a2m	R	R0	22
1704	JQ703506.1	JQ703506.1; H1au1a; Asia_W_Europe; 15; A93G; A263G; C315CC	16569	Asia_W_Europe	H1au1a	H	H	15
1705	JQ703507.1	JQ703507.1; H6a1a4; Asia_W_Europe; 17; T239C; A263G; C309CCT	16570	Asia_W_Europe	H6a1a4	H	H	17
1706	JQ703508.1	JQ703508.1; T2b4f; Asia_W; 37; A73G; T152C; A263G	16570	Asia_W	T2b4f	T	T	37
1707	JQ703509.1	JQ703509.1; H2a1a2; Asia_W_Europe; 14; A263G; C315CC; A750G	16569	Asia_W_Europe	H2a1a2	H	H	14
1708	JQ703510.1	JQ703510.1; H11a2; Asia_W_Europe; 20; T195C; A263G; C309CCT	16570	Asia_W_Europe	H11a2	H	H	20
1709	JQ703511.1	JQ703511.1; W4a1; Asia_W; 35; A73G; G143A; A189G	16569	Asia_W	W4a1	W	W	35
1710	JQ703512.1	JQ703512.1; H1; Asia_W_Europe; 12; A263G; C309CCT; T310C	16570	Asia_W_Europe	H1	H	H	12
1711	JQ703513.1	JQ703513.1; H5a1g1a; Asia_W_Europe; 19; A263G; C315CC; A444G	16567	Asia_W_Europe	H5a1g1a	H	H	19
1712	JQ703514.1	JQ703514.1; B4f; Asia_SE_E_America_N_S; 35; A73G; A200G; A263G	16563	Asia_SE_E_America_N_S	B4f	B	B	35
1713	JQ703515.1	JQ703515.1; J2b1a4; Asia_W; 35; A73G; C150T; T152C	16569	Asia_W	J2b1a4	J	J	35
1714	JQ703516.1	JQ703516.1; J1c12a; Asia_W; 34; A73G; G185A; A189G	16572	Asia_W	J1c12a	J	J	34
1715	JQ703517.1	JQ703517.1; J2b1a; Asia_W; 36; A73G; C150T; T152C	16570	Asia_W	J2b1a	J	J	36
1716	JQ703518.1	JQ703518.1; H1a1; Asia_W_Europe; 16; A73G; T195C; A263G	16570	Asia_W_Europe	H1a1	H	H	16
1717	JQ703519.1	JQ703519.1; H6c; Asia_W_Europe; 15; T239C; A263G; C309CCCT	16571	Asia_W_Europe	H6c	H	H	15
1718	JQ703520.1	JQ703520.1; H; Asia_W_Europe; 10; A263G; C309CCCT; T310C	16571	Asia_W_Europe	H	H	H	10
1719	JQ703521.1	JQ703521.1; K2a6; Asia_W; 33; A73G; T146C; T152C	16570	Asia_W	K2a6	K	K	33
1720	JQ703522.1	JQ703522.1; K1a23; Asia_W; 38; A73G; A249d; A263G	16572	Asia_W	K1a23	K	K	38
1721	JQ703523.1	JQ703523.1; K1a4c1; Asia_W; 38; A73G; T152C; A263G	16570	Asia_W	K1a4c1	K	K	38
1722	JQ703524.1	JQ703524.1; J2a1a1a2; Asia_W; 43; A73G; T146C; C150T	16570	Asia_W	J2a1a1a2	J	J	43

Supplementary Table S4 Human mitochondrial database (hMITO DB v1.0) metadata[a]

Row	Name (accession no.)	Description	Size	Geo_Region	Haplogroup	Macro_ Haplo	Macro_ Haplo2	Total Variants
1723	JQ703525.1	JQ703525.1; H1; Asia_W_Europe; 13; T146C; A153G; A263G	16572	Asia_W_Europe	H1	H	H	13
1724	JQ703526.1	JQ703526.1; K1a13; Asia_W; 38; A73G; T146C; T152C	16574	Asia_W	K1a13	K	K	38
1725	JQ703527.1	JQ703527.1; H1z; Asia_W_Europe; 14; A263G; C309CCCT; T310C	16571	Asia_W_Europe	H1z	H	H	14
1726	JQ703528.1	JQ703528.1; K1a3a; Asia_W; 35; A73G; C150T; A263G	16569	Asia_W	K1a3a	K	K	35
1727	JQ703529.1	JQ703529.1; H8c2; Asia_W_Europe; 20; C114T; T146C; T152C	16571	Asia_W_Europe	H8c2	H	H	20
1728	JQ703530.1	JQ703530.1; U2e1a1; Asia_S_W_Europe; 42; A73G; T152C; T217C	16572	Asia_S_W_Europe	U2e1a1	U	U2	42
1729	JQ703531.1	JQ703531.1; H18; Asia_W_Europe; 12; A263G; C309CCT; T310C	16570	Asia_W_Europe	H18	H	H	12
1730	JQ703532.1	JQ703532.1; H4a1a1a2; Asia_W_Europe; 26; A73G; G228T; A263G	16568	Asia_W_Europe	H4a1a1a2	H	H	26
1731	JQ703533.1	JQ703533.1; H3; Asia_W_Europe; 15; T195C; A263G; C315CC	16569	Asia_W_Europe	H3	H	H	15
1732	JQ703534.1	JQ703534.1; K2a6; Asia_W; 33; A73G; T146C; T152C	16569	Asia_W	K2a6	K	K	33
1733	JQ703535.1	JQ703535.1; H1h1; Asia_W_Europe; 14; A263G; C309CCT; T310C	16570	Asia_W_Europe	H1h1	H	H	14
1734	JQ703536.1	JQ703536.1; H1j2; Asia_W_Europe; 16; A263G; C309CCCT; T310C	16571	Asia_W_Europe	H1j2	H	H	16
1735	JQ703537.1	JQ703537.1; H1a; Asia_W_Europe; 13; A73G; T146C; A263G	16570	Asia_W_Europe	H1a	H	H	13
1736	JQ703538.1	JQ703538.1; J2b1a4; Asia_W; 35; A73G; C150T; T152C	16570	Asia_W	J2b1a4	J	J	35
1737	JQ703539.1	JQ703539.1; H10+(16093); Asia_W_Europe; 12; T204C; A263G; C309CCT	16570	Asia_W_Europe	H10+(16093)	H	H	12
1738	JQ703540.1	JQ703540.1; K2a3a; Asia_W; 35; A73G; T146C; T152C	16570	Asia_W	K2a3a	K	K	35
1739	JQ703541.1	JQ703541.1; H1u1; Asia_W_Europe; 15; T146C; A263G; C309CCT	16570	Asia_W_Europe	H1u1	H	H	15
1740	JQ703542.1	JQ703542.1; H4a1a2a; Asia_W_Europe; 18; A263G; C315CC; CA522d	16567	Asia_W_Europe	H4a1a2a	H	H	18
1741	JQ703543.1	JQ703543.1; H11b1; Asia_W_Europe; 18; T146C; T195C; A263G	16570	Asia_W_Europe	H11b1	H	H	18
1742	JQ703544.1	JQ703544.1; H3w; Asia_W_Europe; 15; A263G; C309CCT; T310C	16570	Asia_W_Europe	H3w	H	H	15
1743	JQ703545.1	JQ703545.1; J1b1b1; Asia_W; 34; A73G; A263G; C295T	16568	Asia_W	J1b1b1	J	J	34
1744	JQ703546.1	JQ703546.1; H; Asia_W_Europe; 11; A263G; C315CC; CA522d	16567	Asia_W_Europe	H	H	H	11
1745	JQ703547.1	JQ703547.1; H5s; Asia_W_Europe; 13; A263G; C309CCT; T310C	16570	Asia_W_Europe	H5s	H	H	13
1746	JQ703548.1	JQ703548.1; K1a4a1f; Asia_W; 38; A73G; T152C; A263G	16569	Asia_W	K1a4a1f	K	K	38
1747	JQ703549.1	JQ703549.1; H3+152; Asia_W_Europe; 14; T152C; A263G; C309CCT	16572	Asia_W_Europe	H3+152	H	H	14
1748	JQ703550.1	JQ703550.1; H5+709; Asia_W_Europe; 14; A263G; C309CCCT; T310C	16571	Asia_W_Europe	H5+709	H	H	14
1749	JQ703551.1	JQ703551.1; H1c15; Asia_W_Europe; 13; A263G; C309CCT; T310C	16573	Asia_W_Europe	H1c15	H	H	13
1750	JQ703552.1	JQ703552.1; J2a1a1c; Asia_W; 40; A73G; C150T; T152C	16570	Asia_W	J2a1a1c	J	J	40
1751	JQ703553.1	JQ703553.1; K2a10; Asia_W; 33; A73G; T146C; T152C	16570	Asia_W	K2a10	K	K	33
1752	JQ703554.1	JQ703554.1; H2a1a1; Asia_W_Europe; 14; A263G; C315CC; A750G	16569	Asia_W_Europe	H2a1a1	H	H	14
1753	JQ703555.1	JQ703555.1; H3c1; Asia_W_Europe; 14; A263G; C309CCCT; T310C	16571	Asia_W_Europe	H3c1	H	H	14
1754	JQ703556.1	JQ703556.1; J1c2; Asia_W; 30; A73G; G185A; A188G	16571	Asia_W	J1c2	J	J	30
1755	JQ703557.1	JQ703557.1; J1c1; Asia_W; 28; A73G; G185A; A263G	16569	Asia_W	J1c1	J	J	28
1756	JQ703558.1	JQ703558.1; J1c5a1; Asia_W; 34; A73G; G185A; G228A	16570	Asia_W	J1c5a1	J	J	34
1757	JQ703559.1	JQ703559.1; H6c1; Asia_W_Europe; 15; T239C; A263G; C309CCT	16570	Asia_W_Europe	H6c1	H	H	15
1758	JQ703560.1	JQ703560.1; X2b4; Asia_W_America_N; 32; A73G; A153G; T195C	16569	Asia_W_America_N	X2b4	X	X	32
1759	JQ703561.1	JQ703561.1; H1as1; Asia_W_Europe; 14; C150T; A263G; C315CC	16569	Asia_W_Europe	H1as1	H	H	14
1760	JQ703562.1	JQ703562.1; J1c7a; Asia_W; 33; A73G; T146C; G185A	16569	Asia_W	J1c7a	J	J	33
1761	JQ703563.1	JQ703563.1; H1a; Asia_W_Europe; 12; A73G; A263G; C315CC	16569	Asia_W_Europe	H1a	H	H	12
1762	JQ703564.1	JQ703564.1; H45a; Asia_W_Europe; 12; A263G; C309CCT; T310C	16570	Asia_W_Europe	H45a	H	H	12
1763	JQ703565.1	JQ703565.1; J1c3c; Asia_W; 28; A73G; G185A; A263G	16569	Asia_W	J1c3c	J	J	28

Supplementary Table S4 Human mitochondrial database (hMITO DB v1.0) metadata[a]

Row	Name (accession no.)	Description	Size	Geo_Region	Haplogroup	Macro_ Haplo	Macro_ Haplo2	Total Variants
1764	JQ703566.1	JQ703566.1; J2a1a1a2; Asia_W; 41; A73G; C150T; T152C	16570	Asia_W	J2a1a1a2	J	J	41
1765	JQ703567.1	JQ703567.1; J1c2b4; Asia_W; 33; A73G; G185A; A188G	16570	Asia_W	J1c2b4	J	J	33
1766	JQ703568.1	JQ703568.1; J2a1a1a3; Asia_W; 42; A73G; C150T; T152C	16570	Asia_W	J2a1a1a3	J	J	42
1767	JQ703569.1	JQ703569.1; J1b1a1; Asia_W; 36; A73G; C242T; A263G	16569	Asia_W	J1b1a1	J	J	36
1768	JQ703570.1	JQ703570.1; J2a1a1a2; Asia_W; 42; A73G; C150T; T152C	16571	Asia_W	J2a1a1a2	J	J	42
1769	JQ703571.1	JQ703571.1; H1bs; Asia_W_Europe; 16; A263G; C309CCCT; T310C	16571	Asia_W_Europe	H1bs	H	H	16
1770	JQ703572.1	JQ703572.1; J1c5; Asia_W; 29; A73G; G185A; G228A	16571	Asia_W	J1c5	J	J	29
1771	JQ703573.1	JQ703573.1; H3g1; Asia_W_Europe; 15; T152C; A263G; C315CC	16569	Asia_W_Europe	H3g1	H	H	15
1772	JQ703574.1	JQ703574.1; J1b1a1a; Asia_W; 39; A73G; C242T; A263G	16569	Asia_W	J1b1a1a	J	J	39
1773	JQ703575.1	JQ703575.1; J1c2o; Asia_W; 34; A73G; G185A; A188G	16569	Asia_W	J1c2o	J	J	34
1774	JQ703576.1	JQ703576.1; H1e1a6; Asia_W_Europe; 16; C150T; A263G; C309CCCT	16571	Asia_W_Europe	H1e1a6	H	H	16
1775	JQ703577.1	JQ703577.1; H5a1; Asia_W_Europe; 13; A263G; C315CC; C456T	16567	Asia_W_Europe	H5a1	H	H	13
1776	JQ703578.1	JQ703578.1; H20a1a; Asia_W_Europe; 15; A249d; A263G; T292C	16569	Asia_W_Europe	H20a1a	H	H	15
1777	JQ703579.1	JQ703579.1; H1ba; Asia_W_Europe; 12; A263G; C315CC; A750G	16569	Asia_W_Europe	H1ba	H	H	12
1778	JQ703580.1	JQ703580.1; J2b1a1; Asia_W; 34; A73G; C150T; T152C	16570	Asia_W	J2b1a1	J	J	34
1779	JQ703581.1	JQ703581.1; K1a2a2; Asia_W; 33; A73G; A263G; C315CC	16569	Asia_W	K1a2a2	K	K	33
1780	JQ703582.1	JQ703582.1; H2a1c; Asia_W_Europe; 10; A263G; C309CCT; T310C	16570	Asia_W_Europe	H2a1c	H	H	10
1781	JQ703583.1	JQ703583.1; J1c7a; Asia_W; 35; A73G; T146C; G185A	16569	Asia_W	J1c7a	J	J	35
1782	JQ703584.1	JQ703584.1; J1c13; Asia_W; 33; A73G; G185A; A189G	16569	Asia_W	J1c13	J	J	33
1783	JQ703585.1	JQ703585.1; J1b1a1+146; Asia_W; 38; A73G; T146C; C242T	16570	Asia_W	J1b1a1+146	J	J	38
1784	JQ703586.1	JQ703586.1; J1c3b1a; Asia_W; 33; A73G; G185A; A263G	16570	Asia_W	J1c3b1a	J	J	33
1785	JQ703587.1	JQ703587.1; J2b1a2; Asia_W; 35; A73G; C150T; T152C	16570	Asia_W	J2b1a2	J	J	35
1786	JQ703588.1	JQ703588.1; J1b1a1a; Asia_W; 40; A73G; C242T; A263G	16570	Asia_W	J1b1a1a	J	J	40
1787	JQ703589.1	JQ703589.1; U2e1a1; Asia_S_W_Europe; 41; A73G; T152C; T217C	16573	Asia_S_W_Europe	U2e1a1	U	U2	41
1788	JQ703590.1	JQ703590.1; U5a1a1c; Asia_W_Europe_C; 29; A73G; A263G; C309CCT	16570	Asia_W_Europe_C	U5a1a1c	U	U5	29
1789	JQ703591.1	JQ703591.1; J1c2; Asia_W; 33; A73G; G185A; A188G	16568	Asia_W	J1c2	J	J	33
1790	JQ703592.1	JQ703592.1; H3h1; Asia_W_Europe; 15; T152C; A263G; C309CCT	16570	Asia_W_Europe	H3h1	H	H	15
1791	JQ703593.1	JQ703593.1; H11a; Asia_W_Europe; 16; T146C; T195C; A263G	16569	Asia_W_Europe	H11a	H	H	16
1792	JQ703594.1	JQ703594.1; J1c1b2; Asia_W; 32; A73G; A263G; C295T	16567	Asia_W	J1c1b2	J	J	32
1793	JQ703595.1	JQ703595.1; J1b1a3; Asia_W; 38; A73G; C242T; A263G	16570	Asia_W	J1b1a3	J	J	38
1794	JQ703596.1	JQ703596.1; H33b; Asia_W_Europe; 12; A263G; C309CCT; T310C	16570	Asia_W_Europe	H33b	H	H	12
1795	JQ703597.1	JQ703597.1; J1c3; Asia_W; 32; A73G; T146C; T152C	16569	Asia_W	J1c3	J	J	32
1796	JQ703598.1	JQ703598.1; H3ae; Asia_W_Europe; 13; A263G; C309CCT; T310C	16570	Asia_W_Europe	H3ae	H	H	13
1797	JQ703599.1	JQ703599.1; J1c1b1a1; Asia_W; 36; A73G; G185A; G228A	16570	Asia_W	J1c1b1a1	J	J	36
1798	JQ703600.1	JQ703600.1; U5b1b1a1b; Asia_W_Europe_C; 31; A73G; C150T; A263G	16569	Asia_W_Europe_C	U5b1b1a1b	U	U5	31
1799	JQ703601.1	JQ703601.1; H7c2; Asia_W_Europe; 12; A263G; C309CCCT; T310C	16571	Asia_W_Europe	H7c2	H	H	12
1800	JQ703602.1	JQ703602.1; J1c3c2; Asia_W; 31; A73G; G185A; G228A	16570	Asia_W	J1c3c2	J	J	31
1801	JQ703603.1	JQ703603.1; H1e1c; Asia_W_Europe; 17; T152C; A263G; C309CCT	16570	Asia_W_Europe	H1e1c	H	H	17
1802	JQ703604.1	JQ703604.1; J1c5b; Asia_W; 31; A73G; G185A; G228A	16569	Asia_W	J1c5b	J	J	31
1803	JQ703605.1	JQ703605.1; J2a2b2; Asia_W; 38; A73G; C150T; T195C	16569	Asia_W	J2a2b2	J	J	38
1804	JQ703606.1	JQ703606.1; H; Asia_W_Europe; 11; A263G; C309CCT; T310C	16570	Asia_W_Europe	H	H	H	11

Supplementary Table S4 Human mitochondrial database (hMITO DB v1.0) metadata[a]

Row	Name (accession no.)	Description	Size	Geo_Region	Haplogroup	Macro_ Haplo	Macro_ Haplo2	Total Variants
1805	JQ703607.1	JQ703607.1; K1a11a; Asia_W; 40; A16T; A73G; C150T	16561	Asia_W	K1a11a	K	K	40
1806	JQ703608.1	JQ703608.1; H27a; Asia_W_Europe; 15; A263G; C309CCCT; T310C	16571	Asia_W_Europe	H27a	H	H	15
1807	JQ703609.1	JQ703609.1; J1c1b1a; Asia_W; 34; A73G; G185A; G228A	16566	Asia_W	J1c1b1a	J	J	34
1808	JQ703610.1	JQ703610.1; J1c2b2; Asia_W; 33; A73G; G185A; A188G	16570	Asia_W	J1c2b2	J	J	33
1809	JQ703611.1	JQ703611.1; H1+16189; Asia_W_Europe; 15; T146C; A263G; C309CCCT	16571	Asia_W_Europe	H1+16189	H	H	15
1810	JQ703612.1	JQ703612.1; J1c2; Asia_W; 32; A73G; G185A; A188G	16570	Asia_W	J1c2	J	J	32
1811	JQ703613.1	JQ703613.1; J1c2b1; Asia_W; 34; A73G; G185A; A188G	16570	Asia_W	J1c2b1	J	J	34
1812	JQ703614.1	JQ703614.1; U5a1a1; Asia_W_Europe_C; 28; A73G; A263G; C309CCT	16570	Asia_W_Europe_C	U5a1a1	U	U5	28
1813	JQ703615.1	JQ703615.1; HV5a; Asia_W; 12; A263G; C315CC; A750G	16569	Asia_W	HV5a	HV	HV	12
1814	JQ703616.1	JQ703616.1; H1a1b; Asia_W_Europe; 15; A73G; A263G; C315CC	16569	Asia_W_Europe	H1a1b	H	H	15
1815	JQ703617.1	JQ703617.1; H1c; Asia_W_Europe; 11; A263G; C315CC; T477C	16569	Asia_W_Europe	H1c	H	H	11
1816	JQ703618.1	JQ703618.1; L1b1a7a; Africa_C; 81; A73G; T152C; C182T	16567	Africa_C	L1b1a7a	L1	L1	81
1817	JQ703619.1	JQ703619.1; H3; Asia_W_Europe; 12; A263G; C309CCCT; T310C	16571	Asia_W_Europe	H3	H	H	12
1818	JQ703620.1	JQ703620.1; H5a1e; Asia_W_Europe; 14; A263G; C315CC; C456T	16567	Asia_W_Europe	H5a1e	H	H	14
1819	JQ703621.1	JQ703621.1; L3f1b; Africa_E; 38; A73G; A189G; A200G	16566	Africa_E	L3f1b	L3	L3	38
1820	JQ703622.1	JQ703622.1; H6a1b2; Asia_W_Europe; 17; T239C; A263G; C309CCT	16570	Asia_W_Europe	H6a1b2	H	H	17
1821	JQ703623.1	JQ703623.1; J1c1; Asia_W; 30; A73G; G185A; A263G	16569	Asia_W	J1c1	J	J	30
1822	JQ703624.1	JQ703624.1; U5a1b1a; Asia_W_Europe_C; 31; A73G; A263G; A272G	16569	Asia_W_Europe_C	U5a1b1a	U	U5	31
1823	JQ703625.1	JQ703625.1; L3e3b; Africa_E; 40; A73G; C150T; T195C	16567	Africa_E	L3e3b	L3	L3	40
1824	JQ703626.1	JQ703626.1; U5a2a1a; Asia_W_Europe_C; 30; A73G; A263G; C309CCT	16570	Asia_W_Europe_C	U5a2a1a	U	U5	30
1825	JQ703627.1	JQ703627.1; H5c2; Asia_W_Europe; 13; A263G; C315CC; C456T	16569	Asia_W_Europe	H5c2	H	H	13
1826	JQ703628.1	JQ703628.1; U5b2a2b1; Asia_W_Europe_C; 34; A73G; C150T; A263G	16570	Asia_W_Europe_C	U5b2a2b1	U	U5	34
1827	JQ703629.1	JQ703629.1; K2a2a1; Asia_W; 38; A73G; T146C; T152C	16569	Asia_W	K2a2a1	K	K	38
1828	JQ703630.1	JQ703630.1; H4a1c1; Asia_W_Europe; 20; A263G; C309CCT; T310C	16568	Asia_W_Europe	H4a1c1	H	H	20
1829	JQ703631.1	JQ703631.1; H1j7; Asia_W_Europe; 15; A263G; C309CCCT; T310C	16571	Asia_W_Europe	H1j7	H	H	15
1830	JQ703632.1	JQ703632.1; H5q; Asia_W_Europe; 12; C150T; A263G; C315CC	16569	Asia_W_Europe	H5q	H	H	12
1831	JQ703633.1	JQ703633.1; R1b1; Asia_S_SE; 32; A73G; T146C; C150T	16573	Asia_S_SE	R1b1	R	R1	32
1832	JQ703634.1	JQ703634.1; H1b1+16362; Asia_W_Europe; 16; A263G; C315CC; CA522d	16567	Asia_W_Europe	H1b1+16362	H	H	16
1833	JQ703635.1	JQ703635.1; H1bu; Asia_W_Europe; 11; A263G; C315CC; A750G	16569	Asia_W_Europe	H1bu	H	H	11
1834	JQ703636.1	JQ703636.1; H24a; Asia_W_Europe; 12; T146C; A263G; C309CCT	16570	Asia_W_Europe	H24a	H	H	12
1835	JQ703637.1	JQ703637.1; H1c9a; Asia_W_Europe; 14; T152C; A263G; C309CCCT	16571	Asia_W_Europe	H1c9a	H	H	14
1836	JQ703638.1	JQ703638.1; J2a1a1a2; Asia_W; 42; A73G; C150T; T152C	16570	Asia_W	J2a1a1a2	J	J	42
1837	JQ703639.1	JQ703639.1; J1c5b; Asia_W; 33; A73G; G185A; G228A	16569	Asia_W	J1c5b	J	J	33
1838	JQ703640.1	JQ703640.1; V11; Europe_S; 18; T72C; A263G; C309CCCT	16571	Europe_S	V11	V	V	18
1839	JQ703641.1	JQ703641.1; B4a(B4a1c3a); Asia_SE_E_America_N_S; 3; C309CCT; T310C; G709	16570	Asia_SE_E_America_N_S	B4a(B4a1c3a)	B	B	3
1840	JQ703642.1	JQ703642.1; U5a2a1e; Asia_W_Europe_C; 29; A73G; T152C; A263G	16569	Asia_W_Europe_C	U5a2a1e	U	U5	29
1841	JQ703643.1	JQ703643.1; H5a1c1a; Asia_W_Europe; 18; A263G; C309CCT; T310C	16568	Asia_W_Europe	H5a1c1a	H	H	18
1842	JQ703644.1	JQ703644.1; H5; Asia_W_Europe; 13; A263G; C309CCT; T310C	16570	Asia_W_Europe	H5	H	H	13
1843	JQ703645.1	JQ703645.1; H10e1a; Asia_W_Europe; 14; A263G; C309CCT; T310C	16570	Asia_W_Europe	H10e1a	H	H	14
1844	JQ703646.1	JQ703646.1; H1c3; Asia_W_Europe; 14; T195C; A257G; A263G	16571	Asia_W_Europe	H1c3	H	H	14
1845	JQ703647.1	JQ703647.1; V2; Europe_S; 20; T72C; A263G; C309CCCT	16571	Europe_S	V2	V	V	20

Supplementary Table S4 Human mitochondrial database (hMITO DB v1.0) metadata[a]

Row	Name (accession no.)	Description	Size	Geo_Region	Haplogroup	Macro_ Haplo	Macro_ Haplo2	Total Variants
1846	JQ703648.1	JQ703648.1; K1a3a1; Asia_W; 35; A73G; A263G; C309CCT	16572	Asia_W	K1a3a1	K	K	35
1847	JQ703649.1	JQ703649.1; H4a1a1; Asia_W_Europe; 17; A263G; C315CC; A750G	16569	Asia_W_Europe	H4a1a1	H	H	17
1848	JQ703650.1	JQ703650.1; H13a1a1a; Asia_W_Europe; 19; T152C; A263G; C309CCT	16570	Asia_W_Europe	H13a1a1a	H	H	19
1849	JQ703651.1	JQ703651.1; K1a9; Asia_W; 33; A73G; T195C; A263G	16569	Asia_W	K1a9	K	K	33
1850	JQ703652.1	JQ703652.1; I1a1a; Asia_W_SW; 44; A73G; T199C; G203A	16573	Asia_W_SW	I1a1a	I	I	44
1851	JQ703653.1	JQ703653.1; J1c1c; Asia_W; 33; A73G; C150T; G185A	16567	Asia_W	J1c1c	J	J	33
1852	JQ703654.1	JQ703654.1; H10b; Asia_W_Europe; 12; A263G; C309CCT; T310C	16570	Asia_W_Europe	H10b	H	H	12
1853	JQ703655.1	JQ703655.1; H1aj1a; Asia_W_Europe; 16; G207A; A263G; C315CC	16569	Asia_W_Europe	H1aj1a	H	H	16
1854	JQ703656.1	JQ703656.1; J1b1b2; Asia_W; 38; A73G; T152C; A263G	16570	Asia_W	J1b1b2	J	J	38
1855	JQ703657.1	JQ703657.1; H13c1; Asia_W_Europe; 18; A263G; C309CCT; T310C	16570	Asia_W_Europe	H13c1	H	H	18
1856	JQ703658.1	JQ703658.1; H102; Asia_W_Europe; 13; A263G; C309CCT; T310C	16570	Asia_W_Europe	H102	H	H	13
1857	JQ703659.1	JQ703659.1; HV9a1a; Asia_W; 20; T131C; T152C; T195C	16571	Asia_W	HV9a1a	HV	HV	20
1858	JQ703660.1	JQ703660.1; H27+16093; Asia_W_Europe; 17; A263G; C315CC; A750G	16569	Asia_W_Europe	H27+16093	H	H	17
1859	JQ703661.1	JQ703661.1; T2e; Asia_W; 35; A73G; C150T; A263G	16571	Asia_W	T2e	T	T	35
1860	JQ703662.1	JQ703662.1; K1a1b1a; Asia_W; 36; A73G; C114T; A263G	16569	Asia_W	K1a1b1a	K	K	36
1861	JQ703663.1	JQ703663.1; T2f1a1; Asia_W; 42; A73G; T195C; A263G	16560	Asia_W	T2f1a1	T	T	42
1862	JQ703664.1	JQ703664.1; H1+152; Asia_W_Europe; 12; T152C; A263G; C315CC	16569	Asia_W_Europe	H1+152	H	H	12
1863	JQ703665.1	JQ703665.1; K1b2b; Asia_W; 39; A73G; T146C; T152C	16570	Asia_W	K1b2b	K	K	39
1864	JQ703666.1	JQ703666.1; V3; Europe_S; 16; T72C; A263G; C309CCCT	16571	Europe_S	V3	V	V	16
1865	JQ703667.1	JQ703667.1; V3; Europe_S; 17; T72C; A263G; C309CCCT	16571	Europe_S	V3	V	V	17
1866	JQ703668.1	JQ703668.1; H1bh; Asia_W_Europe; 13; A263G; C315CC; A750G	16569	Asia_W_Europe	H1bh	H	H	13
1867	JQ703669.1	JQ703669.1; H1bb; Asia_W_Europe; 15; T152C; T199C; A263G	16571	Asia_W_Europe	H1bb	H	H	15
1868	JQ703670.1	JQ703670.1; T1a1; Asia_W; 37; A73G; T152C; T195C	16570	Asia_W	T1a1	T	T	37
1869	JQ703671.1	JQ703671.1; J1c3b1; Asia_W; 31; A73G; G185A; A263G	16570	Asia_W	J1c3b1	J	J	31
1870	JQ703672.1	JQ703672.1; J1c2b4; Asia_W; 33; A73G; G185A; A188G	16569	Asia_W	J1c2b4	J	J	33
1871	JQ703673.1	JQ703673.1; J2a1a1a2; Asia_W; 41; A73G; C150T; T152C	16570	Asia_W	J2a1a1a2	J	J	41
1872	JQ703674.1	JQ703674.1; H10e1a; Asia_W_Europe; 15; A263G; C309CCT; T310C	16570	Asia_W_Europe	H10e1a	H	H	15
1873	JQ703675.1	JQ703675.1; J1c2e; Asia_W; 33; A73G; G185A; A188G	16568	Asia_W	J1c2e	J	J	33
1874	JQ703676.1	JQ703676.1; H11a; Asia_W_Europe; 15; T195C; A263G; C309CCCT	16571	Asia_W_Europe	H11a	H	H	15
1875	JQ703677.1	JQ703677.1; H3h1; Asia_W_Europe; 13; A263G; C315CC; A750G	16569	Asia_W_Europe	H3h1	H	H	13
1876	JQ703678.1	JQ703678.1; H11a1; Asia_W_Europe; 17; T146C; T195C; A263G	16569	Asia_W_Europe	H11a1	H	H	17
1877	JQ703679.1	JQ703679.1; K1c2; Asia_W; 36; A73G; T146C; T152C	16568	Asia_W	K1c2	K	K	36
1878	JQ703680.1	JQ703680.1; T2b; Asia_W; 34; A73G; A263G; C315CC	16569	Asia_W	T2b	T	T	34
1879	JQ703681.1	JQ703681.1; H1be; Asia_W_Europe; 12; A263G; C309CCCT; T310C	16571	Asia_W_Europe	H1be	H	H	12
1880	JQ703682.1	JQ703682.1; J1c7; Asia_W; 31; A73G; G185A; G228A	16569	Asia_W	J1c7	J	J	31
1881	JQ703683.1	JQ703683.1; H1a3b1; Asia_W_Europe; 17; A73G; A263G; C315CC	16567	Asia_W_Europe	H1a3b1	H	H	17
1882	JQ703684.1	JQ703684.1; H11; Asia_W_Europe; 14; T195C; A263G; C315CC	16569	Asia_W_Europe	H11	H	H	14
1883	JQ703685.1	JQ703685.1; K1a4a1a2a; Asia_W; 40; A73G; A263G; C309CCT	16576	Asia_W	K1a4a1a2a	K	K	40
1884	JQ703686.1	JQ703686.1; J1c9; Asia_W; 30; A73G; G185A; G228A	16571	Asia_W	J1c9	J	J	30
1885	JQ703687.1	JQ703687.1; H1ah2; Asia_W_Europe; 14; A263G; C309CCT; T310C	16570	Asia_W_Europe	H1ah2	H	H	14
1886	JQ703688.1	JQ703688.1; H11a1; Asia_W_Europe; 16; T146C; T195C; A263G	16569	Asia_W_Europe	H11a1	H	H	16

Supplementary Table S4 Human mitochondrial database (hMITO DB v1.0) metadata[a]

Row	Name (accession no.)	Description	Size	Geo_Region	Haplogroup	Macro_ Haplo	Macro_ Haplo2	Total Variants
1887	JQ703689.1	JQ703689.1; H3b7; Asia_W_Europe; 14; A93G; A263G; C309CCCT	16571	Asia_W_Europe	H3b7	H	H	14
1888	JQ703690.1	JQ703690.1; H3z1; Asia_W_Europe; 14; A263G; T293C; C315CC	16569	Asia_W_Europe	H3z1	H	H	14
1889	JQ703691.1	JQ703691.1; T2c1a; Asia_W; 40; A73G; T152C; G185A	16572	Asia_W	T2c1a	T	T	40
1890	JQ703692.1	JQ703692.1; T2a1b1a1a2; Asia_W; 45; A73G; T146C; T152C	16570	Asia_W	T2a1b1a1a2	T	T	45
1891	JQ703693.1	JQ703693.1; T1a; Asia_W; 36; A73G; A263G; C309CCT	16570	Asia_W	T1a	T	T	36
1892	JQ703694.1	JQ703694.1; U3b3; Africa_NE_Asia_W; 30; A73G; C150T; T152C	16570	Africa_NE_Asia_W	U3b3	U	U3	30
1893	JQ703695.1	JQ703695.1; K1c1; Asia_W; 34; A73G; T146C; T152C	16568	Asia_W	K1c1	K	K	34
1894	JQ703696.1	JQ703696.1; K1c1c; Asia_W; 35; A73G; T146C; T152C	16568	Asia_W	K1c1c	K	K	35
1895	JQ703697.1	JQ703697.1; T2f8a; Asia_W; 35; A73G; A263G; C309CCT	16561	Asia_W	T2f8a	T	T	35
1896	JQ703698.1	JQ703698.1; H1e2; Asia_W_Europe; 12; A263G; C315CC; A750G	16569	Asia_W_Europe	H1e2	H	H	12
1897	JQ703699.1	JQ703699.1; T2b3+151; Asia_W; 42; A73G; C151T; A263G	16570	Asia_W	T2b3+151	T	T	42
1898	JQ703700.1	JQ703700.1; T2a1b1a1a1; Asia_W; 40; A73G; A263G; C315CC	16569	Asia_W	T2a1b1a1a1	T	T	40
1899	JQ703701.1	JQ703701.1; H44a; Asia_W_Europe; 12; A263G; C315CC; A750G	16569	Asia_W_Europe	H44a	H	H	12
1900	JQ703702.1	JQ703702.1; T2f2; Asia_W; 37; A73G; T195C; A263G	16562	Asia_W	T2f2	T	T	37
1901	JQ703703.1	JQ703703.1; X2c1c1; Asia_W_America_N; 31; A73G; A153G; T195C	16569	Asia_W_America_N	X2c1c1	X	X	31
1902	JQ703704.1	JQ703704.1; K1a4a1; Asia_W; 34; A73G; A263G; C315CC	16569	Asia_W	K1a4a1	K	K	34
1903	JQ703705.1	JQ703705.1; H1+16189; Asia_W_Europe; 11; A263G; C315CC; A750G	16569	Asia_W_Europe	H1+16189	H	H	11
1904	JQ703706.1	JQ703706.1; H1am; Asia_W_Europe; 11; A263G; C309CCT; T310C	16570	Asia_W_Europe	H1am	H	H	11
1905	JQ703707.1	JQ703707.1; M6a1b; Asia_S; 48; A73G; T146C; T152C	16571	Asia_S	M6a1b	M	M6	48
1906	JQ703708.1	JQ703708.1; T1a1; Asia_W; 40; A73G; T152C; T195C	16570	Asia_W	T1a1	T	T	40
1907	JQ703709.1	JQ703709.1; H1i2a; Asia_W_Europe; 15; T152C; A263G; C315CC	16569	Asia_W_Europe	H1i2a	H	H	15
1908	JQ703710.1	JQ703710.1; T2b4; Asia_W; 34; A73G; A263G; C315CC	16569	Asia_W	T2b4	T	T	34
1909	JQ703711.1	JQ703711.1; K1a12a; Asia_W; 36; A73G; A263G; C309CCT	16572	Asia_W	K1a12a	K	K	36
1910	JQ703712.1	JQ703712.1; T2b5a1; Asia_W; 37; A73G; A263G; C315CC	16570	Asia_W	T2b5a1	T	T	37
1911	JQ703713.1	JQ703713.1; T2b11; Asia_W; 37; A73G; G207A; A263G	16570	Asia_W	T2b11	T	T	37
1912	JQ703714.1	JQ703714.1; T2b7a1; Asia_W; 40; A73G; T152C; A263G	16568	Asia_W	T2b7a1	T	T	40
1913	JQ703715.1	JQ703715.1; T2b31; Asia_W; 36; A73G; A263G; C309CCT	16570	Asia_W	T2b31	T	T	36
1914	JQ703716.1	JQ703716.1; T2b5a; Asia_W; 36; A73G; A263G; C309CCT	16570	Asia_W	T2b5a	T	T	36
1915	JQ703717.1	JQ703717.1; U5a1a2a; Asia_W_Europe_C; 36; A73G; A263G; C309CCCT	16573	Asia_W_Europe_C	U5a1a2a	U	U5	36
1916	JQ703718.1	JQ703718.1; J1c2c1a; Asia_W; 36; A73G; T146C; G185A	16569	Asia_W	J1c2c1a	J	J	36
1917	JQ703719.1	JQ703719.1; H1c3; Asia_W_Europe; 16; T195C; A257G; A263G	16571	Asia_W_Europe	H1c3	H	H	16
1918	JQ703720.1	JQ703720.1; HV9+152; Asia_W; 16; T152C; A263G; C309CCT	16570	Asia_W	HV9+152	HV	HV	16
1919	JQ703721.1	JQ703721.1; H10e; Asia_W_Europe; 12; A263G; C309CCT; T310C	16570	Asia_W_Europe	H10e	H	H	12
1920	JQ703722.1	JQ703722.1; T2b; Asia_W; 36; A73G; A263G; C309CCT	16570	Asia_W	T2b	T	T	36
1921	JQ703723.1	JQ703723.1; H4a1a4b; Asia_W_Europe; 20; T195C; A263G; C315CC	16567	Asia_W_Europe	H4a1a4b	H	H	20
1922	JQ703724.1	JQ703724.1; T1a5; Asia_W; 33; A73G; A263G; C315CC	16569	Asia_W	T1a5	T	T	33
1923	JQ703725.1	JQ703725.1; H24a; Asia_W_Europe; 12; A263G; C315CC; A750G	16569	Asia_W_Europe	H24a	H	H	12
1924	JQ703726.1	JQ703726.1; K2a; Asia_W; 32; A73G; T146C; T152C	16569	Asia_W	K2a	K	K	32
1925	JQ703727.1	JQ703727.1; E1a1a1b1; Asia_SE_Oceania; 41; A73G; A263G; C309CCT	16570	Asia_SE_Oceania	E1a1a1b1	E	E	41
1926	JQ703728.1	JQ703728.1; T2b32; Asia_W; 39; A73G; A263G; C315CC	16569	Asia_W	T2b32	T	T	39
1927	JQ703729.1	JQ703729.1; H1y; Asia_W_Europe; 13; A263G; C315CC; C569A	16569	Asia_W_Europe	H1y	H	H	13

Supplementary Table S4 Human mitochondrial database (hMITO DB v1.0) metadata[a]

Row	Name (accession no.)	Description	Size	Geo_Region	Haplogroup	Macro_ Haplo	Macro_ Haplo2	Total Variants
1928	JQ703730.1	JQ703730.1; T2a1b1a; Asia_W; 40; A73G; A153G; T199C	16567	Asia_W	T2a1b1a	T	T	40
1929	JQ703731.1	JQ703731.1; H5a4; Asia_W_Europe; 17; A263G; C309CCCT; T310C	16571	Asia_W_Europe	H5a4	H	H	17
1930	JQ703732.1	JQ703732.1; H6a1b2; Asia_W_Europe; 18; T239C; A263G; C309CCCT	16571	Asia_W_Europe	H6a1b2	H	H	18
1931	JQ703733.1	JQ703733.1; H11a7; Asia_W_Europe; 18; T152C; T195C; A263G	16569	Asia_W_Europe	H11a7	H	H	18
1932	JQ703734.1	JQ703734.1; T2b4d; Asia_W; 38; A73G; T152C; A263G	16570	Asia_W	T2b4d	T	T	38
1933	JQ703735.1	JQ703735.1; H1a3; Asia_W_Europe; 14; A73G; A263G; C309CCT	16570	Asia_W_Europe	H1a3	H	H	14
1934	JQ703736.1	JQ703736.1; D4b1c; Asia_E_America_N_S; 43; A73G; T239C; A263G	16567	Asia_E_America_N_S	D4b1c	D	D	43
1935	JQ703737.1	JQ703737.1; K1a1b1f; Asia_W; 40; A73G; C114T; A263G	16570	Asia_W	K1a1b1f	K	K	40
1936	JQ703738.1	JQ703738.1; H5a1f; Asia_W_Europe; 13; A263G; C456T; CA522d	16566	Asia_W_Europe	H5a1f	H	H	13
1937	JQ703739.1	JQ703739.1; T1a1; Asia_W; 38; A73G; T152C; T195C	16570	Asia_W	T1a1	T	T	38
1938	JQ703740.1	JQ703740.1; J1c5e; Asia_W; 31; A73G; G185A; A263G	16569	Asia_W	J1c5e	J	J	31
1939	JQ703741.1	JQ703741.1; U5b2b1a1; Asia_W_Europe_C; 35; A73G; T146C; C150T	16570	Asia_W_Europe_C	U5b2b1a1	U	U5	35
1940	JQ703742.1	JQ703742.1; H1q; Asia_W_Europe; 13; A263G; C315CC; A750G	16569	Asia_W_Europe	H1q	H	H	13
1941	JQ703743.1	JQ703743.1; C1c4; Asia_NE_America_N_S; 49; A73G; T152C; A214G	16569	Asia_NE_America_N_S	C1c4	C	C	49
1942	JQ703744.1	JQ703744.1; H3c; Asia_W_Europe; 15; A263G; C315CC; C573CCC	16572	Asia_W_Europe	H3c	H	H	15
1943	JQ703745.1	JQ703745.1; H1c5a; Asia_W_Europe; 14; T152C; A249d; A263G	16568	Asia_W_Europe	H1c5a	H	H	14
1944	JQ703746.1	JQ703746.1; J1c2e; Asia_W; 32; A73G; G185A; A188G	16568	Asia_W	J1c2e	J	J	32
1945	JQ703747.1	JQ703747.1; H66a1; Asia_W_Europe; 13; A73G; A263G; C309CCT	16570	Asia_W_Europe	H66a1	H	H	13
1946	JQ703748.1	JQ703748.1; U5a1b1h; Asia_W_Europe_C; 29; A73G; A263G; C315CC	16567	Asia_W_Europe_C	U5a1b1h	U	U5	29
1947	JQ703749.1	JQ703749.1; V19; Europe_S; 17; T72C; C150T; A263G	16569	Europe_S	V19	V	V	17
1948	JQ703750.1	JQ703750.1; J1c5; Asia_W; 32; A73G; G185A; C198T	16567	Asia_W	J1c5	J	J	32
1949	JQ703751.1	JQ703751.1; H1bb; Asia_W_Europe; 14; T152C; A263G; C315CC	16573	Asia_W_Europe	H1bb	H	H	14
1950	JQ703752.1	JQ703752.1; K2a; Asia_W; 33; A73G; T146C; T152C	16569	Asia_W	K2a	K	K	33
1951	JQ703753.1	JQ703753.1; J1c3i; Asia_W; 31; A73G; G185A; G228A	16569	Asia_W	J1c3i	J	J	31
1952	JQ703754.1	JQ703754.1; T2d1a; Asia_W; 39; A73G; T152C; A263G	16570	Asia_W	T2d1a	T	T	39
1953	JQ703755.1	JQ703755.1; U5b1h; Asia_W_Europe_C; 28; A73G; C150T; A263G	16571	Asia_W_Europe_C	U5b1h	U	U5	28
1954	JQ703756.1	JQ703756.1; H1ae3a; Asia_W_Europe; 14; A263G; C309CCCT; T310C	16571	Asia_W_Europe	H1ae3a	H	H	14
1955	JQ703757.1	JQ703757.1; H16b; Asia_W_Europe; 11; A263G; C315CC; A750G	16569	Asia_W_Europe	H16b	H	H	11
1956	JQ703758.1	JQ703758.1; H1a8a; Asia_W_Europe; 13; A73G; A263G; C315CC	16569	Asia_W_Europe	H1a8a	H	H	13
1957	JQ703759.1	JQ703759.1; H1a3a; Asia_W_Europe; 16; A73G; A263G; C309CCT	16570	Asia_W_Europe	H1a3a	H	H	16
1958	JQ703760.1	JQ703760.1; H2a1i; Asia_W_Europe; 13; C113T; A263G; C309CCT	16570	Asia_W_Europe	H2a1i	H	H	13
1959	JQ703761.1	JQ703761.1; H10c1; Asia_W_Europe; 17; A263G; C309CCT; T310C	16570	Asia_W_Europe	H10c1	H	H	17
1960	JQ703762.1	JQ703762.1; T2b13a; Asia_W; 39; A73G; A263G; C309CCT	16570	Asia_W	T2b13a	T	T	39
1961	JQ703763.1	JQ703763.1; K1a4a1a2b; Asia_W; 40; A73G; T146C; A153G	16570	Asia_W	K1a4a1a2b	K	K	40
1962	JQ703764.1	JQ703764.1; K1a10a; Asia_W; 34; A73G; T195C; A263G	16574	Asia_W	K1a10a	K	K	34
1963	JQ703765.1	JQ703765.1; H1n4; Asia_W_Europe; 13; T146C; A263G; C309CCCT	16571	Asia_W_Europe	H1n4	H	H	13
1964	JQ703766.1	JQ703766.1; H28a; Asia_W_Europe; 12; T146C; C186A; A263G	16569	Asia_W_Europe	H28a	H	H	12
1965	JQ703767.1	JQ703767.1; K2b1a1; Asia_W; 35; A73G; T146C; A263G	16569	Asia_W	K2b1a1	K	K	35
1966	JQ703768.1	JQ703768.1; H65; Asia_W_Europe; 13; A5C; T152C; A263G	16569	Asia_W_Europe	H65	H	H	13
1967	JQ703769.1	JQ703769.1; U3a1c; Africa_NE_Asia_W; 31; A73G; T146C; C150T	16569	Africa_NE_Asia_W	U3a1c	U	U3	31
1968	JQ703770.1	JQ703770.1; V10b1; Europe_S; 20; T72C; A263G; C309CCCT	16571	Europe_S	V10b1	V	V	20

Supplementary Table S4 Human mitochondrial database (hMITO DB v1.0) metadata[a]

Row	Name (accession no.)	Description	Size	Geo_Region	Haplogroup	Macro_ Haplo	Macro_ Haplo2	Total Variants
1969	JQ703771.1	JQ703771.1; X2c1; Asia_W_America_N; 30; A153G; T195C; G225A	16570	Asia_W_America_N	X2c1	X	X	30
1970	JQ703772.1	JQ703772.1; T2b6a; Asia_W; 40; A73G; T146C; A263G	16570	Asia_W	T2b6a	T	T	40
1971	JQ703773.1	JQ703773.1; L1c4b; Africa_C; 91; A73G; T152C; C182T	16571	Africa_C	L1c4b	L1	L1	91
1972	JQ703774.1	JQ703774.1; C1c1b; Asia_NE_America_N_S; 45; A73G; A215G; A249d	16567	Asia_NE_America_N_S	C1c1b	C	C	45
1973	JQ703775.1	JQ703775.1; K1a3a4; Asia_W; 37; A73G; A263G; C309CCT	16572	Asia_W	K1a3a4	K	K	37
1974	JQ703776.1	JQ703776.1; T2a1b1a2; Asia_W; 39; A73G; A263G; C315CC	16569	Asia_W	T2a1b1a2	T	T	39
1975	JQ703777.1	JQ703777.1; T2e; Asia_W; 38; A73G; C150T; A263G	16567	Asia_W	T2e	T	T	38
1976	JQ703778.1	JQ703778.1; J1c2b; Asia_W; 33; A73G; G185A; A188G	16570	Asia_W	J1c2b	J	J	33
1977	JQ703779.1	JQ703779.1; U8a1a1b1; Asia_W_Europe ; 33; A73G; A263G; T282C	16570	Asia_W_Europe	U8a1a1b1	U	U8	33
1978	JQ703780.1	JQ703780.1; U5b2b1a2; Asia_W_Europe_C; 37; A73G; C150T; A263G	16570	Asia_W_Europe_C	U5b2b1a2	U	U5	37
1979	JQ703781.1	JQ703781.1; H11b1; Asia_W_Europe; 18; T195C; A263G; C309CCT	16570	Asia_W_Europe	H11b1	H	H	18
1980	JQ703782.1	JQ703782.1; K1a4a1a2b; Asia_W; 41; A73G; T146C; T195C	16569	Asia_W	K1a4a1a2b	K	K	41
1981	JQ703783.1	JQ703783.1; J1c1b; Asia_W; 30; A73G; G185A; G228A	16569	Asia_W	J1c1b	J	J	30
1982	JQ703784.1	JQ703784.1; J2b1b; Asia_W; 35; A73G; C150T; T152C	16570	Asia_W	J2b1b	J	J	35
1983	JQ703785.1	JQ703785.1; J1c2s1; Asia_W; 33; A73G; C114T; G185A	16569	Asia_W	J1c2s1	J	J	33
1984	JQ703786.1	JQ703786.1; J1c3; Asia_W; 27; A73G; A263G; C295T	16570	Asia_W	J1c3	J	J	27
1985	JQ703787.1	JQ703787.1; H1c; Asia_W_Europe; 11; A263G; C315CC; T477C	16569	Asia_W_Europe	H1c	H	H	11
1986	JQ703788.1	JQ703788.1; H1aj1a; Asia_W_Europe; 16; G207A; A263G; C315CC	16569	Asia_W_Europe	H1aj1a	H	H	16
1987	JQ703789.1	JQ703789.1; J1c2o; Asia_W; 32; A73G; G185A; A188G	16570	Asia_W	J1c2o	J	J	32
1988	JQ703790.1	JQ703790.1; U5b2b3a1a; Asia_W_Europe_C; 37; A73G; C150T; A263G	16569	Asia_W_Europe_C	U5b2b3a1a	U	U5	37
1989	JQ703791.1	JQ703791.1; H5; Asia_W_Europe; 15; T152C; A263G; C309CCT	16568	Asia_W_Europe	H5	H	H	15
1990	JQ703792.1	JQ703792.1; J1c3a2; Asia_W; 30; A73G; G228A; A263G	16570	Asia_W	J1c3a2	J	J	30
1991	JQ703793.1	JQ703793.1; U1a1a2; Asia_W; 39; A73G; C262T; A263G	16569	Asia_W	U1a1a2	U	U1	39
1992	JQ703794.1	JQ703794.1; J1c2; Asia_W; 30; A73G; G185A; A188G	16570	Asia_W	J1c2	J	J	30
1993	JQ703795.1	JQ703795.1; H1; Asia_W_Europe; 13; A93G; A263G; C309CCCT	16571	Asia_W_Europe	H1	H	H	13
1994	JQ703796.1	JQ703796.1; U5b2a6; Asia_W_Europe_C; 29; A73G; C150T; A263G	16569	Asia_W_Europe_C	U5b2a6	U	U5	29
1995	JQ703797.1	JQ703797.1; H3; Asia_W_Europe; 13; C33T; A263G; C309CC	16569	Asia_W_Europe	H3	H	H	13
1996	JQ703798.1	JQ703798.1; H+152; Asia_W_Europe; 11; T152C; A263G; C309CCT	16570	Asia_W_Europe	H+152	H	H	11
1997	JQ703799.1	JQ703799.1; H5; Asia_W_Europe; 14; C150T; A263G; C309CCT	16570	Asia_W_Europe	H5	H	H	14
1998	JQ703800.1	JQ703800.1; H5a3b; Asia_W_Europe; 16; T199C; A263G; C309CCT	16570	Asia_W_Europe	H5a3b	H	H	16
1999	JQ703801.1	JQ703801.1; J2a1a1; Asia_W; 40; A73G; C150T; T152C	16572	Asia_W	J2a1a1	J	J	40
2000	JQ703802.1	JQ703802.1; J1b1a1; Asia_W; 38; A73G; C242T; A263G	16569	Asia_W	J1b1a1	J	J	38
2001	JQ703803.1	JQ703803.1; J1c5a; Asia_W; 36; A73G; G185A; T195C	16570	Asia_W	J1c5a	J	J	36
2002	JQ703804.1	JQ703804.1; H61a; Asia_W_Europe; 12; A263G; C315CC; A750G	16569	Asia_W_Europe	H61a	H	H	12
2003	JQ703805.1	JQ703805.1; K1a1b1e; Asia_W; 34; A73G; A263G; C315CC	16569	Asia_W	K1a1b1e	K	K	34
2004	JQ703806.1	JQ703806.1; T2f1a1; Asia_W; 45; A73G; C150T; T195C	16561	Asia_W	T2f1a1	T	T	45
2005	JQ703807.1	JQ703807.1; K1a4a1g; Asia_W; 38; A73G; A263G; C309CCT	16570	Asia_W	K1a4a1g	K	K	38
2006	JQ703808.1	JQ703808.1; H13a1a1d; Asia_W_Europe; 20; A263G; C309CCCT; T310C	16571	Asia_W_Europe	H13a1a1d	H	H	20
2007	JQ703809.1	JQ703809.1; A2l; Asia_NE_America_N; 32; C64T; A73G; T146C	16567	Asia_NE_America_N	A2l	A	A	32
2008	JQ703810.1	JQ703810.1; H1c9; Asia_W_Europe; 14; T152C; A263G; C309CCT	16570	Asia_W_Europe	H1c9	H	H	14
2009	JQ703811.1	JQ703811.1; H1ag1; Asia_W_Europe; 12; A263G; C315CC; A750G	16569	Asia_W_Europe	H1ag1	H	H	12

Supplementary Table S4 Human mitochondrial database (hMITO DB v1.0) metadata[a]

Row	Name (accession no.)	Description	Size	Geo_Region	Haplogroup	Macro_ Haplo	Macro_ Haplo2	Total Variants
2010	JQ703812.1	JQ703812.1; M7c1c3; Asia_E_SE; 41; A73G; T146C; T199C	16568	Asia_E_SE	M7c1c3	M	M7	41
2011	JQ703813.1	JQ703813.1; H6a1a4; Asia_W_Europe; 17; T239C; A263G; C309CCT	16570	Asia_W_Europe	H6a1a4	H	H	17
2012	JQ703814.1	JQ703814.1; H1a1; Asia_W_Europe; 14; A73G; A263G; C315CC	16569	Asia_W_Europe	H1a1	H	H	14
2013	JQ703815.1	JQ703815.1; K1c1f; Asia_W; 37; A73G; T146C; T152C	16569	Asia_W	K1c1f	K	K	37
2014	JQ703816.1	JQ703816.1; H3ap; Asia_W_Europe; 11; A263G; C309CCT; T310C	16568	Asia_W_Europe	H3ap	H	H	11
2015	JQ703817.1	JQ703817.1; H1ba; Asia_W_Europe; 13; A263G; C309CCT; T310C	16570	Asia_W_Europe	H1ba	H	H	13
2016	JQ703818.1	JQ703818.1; J1c2; Asia_W; 30; A73G; G185A; A188G	16569	Asia_W	J1c2	J	J	30
2017	JQ703819.1	JQ703819.1; H3i1; Asia_W_Europe; 13; T152C; A263G; C315CC	16569	Asia_W_Europe	H3i1	H	H	13
2018	JQ703820.1	JQ703820.1; H5a1g2; Asia_W_Europe; 14; A263G; C315CC; C456T	16567	Asia_W_Europe	H5a1g2	H	H	14
2019	JQ703821.1	JQ703821.1; H45b; Asia_W_Europe; 11; A263G; C315CC; A750G	16569	Asia_W_Europe	H45b	H	H	11
2020	JQ703822.1	JQ703822.1; J1b1a1a; Asia_W; 39; A73G; C242T; A263G	16569	Asia_W	J1b1a1a	J	J	39
2021	JQ703823.1	JQ703823.1; T2c1e; Asia_W; 40; A73G; T146C; A263G	16568	Asia_W	T2c1e	T	T	40
2022	JQ703824.1	JQ703824.1; H5a3b; Asia_W_Europe; 14; A263G; C309CCCT; T310C	16571	Asia_W_Europe	H5a3b	H	H	14
2023	JQ703825.1	JQ703825.1; J1c1b1a; Asia_W; 34; A73G; G185A; G228A	16569	Asia_W	J1c1b1a	J	J	34
2024	JQ703826.1	JQ703826.1; A2+(64); Asia_NE_America_N; 36; C64T; A73G; T146C	16568	Asia_NE_America_N	A2+(64)	A	A	36
2025	JQ703827.1	JQ703827.1; C1c7; Asia_NE_America_N_S; 47; A73G; T195C; A249d	16567	Asia_NE_America_N_S	C1c7	C	C	47
2026	JQ703828.1	JQ703828.1; B2a2; Asia_SE_E_America_N_S; 34; A73G; A263G; C309CCCT	16562	Asia_SE_E_America_N_S	B2a2	B	B	34
2027	JQ703829.1	JQ703829.1; I2d; Asia_W_SW; 36; A73G; T152C; T199C	16572	Asia_W_SW	I2d	I	I	36
2028	JQ703830.1	JQ703830.1; V7a; Europe_S; 23; T72C; T89C; A93G	16570	Europe_S	V7a	V	V	23
2029	JQ703831.1	JQ703831.1; U5a2c1; Asia_W_Europe_C; 27; A73G; A263G; C315CC	16569	Asia_W_Europe_C	U5a2c1	U	U5	27
2030	JQ703832.1	JQ703832.1; A2g; Asia_NE_America_N; 36; C64T; A73G; T146C	16568	Asia_NE_America_N	A2g	A	A	36
2031	JQ703833.1	JQ703833.1; A2+(64)+16189; Asia_NE_America_N; 43; C64T; A73G; G143A	16566	Asia_NE_America_N	A2+(64)+16189	A	A	43
2032	JQ703834.1	JQ703834.1; B2a1a1; Asia_SE_E_America_N_S; 32; A73G; T195C; A263G	16561	Asia_SE_E_America_N_S	B2a1a1	B	B	32
2033	JQ703835.1	JQ703835.1; I1a1; Asia_W_SW; 42; A73G; T199C; G203A	16572	Asia_W_SW	I1a1	I	I	42
2034	JQ703836.1	JQ703836.1; V3a1; Europe_S; 21; T72C; A189G; A263G	16572	Europe_S	V3a1	V	V	21
2035	JQ703837.1	JQ703837.1; K2a3; Asia_W; 35; A73G; T146C; T152C	16569	Asia_W	K2a3	K	K	35
2036	JQ703838.1	JQ703838.1; H1b1e; Asia_W_Europe; 13; A263G; C315CC; A750G	16569	Asia_W_Europe	H1b1e	H	H	13
2037	JQ703839.1	JQ703839.1; H1z; Asia_W_Europe; 17; A263G; C309CCCT; T310C	16571	Asia_W_Europe	H1z	H	H	17
2038	JQ703840.1	JQ703840.1; C1c; Asia_NE_America_N_S; 45; A73G; C150T; T152C	16567	Asia_NE_America_N_S	C1c	C	C	45
2039	JQ703841.1	JQ703841.1; X2b+226; Asia_W_America_N; 32; A73G; T195C; A200G	16570	Asia_W_America_N	X2b+226	X	X	32
2040	JQ703842.1	JQ703842.1; T2b4b; Asia_W; 37; A73G; T152C; A263G	16572	Asia_W	T2b4b	T	T	37
2041	JQ703843.1	JQ703843.1; H3y; Asia_W_Europe; 14; T195C; A263G; C315CC	16569	Asia_W_Europe	H3y	H	H	14
2042	JQ703844.1	JQ703844.1; M7c1c3d; Asia_E_SE; 42; A73G; T146C; T199C	16568	Asia_E_SE	M7c1c3d	M	M7	42
2043	JQ703845.1	JQ703845.1; H4a1a1a1a1; Asia_W_Europe; 24; A73G; T152C; A263G	16567	Asia_W_Europe	H4a1a1a1a1	H	H	24
2044	JQ703846.1	JQ703846.1; H5a6; Asia_W_Europe; 14; T152C; A263G; C315CC	16569	Asia_W_Europe	H5a6	H	H	14
2045	JQ703847.1	JQ703847.1; H48; Asia_W_Europe; 11; A263G; C309CCT; T310C	16570	Asia_W_Europe	H48	H	H	11
2046	JQ703848.1	JQ703848.1; H1b1+16362; Asia_W_Europe; 13; A263G; C315CC; CA522d	16567	Asia_W_Europe	H1b1+16362	H	H	13
2047	JQ703849.1	JQ703849.1; U5b1a; Asia_W_Europe_C; 30; A73G; C150T; T204C	16570	Asia_W_Europe_C	U5b1a	U	U5	30
2048	JQ703850.1	JQ703850.1; I2; Asia_W_SW; 37; A73G; T152C; T199C	16572	Asia_W_SW	I2	I	I	37
2049	JQ703851.1	JQ703851.1; B2o; Asia_SE_E_America_N_S; 34; A73G; T159C; A263G	16560	Asia_SE_E_America_N_S	B2o	B	B	34
2050	JQ703852.1	JQ703852.1; B2a4; Asia_SE_E_America_N_S; 33; A73G; A263G; C315CC	16560	Asia_SE_E_America_N_S	B2a4	B	B	33

Supplementary Table S4 Human mitochondrial database (hMITO DB v1.0) metadata[a]

Row	Name (accession no.)	Description	Size	Geo_Region	Haplogroup	Macro_ Haplo	Macro_ Haplo2	Total Variants
2051	JQ703853.1	JQ703853.1; C1b1; Asia_NE_America_N_S; 47; A73G; A249d; A263G	16565	Asia_NE_America_N_S	C1b1	C	C	47
2052	JQ703854.1	JQ703854.1; H2a1; Asia_W_Europe; 10; T152C; A263G; C309CCT	16570	Asia_W_Europe	H2a1	H	H	10
2053	JQ703855.1	JQ703855.1; K1a1b1a; Asia_W; 40; A73G; C114T; A263G	16569	Asia_W	K1a1b1a	K	K	40
2054	JQ703856.1	JQ703856.1; H1j2a; Asia_W_Europe; 17; T152C; A263G; C309CCCT	16571	Asia_W_Europe	H1j2a	H	H	17
2055	JQ703857.1	JQ703857.1; H; Asia_W_Europe; 14; T146C; A263G; C315CC	16571	Asia_W_Europe	H	H	H	14
2056	JQ703858.1	JQ703858.1; H13a1a; Asia_W_Europe; 16; A263G; C309CCCT; T310C	16571	Asia_W_Europe	H13a1a	H	H	16
2057	JQ703859.1	JQ703859.1; K2b1a1; Asia_W; 35; A73G; T146C; A263G	16569	Asia_W	K2b1a1	K	K	35
2058	JQ703860.1	JQ703860.1; K1c1c; Asia_W; 34; A73G; T146C; T152C	16568	Asia_W	K1c1c	K	K	34
2059	JQ703861.1	JQ703861.1; J1c7; Asia_W; 32; A73G; G185A; G228A	16569	Asia_W	J1c7	J	J	32
2060	JQ703862.1	JQ703862.1; I3d1; Asia_W_SW; 41; A73G; T152C; A183G	16579	Asia_W_SW	I3d1	I	I	41
2061	JQ703863.1	JQ703863.1; H13a1a; Asia_W_Europe; 14; A263G; C315CC; A750G	16569	Asia_W_Europe	H13a1a	H	H	14
2062	JQ703864.1	JQ703864.1; H2a2a1b; Asia_W_Europe; 2; G5046A; A9299G;	16568	Asia_W_Europe	H2a2a1b	H	H	2
2063	JQ703865.1	JQ703865.1; D1n; Asia_E_America_N_S; 36; A73G; A263G; C309CCT	16570	Asia_E_America_N_S	D1n	D	D	36
2064	JQ703866.1	JQ703866.1; U5a1a1+152; Asia_W_Europe_C; 30; A73G; T152C; A263G	16569	Asia_W_Europe_C	U5a1a1+152	U	U5	30
2065	JQ703867.1	JQ703867.1; V1a; Europe_S; 17; T72C; A263G; C315CC	16569	Europe_S	V1a	V	V	17
2066	JQ703868.1	JQ703868.1; T2a1b1a1a1; Asia_W; 40; A73G; A263G; C315CC	16569	Asia_W	T2a1b1a1a1	T	T	40
2067	JQ703869.1	JQ703869.1; H1a1; Asia_W_Europe; 13; A73G; A263G; C315CC	16569	Asia_W_Europe	H1a1	H	H	13
2068	JQ703870.1	JQ703870.1; J1c2; Asia_W; 32; A73G; G185A; A188G	16568	Asia_W	J1c2	J	J	32
2069	JQ703871.1	JQ703871.1; H14a2b; Asia_W_Europe; 14; A263G; C315CC; A750G	16569	Asia_W_Europe	H14a2b	H	H	14
2070	JQ703872.1	JQ703872.1; B2g1; Asia_SE_E_America_N_S; 39; A73G; C114G; A263G	16563	Asia_SE_E_America_N_S	B2g1	B	B	39
2071	JQ703873.1	JQ703873.1; A2i; Asia_NE_America_N; 43; C64T; A73G; G94A	16570	Asia_NE_America_N	A2i	A	A	43
2072	JQ703874.1	JQ703874.1; B4g1; Asia_SE_E_America_N_S; 31; A73G; T152C; A263G	16560	Asia_SE_E_America_N_S	B4g1	B	B	31
2073	JQ703875.1	JQ703875.1; A2w1; Asia_NE_America_N; 38; C64T; A73G; T146C	16570	Asia_NE_America_N	A2w1	A	A	38
2074	JQ703876.1	JQ703876.1; K1b2b; Asia_W; 38; A73G; T146C; T195C	16572	Asia_W	K1b2b	K	K	38
2075	JQ703877.1	JQ703877.1; H10e; Asia_W_Europe; 11; A263G; C309CCT; T310C	16570	Asia_W_Europe	H10e	H	H	11
2076	JQ703878.1	JQ703878.1; H13a2c1; Asia_W_Europe; 23; T152C; A189G; A193G	16571	Asia_W_Europe	H13a2c1	H	H	23
2077	JQ703879.1	JQ703879.1; H1u1; Asia_W_Europe; 12; A263G; C315CC; A750G	16569	Asia_W_Europe	H1u1	H	H	12
2078	JQ703880.1	JQ703880.1; K1a4a1a2b; Asia_W; 41; A73G; T146C; A263G	16572	Asia_W	K1a4a1a2b	K	K	41
2079	JQ703881.1	JQ703881.1; H2a1n; Asia_W_Europe; 9; T146C; A263G; C309CCT	16570	Asia_W_Europe	H2a1n	H	H	9
2080	JQ703882.1	JQ703882.1; J1c2c; Asia_W; 34; A73G; T146C; G185A	16569	Asia_W	J1c2c	J	J	34
2081	JQ703883.1	JQ703883.1; I3c; Asia_W_SW; 37; A73G; T152C; T199C	16576	Asia_W_SW	I3c	I	I	37
2082	JQ703884.1	JQ703884.1; B4b1a2; Asia_SE_E_America_N_S; 31; A73G; G207A; A263G	16560	Asia_SE_E_America_N_S	B4b1a2	B	B	31
2083	JQ703885.1	JQ703885.1; C5c1a; Asia_NE_America_N_S; 48; A73G; T152C; A249d	16569	Asia_NE_America_N_S	C5c1a	C	C	48
2084	JQ703886.1	JQ703886.1; U4b1b1a; Asia_N_Europe_N; 34; A73G; T146C; T152C	16570	Asia_N_Europe_N	U4b1b1a	U	U4	34
2085	JQ703887.1	JQ703887.1; U4a1a2; Asia_N_Europe_N; 34; A73G; T152C; T195C	16573	Asia_N_Europe_N	U4a1a2	U	U4	34
2086	JQ703888.1	JQ703888.1; H1ba; Asia_W_Europe; 14; A263G; C309CCT; T310C	16570	Asia_W_Europe	H1ba	H	H	14
2087	JQ703889.1	JQ703889.1; H1c2; Asia_W_Europe; 12; A263G; C315CC; T477C	16569	Asia_W_Europe	H1c2	H	H	12
2088	JQ703890.1	JQ703890.1; T2a1a; Asia_W; 35; A73G; A263G; C315CC	16569	Asia_W	T2a1a	T	T	35
2089	JQ703891.1	JQ703891.1; K1a24; Asia_W; 33; A73G; C150T; A263G	16569	Asia_W	K1a24	K	K	33
2090	JQ703892.1	JQ703892.1; H4a1a1a; Asia_W_Europe; 20; A73G; A263G; C309CCT	16568	Asia_W_Europe	H4a1a1a	H	H	20
2091	JQ703893.1	JQ703893.1; H1; Asia_W_Europe; 10; A93G; A263G; C315CC	16569	Asia_W_Europe	H1	H	H	10

Supplementary Table S4 Human mitochondrial database (hMITO DB v1.0) metadata[a]

Row	Name (accession no.)	Description	Size	Geo_Region	Haplogroup	Macro_ Haplo	Macro_ Haplo2	Total Variants
2092	JQ703894.1	JQ703894.1; T1a1c; Asia_W; 40; A73G; T152C; T195C	16570	Asia_W	T1a1c	T	T	40
2093	JQ703895.1	JQ703895.1; J1c3e1; Asia_W; 35; A73G; G185A; T195C	16570	Asia_W	J1c3e1	J	J	35
2094	JQ703896.1	JQ703896.1; U5b2a1a1; Asia_W_Europe_C; 32; A73G; C150T; A263G	16570	Asia_W_Europe_C	U5b2a1a1	U	U5	32
2095	JQ703897.1	JQ703897.1; H4a1; Asia_W_Europe; 21; A93G; C194T; A263G	16567	Asia_W_Europe	H4a1	H	H	21
2096	JQ703898.1	JQ703898.1; H24a1; Asia_W_Europe; 12; A263G; C315CCC; A750G	16570	Asia_W_Europe	H24a1	H	H	12
2097	JQ703899.1	JQ703899.1; U5b2a1a1; Asia_W_Europe_C; 30; A73G; C150T; A263G	16569	Asia_W_Europe_C	U5b2a1a1	U	U5	30
2098	JQ703900.1	JQ703900.1; U4c1; Asia_N_Europe_N; 36; A73G; T195C; A263G	16571	Asia_N_Europe_N	U4c1	U	U4	36
2099	JQ703901.1	JQ703901.1; H1a1a; Asia_W_Europe; 17; A73G; T152C; A263G	16569	Asia_W_Europe	H1a1a	H	H	17
2100	JQ703902.1	JQ703902.1; U6a5; Asia_SW_Africa_N; 30; A73G; A263G; C309CCT	16570	Asia_SW_Africa_N	U6a5	U	U6	30
2101	JQ703903.1	JQ703903.1; U4a1; Asia_N_Europe_N; 32; A73G; T152C; T195C	16571	Asia_N_Europe_N	U4a1	U	U4	32
2102	JQ703904.1	JQ703904.1; U5b2c2; Asia_W_Europe_C; 32; A73G; C150T; A263G	16571	Asia_W_Europe_C	U5b2c2	U	U5	32
2103	JQ703905.1	JQ703905.1; U5b2b1a1; Asia_W_Europe_C; 32; A73G; C150T; A263G	16569	Asia_W_Europe_C	U5b2b1a1	U	U5	32
2104	JQ703906.1	JQ703906.1; U5a1a1a; Asia_W_Europe_C; 30; A73G; T152C; A263G	16570	Asia_W_Europe_C	U5a1a1a	U	U5	30
2105	JQ703907.1	JQ703907.1; H1bb; Asia_W_Europe; 14; T152C; A263G; C309CCT	16572	Asia_W_Europe	H1bb	H	H	14
2106	JQ703908.1	JQ703908.1; X2d1; Asia_W_America_N; 33; A73G; T195C; T204C	16569	Asia_W_America_N	X2d1	X	X	33
2107	JQ703909.1	JQ703909.1; H65; Asia_W_Europe; 13; A263G; C309CCT; T310C	16570	Asia_W_Europe	H65	H	H	13
2108	JQ703910.1	JQ703910.1; I2a2; Asia_W_SW; 37; A73G; T152C; T199C	16574	Asia_W_SW	I2a2	I	I	37
2109	JQ703911.1	JQ703911.1; U3b1; Africa_NE_Asia_W; 30; A73G; C150T; A263G	16569	Africa_NE_Asia_W	U3b1	U	U3	30
2110	JQ703912.1	JQ703912.1; U4b1a1a; Asia_N_Europe_N; 34; A73G; A189G; T195C	16570	Asia_N_Europe_N	U4b1a1a	U	U4	34
2111	JQ703913.1	JQ703913.1; U7a5; Asia_W; 37; A73G; C151T; T152C	16569	Asia_W	U7a5	U	U7	37
2112	JQ703914.1	JQ703914.1; U5b2b4a; Asia_W_Europe_C; 34; A73G; C150T; G185A	16572	Asia_W_Europe_C	U5b2b4a	U	U5	34
2113	JQ703915.1	JQ703915.1; J1d5; Asia_W; 36; A73G; T146C; T152C	16569	Asia_W	J1d5	J	J	36
2114	JQ703916.1	JQ703916.1; J1c4b; Asia_W; 30; A73G; G185A; G228A	16569	Asia_W	J1c4b	J	J	30
2115	JQ703917.1	JQ703917.1; U2e1b1; Asia_S_W_Europe; 41; A73G; T152C; T217C	16571	Asia_S_W_Europe	U2e1b1	U	U2	41
2116	JQ703918.1	JQ703918.1; V1a1; Europe_S; 16; A263G; C309CCT; T310C	16570	Europe_S	V1a1	V	V	16
2117	JQ703919.1	JQ703919.1; J1c6; Asia_W; 30; A73G; G185A; G228A	16569	Asia_W	J1c6	J	J	30
2118	JQ703920.1	JQ703920.1; H49a; Asia_W_Europe; 15; T195C; A263G; C309CCT	16574	Asia_W_Europe	H49a	H	H	15
2119	JQ703921.1	JQ703921.1; H5b1; Asia_W_Europe; 14; T146C; T195C; A263G	16570	Asia_W_Europe	H5b1	H	H	14
2120	JQ703922.1	JQ703922.1; H1; Asia_W_Europe; 12; T146C; A263G; C315CC	16569	Asia_W_Europe	H1	H	H	12
2121	JQ703923.1	JQ703923.1; H1ay; Asia_W_Europe; 17; T146C; A263G; C315CC	16568	Asia_W_Europe	H1ay	H	H	17
2122	JQ703924.1	JQ703924.1; V15a; Europe_S; 17; T72C; A263G; C309CCCT	16571	Europe_S	V15a	V	V	17
2123	JQ703925.1	JQ703925.1; H1a; Asia_W_Europe; 14; A73G; T152C; A263G	16570	Asia_W_Europe	H1a	H	H	14
2124	JQ703926.1	JQ703926.1; U5a1a2a1; Asia_W_Europe_C; 32; A73G; A263G; C309CCT	16571	Asia_W_Europe_C	U5a1a2a1	U	U5	32
2125	JQ703927.1	JQ703927.1; U5a1b; Asia_W_Europe_C; 26; A73G; A263G; C315CC	16569	Asia_W_Europe_C	U5a1b	U	U5	26
2126	JQ703928.1	JQ703928.1; U5a1b1a2; Asia_W_Europe_C; 34; A73G; A263G; C315CC	16569	Asia_W_Europe_C	U5a1b1a2	U	U5	34
2127	JQ703929.1	JQ703929.1; U4a1a; Asia_N_Europe_N; 36; A73G; T152C; T195C	16571	Asia_N_Europe_N	U4a1a	U	U4	36
2128	JQ703930.1	JQ703930.1; U1a1d; Asia_W; 38; A73G; A263G; C285T	16571	Asia_W	U1a1d	U	U1	38
2129	JQ703931.1	JQ703931.1; N1a1a1a2; Africa_NE_Asia_W; 41; A73G; T152C; A189G	16571	Africa_NE_Asia_W	N1a1a1a2	N	N1	41
2130	JQ703932.1	JQ703932.1; J1c7a; Asia_W; 34; A73G; G185A; G228A	16570	Asia_W	J1c7a	J	J	34
2131	JQ703933.1	JQ703933.1; T2b; Asia_W; 36; A73G; A263G; C315CC	16569	Asia_W	T2b	T	T	36
2132	JQ703934.1	JQ703934.1; U5b1c2; Asia_W_Europe_C; 30; A73G; C150T; A263G	16569	Asia_W_Europe_C	U5b1c2	U	U5	30

Supplementary Table S4 Human mitochondrial database (hMITO DB v1.0) metadata[a]

Row	Name (accession no.)	Description	Size	Geo_Region	Haplogroup	Macro_ Haplo	Macro_ Haplo2	Total Variants
2133	JQ703935.1	JQ703935.1; H+195; Asia_W_Europe; 14; T195C; A263G; C309CCT	16570	Asia_W_Europe	H+195	H	H	14
2134	JQ703936.1	JQ703936.1; U5a2a1; Asia_W_Europe_C; 32; A73G; A263G; C309CCT	16570	Asia_W_Europe_C	U5a2a1	U	U5	32
2135	JQ703937.1	JQ703937.1; U5a1a1b; Asia_W_Europe_C; 30; A73G; T152C; A263G	16569	Asia_W_Europe_C	U5a1a1b	U	U5	30
2136	JQ703938.1	JQ703938.1; H1ag1a; Asia_W_Europe; 14; A263G; C309CCT; T310C	16570	Asia_W_Europe	H1ag1a	H	H	14
2137	JQ703939.1	JQ703939.1; H4a2; Asia_W_Europe; 17; A263G; C309CCT; T310C	16568	Asia_W_Europe	H4a2	H	H	17
2138	JQ703940.1	JQ703940.1; H31b; Asia_W_Europe; 13; T146C; T195C; A263G	16569	Asia_W_Europe	H31b	H	H	13
2139	JQ703941.1	JQ703941.1; H1b1b; Asia_W_Europe; 20; A263G; C309CCCT; T310C	16569	Asia_W_Europe	H1b1b	H	H	20
2140	JQ703942.1	JQ703942.1; U5b2a1a1b; Asia_W_Europe_C; 32; A73G; C150T; A249d	16569	Asia_W_Europe_C	U5b2a1a1b	U	U5	32
2141	JQ703943.1	JQ703943.1; H3v+16093; Asia_W_Europe; 17; A263G; C309CCT; T310C	16570	Asia_W_Europe	H3v+16093	H	H	17
2142	JQ703944.1	JQ703944.1; U5b1c2a; Asia_W_Europe_C; 34; A73G; C150T; A263G	16571	Asia_W_Europe_C	U5b1c2a	U	U5	34
2143	JQ703945.1	JQ703945.1; U5a1b1; Asia_W_Europe_C; 29; A73G; A263G; C315CC	16569	Asia_W_Europe_C	U5a1b1	U	U5	29
2144	JQ703946.1	JQ703946.1; U5b2a2a1; Asia_W_Europe_C; 32; A73G; C150T; A263G	16569	Asia_W_Europe_C	U5b2a2a1	U	U5	32
2145	JQ703947.1	JQ703947.1; U4a1; Asia_N_Europe_N; 31; A73G; T152C; T195C	16573	Asia_N_Europe_N	U4a1	U	U4	31
2146	JQ703948.1	JQ703948.1; H1g2; Asia_W_Europe; 14; A73G; A263G; C315CC	16569	Asia_W_Europe	H1g2	H	H	14
2147	JQ703949.1	JQ703949.1; H11a5; Asia_W_Europe; 16; T195C; A263G; C315CC	16569	Asia_W_Europe	H11a5	H	H	16
2148	JQ703950.1	JQ703950.1; H28a; Asia_W_Europe; 15; T42TG; C182T; C186A	16571	Asia_W_Europe	H28a	H	H	15
2149	JQ703951.1	JQ703951.1; H7a1c; Asia_W_Europe; 16; A263G; C315CC; A750G	16569	Asia_W_Europe	H7a1c	H	H	16
2150	JQ703952.1	JQ703952.1; V3c; Europe_S; 18; T72C; A263G; C309CCT	16570	Europe_S	V3c	V	V	18
2151	JQ703953.1	JQ703953.1; T2b; Asia_W; 33; A73G; C315CC; G709A	16569	Asia_W	T2b	T	T	33
2152	JQ703954.1	JQ703954.1; H2a1c; Asia_W_Europe; 11; T152C; A263G; C309CCT	16570	Asia_W_Europe	H2a1c	H	H	11
2153	JQ703955.1	JQ703955.1; J1c2a2; Asia_W; 34; A73G; G185A; A188G	16570	Asia_W	J1c2a2	J	J	34
2154	JQ703956.1	JQ703956.1; H1c1; Asia_W_Europe; 13; C151T; A263G; C315CC	16569	Asia_W_Europe	H1c1	H	H	13
2155	JQ703957.1	JQ703957.1; U2e1a1; Asia_S_W_Europe; 41; A73G; T152C; T217C	16569	Asia_S_W_Europe	U2e1a1	U	U2	41
2156	JQ703958.1	JQ703958.1; U5b1b1a; Asia_W_Europe_C; 30; A73G; C150T; A263G	16570	Asia_W_Europe_C	U5b1b1a	U	U5	30
2157	JQ703959.1	JQ703959.1; U5b1b1+@16192; Asia_W_Europe_C; 30; A73G; C150T; A263G	16569	Asia_W_Europe_C	U5b1b1+@161[illegible]	U	U5	30
2158	JQ703960.1	JQ703960.1; L2a1f; Africa_W_C; 54; A73G; T146C; T152C	16570	Africa_W_C	L2a1f	L2	L2	54
2159	JQ703961.1	JQ703961.1; H; Asia_W_Europe; 13; A93G; A263G; C309CCT	16570	Asia_W_Europe	H	H	H	13
2160	JQ703962.1	JQ703962.1; U5a2e; Asia_W_Europe_C; 33; A73G; C151T; T152C	16571	Asia_W_Europe_C	U5a2e	U	U5	33
2161	JQ703963.1	JQ703963.1; U5a1b1a; Asia_W_Europe_C; 27; A73G; A263G; C315CC	16569	Asia_W_Europe_C	U5a1b1a	U	U5	27
2162	JQ703964.1	JQ703964.1; U5b2a1a; Asia_W_Europe_C; 32; A73G; C150T; A263G	16560	Asia_W_Europe_C	U5b2a1a	U	U5	32
2163	JQ703965.1	JQ703965.1; U5a1a1; Asia_W_Europe_C; 32; A73G; A263G; C309CCT	16570	Asia_W_Europe_C	U5a1a1	U	U5	32
2164	JQ703966.1	JQ703966.1; Z1a1a; Asia_C_E; 46; A73G; C151T; T152C	16570	Asia_C_E	Z1a1a	Z	Z	46
2165	JQ703967.1	JQ703967.1; H1; Asia_W_Europe; 12; A263G; C309CCCT; T310C	16569	Asia_W_Europe	H1	H	H	12
2166	JQ703968.1	JQ703968.1; U2e2a1a; Asia_S_W_Europe; 41; A73G; T152C; T217C	16570	Asia_S_W_Europe	U2e2a1a	U	U2	41
2167	JQ703969.1	JQ703969.1; K1b2a1; Asia_W; 36; A73G; T146C; T195C	16570	Asia_W	K1b2a1	K	K	36
2168	JQ703970.1	JQ703970.1; W1h1; Asia_W; 35; A73G; T152C; A189G	16569	Asia_W	W1h1	W	W	35
2169	JQ703971.1	JQ703971.1; U5b3b2; Asia_W_Europe_C; 31; A73G; C150T; G228A	16570	Asia_W_Europe_C	U5b3b2	U	U5	31
2170	JQ703972.1	JQ703972.1; H24a; Asia_W_Europe; 12; A263G; C315CC; A750G	16569	Asia_W_Europe	H24a	H	H	12
2171	JQ703973.1	JQ703973.1; V17; Europe_S; 18; T72C; A263G; C309CCT	16570	Europe_S	V17	V	V	18
2172	JQ703974.1	JQ703974.1; H1at1a; Asia_W_Europe; 13; A263G; C315CC; A750G	16569	Asia_W_Europe	H1at1a	H	H	13
2173	JQ703975.1	JQ703975.1; D1; Asia_E_America_N_S; 41; A73G; C150T; A263G	16569	Asia_E_America_N_S	D1	D	D	41

Supplementary Table S4 Human mitochondrial database (hMITO DB v1.0) metadata[a]

Row	Name (accession no.)	Description	Size	Geo_Region	Haplogroup	Macro_ Haplo	Macro_ Haplo2	Total Variants
2174	JQ703976.1	JQ703976.1; A2ak; Asia_NE_America_N; 40; A73G; T146C; A153G	16568	Asia_NE_America_N	A2ak	A	A	40
2175	JQ703977.1	JQ703977.1; U4a1a3; Asia_N_Europe_N; 35; A73G; T152C; T195C	16570	Asia_N_Europe_N	U4a1a3	U	U4	35
2176	JQ703978.1	JQ703978.1; U7a5; Asia_W; 38; A73G; C151T; T152C	16570	Asia_W	U7a5	U	U7	38
2177	JQ703979.1	JQ703979.1; U5a2c1; Asia_W_Europe_C; 24; A73G; A263G; C315CC	16569	Asia_W_Europe_C	U5a2c1	U	U5	24
2178	JQ703980.1	JQ703980.1; U5b2c2b; Asia_W_Europe_C; 37; A73G; C150T; A263G	16571	Asia_W_Europe_C	U5b2c2b	U	U5	37
2179	JQ703981.1	JQ703981.1; HV0f; Asia_W; 13; T195C; A263G; C315CC	16569	Asia_W	HV0f	HV	HV	13
2180	JQ703982.1	JQ703982.1; H24a; Asia_W_Europe; 12; T236C; A263G; C309CCT	16570	Asia_W_Europe	H24a	H	H	12
2181	JQ703983.1	JQ703983.1; U5b1c2; Asia_W_Europe_C; 32; A73G; C150T; A263G	16571	Asia_W_Europe_C	U5b1c2	U	U5	32
2182	JQ703984.1	JQ703984.1; U5b2a1a1; Asia_W_Europe_C; 29; A73G; C150T; A263G	16569	Asia_W_Europe_C	U5b2a1a1	U	U5	29
2183	JQ703985.1	JQ703985.1; V8; Europe_S; 17; T72C; A263G; C309CCT	16570	Europe_S	V8	V	V	17
2184	JQ703986.1	JQ703986.1; L3b3; Africa_E; 42; A73G; G185A; A189G	16566	Africa_E	L3b3	L3	L3	42
2185	JQ703987.1	JQ703987.1; U5a2b; Asia_W_Europe_C; 27; A73G; A263G; C309CCT	16572	Asia_W_Europe_C	U5a2b	U	U5	27
2186	JQ703988.1	JQ703988.1; U5a2a1d; Asia_W_Europe_C; 31; A73G; A263G; C315CC	16571	Asia_W_Europe_C	U5a2a1d	U	U5	31
2187	JQ703989.1	JQ703989.1; V1a; Europe_S; 17; A263G; C309CCT; T310C	16570	Europe_S	V1a	V	V	17
2188	JQ703990.1	JQ703990.1; U3a1a; Africa_NE_Asia_W; 32; A73G; C150T; A263G	16570	Africa_NE_Asia_W	U3a1a	U	U3	32
2189	JQ703991.1	JQ703991.1; V15a; Europe_S; 16; T72C; A263G; C309CCT	16570	Europe_S	V15a	V	V	16
2190	JQ703992.1	JQ703992.1; V1a1; Europe_S; 18; T72C; A263G; C309CCT	16570	Europe_S	V1a1	V	V	18
2191	JQ703993.1	JQ703993.1; V7; Europe_S; 17; A93G; A263G; C309CCT	16570	Europe_S	V7	V	V	17
2192	JQ703994.1	JQ703994.1; U2e1; Asia_S_W_Europe; 40; A73G; T152C; T217C	16571	Asia_S_W_Europe	U2e1	U	U2	40
2193	JQ703995.1	JQ703995.1; H1m1; Asia_W_Europe; 15; T146C; A263G; C309CCCT	16571	Asia_W_Europe	H1m1	H	H	15
2194	JQ703996.1	JQ703996.1; V; Europe_S; 16; T72C; A263G; C309CCT	16570	Europe_S	V	V	V	16
2195	JQ703997.1	JQ703997.1; T2f; Asia_W; 32; A73G; A263G; C315CC	16560	Asia_W	T2f	T	T	32
2196	JQ703998.1	JQ703998.1; H1c19; Asia_W_Europe; 12; A263G; C315CC; T477C	16569	Asia_W_Europe	H1c19	H	H	12
2197	JQ703999.1	JQ703999.1; U5b2a2b1; Asia_W_Europe_C; 33; A73G; C150T; A263G	16569	Asia_W_Europe_C	U5b2a2b1	U	U5	33
2198	JQ704000.1	JQ704000.1; U2e2a1a1; Asia_S_W_Europe; 45; A73G; T146C; T152C	16572	Asia_S_W_Europe	U2e2a1a1	U	U2	45
2199	JQ704001.1	JQ704001.1; U5a1d2a; Asia_W_Europe_C; 34; A73G; T195C; A263G	16572	Asia_W_Europe_C	U5a1d2a	U	U5	34
2200	JQ704002.1	JQ704002.1; H1; Asia_W_Europe; 12; A263G; C309CCT; T310C	16570	Asia_W_Europe	H1	H	H	12
2201	JQ704003.1	JQ704003.1; H1b1; Asia_W_Europe; 16; A263G; C309CCCT; T310C	16569	Asia_W_Europe	H1b1	H	H	16
2202	JQ704004.1	JQ704004.1; K2b1a1; Asia_W; 35; A73G; T146C; A263G	16569	Asia_W	K2b1a1	K	K	35
2203	JQ704005.1	JQ704005.1; V; Europe_S; 15; T72C; A263G; C309CCT	16570	Europe_S	V	V	V	15
2204	JQ704006.1	JQ704006.1; H3a1; Asia_W_Europe; 14; T152C; A263G; C309CCT	16570	Asia_W_Europe	H3a1	H	H	14
2205	JQ704007.1	JQ704007.1; V3c; Europe_S; 19; T72C; A73G; A263G	16570	Europe_S	V3c	V	V	19
2206	JQ704008.1	JQ704008.1; U6b2; Asia_SW_Africa_N; 26; A73G; A263G; C315CC	16567	Asia_SW_Africa_N	U6b2	U	U6	26
2207	JQ704009.1	JQ704009.1; U2e2a1b; Asia_S_W_Europe; 47; A73G; T152C; A200G	16571	Asia_S_W_Europe	U2e2a1b	U	U2	47
2208	JQ704010.1	JQ704010.1; H1au1b; Asia_W_Europe; 15; A263G; C315CC; A750G	16569	Asia_W_Europe	H1au1b	H	H	15
2209	JQ704011.1	JQ704011.1; U8a1a1a; Asia_W_Europe ; 34; A73G; A263G; T282C	16572	Asia_W_Europe	U8a1a1a	U	U8	34
2210	JQ704012.1	JQ704012.1; H5a1; Asia_W_Europe; 14; C150T; A263G; C315CC	16567	Asia_W_Europe	H5a1	H	H	14
2211	JQ704013.1	JQ704013.1; I1a1a; Asia_W_SW; 44; A73G; T199C; G203A	16573	Asia_W_SW	I1a1a	I	I	44
2212	JQ704014.1	JQ704014.1; V16; Europe_S; 17; T72C; A263G; C309CCT	16570	Europe_S	V16	V	V	17
2213	JQ704015.1	JQ704015.1; V3a; Europe_S; 18; T72C; T195C; A263G	16570	Europe_S	V3a	V	V	18
2214	JQ704016.1	JQ704016.1; V; Europe_S; 19; C41T; T72C; A263G	16570	Europe_S	V	V	V	19

Supplementary Table S4 Human mitochondrial database (hMITO DB v1.0) metadata[a]

Row	Name (accession no.)	Description	Size	Geo_Region	Haplogroup	Macro_ Haplo	Macro_ Haplo2	Total Variants
2215	JQ704017.1	JQ704017.1; K1a2b; Asia_W; 36; A73G; T152C; A263G	16573	Asia_W	K1a2b	K	K	36
2216	JQ704018.1	JQ704018.1; I1b; Asia_W_SW; 38; A73G; T199C; T204C	16573	Asia_W_SW	I1b	I	I	38
2217	JQ704019.1	JQ704019.1; V+@16298; Europe_S; 15; T72C; A263G; C315CC	16569	Europe_S	V+@16298	V	V	15
2218	JQ704020.1	JQ704020.1; T2c1d1; Asia_W; 46; A73G; C114T; T146C	16570	Asia_W	T2c1d1	T	T	46
2219	JQ704021.1	JQ704021.1; U4b1b1; Asia_N_Europe_N; 33; A73G; T146C; T152C	16569	Asia_N_Europe_N	U4b1b1	U	U4	33
2220	JQ704022.1	JQ704022.1; U5a1a1c; Asia_W_Europe_C; 29; A73G; A263G; C315CC	16569	Asia_W_Europe_C	U5a1a1c	U	U5	29
2221	JQ704023.1	JQ704023.1; V15a; Europe_S; 17; T72C; A263G; C309CCT	16570	Europe_S	V15a	V	V	17
2222	JQ704024.1	JQ704024.1; H44a; Asia_W_Europe; 11; A263G; C315CC; A750G	16569	Asia_W_Europe	H44a	H	H	11
2223	JQ704025.1	JQ704025.1; H2a1k; Asia_W_Europe; 12; C41T; A263G; C309CCT	16570	Asia_W_Europe	H2a1k	H	H	12
2224	JQ704026.1	JQ704026.1; U5a1h; Asia_W_Europe_C; 33; A73G; C150T; A263G	16569	Asia_W_Europe_C	U5a1h	U	U5	33
2225	JQ704027.1	JQ704027.1; H59a; Asia_W_Europe; 11; A263G; C315CC; A750G	16569	Asia_W_Europe	H59a	H	H	11
2226	JQ704028.1	JQ704028.1; U5a1b1c2; Asia_W_Europe_C; 29; A73G; A263G; C315CC	16569	Asia_W_Europe_C	U5a1b1c2	U	U5	29
2227	JQ704029.1	JQ704029.1; U5b2a2b1; Asia_W_Europe_C; 32; A73G; C150T; T154C	16569	Asia_W_Europe_C	U5b2a2b1	U	U5	32
2228	JQ704030.1	JQ704030.1; U6b1a; Asia_SW_Africa_N; 27; A73G; A263G; C309CCT	16570	Asia_SW_Africa_N	U6b1a	U	U6	27
2229	JQ704031.1	JQ704031.1; V7; Europe_S; 17; T72A; A93G; A263G	16570	Europe_S	V7	V	V	17
2230	JQ704032.1	JQ704032.1; H1ae2; Asia_W_Europe; 14; A263G; C309CCT; T310C	16570	Asia_W_Europe	H1ae2	H	H	14
2231	JQ704033.1	JQ704033.1; J1c2; Asia_W; 32; A73G; G185A; A188G	16569	Asia_W	J1c2	J	J	32
2232	JQ704034.1	JQ704034.1; U1a1c1b; Asia_W; 38; A73G; A263G; C285T	16570	Asia_W	U1a1c1b	U	U1	38
2233	JQ704035.1	JQ704035.1; U5a1b1; Asia_W_Europe_C; 27; A73G; A263G; C315CC	16569	Asia_W_Europe_C	U5a1b1	U	U5	27
2234	JQ704036.1	JQ704036.1; V3b; Europe_S; 18; T72C; A263G; C309CCT	16570	Europe_S	V3b	V	V	18
2235	JQ704037.1	JQ704037.1; V16; Europe_S; 17; T72C; A263G; C315CC	16569	Europe_S	V16	V	V	17
2236	JQ704038.1	JQ704038.1; H1f1; Asia_W_Europe; 14; A263G; C315CC; A385G	16569	Asia_W_Europe	H1f1	H	H	14
2237	JQ704039.1	JQ704039.1; H4a1a5; Asia_W_Europe; 18; A263G; C315CC; CA522d	16567	Asia_W_Europe	H4a1a5	H	H	18
2238	JQ704040.1	JQ704040.1; C1c6; Asia_NE_America_N_S; 47; A73G; T195C; A249d	16567	Asia_NE_America_N_S	C1c6	C	C	47
2239	JQ704041.1	JQ704041.1; J2a1; Asia_W; 34; A73G; C150T; T152C	16570	Asia_W	J2a1	J	J	34
2240	JQ704042.1	JQ704042.1; U2e1; Asia_S_W_Europe; 38; A73G; T152C; T217C	16575	Asia_S_W_Europe	U2e1	U	U2	38
2241	JQ704043.1	JQ704043.1; U5b1c2b; Asia_W_Europe_C; 31; A73G; C150T; A263G	16569	Asia_W_Europe_C	U5b1c2b	U	U5	31
2242	JQ704044.1	JQ704044.1; U5a2b1b; Asia_W_Europe_C; 27; A73G; A263G; C315CC	16570	Asia_W_Europe_C	U5a2b1b	U	U5	27
2243	JQ704045.1	JQ704045.1; U5a1b+16362; Asia_W_Europe_C; 26; A73G; A263G; C315CC	16569	Asia_W_Europe_C	U5a1b+16362	U	U5	26
2244	JQ704046.1	JQ704046.1; U5a1b1; Asia_W_Europe_C; 28; A73G; A263G; C315CC	16569	Asia_W_Europe_C	U5a1b1	U	U5	28
2245	JQ704047.1	JQ704047.1; V; Europe_S; 16; T72C; A263G; C309CCT	16570	Europe_S	V	V	V	16
2246	JQ704048.1	JQ704048.1; H3+152; Asia_W_Europe; 12; T152C; A263G; C315CC	16571	Asia_W_Europe	H3+152	H	H	12
2247	JQ704049.1	JQ704049.1; M1b1a; Africa_E_Nile Valley; 44; A73G; T195C; A200G	16569	Africa_E_Nile Valley	M1b1a	M	M1	44
2248	JQ704050.1	JQ704050.1; H1a3a; Asia_W_Europe; 14; A73G; A263G; C309CCT	16570	Asia_W_Europe	H1a3a	H	H	14
2249	JQ704051.1	JQ704051.1; J1c5c1; Asia_W; 35; A73G; G185A; G228A	16567	Asia_W	J1c5c1	J	J	35
2250	JQ704052.1	JQ704052.1; U4a2; Asia_N_Europe_N; 31; A73G; T195C; A263G	16572	Asia_N_Europe_N	U4a2	U	U4	31
2251	JQ704053.1	JQ704053.1; U5b1h; Asia_W_Europe_C; 28; A73G; C150T; A263G	16569	Asia_W_Europe_C	U5b1h	U	U5	28
2252	JQ704054.1	JQ704054.1; U5b2c1; Asia_W_Europe_C; 35; A73G; C150T; G185A	16575	Asia_W_Europe_C	U5b2c1	U	U5	35
2253	JQ704055.1	JQ704055.1; T2i1; Asia_W; 36; A73G; A263G; C309CCT	16570	Asia_W	T2i1	T	T	36
2254	JQ704056.1	JQ704056.1; K1a1b1e; Asia_W; 35; A73G; A263G; C315CC	16569	Asia_W	K1a1b1e	K	K	35
2255	JQ704057.1	JQ704057.1; K1b1a1a; Asia_W; 44; A73G; T152C; T199C	16571	Asia_W	K1b1a1a	K	K	44

Supplementary Table S4 Human mitochondrial database (hMITO DB v1.0) metadata[a]

Row	Name (accession no.)	Description	Size	Geo_Region	Haplogroup	Macro_ Haplo	Macro_ Haplo2	Total Variants
2256	JQ704058.1	JQ704058.1; H2a1; Asia_W_Europe; 9; A263G; C309CCT; T310C	16570	Asia_W_Europe	H2a1	H	H	9
2257	JQ704059.1	JQ704059.1; H5a1b; Asia_W_Europe; 15; A263G; C309CCT; T310C	16568	Asia_W_Europe	H5a1b	H	H	15
2258	JQ704060.1	JQ704060.1; H5a1j; Asia_W_Europe; 15; A263G; C309CCT; T310C	16570	Asia_W_Europe	H5a1j	H	H	15
2259	JQ704061.1	JQ704061.1; U2e1a1; Asia_S_W_Europe; 42; A73G; T152C; T217C	16572	Asia_S_W_Europe	U2e1a1	U	U2	42
2260	JQ704062.1	JQ704062.1; K1a16; Asia_W; 33; A73G; T195C; A263G	16573	Asia_W	K1a16	K	K	33
2261	JQ704063.1	JQ704063.1; V2b2; Europe_S; 18; T72C; A263G; C309CCT	16570	Europe_S	V2b2	V	V	18
2262	JQ704064.1	JQ704064.1; K2b1b; Asia_W; 38; A73G; T146C; A263G	16570	Asia_W	K2b1b	K	K	38
2263	JQ704065.1	JQ704065.1; U5b1b1a; Asia_W_Europe_C; 32; A73G; C150T; A263G	16570	Asia_W_Europe_C	U5b1b1a	U	U5	32
2264	JQ704066.1	JQ704066.1; H1at1a; Asia_W_Europe; 12; A263G; C315CC; A750G	16569	Asia_W_Europe	H1at1a	H	H	12
2265	JQ704067.1	JQ704067.1; U5a2c2; Asia_W_Europe_C; 26; A73G; A263G; C315CC	16569	Asia_W_Europe_C	U5a2c2	U	U5	26
2266	JQ704068.1	JQ704068.1; N1b1a2b; Africa_NE_Asia_W; 40; A73G; T152C; A263G	16569	Africa_NE_Asia_W	N1b1a2b	N	N1	40
2267	JQ704069.1	JQ704069.1; U4a1a; Asia_N_Europe_N; 34; A73G; T152C; T195C	16573	Asia_N_Europe_N	U4a1a	U	U4	34
2268	JQ704070.1	JQ704070.1; K2b1; Asia_W; 40; A73G; T146C; T152C	16572	Asia_W	K2b1	K	K	40
2269	JQ704071.1	JQ704071.1; H5b1; Asia_W_Europe; 15; T146C; T195C; A263G	16570	Asia_W_Europe	H5b1	H	H	15
2270	JQ704072.1	JQ704072.1; H13a1a1; Asia_W_Europe; 16; A263G; C309CCT; T310C	16571	Asia_W_Europe	H13a1a1	H	H	16
2271	JQ704073.1	JQ704073.1; N1a3a1a; Africa_NE_Asia_W; 41; A73G; T152C; A189G	16567	Africa_NE_Asia_W	N1a3a1a	N	N1	41
2272	JQ704074.1	JQ704074.1; U5a2a1+152; Asia_W_Europe_C; 29; A73G; T152C; A263G	16570	Asia_W_Europe_C	U5a2a1+152	U	U5	29
2273	JQ704075.1	JQ704075.1; B4b1a2; Asia_SE_E_America_N_S; 32; A73G; G207A; A263G	16560	Asia_SE_E_America_N_S	B4b1a2	B	B	32
2274	JQ704076.1	JQ704076.1; K2a5a; Asia_W; 35; A73G; T146C; T152C	16571	Asia_W	K2a5a	K	K	35
2275	JQ704077.1	JQ704077.1; I1f; Asia_W_SW; 41; A73G; T199C; T204C	16571	Asia_W_SW	I1f	I	I	41
2276	JQ704078.1	JQ704078.1; K1a4a1; Asia_W; 40; A73G; A263G; C315CC	16569	Asia_W	K1a4a1	K	K	40
2277	JQ704079.1	JQ704079.1; H3; Asia_W_Europe; 13; A263G; C315CC; A750G	16569	Asia_W_Europe	H3	H	H	13
2278	JQ704080.1	JQ704080.1; H1c6; Asia_W_Europe; 11; A263G; C309CCCT; T310C	16571	Asia_W_Europe	H1c6	H	H	11
2279	JQ704081.1	JQ704081.1; H11b1; Asia_W_Europe; 16; T152C; T195C; A263G	16569	Asia_W_Europe	H11b1	H	H	16
2280	JQ704082.1	JQ704082.1; H10e; Asia_W_Europe; 13; A263G; C309CCCT; T310C	16571	Asia_W_Europe	H10e	H	H	13
2281	JQ704083.1	JQ704083.1; H17; Asia_W_Europe; 13; C256T; A263G; C315CC	16569	Asia_W_Europe	H17	H	H	13
2282	JQ704084.1	JQ704084.1; U5a1a1+152; Asia_W_Europe_C; 29; A73G; T152C; A263G	16570	Asia_W_Europe_C	U5a1a1+152	U	U5	29
2283	JQ704085.1	JQ704085.1; H1+16189; Asia_W_Europe; 12; G207A; A263G; C309CCCT	16571	Asia_W_Europe	H1+16189	H	H	12
2284	JQ704086.1	JQ704086.1; H1b1+16362; Asia_W_Europe; 15; A263G; C315CC; CA522d	16567	Asia_W_Europe	H1b1+16362	H	H	15
2285	JQ704087.1	JQ704087.1; V+@16298; Europe_S; 13; T72C; A263G; C315CC	16569	Europe_S	V+@16298	V	V	13
2286	JQ704088.1	JQ704088.1; H; Asia_W_Europe; 10; A263G; C309CCT; T310C	16570	Asia_W_Europe	H	H	H	10
2287	JQ704089.1	JQ704089.1; H1br; Asia_W_Europe; 12; A263G; C315CC; A750G	16569	Asia_W_Europe	H1br	H	H	12
2288	JQ704090.1	JQ704090.1; H1j5; Asia_W_Europe; 15; A263G; C309CCCT; T310C	16571	Asia_W_Europe	H1j5	H	H	15
2289	JQ704091.1	JQ704091.1; H1a; Asia_W_Europe; 14; A73G; A263G; C309CCT	16570	Asia_W_Europe	H1a	H	H	14
2290	JQ704092.1	JQ704092.1; K1a4a1; Asia_W; 40; A73G; A200G; A235G	16571	Asia_W	K1a4a1	K	K	40
2291	JQ704093.1	JQ704093.1; H14a; Asia_W_Europe; 13; A263G; C309CCT; T310C	16570	Asia_W_Europe	H14a	H	H	13
2292	JQ704094.1	JQ704094.1; L2c2b1a; Africa_W_C; 65; A73G; A93G; T146C	16567	Africa_W_C	L2c2b1a	L2	L2	65
2293	JQ704095.1	JQ704095.1; H1n1b; Asia_W_Europe; 17; T146C; A263G; C309CCT	16574	Asia_W_Europe	H1n1b	H	H	17
2294	JQ704096.1	JQ704096.1; K1a1b1d; Asia_W; 36; A73G; A263G; C315CC	16569	Asia_W	K1a1b1d	K	K	36
2295	JQ704097.1	JQ704097.1; J1b1a1b; Asia_W; 39; A73G; C242T; A263G	16569	Asia_W	J1b1a1b	J	J	39
2296	JQ704098.1	JQ704098.1; U4b1a2a; Asia_N_Europe_N; 38; A73G; T195C; A263G	16572	Asia_N_Europe_N	U4b1a2a	U	U4	38

Supplementary Table S4 Human mitochondrial database (hMITO DB v1.0) metadata[a]

Row	Name (accession no.)	Description	Size	Geo_Region	Haplogroup	Macro_ Haplo	Macro_ Haplo2	Total Variants
2297	JQ704099.1	JQ704099.1; U6a3a2a; Asia_SW_Africa_N; 31; A73G; G185A; A263G	16569	Asia_SW_Africa_N	U6a3a2a	U	U6	31
2298	JQ704100.1	JQ704100.1; U7b2; Asia_W; 34; A73G; T152C; A263G	16567	Asia_W	U7b2	U	U7	34
2299	JQ704101.1	JQ704101.1; H11a; Asia_W_Europe; 17; T195C; G228A; A263G	16571	Asia_W_Europe	H11a	H	H	17
2300	JQ704102.1	JQ704102.1; U2e3a; Asia_S_W_Europe; 44; A73G; T152C; T217C	16572	Asia_S_W_Europe	U2e3a	U	U2	44
2301	JQ704103.1	JQ704103.1; J1c7a; Asia_W; 35; A73G; G185A; G228A	16569	Asia_W	J1c7a	J	J	35
2302	JQ704104.1	JQ704104.1; V7b; Europe_S; 19; T72C; A93G; A263G	16570	Europe_S	V7b	V	V	19
2303	JQ704105.1	JQ704105.1; H3b1b1a; Asia_W_Europe; 19; A153G; A263G; C309CCT	16570	Asia_W_Europe	H3b1b1a	H	H	19
2304	JQ704106.1	JQ704106.1; H1a3; Asia_W_Europe; 14; A73G; A263G; C315CC	16569	Asia_W_Europe	H1a3	H	H	14
2305	JQ704107.1	JQ704107.1; M3a1+204; Asia_S; 31; A73G; T204C; A235G	16570	Asia_S	M3a1+204	M	M3	31
2306	JQ704108.1	JQ704108.1; H10e1a; Asia_W_Europe; 14; A263G; C309CCT; T310C	16570	Asia_W_Europe	H10e1a	H	H	14
2307	JQ704109.1	JQ704109.1; K1a4a1; Asia_W; 35; A73G; A263G; C309CCT	16572	Asia_W	K1a4a1	K	K	35
2308	JQ704110.1	JQ704110.1; H26c; Asia_W_Europe; 12; T146C; A263G; C315CC	16569	Asia_W_Europe	H26c	H	H	12
2309	JQ704111.1	JQ704111.1; H13b1a; Asia_W_Europe; 14; A200G; A263G; C315CC	16569	Asia_W_Europe	H13b1a	H	H	14
2310	JQ704112.1	JQ704112.1; U5b1; Asia_W_Europe_C; 29; A73G; T146C; C150T	16568	Asia_W_Europe_C	U5b1	U	U5	29
2311	JQ704113.1	JQ704113.1; H13a1a1; Asia_W_Europe; 16; T152C; A263G; C309CCT	16570	Asia_W_Europe	H13a1a1	H	H	16
2312	JQ704114.1	JQ704114.1; H3h7; Asia_W_Europe; 13; A93G; A263G; C315CC	16569	Asia_W_Europe	H3h7	H	H	13
2313	JQ704115.1	JQ704115.1; H3h; Asia_W_Europe; 12; A263G; C315CC; A750G	16569	Asia_W_Europe	H3h	H	H	12
2314	JQ704116.1	JQ704116.1; H6a1b3a; Asia_W_Europe; 21; T204C; T239C; A263G	16571	Asia_W_Europe	H6a1b3a	H	H	21
2315	JQ704117.1	JQ704117.1; H1a2; Asia_W_Europe; 13; A73G; A263G; C309CCT	16570	Asia_W_Europe	H1a2	H	H	13
2316	JQ704118.1	JQ704118.1; J1b1a1a; Asia_W; 40; A73G; C242T; A263G	16569	Asia_W	J1b1a1a	J	J	40
2317	JQ704119.1	JQ704119.1; H1c3a; Asia_W_Europe; 16; T146C; A257G; A263G	16570	Asia_W_Europe	H1c3a	H	H	16
2318	JQ704120.1	JQ704120.1; H3s; Asia_W_Europe; 12; C194T; A263G; C315CC	16569	Asia_W_Europe	H3s	H	H	12
2319	JQ704121.1	JQ704121.1; U4c1; Asia_N_Europe_N; 37; A73G; A189G; T195C	16569	Asia_N_Europe_N	U4c1	U	U4	37
2320	JQ704122.1	JQ704122.1; H1e1a; Asia_W_Europe; 16; T55TT; T57C; T59C	16572	Asia_W_Europe	H1e1a	H	H	16
2321	JQ704123.1	JQ704123.1; H2a1; Asia_W_Europe; 9; A263G; C315CC; A750G	16569	Asia_W_Europe	H2a1	H	H	9
2322	JQ704124.1	JQ704124.1; HV16; Asia_W; 14; A263G; C309CCT; T310C	16570	Asia_W	HV16	HV	HV	14
2323	JQ704125.1	JQ704125.1; H26a1; Asia_W_Europe; 13; T152C; A263G; C315CC	16571	Asia_W_Europe	H26a1	H	H	13
2324	JQ704126.1	JQ704126.1; V2c; Europe_S; 16; T72C; A263G; C315CC	16569	Europe_S	V2c	V	V	16
2325	JQ704127.1	JQ704127.1; H6a1a3; Asia_W_Europe; 18; T239C; A263G; C309CCCT	16569	Asia_W_Europe	H6a1a3	H	H	18
2326	JQ704128.1	JQ704128.1; H1e2a; Asia_W_Europe; 14; A263G; C309CCT; T310C	16570	Asia_W_Europe	H1e2a	H	H	14
2327	JQ704129.1	JQ704129.1; T2f; Asia_W; 37; A73G; T152C; A263G	16563	Asia_W	T2f	T	T	37
2328	JQ704130.1	JQ704130.1; U3b2; Africa_NE_Asia_W; 30; A73G; C150T; T195C	16567	Africa_NE_Asia_W	U3b2	U	U3	30
2329	JQ704131.1	JQ704131.1; H3; Asia_W_Europe; 13; T72G; A263G; C309CCT	16570	Asia_W_Europe	H3	H	H	13
2330	JQ704132.1	JQ704132.1; H42; Asia_W_Europe; 11; A263G; C315CC; A750G	16569	Asia_W_Europe	H42	H	H	11
2331	JQ704133.1	JQ704133.1; H3p; Asia_W_Europe; 16; T146C; A263G; C309CCT	16570	Asia_W_Europe	H3p	H	H	16
2332	JQ704134.1	JQ704134.1; H1c1; Asia_W_Europe; 13; A263G; C309CCT; T310C	16570	Asia_W_Europe	H1c1	H	H	13
2333	JQ704135.1	JQ704135.1; H3aj; Asia_W_Europe; 12; A263G; C315CC; C573CC	16570	Asia_W_Europe	H3aj	H	H	12
2334	JQ704136.1	JQ704136.1; H3b2; Asia_W_Europe; 13; A263G; C309CCT; T310C	16570	Asia_W_Europe	H3b2	H	H	13
2335	JQ704137.1	JQ704137.1; H78; Asia_W_Europe; 15; A263G; C309CCCT; T310C	16571	Asia_W_Europe	H78	H	H	15
2336	JQ704138.1	JQ704138.1; H1bd; Asia_W_Europe; 13; T204C; A263G; C315CC	16569	Asia_W_Europe	H1bd	H	H	13
2337	JQ704139.1	JQ704139.1; H1b3; Asia_W_Europe; 16; A263G; C309CCCT; T310C	16571	Asia_W_Europe	H1b3	H	H	16

Supplementary Table S4 Human mitochondrial database (hMITO DB v1.0) metadata[a]

Row	Name (accession no.)	Description	Size	Geo_Region	Haplogroup	Macro_ Haplo	Macro_ Haplo2	Total Variants
2338	JQ704140.1	JQ704140.1; H2a2b2; Asia_W_Europe; 8; A263G; C309CCT; T310C	16570	Asia_W_Europe	H2a2b2	H	H	8
2339	JQ704141.1	JQ704141.1; H1a5; Asia_W_Europe; 12; A73G; A263G; C315CC	16569	Asia_W_Europe	H1a5	H	H	12
2340	JQ704142.1	JQ704142.1; HV0g; Asia_W; 17; T72C; T195C; A263G	16573	Asia_W	HV0g	HV	HV	17
2341	JQ704143.1	JQ704143.1; HV0c; Asia_W; 14; T72C; T195C; A263G	16570	Asia_W	HV0c	HV	HV	14
2342	JQ704144.1	JQ704144.1; H51a; Asia_W_Europe; 13; A263G; C309CCT; T310C	16570	Asia_W_Europe	H51a	H	H	13
2343	JQ704145.1	JQ704145.1; H6a1a; Asia_W_Europe; 18; T146C; T239C; A263G	16570	Asia_W_Europe	H6a1a	H	H	18
2344	JQ704146.1	JQ704146.1; H5a1; Asia_W_Europe; 13; A263G; C315CC; C456T	16567	Asia_W_Europe	H5a1	H	H	13
2345	JQ704147.1	JQ704147.1; H13b1b; Asia_W_Europe; 21; A200G; A263G; C309CCT	16568	Asia_W_Europe	H13b1b	H	H	21
2346	JQ704148.1	JQ704148.1; H5a2; Asia_W_Europe; 14; A263G; C309CCT; T310C	16570	Asia_W_Europe	H5a2	H	H	14
2347	JQ704149.1	JQ704149.1; H63; Asia_W_Europe; 10; A263G; C315CC; A750G	16569	Asia_W_Europe	H63	H	H	10
2348	JQ704150.1	JQ704150.1; H5a7; Asia_W_Europe; 14; A263G; C309CCT; T310C	16570	Asia_W_Europe	H5a7	H	H	14
2349	JQ704151.1	JQ704151.1; H67; Asia_W_Europe; 13; A263G; C309CCT; T310C	16570	Asia_W_Europe	H67	H	H	13
2350	JQ704152.1	JQ704152.1; H28a; Asia_W_Europe; 13; C186A; A263G; C315CC	16569	Asia_W_Europe	H28a	H	H	13
2351	JQ704153.1	JQ704153.1; U1a1b; Asia_W; 35; A73G; T152C; A263G	16570	Asia_W	U1a1b	U	U1	35
2352	JQ704154.1	JQ704154.1; H1aw; Asia_W_Europe; 14; A263G; C315CC; T460C	16569	Asia_W_Europe	H1aw	H	H	14
2353	JQ704155.1	JQ704155.1; U4b1b1b; Asia_N_Europe_N; 36; A73G; T152C; T195C	16569	Asia_N_Europe_N	U4b1b1b	U	U4	36
2354	JQ704156.1	JQ704156.1; H1be; Asia_W_Europe; 13; A263G; C309CCT; T310C	16570	Asia_W_Europe	H1be	H	H	13
2355	JQ704157.1	JQ704157.1; H1y; Asia_W_Europe; 12; A263G; C309CCT; T310C	16570	Asia_W_Europe	H1y	H	H	12
2356	JQ704158.1	JQ704158.1; H1as1a; Asia_W_Europe; 16; C150T; A263G; C309CCT	16570	Asia_W_Europe	H1as1a	H	H	16
2357	JQ704159.1	JQ704159.1; H1b1c; Asia_W_Europe; 16; A263G; C315CC; C483T	16567	Asia_W_Europe	H1b1c	H	H	16
2358	JQ704160.1	JQ704160.1; W3a1d; Asia_W; 40; A73G; A189G; C194T	16570	Asia_W	W3a1d	W	W	40
2359	JQ704161.1	JQ704161.1; H14a2a; Asia_W_Europe; 17; A263G; C309CCT; T310C	16570	Asia_W_Europe	H14a2a	H	H	17
2360	JQ704162.1	JQ704162.1; H1bc; Asia_W_Europe; 11; T152C; A263G; C315CC	16569	Asia_W_Europe	H1bc	H	H	11
2361	JQ704163.1	JQ704163.1; H1af1a; Asia_W_Europe; 13; T152C; A263G; C315CC	16569	Asia_W_Europe	H1af1a	H	H	13
2362	JQ704164.1	JQ704164.1; H11a+152; Asia_W_Europe; 16; T152C; T195C; A263G	16570	Asia_W_Europe	H11a+152	H	H	16
2363	JQ704165.1	JQ704165.1; H26a1; Asia_W_Europe; 18; T152C; A263G; C309CCCT	16571	Asia_W_Europe	H26a1	H	H	18
2364	JQ704166.1	JQ704166.1; H11a2; Asia_W_Europe; 16; T152C; T195C; A263G	16569	Asia_W_Europe	H11a2	H	H	16
2365	JQ704167.1	JQ704167.1; H6a1b2; Asia_W_Europe; 17; T239C; A263G; C315CC	16569	Asia_W_Europe	H6a1b2	H	H	17
2366	JQ704168.1	JQ704168.1; H3k1a; Asia_W_Europe; 15; T152C; A263G; C315CC	16569	Asia_W_Europe	H3k1a	H	H	15
2367	JQ704169.1	JQ704169.1; H17; Asia_W_Europe; 13; A263G; C309CCT; T310C	16570	Asia_W_Europe	H17	H	H	13
2368	JQ704170.1	JQ704170.1; H1ag1b; Asia_W_Europe; 14; A263G; C309CCT; T310C	16570	Asia_W_Europe	H1ag1b	H	H	14
2369	JQ704171.1	JQ704171.1; H7a2; Asia_W_Europe; 14; A263G; C309CCT; T310C	16570	Asia_W_Europe	H7a2	H	H	14
2370	JQ704172.1	JQ704172.1; H3c2b; Asia_W_Europe; 13; T195C; A263G; C315CC	16569	Asia_W_Europe	H3c2b	H	H	13
2371	JQ704173.1	JQ704173.1; H2a2a2; Asia_W_Europe; 7; T152C; A263G; C309CCT	16571	Asia_W_Europe	H2a2a2	H	H	7
2372	JQ704174.1	JQ704174.1; H6a1b4; Asia_W_Europe; 19; T239C; A263G; C309CCT	16570	Asia_W_Europe	H6a1b4	H	H	19
2373	JQ704175.1	JQ704175.1; H1c9a; Asia_W_Europe; 14; T152C; A263G; C315CC	16569	Asia_W_Europe	H1c9a	H	H	14
2374	JQ704176.1	JQ704176.1; H13a1a6; Asia_W_Europe; 14; G228A; A263G; C309CCCT	16571	Asia_W_Europe	H13a1a6	H	H	14
2375	JQ704177.1	JQ704177.1; H1ax1; Asia_W_Europe; 17; C150T; A263G; C309CCCT	16571	Asia_W_Europe	H1ax1	H	H	17
2376	JQ704178.1	JQ704178.1; H3z2; Asia_W_Europe; 15; A263G; T293C; C309CCT	16570	Asia_W_Europe	H3z2	H	H	15
2377	JQ704179.1	JQ704179.1; H6c; Asia_W_Europe; 13; T239C; A263G; C315CC	16569	Asia_W_Europe	H6c	H	H	13
2378	JQ704180.1	JQ704180.1; A2+(64); Asia_NE_America_N; 32; C64T; A73G; T146C	16568	Asia_NE_America_N	A2+(64)	A	A	32

Supplementary Table S4 Human mitochondrial database (hMITO DB v1.0) metadata[a]

Row	Name (accession no.)	Description	Size	Geo_Region	Haplogroup	Macro_ Haplo	Macro_ Haplo2	Total Variants
2379	JQ704181.1	JQ704181.1; U4c1; Asia_N_Europe_N; 35; A73G; T195C; A263G	16570	Asia_N_Europe_N	U4c1	U	U4	35
2380	JQ704182.1	JQ704182.1; H17; Asia_W_Europe; 12; A263G; C315CC; A750G	16569	Asia_W_Europe	H17	H	H	12
2381	JQ704183.1	JQ704183.1; H3ao1; Asia_W_Europe; 13; A263G; C315CC; A750G	16569	Asia_W_Europe	H3ao1	H	H	13
2382	JQ704184.1	JQ704184.1; HV15; Asia_W; 14; A263G; C309CCT; T310C	16570	Asia_W	HV15	HV	HV	14
2383	JQ704185.1	JQ704185.1; H11a; Asia_W_Europe; 16; T195C; A263G; C309CCT	16570	Asia_W_Europe	H11a	H	H	16
2384	JQ704186.1	JQ704186.1; H1bf; Asia_W_Europe; 16; T152C; A263G; C315CC	16569	Asia_W_Europe	H1bf	H	H	16
2385	JQ704187.1	JQ704187.1; K1a10a; Asia_W; 34; A73G; T195C; A263G	16573	Asia_W	K1a10a	K	K	34
2386	JQ704188.1	JQ704188.1; HV0a; Asia_W; 15; T72C; A263G; C295T	16570	Asia_W	HV0a	HV	HV	15
2387	JQ704189.1	JQ704189.1; H2a1e1; Asia_W_Europe; 11; A263G; C309CCT; T310C	16570	Asia_W_Europe	H2a1e1	H	H	11
2388	JQ704190.1	JQ704190.1; H3e; Asia_W_Europe; 13; A263G; C309CCCT; T310C	16571	Asia_W_Europe	H3e	H	H	13
2389	JQ704191.1	JQ704191.1; H1; Asia_W_Europe; 12; A263G; C315CC; A750G	16569	Asia_W_Europe	H1	H	H	12
2390	JQ704192.1	JQ704192.1; H3as; Asia_W_Europe; 10; A263G; C315CC; A750G	16569	Asia_W_Europe	H3as	H	H	10
2391	JQ704193.1	JQ704193.1; H1m; Asia_W_Europe; 17; C150T; A263G; C309CCT	16570	Asia_W_Europe	H1m	H	H	17
2392	JQ704194.1	JQ704194.1; H1t; Asia_W_Europe; 19; A95C; T195C; A263G	16570	Asia_W_Europe	H1t	H	H	19
2393	JQ704195.1	JQ704195.1; H6a1a6; Asia_W_Europe; 17; T239C; A263G; A288G	16572	Asia_W_Europe	H6a1a6	H	H	17
2394	JQ704196.1	JQ704196.1; H1ae; Asia_W_Europe; 17; A263G; C309CCT; T310C	16570	Asia_W_Europe	H1ae	H	H	17
2395	JQ704197.1	JQ704197.1; H3b4; Asia_W_Europe; 13; A263G; C309CCCT; T310C	16571	Asia_W_Europe	H3b4	H	H	13
2396	JQ704198.1	JQ704198.1; H2a1a1; Asia_W_Europe; 14; A263G; C315CC; A750G	16569	Asia_W_Europe	H2a1a1	H	H	14
2397	JQ704199.1	JQ704199.1; H1j4; Asia_W_Europe; 13; A263G; C309CCCT; T310C	16571	Asia_W_Europe	H1j4	H	H	13
2398	JQ704200.1	JQ704200.1; H1b1+16362; Asia_W_Europe; 14; C151T; A263G; C315CC	16567	Asia_W_Europe	H1b1+16362	H	H	14
2399	JQ704201.1	JQ704201.1; H56; Asia_W_Europe; 12; A263G; C309CCT; T310C	16570	Asia_W_Europe	H56	H	H	12
2400	JQ704202.1	JQ704202.1; H1n+146; Asia_W_Europe; 11; T146C; A263G; C315CC	16569	Asia_W_Europe	H1n+146	H	H	11
2401	JQ704203.1	JQ704203.1; H1+16189; Asia_W_Europe; 14; A263G; C309CCCT; T310C	16571	Asia_W_Europe	H1+16189	H	H	14
2402	JQ704204.1	JQ704204.1; H1a3; Asia_W_Europe; 14; A73G; A263G; C315CC	16569	Asia_W_Europe	H1a3	H	H	14
2403	JQ704205.1	JQ704205.1; H18b; Asia_W_Europe; 12; A263G; C315CC; A750G	16569	Asia_W_Europe	H18b	H	H	12
2404	JQ704206.1	JQ704206.1; H27; Asia_W_Europe; 19; T63C; C64T; G66A	16569	Asia_W_Europe	H27	H	H	19
2405	JQ704207.1	JQ704207.1; K1a1b1e; Asia_W; 37; A73G; C114T; T146C	16567	Asia_W	K1a1b1e	K	K	37
2406	JQ704208.1	JQ704208.1; H6a2a; Asia_W_Europe; 16; T152C; T239C; A263G	16570	Asia_W_Europe	H6a2a	H	H	16
2407	JQ704209.1	JQ704209.1; H10e; Asia_W_Europe; 12; A263G; C309CCCT; T310C	16571	Asia_W_Europe	H10e	H	H	12
2408	JQ704210.1	JQ704210.1; C1b11; Asia_NE_America_N_S; 46; T63d; A73G; C194T	16565	Asia_NE_America_N_S	C1b11	C	C	46
2409	JQ704211.1	JQ704211.1; J1c3; Asia_W; 33; A73G; C150T; G185A	16570	Asia_W	J1c3	J	J	33
2410	JQ704212.1	JQ704212.1; H1bi; Asia_W_Europe; 11; A263G; C309CCCT; T310C	16571	Asia_W_Europe	H1bi	H	H	11
2411	JQ704213.1	JQ704213.1; V1a; Europe_S; 19; A263G; C309CCCT; T310C	16571	Europe_S	V1a	V	V	19
2412	JQ704214.1	JQ704214.1; H2a5; Asia_W_Europe; 8; A263G; C309CCCT; T310C	16571	Asia_W_Europe	H2a5	H	H	8
2413	JQ704215.1	JQ704215.1; U4a1; Asia_N_Europe_N; 32; A73G; T152C; T195C	16569	Asia_N_Europe_N	U4a1	U	U4	32
2414	JQ704216.1	JQ704216.1; K1a1b1a; Asia_W; 38; A73G; C114T; A263G	16569	Asia_W	K1a1b1a	K	K	38
2415	JQ704217.1	JQ704217.1; HV10; Asia_W; 14; A263G; C309CCCT; T310C	16571	Asia_W	HV10	HV	HV	14
2416	JQ704218.1	JQ704218.1; H13a1a; Asia_W_Europe; 13; A263G; C309CCT; T310C	16572	Asia_W_Europe	H13a1a	H	H	13
2417	JQ704219.1	JQ704219.1; H1bs; Asia_W_Europe; 12; T152C; A263G; C309CCT	16570	Asia_W_Europe	H1bs	H	H	12
2418	JQ704220.1	JQ704220.1; H2a3a1; Asia_W_Europe; 12; T146C; A263G; C309CCT	16570	Asia_W_Europe	H2a3a1	H	H	12
2419	JQ704221.1	JQ704221.1; H15a1; Asia_W_Europe; 14; T55C; T57C; A263G	16571	Asia_W_Europe	H15a1	H	H	14

Supplementary Table S4 Human mitochondrial database (hMITO DB v1.0) metadata[a]

Row	Name (accession no.)	Description	Size	Geo_Region	Haplogroup	Macro_ Haplo	Macro_ Haplo2	Total Variants
2420	JQ704222.1	JQ704222.1; H3+16189; Asia_W_Europe; 15; A263G; C309CCT; T310C	16570	Asia_W_Europe	H3+16189	H	H	15
2421	JQ704223.1	JQ704223.1; H1a4; Asia_W_Europe; 13; A73G; A263G; C309CCT	16570	Asia_W_Europe	H1a4	H	H	13
2422	JQ704224.1	JQ704224.1; H2a2a1g; Asia_W_Europe; 3; C315CC; T14798C; T16189C	16569	Asia_W_Europe	H2a2a1g	H	H	3
2423	JQ704225.1	JQ704225.1; H5a1; Asia_W_Europe; 16; T195C; A240G; A263G	16567	Asia_W_Europe	H5a1	H	H	16
2424	JQ704226.1	JQ704226.1; H1+16278; Asia_W_Europe; 12; A93G; T146C; A263G	16569	Asia_W_Europe	H1+16278	H	H	12
2425	JQ704227.1	JQ704227.1; H27e; Asia_W_Europe; 13; A263G; C309CCT; T310C	16561	Asia_W_Europe	H27e	H	H	13
2426	JQ704228.1	JQ704228.1; K1a9; Asia_W; 33; A73G; T195C; A263G	16569	Asia_W	K1a9	K	K	33
2427	JQ704229.1	JQ704229.1; HV9a1; Asia_W; 17; T131C; T152C; A263G	16570	Asia_W	HV9a1	HV	HV	17
2428	JQ704230.1	JQ704230.1; H1+16239; Asia_W_Europe; 10; A263G; C315CC; A750G	16569	Asia_W_Europe	H1+16239	H	H	10
2429	JQ704231.1	JQ704231.1; H4a1a1a; Asia_W_Europe; 21; A73G; A263G; C309CCT	16568	Asia_W_Europe	H4a1a1a	H	H	21
2430	JQ704232.1	JQ704232.1; H3ao; Asia_W_Europe; 11; A263G; C315CC; A750G	16569	Asia_W_Europe	H3ao	H	H	11
2431	JQ704233.1	JQ704233.1; H5b; Asia_W_Europe; 15; T146C; A263G; C309CCT	16570	Asia_W_Europe	H5b	H	H	15
2432	JQ704234.1	JQ704234.1; H13a1a1b; Asia_W_Europe; 17; A214G; A263G; C309CCT	16570	Asia_W_Europe	H13a1a1b	H	H	17
2433	JQ704235.1	JQ704235.1; H1ao1; Asia_W_Europe; 16; A93G; T146C; A263G	16569	Asia_W_Europe	H1ao1	H	H	16
2434	JQ704236.1	JQ704236.1; H3c1; Asia_W_Europe; 14; A189G; A263G; C315CC	16569	Asia_W_Europe	H3c1	H	H	14
2435	JQ704237.1	JQ704237.1; H5a6a; Asia_W_Europe; 16; T152C; A263G; C315CC	16569	Asia_W_Europe	H5a6a	H	H	16
2436	JQ704238.1	JQ704238.1; H5b1; Asia_W_Europe; 15; T146C; T195C; A263G	16571	Asia_W_Europe	H5b1	H	H	15
2437	JQ704239.1	JQ704239.1; H6a1b2c; Asia_W_Europe; 21; T239C; A263G; C309CCCT	16573	Asia_W_Europe	H6a1b2c	H	H	21
2438	JQ704240.1	JQ704240.1; H1; Asia_W_Europe; 14; A93G; A263G; C315CC	16569	Asia_W_Europe	H1	H	H	14
2439	JQ704241.1	JQ704241.1; H26a1; Asia_W_Europe; 12; A263G; C315CC; A750G	16569	Asia_W_Europe	H26a1	H	H	12
2440	JQ704242.1	JQ704242.1; H1c12; Asia_W_Europe; 12; A263G; C315CC; T477C	16569	Asia_W_Europe	H1c12	H	H	12
2441	JQ704243.1	JQ704243.1; H1a3c1; Asia_W_Europe; 16; A73G; A263G; C315CC	16569	Asia_W_Europe	H1a3c1	H	H	16
2442	JQ704244.1	JQ704244.1; H3a1; Asia_W_Europe; 17; T152C; A263G; C309CCT	16572	Asia_W_Europe	H3a1	H	H	17
2443	JQ704245.1	JQ704245.1; H1a3; Asia_W_Europe; 14; A73G; A263G; C315CC	16569	Asia_W_Europe	H1a3	H	H	14
2444	JQ704246.1	JQ704246.1; H5a3a; Asia_W_Europe; 16; A263G; C309CCT; T310C	16570	Asia_W_Europe	H5a3a	H	H	16
2445	JQ704247.1	JQ704247.1; U5a2a1; Asia_W_Europe_C; 32; A73G; A263G; C309CCT	16570	Asia_W_Europe_C	U5a2a1	U	U5	32
2446	JQ704248.1	JQ704248.1; H5a1; Asia_W_Europe; 14; T146C; A263G; C315CC	16567	Asia_W_Europe	H5a1	H	H	14
2447	JQ704249.1	JQ704249.1; T1a1; Asia_W; 35; A73G; T152C; T195C	16570	Asia_W	T1a1	T	T	35
2448	JQ704250.1	JQ704250.1; H1e5a; Asia_W_Europe; 17; T152C; A263G; C315CC	16569	Asia_W_Europe	H1e5a	H	H	17
2449	JQ704251.1	JQ704251.1; H85; Asia_W_Europe; 13; A263G; C309CCCT; T310C	16571	Asia_W_Europe	H85	H	H	13
2450	JQ704252.1	JQ704252.1; H3ap; Asia_W_Europe; 13; A263G; C309CCT; T310C	16570	Asia_W_Europe	H3ap	H	H	13
2451	JQ704253.1	JQ704253.1; H3a1; Asia_W_Europe; 16; T152C; A263G; C309CCT	16570	Asia_W_Europe	H3a1	H	H	16
2452	JQ704254.1	JQ704254.1; H14b4; Asia_W_Europe; 15; A263G; C309CCT; T310C	16570	Asia_W_Europe	H14b4	H	H	15
2453	JQ704255.1	JQ704255.1; HV0+195; Asia_W; 17; T72C; T195C; A263G	16570	Asia_W	HV0+195	HV	HV	17
2454	JQ704256.1	JQ704256.1; H82; Asia_W_Europe; 12; T195C; A263G; C309CCT	16570	Asia_W_Europe	H82	H	H	12
2455	JQ704257.1	JQ704257.1; H1a1; Asia_W_Europe; 15; A73G; A263G; C315CC	16569	Asia_W_Europe	H1a1	H	H	15
2456	JQ704258.1	JQ704258.1; H1as1a; Asia_W_Europe; 16; A73G; C150T; A263G	16570	Asia_W_Europe	H1as1a	H	H	16
2457	JQ704259.1	JQ704259.1; H1c6; Asia_W_Europe; 12; A263G; C315CC; T477C	16569	Asia_W_Europe	H1c6	H	H	12
2458	JQ704260.1	JQ704260.1; H24a; Asia_W_Europe; 13; A263G; C315CC; A750G	16569	Asia_W_Europe	H24a	H	H	13
2459	JQ704261.1	JQ704261.1; H94; Asia_W_Europe; 11; A263G; C309CCT; T310C	16570	Asia_W_Europe	H94	H	H	11
2460	JQ704262.1	JQ704262.1; H2a2b3; Asia_W_Europe; 7; A263G; C309CCT; T310C	16570	Asia_W_Europe	H2a2b3	H	H	7

Supplementary Table S4 Human mitochondrial database (hMITO DB v1.0) metadata[a]

Row	Name (accession no.)	Description	Size	Geo_Region	Haplogroup	Macro_ Haplo	Macro_ Haplo2	Total Variants
2461	JQ704263.1	JQ704263.1; H1+16239; Asia_W_Europe; 14; T152C; A263G; C309CCT	16570	Asia_W_Europe	H1+16239	H	H	14
2462	JQ704264.1	JQ704264.1; H5b2; Asia_W_Europe; 15; A263G; C309CCT; T310C	16570	Asia_W_Europe	H5b2	H	H	15
2463	JQ704265.1	JQ704265.1; H1a1a1; Asia_W_Europe; 16; A73G; A263G; C309CCT	16570	Asia_W_Europe	H1a1a1	H	H	16
2464	JQ704266.1	JQ704266.1; L1b1a15a; Africa_C; 83; A73G; T146C; T152C	16567	Africa_C	L1b1a15a	L1	L1	83
2465	JQ704267.1	JQ704267.1; H1o; Asia_W_Europe; 14; C150T; A263G; C315CC	16569	Asia_W_Europe	H1o	H	H	14
2466	JQ704268.1	JQ704268.1; U2e1d; Asia_S_W_Europe; 42; A73G; T152C; T195C	16573	Asia_S_W_Europe	U2e1d	U	U2	42
2467	JQ704269.1	JQ704269.1; H2a2a2; Asia_W_Europe; 5; T152C; A263G; C309CCT	16570	Asia_W_Europe	H2a2a2	H	H	5
2468	JQ704270.1	JQ704270.1; H6a1b4; Asia_W_Europe; 17; T239C; A263G; C315CC	16569	Asia_W_Europe	H6a1b4	H	H	17
2469	JQ704271.1	JQ704271.1; U5a1b; Asia_W_Europe_C; 25; A73G; A263G; C315CC	16569	Asia_W_Europe_C	U5a1b	U	U5	25
2470	JQ704272.1	JQ704272.1; H5s; Asia_W_Europe; 15; A263G; C309CCT; T310C	16571	Asia_W_Europe	H5s	H	H	15
2471	JQ704273.1	JQ704273.1; H4a1a1a1a; Asia_W_Europe; 22; A73G; C150T; A263G	16566	Asia_W_Europe	H4a1a1a1a	H	H	22
2472	JQ704274.1	JQ704274.1; H2; Asia_W_Europe; 11; A263G; C309CCCT; T310C	16571	Asia_W_Europe	H2	H	H	11
2473	JQ704275.1	JQ704275.1; H; Asia_W_Europe; 16; T131C; A263G; C309CCCT	16573	Asia_W_Europe	H	H	H	16
2474	JQ704276.1	JQ704276.1; A2ae; Asia_NE_America_N; 38; C64T; A73G; T146C	16568	Asia_NE_America_N	A2ae	A	A	38
2475	JQ704277.1	JQ704277.1; H1q; Asia_W_Europe; 15; A263G; C315CC; A750G	16569	Asia_W_Europe	H1q	H	H	15
2476	JQ704278.1	JQ704278.1; H5a1j; Asia_W_Europe; 17; T152C; A263G; C309CCT	16568	Asia_W_Europe	H5a1j	H	H	17
2477	JQ704279.1	JQ704279.1; H1+16239; Asia_W_Europe; 11; T146C; A263G; C315CC	16569	Asia_W_Europe	H1+16239	H	H	11
2478	JQ704280.1	JQ704280.1; H11a4; Asia_W_Europe; 18; C151T; T195C; A263G	16572	Asia_W_Europe	H11a4	H	H	18
2479	JQ704281.1	JQ704281.1; H67a; Asia_W_Europe; 14; A263G; C309CCT; T310C	16570	Asia_W_Europe	H67a	H	H	14
2480	JQ704282.1	JQ704282.1; H1c1; Asia_W_Europe; 14; A263G; C309CCCT; T310C	16571	Asia_W_Europe	H1c1	H	H	14
2481	JQ704283.1	JQ704283.1; H4c1; Asia_W_Europe; 16; A73G; A263G; C315CC	16567	Asia_W_Europe	H4c1	H	H	16
2482	JQ704284.1	JQ704284.1; HV1b3b; Asia_W; 23; T152C; T195C; A263G	16569	Asia_W	HV1b3b	HV	HV	23
2483	JQ704285.1	JQ704285.1; H1ax; Asia_W_Europe; 15; A263G; C309CCT; T310C	16570	Asia_W_Europe	H1ax	H	H	15
2484	JQ704286.1	JQ704286.1; H11b1; Asia_W_Europe; 14; T195C; A263G; C315CC	16569	Asia_W_Europe	H11b1	H	H	14
2485	JQ704287.1	JQ704287.1; H10a1a; Asia_W_Europe; 13; A263G; C315CC; CA522d	16567	Asia_W_Europe	H10a1a	H	H	13
2486	JQ704288.1	JQ704288.1; H2a2a1; Asia_W_Europe; 5; C309CCT; T310C; A2220G	16570	Asia_W_Europe	H2a2a1	H	H	5
2487	JQ704289.1	JQ704289.1; H1b1; Asia_W_Europe; 15; T152C; A263G; C309CCT	16570	Asia_W_Europe	H1b1	H	H	15
2488	JQ704290.1	JQ704290.1; H44b; Asia_W_Europe; 16; T195C; A263G; C309CCT	16570	Asia_W_Europe	H44b	H	H	16
2489	JQ704291.1	JQ704291.1; H1ar1; Asia_W_Europe; 14; A183G; A263G; C309CCCT	16571	Asia_W_Europe	H1ar1	H	H	14
2490	JQ704292.1	JQ704292.1; H1e2; Asia_W_Europe; 12; A263G; C315CC; A750G	16569	Asia_W_Europe	H1e2	H	H	12
2491	JQ704293.1	JQ704293.1; H1ah2; Asia_W_Europe; 15; A263G; C315CC; A750G	16569	Asia_W_Europe	H1ah2	H	H	15
2492	JQ704294.1	JQ704294.1; H1c8; Asia_W_Europe; 13; A263G; C309CCT; T310C	16570	Asia_W_Europe	H1c8	H	H	13
2493	JQ704295.1	JQ704295.1; H10c1; Asia_W_Europe; 19; A249G; A263G; C309CCT	16570	Asia_W_Europe	H10c1	H	H	19
2494	JQ704296.1	JQ704296.1; H6a1a; Asia_W_Europe; 17; T239C; A263G; C315CC	16569	Asia_W_Europe	H6a1a	H	H	17
2495	JQ704297.1	JQ704297.1; H2a(H2a2a1); Asia_W_Europe; 5; C309CCCT; T310C; G5147A	16571	Asia_W_Europe	H2a(H2a2a1)	H	H	5
2496	JQ704298.1	JQ704298.1; H1f; Asia_W_Europe; 13; A263G; C309CCCT; T310C	16571	Asia_W_Europe	H1f	H	H	13
2497	JQ704299.1	JQ704299.1; H6a1a; Asia_W_Europe; 16; T239C; A263G; C309CCCT	16571	Asia_W_Europe	H6a1a	H	H	16
2498	JQ704300.1	JQ704300.1; H1; Asia_W_Europe; 10; A263G; C315CC; A750G	16569	Asia_W_Europe	H1	H	H	10
2499	JQ704301.1	JQ704301.1; H1a3c; Asia_W_Europe; 14; A73G; A263G; C315CC	16569	Asia_W_Europe	H1a3c	H	H	14
2500	JQ704302.1	JQ704302.1; U5b1e1; Asia_W_Europe_C; 31; A73G; T146C; C150T	16567	Asia_W_Europe_C	U5b1e1	U	U5	31
2501	JQ704303.1	JQ704303.1; H1af; Asia_W_Europe; 10; A263G; C315CC; A750G	16569	Asia_W_Europe	H1af	H	H	10

Supplementary Table S4 Human mitochondrial database (hMITO DB v1.0) metadata[a]

Row	Name (accession no.)	Description	Size	Geo_Region	Haplogroup	Macro_ Haplo	Macro_ Haplo2	Total Variants
2502	JQ704304.1	JQ704304.1; H6a1a; Asia_W_Europe; 16; T239C; A263G; C309CCT	16570	Asia_W_Europe	H6a1a	H	H	16
2503	JQ704305.1	JQ704305.1; HV0+195; Asia_W; 15; T72C; T195C; A263G	16570	Asia_W	HV0+195	HV	HV	15
2504	JQ704306.1	JQ704306.1; J1c16; Asia_W; 33; A73G; T152C; G185A	16569	Asia_W	J1c16	J	J	33
2505	JQ704307.1	JQ704307.1; H1n1a; Asia_W_Europe; 17; T146C; A263G; C309CCT	16572	Asia_W_Europe	H1n1a	H	H	17
2506	JQ704308.1	JQ704308.1; H5b1; Asia_W_Europe; 16; T146C; C150T; T195C	16570	Asia_W_Europe	H5b1	H	H	16
2507	JQ704309.1	JQ704309.1; H1b2; Asia_W_Europe; 17; C151T; A263G; C309CCCT	16571	Asia_W_Europe	H1b2	H	H	17
2508	JQ704310.1	JQ704310.1; H1c+152; Asia_W_Europe; 12; T152C; A263G; C315CC	16569	Asia_W_Europe	H1c+152	H	H	12
2509	JQ704311.1	JQ704311.1; H1e1a; Asia_W_Europe; 13; A263G; C315CC; A750G	16569	Asia_W_Europe	H1e1a	H	H	13
2510	JQ704312.1	JQ704312.1; H1a; Asia_W_Europe; 12; A73G; A263G; C309CCT	16570	Asia_W_Europe	H1a	H	H	12
2511	JQ704313.1	JQ704313.1; H13b2; Asia_W_Europe; 16; A263G; C309CCT; T310C	16568	Asia_W_Europe	H13b2	H	H	16
2512	JQ704314.1	JQ704314.1; H1c1; Asia_W_Europe; 12; A263G; C315CC; T477C	16569	Asia_W_Europe	H1c1	H	H	12
2513	JQ704315.1	JQ704315.1; H3; Asia_W_Europe; 12; A263G; C315CC; A750G	16569	Asia_W_Europe	H3	H	H	12
2514	JQ704316.1	JQ704316.1; H13a1a; Asia_W_Europe; 13; A263G; C309CCCT; T310C	16571	Asia_W_Europe	H13a1a	H	H	13
2515	JQ704317.1	JQ704317.1; H1a5; Asia_W_Europe; 15; A73G; A263G; C315CC	16569	Asia_W_Europe	H1a5	H	H	15
2516	JQ704318.1	JQ704318.1; H3q; Asia_W_Europe; 10; A263G; C315CC; A750G	16569	Asia_W_Europe	H3q	H	H	10
2517	JQ704319.1	JQ704319.1; T2b; Asia_W; 34; A73G; A200G; A263G	16569	Asia_W	T2b	T	T	34
2518	JQ704320.1	JQ704320.1; K1a4a1; Asia_W; 37; A73G; T146C; A263G	16571	Asia_W	K1a4a1	K	K	37
2519	JQ704321.1	JQ704321.1; U4a2a2; Asia_N_Europe_N; 28; A73G; T195C; A263G	16572	Asia_N_Europe_N	U4a2a2	U	U4	28
2520	JQ704322.1	JQ704322.1; HV6; Asia_W; 15; A263G; C309CCCT; T310C	16571	Asia_W	HV6	HV	HV	15
2521	JQ704323.1	JQ704323.1; H1c1a; Asia_W_Europe; 15; A263G; C315CC; T477C	16569	Asia_W_Europe	H1c1a	H	H	15
2522	JQ704324.1	JQ704324.1; H5a1n; Asia_W_Europe; 15; T152C; A263G; C309CCT	16568	Asia_W_Europe	H5a1n	H	H	15
2523	JQ704325.1	JQ704325.1; H39c; Asia_W_Europe; 11; A263G; C309CCCT; T310C	16571	Asia_W_Europe	H39c	H	H	11
2524	JQ704326.1	JQ704326.1; U4c1; Asia_N_Europe_N; 37; A73G; C150T; T195C	16573	Asia_N_Europe_N	U4c1	U	U4	37
2525	JQ704327.1	JQ704327.1; H1e2a; Asia_W_Europe; 14; A263G; C315CC; A750G	16569	Asia_W_Europe	H1e2a	H	H	14
2526	JQ704328.1	JQ704328.1; H3ao; Asia_W_Europe; 12; A93G; A263G; C309CCT	16570	Asia_W_Europe	H3ao	H	H	12
2527	JQ704329.1	JQ704329.1; H1c3; Asia_W_Europe; 15; T195C; A257G; A263G	16570	Asia_W_Europe	H1c3	H	H	15
2528	JQ704330.1	JQ704330.1; H27; Asia_W_Europe; 13; A263G; C309CCCT; T310C	16571	Asia_W_Europe	H27	H	H	13
2529	JQ704331.1	JQ704331.1; H27+16093; Asia_W_Europe; 13; A263G; C315CC; A750G	16569	Asia_W_Europe	H27+16093	H	H	13
2530	JQ704332.1	JQ704332.1; H5a1d; Asia_W_Europe; 15; A263G; C315CC; C456T	16567	Asia_W_Europe	H5a1d	H	H	15
2531	JQ704333.1	JQ704333.1; H6a1a2a; Asia_W_Europe; 19; C41T; T239C; A263G	16571	Asia_W_Europe	H6a1a2a	H	H	19
2532	JQ704334.1	JQ704334.1; H3+16311; Asia_W_Europe; 12; C194T; A263G; C315CC	16569	Asia_W_Europe	H3+16311	H	H	12
2533	JQ704335.1	JQ704335.1; U5b1d1a; Asia_W_Europe_C; 33; A73G; C150T; A263G	16572	Asia_W_Europe_C	U5b1d1a	U	U5	33
2534	JQ704336.1	JQ704336.1; H1ao; Asia_W_Europe; 16; A93G; T146C; T152C	16569	Asia_W_Europe	H1ao	H	H	16
2535	JQ704337.1	JQ704337.1; H2a2a+(16235); Asia_W_Europe; 7; A263G; C309CCT; T310C	16570	Asia_W_Europe	H2a2a+(16235)	H	H	7
2536	JQ704338.1	JQ704338.1; H3g3; Asia_W_Europe; 14; T152C; A263G; C315CC	16569	Asia_W_Europe	H3g3	H	H	14
2537	JQ704339.1	JQ704339.1; H6a1a; Asia_W_Europe; 17; T239C; A263G; C309CCT	16570	Asia_W_Europe	H6a1a	H	H	17
2538	JQ704340.1	JQ704340.1; V24; Europe_S; 16; T72C; A263G; C315CC	16569	Europe_S	V24	V	V	16
2539	JQ704341.1	JQ704341.1; H5h; Asia_W_Europe; 12; A263G; C315CC; C456T	16569	Asia_W_Europe	H5h	H	H	12
2540	JQ704342.1	JQ704342.1; V1b; Europe_S; 21; T72C; A263G; C309CCT	16570	Europe_S	V1b	V	V	21
2541	JQ704343.1	JQ704343.1; H1e2a; Asia_W_Europe; 13; A263G; C315CC; A750G	16569	Asia_W_Europe	H1e2a	H	H	13
2542	JQ704344.1	JQ704344.1; H18; Asia_W_Europe; 12; A263G; C315CC; A750G	16569	Asia_W_Europe	H18	H	H	12

Supplementary Table S4 Human mitochondrial database (hMITO DB v1.0) metadata[a]

Row	Name (accession no.)	Description	Size	Geo_Region	Haplogroup	Macro_ Haplo	Macro_ Haplo2	Total Variants
2543	JQ704345.1	JQ704345.1; U4a1a3; Asia_N_Europe_N; 35; A73G; T152C; T195C	16573	Asia_N_Europe_N	U4a1a3	U	U4	35
2544	JQ704346.1	JQ704346.1; H2a2a1b; Asia_W_Europe; 2; A9299G; G16145A;	16568	Asia_W_Europe	H2a2a1b	H	H	2
2545	JQ704347.1	JQ704347.1; H5a1f; Asia_W_Europe; 14; A263G; T310C; C456T	16566	Asia_W_Europe	H5a1f	H	H	14
2546	JQ704348.1	JQ704348.1; H3a1a; Asia_W_Europe; 15; T152C; A263G; C309CCT	16570	Asia_W_Europe	H3a1a	H	H	15
2547	JQ704349.1	JQ704349.1; HV4a2b; Asia_W; 18; A93G; A263G; C309CCT	16570	Asia_W	HV4a2b	HV	HV	18
2548	JQ704350.1	JQ704350.1; A2+(64); Asia_NE_America_N; 35; C64T; A73G; T146C	16568	Asia_NE_America_N	A2+(64)	A	A	35
2549	JQ704351.1	JQ704351.1; H13a1a1a; Asia_W_Europe; 17; T152C; A263G; C309CCT	16570	Asia_W_Europe	H13a1a1a	H	H	17
2550	JQ704352.1	JQ704352.1; H1c11; Asia_W_Europe; 13; A263G; C315CC; T477C	16568	Asia_W_Europe	H1c11	H	H	13
2551	JQ704353.1	JQ704353.1; H; Asia_W_Europe; 10; A263G; C315CC; A750G	16569	Asia_W_Europe	H	H	H	10
2552	JQ704354.1	JQ704354.1; H3; Asia_W_Europe; 14; G207A; A263G; C309CCT	16570	Asia_W_Europe	H3	H	H	14
2553	JQ704355.1	JQ704355.1; H3ap; Asia_W_Europe; 12; A263G; C309CCT; T310C	16569	Asia_W_Europe	H3ap	H	H	12
2554	JQ704356.1	JQ704356.1; H1c1a; Asia_W_Europe; 16; A263G; C315CC; T477C	16571	Asia_W_Europe	H1c1a	H	H	16
2555	JQ704357.1	JQ704357.1; H7d1; Asia_W_Europe; 13; A263G; C315CC; A750G	16569	Asia_W_Europe	H7d1	H	H	13
2556	JQ704358.1	JQ704358.1; H1j3; Asia_W_Europe; 11; A263G; C315CC; A750G	16569	Asia_W_Europe	H1j3	H	H	11
2557	JQ704359.1	JQ704359.1; H5a1+16093; Asia_W_Europe; 15; A263G; C315CC; C456T	16567	Asia_W_Europe	H5a1+16093	H	H	15
2558	JQ704360.1	JQ704360.1; H7b; Asia_W_Europe; 12; A263G; C315CC; A750G	16569	Asia_W_Europe	H7b	H	H	12
2559	JQ704361.1	JQ704361.1; H2a2b1a1; Asia_W_Europe; 12; A263G; C309CCCT; T310C	16571	Asia_W_Europe	H2a2b1a1	H	H	12
2560	JQ704362.1	JQ704362.1; H7a; Asia_W_Europe; 13; A263G; C309CCCT; T310C	16571	Asia_W_Europe	H7a	H	H	13
2561	JQ704363.1	JQ704363.1; H3m; Asia_W_Europe; 14; A263G; C315CC; C463T	16569	Asia_W_Europe	H3m	H	H	14
2562	JQ704364.1	JQ704364.1; H15a1a; Asia_W_Europe; 13; T55C; T57C; A263G	16569	Asia_W_Europe	H15a1a	H	H	13
2563	JQ704365.1	JQ704365.1; H17c; Asia_W_Europe; 13; A73G; A263G; C315CC	16569	Asia_W_Europe	H17c	H	H	13
2564	JQ704366.1	JQ704366.1; H1bw; Asia_W_Europe; 13; A263G; C309CCT; T310C	16570	Asia_W_Europe	H1bw	H	H	13
2565	JQ704367.1	JQ704367.1; H6a1b2; Asia_W_Europe; 20; T152C; T239C; A263G	16571	Asia_W_Europe	H6a1b2	H	H	20
2566	JQ704368.1	JQ704368.1; H1c; Asia_W_Europe; 16; A263G; C315CC; T477C	16567	Asia_W_Europe	H1c	H	H	16
2567	JQ704369.1	JQ704369.1; H1bb; Asia_W_Europe; 12; T152C; A263G; C315CC	16571	Asia_W_Europe	H1bb	H	H	12
2568	JQ704370.1	JQ704370.1; H1as2; Asia_W_Europe; 12; A263G; C309CCT; T310C	16570	Asia_W_Europe	H1as2	H	H	12
2569	JQ704371.1	JQ704371.1; H56c; Asia_W_Europe; 12; A263G; C309CCT; T310C	16570	Asia_W_Europe	H56c	H	H	12
2570	JQ704372.1	JQ704372.1; H5a3a; Asia_W_Europe; 21; A263G; C309CCT; T310C	16570	Asia_W_Europe	H5a3a	H	H	21
2571	JQ704373.1	JQ704373.1; H1q1; Asia_W_Europe; 12; A263G; C315CC; A750G	16569	Asia_W_Europe	H1q1	H	H	12
2572	JQ704374.1	JQ704374.1; H4a1a1; Asia_W_Europe; 15; A263G; C315CC; A750G	16569	Asia_W_Europe	H4a1a1	H	H	15
2573	JQ704375.1	JQ704375.1; H13b1a; Asia_W_Europe; 13; A263G; C315CC; A750G	16569	Asia_W_Europe	H13b1a	H	H	13
2574	JQ704376.1	JQ704376.1; H6a1b2a; Asia_W_Europe; 18; T239C; A263G; C309CCT	16570	Asia_W_Europe	H6a1b2a	H	H	18
2575	JQ704377.1	JQ704377.1; H7f; Asia_W_Europe; 10; A263G; C315CC; A750G	16569	Asia_W_Europe	H7f	H	H	10
2576	JQ704378.1	JQ704378.1; H1c4b; Asia_W_Europe; 12; A263G; C315CC; T477C	16569	Asia_W_Europe	H1c4b	H	H	12
2577	JQ704379.1	JQ704379.1; H7a1; Asia_W_Europe; 14; A263G; C309CCT; T310C	16570	Asia_W_Europe	H7a1	H	H	14
2578	JQ704380.1	JQ704380.1; H6a1a9; Asia_W_Europe; 18; T204C; T239C; A263G	16570	Asia_W_Europe	H6a1a9	H	H	18
2579	JQ704381.1	JQ704381.1; H1ag1; Asia_W_Europe; 15; T152C; A263G; A291AA	16570	Asia_W_Europe	H1ag1	H	H	15
2580	JQ704382.1	JQ704382.1; H1c2; Asia_W_Europe; 13; A263G; C309CCT; T310C	16570	Asia_W_Europe	H1c2	H	H	13
2581	JQ704383.1	JQ704383.1; H3aq; Asia_W_Europe; 11; A263G; C315CC; A750G	16569	Asia_W_Europe	H3aq	H	H	11
2582	JQ704384.1	JQ704384.1; J1c2b; Asia_W; 33; A73G; T152C; G185A	16568	Asia_W	J1c2b	J	J	33
2583	JQ704385.1	JQ704385.1; H10e; Asia_W_Europe; 12; C150T; A263G; C309CCT	16570	Asia_W_Europe	H10e	H	H	12

Supplementary Table S4 Human mitochondrial database (hMITO DB v1.0) metadata[a]

Row	Name (accession no.)	Description	Size	Geo_Region	Haplogroup	Macro_ Haplo	Macro_ Haplo2	Total Variants
2584	JQ704386.1	JQ704386.1; H5j; Asia_W_Europe; 17; A263G; C309CCCT; T310C	16571	Asia_W_Europe	H5j	H	H	17
2585	JQ704387.1	JQ704387.1; H10e1a; Asia_W_Europe; 14; A263G; C309CCT; T310C	16570	Asia_W_Europe	H10e1a	H	H	14
2586	JQ704388.1	JQ704388.1; H; Asia_W_Europe; 12; A263G; C315CC; A750G	16569	Asia_W_Europe	H	H	H	12
2587	JQ704389.1	JQ704389.1; H30b; Asia_W_Europe; 15; A263G; C315CC; A750G	16569	Asia_W_Europe	H30b	H	H	15
2588	JQ704390.1	JQ704390.1; H6a1a; Asia_W_Europe; 16; T239C; A263G; C309CCT	16570	Asia_W_Europe	H6a1a	H	H	16
2589	JQ704391.1	JQ704391.1; H1; Asia_W_Europe; 14; A263G; C309CCT; T310C	16570	Asia_W_Europe	H1	H	H	14
2590	JQ704392.1	JQ704392.1; H1e4; Asia_W_Europe; 13; A263G; C315CC; A750G	16569	Asia_W_Europe	H1e4	H	H	13
2591	JQ704393.1	JQ704393.1; H6a1a; Asia_W_Europe; 18; T239C; A263G; C309CCT	16570	Asia_W_Europe	H6a1a	H	H	18
2592	JQ704394.1	JQ704394.1; H93; Asia_W_Europe; 9; A263G; C315CC; A750G	16569	Asia_W_Europe	H93	H	H	9
2593	JQ704395.1	JQ704395.1; H11a2a; Asia_W_Europe; 17; T195C; A263G; C315CC	16569	Asia_W_Europe	H11a2a	H	H	17
2594	JQ704396.1	JQ704396.1; H5a7; Asia_W_Europe; 13; A263G; C309CCT; T310C	16570	Asia_W_Europe	H5a7	H	H	13
2595	JQ704397.1	JQ704397.1; H5a1; Asia_W_Europe; 14; A263G; C309CCCT; T310C	16571	Asia_W_Europe	H5a1	H	H	14
2596	JQ704398.1	JQ704398.1; H1j4; Asia_W_Europe; 13; A263G; C309CCT; T310C	16570	Asia_W_Europe	H1j4	H	H	13
2597	JQ704399.1	JQ704399.1; H5a1; Asia_W_Europe; 14; A263G; C309CCT; T310C	16568	Asia_W_Europe	H5a1	H	H	14
2598	JQ704400.1	JQ704400.1; H1be; Asia_W_Europe; 14; A263G; C309CCT; T310C	16570	Asia_W_Europe	H1be	H	H	14
2599	JQ704401.1	JQ704401.1; H5b1; Asia_W_Europe; 17; T146C; T152C; T195C	16570	Asia_W_Europe	H5b1	H	H	17
2600	JQ704402.1	JQ704402.1; H1b; Asia_W_Europe; 13; A263G; C315CC; A750G	16569	Asia_W_Europe	H1b	H	H	13
2601	JQ704403.1	JQ704403.1; H28a; Asia_W_Europe; 12; C186A; A263G; C315CC	16569	Asia_W_Europe	H28a	H	H	12
2602	JQ704404.1	JQ704404.1; H17a1; Asia_W_Europe; 18; A263G; C309CCCT; T310C	16571	Asia_W_Europe	H17a1	H	H	18
2603	JQ704405.1	JQ704405.1; V11; Europe_S; 18; T72C; A263G; C309CCCT	16571	Europe_S	V11	V	V	18
2604	JQ704406.1	JQ704406.1; V7a; Europe_S; 17; T72C; A93G; T195C	16569	Europe_S	V7a	V	V	17
2605	JQ704407.1	JQ704407.1; H1b1+16362; Asia_W_Europe; 15; T152C; A263G; C315CC	16567	Asia_W_Europe	H1b1+16362	H	H	15
2606	JQ704408.1	JQ704408.1; H4a1a1a3; Asia_W_Europe; 21; A73G; T146C; A263G	16567	Asia_W_Europe	H4a1a1a3	H	H	21
2607	JQ704409.1	JQ704409.1; H16; Asia_W_Europe; 10; A263G; C309CCT; T310C	16570	Asia_W_Europe	H16	H	H	10
2608	JQ704410.1	JQ704410.1; H1ah1; Asia_W_Europe; 18; A263G; C309CCCT; T310C	16571	Asia_W_Europe	H1ah1	H	H	18
2609	JQ704411.1	JQ704411.1; H1aw; Asia_W_Europe; 14; A263G; C309CCCT; T310C	16571	Asia_W_Europe	H1aw	H	H	14
2610	JQ704412.1	JQ704412.1; H1ae1; Asia_W_Europe; 13; A263G; C315CC; A750G	16569	Asia_W_Europe	H1ae1	H	H	13
2611	JQ704413.1	JQ704413.1; H1a1a; Asia_W_Europe; 16; A73G; A263G; C315CCC	16570	Asia_W_Europe	H1a1a	H	H	16
2612	JQ704414.1	JQ704414.1; H1cj; Asia_W_Europe; 16; A263G; C309CCT; T310C	16570	Asia_W_Europe	H1cj	H	H	16
2613	JQ704415.1	JQ704415.1; H5a2; Asia_W_Europe; 13; A263G; C309CCT; T310C	16570	Asia_W_Europe	H5a2	H	H	13
2614	JQ704416.1	JQ704416.1; H28a1; Asia_W_Europe; 15; C186A; A263G; C309CCT	16570	Asia_W_Europe	H28a1	H	H	15
2615	JQ704417.1	JQ704417.1; H6a1a2a; Asia_W_Europe; 21; C41T; T146C; T152C	16571	Asia_W_Europe	H6a1a2a	H	H	21
2616	JQ704418.1	JQ704418.1; H1e1a6; Asia_W_Europe; 15; C150T; A263G; C315CC	16569	Asia_W_Europe	H1e1a6	H	H	15
2617	JQ704419.1	JQ704419.1; H1bt1; Asia_W_Europe; 13; A263G; C315CC; A750G	16569	Asia_W_Europe	H1bt1	H	H	13
2618	JQ704420.1	JQ704420.1; U3a1c1; Africa_NE_Asia_W; 32; A73G; C150T; A263G	16569	Africa_NE_Asia_W	U3a1c1	U	U3	32
2619	JQ704421.1	JQ704421.1; H; Asia_W_Europe; 10; A263G; C315CC; A750G	16569	Asia_W_Europe	H	H	H	10
2620	JQ704422.1	JQ704422.1; H3; Asia_W_Europe; 11; A263G; C315CC; A750G	16569	Asia_W_Europe	H3	H	H	11
2621	JQ704423.1	JQ704423.1; H5d; Asia_W_Europe; 11; A263G; C315CC; C456T	16569	Asia_W_Europe	H5d	H	H	11
2622	JQ704424.1	JQ704424.1; H1be; Asia_W_Europe; 13; A263G; C309CCT; T310C	16570	Asia_W_Europe	H1be	H	H	13
2623	JQ704425.1	JQ704425.1; H2a1; Asia_W_Europe; 8; A263G; C315CC; A750G	16569	Asia_W_Europe	H2a1	H	H	8
2624	JQ704426.1	JQ704426.1; T2f3; Asia_W; 36; A73G; A263G; C309CCT	16561	Asia_W	T2f3	T	T	36

Supplementary Table S4 Human mitochondrial database (hMITO DB v1.0) metadata[a]

Row	Name (accession no.)	Description	Size	Geo_Region	Haplogroup	Macro_ Haplo	Macro_ Haplo2	Total Variants
2625	JQ704427.1	JQ704427.1; H11a2a2; Asia_W_Europe; 20; T152C; T195C; A263G	16569	Asia_W_Europe	H11a2a2	H	H	20
2626	JQ704428.1	JQ704428.1; H7b6; Asia_W_Europe; 12; A263G; C309CCT; T310C	16570	Asia_W_Europe	H7b6	H	H	12
2627	JQ704429.1	JQ704429.1; H1ao; Asia_W_Europe; 14; A93G; T146C; A263G	16570	Asia_W_Europe	H1ao	H	H	14
2628	JQ704430.1	JQ704430.1; H3a1; Asia_W_Europe; 16; T152C; A263G; C309CCCT	16571	Asia_W_Europe	H3a1	H	H	16
2629	JQ704431.1	JQ704431.1; H1c1; Asia_W_Europe; 13; A215G; A263G; C315CC	16569	Asia_W_Europe	H1c1	H	H	13
2630	JQ704432.1	JQ704432.1; J1c2f; Asia_W; 30; A73G; G185A; A188G	16569	Asia_W	J1c2f	J	J	30
2631	JQ704433.1	JQ704433.1; H1ae1; Asia_W_Europe; 12; A263G; C315CC; A750G	16569	Asia_W_Europe	H1ae1	H	H	12
2632	JQ704434.1	JQ704434.1; H2a1+146; Asia_W_Europe; 8; T146C; A263G; C315CC	16569	Asia_W_Europe	H2a1+146	H	H	8
2633	JQ704435.1	JQ704435.1; H1bi; Asia_W_Europe; 13; A263G; C309CCT; T310C	16570	Asia_W_Europe	H1bi	H	H	13
2634	JQ704436.1	JQ704436.1; H3h3a; Asia_W_Europe; 13; A263G; C315CC; A750G	16569	Asia_W_Europe	H3h3a	H	H	13
2635	JQ704437.1	JQ704437.1; H5; Asia_W_Europe; 13; A263G; C309CCT; T310C	16570	Asia_W_Europe	H5	H	H	13
2636	JQ704438.1	JQ704438.1; H1c2a; Asia_W_Europe; 12; A263G; C315CC; T477C	16569	Asia_W_Europe	H1c2a	H	H	12
2637	JQ704439.1	JQ704439.1; H1c1; Asia_W_Europe; 14; A263G; C309CCT; T310C	16570	Asia_W_Europe	H1c1	H	H	14
2638	JQ704440.1	JQ704440.1; U3a3; Africa_NE_Asia_W; 35; A73G; C150T; T152C	16569	Africa_NE_Asia_W	U3a3	U	U3	35
2639	JQ704441.1	JQ704441.1; HV0a; Asia_W; 19; T72C; A263G; C309CCT	16570	Asia_W	HV0a	HV	HV	19
2640	JQ704442.1	JQ704442.1; H5a1b; Asia_W_Europe; 14; A263G; C315CC; C456T	16567	Asia_W_Europe	H5a1b	H	H	14
2641	JQ704443.1	JQ704443.1; H18; Asia_W_Europe; 10; A263G; C315CC; A750G	16569	Asia_W_Europe	H18	H	H	10
2642	JQ704444.1	JQ704444.1; H1j; Asia_W_Europe; 12; T152C; A263G; C315CC	16569	Asia_W_Europe	H1j	H	H	12
2643	JQ704445.1	JQ704445.1; HV4a1; Asia_W; 17; A263G; C309CCT; T310C	16570	Asia_W	HV4a1	HV	HV	17
2644	JQ704446.1	JQ704446.1; H1b1+16362; Asia_W_Europe; 16; T195C; A263G; C315CC	16567	Asia_W_Europe	H1b1+16362	H	H	16
2645	JQ704447.1	JQ704447.1; H1+16239; Asia_W_Europe; 10; A263G; C315CC; A750G	16569	Asia_W_Europe	H1+16239	H	H	10
2646	JQ704448.1	JQ704448.1; H1ag1; Asia_W_Europe; 13; A263G; C315CC; A750G	16569	Asia_W_Europe	H1ag1	H	H	13
2647	JQ704449.1	JQ704449.1; H3k1a; Asia_W_Europe; 16; T152C; A263G; C309CCT	16570	Asia_W_Europe	H3k1a	H	H	16
2648	JQ704450.1	JQ704450.1; A2ae; Asia_NE_America_N; 38; C64T; A73G; T146C	16568	Asia_NE_America_N	A2ae	A	A	38
2649	JQ704451.1	JQ704451.1; H26a1; Asia_W_Europe; 12; A263G; C315CC; A750G	16569	Asia_W_Europe	H26a1	H	H	12
2650	JQ704452.1	JQ704452.1; H10a1a1; Asia_W_Europe; 15; A263G; C309CCT; T310C	16570	Asia_W_Europe	H10a1a1	H	H	15
2651	JQ704453.1	JQ704453.1; H6a1b2; Asia_W_Europe; 17; T239C; A263G; C315CC	16569	Asia_W_Europe	H6a1b2	H	H	17
2652	JQ704454.1	JQ704454.1; H1b; Asia_W_Europe; 15; A263G; C315CC; A750G	16569	Asia_W_Europe	H1b	H	H	15
2653	JQ704455.1	JQ704455.1; H3g2; Asia_W_Europe; 13; T152C; A263G; C315CC	16571	Asia_W_Europe	H3g2	H	H	13
2654	JQ704456.1	JQ704456.1; H3b1b; Asia_W_Europe; 16; A153G; A263G; C309CCCT	16571	Asia_W_Europe	H3b1b	H	H	16
2655	JQ704457.1	JQ704457.1; H17c; Asia_W_Europe; 13; A73G; A263G; C315CC	16569	Asia_W_Europe	H17c	H	H	13
2656	JQ704458.1	JQ704458.1; H6a1b4; Asia_W_Europe; 18; T152C; T239C; A263G	16569	Asia_W_Europe	H6a1b4	H	H	18
2657	JQ704459.1	JQ704459.1; H1m1; Asia_W_Europe; 15; T146C; A263G; C315CC	16569	Asia_W_Europe	H1m1	H	H	15
2658	JQ704460.1	JQ704460.1; H55b; Asia_W_Europe; 13; A153G; A263G; C309CCCT	16571	Asia_W_Europe	H55b	H	H	13
2659	JQ704461.1	JQ704461.1; H39a1; Asia_W_Europe; 13; A263G; C309CCT; T310C	16570	Asia_W_Europe	H39a1	H	H	13
2660	JQ704462.1	JQ704462.1; H1j; Asia_W_Europe; 13; A263G; C315CC; A750G	16569	Asia_W_Europe	H1j	H	H	13
2661	JQ704463.1	JQ704463.1; HV2a1; Asia_W; 27; A73G; T152C; T195C	16572	Asia_W	HV2a1	HV	HV	27
2662	JQ704464.1	JQ704464.1; H1e1a+16278; Asia_W_Europe; 16; A263G; C315CC; A750G	16569	Asia_W_Europe	H1e1a+16278	H	H	16
2663	JQ704465.1	JQ704465.1; H1ch; Asia_W_Europe; 15; A263G; C309CCT; T310C	16570	Asia_W_Europe	H1ch	H	H	15
2664	JQ704466.1	JQ704466.1; H1at1a; Asia_W_Europe; 15; A263G; C309CCCT; T310C	16571	Asia_W_Europe	H1at1a	H	H	15
2665	JQ704467.1	JQ704467.1; H5a+152; Asia_W_Europe; 14; T152C; G207A; A263G	16570	Asia_W_Europe	H5a+152	H	H	14

Supplementary Table S4 Human mitochondrial database (hMITO DB v1.0) metadata[a]

Row	Name (accession no.)	Description	Size	Geo_Region	Haplogroup	Macro_ Haplo	Macro_ Haplo2	Total Variants
2666	JQ704468.1	JQ704468.1; V16; Europe_S; 15; T72C; A263G; C315CC	16569	Europe_S	V16	V	V	15
2667	JQ704469.1	JQ704469.1; H6a1a8a; Asia_W_Europe; 19; T239C; A263G; C309CCT	16570	Asia_W_Europe	H6a1a8a	H	H	19
2668	JQ704470.1	JQ704470.1; H1+16239; Asia_W_Europe; 10; A263G; C315CC; A750G	16569	Asia_W_Europe	H1+16239	H	H	10
2669	JQ704471.1	JQ704471.1; H4a1; Asia_W_Europe; 19; A263G; C315CC; CA522d	16567	Asia_W_Europe	H4a1	H	H	19
2670	JQ704472.1	JQ704472.1; H6a1a4; Asia_W_Europe; 19; A93G; T239C; A263G	16571	Asia_W_Europe	H6a1a4	H	H	19
2671	JQ704473.1	JQ704473.1; H7c; Asia_W_Europe; 13; A263G; C315CC; CA522d	16567	Asia_W_Europe	H7c	H	H	13
2672	JQ704474.1	JQ704474.1; H6a1b2; Asia_W_Europe; 17; T239C; A263G; C309CCCT	16571	Asia_W_Europe	H6a1b2	H	H	17
2673	JQ704475.1	JQ704475.1; H13b1b; Asia_W_Europe; 18; A200G; A263G; C309CCCT	16571	Asia_W_Europe	H13b1b	H	H	18
2674	JQ704476.1	JQ704476.1; H6a1b3b; Asia_W_Europe; 22; T204C; G207A; T239C	16571	Asia_W_Europe	H6a1b3b	H	H	22
2675	JQ704477.1	JQ704477.1; H3au; Asia_W_Europe; 11; A263G; C315CC; A750G	16569	Asia_W_Europe	H3au	H	H	11
2676	JQ704478.1	JQ704478.1; H2a2a1; Asia_W_Europe; 7; C315CC; C2225T; C9245G	16569	Asia_W_Europe	H2a2a1	H	H	7
2677	JQ704479.1	JQ704479.1; H6a1a; Asia_W_Europe; 14; T239C; A263G; C309CCT	16570	Asia_W_Europe	H6a1a	H	H	14
2678	JQ704480.1	JQ704480.1; H39; Asia_W_Europe; 11; A263G; C309CCCT; T310C	16571	Asia_W_Europe	H39	H	H	11
2679	JQ704481.1	JQ704481.1; H1; Asia_W_Europe; 13; A263G; C309CCT; T310C	16570	Asia_W_Europe	H1	H	H	13
2680	JQ704482.1	JQ704482.1; H3ak; Asia_W_Europe; 15; A73G; G143A; A263G	16569	Asia_W_Europe	H3ak	H	H	15
2681	JQ704483.1	JQ704483.1; H13a1a1; Asia_W_Europe; 17; C150T; A263G; T310C	16569	Asia_W_Europe	H13a1a1	H	H	17
2682	JQ704484.1	JQ704484.1; H3h4; Asia_W_Europe; 15; T146C; T217C; A263G	16569	Asia_W_Europe	H3h4	H	H	15
2683	JQ704485.1	JQ704485.1; H1ag1; Asia_W_Europe; 14; A263G; C309CCT; T310C	16570	Asia_W_Europe	H1ag1	H	H	14
2684	JQ704486.1	JQ704486.1; H1b5; Asia_W_Europe; 16; A263G; C315CC; A750G	16569	Asia_W_Europe	H1b5	H	H	16
2685	JQ704487.1	JQ704487.1; H1c1; Asia_W_Europe; 13; A263G; C315CC; T477C	16569	Asia_W_Europe	H1c1	H	H	13
2686	JQ704488.1	JQ704488.1; H2a1+146; Asia_W_Europe; 9; T146C; A263G; C309CC	16569	Asia_W_Europe	H2a1+146	H	H	9
2687	JQ704489.1	JQ704489.1; H6a1a; Asia_W_Europe; 18; T239C; A263G; C309CCT	16570	Asia_W_Europe	H6a1a	H	H	18
2688	JQ704490.1	JQ704490.1; H3p; Asia_W_Europe; 10; A263G; C315CC; A750G	16569	Asia_W_Europe	H3p	H	H	10
2689	JQ704491.1	JQ704491.1; H63; Asia_W_Europe; 12; A263G; C309CCCT; T310C	16571	Asia_W_Europe	H63	H	H	12
2690	JQ704492.1	JQ704492.1; H2a2b; Asia_W_Europe; 11; A263G; C309CCT; T310C	16570	Asia_W_Europe	H2a2b	H	H	11
2691	JQ704493.1	JQ704493.1; H60a; Asia_W_Europe; 13; A263G; C315CC; T466C	16569	Asia_W_Europe	H60a	H	H	13
2692	JQ704494.1	JQ704494.1; H10e1a; Asia_W_Europe; 15; A263G; C309CCT; T310C	16570	Asia_W_Europe	H10e1a	H	H	15
2693	JQ704495.1	JQ704495.1; H6a1b2; Asia_W_Europe; 18; T239C; A263G; C309CCCT	16571	Asia_W_Europe	H6a1b2	H	H	18
2694	JQ704496.1	JQ704496.1; H2c1; Asia_W_Europe; 16; C194T; G205A; A263G	16570	Asia_W_Europe	H2c1	H	H	16
2695	JQ704497.1	JQ704497.1; H10+(16093); Asia_W_Europe; 14; A93G; A263G; C315CC	16567	Asia_W_Europe	H10+(16093)	H	H	14
2696	JQ704498.1	JQ704498.1; H5d; Asia_W_Europe; 10; T131C; A263G; C456T	16568	Asia_W_Europe	H5d	H	H	10
2697	JQ704499.1	JQ704499.1; H2c1; Asia_W_Europe; 16; C194T; G205A; A263G	16568	Asia_W_Europe	H2c1	H	H	16
2698	JQ704500.1	JQ704500.1; H41a; Asia_W_Europe; 19; C262T; A263G; C309CCT	16570	Asia_W_Europe	H41a	H	H	19
2699	JQ704501.1	JQ704501.1; H1e1a; Asia_W_Europe; 12; A263G; C315CC; A750G	16569	Asia_W_Europe	H1e1a	H	H	12
2700	JQ704502.1	JQ704502.1; H1a3a; Asia_W_Europe; 15; A73G; A263G; C309CCCT	16571	Asia_W_Europe	H1a3a	H	H	15
2701	JQ704503.1	JQ704503.1; H2c; Asia_W_Europe; 13; T152C; A263G; C315CC	16567	Asia_W_Europe	H2c	H	H	13
2702	JQ704504.1	JQ704504.1; H15a1; Asia_W_Europe; 16; T55C; T57C; A263G	16570	Asia_W_Europe	H15a1	H	H	16
2703	JQ704505.1	JQ704505.1; T2b; Asia_W; 35; A73G; A263G; C315CC	16569	Asia_W	T2b	T	T	35
2704	JQ704506.1	JQ704506.1; H7d1; Asia_W_Europe; 14; A263G; C315CC; A750G	16569	Asia_W_Europe	H7d1	H	H	14
2705	JQ704507.1	JQ704507.1; H1a; Asia_W_Europe; 14; A73G; G185A; A263G	16570	Asia_W_Europe	H1a	H	H	14
2706	JQ704508.1	JQ704508.1; K1a10a; Asia_W; 35; A73G; T195C; A263G	16573	Asia_W	K1a10a	K	K	35

Supplementary Table S4 Human mitochondrial database (hMITO DB v1.0) metadata[a]

Row	Name (accession no.)	Description	Size	Geo_Region	Haplogroup	Macro_ Haplo	Macro_ Haplo2	Total Variants
2707	JQ704509.1	JQ704509.1; H1ae3a; Asia_W_Europe; 15; A263G; C309CCT; T310C	16570	Asia_W_Europe	H1ae3a	H	H	15
2708	JQ704510.1	JQ704510.1; H31a; Asia_W_Europe; 15; T72G; T146C; T195C	16570	Asia_W_Europe	H31a	H	H	15
2709	JQ704511.1	JQ704511.1; H1+16189; Asia_W_Europe; 12; T152C; A263G; C309CCT	16570	Asia_W_Europe	H1+16189	H	H	12
2710	JQ704512.1	JQ704512.1; H6a1b3; Asia_W_Europe; 24; T204C; T239C; A263G	16570	Asia_W_Europe	H6a1b3	H	H	24
2711	JQ704513.1	JQ704513.1; H1ab1; Asia_W_Europe; 16; A263G; C309CCT; T310C	16570	Asia_W_Europe	H1ab1	H	H	16
2712	JQ704514.1	JQ704514.1; V19; Europe_S; 19; T72C; C150T; A263G	16570	Europe_S	V19	V	V	19
2713	JQ704515.1	JQ704515.1; H6a2; Asia_W_Europe; 16; C150T; T152C; T239C	16569	Asia_W_Europe	H6a2	H	H	16
2714	JQ704516.1	JQ704516.1; H1+16189; Asia_W_Europe; 12; A263G; C309CCCT; T310C	16571	Asia_W_Europe	H1+16189	H	H	12
2715	JQ704517.1	JQ704517.1; U5b1b1e; Asia_W_Europe_C; 31; A73G; C150T; T152C	16570	Asia_W_Europe_C	U5b1b1e	U	U5	31
2716	JQ704518.1	JQ704518.1; H7a1a; Asia_W_Europe; 20; A93G; A263G; C315CC	16567	Asia_W_Europe	H7a1a	H	H	20
2717	JQ704519.1	JQ704519.1; H1g1; Asia_W_Europe; 15; A200G; A263G; C315CC	16569	Asia_W_Europe	H1g1	H	H	15
2718	JQ704520.1	JQ704520.1; H2a2a1f; Asia_W_Europe; 2; A93G; C315CC;	16569	Asia_W_Europe	H2a2a1f	H	H	2
2719	JQ704521.1	JQ704521.1; H76a; Asia_W_Europe; 14; T152C; A263G; C309CCT	16570	Asia_W_Europe	H76a	H	H	14
2720	JQ704522.1	JQ704522.1; H1c1; Asia_W_Europe; 14; A263G; C315CC; T477C	16569	Asia_W_Europe	H1c1	H	H	14
2721	JQ704523.1	JQ704523.1; J2b1a6; Asia_W; 37; A73G; C150T; A263G	16572	Asia_W	J2b1a6	J	J	37
2722	JQ704524.1	JQ704524.1; H7a1c; Asia_W_Europe; 13; A263G; C309CCT; T310C	16570	Asia_W_Europe	H7a1c	H	H	13
2723	JQ704525.1	JQ704525.1; H1a5; Asia_W_Europe; 15; A73G; A263G; C309CCT	16570	Asia_W_Europe	H1a5	H	H	15
2724	JQ704526.1	JQ704526.1; K1a4a1e; Asia_W; 35; A73G; T152C; A263G	16568	Asia_W	K1a4a1e	K	K	35
2725	JQ704527.1	JQ704527.1; H7e; Asia_W_Europe; 13; A263G; C309CCT; T310C	16570	Asia_W_Europe	H7e	H	H	13
2726	JQ704528.1	JQ704528.1; H3ap; Asia_W_Europe; 15; A73G; T199C; A263G	16570	Asia_W_Europe	H3ap	H	H	15
2727	JQ704529.1	JQ704529.1; H7c1; Asia_W_Europe; 17; C198T; A263G; C309CCT	16572	Asia_W_Europe	H7c1	H	H	17
2728	JQ704530.1	JQ704530.1; H2b; Asia_W_Europe; 12; T152C; A263G; C309CCT	16570	Asia_W_Europe	H2b	H	H	12
2729	JQ704531.1	JQ704531.1; H10a1b; Asia_W_Europe; 17; A263G; C309CCT; T310C	16570	Asia_W_Europe	H10a1b	H	H	17
2730	JQ704532.1	JQ704532.1; H2a2a1h; Asia_W_Europe; 3; C309CCCT; T310C; C16320T	16571	Asia_W_Europe	H2a2a1h	H	H	3
2731	JQ704533.1	JQ704533.1; H2a2; Asia_W_Europe; 7; A263G; C309CCT; T310C	16570	Asia_W_Europe	H2a2	H	H	7
2732	JQ704534.1	JQ704534.1; H10e1; Asia_W_Europe; 14; A263G; C309CCT; T310C	16570	Asia_W_Europe	H10e1	H	H	14
2733	JQ704535.1	JQ704535.1; H49; Asia_W_Europe; 13; A263G; C309CCT; T310C	16570	Asia_W_Europe	H49	H	H	13
2734	JQ704536.1	JQ704536.1; H1a3d; Asia_W_Europe; 14; A73G; A263G; C315CC	16569	Asia_W_Europe	H1a3d	H	H	14
2735	JQ704537.1	JQ704537.1; K1a10a; Asia_W; 34; A73G; T195C; A263G	16573	Asia_W	K1a10a	K	K	34
2736	JQ704538.1	JQ704538.1; H1av; Asia_W_Europe; 11; A263G; C315CC; A750G	16569	Asia_W_Europe	H1av	H	H	11
2737	JQ704539.1	JQ704539.1; U6a1a2; Asia_SW_Africa_N; 32; A73G; A263G; C309CCCT	16571	Asia_SW_Africa_N	U6a1a2	U	U6	32
2738	JQ704540.1	JQ704540.1; H29b; Asia_W_Europe; 16; A93G; A95T; A263G	16571	Asia_W_Europe	H29b	H	H	16
2739	JQ704541.1	JQ704541.1; H27e; Asia_W_Europe; 13; A263G; C315CC; A750G	16560	Asia_W_Europe	H27e	H	H	13
2740	JQ704542.1	JQ704542.1; H3g2; Asia_W_Europe; 13; T152C; A263G; C315CC	16569	Asia_W_Europe	H3g2	H	H	13
2741	JQ704543.1	JQ704543.1; H6a1b3a; Asia_W_Europe; 22; T204C; T239C; A263G	16571	Asia_W_Europe	H6a1b3a	H	H	22
2742	JQ704544.1	JQ704544.1; H1+16239; Asia_W_Europe; 13; A263G; C315CC; A750G	16569	Asia_W_Europe	H1+16239	H	H	13
2743	JQ704545.1	JQ704545.1; H3v2; Asia_W_Europe; 16; A73G; A263G; C315CC	16569	Asia_W_Europe	H3v2	H	H	16
2744	JQ704546.1	JQ704546.1; H6a1b3; Asia_W_Europe; 21; T204C; T239C; A263G	16571	Asia_W_Europe	H6a1b3	H	H	21
2745	JQ704547.1	JQ704547.1; H3v; Asia_W_Europe; 12; A263G; C315CC; T408A	16569	Asia_W_Europe	H3v	H	H	12
2746	JQ704548.1	JQ704548.1; K1a13a; Asia_W; 37; A73G; T146C; T195C	16572	Asia_W	K1a13a	K	K	37
2747	JQ704549.1	JQ704549.1; H13a1a1; Asia_W_Europe; 16; A263G; C309CCCT; T310C	16571	Asia_W_Europe	H13a1a1	H	H	16

Supplementary Table S4 Human mitochondrial database (hMITO DB v1.0) metadata[a]

Row	Name (accession no.)	Description	Size	Geo_Region	Haplogroup	Macro_ Haplo	Macro_ Haplo2	Total Variants
2748	JQ704550.1	JQ704550.1; H3a1; Asia_W_Europe; 16; T152C; T195C; A263G	16570	Asia_W_Europe	H3a1	H	H	16
2749	JQ704551.1	JQ704551.1; H1b1+16362; Asia_W_Europe; 16; A263G; C315CC; CA522d	16567	Asia_W_Europe	H1b1+16362	H	H	16
2750	JQ704552.1	JQ704552.1; H3h3a; Asia_W_Europe; 14; A263G; C315CC; A750G	16569	Asia_W_Europe	H3h3a	H	H	14
2751	JQ704553.1	JQ704553.1; H1bd; Asia_W_Europe; 12; A200G; T204C; A263G	16569	Asia_W_Europe	H1bd	H	H	12
2752	JQ704554.1	JQ704554.1; J1c2b; Asia_W; 30; A73G; G185A; A188G	16569	Asia_W	J1c2b	J	J	30
2753	JQ704555.1	JQ704555.1; U5a1c2a; Asia_W_Europe_C; 32; A73G; A183G; A263G	16576	Asia_W_Europe_C	U5a1c2a	U	U5	32
2754	JQ704556.1	JQ704556.1; H49b; Asia_W_Europe; 15; T63C; C64T; A263G	16571	Asia_W_Europe	H49b	H	H	15
2755	JQ704557.1	JQ704557.1; H1+16239; Asia_W_Europe; 13; T152C; A263G; C309CCT	16570	Asia_W_Europe	H1+16239	H	H	13
2756	JQ704558.1	JQ704558.1; H1c; Asia_W_Europe; 12; A263G; C315CC; T477C	16569	Asia_W_Europe	H1c	H	H	12
2757	JQ704559.1	JQ704559.1; H3a1a; Asia_W_Europe; 15; T152C; A263G; C309CCT	16570	Asia_W_Europe	H3a1a	H	H	15
2758	JQ704560.1	JQ704560.1; H27; Asia_W_Europe; 13; A263G; C315CC; A750G	16569	Asia_W_Europe	H27	H	H	13
2759	JQ704561.1	JQ704561.1; H10d; Asia_W_Europe; 13; A73G; A263G; C299A	16569	Asia_W_Europe	H10d	H	H	13
2760	JQ704562.1	JQ704562.1; H1z1; Asia_W_Europe; 15; A263G; C309CCCT; T310C	16571	Asia_W_Europe	H1z1	H	H	15
2761	JQ704563.1	JQ704563.1; H2a5; Asia_W_Europe; 10; A263G; C309CCT; T310C	16570	Asia_W_Europe	H2a5	H	H	10
2762	JQ704564.1	JQ704564.1; H1az; Asia_W_Europe; 13; A263G; C309CCCT; T310C	16571	Asia_W_Europe	H1az	H	H	13
2763	JQ704565.1	JQ704565.1; H2a2b5a; Asia_W_Europe; 10; A263G; C309CCT; T310C	16570	Asia_W_Europe	H2a2b5a	H	H	10
2764	JQ704566.1	JQ704566.1; H1n1a; Asia_W_Europe; 16; T146C; A263G; C309CCT	16572	Asia_W_Europe	H1n1a	H	H	16
2765	JQ704567.1	JQ704567.1; H1e2; Asia_W_Europe; 14; T146C; A263G; C315CC	16569	Asia_W_Europe	H1e2	H	H	14
2766	JQ704568.1	JQ704568.1; U5b1d1c; Asia_W_Europe_C; 31; A73G; C150T; A263G	16571	Asia_W_Europe_C	U5b1d1c	U	U5	31
2767	JQ704569.1	JQ704569.1; J1c14; Asia_W; 36; A73G; G185A; T195C	16570	Asia_W	J1c14	J	J	36
2768	JQ704570.1	JQ704570.1; H10; Asia_W_Europe; 12; A263G; C309CCCT; T310C	16573	Asia_W_Europe	H10	H	H	12
2769	JQ704571.1	JQ704571.1; H1ad; Asia_W_Europe; 12; A263G; C309CCCT; T310C	16571	Asia_W_Europe	H1ad	H	H	12
2770	JQ704572.1	JQ704572.1; U5b2b5; Asia_W_Europe_C; 37; A73G; C150T; A263G	16569	Asia_W_Europe_C	U5b2b5	U	U5	37
2771	JQ704573.1	JQ704573.1; H10b1; Asia_W_Europe; 13; A263G; C309CCT; T310C	16570	Asia_W_Europe	H10b1	H	H	13
2772	JQ704574.1	JQ704574.1; H; Asia_W_Europe; 11; A263G; C315CC; A750G	16569	Asia_W_Europe	H	H	H	11
2773	JQ704575.1	JQ704575.1; H1a1; Asia_W_Europe; 14; A73G; A263G; C315CC	16569	Asia_W_Europe	H1a1	H	H	14
2774	JQ704576.1	JQ704576.1; J1c8a; Asia_W; 30; A73G; G185A; G228A	16571	Asia_W	J1c8a	J	J	30
2775	JQ704577.1	JQ704577.1; H50; Asia_W_Europe; 11; A263G; C309CCT; T310C	16570	Asia_W_Europe	H50	H	H	11
2776	JQ704578.1	JQ704578.1; H3s; Asia_W_Europe; 16; T152C; C194T; A263G	16569	Asia_W_Europe	H3s	H	H	16
2777	JQ704579.1	JQ704579.1; H2a1a; Asia_W_Europe; 10; C315CC; CA522d(=CA514d†); A750G	16567	Asia_W_Europe	H2a1a	H	H	10
2778	JQ704580.1	JQ704580.1; H3y; Asia_W_Europe; 14; A263G; C309CCT; T310C	16570	Asia_W_Europe	H3y	H	H	14
2779	JQ704581.1	JQ704581.1; J1c12a; Asia_W; 36; A73G; G185A; A189G	16572	Asia_W	J1c12a	J	J	36
2780	JQ704582.1	JQ704582.1; K1a10a; Asia_W; 35; A73G; T195C; A263G	16573	Asia_W	K1a10a	K	K	35
2781	JQ704583.1	JQ704583.1; H3ar; Asia_W_Europe; 11; A263G; C309CC; A750G	16569	Asia_W_Europe	H3ar	H	H	11
2782	JQ704584.1	JQ704584.1; H3; Asia_W_Europe; 11; A263G; C315CC; A750G	16569	Asia_W_Europe	H3	H	H	11
2783	JQ704585.1	JQ704585.1; H1c; Asia_W_Europe; 15; A263G; C309CCT; T310C	16570	Asia_W_Europe	H1c	H	H	15
2784	JQ704586.1	JQ704586.1; B2g1; Asia_SE_E_America_N_S; 43; G62C; C64T; G66T	16562	Asia_SE_E_America_N_S	B2g1	B	B	43
2785	JQ704587.1	JQ704587.1; H1ay; Asia_W_Europe; 16; A263G; C315CC; A750G	16569	Asia_W_Europe	H1ay	H	H	16
2786	JQ704588.1	JQ704588.1; H1bg; Asia_W_Europe; 15; C150T; A263G; C315CC	16569	Asia_W_Europe	H1bg	H	H	15
2787	JQ704589.1	JQ704589.1; H1b1a; Asia_W_Europe; 17; T152C; A263G; C315CC	16569	Asia_W_Europe	H1b1a	H	H	17
2788	JQ704590.1	JQ704590.1; U5a1a1; Asia_W_Europe_C; 28; A73G; A263G; C315CC	16569	Asia_W_Europe_C	U5a1a1	U	U5	28

Supplementary Table S4 Human mitochondrial database (hMITO DB v1.0) metadata[a]

Row	Name (accession no.)	Description	Size	Geo_Region	Haplogroup	Macro_ Haplo	Macro_ Haplo2	Total Variants
2789	JQ704591.1	JQ704591.1; K1a26; Asia_W; 34; A73G; T195C; A263G	16571	Asia_W	K1a26	K	K	34
2790	JQ704592.1	JQ704592.1; H6a1b; Asia_W_Europe; 17; T239C; A263G; C309CCT	16570	Asia_W_Europe	H6a1b	H	H	17
2791	JQ704593.1	JQ704593.1; H1a; Asia_W_Europe; 14; A73G; A263G; G275A	16569	Asia_W_Europe	H1a	H	H	14
2792	JQ704594.1	JQ704594.1; T1a1; Asia_W; 37; A73G; T152C; T195C	16570	Asia_W	T1a1	T	T	37
2793	JQ704595.1	JQ704595.1; H3h2; Asia_W_Europe; 13; A263G; C315CC; A750G	16569	Asia_W_Europe	H3h2	H	H	13
2794	JQ704596.1	JQ704596.1; T2b4f; Asia_W; 37; A73G; T152C; A263G	16570	Asia_W	T2b4f	T	T	37
2795	JQ704597.1	JQ704597.1; T2b4; Asia_W; 35; A73G; A263G; C309CCT	16570	Asia_W	T2b4	T	T	35
2796	JQ704598.1	JQ704598.1; U5b1+16189+@16192; Asia_W_Europe_C; 29; A73G; C150T; A263	16570	Asia_W_Europe_C	U5b1+16189+@	U	U5	29
2797	JQ704599.1	JQ704599.1; D1m; Asia_E_America_N_S; 38; A73G; A263G; C315CC	16567	Asia_E_America_N_S	D1m	D	D	38
2798	JQ704600.1	JQ704600.1; J2a1a1; Asia_W; 40; A73G; C150T; T152C	16570	Asia_W	J2a1a1	J	J	40
2799	JQ704601.1	JQ704601.1; H1a1; Asia_W_Europe; 14; A73G; A263G; C315CC	16569	Asia_W_Europe	H1a1	H	H	14
2800	JQ704602.1	JQ704602.1; V10a; Europe_S; 18; T72C; A263G; C309CCT	16570	Europe_S	V10a	V	V	18
2801	JQ704603.1	JQ704603.1; H5a3; Asia_W_Europe; 16; A263G; C309CCT; T310C	16570	Asia_W_Europe	H5a3	H	H	16
2802	JQ704604.1	JQ704604.1; J1c8a; Asia_W; 29; A73G; G185A; G228A	16570	Asia_W	J1c8a	J	J	29
2803	JQ704605.1	JQ704605.1; H7c2; Asia_W_Europe; 12; A263G; C309CCCT; T310C	16571	Asia_W_Europe	H7c2	H	H	12
2804	JQ704606.1	JQ704606.1; H1br; Asia_W_Europe; 13; A73G; A263G; C309CCT	16570	Asia_W_Europe	H1br	H	H	13
2805	JQ704607.1	JQ704607.1; H7e; Asia_W_Europe; 15; A263G; C309CCCT; T310C	16571	Asia_W_Europe	H7e	H	H	15
2806	JQ704608.1	JQ704608.1; H6a1a2a; Asia_W_Europe; 18; C41T; C150T; T239C	16571	Asia_W_Europe	H6a1a2a	H	H	18
2807	JQ704609.1	JQ704609.1; J1b1a1; Asia_W; 36; A73G; C242T; A263G	16570	Asia_W	J1b1a1	J	J	36
2808	JQ704610.1	JQ704610.1; HV0d; Asia_W; 17; C64T; T72C; T195C	16570	Asia_W	HV0d	HV	HV	17
2809	JQ704611.1	JQ704611.1; H3h2; Asia_W_Europe; 12; A263G; C315CC; A750G	16569	Asia_W_Europe	H3h2	H	H	12
2810	JQ704612.1	JQ704612.1; H1a3a1; Asia_W_Europe; 15; A73G; A263G; C309CCT	16570	Asia_W_Europe	H1a3a1	H	H	15
2811	JQ704613.1	JQ704613.1; H1n6; Asia_W_Europe; 18; T195C; A263G; C315CC	16571	Asia_W_Europe	H1n6	H	H	18
2812	JQ704614.1	JQ704614.1; H6a1a; Asia_W_Europe; 17; T239C; A263G; C309CCT	16570	Asia_W_Europe	H6a1a	H	H	17
2813	JQ704615.1	JQ704615.1; J1c3a2; Asia_W; 28; A73G; A263G; C295T	16569	Asia_W	J1c3a2	J	J	28
2814	JQ704616.1	JQ704616.1; U5a1f1a1; Asia_W_Europe_C; 34; A73G; T199C; A263G	16571	Asia_W_Europe_C	U5a1f1a1	U	U5	34
2815	JQ704617.1	JQ704617.1; T2a1a2; Asia_W; 36; A73G; A263G; C315CC	16569	Asia_W	T2a1a2	T	T	36
2816	JQ704618.1	JQ704618.1; H1c3b; Asia_W_Europe; 18; A257G; A263G; C309CCCT	16571	Asia_W_Europe	H1c3b	H	H	18
2817	JQ704619.1	JQ704619.1; H+152; Asia_W_Europe; 15; T152C; A263G; C309CCCT	16573	Asia_W_Europe	H+152	H	H	15
2818	JQ704620.1	JQ704620.1; H+16129; Asia_W_Europe; 16; A263G; C309CCT; T310C	16570	Asia_W_Europe	H+16129	H	H	16
2819	JQ704621.1	JQ704621.1; H6a1b2; Asia_W_Europe; 17; T239C; A263G; C309CCT	16570	Asia_W_Europe	H6a1b2	H	H	17
2820	JQ704622.1	JQ704622.1; H3a1a; Asia_W_Europe; 15; T152C; A263G; C309CCCT	16571	Asia_W_Europe	H3a1a	H	H	15
2821	JQ704623.1	JQ704623.1; V; Europe_S; 15; T72C; A263G; C309CCT	16570	Europe_S	V	V	V	15
2822	JQ704624.1	JQ704624.1; H1b; Asia_W_Europe; 15; T199C; A263G; C315CC	16569	Asia_W_Europe	H1b	H	H	15
2823	JQ704625.1	JQ704625.1; K2a; Asia_W; 32; A73G; T146C; T152C	16570	Asia_W	K2a	K	K	32
2824	JQ704626.1	JQ704626.1; J1b1a1+146; Asia_W; 37; A73G; T146C; C242T	16570	Asia_W	J1b1a1+146	J	J	37
2825	JQ704627.1	JQ704627.1; U2e2a1c; Asia_S_W_Europe; 45; A73G; T146C; T152C	16570	Asia_S_W_Europe	U2e2a1c	U	U2	45
2826	JQ704628.1	JQ704628.1; H; Asia_W_Europe; 9; A263G; C315CC; A750G	16569	Asia_W_Europe	H	H	H	9
2827	JQ704629.1	JQ704629.1; H7d1; Asia_W_Europe; 13; A263G; C315CC; A750G	16569	Asia_W_Europe	H7d1	H	H	13
2828	JQ704630.1	JQ704630.1; H1q; Asia_W_Europe; 11; T204C; A263G; C315CC	16569	Asia_W_Europe	H1q	H	H	11
2829	JQ704631.1	JQ704631.1; H31; Asia_W_Europe; 13; T146C; T195C; A263G	16569	Asia_W_Europe	H31	H	H	13

Supplementary Table S4 Human mitochondrial database (hMITO DB v1.0) metadata[a]

Row	Name (accession no.)	Description	Size	Geo_Region	Haplogroup	Macro_ Haplo	Macro_ Haplo2	Total Variants
2830	JQ704632.1	JQ704632.1; H3i1; Asia_W_Europe; 14; T152C; A263G; C315CC	16569	Asia_W_Europe	H3i1	H	H	14
2831	JQ704633.1	JQ704633.1; H3n; Asia_W_Europe; 15; A263G; C309CCT; T310C	16570	Asia_W_Europe	H3n	H	H	15
2832	JQ704634.1	JQ704634.1; V1b; Europe_S; 21; T72C; T217C; A263G	16570	Europe_S	V1b	V	V	21
2833	JQ704635.1	JQ704635.1; H3b1b1; Asia_W_Europe; 18; A263G; C309CCT; T310C	16570	Asia_W_Europe	H3b1b1	H	H	18
2834	JQ704636.1	JQ704636.1; H1b1+16362; Asia_W_Europe; 16; A263G; C309CCT; T310C	16568	Asia_W_Europe	H1b1+16362	H	H	16
2835	JQ704637.1	JQ704637.1; H1c1; Asia_W_Europe; 13; A263G; C315CC; T477C	16569	Asia_W_Europe	H1c1	H	H	13
2836	JQ704638.1	JQ704638.1; H3aa; Asia_W_Europe; 11; A263G; C309CCT; T310C	16570	Asia_W_Europe	H3aa	H	H	11
2837	JQ704639.1	JQ704639.1; H1c1; Asia_W_Europe; 13; A263G; C315CC; T477C	16569	Asia_W_Europe	H1c1	H	H	13
2838	JQ704640.1	JQ704640.1; H79; Asia_W_Europe; 13; A263G; C309CCCT; T310C	16571	Asia_W_Europe	H79	H	H	13
2839	JQ704641.1	JQ704641.1; H3af; Asia_W_Europe; 12; A263G; C309CCT; T310C	16570	Asia_W_Europe	H3af	H	H	12
2840	JQ704642.1	JQ704642.1; A2f3; Asia_NE_America_N; 39; C64T; A73G; T146C	16567	Asia_NE_America_N	A2f3	A	A	39
2841	JQ704643.1	JQ704643.1; T2b7a2; Asia_W; 39; A73G; A263G; C309CCCT	16571	Asia_W	T2b7a2	T	T	39
2842	JQ704644.1	JQ704644.1; H27f; Asia_W_Europe; 13; A263G; C315CC; A750G	16569	Asia_W_Europe	H27f	H	H	13
2843	JQ704645.1	JQ704645.1; U5a2a1d; Asia_W_Europe_C; 33; A73G; A263G; C309CCT	16570	Asia_W_Europe_C	U5a2a1d	U	U5	33
2844	JQ704646.1	JQ704646.1; H7f; Asia_W_Europe; 13; A263G; C315CC; A750G	16569	Asia_W_Europe	H7f	H	H	13
2845	JQ704647.1	JQ704647.1; H1bh; Asia_W_Europe; 13; A263G; C309CCT; T310C	16570	Asia_W_Europe	H1bh	H	H	13
2846	JQ704648.1	JQ704648.1; H4a1d; Asia_W_Europe; 20; A263G; C309CCT; T310C	16570	Asia_W_Europe	H4a1d	H	H	20
2847	JQ704649.1	JQ704649.1; H3af; Asia_W_Europe; 12; A263G; C315CC; A750G	16569	Asia_W_Europe	H3af	H	H	12
2848	JQ704650.1	JQ704650.1; H5a1g1a; Asia_W_Europe; 19; T152C; A263G; C315CC	16567	Asia_W_Europe	H5a1g1a	H	H	19
2849	JQ704651.1	JQ704651.1; H6a1b3; Asia_W_Europe; 18; T204C; T239C; A263G	16569	Asia_W_Europe	H6a1b3	H	H	18
2850	JQ704652.1	JQ704652.1; H5a1g1a; Asia_W_Europe; 18; A263G; C315CC; A444G	16567	Asia_W_Europe	H5a1g1a	H	H	18
2851	JQ704653.1	JQ704653.1; H6a1b2; Asia_W_Europe; 16; T152C; T239C; A263G	16570	Asia_W_Europe	H6a1b2	H	H	16
2852	JQ704654.1	JQ704654.1; K1a1b1a; Asia_W; 37; A73G; C114T; A263G	16569	Asia_W	K1a1b1a	K	K	37
2853	JQ704655.1	JQ704655.1; H1n1; Asia_W_Europe; 15; T146C; T152C; A215G	16569	Asia_W_Europe	H1n1	H	H	15
2854	JQ704656.1	JQ704656.1; H1e1b1b; Asia_W_Europe; 15; A263G; C315CC; T453C	16569	Asia_W_Europe	H1e1b1b	H	H	15
2855	JQ704657.1	JQ704657.1; H1ag1; Asia_W_Europe; 13; A263G; C309CCCT; T310C	16571	Asia_W_Europe	H1ag1	H	H	13
2856	JQ704658.1	JQ704658.1; U5a1a1c; Asia_W_Europe_C; 30; A73G; A263G; C315CC	16569	Asia_W_Europe_C	U5a1a1c	U	U5	30
2857	JQ704659.1	JQ704659.1; H1; Asia_W_Europe; 10; A263G; T279C; C315CC	16569	Asia_W_Europe	H1	H	H	10
2858	JQ704660.1	JQ704660.1; V7b; Europe_S; 20; T72C; A93G; A263G	16570	Europe_S	V7b	V	V	20
2859	JQ704661.1	JQ704661.1; U4c1; Asia_N_Europe_N; 35; A73G; C150T; T195C	16575	Asia_N_Europe_N	U4c1	U	U4	35
2860	JQ704662.1	JQ704662.1; K2a6; Asia_W; 35; A73G; T146C; T152C	16570	Asia_W	K2a6	K	K	35
2861	JQ704663.1	JQ704663.1; K1a+150; Asia_W; 33; A73G; C150T; T152C	16569	Asia_W	K1a+150	K	K	33
2862	JQ704664.1	JQ704664.1; H11b1; Asia_W_Europe; 18; T146C; T195C; A263G	16570	Asia_W_Europe	H11b1	H	H	18
2863	JQ704665.1	JQ704665.1; J1c3f; Asia_W; 31; A73G; A263G; C295T	16569	Asia_W	J1c3f	J	J	31
2864	JQ704666.1	JQ704666.1; H; Asia_W_Europe; 10; A73G; A263G; C315CC	16569	Asia_W_Europe	H	H	H	10
2865	JQ704667.1	JQ704667.1; H1q; Asia_W_Europe; 16; A73G; A263G; C315CC	16569	Asia_W_Europe	H1q	H	H	16
2866	JQ704668.1	JQ704668.1; L2a1f; Africa_W_C; 54; A73G; T146C; T152C	16570	Africa_W_C	L2a1f	L2	L2	54
2867	JQ704669.1	JQ704669.1; H1ap1; Asia_W_Europe; 14; T152C; A263G; C309CCT	16570	Asia_W_Europe	H1ap1	H	H	14
2868	JQ704670.1	JQ704670.1; L3f1b1a; Africa_E; 45; A73G; A189G; A200G	16569	Africa_E	L3f1b1a	L3	L3	45
2869	JQ704671.1	JQ704671.1; H1ar1; Asia_W_Europe; 15; T152C; A183G; A263G	16570	Asia_W_Europe	H1ar1	H	H	15
2870	JQ704672.1	JQ704672.1; H24b; Asia_W_Europe; 12; A263G; C309CCT; T310C	16570	Asia_W_Europe	H24b	H	H	12

Supplementary Table S4 Human mitochondrial database (hMITO DB v1.0) metadata[a]

Row	Name (accession no.)	Description	Size	Geo_Region	Haplogroup	Macro_ Haplo	Macro_ Haplo2	Total Variants
2871	JQ704673.1	JQ704673.1; H2a2b5a; Asia_W_Europe; 12; A263G; C309CCT; T310C	16570	Asia_W_Europe	H2a2b5a	H	H	12
2872	JQ704674.1	JQ704674.1; H3c1; Asia_W_Europe; 16; A189G; A263G; C315CC	16569	Asia_W_Europe	H3c1	H	H	16
2873	JQ704675.1	JQ704675.1; U4c1; Asia_N_Europe_N; 38; A73G; T195C; A263G	16572	Asia_N_Europe_N	U4c1	U	U4	38
2874	JQ704676.1	JQ704676.1; U4b1b2; Asia_N_Europe_N; 33; A73G; T152C; T195C	16571	Asia_N_Europe_N	U4b1b2	U	U4	33
2875	JQ704677.1	JQ704677.1; H57; Asia_W_Europe; 14; C64T; A263G; C309CCT	16569	Asia_W_Europe	H57	H	H	14
2876	JQ704678.1	JQ704678.1; H3ap; Asia_W_Europe; 13; C150T; A263G; C309CCT	16570	Asia_W_Europe	H3ap	H	H	13
2877	JQ704679.1	JQ704679.1; U5a1b1e; Asia_W_Europe_C; 30; A73G; A263G; C309CCT	16570	Asia_W_Europe_C	U5a1b1e	U	U5	30
2878	JQ704680.1	JQ704680.1; H16b; Asia_W_Europe; 12; A263G; C315CC; A750G	16569	Asia_W_Europe	H16b	H	H	12
2879	JQ704681.1	JQ704681.1; B2a1a1; Asia_SE_E_America_N_S; 30; A73G; A263G; C315CC	16560	Asia_SE_E_America_N_S	B2a1a1	B	B	30
2880	JQ704682.1	JQ704682.1; H13a1a1; Asia_W_Europe; 14; A263G; C309CCCT; T310C	16571	Asia_W_Europe	H13a1a1	H	H	14
2881	JQ704683.1	JQ704683.1; L1b1a17; Africa_C; 82; A73G; T152C; C182T	16569	Africa_C	L1b1a17	L1	L1	82
2882	JQ704684.1	JQ704684.1; H4a1a1a; Asia_W_Europe; 17; A73G; A263G; C309CCT	16568	Asia_W_Europe	H4a1a1a	H	H	17
2883	JQ704685.1	JQ704685.1; H1c4b1; Asia_W_Europe; 13; A263G; C315CC; T477C	16569	Asia_W_Europe	H1c4b1	H	H	13
2884	JQ704686.1	JQ704686.1; H2a2b; Asia_W_Europe; 9; A263G; C309CCT; T310C	16570	Asia_W_Europe	H2a2b	H	H	9
2885	JQ704687.1	JQ704687.1; H2a5b1; Asia_W_Europe; 10; A249d; A263G; C309CCCT	16570	Asia_W_Europe	H2a5b1	H	H	10
2886	JQ704688.1	JQ704688.1; T2b4b; Asia_W; 38; A73G; T152C; A263G	16570	Asia_W	T2b4b	T	T	38
2887	JQ704689.1	JQ704689.1; H52; Asia_W_Europe; 12; T152C; A263G; C309CCT	16570	Asia_W_Europe	H52	H	H	12
2888	JQ704690.1	JQ704690.1; I1a1b; Asia_W_SW; 42; A73G; T199C; G203A	16572	Asia_W_SW	I1a1b	I	I	42
2889	JQ704691.1	JQ704691.1; H7a1; Asia_W_Europe; 14; C194T; A263G; C309CCT	16570	Asia_W_Europe	H7a1	H	H	14
2890	JQ704692.1	JQ704692.1; H14a2c; Asia_W_Europe; 18; A263G; C309CCCT; T310C	16571	Asia_W_Europe	H14a2c	H	H	18
2891	JQ704693.1	JQ704693.1; H1e1a; Asia_W_Europe; 17; T146C; A263G; C315CC	16569	Asia_W_Europe	H1e1a	H	H	17
2892	JQ704694.1	JQ704694.1; H; Asia_W_Europe; 13; C151T; A263G; C315CC	16569	Asia_W_Europe	H	H	H	13
2893	JQ704695.1	JQ704695.1; U5a1a1d1; Asia_W_Europe_C; 32; A73G; G185A; A189G	16569	Asia_W_Europe_C	U5a1a1d1	U	U5	32
2894	JQ704696.1	JQ704696.1; K1a1a2; Asia_W; 35; A73G; A263G; C315CC	16569	Asia_W	K1a1a2	K	K	35
2895	JQ704697.1	JQ704697.1; T2b25; Asia_W; 36; A73G; A263G; C315CCC	16570	Asia_W	T2b25	T	T	36
2896	JQ704698.1	JQ704698.1; H2a2a1a; Asia_W_Europe; 4; C315CC; T7220C; G15314A	16569	Asia_W_Europe	H2a2a1a	H	H	4
2897	JQ704699.1	JQ704699.1; T2b19; Asia_W; 36; A73G; A263G; C315CC	16567	Asia_W	T2b19	T	T	36
2898	JQ704700.1	JQ704700.1; U5a1d2a; Asia_W_Europe_C; 34; A73G; T195C; A263G	16571	Asia_W_Europe_C	U5a1d2a	U	U5	34
2899	JQ704701.1	JQ704701.1; U5a1a2b; Asia_W_Europe_C; 31; A73G; A263G; C309CCT	16571	Asia_W_Europe_C	U5a1a2b	U	U5	31
2900	JQ704702.1	JQ704702.1; H1g1; Asia_W_Europe; 13; A263G; C309CCCT; T310C	16571	Asia_W_Europe	H1g1	H	H	13
2901	JQ704703.1	JQ704703.1; V20; Europe_S; 18; T72C; A263G; C315CC	16569	Europe_S	V20	V	V	18
2902	JQ704704.1	JQ704704.1; H3k; Asia_W_Europe; 13; T152C; A263G; C309CCCT	16571	Asia_W_Europe	H3k	H	H	13
2903	JQ704705.1	JQ704705.1; I2; Asia_W_SW; 36; A73G; T152C; T199C	16572	Asia_W_SW	I2	I	I	36
2904	JQ704706.1	JQ704706.1; H5e1a; Asia_W_Europe; 14; A263G; C315CC; C456T	16569	Asia_W_Europe	H5e1a	H	H	14
2905	JQ704707.1	JQ704707.1; K2a3; Asia_W; 33; A73G; T146C; T152C	16570	Asia_W	K2a3	K	K	33
2906	JQ704708.1	JQ704708.1; U5a1a1; Asia_W_Europe_C; 26; A73G; A263G; C315CC	16569	Asia_W_Europe_C	U5a1a1	U	U5	26
2907	JQ704709.1	JQ704709.1; H1b1+16362; Asia_W_Europe; 18; A263G; C309CCCT; T310C	16569	Asia_W_Europe	H1b1+16362	H	H	18
2908	JQ704710.1	JQ704710.1; J1c2b1; Asia_W; 35; A73G; G185A; A188G	16570	Asia_W	J1c2b1	J	J	35
2909	JQ704711.1	JQ704711.1; H7d2; Asia_W_Europe; 14; A263G; A291AA; C309CCT	16571	Asia_W_Europe	H7d2	H	H	14
2910	JQ704712.1	JQ704712.1; H7d; Asia_W_Europe; 12; A263G; C309CCT; T310C	16570	Asia_W_Europe	H7d	H	H	12
2911	JQ704713.1	JQ704713.1; I5a1b; Asia_W_SW; 38; A73G; T199C; T250C	16562	Asia_W_SW	I5a1b	I	I	38

Supplementary Table S4 Human mitochondrial database (hMITO DB v1.0) metadata[a]

Row	Name (accession no.)	Description	Size	Geo_Region	Haplogroup	Macro_ Haplo	Macro_ Haplo2	Total Variants
2912	JQ704714.1	JQ704714.1; H7c2; Asia_W_Europe; 13; A263G; C309CCCT; T310C	16571	Asia_W_Europe	H7c2	H	H	13
2913	JQ704715.1	JQ704715.1; H1ab; Asia_W_Europe; 14; A263G; C315CC; A750G	16569	Asia_W_Europe	H1ab	H	H	14
2914	JQ704716.1	JQ704716.1; H5g; Asia_W_Europe; 14; T146C; A263G; C309CCCT	16573	Asia_W_Europe	H5g	H	H	14
2915	JQ704717.1	JQ704717.1; H1h2; Asia_W_Europe; 13; A263G; C315CC; A750G	16569	Asia_W_Europe	H1h2	H	H	13
2916	JQ704718.1	JQ704718.1; H1c3; Asia_W_Europe; 14; A257G; A263G; C315CC	16569	Asia_W_Europe	H1c3	H	H	14
2917	JQ704719.1	JQ704719.1; H1c1; Asia_W_Europe; 13; A263G; C315CC; T477C	16569	Asia_W_Europe	H1c1	H	H	13
2918	JQ704720.1	JQ704720.1; H1q; Asia_W_Europe; 12; A263G; C315CC; A750G	16569	Asia_W_Europe	H1q	H	H	12
2919	JQ704721.1	JQ704721.1; H1c; Asia_W_Europe; 11; A263G; C315CC; T477C	16569	Asia_W_Europe	H1c	H	H	11
2920	JQ704722.1	JQ704722.1; H3g4; Asia_W_Europe; 16; T152C; A263G; C309CCT	16570	Asia_W_Europe	H3g4	H	H	16
2921	JQ704723.1	JQ704723.1; U5a1a1b; Asia_W_Europe_C; 29; A73G; T152C; A263G	16570	Asia_W_Europe_C	U5a1a1b	U	U5	29
2922	JQ704724.1	JQ704724.1; K1d; Asia_W; 36; A73G; T195C; A263G	16574	Asia_W	K1d	K	K	36
2923	JQ704725.1	JQ704725.1; U5a1a1b; Asia_W_Europe_C; 29; A73G; T152C; A263G	16569	Asia_W_Europe_C	U5a1a1b	U	U5	29
2924	JQ704726.1	JQ704726.1; U5b2a1a+16311; Asia_W_Europe_C; 29; A73G; C150T; A263G	16570	Asia_W_Europe_C	U5b2a1a+1631:	U	U5	29
2925	JQ704727.1	JQ704727.1; K1a4a1; Asia_W; 35; A73G; A200G; A263G	16571	Asia_W	K1a4a1	K	K	35
2926	JQ704728.1	JQ704728.1; L3e1f; Africa_E; 39; A73G; C150T; A189G	16563	Africa_E	L3e1f	L3	L3	39
2927	JQ704729.1	JQ704729.1; K1c2; Asia_W; 35; A73G; T146C; T152C	16568	Asia_W	K1c2	K	K	35
2928	JQ704730.1	JQ704730.1; H5a1f; Asia_W_Europe; 14; A263G; C315CC; C456T	16567	Asia_W_Europe	H5a1f	H	H	14
2929	JQ704731.1	JQ704731.1; H1; Asia_W_Europe; 12; A263G; C315CC; A750G	16569	Asia_W_Europe	H1	H	H	12
2930	JQ704732.1	JQ704732.1; T1a1g; Asia_W; 37; A73G; T152C; T195C	16569	Asia_W	T1a1g	T	T	37
2931	JQ704733.1	JQ704733.1; H3m; Asia_W_Europe; 13; A263G; C315CC; A750G	16569	Asia_W_Europe	H3m	H	H	13
2932	JQ704734.1	JQ704734.1; U5a1b1; Asia_W_Europe_C; 29; A73G; C150T; A263G	16569	Asia_W_Europe_C	U5a1b1	U	U5	29
2933	JQ704735.1	JQ704735.1; T1a1; Asia_W; 35; A73G; T152C; T195C	16570	Asia_W	T1a1	T	T	35
2934	JQ704736.1	JQ704736.1; J1c5a1; Asia_W; 32; A73G; G185A; G228A	16569	Asia_W	J1c5a1	J	J	32
2935	JQ704737.1	JQ704737.1; K1c2; Asia_W; 38; A73G; T146C; C150T	16569	Asia_W	K1c2	K	K	38
2936	JQ704738.1	JQ704738.1; T2b4a1; Asia_W; 36; A73G; A263G; C309CCT	16570	Asia_W	T2b4a1	T	T	36
2937	JQ704739.1	JQ704739.1; H27a; Asia_W_Europe; 13; A263G; C315CC; A750G	16569	Asia_W_Europe	H27a	H	H	13
2938	JQ704740.1	JQ704740.1; L2c2a1; Africa_W_C; 62; A73G; A93G; T146C	16568	Africa_W_C	L2c2a1	L2	L2	62
2939	JQ704741.1	JQ704741.1; U4d1a1a; Asia_N_Europe_N; 35; A73G; T195C; A263G	16573	Asia_N_Europe_N	U4d1a1a	U	U4	35
2940	JQ704742.1	JQ704742.1; T2b4c; Asia_W; 37; A73G; T152C; A263G	16572	Asia_W	T2b4c	T	T	37
2941	JQ704743.1	JQ704743.1; H1bb; Asia_W_Europe; 14; T152C; C194T; T199C	16569	Asia_W_Europe	H1bb	H	H	14
2942	JQ704744.1	JQ704744.1; H1b3; Asia_W_Europe; 16; A263G; C315CC; A750G	16569	Asia_W_Europe	H1b3	H	H	16
2943	JQ704745.1	JQ704745.1; J1c3a1; Asia_W; 32; A73G; G228A; A263G	16570	Asia_W	J1c3a1	J	J	32
2944	JQ704746.1	JQ704746.1; T2b4; Asia_W; 36; A73G; T217C; A263G	16569	Asia_W	T2b4	T	T	36
2945	JQ704747.1	JQ704747.1; H54; Asia_W_Europe; 14; T72C; C182T; A263G	16569	Asia_W_Europe	H54	H	H	14
2946	JQ704748.1	JQ704748.1; H3b1a; Asia_W_Europe; 17; A263G; C309CCT; T310C	16570	Asia_W_Europe	H3b1a	H	H	17
2947	JQ704749.1	JQ704749.1; U6a1a2; Asia_SW_Africa_N; 33; A73G; T152C; G251A	16569	Asia_SW_Africa_N	U6a1a2	U	U6	33
2948	JQ704750.1	JQ704750.1; H5; Asia_W_Europe; 14; A263G; C309CCT; T310C	16570	Asia_W_Europe	H5	H	H	14
2949	JQ704751.1	JQ704751.1; J1c10; Asia_W; 32; A73G; G228A; A263G	16570	Asia_W	J1c10	J	J	32
2950	JQ704752.1	JQ704752.1; V7a; Europe_S; 23; T72C; T89C; A93G	16570	Europe_S	V7a	V	V	23
2951	JQ704753.1	JQ704753.1; H3+152; Asia_W_Europe; 12; T152C; A263G; C309CCT	16570	Asia_W_Europe	H3+152	H	H	12
2952	JQ704754.1	JQ704754.1; X2b7; Asia_W_America_N; 34; A73G; A153G; T195C	16570	Asia_W_America_N	X2b7	X	X	34

Supplementary Table S4 Human mitochondrial database (hMITO DB v1.0) metadata[a]

Row	Name (accession no.)	Description	Size	Geo_Region	Haplogroup	Macro_ Haplo	Macro_ Haplo2	Total Variants
2953	JQ704755.1	JQ704755.1; H27b; Asia_W_Europe; 15; A263G; C315CC; CA522d	16567	Asia_W_Europe	H27b	H	H	15
2954	JQ704756.1	JQ704756.1; H1bj; Asia_W_Europe; 12; A263G; C309CCT; T310C	16570	Asia_W_Europe	H1bj	H	H	12
2955	JQ704757.1	JQ704757.1; H23; Asia_W_Europe; 10; A263G; C315CC; A750G	16569	Asia_W_Europe	H23	H	H	10
2956	JQ704758.1	JQ704758.1; H10e; Asia_W_Europe; 12; A263G; C309CCT; T310C	16570	Asia_W_Europe	H10e	H	H	12
2957	JQ704759.1	JQ704759.1; K1a4a1g; Asia_W; 36; A73G; A263G; C309CCT	16570	Asia_W	K1a4a1g	K	K	36
2958	JQ704760.1	JQ704760.1; K1a4d; Asia_W; 36; A73G; A153G; A263G	16572	Asia_W	K1a4d	K	K	36
2959	JQ704761.1	JQ704761.1; K1a4e; Asia_W; 35; A73G; A263G; C309CCT	16570	Asia_W	K1a4e	K	K	35
2960	JQ704762.1	JQ704762.1; T2; Asia_W; 30; A73G; A263G; C309CCT	16570	Asia_W	T2	T	T	30
2961	JQ704763.1	JQ704763.1; M1a1a1; Africa_E_Nile Valley; 44; A73G; T195C; A263G	16571	Africa_E_Nile Valley	M1a1a1	M	M1	44
2962	JQ704764.1	JQ704764.1; K1a4f1; Asia_W; 34; A73G; A263G; C315CC	16569	Asia_W	K1a4f1	K	K	34
2963	JQ704765.1	JQ704765.1; I2; Asia_W_SW; 36; A73G; T152C; T199C	16572	Asia_W_SW	I2	I	I	36
2964	JQ704766.1	JQ704766.1; H5a1q; Asia_W_Europe; 14; A263G; C309CCCT; T310C	16569	Asia_W_Europe	H5a1q	H	H	14
2965	JQ704767.1	JQ704767.1; V+@72; Europe_S; 14; A263G; C309CCT; T310C	16570	Europe_S	V+@72	V	V	14
2966	JQ704768.1	JQ704768.1; I5a1c; Asia_W_SW; 39; A73G; T199C; T204C	16563	Asia_W_SW	I5a1c	I	I	39
2967	JQ704769.1	JQ704769.1; U8a1a1; Asia_W_Europe ; 31; A73G; A263G; T282C	16570	Asia_W_Europe	U8a1a1	U	U8	31
2968	JQ704770.1	JQ704770.1; K1a4a1; Asia_W; 37; A73G; C150T; A263G	16573	Asia_W	K1a4a1	K	K	37
2969	JQ704771.1	JQ704771.1; U5b2a6; Asia_W_Europe_C; 29; A73G; C150T; A263G	16570	Asia_W_Europe_C	U5b2a6	U	U5	29
2970	JQ704772.1	JQ704772.1; U4a2e; Asia_N_Europe_N; 33; A73G; T195C; A263G	16570	Asia_N_Europe_N	U4a2e	U	U4	33
2971	JQ704773.1	JQ704773.1; U2e1b1; Asia_S_W_Europe; 39; A73G; T152C; T217C	16572	Asia_S_W_Europe	U2e1b1	U	U2	39
2972	JQ704774.1	JQ704774.1; H2a2b; Asia_W_Europe; 8; A200G; A263G; C309CCT	16570	Asia_W_Europe	H2a2b	H	H	8
2973	JQ704775.1	JQ704775.1; U8a1a1; Asia_W_Europe ; 31; A73G; A263G; T282C	16570	Asia_W_Europe	U8a1a1	U	U8	31
2974	JQ704776.1	JQ704776.1; H1c8; Asia_W_Europe; 14; A263G; C315CC; T477C	16569	Asia_W_Europe	H1c8	H	H	14
2975	JQ704777.1	JQ704777.1; J1b1a1+146; Asia_W; 39; A73G; T146C; C242T	16569	Asia_W	J1b1a1+146	J	J	39
2976	JQ704778.1	JQ704778.1; U5b1b1+@16192; Asia_W_Europe_C; 28; A73G; C150T; A263G	16570	Asia_W_Europe_C	U5b1b1+@161[illegible]	U	U5	28
2977	JQ704779.1	JQ704779.1; J1c3g; Asia_W; 28; A73G; A263G; C295T	16569	Asia_W	J1c3g	J	J	28
2978	JQ704780.1	JQ704780.1; T2b; Asia_W; 37; A73G; G228A; A263G	16568	Asia_W	T2b	T	T	37
2979	JQ704781.1	JQ704781.1; H1bu; Asia_W_Europe; 11; A263G; C315CC; A750G	16569	Asia_W_Europe	H1bu	H	H	11
2980	JQ704782.1	JQ704782.1; H3g1; Asia_W_Europe; 14; T152C; A263G; C315CC	16569	Asia_W_Europe	H3g1	H	H	14
2981	JQ704783.1	JQ704783.1; T2b17a; Asia_W; 40; A73G; T152C; A263G	16570	Asia_W	T2b17a	T	T	40
2982	JQ704784.1	JQ704784.1; H1b1; Asia_W_Europe; 16; A263G; C309CCCT; T310C	16569	Asia_W_Europe	H1b1	H	H	16
2983	JQ704785.1	JQ704785.1; T2b6b; Asia_W; 43; A73G; T146C; A263G	16570	Asia_W	T2b6b	T	T	43
2984	JQ704786.1	JQ704786.1; T2j; Asia_W; 39; A73G; A249d; A263G	16569	Asia_W	T2j	T	T	39
2985	JQ704787.1	JQ704787.1; W1a; Asia_W; 35; A73G; A189G; T195C	16570	Asia_W	W1a	W	W	35
2986	JQ704788.1	JQ704788.1; T2b23a; Asia_W; 44; A73G; A263G; C309CCT	16570	Asia_W	T2b23a	T	T	44
2987	JQ704789.1	JQ704789.1; A2ac1; Asia_NE_America_N; 40; C64T; A73G; T146C	16568	Asia_NE_America_N	A2ac1	A	A	40
2988	JQ704790.1	JQ704790.1; W1c1; Asia_W; 39; A73G; T119C; A189G	16569	Asia_W	W1c1	W	W	39
2989	JQ704791.1	JQ704791.1; J1b1a1; Asia_W; 35; A73G; C242T; A263G	16569	Asia_W	J1b1a1	J	J	35
2990	JQ704792.1	JQ704792.1; H70; Asia_W_Europe; 11; A73G; A263G; C315CC	16569	Asia_W_Europe	H70	H	H	11
2991	JQ704793.1	JQ704793.1; H6a1b3a; Asia_W_Europe; 22; T204C; T239C; A263G	16571	Asia_W_Europe	H6a1b3a	H	H	22
2992	JQ704794.1	JQ704794.1; H1bm; Asia_W_Europe; 12; A263G; C309CCT; T310C	16570	Asia_W_Europe	H1bm	H	H	12
2993	JQ704795.1	JQ704795.1; H14b2a; Asia_W_Europe; 18; T152C; A197G; A263G	16572	Asia_W_Europe	H14b2a	H	H	18

Supplementary Table S4 Human mitochondrial database (hMITO DB v1.0) metadata[a]

Row	Name (accession no.)	Description	Size	Geo_Region	Haplogroup	Macro_ Haplo	Macro_ Haplo2	Total Variants
2994	JQ704796.1	JQ704796.1; U5a1a1e; Asia_W_Europe_C; 28; T55C; A73G; A263G	16569	Asia_W_Europe_C	U5a1a1e	U	U5	28
2995	JQ704797.1	JQ704797.1; T2a1a6; Asia_W; 37; A73G; A263G; C315CC	16569	Asia_W	T2a1a6	T	T	37
2996	JQ704798.1	JQ704798.1; H14b; Asia_W_Europe; 13; A263G; C309CCCT; T310C	16571	Asia_W_Europe	H14b	H	H	13
2997	JQ704799.1	JQ704799.1; HV9b; Asia_W; 15; A263G; C309CCT; T310C	16570	Asia_W	HV9b	HV	HV	15
2998	JQ704800.1	JQ704800.1; U6a3a1; Asia_SW_Africa_N; 29; A73G; A263G; C315CC	16567	Asia_SW_Africa_N	U6a3a1	U	U6	29
2999	JQ704801.1	JQ704801.1; T1a1c; Asia_W; 37; A73G; T152C; T195C	16570	Asia_W	T1a1c	T	T	37
3000	JQ704802.1	JQ704802.1; H2a2a1; Asia_W_Europe; 1; C315CC; ;	16569	Asia_W_Europe	H2a2a1	H	H	1
3001	JQ704803.1	JQ704803.1; J1c3g; Asia_W; 30; A73G; G185A; A263G	16570	Asia_W	J1c3g	J	J	30
3002	JQ704804.1	JQ704804.1; R6a2; Asia_S_SE; 27; A73G; G228A; A263G	16570	Asia_S_SE	R6a2	R	R6	27
3003	JQ704805.1	JQ704805.1; H1e1a; Asia_W_Europe; 14; A263G; C315CC; A750G	16569	Asia_W_Europe	H1e1a	H	H	14
3004	JQ704806.1	JQ704806.1; M7b1a1f; Asia_E_SE; 44; A73G; C150T; T199C	16570	Asia_E_SE	M7b1a1f	M	M7	44
3005	JQ704807.1	JQ704807.1; T2f1a; Asia_W; 41; A73G; T195C; A263G	16562	Asia_W	T2f1a	T	T	41
3006	JQ704808.1	JQ704808.1; K1a4a1; Asia_W; 36; A73G; T146C; A263G	16569	Asia_W	K1a4a1	K	K	36
3007	JQ704809.1	JQ704809.1; J1d3a2; Asia_W; 35; A73G; T152C; A263G	16569	Asia_W	J1d3a2	J	J	35
3008	JQ704810.1	JQ704810.1; H1b1+16362; Asia_W_Europe; 13; A263G; C315CC; CA522d	16567	Asia_W_Europe	H1b1+16362	H	H	13
3009	JQ704811.1	JQ704811.1; U4a2f; Asia_N_Europe_N; 34; A73G; T195C; C198T	16568	Asia_N_Europe_N	U4a2f	U	U4	34
3010	JQ704812.1	JQ704812.1; K1a1b1a; Asia_W; 36; A73G; C114T; A263G	16569	Asia_W	K1a1b1a	K	K	36
3011	JQ704813.1	JQ704813.1; H71; Asia_W_Europe; 11; A263G; C315CC; A750G	16569	Asia_W_Europe	H71	H	H	11
3012	JQ704814.1	JQ704814.1; H13a1a; Asia_W_Europe; 14; A263G; C315CC; A750G	16569	Asia_W_Europe	H13a1a	H	H	14
3013	JQ704815.1	JQ704815.1; A14; Asia_NE_America_N; 31; A73G; C151T; T152C	16567	Asia_NE_America_N	A14	A	A	31
3014	JQ704816.1	JQ704816.1; J1c5c; Asia_W; 31; A73G; G185A; G228A	16567	Asia_W	J1c5c	J	J	31
3015	JQ704817.1	JQ704817.1; U2e1a1c; Asia_S_W_Europe; 42; A73G; T152C; T217C	16572	Asia_S_W_Europe	U2e1a1c	U	U2	42
3016	JQ704818.1	JQ704818.1; T2e; Asia_W; 34; A73G; C150T; A263G	16570	Asia_W	T2e	T	T	34
3017	JQ704819.1	JQ704819.1; H1+152; Asia_W_Europe; 15; T152C; A263G; C315CC	16569	Asia_W_Europe	H1+152	H	H	15
3018	JQ704820.1	JQ704820.1; U5b2c2b; Asia_W_Europe_C; 35; A73G; C150T; A263G	16571	Asia_W_Europe_C	U5b2c2b	U	U5	35
3019	JQ704821.1	JQ704821.1; T2a1b2a; Asia_W; 43; A73G; A263G; C315CC	16571	Asia_W	T2a1b2a	T	T	43
3020	JQ704822.1	JQ704822.1; K2a; Asia_W; 33; C64T; A73G; T146C	16569	Asia_W	K2a	K	K	33
3021	JQ704823.1	JQ704823.1; U5a1a1; Asia_W_Europe_C; 32; A73G; T195C; A263G	16570	Asia_W_Europe_C	U5a1a1	U	U5	32
3022	JQ704824.1	JQ704824.1; H1; Asia_W_Europe; 10; A263G; C315CC; A750G	16569	Asia_W_Europe	H1	H	H	10
3023	JQ704825.1	JQ704825.1; L1b1a7a; Africa_C; 81; A73G; T152C; C182T	16567	Africa_C	L1b1a7a	L1	L1	81
3024	JQ704826.1	JQ704826.1; V9a2; Europe_S; 20; T72C; G207A; A263G	16570	Europe_S	V9a2	V	V	20
3025	JQ704827.1	JQ704827.1; H27a; Asia_W_Europe; 14; A263G; C315CC; A750G	16569	Asia_W_Europe	H27a	H	H	14
3026	JQ704828.1	JQ704828.1; W1c1; Asia_W; 41; A73G; A189G; T195C	16569	Asia_W	W1c1	W	W	41
3027	JQ704829.1	JQ704829.1; J1c2l; Asia_W; 34; A73G; G185A; A188G	16569	Asia_W	J1c2l	J	J	34
3028	JQ704830.1	JQ704830.1; K1a3a3; Asia_W; 37; A73G; A263G; C309CCT	16570	Asia_W	K1a3a3	K	K	37
3029	JQ704831.1	JQ704831.1; H81a; Asia_W_Europe; 14; A263G; C315CC; A750G	16569	Asia_W_Europe	H81a	H	H	14
3030	JQ704832.1	JQ704832.1; H1c3; Asia_W_Europe; 14; A257G; A263G; C315CC	16569	Asia_W_Europe	H1c3	H	H	14
3031	JQ704833.1	JQ704833.1; V14; Europe_S; 15; T72C; A263G; C309CCT	16570	Europe_S	V14	V	V	15
3032	JQ704834.1	JQ704834.1; H3a; Asia_W_Europe; 13; T152C; A263G; C309CCCT	16571	Asia_W_Europe	H3a	H	H	13
3033	JQ704835.1	JQ704835.1; H72; Asia_W_Europe; 12; A263G; C309CCT; T310C	16570	Asia_W_Europe	H72	H	H	12
3034	JQ704836.1	JQ704836.1; K1c1; Asia_W; 37; A73G; T146C; T152C	16569	Asia_W	K1c1	K	K	37

Supplementary Table S4 Human mitochondrial database (hMITO DB v1.0) metadata[a]

Row	Name (accession no.)	Description	Size	Geo_Region	Haplogroup	Macro_ Haplo	Macro_ Haplo2	Total Variants
3035	JQ704837.1	JQ704837.1; I3a1; Asia_W_SW; 39; A73G; T152C; T199C	16578	Asia_W_SW	I3a1	I	I	39
3036	JQ704838.1	JQ704838.1; H1a3a2; Asia_W_Europe; 14; T42TG; A73G; A263G	16570	Asia_W_Europe	H1a3a2	H	H	14
3037	JQ704839.1	JQ704839.1; H1+16239; Asia_W_Europe; 11; A263G; C315CC; A750G	16569	Asia_W_Europe	H1+16239	H	H	11
3038	JQ704840.1	JQ704840.1; J1c3e2; Asia_W; 35; A73G; T152C; G185A	16570	Asia_W	J1c3e2	J	J	35
3039	JQ704841.1	JQ704841.1; H13a1a1d1; Asia_W_Europe; 19; A263G; C309CCCT; T310C	16571	Asia_W_Europe	H13a1a1d1	H	H	19
3040	JQ704842.1	JQ704842.1; H2a2a1d; Asia_W_Europe; 3; C309CCCT; T310C; T16172C	16571	Asia_W_Europe	H2a2a1d	H	H	3
3041	JQ704843.1	JQ704843.1; K2a5a1; Asia_W; 37; A73G; T146C; T152C	16570	Asia_W	K2a5a1	K	K	37
3042	JQ704844.1	JQ704844.1; H1c1; Asia_W_Europe; 13; A263G; C315CC; T477C	16569	Asia_W_Europe	H1c1	H	H	13
3043	JQ704845.1	JQ704845.1; H3; Asia_W_Europe; 10; A263G; C315CC; A750G	16569	Asia_W_Europe	H3	H	H	10
3044	JQ704846.1	JQ704846.1; H11a; Asia_W_Europe; 15; T195C; A263G; C309CCCT	16571	Asia_W_Europe	H11a	H	H	15
3045	JQ704847.1	JQ704847.1; H2a1e1b; Asia_W_Europe; 13; A263G; C309CCCT; T310C	16572	Asia_W_Europe	H2a1e1b	H	H	13
3046	JQ704848.1	JQ704848.1; H6b1; Asia_W_Europe; 20; C44CC; T152C; A214G	16572	Asia_W_Europe	H6b1	H	H	20
3047	JQ704849.1	JQ704849.1; H1; Asia_W_Europe; 16; C150T; A263G; C309CCT	16568	Asia_W_Europe	H1	H	H	16
3048	JQ704850.1	JQ704850.1; H7b4; Asia_W_Europe; 14; A263G; C309CCT; T310C	16572	Asia_W_Europe	H7b4	H	H	14
3049	JQ704851.1	JQ704851.1; H4a1a2a1; Asia_W_Europe; 22; A263G; C309CCCT; T310C	16569	Asia_W_Europe	H4a1a2a1	H	H	22
3050	JQ704852.1	JQ704852.1; H1; Asia_W_Europe; 13; A263G; C309CCCT; T310C	16571	Asia_W_Europe	H1	H	H	13
3051	JQ704853.1	JQ704853.1; H63a; Asia_W_Europe; 13; C64T; A263G; C315CC	16569	Asia_W_Europe	H63a	H	H	13
3052	JQ704854.1	JQ704854.1; V1a1; Europe_S; 18; T72C; A263G; C309CCT	16570	Europe_S	V1a1	V	V	18
3053	JQ704855.1	JQ704855.1; H26a1a1; Asia_W_Europe; 15; T196C; A263G; C309CCCT	16571	Asia_W_Europe	H26a1a1	H	H	15
3054	JQ704856.1	JQ704856.1; H48; Asia_W_Europe; 10; A235G; A263G; C315CC	16569	Asia_W_Europe	H48	H	H	10
3055	JQ704857.1	JQ704857.1; H52; Asia_W_Europe; 13; T152C; A263G; C309CCT	16570	Asia_W_Europe	H52	H	H	13
3056	JQ704858.1	JQ704858.1; H50; Asia_W_Europe; 12; A263G; C309CCT; T310C	16570	Asia_W_Europe	H50	H	H	12
3057	JQ704859.1	JQ704859.1; X2b12; Asia_W_America_N; 32; A73G; A153G; T195C	16570	Asia_W_America_N	X2b12	X	X	32
3058	JQ704860.1	JQ704860.1; H10+(16093); Asia_W_Europe; 12; A188G; A197G; C315CC	16569	Asia_W_Europe	H10+(16093)	H	H	12
3059	JQ704861.1	JQ704861.1; H1c1a; Asia_W_Europe; 13; A263G; C315CC; T477C	16569	Asia_W_Europe	H1c1a	H	H	13
3060	JQ704862.1	JQ704862.1; U5b2a1b; Asia_W_Europe_C; 32; A73G; C150T; T152C	16569	Asia_W_Europe_C	U5b2a1b	U	U5	32
3061	JQ704863.1	JQ704863.1; H5b2; Asia_W_Europe; 14; A263G; C309CCCT; T310C	16571	Asia_W_Europe	H5b2	H	H	14
3062	JQ704864.1	JQ704864.1; H7; Asia_W_Europe; 13; T152C; A263G; C309CCT	16574	Asia_W_Europe	H7	H	H	13
3063	JQ704865.1	JQ704865.1; H69; Asia_W_Europe; 11; T152C; A263G; C315CC	16569	Asia_W_Europe	H69	H	H	11
3064	JQ704866.1	JQ704866.1; X2k; Asia_W_America_N; 27; A73G; A153G; T195C	16567	Asia_W_America_N	X2k	X	X	27
3065	JQ704867.1	JQ704867.1; J1c1c; Asia_W; 34; A73G; C150T; G185A	16568	Asia_W	J1c1c	J	J	34
3066	JQ704868.1	JQ704868.1; H34; Asia_W_Europe; 14; T152C; A263G; C315CC	16569	Asia_W_Europe	H34	H	H	14
3067	JQ704869.1	JQ704869.1; T2b4+152; Asia_W; 35; A73G; T152C; A263G	16570	Asia_W	T2b4+152	T	T	35
3068	JQ704870.1	JQ704870.1; H15b; Asia_W_Europe; 13; T55C; T57C; A263G	16571	Asia_W_Europe	H15b	H	H	13
3069	JQ704871.1	JQ704871.1; N1a1a1a3; Africa_NE_Asia_W; 40; A73G; T152C; T199C	16572	Africa_NE_Asia_W	N1a1a1a3	N	N1	40
3070	JQ704872.1	JQ704872.1; H1+16189; Asia_W_Europe; 10; A263G; C315CC; A750G	16569	Asia_W_Europe	H1+16189	H	H	10
3071	JQ704873.1	JQ704873.1; K1a4a1a+195; Asia_W; 39; A73G; T195C; A263G	16571	Asia_W	K1a4a1a+195	K	K	39
3072	JQ704874.1	JQ704874.1; H2a2a1; Asia_W_Europe; 6; T152C; C309CCCT; T310C	16571	Asia_W_Europe	H2a2a1	H	H	6
3073	JQ704875.1	JQ704875.1; U5b1a; Asia_W_Europe_C; 28; A73G; C150T; A263G	16572	Asia_W_Europe_C	U5b1a	U	U5	28
3074	JQ704876.1	JQ704876.1; H40; Asia_W_Europe; 12; A263G; C309CCT; T310C	16572	Asia_W_Europe	H40	H	H	12
3075	JQ704877.1	JQ704877.1; H1a; Asia_W_Europe; 13; A73G; A263G; C309CCT	16570	Asia_W_Europe	H1a	H	H	13

Supplementary Table S4 Human mitochondrial database (hMITO DB v1.0) metadata[a]

Row	Name (accession no.)	Description	Size	Geo_Region	Haplogroup	Macro_ Haplo	Macro_ Haplo2	Total Variants
3076	JQ704878.1	JQ704878.1; A2+(64); Asia_NE_America_N; 37; C64T; A73G; T131C	16567	Asia_NE_America_N	A2+(64)	A	A	37
3077	JQ704879.1	JQ704879.1; K2a; Asia_W; 31; A73G; T146C; T152C	16569	Asia_W	K2a	K	K	31
3078	JQ704880.1	JQ704880.1; H1a1; Asia_W_Europe; 15; A73G; A263G; C315CC	16569	Asia_W_Europe	H1a1	H	H	15
3079	JQ704881.1	JQ704881.1; H1b; Asia_W_Europe; 14; A263G; C309CCCT; T310C	16571	Asia_W_Europe	H1b	H	H	14
3080	JQ704882.1	JQ704882.1; H1b2a; Asia_W_Europe; 17; A183G; A263G; C309CCCT	16571	Asia_W_Europe	H1b2a	H	H	17
3081	JQ704883.1	JQ704883.1; H4a1a+195; Asia_W_Europe; 20; T195C; A263G; C309CCCT	16569	Asia_W_Europe	H4a1a+195	H	H	20
3082	JQ704884.1	JQ704884.1; C1b11; Asia_NE_America_N_S; 50; T72C; A73G; C194T	16565	Asia_NE_America_N_S	C1b11	C	C	50
3083	JQ704885.1	JQ704885.1; H5b; Asia_W_Europe; 12; A263G; C309CCT; T310C	16570	Asia_W_Europe	H5b	H	H	12
3084	JQ704886.1	JQ704886.1; H5a1g1a; Asia_W_Europe; 18; A263G; C315CC; A444G	16567	Asia_W_Europe	H5a1g1a	H	H	18
3085	JQ704887.1	JQ704887.1; H3r; Asia_W_Europe; 12; A263G; C315CC; A750G	16569	Asia_W_Europe	H3r	H	H	12
3086	JQ704888.1	JQ704888.1; H27a; Asia_W_Europe; 14; A263G; C309CCT; T310C	16570	Asia_W_Europe	H27a	H	H	14
3087	JQ704889.1	JQ704889.1; H6a1b4; Asia_W_Europe; 17; T152C; T239C; A263G	16570	Asia_W_Europe	H6a1b4	H	H	17
3088	JQ704890.1	JQ704890.1; H7b; Asia_W_Europe; 17; T195C; A263G; C309CCCT	16571	Asia_W_Europe	H7b	H	H	17
3089	JQ704891.1	JQ704891.1; J1c8a; Asia_W; 35; A73G; G185A; G228A	16574	Asia_W	J1c8a	J	J	35
3090	JQ704892.1	JQ704892.1; B4c1a1a; Asia_SE_E_America_N_S; 30; A73G; T146C; A263G	16564	Asia_SE_E_America_N_S	B4c1a1a	B	B	30
3091	JQ704893.1	JQ704893.1; N1b1b1; Africa_NE_Asia_W; 40; A73G; T152C; A263G	16570	Africa_NE_Asia_W	N1b1b1	N	N1	40
3092	JQ704894.1	JQ704894.1; H1b2a1; Asia_W_Europe; 18; A183G; A263G; C309CCCT	16571	Asia_W_Europe	H1b2a1	H	H	18
3093	JQ704895.1	JQ704895.1; U5a2b4a; Asia_W_Europe_C; 26; A73G; A263G; C309CCT	16570	Asia_W_Europe_C	U5a2b4a	U	U5	26
3094	JQ704896.1	JQ704896.1; U6b1a; Asia_SW_Africa_N; 26; A73G; A263G; C315CC	16569	Asia_SW_Africa_N	U6b1a	U	U6	26
3095	JQ704897.1	JQ704897.1; T2b; Asia_W; 38; A73G; T204C; G207A	16570	Asia_W	T2b	T	T	38
3096	JQ704898.1	JQ704898.1; H; Asia_W_Europe; 10; A263G; C315CC; A750G	16569	Asia_W_Europe	H	H	H	10
3097	JQ704899.1	JQ704899.1; H26c; Asia_W_Europe; 12; T146C; A263G; C315CC	16569	Asia_W_Europe	H26c	H	H	12
3098	JQ704900.1	JQ704900.1; K2b1b; Asia_W; 38; A73G; T146C; T195C	16569	Asia_W	K2b1b	K	K	38
3099	JQ704901.1	JQ704901.1; V+@72; Europe_S; 18; A263G; C315CC; CA522d	16567	Europe_S	V+@72	V	V	18
3100	JQ704902.1	JQ704902.1; C1c1b; Asia_NE_America_N_S; 44; A73G; A215G; A249d	16567	Asia_NE_America_N_S	C1c1b	C	C	44
3101	JQ704903.1	JQ704903.1; H3v; Asia_W_Europe; 11; A263G; C309CCT; T310C	16570	Asia_W_Europe	H3v	H	H	11
3102	JQ704904.1	JQ704904.1; K1c2; Asia_W; 38; A73G; T146C; T152C	16568	Asia_W	K1c2	K	K	38
3103	JQ704905.1	JQ704905.1; H1e2; Asia_W_Europe; 13; A263G; C315CC; A750G	16569	Asia_W_Europe	H1e2	H	H	13
3104	JQ704906.1	JQ704906.1; J2a1a1a2; Asia_W; 41; A73G; C150T; T152C	16570	Asia_W	J2a1a1a2	J	J	41
3105	JQ704907.1	JQ704907.1; K1a+195; Asia_W; 31; A73G; T195C; A263G	16573	Asia_W	K1a+195	K	K	31
3106	JQ704908.1	JQ704908.1; J1c1b2a; Asia_W; 35; A73G; G228A; A263G	16567	Asia_W	J1c1b2a	J	J	35
3107	JQ704909.1	JQ704909.1; H1c15; Asia_W_Europe; 14; T152C; A263G; C309CCT	16573	Asia_W_Europe	H1c15	H	H	14
3108	JQ704910.1	JQ704910.1; H6a1b2; Asia_W_Europe; 18; T239C; A263G; C309CCT	16572	Asia_W_Europe	H6a1b2	H	H	18
3109	JQ704911.1	JQ704911.1; T2b4a; Asia_W; 36; A73G; A263G; C309CCT	16570	Asia_W	T2b4a	T	T	36
3110	JQ704912.1	JQ704912.1; K2a7; Asia_W; 35; A73G; T146C; T152C	16568	Asia_W	K2a7	K	K	35
3111	JQ704913.1	JQ704913.1; H1b1e; Asia_W_Europe; 14; A263G; C315CC; A750G	16569	Asia_W_Europe	H1b1e	H	H	14
3112	JQ704914.1	JQ704914.1; H1cf; Asia_W_Europe; 14; A263G; C315CC; A750G	16569	Asia_W_Europe	H1cf	H	H	14
3113	JQ704915.1	JQ704915.1; U5a1a1; Asia_W_Europe_C; 29; A73G; A263G; C309CCCT	16571	Asia_W_Europe_C	U5a1a1	U	U5	29
3114	JQ704916.1	JQ704916.1; H1f1a; Asia_W_Europe; 17; A263G; C315CC; C459d(=C456d†)	16568	Asia_W_Europe	H1f1a	H	H	17
3115	JQ704917.1	JQ704917.1; L3e2b1a1; Africa_E; 37; A73G; C150T; T195C	16571	Africa_E	L3e2b1a1	L3	L3	37
3116	JQ704918.1	JQ704918.1; U5a1h; Asia_W_Europe_C; 34; A73G; C150T; A263G	16569	Asia_W_Europe_C	U5a1h	U	U5	34

Supplementary Table S4 Human mitochondrial database (hMITO DB v1.0) metadata[a]

Row	Name (accession no.)	Description	Size	Geo_Region	Haplogroup	Macro_ Haplo	Macro_ Haplo2	Total Variants
3117	JQ704919.1	JQ704919.1; L3b3; Africa_E; 41; A73G; C150T; G185A	16566	Africa_E	L3b3	L3	L3	41
3118	JQ704920.1	JQ704920.1; U5a1a1+16362; Asia_W_Europe_C; 33; A73G; A263G; C309CCT	16573	Asia_W_Europe_C	U5a1a1+16362	U	U5	33
3119	JQ704921.1	JQ704921.1; U5b2a1a1; Asia_W_Europe_C; 29; A73G; C150T; A263G	16569	Asia_W_Europe_C	U5b2a1a1	U	U5	29
3120	JQ704922.1	JQ704922.1; B4a1a1c; Asia_SE_E_America_N_S; 30; A73G; T146C; A263G	16560	Asia_SE_E_America_N_S	B4a1a1c	B	B	30
3121	JQ704923.1	JQ704923.1; HV0+195; Asia_W; 18; C64T; T72C; T195C	16570	Asia_W	HV0+195	HV	HV	18
3122	JQ704924.1	JQ704924.1; K1a24a; Asia_W; 37; A73G; C150T; T195C	16567	Asia_W	K1a24a	K	K	37
3123	JQ704925.1	JQ704925.1; T2b4b; Asia_W; 37; A73G; T152C; A263G	16570	Asia_W	T2b4b	T	T	37
3124	JQ704926.1	JQ704926.1; X2b+226; Asia_W_America_N; 34; A73G; A153G; A188G	16572	Asia_W_America_N	X2b+226	X	X	34
3125	JQ704927.1	JQ704927.1; H1a3b; Asia_W_Europe; 14; A73G; A263G; C315CC	16567	Asia_W_Europe	H1a3b	H	H	14
3126	JQ704928.1	JQ704928.1; H13a1a; Asia_W_Europe; 14; A263G; C309CCT; T310C	16570	Asia_W_Europe	H13a1a	H	H	14
3127	JQ704929.1	JQ704929.1; V; Europe_S; 16; T72C; A263G; C309CCT	16570	Europe_S	V	V	V	16
3128	JQ704930.1	JQ704930.1; U5a1a1; Asia_W_Europe_C; 29; A73G; A263G; C315CC	16569	Asia_W_Europe_C	U5a1a1	U	U5	29
3129	JQ704931.1	JQ704931.1; L3e3b1; Africa_E; 41; A73G; C150T; T195C	16567	Africa_E	L3e3b1	L3	L3	41
3130	JQ704932.1	JQ704932.1; H3b1b; Asia_W_Europe; 16; A153G; A263G; C309CCT	16570	Asia_W_Europe	H3b1b	H	H	16
3131	JQ704933.1	JQ704933.1; K2b1a3; Asia_W; 35; A73G; T146C; A263G	16569	Asia_W	K2b1a3	K	K	35
3132	JQ704934.1	JQ704934.1; H1c3b; Asia_W_Europe; 16; A257G; A263G; C309CCCT	16571	Asia_W_Europe	H1c3b	H	H	16
3133	JQ704935.1	JQ704935.1; J1c2; Asia_W; 32; A73G; G185A; A188G	16568	Asia_W	J1c2	J	J	32
3134	JQ704936.1	JQ704936.1; I2; Asia_W_SW; 40; A73G; T152C; T199C	16573	Asia_W_SW	I2	I	I	40
3135	JQ704937.1	JQ704937.1; J1c; Asia_W; 30; A73G; C150T; G185A	16569	Asia_W	J1c	J	J	30
3136	JQ704938.1	JQ704938.1; H15a1; Asia_W_Europe; 17; T55C; T57C; A263G	16570	Asia_W_Europe	H15a1	H	H	17
3137	JQ704939.1	JQ704939.1; H2a1c; Asia_W_Europe; 12; A263G; C309CCCT; T310C	16571	Asia_W_Europe	H2a1c	H	H	12
3138	JQ704940.1	JQ704940.1; H61; Asia_W_Europe; 12; A73G; A263G; C315CC	16569	Asia_W_Europe	H61	H	H	12
3139	JQ704941.1	JQ704941.1; U5a1b1a; Asia_W_Europe_C; 30; A73G; A263G; A272G	16569	Asia_W_Europe_C	U5a1b1a	U	U5	30
3140	JQ704942.1	JQ704942.1; J1b1a1; Asia_W; 36; A73G; C242T; A263G	16569	Asia_W	J1b1a1	J	J	36
3141	JQ704943.1	JQ704943.1; H1a; Asia_W_Europe; 15; A73G; A263G; C309CCT	16570	Asia_W_Europe	H1a	H	H	15
3142	JQ704944.1	JQ704944.1; K1a9; Asia_W; 32; A73G; T195C; A263G	16569	Asia_W	K1a9	K	K	32
3143	JQ704945.1	JQ704945.1; V21; Europe_S; 16; T152C; A263G; C309CCCT	16571	Europe_S	V21	V	V	16
3144	JQ704946.1	JQ704946.1; J1c2g; Asia_W; 32; A73G; G185A; A188G	16569	Asia_W	J1c2g	J	J	32
3145	JQ704947.1	JQ704947.1; J2a1a1; Asia_W; 41; A73G; C150T; T152C	16570	Asia_W	J2a1a1	J	J	41
3146	JQ704948.1	JQ704948.1; K1a4a1b2; Asia_W; 40; A73G; A263G; C309CCCT	16571	Asia_W	K1a4a1b2	K	K	40
3147	JQ704949.1	JQ704949.1; H56; Asia_W_Europe; 13; A263G; C309CCT; T310C	16570	Asia_W_Europe	H56	H	H	13
3148	JQ704950.1	JQ704950.1; U3a1a1; Africa_NE_Asia_W; 37; A73G; C150T; A263G	16571	Africa_NE_Asia_W	U3a1a1	U	U3	37
3149	JQ704951.1	JQ704951.1; K1c2; Asia_W; 37; A73G; T146C; T152C	16568	Asia_W	K1c2	K	K	37
3150	JQ704952.1	JQ704952.1; H; Asia_W_Europe; 17; A200G; G225A; A227G	16571	Asia_W_Europe	H	H	H	17
3151	JQ704953.1	JQ704953.1; H17; Asia_W_Europe; 14; T152C; A263G; C309CCCT	16571	Asia_W_Europe	H17	H	H	14
3152	JQ704954.1	JQ704954.1; H1; Asia_W_Europe; 12; A263G; C309CCT; T310C	16569	Asia_W_Europe	H1	H	H	12
3153	JQ704955.1	JQ704955.1; H10c; Asia_W_Europe; 12; A263G; C309CCCT; T310C	16571	Asia_W_Europe	H10c	H	H	12
3154	JQ704956.1	JQ704956.1; D4b2b7; Asia_E_America_N_S; 42; A73G; C194T; A263G	16567	Asia_E_America_N_S	D4b2b7	D	D	42
3155	JQ704957.1	JQ704957.1; H1af1b; Asia_W_Europe; 13; A73G; A263G; C315CC	16569	Asia_W_Europe	H1af1b	H	H	13
3156	JQ704958.1	JQ704958.1; H3b1a; Asia_W_Europe; 16; A263G; C315CC; A750G	16569	Asia_W_Europe	H3b1a	H	H	16
3157	JQ704959.1	JQ704959.1; X2b+226; Asia_W_America_N; 29; A73G; A153G; T195C	16569	Asia_W_America_N	X2b+226	X	X	29

Supplementary Table S4 Human mitochondrial database (hMITO DB v1.0) metadata[a]

Row	Name (accession no.)	Description	Size	Geo_Region	Haplogroup	Macro_ Haplo	Macro_ Haplo2	Total Variants
3158	JQ704960.1	JQ704960.1; V; Europe_S; 14; T72C; A263G; C309CCT	16570	Europe_S	V	V	V	14
3159	JQ704961.1	JQ704961.1; U5a1a1+16362; Asia_W_Europe_C; 29; A73G; T204C; A263G	16570	Asia_W_Europe_C	U5a1a1+16362	U	U5	29
3160	JQ704962.1	JQ704962.1; U5a1f1a; Asia_W_Europe_C; 33; A73G; T199C; A263G	16569	Asia_W_Europe_C	U5a1f1a	U	U5	33
3161	JQ704963.1	JQ704963.1; K1a4d; Asia_W; 36; A73G; A263G; C315CC	16571	Asia_W	K1a4d	K	K	36
3162	JQ704964.1	JQ704964.1; U8a1a1; Asia_W_Europe ; 29; A73G; A263G; T282C	16569	Asia_W_Europe	U8a1a1	U	U8	29
3163	JQ704965.1	JQ704965.1; J1c2d; Asia_W; 33; A73G; G185A; A188G	16568	Asia_W	J1c2d	J	J	33
3164	JQ704966.1	JQ704966.1; HV+16311; Asia_W_Europe; 17; A263G; C309CCCT; T310C	16571	Asia_W_Europe	HV+16311	H	H	17
3165	JQ704967.1	JQ704967.1; T1a1; Asia_W; 36; A73G; T152C; T195C	16570	Asia_W	T1a1	T	T	36
3166	JQ704968.1	JQ704968.1; L1b1a8; Africa_C; 78; A73G; T152C; C182T	16567	Africa_C	L1b1a8	L1	L1	78
3167	JQ704969.1	JQ704969.1; X2b+226; Asia_W_America_N; 32; A73G; A153G; T195C	16569	Asia_W_America_N	X2b+226	X	X	32
3168	JQ704970.1	JQ704970.1; U8b1b1; Asia_W_Europe ; 29; A73G; T195C; A263G	16570	Asia_W_Europe	U8b1b1	U	U8	29
3169	JQ704971.1	JQ704971.1; U5b3b; Asia_W_Europe_C; 28; A73G; C150T; G228A	16570	Asia_W_Europe_C	U5b3b	U	U5	28
3170	JQ704972.1	JQ704972.1; H17b; Asia_W_Europe; 13; T195C; A263G; C315CC	16570	Asia_W_Europe	H17b	H	H	13
3171	JQ704973.1	JQ704973.1; T2b28; Asia_W; 36; A73G; A153G; A263G	16569	Asia_W	T2b28	T	T	36
3172	JQ704974.1	JQ704974.1; D4; Asia_E_America_N_S; 31; A73G; A263G; C309CCT	16570	Asia_E_America_N_S	D4	D	D	31
3173	JQ704975.1	JQ704975.1; W1g; Asia_W; 34; A73G; A189G; T195C	16569	Asia_W	W1g	W	W	34
3174	JQ704976.1	JQ704976.1; I4b; Asia_W_SW; 40; A73G; T199C; T204C	16573	Asia_W_SW	I4b	I	I	40
3175	JQ704977.1	JQ704977.1; X1c; Asia_W_America_N; 32; A73G; T146C; A153G	16567	Asia_W_America_N	X1c	X	X	32
3176	JQ704978.1	JQ704978.1; L2c; Africa_W_C; 59; A73G; T146C; C150T	16567	Africa_W_C	L2c	L2	L2	59
3177	JQ704979.1	JQ704979.1; H3h3a; Asia_W_Europe; 14; A263G; C315CC; A750G	16569	Asia_W_Europe	H3h3a	H	H	14
3178	JQ704980.1	JQ704980.1; H1+16189; Asia_W_Europe; 14; A263G; C315CC; A750G	16569	Asia_W_Europe	H1+16189	H	H	14
3179	JQ704981.1	JQ704981.1; U3b2b; Africa_NE_Asia_W; 33; A73G; C150T; A263G	16566	Africa_NE_Asia_W	U3b2b	U	U3	33
3180	JQ704982.1	JQ704982.1; L3e2a1a; Africa_E; 37; A73G; C150T; T195C	16569	Africa_E	L3e2a1a	L3	L3	37
3181	JQ704983.1	JQ704983.1; K1a1b1b1; Asia_W; 38; A73G; A263G; C309CCT	16570	Asia_W	K1a1b1b1	K	K	38
3182	JQ704984.1	JQ704984.1; U5b1+16189+@16192; Asia_W_Europe_C; 27; GGAGCA106-; T146	16563	Asia_W_Europe_C	U5b1+16189+@	U	U5	27
3183	JQ704985.1	JQ704985.1; X2l; Asia_W_America_N; 30; A73G; T195C; G225A	16571	Asia_W_America_N	X2l	X	X	30
3184	JQ704986.1	JQ704986.1; H3ah; Asia_W_Europe; 15; T152C; A263G; C309CCT	16568	Asia_W_Europe	H3ah	H	H	15
3185	JQ704987.1	JQ704987.1; H54; Asia_W_Europe; 12; C182T; A263G; C309CCT	16570	Asia_W_Europe	H54	H	H	12
3186	JQ704988.1	JQ704988.1; V23; Europe_S; 16; T72C; A263G; C309CCT	16570	Europe_S	V23	V	V	16
3187	JQ704989.1	JQ704989.1; W1g; Asia_W; 33; A73G; A189G; T195C	16569	Asia_W	W1g	W	W	33
3188	JQ704990.1	JQ704990.1; U5a1a2a; Asia_W_Europe_C; 31; A73G; A263G; C309CCT	16572	Asia_W_Europe_C	U5a1a2a	U	U5	31
3189	JQ704991.1	JQ704991.1; U5a1a2b1; Asia_W_Europe_C; 34; A73G; A263G; C309CCT	16570	Asia_W_Europe_C	U5a1a2b1	U	U5	34
3190	JQ704992.1	JQ704992.1; T2b; Asia_W; 35; A73G; A263G; C309CCT	16570	Asia_W	T2b	T	T	35
3191	JQ704993.1	JQ704993.1; U4c1; Asia_N_Europe_N; 37; A73G; T195C; A263G	16569	Asia_N_Europe_N	U4c1	U	U4	37
3192	JQ704994.1	JQ704994.1; H1ac; Asia_W_Europe; 13; A263G; C309CCCT; T310C	16571	Asia_W_Europe	H1ac	H	H	13
3193	JQ704995.1	JQ704995.1; T2n; Asia_W; 33; A73G; T152C; A263G	16570	Asia_W	T2n	T	T	33
3194	JQ704996.1	JQ704996.1; W3a1; Asia_W; 37; A73G; A189G; C194T	16570	Asia_W	W3a1	W	W	37
3195	JQ704997.1	JQ704997.1; T2b7a2; Asia_W; 39; A73G; A263G; C309CCT	16570	Asia_W	T2b7a2	T	T	39
3196	JQ704998.1	JQ704998.1; V; Europe_S; 15; T72C; A263G; C309CCT	16570	Europe_S	V	V	V	15
3197	JQ704999.1	JQ704999.1; U2e1a1b; Asia_S_W_Europe; 42; A73G; A108G; T152C	16573	Asia_S_W_Europe	U2e1a1b	U	U2	42
3198	JQ705000.1	JQ705000.1; I2; Asia_W_SW; 36; A73G; T152C; T199C	16571	Asia_W_SW	I2	I	I	36

Supplementary Table S4 Human mitochondrial database (hMITO DB v1.0) metadata[a]

Row	Name (accession no.)	Description	Size	Geo_Region	Haplogroup	Macro_ Haplo	Macro_ Haplo2	Total Variants
3199	JQ705001.1	JQ705001.1; L2a1c5; Africa_W_C; 55; A73G; G143A; T146C	16569	Africa_W_C	L2a1c5	L2	L2	55
3200	JQ705002.1	JQ705002.1; N1a1a1a2; Africa_NE_Asia_W; 44; A73G; T152C; T199C	16571	Africa_NE_Asia_W	N1a1a1a2	N	N1	44
3201	JQ705003.1	JQ705003.1; H3b1b1a; Asia_W_Europe; 18; A153G; A263G; C309CCCT	16571	Asia_W_Europe	H3b1b1a	H	H	18
3202	JQ705004.1	JQ705004.1; HV1b2; Asia_W; 21; T152C; A263G; C309CCT	16570	Asia_W	HV1b2	HV	HV	21
3203	JQ705005.1	JQ705005.1; H46a; Asia_W_Europe; 13; T152C; A263G; C315CC	16567	Asia_W_Europe	H46a	H	H	13
3204	JQ705006.1	JQ705006.1; J1c2a3; Asia_W; 35; A73G; G185A; A188G	16570	Asia_W	J1c2a3	J	J	35
3205	JQ705007.1	JQ705007.1; U5b2a1b; Asia_W_Europe_C; 30; A73G; C150T; T152C	16569	Asia_W_Europe_C	U5b2a1b	U	U5	30
3206	JQ705008.1	JQ705008.1; HV0+195; Asia_W; 16; T72C; T195C; A263G	16571	Asia_W	HV0+195	HV	HV	16
3207	JQ705009.1	JQ705009.1; T2b11; Asia_W; 38; A73G; G207A; A263G	16571	Asia_W	T2b11	T	T	38
3208	JQ705010.1	JQ705010.1; K1c1; Asia_W; 35; A73G; T146C; T152C	16568	Asia_W	K1c1	K	K	35
3209	JQ705011.1	JQ705011.1; H1b1; Asia_W_Europe; 13; A263G; C315CC; CA522d	16567	Asia_W_Europe	H1b1	H	H	13
3210	JQ705012.1	JQ705012.1; L3e2b; Africa_E; 35; A73G; C150T; T195C	16570	Africa_E	L3e2b	L3	L3	35
3211	JQ705013.1	JQ705013.1; H1; Asia_W_Europe; 11; A263G; C315CC; A750G	16569	Asia_W_Europe	H1	H	H	11
3212	JQ705014.1	JQ705014.1; U3a1; Africa_NE_Asia_W; 34; A73G; C150T; A263G	16570	Africa_NE_Asia_W	U3a1	U	U3	34
3213	JQ705015.1	JQ705015.1; T2f1a1; Asia_W; 40; A73G; T195C; A263G	16560	Asia_W	T2f1a1	T	T	40
3214	JQ705016.1	JQ705016.1; K1a1b1a; Asia_W; 37; A73G; C114T; A263G	16569	Asia_W	K1a1b1a	K	K	37
3215	JQ705017.1	JQ705017.1; K1a9; Asia_W; 32; A73G; T195C; A263G	16569	Asia_W	K1a9	K	K	32
3216	JQ705018.1	JQ705018.1; T2h1; Asia_W; 36; A73G; A263G; C309CCT	16570	Asia_W	T2h1	T	T	36
3217	JQ705019.1	JQ705019.1; L3d2b; Africa_E; 41; A73G; T152C; T199C	16568	Africa_E	L3d2b	L3	L3	41
3218	JQ705020.1	JQ705020.1; H3y; Asia_W_Europe; 14; A263G; C309CCCT; T310C	16571	Asia_W_Europe	H3y	H	H	14
3219	JQ705021.1	JQ705021.1; J2b1a+16311; Asia_W; 33; A73G; C150T; T152C	16569	Asia_W	J2b1a+16311	J	J	33
3220	JQ705022.1	JQ705022.1; H9a; Asia_W_Europe; 15; T152C; A263G; C309CCT	16570	Asia_W_Europe	H9a	H	H	15
3221	JQ705023.1	JQ705023.1; T2f1a1; Asia_W; 44; A73G; T195C; A263G	16558	Asia_W	T2f1a1	T	T	44
3222	JQ705024.1	JQ705024.1; H1e1; Asia_W_Europe; 17; T146C; A263G; C309CCT	16568	Asia_W_Europe	H1e1	H	H	17
3223	JQ705025.1	JQ705025.1; I1a1; Asia_W_SW; 44; A73G; T199C; A202G	16573	Asia_W_SW	I1a1	I	I	44
3224	JQ705026.1	JQ705026.1; K1a9; Asia_W; 32; A73G; T195C; A263G	16569	Asia_W	K1a9	K	K	32
3225	JQ705027.1	JQ705027.1; H1c+152; Asia_W_Europe; 13; T152C; A263G; C309CCT	16568	Asia_W_Europe	H1c+152	H	H	13
3226	JQ705028.1	JQ705028.1; H1+16311; Asia_W_Europe; 10; A263G; C315CC; A750G	16569	Asia_W_Europe	H1+16311	H	H	10
3227	JQ705029.1	JQ705029.1; K2a8; Asia_W; 35; A73G; T146C; T152C	16569	Asia_W	K2a8	K	K	35
3228	JQ705030.1	JQ705030.1; X2c1; Asia_W_America_N; 33; A73G; A153G; T195C	16569	Asia_W_America_N	X2c1	X	X	33
3229	JQ705031.1	JQ705031.1; J1c5; Asia_W; 29; A73G; G185A; G228A	16569	Asia_W	J1c5	J	J	29
3230	JQ705032.1	JQ705032.1; H10b; Asia_W_Europe; 12; A263G; C309CCT; T310C	16570	Asia_W_Europe	H10b	H	H	12
3231	JQ705033.1	JQ705033.1; H27; Asia_W_Europe; 12; A263G; C315CC; A750G	16569	Asia_W_Europe	H27	H	H	12
3232	JQ705034.1	JQ705034.1; X2b5; Asia_W_America_N; 33; A73G; A153G; T195C	16569	Asia_W_America_N	X2b5	X	X	33
3233	JQ705035.1	JQ705035.1; H5a1d; Asia_W_Europe; 13; A263G; C315CC; C456T	16567	Asia_W_Europe	H5a1d	H	H	13
3234	JQ705036.1	JQ705036.1; W1+119; Asia_W; 37; A73G; T119C; A189G	16570	Asia_W	W1+119	W	W	37
3235	JQ705037.1	JQ705037.1; T2b3d; Asia_W; 38; A73G; C151T; T199C	16570	Asia_W	T2b3d	T	T	38
3236	JQ705038.1	JQ705038.1; T2b24; Asia_W; 36; A73G; T146C; A263G	16570	Asia_W	T2b24	T	T	36
3237	JQ705039.1	JQ705039.1; H1i2a; Asia_W_Europe; 13; T152C; A263G; C315CC	16569	Asia_W_Europe	H1i2a	H	H	13
3238	JQ705040.1	JQ705040.1; H87; Asia_W_Europe; 11; C150T; A263G; C309CCCT	16571	Asia_W_Europe	H87	H	H	11
3239	JQ705041.1	JQ705041.1; H3h5; Asia_W_Europe; 12; A263G; C315CC; A750G	16569	Asia_W_Europe	H3h5	H	H	12

Supplementary Table S4 Human mitochondrial database (hMITO DB v1.0) metadata[a]

Row	Name (accession no.)	Description	Size	Geo_Region	Haplogroup	Macro_ Haplo	Macro_ Haplo2	Total Variants
3240	JQ705042.1	JQ705042.1; J2a1a1b; Asia_W; 42; A73G; C150T; T152C	16571	Asia_W	J2a1a1b	J	J	42
3241	JQ705043.1	JQ705043.1; U3b1b; Africa_NE_Asia_W; 29; A73G; C150T; A263G	16570	Africa_NE_Asia_W	U3b1b	U	U3	29
3242	JQ705044.1	JQ705044.1; K1b2a2; Asia_W; 35; A73G; T146C; T195C	16571	Asia_W	K1b2a2	K	K	35
3243	JQ705045.1	JQ705045.1; H7d5; Asia_W_Europe; 16; A263G; C309CCT; T310C	16570	Asia_W_Europe	H7d5	H	H	16
3244	JQ705046.1	JQ705046.1; L2a1c1a1; Africa_W_C; 61; A73G; G143A; T146C	16569	Africa_W_C	L2a1c1a1	L2	L2	61
3245	JQ705047.1	JQ705047.1; T2b6b; Asia_W; 43; A73G; T146C; A263G	16570	Asia_W	T2b6b	T	T	43
3246	JQ705048.1	JQ705048.1; H1a1; Asia_W_Europe; 14; A73G; A263G; C315CC	16569	Asia_W_Europe	H1a1	H	H	14
3247	JQ705049.1	JQ705049.1; L2a1l2a; Africa_W_C; 55; A73G; T146C; T152C	16570	Africa_W_C	L2a1l2a	L2	L2	55
3248	JQ705050.1	JQ705050.1; H1e2a; Asia_W_Europe; 13; A263G; C315CC; A750G	16569	Asia_W_Europe	H1e2a	H	H	13
3249	JQ705051.1	JQ705051.1; J1c1; Asia_W; 35; A73G; G228A; A263G	16569	Asia_W	J1c1	J	J	35
3250	JQ705052.1	JQ705052.1; H1as2; Asia_W_Europe; 11; A263G; C315CC; A750G	16569	Asia_W_Europe	H1as2	H	H	11
3251	JQ705053.1	JQ705053.1; H28a1; Asia_W_Europe; 15; C182T; C186A; A263G	16570	Asia_W_Europe	H28a1	H	H	15
3252	JQ705054.1	JQ705054.1; J1c2t; Asia_W; 32; A73G; T152C; G185A	16570	Asia_W	J1c2t	J	J	32
3253	JQ705055.1	JQ705055.1; L2a1a2a1a; Africa_W_C; 61; A73G; T146C; T152C	16570	Africa_W_C	L2a1a2a1a	L2	L2	61
3254	JQ705056.1	JQ705056.1; W1+119; Asia_W; 35; A73G; T119C; A189G	16569	Asia_W	W1+119	W	W	35
3255	JQ705057.1	JQ705057.1; T2a1a; Asia_W; 34; A73G; A263G; C315CC	16569	Asia_W	T2a1a	T	T	34
3256	JQ705058.1	JQ705058.1; T1a; Asia_W; 37; A73G; T239C; A263G	16570	Asia_W	T1a	T	T	37
3257	JQ705059.1	JQ705059.1; K2a6; Asia_W; 34; A73G; T146C; T152C	16570	Asia_W	K2a6	K	K	34
3258	JQ705060.1	JQ705060.1; I4a1; Asia_W_SW; 37; A73G; T199C; T250C	16571	Asia_W_SW	I4a1	I	I	37
3259	JQ705061.1	JQ705061.1; R1a1a2; Asia_S_SE; 37; A73G; A263G; C295A	16570	Asia_S_SE	R1a1a2	R	R1	37
3260	JQ705062.1	JQ705062.1; H+152; Asia_W_Europe; 14; C151T; T152C; A263G	16570	Asia_W_Europe	H+152	H	H	14
3261	JQ705063.1	JQ705063.1; K1e1; Asia_W; 41; A73G; C151T; T152C	16572	Asia_W	K1e1	K	K	41
3262	JQ705064.1	JQ705064.1; J2b1; Asia_W; 32; A73G; C150T; T152C	16569	Asia_W	J2b1	J	J	32
3263	JQ705065.1	JQ705065.1; U5a1a1; Asia_W_Europe_C; 29; A73G; A263G; C309CCT	16570	Asia_W_Europe_C	U5a1a1	U	U5	29
3264	JQ705066.1	JQ705066.1; H3b+16129; Asia_W_Europe; 12; A263G; C315CC; A750G	16569	Asia_W_Europe	H3b+16129	H	H	12
3265	JQ705067.1	JQ705067.1; X2i1; Asia_W_America_N; 30; A73G; A153G; T195C	16570	Asia_W_America_N	X2i1	X	X	30
3266	JQ705068.1	JQ705068.1; U2e1g; Asia_S_W_Europe; 39; A73G; T152C; T217C	16570	Asia_S_W_Europe	U2e1g	U	U2	39
3267	JQ705069.1	JQ705069.1; A2z; Asia_NE_America_N; 37; A73G; T146C; T152C	16569	Asia_NE_America_N	A2z	A	A	37
3268	JQ705070.1	JQ705070.1; U5b2b3a1a; Asia_W_Europe_C; 38; A73G; C150T; A263G	16569	Asia_W_Europe_C	U5b2b3a1a	U	U5	38
3269	JQ705071.1	JQ705071.1; H4a1a1a; Asia_W_Europe; 18; A73G; T146C; A263G	16569	Asia_W_Europe	H4a1a1a	H	H	18
3270	JQ705072.1	JQ705072.1; J1c7a; Asia_W; 35; A73G; G185A; T195C	16570	Asia_W	J1c7a	J	J	35
3271	JQ705073.1	JQ705073.1; K2a6; Asia_W; 33; A73G; T146C; T152C	16569	Asia_W	K2a6	K	K	33
3272	JQ705074.1	JQ705074.1; H27e; Asia_W_Europe; 14; A263G; C315CC; A750G	16560	Asia_W_Europe	H27e	H	H	14
3273	JQ705075.1	JQ705075.1; N1a1a1a2; Africa_NE_Asia_W; 38; A73G; T152C; T199C	16571	Africa_NE_Asia_W	N1a1a1a2	N	N1	38
3274	JQ705076.1	JQ705076.1; H27e; Asia_W_Europe; 14; T152C; A263G; C315CC	16560	Asia_W_Europe	H27e	H	H	14
3275	JQ705077.1	JQ705077.1; L3d1b2; Africa_E; 38; A73G; C150T; T152C	16567	Africa_E	L3d1b2	L3	L3	38
3276	JQ705078.1	JQ705078.1; H1ap1; Asia_W_Europe; 15; T152C; A263G; C315CC	16569	Asia_W_Europe	H1ap1	H	H	15
3277	JQ705079.1	JQ705079.1; T2g1a1; Asia_W; 41; A73G; T152C; A200G	16570	Asia_W	T2g1a1	T	T	41
3278	JQ705080.1	JQ705080.1; H3g3; Asia_W_Europe; 15; T152C; A263G; C309CCT	16570	Asia_W_Europe	H3g3	H	H	15
3279	JQ705081.1	JQ705081.1; H3+16311; Asia_W_Europe; 12; T152C; A263G; C315CC	16569	Asia_W_Europe	H3+16311	H	H	12
3280	JQ705082.1	JQ705082.1; X2b7; Asia_W_America_N; 36; A73G; T146C; A153G	16570	Asia_W_America_N	X2b7	X	X	36

Supplementary Table S4 Human mitochondrial database (hMITO DB v1.0) metadata[a]

Row	Name (accession no.)	Description	Size	Geo_Region	Haplogroup	Macro_ Haplo	Macro_ Haplo2	Total Variants
3281	JQ705083.1	JQ705083.1; J1c16; Asia_W; 32; A73G; T152C; G185A	16569	Asia_W	J1c16	J	J	32
3282	JQ705084.1	JQ705084.1; H14a+146; Asia_W_Europe; 15; T146C; A263G; C309CCT	16570	Asia_W_Europe	H14a+146	H	H	15
3283	JQ705085.1	JQ705085.1; J1c3b; Asia_W; 30; A73G; G185A; A263G	16570	Asia_W	J1c3b	J	J	30
3284	JQ705086.1	JQ705086.1; V; Europe_S; 15; T72C; A263G; C309CCCT	16571	Europe_S	V	V	V	15
3285	JQ705087.1	JQ705087.1; L2a1a; Africa_W_C; 55; A73G; T146C; T195C	16571	Africa_W_C	L2a1a	L2	L2	55
3286	JQ705088.1	JQ705088.1; H6a1a; Asia_W_Europe; 14; T239C; A263G; C315CC	16569	Asia_W_Europe	H6a1a	H	H	14
3287	JQ705089.1	JQ705089.1; H16b; Asia_W_Europe; 14; A263G; C315CC; A750G	16569	Asia_W_Europe	H16b	H	H	14
3288	JQ705090.1	JQ705090.1; K2a2a1; Asia_W; 36; A73G; T146C; T152C	16569	Asia_W	K2a2a1	K	K	36
3289	JQ705091.1	JQ705091.1; H3aa; Asia_W_Europe; 15; C242T; A263G; C315CC	16569	Asia_W_Europe	H3aa	H	H	15
3290	JQ705092.1	JQ705092.1; K2b1a1; Asia_W; 34; A73G; T146C; A263G	16569	Asia_W	K2b1a1	K	K	34
3291	JQ705093.1	JQ705093.1; J1b1a1; Asia_W; 37; A73G; C242T; A263G	16569	Asia_W	J1b1a1	J	J	37
3292	JQ705094.1	JQ705094.1; J1c7a; Asia_W; 36; A73G; T146C; T152C	16569	Asia_W	J1c7a	J	J	36
3293	JQ705095.1	JQ705095.1; U5b1c1a1; Asia_W_Europe_C; 33; T55A; A73G; C150T	16570	Asia_W_Europe_C	U5b1c1a1	U	U5	33
3294	JQ705096.1	JQ705096.1; I5a1a; Asia_W_SW; 36; A73G; T199C; T204C	16562	Asia_W_SW	I5a1a	I	I	36
3295	JQ705097.1	JQ705097.1; T2g1a1; Asia_W; 40; A73G; A200G; A263G	16569	Asia_W	T2g1a1	T	T	40
3296	JQ705098.1	JQ705098.1; H13a1a; Asia_W_Europe; 15; A200G; A263G; C309CCCT	16571	Asia_W_Europe	H13a1a	H	H	15
3297	JQ705099.1	JQ705099.1; V7a; Europe_S; 21; T72C; A93G; T195C	16570	Europe_S	V7a	V	V	21
3298	JQ705100.1	JQ705100.1; K1a4a; Asia_W; 36; A73G; C150T; A263G	16569	Asia_W	K1a4a	K	K	36
3299	JQ705101.1	JQ705101.1; U5a1b3a1; Asia_W_Europe_C; 29; A73G; A249G; A263G	16569	Asia_W_Europe_C	U5a1b3a1	U	U5	29
3300	JQ705102.1	JQ705102.1; H1ae2; Asia_W_Europe; 14; A263G; C315CC; A750G	16569	Asia_W_Europe	H1ae2	H	H	14
3301	JQ705103.1	JQ705103.1; K1b2a1a1; Asia_W; 38; A73G; T146C; T195C	16573	Asia_W	K1b2a1a1	K	K	38
3302	JQ705104.1	JQ705104.1; R1a1a2; Asia_S_SE; 40; A73G; A263G; C295A	16572	Asia_S_SE	R1a1a2	R	R1	40
3303	JQ705105.1	JQ705105.1; U5b1b2; Asia_W_Europe_C; 30; A73G; C150T; T217C	16571	Asia_W_Europe_C	U5b1b2	U	U5	30
3304	JQ705106.1	JQ705106.1; T2b17a; Asia_W; 37; A73G; A263G; C315CC	16569	Asia_W	T2b17a	T	T	37
3305	JQ705107.1	JQ705107.1; H1bb; Asia_W_Europe; 13; T152C; A263G; C315CC	16573	Asia_W_Europe	H1bb	H	H	13
3306	JQ705108.1	JQ705108.1; W6b; Asia_W; 42; A73G; A189G; T195C	16571	Asia_W	W6b	W	W	42
3307	JQ705109.1	JQ705109.1; L0a2a2a; Africa_S_SE; 93; C64T; A93G; C150T	16558	Africa_S_SE	L0a2a2a	L0	L0	93
3308	JQ705110.1	JQ705110.1; H3k1a; Asia_W_Europe; 14; T152C; A263G; C315CC	16569	Asia_W_Europe	H3k1a	H	H	14
3309	JQ705111.1	JQ705111.1; U5a2+16362; Asia_W_Europe_C; 28; A73G; A263G; C315CC	16569	Asia_W_Europe_C	U5a2+16362	U	U5	28
3310	JQ705112.1	JQ705112.1; H4c1; Asia_W_Europe; 15; A73G; A263G; C315CC	16567	Asia_W_Europe	H4c1	H	H	15
3311	JQ705113.1	JQ705113.1; H3ap; Asia_W_Europe; 13; A263G; C315CC; A750G	16569	Asia_W_Europe	H3ap	H	H	13
3312	JQ705114.1	JQ705114.1; J1c1a; Asia_W; 34; A73G; G228A; A263G	16569	Asia_W	J1c1a	J	J	34
3313	JQ705115.1	JQ705115.1; L1b1a4; Africa_C; 82; A73G; T152C; C182T	16567	Africa_C	L1b1a4	L1	L1	82
3314	JQ705116.1	JQ705116.1; H3y; Asia_W_Europe; 14; A263G; C309CCT; T310C	16570	Asia_W_Europe	H3y	H	H	14
3315	JQ705117.1	JQ705117.1; U2e1a1a; Asia_S_W_Europe; 42; A73G; T152C; A263G	16572	Asia_S_W_Europe	U2e1a1a	U	U2	42
3316	JQ705118.1	JQ705118.1; X2e1a1; Asia_W_America_N; 32; A73G; A153G; T195C	16570	Asia_W_America_N	X2e1a1	X	X	32
3317	JQ705119.1	JQ705119.1; T2b2b1; Asia_W; 35; A73G; A263G; C315CC	16569	Asia_W	T2b2b1	T	T	35
3318	JQ705120.1	JQ705120.1; L2c; Africa_W_C; 63; A73G; A93G; T146C	16569	Africa_W_C	L2c	L2	L2	63
3319	JQ705121.1	JQ705121.1; U4a2b; Asia_N_Europe_N; 31; A73G; A189G; T195C	16570	Asia_N_Europe_N	U4a2b	U	U4	31
3320	JQ705122.1	JQ705122.1; K1c2a; Asia_W; 37; A73G; T146C; T152C	16568	Asia_W	K1c2a	K	K	37
3321	JQ705123.1	JQ705123.1; U5a2d1a; Asia_W_Europe_C; 32; A73G; A263G; C315CC	16567	Asia_W_Europe_C	U5a2d1a	U	U5	32

Supplementary Table S4 Human mitochondrial database (hMITO DB v1.0) metadata[a]

Row	Name (accession no.)	Description	Size	Geo_Region	Haplogroup	Macro_ Haplo	Macro_ Haplo2	Total Variants
3322	JQ705124.1	JQ705124.1; H3ag1; Asia_W_Europe; 12; A263G; C315CC; A750G	16569	Asia_W_Europe	H3ag1	H	H	12
3323	JQ705125.1	JQ705125.1; H1a6; Asia_W_Europe; 14; A73G; C151T; A263G	16569	Asia_W_Europe	H1a6	H	H	14
3324	JQ705126.1	JQ705126.1; H1b1d; Asia_W_Europe; 15; A93G; A263G; C315CC	16567	Asia_W_Europe	H1b1d	H	H	15
3325	JQ705127.1	JQ705127.1; HV0f; Asia_W; 13; T195C; A263G; C315CC	16569	Asia_W	HV0f	HV	HV	13
3326	JQ705128.1	JQ705128.1; H45a; Asia_W_Europe; 12; T199C; A263G; C315CC	16569	Asia_W_Europe	H45a	H	H	12
3327	JQ705129.1	JQ705129.1; J1c2f; Asia_W; 31; A73G; G185A; A188G	16569	Asia_W	J1c2f	J	J	31
3328	JQ705130.1	JQ705130.1; U4b; Asia_N_Europe_N; 32; A73G; T195C; T196C	16569	Asia_N_Europe_N	U4b	U	U4	32
3329	JQ705131.1	JQ705131.1; K1c1; Asia_W; 35; A73G; T146C; T152C	16568	Asia_W	K1c1	K	K	35
3330	JQ705132.1	JQ705132.1; U5a1a1; Asia_W_Europe_C; 30; A73G; A263G; C309CCT	16570	Asia_W_Europe_C	U5a1a1	U	U5	30
3331	JQ705133.1	JQ705133.1; T2a1a; Asia_W; 36; A73G; A263G; C309CCT	16570	Asia_W	T2a1a	T	T	36
3332	JQ705134.1	JQ705134.1; H29; Asia_W_Europe; 17; A93G; A263G; C315CC	16571	Asia_W_Europe	H29	H	H	17
3333	JQ705135.1	JQ705135.1; H1bb; Asia_W_Europe; 13; T152C; A263G; C315CC	16571	Asia_W_Europe	H1bb	H	H	13
3334	JQ705136.1	JQ705136.1; L3e2a1b1; Africa_E; 38; A73G; T146C; C150T	16567	Africa_E	L3e2a1b1	L3	L3	38
3335	JQ705137.1	JQ705137.1; L3h1b1a; Africa_E; 47; A73G; A189C; T195C	16567	Africa_E	L3h1b1a	L3	L3	47
3336	JQ705138.1	JQ705138.1; H1bn; Asia_W_Europe; 12; A263G; C315CC; A750G	16569	Asia_W_Europe	H1bn	H	H	12
3337	JQ705139.1	JQ705139.1; H2a2a1d; Asia_W_Europe; 4; C309CCT; T310C; A14357G	16570	Asia_W_Europe	H2a2a1d	H	H	4
3338	JQ705140.1	JQ705140.1; I1a1a; Asia_W_SW; 44; A73G; T199C; G203A	16572	Asia_W_SW	I1a1a	I	I	44
3339	JQ705141.1	JQ705141.1; J1c1b2; Asia_W; 32; A73G; G228A; A263G	16567	Asia_W	J1c1b2	J	J	32
3340	JQ705142.1	JQ705142.1; V3c; Europe_S; 16; T72C; A263G; C309CCT	16570	Europe_S	V3c	V	V	16
3341	JQ705143.1	JQ705143.1; H47a; Asia_W_Europe; 13; T152C; A263G; C309CCCT	16571	Asia_W_Europe	H47a	H	H	13
3342	JQ705144.1	JQ705144.1; H45b; Asia_W_Europe; 15; A263G; C309CCT; T310C	16570	Asia_W_Europe	H45b	H	H	15
3343	JQ705145.1	JQ705145.1; L2a1c3a1; Africa_W_C; 60; A73G; G143A; T146C	16569	Africa_W_C	L2a1c3a1	L2	L2	60
3344	JQ705146.1	JQ705146.1; H17b; Asia_W_Europe; 15; T146C; A263G; C315CC	16569	Asia_W_Europe	H17b	H	H	15
3345	JQ705147.1	JQ705147.1; B2o; Asia_SE_E_America_N_S; 33; A73G; A263G; T310C	16559	Asia_SE_E_America_N_S	B2o	B	B	33
3346	JQ705148.1	JQ705148.1; T2e; Asia_W; 34; A73G; C150T; A263G	16570	Asia_W	T2e	T	T	34
3347	JQ705149.1	JQ705149.1; K1a2c; Asia_W; 41; G54T; A73G; T146C	16573	Asia_W	K1a2c	K	K	41
3348	JQ705150.1	JQ705150.1; L2a1f; Africa_W_C; 54; A73G; T146C; T152C	16570	Africa_W_C	L2a1f	L2	L2	54
3349	JQ705151.1	JQ705151.1; H39a; Asia_W_Europe; 12; A263G; C309CCCT; T310C	16571	Asia_W_Europe	H39a	H	H	12
3350	JQ705152.1	JQ705152.1; U4a2d; Asia_N_Europe_N; 30; A73G; T195C; A263G	16570	Asia_N_Europe_N	U4a2d	U	U4	30
3351	JQ705153.1	JQ705153.1; C1b12; Asia_NE_America_N_S; 41; A73G; A249d; A263G	16564	Asia_NE_America_N_S	C1b12	C	C	41
3352	JQ705154.1	JQ705154.1; X2b8; Asia_W_America_N; 33; A73G; A153G; G225A	16570	Asia_W_America_N	X2b8	X	X	33
3353	JQ705155.1	JQ705155.1; X2c2; Asia_W_America_N; 32; A73G; A153G; T195C	16572	Asia_W_America_N	X2c2	X	X	32
3354	JQ705156.1	JQ705156.1; K2a4; Asia_W; 34; A73G; T146C; T152C	16569	Asia_W	K2a4	K	K	34
3355	JQ705157.1	JQ705157.1; H1+16239; Asia_W_Europe; 12; A263G; C309CCCT; T310C	16571	Asia_W_Europe	H1+16239	H	H	12
3356	JQ705158.1	JQ705158.1; U5b2a2a; Asia_W_Europe_C; 30; A73G; C150T; A263G	16569	Asia_W_Europe_C	U5b2a2a	U	U5	30
3357	JQ705159.1	JQ705159.1; H1c; Asia_W_Europe; 12; A263G; C315CC; T477C	16569	Asia_W_Europe	H1c	H	H	12
3358	JQ705160.1	JQ705160.1; H3; Asia_W_Europe; 14; A189G; A263G; C309CCCT	16571	Asia_W_Europe	H3	H	H	14
3359	JQ705161.1	JQ705161.1; C5c1a; Asia_NE_America_N_S; 45; A73G; T152C; A249d	16569	Asia_NE_America_N_S	C5c1a	C	C	45
3360	JQ705162.1	JQ705162.1; K1a4; Asia_W; 34; A73G; A263G; C315CC	16573	Asia_W	K1a4	K	K	34
3361	JQ705163.1	JQ705163.1; H+195; Asia_W_Europe; 13; G143A; T195C; A263G	16570	Asia_W_Europe	H+195	H	H	13
3362	JQ705164.1	JQ705164.1; J1c1b1a1; Asia_W; 36; A73G; G185A; T195C	16569	Asia_W	J1c1b1a1	J	J	36

Supplementary Table S4 Human mitochondrial database (hMITO DB v1.0) metadata[a]

Row	Name (accession no.)	Description	Size	Geo_Region	Haplogroup	Macro_ Haplo	Macro_ Haplo2	Total Variants
3363	JQ705165.1	JQ705165.1; H35; Asia_W_Europe; 12; A263G; C309CCT; T310C	16570	Asia_W_Europe	H35	H	H	12
3364	JQ705166.1	JQ705166.1; B2; Asia_SE_E_America_N_S; 29; A73G; T146C; A263G	16559	Asia_SE_E_America_N_S	B2	B	B	29
3365	JQ705167.1	JQ705167.1; H1c1; Asia_W_Europe; 13; T195C; A263G; C315CC	16569	Asia_W_Europe	H1c1	H	H	13
3366	JQ705168.1	JQ705168.1; V20; Europe_S; 18; T72C; T152C; A263G	16570	Europe_S	V20	V	V	18
3367	JQ705169.1	JQ705169.1; H1a3a1; Asia_W_Europe; 16; A73G; A263G; C309CCT	16570	Asia_W_Europe	H1a3a1	H	H	16
3368	JQ705170.1	JQ705170.1; H1e2a; Asia_W_Europe; 14; A263G; C309CCT; T310C	16570	Asia_W_Europe	H1e2a	H	H	14
3369	JQ705171.1	JQ705171.1; H1ax; Asia_W_Europe; 15; A263G; C309CCT; T310C	16570	Asia_W_Europe	H1ax	H	H	15
3370	JQ705172.1	JQ705172.1; H96; Asia_W_Europe; 22; G185A; A189G; A263G	16567	Asia_W_Europe	H96	H	H	22
3371	JQ705173.1	JQ705173.1; K1c2; Asia_W; 38; A73G; T146C; T152C	16570	Asia_W	K1c2	K	K	38
3372	JQ705174.1	JQ705174.1; H17a; Asia_W_Europe; 12; A263G; C309CCT; T310C	16570	Asia_W_Europe	H17a	H	H	12
3373	JQ705175.1	JQ705175.1; I2a3; Asia_W_SW; 41; A73G; T152C; T199C	16572	Asia_W_SW	I2a3	I	I	41
3374	JQ705176.1	JQ705176.1; H51; Asia_W_Europe; 10; A263G; C309CCT; T310C	16570	Asia_W_Europe	H51	H	H	10
3375	JQ705177.1	JQ705177.1; H4a1a1a; Asia_W_Europe; 19; A73G; A263G; C309CCT	16568	Asia_W_Europe	H4a1a1a	H	H	19
3376	JQ705178.1	JQ705178.1; K1c2; Asia_W; 35; A73G; T146C; T152C	16568	Asia_W	K1c2	K	K	35
3377	JQ705179.1	JQ705179.1; X2b6; Asia_W_America_N; 36; A73G; A153G; T195C	16569	Asia_W_America_N	X2b6	X	X	36
3378	JQ705180.1	JQ705180.1; U5b2b4; Asia_W_Europe_C; 33; A73G; C150T; A263G	16572	Asia_W_Europe_C	U5b2b4	U	U5	33
3379	JQ705181.1	JQ705181.1; H3g1; Asia_W_Europe; 14; T152C; A263G; C309CCT	16570	Asia_W_Europe	H3g1	H	H	14
3380	JQ705182.1	JQ705182.1; H1c3b; Asia_W_Europe; 19; A257G; A263G; C309CCCT	16571	Asia_W_Europe	H1c3b	H	H	19
3381	JQ705183.1	JQ705183.1; U5b1h; Asia_W_Europe_C; 27; A73G; C150T; A263G	16569	Asia_W_Europe_C	U5b1h	U	U5	27
3382	JQ705184.1	JQ705184.1; U5b1b1a1a1; Asia_W_Europe_C; 32; A73G; C150T; A263G	16569	Asia_W_Europe_C	U5b1b1a1a1	U	U5	32
3383	JQ705185.1	JQ705185.1; L2a1l2a; Africa_W_C; 55; A73G; T146C; T152C	16570	Africa_W_C	L2a1l2a	L2	L2	55
3384	JQ705186.1	JQ705186.1; N1b1a2; Africa_NE_Asia_W; 39; A73G; C151T; T152C	16567	Africa_NE_Asia_W	N1b1a2	N	N1	39
3385	JQ705187.1	JQ705187.1; I2c; Asia_W_SW; 37; A73G; T152C; T199C	16574	Asia_W_SW	I2c	I	I	37
3386	JQ705188.1	JQ705188.1; H6a1b3; Asia_W_Europe; 19; G203A; T204C; T239C	16570	Asia_W_Europe	H6a1b3	H	H	19
3387	JQ705189.1	JQ705189.1; I1a1d; Asia_W_SW; 46; A73G; T199C; G203A	16573	Asia_W_SW	I1a1d	I	I	46
3388	JQ705190.1	JQ705190.1; I1c1a; Asia_W_SW; 43; A73G; T199C; T204C	16573	Asia_W_SW	I1c1a	I	I	43
3389	JQ705191.1	JQ705191.1; I4a1; Asia_W_SW; 36; A73G; T199C; T204C	16571	Asia_W_SW	I4a1	I	I	36
3390	JQ705192.1	JQ705192.1; K1a3a3; Asia_W; 39; A73G; T146C; A263G	16570	Asia_W	K1a3a3	K	K	39
3391	JQ705193.1	JQ705193.1; H16b; Asia_W_Europe; 10; A263G; C315CC; A750G	16569	Asia_W_Europe	H16b	H	H	10
3392	JQ705194.1	JQ705194.1; H13a1a; Asia_W_Europe; 14; T146C; A263G; C309CCCT	16571	Asia_W_Europe	H13a1a	H	H	14
3393	JQ705195.1	JQ705195.1; H1c1; Asia_W_Europe; 13; T152C; A263G; C315CC	16569	Asia_W_Europe	H1c1	H	H	13
3394	JQ705196.1	JQ705196.1; R0a2m; Africa_NE_Asia_W; 23; T57TC; C64T; A263G	16572	Africa_NE_Asia_W	R0a2m	R	R0	23
3395	JQ705197.1	JQ705197.1; H3; Asia_W_Europe; 12; A263G; C309CCT; T310C	16570	Asia_W_Europe	H3	H	H	12
3396	JQ705198.1	JQ705198.1; U7b; Asia_W; 34; A73G; T152C; A263G	16568	Asia_W	U7b	U	U7	34
3397	JQ705199.1	JQ705199.1; T2b3e; Asia_W; 38; A73G; C151T; A263G	16570	Asia_W	T2b3e	T	T	38
3398	JQ705200.1	JQ705200.1; X2c1; Asia_W_America_N; 32; A73G; A153G; T195C	16570	Asia_W_America_N	X2c1	X	X	32
3399	JQ705201.1	JQ705201.1; H2a1f1; Asia_W_Europe; 11; T127C; T152C; A263G	16571	Asia_W_Europe	H2a1f1	H	H	11
3400	JQ705202.1	JQ705202.1; H10e1; Asia_W_Europe; 13; A263G; C309CCT; T310C	16570	Asia_W_Europe	H10e1	H	H	13
3401	JQ705203.1	JQ705203.1; H55b; Asia_W_Europe; 13; A153G; T204C; A263G	16571	Asia_W_Europe	H55b	H	H	13
3402	JQ705204.1	JQ705204.1; K1a1b1a; Asia_W; 39; A73G; C114T; A263G	16569	Asia_W	K1a1b1a	K	K	39
3403	JQ705205.1	JQ705205.1; H1a6; Asia_W_Europe; 16; A73G; C151T; A263G	16570	Asia_W_Europe	H1a6	H	H	16

Supplementary Table S4 Human mitochondrial database (hMITO DB v1.0) metadata[a]

Row	Name (accession no.)	Description	Size	Geo_Region	Haplogroup	Macro_ Haplo	Macro_ Haplo2	Total Variants
3404	JQ705206.1	JQ705206.1; H6a1a; Asia_W_Europe; 15; T239C; A263G; C309CCT	16570	Asia_W_Europe	H6a1a	H	H	15
3405	JQ705207.1	JQ705207.1; T1a1; Asia_W; 35; A73G; T152C; A263G	16570	Asia_W	T1a1	T	T	35
3406	JQ705208.1	JQ705208.1; T2b2b1; Asia_W; 35; A73G; A263G; C315CC	16569	Asia_W	T2b2b1	T	T	35
3407	JQ705209.1	JQ705209.1; W6a; Asia_W; 39; A73G; A189G; C194T	16573	Asia_W	W6a	W	W	39
3408	JQ705210.1	JQ705210.1; H1g1; Asia_W_Europe; 14; A263G; C309CCCT; T310C	16571	Asia_W_Europe	H1g1	H	H	14
3409	JQ705211.1	JQ705211.1; U5a1b1e; Asia_W_Europe_C; 31; A73G; A263G; C315CC	16567	Asia_W_Europe_C	U5a1b1e	U	U5	31
3410	JQ705212.1	JQ705212.1; J1c3g; Asia_W; 28; A73G; G185A; A263G	16569	Asia_W	J1c3g	J	J	28
3411	JQ705213.1	JQ705213.1; H1br; Asia_W_Europe; 13; A263G; C315CC; A750G	16569	Asia_W_Europe	H1br	H	H	13
3412	JQ705214.1	JQ705214.1; J2a1a1a2; Asia_W; 44; A73G; T146C; C150T	16570	Asia_W	J2a1a1a2	J	J	44
3413	JQ705215.1	JQ705215.1; K1a4a1c1; Asia_W; 38; A73G; T199C; T204C	16570	Asia_W	K1a4a1c1	K	K	38
3414	JQ705216.1	JQ705216.1; H1c1; Asia_W_Europe; 13; A263G; C315CC; T477C	16569	Asia_W_Europe	H1c1	H	H	13
3415	JQ705217.1	JQ705217.1; X2m2; Asia_W_America_N; 33; A73G; G143A; T195C	16570	Asia_W_America_N	X2m2	X	X	33
3416	JQ705218.1	JQ705218.1; K1a24a; Asia_W; 38; A73G; C150T; T195C	16569	Asia_W	K1a24a	K	K	38
3417	JQ705219.1	JQ705219.1; H4a1a1a1a1; Asia_W_Europe; 22; A73G; A263G; C315CC	16567	Asia_W_Europe	H4a1a1a1a1	H	H	22
3418	JQ705220.1	JQ705220.1; U5a1b1a; Asia_W_Europe_C; 32; A73G; T199C; A263G	16569	Asia_W_Europe_C	U5a1b1a	U	U5	32
3419	JQ705221.1	JQ705221.1; T2f3; Asia_W; 36; A73G; A263G; C309CCT	16559	Asia_W	T2f3	T	T	36
3420	JQ705222.1	JQ705222.1; C4a2a1; Asia_NE_America_N_S; 51; A73G; G207A; A249d	16570	Asia_NE_America_N_S	C4a2a1	C	C	51
3421	JQ705223.1	JQ705223.1; H2a1j; Asia_W_Europe; 11; A263G; C309CCCT; T310C	16571	Asia_W_Europe	H2a1j	H	H	11
3422	JQ705224.1	JQ705224.1; J1c2k; Asia_W; 34; A73G; G185A; A188G	16572	Asia_W	J1c2k	J	J	34
3423	JQ705225.1	JQ705225.1; K1b1a1+199; Asia_W; 43; A73G; T152C; T199C	16572	Asia_W	K1b1a1+199	K	K	43
3424	JQ705226.1	JQ705226.1; H23; Asia_W_Europe; 10; A263G; C315CC; A750G	16569	Asia_W_Europe	H23	H	H	10
3425	JQ705227.1	JQ705227.1; H2b; Asia_W_Europe; 10; T152C; A263G; C315CC	16569	Asia_W_Europe	H2b	H	H	10
3426	JQ705228.1	JQ705228.1; U5a2d1a; Asia_W_Europe_C; 33; A73G; A263G; C309CCT	16568	Asia_W_Europe_C	U5a2d1a	U	U5	33
3427	JQ705229.1	JQ705229.1; K1a4a1; Asia_W; 37; A73G; T146C; A263G	16570	Asia_W	K1a4a1	K	K	37
3428	JQ705230.1	JQ705230.1; H7d2a; Asia_W_Europe; 14; A263G; A291AA; C315CC	16570	Asia_W_Europe	H7d2a	H	H	14
3429	JQ705231.1	JQ705231.1; H1b; Asia_W_Europe; 13; A263G; C309CCT; T310C	16570	Asia_W_Europe	H1b	H	H	13
3430	JQ705232.1	JQ705232.1; H45a; Asia_W_Europe; 11; A263G; C315CC; A750G	16569	Asia_W_Europe	H45a	H	H	11
3431	JQ705233.1	JQ705233.1; H14a2a; Asia_W_Europe; 17; A263G; C309CCT; T310C	16570	Asia_W_Europe	H14a2a	H	H	17
3432	JQ705234.1	JQ705234.1; HV2a1; Asia_W; 29; T72C; A73G; C151T	16570	Asia_W	HV2a1	HV	HV	29
3433	JQ705235.1	JQ705235.1; H1; Asia_W_Europe; 11; A263G; T310C; A750G	16568	Asia_W_Europe	H1	H	H	11
3434	JQ705236.1	JQ705236.1; H1e4a; Asia_W_Europe; 12; A263G; C315CC; A750G	16569	Asia_W_Europe	H1e4a	H	H	12
3435	JQ705237.1	JQ705237.1; H5a1g2; Asia_W_Europe; 14; A263G; C315CC; C456T	16567	Asia_W_Europe	H5a1g2	H	H	14
3436	JQ705238.1	JQ705238.1; H1c17; Asia_W_Europe; 12; T46C; A263G; C315CC	16569	Asia_W_Europe	H1c17	H	H	12
3437	JQ705239.1	JQ705239.1; H1b; Asia_W_Europe; 17; T152C; A263G; C309CCT	16570	Asia_W_Europe	H1b	H	H	17
3438	JQ705240.1	JQ705240.1; U5b2a1a2; Asia_W_Europe_C; 31; A73G; C150T; A263G	16569	Asia_W_Europe_C	U5b2a1a2	U	U5	31
3439	JQ705241.1	JQ705241.1; H5a1h; Asia_W_Europe; 13; A263G; C315CC; C456T	16567	Asia_W_Europe	H5a1h	H	H	13
3440	JQ705242.1	JQ705242.1; T2b4+152; Asia_W; 34; A73G; T152C; A263G	16569	Asia_W	T2b4+152	T	T	34
3441	JQ705243.1	JQ705243.1; U5a1a1g; Asia_W_Europe_C; 28; A73G; A263G; C315CC	16569	Asia_W_Europe_C	U5a1a1g	U	U5	28
3442	JQ705244.1	JQ705244.1; I2d; Asia_W_SW; 37; A73G; T152C; T204C	16572	Asia_W_SW	I2d	I	I	37
3443	JQ705245.1	JQ705245.1; U5a1b1a1; Asia_W_Europe_C; 28; A73G; A263G; C315CC	16569	Asia_W_Europe_C	U5a1b1a1	U	U5	28
3444	JQ705246.1	JQ705246.1; K1b2a1a1; Asia_W; 39; A73G; T146C; T195C	16573	Asia_W	K1b2a1a1	K	K	39

Supplementary Table S4 Human mitochondrial database (hMITO DB v1.0) metadata[a]

Row	Name (accession no.)	Description	Size	Geo_Region	Haplogroup	Macro_ Haplo	Macro_ Haplo2	Total Variants
3445	JQ705247.1	JQ705247.1; J1b1a1; Asia_W; 36; A73G; T195C; C242T	16569	Asia_W	J1b1a1	J	J	36
3446	JQ705248.1	JQ705248.1; H4a1a4b; Asia_W_Europe; 21; T195C; A263G; C315CC	16567	Asia_W_Europe	H4a1a4b	H	H	21
3447	JQ705249.1	JQ705249.1; L1b1a6; Africa_C; 82; A73G; T152C; C182T	16567	Africa_C	L1b1a6	L1	L1	82
3448	JQ705250.1	JQ705250.1; L2a1a2; Africa_W_C; 57; A73G; T146C; T152C	16567	Africa_W_C	L2a1a2	L2	L2	57
3449	JQ705251.1	JQ705251.1; D2a1a; Asia_E_America_N_S; 42; A73G; A153G; A263G	16570	Asia_E_America_N_S	D2a1a	D	D	42
3450	JQ705252.1	JQ705252.1; H1g1; Asia_W_Europe; 14; T152C; A263G; C315CC	16569	Asia_W_Europe	H1g1	H	H	14
3451	JQ705253.1	JQ705253.1; V; Europe_S; 17; T72C; C194T; A263G	16570	Europe_S	V	V	V	17
3452	JQ705254.1	JQ705254.1; V2c; Europe_S; 16; T72C; A263G; C315CC	16569	Europe_S	V2c	V	V	16
3453	JQ705255.1	JQ705255.1; U4b1a1a; Asia_N_Europe_N; 32; A73G; T195C; A263G	16569	Asia_N_Europe_N	U4b1a1a	U	U4	32
3454	JQ705256.1	JQ705256.1; H1e1; Asia_W_Europe; 13; A263G; C315CC; A750G	16569	Asia_W_Europe	H1e1	H	H	13
3455	JQ705257.1	JQ705257.1; K1b2a; Asia_W; 35; A73G; T146C; T195C	16571	Asia_W	K1b2a	K	K	35
3456	JQ705258.1	JQ705258.1; H27; Asia_W_Europe; 11; A263G; C315CC; A750G	16569	Asia_W_Europe	H27	H	H	11
3457	JQ705259.1	JQ705259.1; B2y1; Asia_SE_E_America_N_S; 31; A73G; T146C; A263G	16561	Asia_SE_E_America_N_S	B2y1	B	B	31
3458	JQ705260.1	JQ705260.1; H24a; Asia_W_Europe; 11; A263G; C309CCCT; T310C	16571	Asia_W_Europe	H24a	H	H	11
3459	JQ705261.1	JQ705261.1; HV+16311; Asia_W_Europe; 13; T195C; A263G; C309CCT	16570	Asia_W_Europe	HV+16311	H	H	13
3460	JQ705262.1	JQ705262.1; M37e(M37e); Asia_S; 3; C315CC; A8701G; C16295T	16569	Asia_S	M37e(M37e)	M	M	3
3461	JQ705263.1	JQ705263.1; H4a1a1a; Asia_W_Europe; 19; A73G; A263G; C309CCT	16568	Asia_W_Europe	H4a1a1a	H	H	19
3462	JQ705264.1	JQ705264.1; H10e2; Asia_W_Europe; 13; A263G; C309CCT; T310C	16570	Asia_W_Europe	H10e2	H	H	13
3463	JQ705265.1	JQ705265.1; H2a2a1g; Asia_W_Europe; 2; C315CC; T16189C;	16569	Asia_W_Europe	H2a2a1g	H	H	2
3464	JQ705266.1	JQ705266.1; T1a1; Asia_W; 36; A73G; T152C; T195C	16570	Asia_W	T1a1	T	T	36
3465	JQ705267.1	JQ705267.1; U5b1e1; Asia_W_Europe_C; 30; A73G; C150T; T152C	16567	Asia_W_Europe_C	U5b1e1	U	U5	30
3466	JQ705268.1	JQ705268.1; N1b1a2; Africa_NE_Asia_W; 39; A73G; T152C; A263G	16570	Africa_NE_Asia_W	N1b1a2	N	N1	39
3467	JQ705269.1	JQ705269.1; T1a1; Asia_W; 36; A73G; T152C; T195C	16570	Asia_W	T1a1	T	T	36
3468	JQ705270.1	JQ705270.1; K1a4a1a2a; Asia_W; 40; A73G; A263G; C309CCT	16574	Asia_W	K1a4a1a2a	K	K	40
3469	JQ705271.1	JQ705271.1; J1c7a; Asia_W; 36; A73G; G185A; T195C	16572	Asia_W	J1c7a	J	J	36
3470	JQ705272.1	JQ705272.1; U5a1h; Asia_W_Europe_C; 37; A73G; C150T; G207A	16570	Asia_W_Europe_C	U5a1h	U	U5	37
3471	JQ705273.1	JQ705273.1; V; Europe_S; 15; T72C; A263G; C309CCT	16570	Europe_S	V	V	V	15
3472	JQ705274.1	JQ705274.1; H10; Asia_W_Europe; 12; A263G; C309CCT; T310C	16570	Asia_W_Europe	H10	H	H	12
3473	JQ705275.1	JQ705275.1; L1c1a; Africa_C; 93; A73G; C151T; T152C	16566	Africa_C	L1c1a	L1	L1	93
3474	JQ705276.1	JQ705276.1; H66a; Asia_W_Europe; 11; A263G; C315CC; A750G	16569	Asia_W_Europe	H66a	H	H	11
3475	JQ705277.1	JQ705277.1; U6a1a1; Asia_SW_Africa_N; 30; A73G; A263G; C309CCT	16570	Asia_SW_Africa_N	U6a1a1	U	U6	30
3476	JQ705278.1	JQ705278.1; W5a2b; Asia_W; 42; A73G; C150T; A189G	16567	Asia_W	W5a2b	W	W	42
3477	JQ705279.1	JQ705279.1; U5a1a2b1; Asia_W_Europe_C; 33; A73G; A263G; C309CCT	16570	Asia_W_Europe_C	U5a1a2b1	U	U5	33
3478	JQ705280.1	JQ705280.1; J1c3b; Asia_W; 30; A73G; G185A; A263G	16569	Asia_W	J1c3b	J	J	30
3479	JQ705281.1	JQ705281.1; U8a1a1a1; Asia_W_Europe ; 33; A73G; A263G; T282C	16570	Asia_W_Europe	U8a1a1a1	U	U8	33
3480	JQ705282.1	JQ705282.1; N9a2a; Africa_NE_Asia_E; 28; A73G; C150T; A263G	16570	Africa_NE_Asia_E	N9a2a	N	N9	28
3481	JQ705283.1	JQ705283.1; H48; Asia_W_Europe; 12; A263G; C264T; C315CC	16569	Asia_W_Europe	H48	H	H	12
3482	JQ705284.1	JQ705284.1; H10a1; Asia_W_Europe; 14; A263G; C309CCT; T310C	16570	Asia_W_Europe	H10a1	H	H	14
3483	JQ705285.1	JQ705285.1; L3e4a; Africa_E; 36; A73G; C150T; A263G	16577	Africa_E	L3e4a	L3	L3	36
3484	JQ705286.1	JQ705286.1; W3a1a1; Asia_W; 40; A73G; A189G; T195C	16570	Asia_W	W3a1a1	W	W	40
3485	JQ705287.1	JQ705287.1; H14a; Asia_W_Europe; 20; A263G; C309CCT; T310C	16570	Asia_W_Europe	H14a	H	H	20

Supplementary Table S4 Human mitochondrial database (hMITO DB v1.0) metadata[a]

Row	Name (accession no.)	Description	Size	Geo_Region	Haplogroup	Macro_ Haplo	Macro_ Haplo2	Total Variants
3486	JQ705288.1	JQ705288.1; K1a; Asia_W; 32; A73G; T146C; T152C	16569	Asia_W	K1a	K	K	32
3487	JQ705289.1	JQ705289.1; K1b2a1a; Asia_W; 38; A73G; T146C; T195C	16569	Asia_W	K1b2a1a	K	K	38
3488	JQ705290.1	JQ705290.1; U5a1c1; Asia_W_Europe_C; 29; A73G; T195C; A263G	16571	Asia_W_Europe_C	U5a1c1	U	U5	29
3489	JQ705291.1	JQ705291.1; K1c2a; Asia_W; 38; A73G; T146C; T152C	16569	Asia_W	K1c2a	K	K	38
3490	JQ705292.1	JQ705292.1; U1b3; Asia_W; 37; A73G; T146C; T152C	16569	Asia_W	U1b3	U	U1	37
3491	JQ705293.1	JQ705293.1; A2f1a; Asia_NE_America_N; 40; C64T; A73G; T146C	16567	Asia_NE_America_N	A2f1a	A	A	40
3492	JQ705294.1	JQ705294.1; H1j; Asia_W_Europe; 10; A263G; C315CC; A750G	16569	Asia_W_Europe	H1j	H	H	10
3493	JQ705295.1	JQ705295.1; R1a1a; Asia_S_SE; 39; A73G; T204C; G207A	16571	Asia_S_SE	R1a1a	R	R1	39
3494	JQ705296.1	JQ705296.1; H1; Asia_W_Europe; 12; A263G; C309CCCT; T310C	16569	Asia_W_Europe	H1	H	H	12
3495	JQ705297.1	JQ705297.1; U5a1b1c1; Asia_W_Europe_C; 29; A73G; A263G; C315CC	16569	Asia_W_Europe_C	U5a1b1c1	U	U5	29
3496	JQ705298.1	JQ705298.1; U5b2a4a; Asia_W_Europe_C; 33; A73G; C150T; A263G	16569	Asia_W_Europe_C	U5b2a4a	U	U5	33
3497	JQ705299.1	JQ705299.1; H79a; Asia_W_Europe; 12; A263G; C315CC; CA522d	16567	Asia_W_Europe	H79a	H	H	12
3498	JQ705300.1	JQ705300.1; T2; Asia_W; 34; A73G; A263G; C309CCT	16570	Asia_W	T2	T	T	34
3499	JQ705301.1	JQ705301.1; K2a6; Asia_W; 34; A73G; T146C; T152C	16570	Asia_W	K2a6	K	K	34
3500	JQ705302.1	JQ705302.1; V19; Europe_S; 17; T72C; C150T; A263G	16570	Europe_S	V19	V	V	17
3501	JQ705303.1	JQ705303.1; I4a; Asia_W_SW; 34; A73G; T199C; T204C	16567	Asia_W_SW	I4a	I	I	34
3502	JQ705304.1	JQ705304.1; I2; Asia_W_SW; 36; A73G; T152C; T199C	16573	Asia_W_SW	I2	I	I	36
3503	JQ705305.1	JQ705305.1; R0a4; Africa_NE_Asia_W; 22; T57TC; C64T; C150T	16571	Africa_NE_Asia_W	R0a4	R	R0	22
3504	JQ705306.1	JQ705306.1; H1a5; Asia_W_Europe; 16; A73G; T152C; A263G	16570	Asia_W_Europe	H1a5	H	H	16
3505	JQ705307.1	JQ705307.1; H7f; Asia_W_Europe; 11; A263G; C315CC; A750G	16569	Asia_W_Europe	H7f	H	H	11
3506	JQ705308.1	JQ705308.1; J1c3e2; Asia_W; 32; A73G; G185A; G228A	16569	Asia_W	J1c3e2	J	J	32
3507	JQ705309.1	JQ705309.1; V; Europe_S; 19; T72C; A263G; C309CCCT	16571	Europe_S	V	V	V	19
3508	JQ705310.1	JQ705310.1; L3k1; Africa_E; 39; A73G; C150T; T152C	16569	Africa_E	L3k1	L3	L3	39
3509	JQ705311.1	JQ705311.1; H1a; Asia_W_Europe; 13; A73G; A263G; C309CCT	16570	Asia_W_Europe	H1a	H	H	13
3510	JQ705312.1	JQ705312.1; H28a; Asia_W_Europe; 14; C186A; A263G; C309CCCT	16571	Asia_W_Europe	H28a	H	H	14
3511	JQ705313.1	JQ705313.1; W3a; Asia_W; 35; A73G; A189G; C194T	16569	Asia_W	W3a	W	W	35
3512	JQ705314.1	JQ705314.1; A2ae; Asia_NE_America_N; 36; C64T; A73G; T146C	16568	Asia_NE_America_N	A2ae	A	A	36
3513	JQ705315.1	JQ705315.1; H5a1; Asia_W_Europe; 14; A263G; C309CCT; T310C	16570	Asia_W_Europe	H5a1	H	H	14
3514	JQ705316.1	JQ705316.1; U5b2a5; Asia_W_Europe_C; 34; A73G; C150T; A263G	16570	Asia_W_Europe_C	U5b2a5	U	U5	34
3515	JQ705317.1	JQ705317.1; K1c2; Asia_W; 38; A73G; T146C; T152C	16571	Asia_W	K1c2	K	K	38
3516	JQ705318.1	JQ705318.1; U5a1a1d1; Asia_W_Europe_C; 32; A73G; G185A; A189G	16570	Asia_W_Europe_C	U5a1a1d1	U	U5	32
3517	JQ705319.1	JQ705319.1; J1d1a1; Asia_W; 37; A73G; T152C; A263G	16573	Asia_W	J1d1a1	J	J	37
3518	JQ705320.1	JQ705320.1; L3e4a1; Africa_E; 34; A73G; C150T; A263G	16568	Africa_E	L3e4a1	L3	L3	34
3519	JQ705321.1	JQ705321.1; X2b4; Asia_W_America_N; 33; A73G; A153G; T195C	16570	Asia_W_America_N	X2b4	X	X	33
3520	JQ705322.1	JQ705322.1; J1c7a; Asia_W; 35; A73G; T146C; G185A	16569	Asia_W	J1c7a	J	J	35
3521	JQ705323.1	JQ705323.1; J2a1a1a3; Asia_W; 44; A73G; C150T; T152C	16568	Asia_W	J2a1a1a3	J	J	44
3522	JQ705324.1	JQ705324.1; H10e; Asia_W_Europe; 11; A263G; C309CCCT; T310C	16571	Asia_W_Europe	H10e	H	H	11
3523	JQ705325.1	JQ705325.1; H6a1a; Asia_W_Europe; 15; T239C; A249d; A263G	16568	Asia_W_Europe	H6a1a	H	H	15
3524	JQ705326.1	JQ705326.1; U5a1i1; Asia_W_Europe_C; 30; A73G; A263G; C315CC	16569	Asia_W_Europe_C	U5a1i1	U	U5	30
3525	JQ705327.1	JQ705327.1; K2b1a1a; Asia_W; 35; A73G; T146C; A263G	16569	Asia_W	K2b1a1a	K	K	35
3526	JQ705328.1	JQ705328.1; T2b; Asia_W; 34; A73G; A263G; C315CC	16569	Asia_W	T2b	T	T	34

Supplementary Table S4 Human mitochondrial database (hMITO DB v1.0) metadata[a]

Row	Name (accession no.)	Description	Size	Geo_Region	Haplogroup	Macro_ Haplo	Macro_ Haplo2	Total Variants
3527	JQ705329.1	JQ705329.1; H3a1a; Asia_W_Europe; 14; T152C; A263G; C309CCT	16570	Asia_W_Europe	H3a1a	H	H	14
3528	JQ705330.1	JQ705330.1; H1e1a; Asia_W_Europe; 13; A263G; C315CC; T408A	16569	Asia_W_Europe	H1e1a	H	H	13
3529	JQ705331.1	JQ705331.1; H1e1a8; Asia_W_Europe; 18; T195C; A263G; C315CC	16571	Asia_W_Europe	H1e1a8	H	H	18
3530	JQ705332.1	JQ705332.1; J1c7a; Asia_W; 35; A73G; T146C; G185A	16569	Asia_W	J1c7a	J	J	35
3531	JQ705333.1	JQ705333.1; X2b4; Asia_W_America_N; 33; A73G; A153G; T195C	16571	Asia_W_America_N	X2b4	X	X	33
3532	JQ705334.1	JQ705334.1; X2b+226+16192; Asia_W_America_N; 33; A73G; A153G; T195C	16572	Asia_W_America_N	X2b+226+1619	X	X	33
3533	JQ705335.1	JQ705335.1; T1a1; Asia_W; 37; A73G; T152C; T195C	16570	Asia_W	T1a1	T	T	37
3534	JQ705336.1	JQ705336.1; K1a11a1; Asia_W; 38; A16T; A73G; C150T	16560	Asia_W	K1a11a1	K	K	38
3535	JQ705337.1	JQ705337.1; U5b2a4; Asia_W_Europe_C; 31; A73G; C150T; A263G	16569	Asia_W_Europe_C	U5b2a4	U	U5	31
3536	JQ705338.1	JQ705338.1; T2b21b; Asia_W; 39; A73G; T152C; A263G	16570	Asia_W	T2b21b	T	T	39
3537	JQ705339.1	JQ705339.1; H4a1a1a; Asia_W_Europe; 19; A73G; A263G; C309CCT	16568	Asia_W_Europe	H4a1a1a	H	H	19
3538	JQ705340.1	JQ705340.1; H3c2b1; Asia_W_Europe; 14; T195C; A263G; C315CC	16569	Asia_W_Europe	H3c2b1	H	H	14
3539	JQ705341.1	JQ705341.1; H3ao1; Asia_W_Europe; 15; T152C; T195C; A263G	16570	Asia_W_Europe	H3ao1	H	H	15
3540	JQ705342.1	JQ705342.1; U4b1b1; Asia_N_Europe_N; 33; A73G; T146C; T152C	16571	Asia_N_Europe_N	U4b1b1	U	U4	33
3541	JQ705343.1	JQ705343.1; H1aa1; Asia_W_Europe; 13; A263G; C315CC; A750G	16569	Asia_W_Europe	H1aa1	H	H	13
3542	JQ705344.1	JQ705344.1; HV4a1; Asia_W; 18; T146C; A263G; C309CCT	16570	Asia_W	HV4a1	HV	HV	18
3543	JQ705345.1	JQ705345.1; H13a1a1; Asia_W_Europe; 17; C150T; T152C; A263G	16570	Asia_W_Europe	H13a1a1	H	H	17
3544	JQ705346.1	JQ705346.1; U8a1a1b1; Asia_W_Europe ; 33; A73G; A263G; T282C	16570	Asia_W_Europe	U8a1a1b1	U	U8	33
3545	JQ705347.1	JQ705347.1; U8a1a1; Asia_W_Europe ; 30; A73G; A263G; T282C	16570	Asia_W_Europe	U8a1a1	U	U8	30
3546	JQ705348.1	JQ705348.1; A2n; Asia_NE_America_N; 45; C64T; A73G; T146C	16568	Asia_NE_America_N	A2n	A	A	45
3547	JQ705349.1	JQ705349.1; B2c2b; Asia_SE_E_America_N_S; 38; A73G; T146C; T152C	16562	Asia_SE_E_America_N_S	B2c2b	B	B	38
3548	JQ705350.1	JQ705350.1; H1b3; Asia_W_Europe; 17; A263G; C309CCCT; T310C	16571	Asia_W_Europe	H1b3	H	H	17
3549	JQ705351.1	JQ705351.1; T2b; Asia_W; 36; A73G; A263G; C309CCT	16570	Asia_W	T2b	T	T	36
3550	JQ705352.1	JQ705352.1; T1a3a; Asia_W; 38; A73G; T152C; T195C	16570	Asia_W	T1a3a	T	T	38
3551	JQ705353.1	JQ705353.1; T1a1; Asia_W; 36; A73G; T152C; T195C	16571	Asia_W	T1a1	T	T	36
3552	JQ705354.1	JQ705354.1; K1a2a; Asia_W; 38; A73G; T152C; A263G	16581	Asia_W	K1a2a	K	K	38
3553	JQ705355.1	JQ705355.1; U5b1b2; Asia_W_Europe_C; 28; A73G; C150T; T217C	16571	Asia_W_Europe_C	U5b1b2	U	U5	28
3554	JQ705356.1	JQ705356.1; J2b1a1; Asia_W; 41; A73G; C150T; T152C	16570	Asia_W	J2b1a1	J	J	41
3555	JQ705357.1	JQ705357.1; T2b6+146; Asia_W; 39; A73G; T146C; A263G	16569	Asia_W	T2b6+146	T	T	39
3556	JQ705358.1	JQ705358.1; J2a1a1; Asia_W; 39; A73G; C150T; T152C	16570	Asia_W	J2a1a1	J	J	39
3557	JQ705359.1	JQ705359.1; U5a2a1+152; Asia_W_Europe_C; 29; A73G; T152C; A263G	16569	Asia_W_Europe_C	U5a2a1+152	U	U5	29
3558	JQ705360.1	JQ705360.1; J1c3; Asia_W; 31; A73G; G228A; A263G	16569	Asia_W	J1c3	J	J	31
3559	JQ705361.1	JQ705361.1; L3b1a; Africa_E; 39; A73G; A263G; C315CC	16566	Africa_E	L3b1a	L3	L3	39
3560	JQ705362.1	JQ705362.1; H1av; Asia_W_Europe; 14; A263G; C309CCCT; T310C	16571	Asia_W_Europe	H1av	H	H	14
3561	JQ705363.1	JQ705363.1; U4a2a; Asia_N_Europe_N; 27; A73G; T195C; A263G	16568	Asia_N_Europe_N	U4a2a	U	U4	27
3562	JQ705364.1	JQ705364.1; I1c1a; Asia_W_SW; 44; A73G; T199C; T204C	16573	Asia_W_SW	I1c1a	I	I	44
3563	JQ705365.1	JQ705365.1; H2a2b1; Asia_W_Europe; 11; C182T; A263G; C309CCCT	16571	Asia_W_Europe	H2a2b1	H	H	11
3564	JQ705366.1	JQ705366.1; H27; Asia_W_Europe; 11; A263G; C315CC; A750G	16569	Asia_W_Europe	H27	H	H	11
3565	JQ705367.1	JQ705367.1; H1b1+16362; Asia_W_Europe; 17; A263G; C309CCCT; T310C	16569	Asia_W_Europe	H1b1+16362	H	H	17
3566	JQ705368.1	JQ705368.1; HV9a; Asia_W; 15; T131C; T152C; A263G	16570	Asia_W	HV9a	HV	HV	15
3567	JQ705369.1	JQ705369.1; R0a1a; Africa_NE_Asia_W; 25; T58C; C64T; T146C	16570	Africa_NE_Asia_W	R0a1a	R	R0	25

Supplementary Table S4 Human mitochondrial database (hMITO DB v1.0) metadata[a]

Row	Name (accession no.)	Description	Size	Geo_Region	Haplogroup	Macro_ Haplo	Macro_ Haplo2	Total Variants
3568	JQ705370.1	JQ705370.1; H2a1k; Asia_W_Europe; 11; A263G; C309CCT; T310C	16571	Asia_W_Europe	H2a1k	H	H	11
3569	JQ705371.1	JQ705371.1; H1ap1; Asia_W_Europe; 15; T152C; A263G; C309CCT	16570	Asia_W_Europe	H1ap1	H	H	15
3570	JQ705372.1	JQ705372.1; T1b3; Asia_W; 36; A73G; A263G; C309CCT	16572	Asia_W	T1b3	T	T	36
3571	JQ705373.1	JQ705373.1; H; Asia_W_Europe; 10; A263G; C315CC; A750G	16569	Asia_W_Europe	H	H	H	10
3572	JQ705374.1	JQ705374.1; X2b6a; Asia_W_America_N; 36; A73G; A153G; T195C	16569	Asia_W_America_N	X2b6a	X	X	36
3573	JQ705375.1	JQ705375.1; M7c1a1a1; Asia_E_SE; 43; A73G; T146C; T199C	16568	Asia_E_SE	M7c1a1a1	M	M7	43
3574	JQ705376.1	JQ705376.1; I1b; Asia_W_SW; 39; A73G; T199C; T204C	16575	Asia_W_SW	I1b	I	I	39
3575	JQ705377.1	JQ705377.1; I3b; Asia_W_SW; 38; A73G; T152C; T199C	16576	Asia_W_SW	I3b	I	I	38
3576	JQ705378.1	JQ705378.1; I1a1a3; Asia_W_SW; 49; A73G; A189G; T199C	16573	Asia_W_SW	I1a1a3	I	I	49
3577	JQ705379.1	JQ705379.1; I2; Asia_W_SW; 36; A73G; T152C; T199C	16573	Asia_W_SW	I2	I	I	36
3578	JQ705380.1	JQ705380.1; A2z; Asia_NE_America_N; 36; A73G; T146C; T152C	16569	Asia_NE_America_N	A2z	A	A	36
3579	JQ705381.1	JQ705381.1; C4c2; Asia_NE_America_N_S; 47; C96T; GGAGCC100-; C114T	16565	Asia_NE_America_N_S	C4c2	C	C	47
3580	JQ705382.1	JQ705382.1; I6a; Asia_W_SW; 41; A73G; T199C; G203A	16571	Asia_W_SW	I6a	I	I	41
3581	JQ705383.1	JQ705383.1; H1u; Asia_W_Europe; 15; A263G; C309CCT; T310C	16568	Asia_W_Europe	H1u	H	H	15
3582	JQ705384.1	JQ705384.1; U2e1b1; Asia_S_W_Europe; 40; A73G; T152C; T217C	16571	Asia_S_W_Europe	U2e1b1	U	U2	40
3583	JQ705385.1	JQ705385.1; H2a1; Asia_W_Europe; 10; A263G; C309CCT; T310C	16570	Asia_W_Europe	H2a1	H	H	10
3584	JQ705386.1	JQ705386.1; U2e2a1c; Asia_S_W_Europe; 42; A73G; T152C; T217C	16570	Asia_S_W_Europe	U2e2a1c	U	U2	42
3585	JQ705387.1	JQ705387.1; U3a1; Africa_NE_Asia_W; 33; A73G; C150T; A263G	16570	Africa_NE_Asia_W	U3a1	U	U3	33
3586	JQ705388.1	JQ705388.1; J1c4; Asia_W; 32; A73G; G185A; G228A	16570	Asia_W	J1c4	J	J	32
3587	JQ705389.1	JQ705389.1; X2m2; Asia_W_America_N; 33; A73G; G143A; T195C	16570	Asia_W_America_N	X2m2	X	X	33
3588	JQ705390.1	JQ705390.1; J2a1a1b; Asia_W; 40; A73G; C150T; T152C	16569	Asia_W	J2a1a1b	J	J	40
3589	JQ705391.1	JQ705391.1; H1y; Asia_W_Europe; 17; A73G; A263G; C315CC	16569	Asia_W_Europe	H1y	H	H	17
3590	JQ705392.1	JQ705392.1; H56; Asia_W_Europe; 11; A263G; C309CCCT; T310C	16571	Asia_W_Europe	H56	H	H	11
3591	JQ705393.1	JQ705393.1; K1a1a2a1; Asia_W; 37; A73G; A263G; C309CCT	16571	Asia_W	K1a1a2a1	K	K	37
3592	JQ705394.1	JQ705394.1; J1c7a; Asia_W; 35; A73G; T146C; G185A	16569	Asia_W	J1c7a	J	J	35
3593	JQ705395.1	JQ705395.1; H1; Asia_W_Europe; 12; A263G; C309CCT; T310C	16570	Asia_W_Europe	H1	H	H	12
3594	JQ705396.1	JQ705396.1; V11; Europe_S; 19; T72C; A263G; C309CCCT	16571	Europe_S	V11	V	V	19
3595	JQ705397.1	JQ705397.1; J1c5b; Asia_W; 30; A73G; G185A; G228A	16569	Asia_W	J1c5b	J	J	30
3596	JQ705398.1	JQ705398.1; U5a1b; Asia_W_Europe_C; 24; A73G; A263G; C315CC	16569	Asia_W_Europe_C	U5a1b	U	U5	24
3597	JQ705399.1	JQ705399.1; H13a1a1; Asia_W_Europe; 15; A263G; C309CCCT; T310C	16571	Asia_W_Europe	H13a1a1	H	H	15
3598	JQ705400.1	JQ705400.1; K1b1a1; Asia_W; 41; A73G; T152C; A263G	16572	Asia_W	K1b1a1	K	K	41
3599	JQ705401.1	JQ705401.1; M20; Asia_S; 49; A73G; T152C; G225A	16566	Asia_S	M20	M	M20	49
3600	JQ705402.1	JQ705402.1; H6a1a; Asia_W_Europe; 18; T152C; T239C; A263G	16569	Asia_W_Europe	H6a1a	H	H	18
3601	JQ705403.1	JQ705403.1; H83; Asia_W_Europe; 9; A263G; C315CC; A750G	16569	Asia_W_Europe	H83	H	H	9
3602	JQ705404.1	JQ705404.1; K1a4a1d; Asia_W; 36; A73G; A263G; C315CC	16569	Asia_W	K1a4a1d	K	K	36
3603	JQ705405.1	JQ705405.1; U5b1e1; Asia_W_Europe_C; 32; A73G; C150T; T152C	16567	Asia_W_Europe_C	U5b1e1	U	U5	32
3604	JQ705406.1	JQ705406.1; K1c1d; Asia_W; 39; A73G; T146C; T152C	16566	Asia_W	K1c1d	K	K	39
3605	JQ705407.1	JQ705407.1; H66a; Asia_W_Europe; 10; A263G; C315CC; A750G	16569	Asia_W_Europe	H66a	H	H	10
3606	JQ705408.1	JQ705408.1; H2a2; Asia_W_Europe; 7; A263G; C309CCCT; T310C	16571	Asia_W_Europe	H2a2	H	H	7
3607	JQ705409.1	JQ705409.1; U4a1b1; Asia_N_Europe_N; 35; A73G; T152C; T195C	16570	Asia_N_Europe_N	U4a1b1	U	U4	35
3608	JQ705410.1	JQ705410.1; L3d2b; Africa_E; 34; A73G; C150T; T199C	16567	Africa_E	L3d2b	L3	L3	34

Supplementary Table S4 Human mitochondrial database (hMITO DB v1.0) metadata[a]

Row	Name (accession no.)	Description	Size	Geo_Region	Haplogroup	Macro_ Haplo	Macro_ Haplo2	Total Variants
3609	JQ705411.1	JQ705411.1; H2a1g; Asia_W_Europe; 9; A263G; C315CC; A750G	16569	Asia_W_Europe	H2a1g	H	H	9
3610	JQ705412.1	JQ705412.1; H5a1m; Asia_W_Europe; 16; T152C; A263G; C309CCT	16568	Asia_W_Europe	H5a1m	H	H	16
3611	JQ705413.1	JQ705413.1; H2a2b3; Asia_W_Europe; 8; A263G; C309CCT; T310C	16570	Asia_W_Europe	H2a2b3	H	H	8
3612	JQ705414.1	JQ705414.1; J1c4; Asia_W; 29; A73G; G228A; A263G	16569	Asia_W	J1c4	J	J	29
3613	JQ705415.1	JQ705415.1; H5a1g2; Asia_W_Europe; 15; A263G; C315CC; C456T	16567	Asia_W_Europe	H5a1g2	H	H	15
3614	JQ705416.1	JQ705416.1; HV17a; Asia_W; 17; T152C; T195C; A263G	16570	Asia_W	HV17a	HV	HV	17
3615	JQ705417.1	JQ705417.1; T2b3e; Asia_W; 38; A73G; A263G; C309CCT	16570	Asia_W	T2b3e	T	T	38
3616	JQ705418.1	JQ705418.1; U5b1b1f; Asia_W_Europe_C; 29; A73G; C150T; A263G	16569	Asia_W_Europe_C	U5b1b1f	U	U5	29
3617	JQ705419.1	JQ705419.1; U5b2a4a; Asia_W_Europe_C; 32; A73G; C150T; A263G	16569	Asia_W_Europe_C	U5b2a4a	U	U5	32
3618	JQ705420.1	JQ705420.1; H7a1b; Asia_W_Europe; 14; A263G; C309CCT; T310C	16570	Asia_W_Europe	H7a1b	H	H	14
3619	JQ705421.1	JQ705421.1; J1c2n1; Asia_W; 33; A73G; G185A; A188G	16571	Asia_W	J1c2n1	J	J	33
3620	JQ705422.1	JQ705422.1; H3aa; Asia_W_Europe; 12; A263G; C315CC; A750G	16569	Asia_W_Europe	H3aa	H	H	12
3621	JQ705423.1	JQ705423.1; H13a1a; Asia_W_Europe; 14; A263G; C309CCT; T310C	16570	Asia_W_Europe	H13a1a	H	H	14
3622	JQ705424.1	JQ705424.1; J1c14; Asia_W; 36; A73G; G185A; T195C	16572	Asia_W	J1c14	J	J	36
3623	JQ705425.1	JQ705425.1; H3c2; Asia_W_Europe; 16; T195C; A263G; C315CC	16569	Asia_W_Europe	H3c2	H	H	16
3624	JQ705426.1	JQ705426.1; H1e6; Asia_W_Europe; 15; T146C; A263G; C309CCT	16570	Asia_W_Europe	H1e6	H	H	15
3625	JQ705427.1	JQ705427.1; H1e1a; Asia_W_Europe; 14; T146C; A263G; C309CCCT	16571	Asia_W_Europe	H1e1a	H	H	14
3626	JQ705428.1	JQ705428.1; U4a3a; Asia_N_Europe_N; 38; A73G; T195C; G247A	16573	Asia_N_Europe_N	U4a3a	U	U4	38
3627	JQ705429.1	JQ705429.1; U5b2; Asia_W_Europe_C; 30; A73G; C150T; T195C	16570	Asia_W_Europe_C	U5b2	U	U5	30
3628	JQ705430.1	JQ705430.1; U5a1a1; Asia_W_Europe_C; 31; A73G; A263G; C315CC	16569	Asia_W_Europe_C	U5a1a1	U	U5	31
3629	JQ705431.1	JQ705431.1; H5b; Asia_W_Europe; 14; T152C; A263G; C309CCT	16570	Asia_W_Europe	H5b	H	H	14
3630	JQ705432.1	JQ705432.1; T2b5a1; Asia_W; 37; A73G; A263G; C315CC	16571	Asia_W	T2b5a1	T	T	37
3631	JQ705433.1	JQ705433.1; K1a26; Asia_W; 35; A73G; T195C; A263G	16571	Asia_W	K1a26	K	K	35
3632	JQ705434.1	JQ705434.1; A2j; Asia_NE_America_N; 33; C64T; A73G; T146C	16567	Asia_NE_America_N	A2j	A	A	33
3633	JQ705435.1	JQ705435.1; H1af; Asia_W_Europe; 11; A263G; C315CC; A374G	16569	Asia_W_Europe	H1af	H	H	11
3634	JQ705436.1	JQ705436.1; H1ae; Asia_W_Europe; 15; A263G; C309CCT; T310C	16570	Asia_W_Europe	H1ae	H	H	15
3635	JQ705437.1	JQ705437.1; H3ai; Asia_W_Europe; 11; A263G; C315CC; A750G	16569	Asia_W_Europe	H3ai	H	H	11
3636	JQ705438.1	JQ705438.1; H5a7; Asia_W_Europe; 17; T152C; A263G; C309CCT	16570	Asia_W_Europe	H5a7	H	H	17
3637	JQ705439.1	JQ705439.1; J1c4c; Asia_W; 31; A73G; G185A; G228A	16570	Asia_W	J1c4c	J	J	31
3638	JQ705440.1	JQ705440.1; H6a1a9; Asia_W_Europe; 18; T239C; A263G; C309CCT	16570	Asia_W_Europe	H6a1a9	H	H	18
3639	JQ705441.1	JQ705441.1; T1a1i; Asia_W; 38; A73G; T152C; T195C	16572	Asia_W	T1a1i	T	T	38
3640	JQ705442.1	JQ705442.1; J1c2; Asia_W; 29; A73G; G185A; A188G	16569	Asia_W	J1c2	J	J	29
3641	JQ705443.1	JQ705443.1; H1b(H1b2); Asia_W_Europe; 2; C315CC; G8251A;	16569	Asia_W_Europe	H1b(H1b2)	H	H	2
3642	JQ705444.1	JQ705444.1; T2b27; Asia_W; 35; A73G; A263G; C315CC	16569	Asia_W	T2b27	T	T	35
3643	JQ705445.1	JQ705445.1; K1a; Asia_W; 35; A73G; A263G; C315CC	16571	Asia_W	K1a	K	K	35
3644	JQ705446.1	JQ705446.1; T2b; Asia_W; 34; A73G; A263G; C315CC	16569	Asia_W	T2b	T	T	34
3645	JQ705447.1	JQ705447.1; J1c3b1; Asia_W; 31; A73G; G185A; A263G	16570	Asia_W	J1c3b1	J	J	31
3646	JQ705448.1	JQ705448.1; H1; Asia_W_Europe; 13; T146C; A263G; C315CC	16569	Asia_W_Europe	H1	H	H	13
3647	JQ705449.1	JQ705449.1; J1c8b; Asia_W; 28; A73G; G185A; G228A	16569	Asia_W	J1c8b	J	J	28
3648	JQ705450.1	JQ705450.1; H76; Asia_W_Europe; 11; A263G; C309CCT; T310C	16570	Asia_W_Europe	H76	H	H	11
3649	JQ705451.1	JQ705451.1; C1b11; Asia_NE_America_N_S; 44; A73G; C194T; A249d	16565	Asia_NE_America_N_S	C1b11	C	C	44

Supplementary Table S4 Human mitochondrial database (hMITO DB v1.0) metadata[a]

Row	Name (accession no.)	Description	Size	Geo_Region	Haplogroup	Macro_ Haplo	Macro_ Haplo2	Total Variants
3650	JQ705452.1	JQ705452.1; K2a; Asia_W; 32; A73G; T146C; T152C	16569	Asia_W	K2a	K	K	32
3651	JQ705453.1	JQ705453.1; H5e1a; Asia_W_Europe; 15; T152C; A263G; C315CC	16573	Asia_W_Europe	H5e1a	H	H	15
3652	JQ705454.1	JQ705454.1; K1a1c; Asia_W; 36; A73G; C114T; A263G	16569	Asia_W	K1a1c	K	K	36
3653	JQ705455.1	JQ705455.1; L2a1e1; Africa_W_C; 56; A73G; T146C; T152C	16569	Africa_W_C	L2a1e1	L2	L2	56
3654	JQ705456.1	JQ705456.1; K1a2a; Asia_W; 34; A73G; T195C; A263G	16570	Asia_W	K1a2a	K	K	34
3655	JQ705457.1	JQ705457.1; E1a(E1a2a4); Asia_SE_Oceania; 3; T152C; C309CCT; T310C	16570	Asia_SE_Oceania	E1a(E1a2a4)	E	E	3
3656	JQ705458.1	JQ705458.1; X2b4a1; Asia_W_America_N; 35; A73G; A153G; T195C	16570	Asia_W_America_N	X2b4a1	X	X	35
3657	JQ705459.1	JQ705459.1; H11a; Asia_W_Europe; 16; T195C; A263G; C309CCT	16570	Asia_W_Europe	H11a	H	H	16
3658	JQ705460.1	JQ705460.1; K1a27; Asia_W; 39; A73G; A263G; C309CCT	16576	Asia_W	K1a27	K	K	39
3659	JQ705461.1	JQ705461.1; M7b1a1+(16192); Asia_E_SE; 44; A73G; C150T; T199C	16570	Asia_E_SE	M7b1a1+(1619:	M	M7	44
3660	JQ705462.1	JQ705462.1; H1e1a; Asia_W_Europe; 15; T195C; A263G; C315CC	16568	Asia_W_Europe	H1e1a	H	H	15
3661	JQ705463.1	JQ705463.1; T1a1; Asia_W; 35; A73G; T152C; T195C	16569	Asia_W	T1a1	T	T	35
3662	JQ705464.1	JQ705464.1; HV0d; Asia_W; 18; T72C; T195C; A263G	16568	Asia_W	HV0d	HV	HV	18
3663	JQ705465.1	JQ705465.1; T2e1a; Asia_W; 36; C41T; A73G; C150T	16569	Asia_W	T2e1a	T	T	36
3664	JQ705466.1	JQ705466.1; J2b1a4; Asia_W; 35; A73G; C150T; T152C	16570	Asia_W	J2b1a4	J	J	35
3665	JQ705467.1	JQ705467.1; K1b2a; Asia_W; 35; A73G; T146C; T195C	16573	Asia_W	K1b2a	K	K	35
3666	JQ705468.1	JQ705468.1; H1c10; Asia_W_Europe; 13; A263G; C315CC; T477C	16569	Asia_W_Europe	H1c10	H	H	13
3667	JQ705469.1	JQ705469.1; K2b1a1; Asia_W; 39; A73G; T146C; A263G	16569	Asia_W	K2b1a1	K	K	39
3668	JQ705470.1	JQ705470.1; J1c1b; Asia_W; 32; A73G; G185A; G228A	16569	Asia_W	J1c1b	J	J	32
3669	JQ705471.1	JQ705471.1; H2a1c; Asia_W_Europe; 11; A263G; C309CCC; A750G	16570	Asia_W_Europe	H2a1c	H	H	11
3670	JQ705472.1	JQ705472.1; J1c3b1a; Asia_W; 33; A73G; G185A; A263G	16571	Asia_W	J1c3b1a	J	J	33
3671	JQ705473.1	JQ705473.1; U5b1c2b; Asia_W_Europe_C; 31; A73G; C150T; A263G	16569	Asia_W_Europe_C	U5b1c2b	U	U5	31
3672	JQ705474.1	JQ705474.1; X2b+226; Asia_W_America_N; 33; A73G; A153G; T195C	16571	Asia_W_America_N	X2b+226	X	X	33
3673	JQ705475.1	JQ705475.1; T2b; Asia_W; 35; A73G; A263G; C315CC	16569	Asia_W	T2b	T	T	35
3674	JQ705476.1	JQ705476.1; K1a; Asia_W; 33; A73G; T146C; A263G	16567	Asia_W	K1a	K	K	33
3675	JQ705477.1	JQ705477.1; V; Europe_S; 16; T72C; A263G; C309CCT	16569	Europe_S	V	V	V	16
3676	JQ705478.1	JQ705478.1; L1b1a6; Africa_C; 81; A73G; T152C; C182T	16567	Africa_C	L1b1a6	L1	L1	81
3677	JQ705479.1	JQ705479.1; U5a1a1d; Asia_W_Europe_C; 32; A73G; G185A; T204C	16572	Asia_W_Europe_C	U5a1a1d	U	U5	32
3678	JQ705480.1	JQ705480.1; X2c1d; Asia_W_America_N; 30; A73G; A153G; T195C	16569	Asia_W_America_N	X2c1d	X	X	30
3679	JQ705481.1	JQ705481.1; K1c1; Asia_W; 33; A73G; T146C; A263G	16568	Asia_W	K1c1	K	K	33
3680	JQ705482.1	JQ705482.1; H1b1c; Asia_W_Europe; 17; A263G; C315CC; C483T	16567	Asia_W_Europe	H1b1c	H	H	17
3681	JQ705483.1	JQ705483.1; K1a1a; Asia_W; 32; A73G; C114T; A263G	16569	Asia_W	K1a1a	K	K	32
3682	JQ705484.1	JQ705484.1; T2b33; Asia_W; 41; A73G; C198T; A263G	16570	Asia_W	T2b33	T	T	41
3683	JQ705485.1	JQ705485.1; K1a3a; Asia_W; 33; A73G; A263G; C315CC	16569	Asia_W	K1a3a	K	K	33
3684	JQ705486.1	JQ705486.1; H24a2; Asia_W_Europe; 11; A263G; C315CC; G513A	16569	Asia_W_Europe	H24a2	H	H	11
3685	JQ705487.1	JQ705487.1; W3a2; Asia_W; 40; A73G; T152C; A189G	16570	Asia_W	W3a2	W	W	40
3686	JQ705488.1	JQ705488.1; J1c5a1; Asia_W; 33; A73G; G185A; G228A	16569	Asia_W	J1c5a1	J	J	33
3687	JQ705489.1	JQ705489.1; J1c+16261; Asia_W; 29; A73G; G185A; A263G	16570	Asia_W	J1c+16261	J	J	29
3688	JQ705490.1	JQ705490.1; H18; Asia_W_Europe; 14; C150T; A263G; C309CCCT	16571	Asia_W_Europe	H18	H	H	14
3689	JQ705491.1	JQ705491.1; A2+(64); Asia_NE_America_N; 37; C64T; A73G; G143A	16569	Asia_NE_America_N	A2+(64)	A	A	37
3690	JQ705492.1	JQ705492.1; T2b13b; Asia_W; 39; A73G; A263G; C309CC	16570	Asia_W	T2b13b	T	T	39

Supplementary Table S4 Human mitochondrial database (hMITO DB v1.0) metadata[a]

Row	Name (accession no.)	Description	Size	Geo_Region	Haplogroup	Macro_ Haplo	Macro_ Haplo2	Total Variants
3691	JQ705493.1	JQ705493.1; H1m; Asia_W_Europe; 13; C150T; A263G; C315CC	16569	Asia_W_Europe	H1m	H	H	13
3692	JQ705494.1	JQ705494.1; T2b6a; Asia_W; 39; A73G; A263G; C309CCT	16570	Asia_W	T2b6a	T	T	39
3693	JQ705495.1	JQ705495.1; H1c11; Asia_W_Europe; 11; A263G; C315CC; T477C	16569	Asia_W_Europe	H1c11	H	H	11
3694	JQ705496.1	JQ705496.1; H1e1a2; Asia_W_Europe; 17; A263G; C309CCT; T310C	16570	Asia_W_Europe	H1e1a2	H	H	17
3695	JQ705497.1	JQ705497.1; T1a1; Asia_W; 39; A73G; T152C; T195C	16570	Asia_W	T1a1	T	T	39
3696	JQ705498.1	JQ705498.1; U5a1d2a1; Asia_W_Europe_C; 36; A73G; T195C; A263G	16572	Asia_W_Europe_C	U5a1d2a1	U	U5	36
3697	JQ705499.1	JQ705499.1; T2a1a5; Asia_W; 35; A73G; A263G; C315CC	16569	Asia_W	T2a1a5	T	T	35
3698	JQ705500.1	JQ705500.1; U5b2c2; Asia_W_Europe_C; 33; A73G; C150T; T152C	16571	Asia_W_Europe_C	U5b2c2	U	U5	33
3699	JQ705501.1	JQ705501.1; A2v1b; Asia_NE_America_N; 36; C64T; A73G; T146C	16569	Asia_NE_America_N	A2v1b	A	A	36
3700	JQ705502.1	JQ705502.1; U5b2a1b; Asia_W_Europe_C; 30; A73G; C150T; T152C	16569	Asia_W_Europe_C	U5b2a1b	U	U5	30
3701	JQ705503.1	JQ705503.1; M7a1a; Asia_E_SE; 41; A73G; A263G; C309CCT	16569	Asia_E_SE	M7a1a	M	M7	41
3702	JQ705504.1	JQ705504.1; H3+152; Asia_W_Europe; 12; T152C; A263G; C309CCCT	16571	Asia_W_Europe	H3+152	H	H	12
3703	JQ705505.1	JQ705505.1; H3v; Asia_W_Europe; 11; A263G; C309CCCT; T310C	16571	Asia_W_Europe	H3v	H	H	11
3704	JQ705506.1	JQ705506.1; V12; Europe_S; 16; T72C; A263G; C309CCT	16570	Europe_S	V12	V	V	16
3705	JQ705507.1	JQ705507.1; H5a1; Asia_W_Europe; 14; A263G; C309CCCT; T310C	16569	Asia_W_Europe	H5a1	H	H	14
3706	JQ705508.1	JQ705508.1; H13a1a; Asia_W_Europe; 14; A263G; C309CCCT; T310C	16571	Asia_W_Europe	H13a1a	H	H	14
3707	JQ705509.1	JQ705509.1; X2b5; Asia_W_America_N; 33; A73G; A153G; T195C	16569	Asia_W_America_N	X2b5	X	X	33
3708	JQ705510.1	JQ705510.1; H2a2a1; Asia_W_Europe; 5; T152C; A200G; C309CCT	16570	Asia_W_Europe	H2a2a1	H	H	5
3709	JQ705511.1	JQ705511.1; H5b1; Asia_W_Europe; 15; T146C; T195C; A263G	16570	Asia_W_Europe	H5b1	H	H	15
3710	JQ705512.1	JQ705512.1; J2a1a1c; Asia_W; 41; A73G; C150T; T152C	16570	Asia_W	J2a1a1c	J	J	41
3711	JQ705513.1	JQ705513.1; V2a1a; Europe_S; 21; T72C; A263G; C309CCT	16570	Europe_S	V2a1a	V	V	21
3712	JQ705514.1	JQ705514.1; I4a; Asia_W_SW; 34; A73G; T199C; T204C	16572	Asia_W_SW	I4a	I	I	34
3713	JQ705515.1	JQ705515.1; U1a1b; Asia_W; 38; A73G; T152C; A263G	16571	Asia_W	U1a1b	U	U1	38
3714	JQ705516.1	JQ705516.1; H1c4; Asia_W_Europe; 11; A263G; C315CC; T477C	16569	Asia_W_Europe	H1c4	H	H	11
3715	JQ705517.1	JQ705517.1; H49a1; Asia_W_Europe; 13; A263G; C309CCT; T310C	16572	Asia_W_Europe	H49a1	H	H	13
3716	JQ705518.1	JQ705518.1; HV+16311; Asia_W_Europe; 14; A263G; C309CCCT; T310C	16571	Asia_W_Europe	HV+16311	H	H	14
3717	JQ705519.1	JQ705519.1; K1b2a1a1; Asia_W; 37; A73G; T146C; T195C	16573	Asia_W	K1b2a1a1	K	K	37
3718	JQ705520.1	JQ705520.1; K1c1; Asia_W; 32; A73G; T146C; T152C	16568	Asia_W	K1c1	K	K	32
3719	JQ705521.1	JQ705521.1; L3e1f2; Africa_E; 37; A73G; C150T; A189G	16560	Africa_E	L3e1f2	L3	L3	37
3720	JQ705522.1	JQ705522.1; N1a1a1a2; Africa_NE_Asia_W; 38; A73G; T152C; T199C	16571	Africa_NE_Asia_W	N1a1a1a2	N	N1	38
3721	JQ705523.1	JQ705523.1; K1a11; Asia_W; 35; A16T; A73G; C150T	16560	Asia_W	K1a11	K	K	35
3722	JQ705524.1	JQ705524.1; H13a2b3; Asia_W_Europe; 16; A73G; G143A; A263G	16571	Asia_W_Europe	H13a2b3	H	H	16
3723	JQ705525.1	JQ705525.1; X4; Asia_W_America_N; 33; A73G; T146C; T152C	16570	Asia_W_America_N	X4	X	X	33
3724	JQ705526.1	JQ705526.1; HV1c; Asia_W; 19; T65C; G207A; A263G	16570	Asia_W	HV1c	HV	HV	19
3725	JQ705527.1	JQ705527.1; N1a1a1a2; Africa_NE_Asia_W; 41; A73G; T152C; T199C	16571	Africa_NE_Asia_W	N1a1a1a2	N	N1	41
3726	JQ705528.1	JQ705528.1; T2f4; Asia_W; 36; A73G; A263G; C309CCCT	16562	Asia_W	T2f4	T	T	36
3727	JQ705529.1	JQ705529.1; L2a1a3a; Africa_W_C; 60; A73G; G143A; T146C	16572	Africa_W_C	L2a1a3a	L2	L2	60
3728	JQ705530.1	JQ705530.1; U5a2a2; Asia_W_Europe_C; 27; A73G; C151T; A263G	16569	Asia_W_Europe_C	U5a2a2	U	U5	27
3729	JQ705531.1	JQ705531.1; H7; Asia_W_Europe; 13; A263G; C309CCT; T310C	16570	Asia_W_Europe	H7	H	H	13
3730	JQ705532.1	JQ705532.1; H2a2b; Asia_W_Europe; 10; A263G; C309CCT; T310C	16570	Asia_W_Europe	H2a2b	H	H	10
3731	JQ705533.1	JQ705533.1; H1a3b1; Asia_W_Europe; 17; A73G; A263G; C315CC	16567	Asia_W_Europe	H1a3b1	H	H	17

Supplementary Table S4 Human mitochondrial database (hMITO DB v1.0) metadata[a]

Row	Name (accession no.)	Description	Size	Geo_Region	Haplogroup	Macro_ Haplo	Macro_ Haplo2	Total Variants
3732	JQ705534.1	JQ705534.1; A2h1; Asia_NE_America_N; 34; C64T; A73G; T146C	16566	Asia_NE_America_N	A2h1	A	A	34
3733	JQ705535.1	JQ705535.1; J2a1a1a2; Asia_W; 40; A73G; C150T; T152C	16570	Asia_W	J2a1a1a2	J	J	40
3734	JQ705536.1	JQ705536.1; W5a1a; Asia_W; 41; A73G; G143A; A189G	16570	Asia_W	W5a1a	W	W	41
3735	JQ705537.1	JQ705537.1; K1a13a; Asia_W; 36; A73G; T146C; T195C	16572	Asia_W	K1a13a	K	K	36
3736	JQ705538.1	JQ705538.1; K1e; Asia_W; 39; A73G; T152C; A263G	16572	Asia_W	K1e	K	K	39
3737	JQ705539.1	JQ705539.1; H15a1; Asia_W_Europe; 13; T55C; T57C; A263G	16570	Asia_W_Europe	H15a1	H	H	13
3738	JQ705540.1	JQ705540.1; HV1b2; Asia_W; 22; T152C; A263G; C309CCCT	16571	Asia_W	HV1b2	HV	HV	22
3739	JQ705541.1	JQ705541.1; B2; Asia_SE_E_America_N_S; 38; T63C; C64T; A73G	16570	Asia_SE_E_America_N_S	B2	B	B	38
3740	JQ705542.1	JQ705542.1; T1a1i; Asia_W; 40; A73G; T152C; A183G	16572	Asia_W	T1a1i	T	T	40
3741	JQ705543.1	JQ705543.1; K1a4a1; Asia_W; 38; A73G; A189G; A263G	16572	Asia_W	K1a4a1	K	K	38
3742	JQ705544.1	JQ705544.1; HV0; Asia_W; 21; T72C; C150T; C186T	16570	Asia_W	HV0	HV	HV	21
3743	JQ705545.1	JQ705545.1; H3g1b; Asia_W_Europe; 15; T152C; A263G; C315CC	16569	Asia_W_Europe	H3g1b	H	H	15
3744	JQ705546.1	JQ705546.1; H33; Asia_W_Europe; 11; A263G; C309CCT; T310C	16570	Asia_W_Europe	H33	H	H	11
3745	JQ705547.1	JQ705547.1; U4a1a; Asia_N_Europe_N; 33; A73G; T152C; T195C	16569	Asia_N_Europe_N	U4a1a	U	U4	33
3746	JQ705548.1	JQ705548.1; H3n; Asia_W_Europe; 14; A263G; C315CC; A750G	16569	Asia_W_Europe	H3n	H	H	14
3747	JQ705549.1	JQ705549.1; H1ba; Asia_W_Europe; 12; A263G; C309CCT; T310C	16570	Asia_W_Europe	H1ba	H	H	12
3748	JQ705550.1	JQ705550.1; X2b12; Asia_W_America_N; 34; A73G; A153G; T195C	16570	Asia_W_America_N	X2b12	X	X	34
3749	JQ705551.1	JQ705551.1; H6a1b2; Asia_W_Europe; 20; G207A; T239C; A263G	16570	Asia_W_Europe	H6a1b2	H	H	20
3750	JQ705552.1	JQ705552.1; N1b1a2a; Africa_NE_Asia_W; 41; A73G; T152C; A200G	16572	Africa_NE_Asia_W	N1b1a2a	N	N1	41
3751	JQ705553.1	JQ705553.1; U4a1a; Asia_N_Europe_N; 33; A73G; T152C; T195C	16571	Asia_N_Europe_N	U4a1a	U	U4	33
3752	JQ705554.1	JQ705554.1; H41a; Asia_W_Europe; 16; C262T; A263G; C309CCT	16570	Asia_W_Europe	H41a	H	H	16
3753	JQ705555.1	JQ705555.1; H1e1a; Asia_W_Europe; 15; A263G; C309CCT; T310C	16570	Asia_W_Europe	H1e1a	H	H	15
3754	JQ705556.1	JQ705556.1; K1a4a1a2a; Asia_W; 40; A73G; A263G; C309CCT	16576	Asia_W	K1a4a1a2a	K	K	40
3755	JQ705557.1	JQ705557.1; A2q1; Asia_NE_America_N; 42; C64T; A73G; G94A	16568	Asia_NE_America_N	A2q1	A	A	42
3756	JQ705558.1	JQ705558.1; K1a4a1d; Asia_W; 36; A73G; A263G; C309CCT	16570	Asia_W	K1a4a1d	K	K	36
3757	JQ705559.1	JQ705559.1; U2e1a1; Asia_S_W_Europe; 42; A73G; T152C; T217C	16572	Asia_S_W_Europe	U2e1a1	U	U2	42
3758	JQ705560.1	JQ705560.1; U4b1b1; Asia_N_Europe_N; 35; A73G; T146C; T152C	16575	Asia_N_Europe_N	U4b1b1	U	U4	35
3759	JQ705561.1	JQ705561.1; R1a1a; Asia_S_SE; 33; A73G; A263G; C295A	16570	Asia_S_SE	R1a1a	R	R1	33
3760	JQ705562.1	JQ705562.1; J1c1b; Asia_W; 36; A73G; G185A; G228A	16570	Asia_W	J1c1b	J	J	36
3761	JQ705563.1	JQ705563.1; H2a2b1; Asia_W_Europe; 9; A263G; C309CCCT; T310C	16571	Asia_W_Europe	H2a2b1	H	H	9
3762	JQ705564.1	JQ705564.1; H10e; Asia_W_Europe; 12; A263G; C309CCT; T310C	16570	Asia_W_Europe	H10e	H	H	12
3763	JQ705565.1	JQ705565.1; HV0; Asia_W; 16; T72C; A263G; C309CCT	16570	Asia_W	HV0	HV	HV	16
3764	JQ705566.1	JQ705566.1; U4a2a2; Asia_N_Europe_N; 28; A73G; T195C; A263G	16570	Asia_N_Europe_N	U4a2a2	U	U4	28
3765	JQ705567.1	JQ705567.1; HV0a1; Asia_W; 16; A263G; C309CCCT; T310C	16569	Asia_W	HV0a1	HV	HV	16
3766	JQ705568.1	JQ705568.1; K1a1b1a; Asia_W; 36; A73G; C114T; A263G	16569	Asia_W	K1a1b1a	K	K	36
3767	JQ705569.1	JQ705569.1; H5c1a; Asia_W_Europe; 12; A263G; C315CC; C456T	16569	Asia_W_Europe	H5c1a	H	H	12
3768	JQ705570.1	JQ705570.1; C1b5b; Asia_NE_America_N_S; 47; A73G; A249d; G255A	16564	Asia_NE_America_N_S	C1b5b	C	C	47
3769	JQ705571.1	JQ705571.1; T2c1e; Asia_W; 44; A73G; T146C; C150T	16568	Asia_W	T2c1e	T	T	44
3770	JQ705572.1	JQ705572.1; F1f; Asia_SE_E; 32; A73G; A249d; A263G	16566	Asia_SE_E	F1f	F	F	32
3771	JQ705573.1	JQ705573.1; U5a1c1a; Asia_W_Europe_C; 29; A73G; A153G; T195C	16569	Asia_W_Europe_C	U5a1c1a	U	U5	29
3772	JQ705574.1	JQ705574.1; C1c6; Asia_NE_America_N_S; 46; A73G; T195C; A249d	16567	Asia_NE_America_N_S	C1c6	C	C	46

Supplementary Table S4 Human mitochondrial database (hMITO DB v1.0) metadata[a]

Row	Name (accession no.)	Description	Size	Geo_Region	Haplogroup	Macro_ Haplo	Macro_ Haplo2	Total Variants
3773	JQ705575.1	JQ705575.1; J2a1a1; Asia_W; 38; A73G; C150T; T195C	16570	Asia_W	J2a1a1	J	J	38
3774	JQ705576.1	JQ705576.1; L1c2b1c; Africa_C; 95; A73G; T152C; C182T	16568	Africa_C	L1c2b1c	L1	L1	95
3775	JQ705577.1	JQ705577.1; U5b2b3; Asia_W_Europe_C; 37; A73G; T146C; C150T	16570	Asia_W_Europe_C	U5b2b3	U	U5	37
3776	JQ705578.1	JQ705578.1; H2a2a1; Asia_N_Europe_N; 3; C309CCCT; T310C; A14375G	16571	Asia_N_Europe_N	H2a2a1	U	U4	3
3777	JQ705579.1	JQ705579.1; H1bm; Asia_W_Europe; 11; A263G; C309CCT; T310C	16570	Asia_W_Europe	H1bm	H	H	11
3778	JQ705580.1	JQ705580.1; H11a2a; Asia_W_Europe; 16; T195C; A263G; C315CC	16569	Asia_W_Europe	H11a2a	H	H	16
3779	JQ705581.1	JQ705581.1; J1c5; Asia_W; 32; A73G; G185A; G228A	16573	Asia_W	J1c5	J	J	32
3780	JQ705582.1	JQ705582.1; H5a2a; Asia_W_Europe; 14; A263G; C309CCCT; T310C	16571	Asia_W_Europe	H5a2a	H	H	14
3781	JQ705583.1	JQ705583.1; H60a; Asia_W_Europe; 13; G185A; A263G; C315CC	16569	Asia_W_Europe	H60a	H	H	13
3782	JQ705584.1	JQ705584.1; H1at1a; Asia_W_Europe; 14; A263G; C315CC; A750G	16569	Asia_W_Europe	H1at1a	H	H	14
3783	JQ705585.1	JQ705585.1; C1b4; Asia_NE_America_N_S; 47; A73G; G143A; T152C	16565	Asia_NE_America_N_S	C1b4	C	C	47
3784	JQ705586.1	JQ705586.1; H1e1a; Asia_W_Europe; 13; A263G; C309CCT; T310C	16570	Asia_W_Europe	H1e1a	H	H	13
3785	JQ705587.1	JQ705587.1; L1b1a15a; Africa_C; 85; A73G; T146C; T152C	16567	Africa_C	L1b1a15a	L1	L1	85
3786	JQ705588.1	JQ705588.1; J1b1a1d; Asia_W; 40; A73G; G185A; C242T	16569	Asia_W	J1b1a1d	J	J	40
3787	JQ705589.1	JQ705589.1; L2a1l2a1; Africa_W_C; 56; A73G; T146C; T152C	16570	Africa_W_C	L2a1l2a1	L2	L2	56
3788	JQ705590.1	JQ705590.1; N1b1b1; Africa_NE_Asia_W; 40; A73G; T152C; A263G	16570	Africa_NE_Asia_W	N1b1b1	N	N1	40
3789	JQ705591.1	JQ705591.1; U4a1a; Asia_N_Europe_N; 33; A73G; T152C; T195C	16572	Asia_N_Europe_N	U4a1a	U	U4	33
3790	JQ705592.1	JQ705592.1; H6a2; Asia_W_Europe; 14; T239C; A263G; C315CC	16569	Asia_W_Europe	H6a2	H	H	14
3791	JQ705593.1	JQ705593.1; J1c2h; Asia_W; 31; A73G; G185A; A188G	16570	Asia_W	J1c2h	J	J	31
3792	JQ705594.1	JQ705594.1; H7; Asia_W_Europe; 10; A263G; C315CC; A750G	16569	Asia_W_Europe	H7	H	H	10
3793	JQ705595.1	JQ705595.1; I1a1b; Asia_W_SW; 42; A73G; T199C; G203A	16572	Asia_W_SW	I1a1b	I	I	42
3794	JQ705596.1	JQ705596.1; L3e5b; Africa_E; 30; A73G; C150T; C315CC	16567	Africa_E	L3e5b	L3	L3	30
3795	JQ705597.1	JQ705597.1; L1c2b1c; Africa_C; 97; A73G; T152C; C182T	16568	Africa_C	L1c2b1c	L1	L1	97
3796	JQ705598.1	JQ705598.1; B2a1b; Asia_SE_E_America_N_S; 34; A73G; A263G; C315CC	16560	Asia_SE_E_America_N_S	B2a1b	B	B	34
3797	JQ705599.1	JQ705599.1; HV0; Asia_W; 14; T72C; A263G; C309CCCT	16571	Asia_W	HV0	HV	HV	14
3798	JQ705600.1	JQ705600.1; T2b4a; Asia_W; 37; A73G; A263G; C309CCT	16570	Asia_W	T2b4a	T	T	37
3799	JQ705601.1	JQ705601.1; U1a1a3; Asia_W; 42; A73G; T152C; T195C	16570	Asia_W	U1a1a3	U	U1	42
3800	JQ705602.1	JQ705602.1; U2e1a1; Asia_S_W_Europe; 44; A73G; T152C; T217C	16572	Asia_S_W_Europe	U2e1a1	U	U2	44
3801	JQ705603.1	JQ705603.1; H6a1b; Asia_W_Europe; 16; T152C; T239C; A263G	16571	Asia_W_Europe	H6a1b	H	H	16
3802	JQ705604.1	JQ705604.1; V8; Europe_S; 17; T72C; A263G; C309CCT	16570	Europe_S	V8	V	V	17
3803	JQ705605.1	JQ705605.1; W3b; Asia_W; 40; A73G; A189G; C194T	16570	Asia_W	W3b	W	W	40
3804	JQ705606.1	JQ705606.1; L1b1a18; Africa_C; 81; A73G; T152C; G185T	16568	Africa_C	L1b1a18	L1	L1	81
3805	JQ705607.1	JQ705607.1; H1f1a; Asia_W_Europe; 16; A263G; C315CC; C459d(=C456d†)	16568	Asia_W_Europe	H1f1a	H	H	16
3806	JQ705608.1	JQ705608.1; U5b1e1; Asia_W_Europe_C; 32; A73G; C150T; T152C	16567	Asia_W_Europe_C	U5b1e1	U	U5	32
3807	JQ705609.1	JQ705609.1; U4a2; Asia_N_Europe_N; 32; A73G; T195C; A263G	16572	Asia_N_Europe_N	U4a2	U	U4	32
3808	JQ705610.1	JQ705610.1; V+@72; Europe_S; 15; A263G; C309CCCT; T310C	16571	Europe_S	V+@72	V	V	15
3809	JQ705611.1	JQ705611.1; K2a; Asia_W; 34; A73G; T146C; T152C	16569	Asia_W	K2a	K	K	34
3810	JQ705612.1	JQ705612.1; X2c1c; Asia_W_America_N; 32; A73G; A153G; T195C	16570	Asia_W_America_N	X2c1c	X	X	32
3811	JQ705613.1	JQ705613.1; U5b1b1a; Asia_W_Europe_C; 31; A73G; C150T; A263G	16570	Asia_W_Europe_C	U5b1b1a	U	U5	31
3812	JQ705614.1	JQ705614.1; V; Europe_S; 17; T72C; A263G; C309CCT	16570	Europe_S	V	V	V	17
3813	JQ705615.1	JQ705615.1; H1g1; Asia_W_Europe; 14; A263G; C309CCCT; T310C	16571	Asia_W_Europe	H1g1	H	H	14

Supplementary Table S4 Human mitochondrial database (hMITO DB v1.0) metadata[a]

Row	Name (accession no.)	Description	Size	Geo_Region	Haplogroup	Macro_ Haplo	Macro_ Haplo2	Total Variants
3814	JQ705616.1	JQ705616.1; H7e; Asia_W_Europe; 13; A263G; C309CCCT; T310C	16571	Asia_W_Europe	H7e	H	H	13
3815	JQ705617.1	JQ705617.1; U5a1a1; Asia_W_Europe_C; 28; A73G; A263G; C315CC	16569	Asia_W_Europe_C	U5a1a1	U	U5	28
3816	JQ705618.1	JQ705618.1; H1h1; Asia_W_Europe; 16; T152C; A263G; C309CCT	16570	Asia_W_Europe	H1h1	H	H	16
3817	JQ705619.1	JQ705619.1; M7c1c3; Asia_E_SE; 39; A73G; T146C; T199C	16567	Asia_E_SE	M7c1c3	M	M7	39
3818	JQ705620.1	JQ705620.1; K1a2a; Asia_W; 34; A73G; A263G; C309CCT	16570	Asia_W	K1a2a	K	K	34
3819	JQ705621.1	JQ705621.1; U5a1a1e; Asia_W_Europe_C; 26; A73G; A263G; C315CC	16569	Asia_W_Europe_C	U5a1a1e	U	U5	26
3820	JQ705622.1	JQ705622.1; H1c3; Asia_W_Europe; 16; T195C; A257G; A263G	16569	Asia_W_Europe	H1c3	H	H	16
3821	JQ705623.1	JQ705623.1; J2b1; Asia_W; 35; A73G; C150T; T152C	16571	Asia_W	J2b1	J	J	35
3822	JQ705624.1	JQ705624.1; H59a; Asia_W_Europe; 12; A263G; C315CC; A750G	16569	Asia_W_Europe	H59a	H	H	12
3823	JQ705625.1	JQ705625.1; J2a1a1a2; Asia_W; 43; A73G; C150T; T152C	16570	Asia_W	J2a1a1a2	J	J	43
3824	JQ705626.1	JQ705626.1; L2c; Africa_W_C; 60; A73G; A93G; T146C	16568	Africa_W_C	L2c	L2	L2	60
3825	JQ705627.1	JQ705627.1; U5a2c4; Asia_W_Europe_C; 26; A73G; T146C; A263G	16569	Asia_W_Europe_C	U5a2c4	U	U5	26
3826	JQ705628.1	JQ705628.1; K1a1b1a; Asia_W; 38; A73G; C114T; A189G	16569	Asia_W	K1a1b1a	K	K	38
3827	JQ705629.1	JQ705629.1; K1a4d; Asia_W; 36; A73G; T195C; A263G	16571	Asia_W	K1a4d	K	K	36
3828	JQ705630.1	JQ705630.1; T1a1c; Asia_W; 38; A73G; T152C; T195C	16570	Asia_W	T1a1c	T	T	38
3829	JQ705631.1	JQ705631.1; J2a1a1a2; Asia_W; 41; A73G; C150T; T152C	16570	Asia_W	J2a1a1a2	J	J	41
3830	JQ705632.1	JQ705632.1; J2a1a1a2; Asia_W; 41; A73G; C150T; T152C	16570	Asia_W	J2a1a1a2	J	J	41
3831	JQ705633.1	JQ705633.1; J1c4c; Asia_W; 31; A73G; G185A; G228A	16569	Asia_W	J1c4c	J	J	31
3832	JQ705634.1	JQ705634.1; H82; Asia_W_Europe; 12; T195C; A263G; C315CC	16567	Asia_W_Europe	H82	H	H	12
3833	JQ705635.1	JQ705635.1; J1c3f; Asia_W; 32; A73G; G228A; A263G	16570	Asia_W	J1c3f	J	J	32
3834	JQ705636.1	JQ705636.1; J1c3b; Asia_W; 29; A73G; G185A; A263G	16571	Asia_W	J1c3b	J	J	29
3835	JQ705637.1	JQ705637.1; F1b1a1a2; Asia_SE_E; 45; A73G; T152C; A249d	16568	Asia_SE_E	F1b1a1a2	F	F	45
3836	JQ705638.1	JQ705638.1; V3c; Europe_S; 19; T72C; A263G; C309CCCT	16571	Europe_S	V3c	V	V	19
3837	JQ705639.1	JQ705639.1; T2f3; Asia_W; 34; A73G; A263G; C309CCCT	16562	Asia_W	T2f3	T	T	34
3838	JQ705640.1	JQ705640.1; H16b; Asia_W_Europe; 12; A263G; C315CC; A750G	16569	Asia_W_Europe	H16b	H	H	12
3839	JQ705641.1	JQ705641.1; T2a1b1a; Asia_W; 40; A73G; T131C; A263G	16570	Asia_W	T2a1b1a	T	T	40
3840	JQ705642.1	JQ705642.1; W3a1a2; Asia_W; 39; A73G; A189G; C194T	16569	Asia_W	W3a1a2	W	W	39
3841	JQ705643.1	JQ705643.1; H11a8; Asia_W_Europe; 15; T195C; A263G; C309CCT	16570	Asia_W_Europe	H11a8	H	H	15
3842	JQ705644.1	JQ705644.1; T2f1a1; Asia_W; 41; A73G; T195C; A263G	16560	Asia_W	T2f1a1	T	T	41
3843	JQ705645.1	JQ705645.1; I1a1; Asia_W_SW; 43; A73G; T199C; G203A	16573	Asia_W_SW	I1a1	I	I	43
3844	JQ705646.1	JQ705646.1; K1a2a; Asia_W; 35; A73G; A263G; C309CCT	16572	Asia_W	K1a2a	K	K	35
3845	JQ705647.1	JQ705647.1; T2b21; Asia_W; 34; A73G; T195C; A263G	16569	Asia_W	T2b21	T	T	34
3846	JQ705648.1	JQ705648.1; B2t; Asia_SE_E_America_N_S; 37; A73G; A263G; C309CCT	16561	Asia_SE_E_America_N_S	B2t	B	B	37
3847	JQ705649.1	JQ705649.1; H1; Asia_W_Europe; 14; A263G; C309CCT; T310C	16570	Asia_W_Europe	H1	H	H	14
3848	JQ705650.1	JQ705650.1; L1c2b1b1; Africa_C; 96; A73G; C151T; T152C	16569	Africa_C	L1c2b1b1	L1	L1	96
3849	JQ705651.1	JQ705651.1; U2e2a1c; Asia_S_W_Europe; 41; A73G; T152C; T217C	16571	Asia_S_W_Europe	U2e2a1c	U	U2	41
3850	JQ705652.1	JQ705652.1; H4a1a4b; Asia_W_Europe; 20; T195C; A263G; C315CC	16567	Asia_W_Europe	H4a1a4b	H	H	20
3851	JQ705653.1	JQ705653.1; U8a1a1; Asia_W_Europe ; 32; A73G; A263G; T282C	16570	Asia_W_Europe	U8a1a1	U	U8	32
3852	JQ705654.1	JQ705654.1; H1a1; Asia_W_Europe; 14; A73G; A263G; C309CCT	16570	Asia_W_Europe	H1a1	H	H	14
3853	JQ705655.1	JQ705655.1; U4a2; Asia_N_Europe_N; 30; A73G; T195C; A263G	16572	Asia_N_Europe_N	U4a2	U	U4	30
3854	JQ705656.1	JQ705656.1; K1a4g; Asia_W; 38; A73G; A263G; C264T	16570	Asia_W	K1a4g	K	K	38

Supplementary Table S4 Human mitochondrial database (hMITO DB v1.0) metadata[a]

Row	Name (accession no.)	Description	Size	Geo_Region	Haplogroup	Macro_ Haplo	Macro_ Haplo2	Total Variants
3855	JQ705657.1	JQ705657.1; H1+16239; Asia_W_Europe; 14; A263G; C309CCT; T310C	16570	Asia_W_Europe	H1+16239	H	H	14
3856	JQ705658.1	JQ705658.1; V2; Europe_S; 17; T72C; A263G; C309CCT	16570	Europe_S	V2	V	V	17
3857	JQ705659.1	JQ705659.1; H6a1b2e; Asia_W_Europe; 19; T239C; A263G; C309CCCT	16571	Asia_W_Europe	H6a1b2e	H	H	19
3858	JQ705660.1	JQ705660.1; H6a1a8a; Asia_W_Europe; 19; T239C; A263G; C309CCT	16570	Asia_W_Europe	H6a1a8a	H	H	19
3859	JQ705661.1	JQ705661.1; K1a4a1; Asia_W; 35; A73G; A263G; C309CCT	16572	Asia_W	K1a4a1	K	K	35
3860	JQ705662.1	JQ705662.1; M18b; Africa_E; 34; A73G; T199C; T246C	16569	Africa_E	M18b	M	M18	34
3861	JQ705663.1	JQ705663.1; T1a1; Asia_W; 37; A73G; T152C; T195C	16570	Asia_W	T1a1	T	T	37
3862	JQ705664.1	JQ705664.1; T2b19; Asia_W; 35; A73G; A263G; C315CC	16567	Asia_W	T2b19	T	T	35
3863	JQ705665.1	JQ705665.1; C1b; Asia_NE_America_N_S; 43; A73G; A249d; A263G	16563	Asia_NE_America_N_S	C1b	C	C	43
3864	JQ705666.1	JQ705666.1; I2c; Asia_W_SW; 37; A73G; T152C; T199C	16571	Asia_W_SW	I2c	I	I	37
3865	JQ705667.1	JQ705667.1; U2e2a1a2; Asia_S_W_Europe; 42; A73G; T152C; A263G	16571	Asia_S_W_Europe	U2e2a1a2	U	U2	42
3866	JQ705668.1	JQ705668.1; T2c1d1a; Asia_W; 40; A73G; T146C; A263G	16570	Asia_W	T2c1d1a	T	T	40
3867	JQ705669.1	JQ705669.1; L1b1a3a; Africa_C; 83; A73G; T152C; C182T	16568	Africa_C	L1b1a3a	L1	L1	83
3868	JQ705670.1	JQ705670.1; H10e; Asia_W_Europe; 12; A263G; C309CCT; T310C	16570	Asia_W_Europe	H10e	H	H	12
3869	JQ705671.1	JQ705671.1; X2b+226; Asia_W_America_N; 33; A73G; A153G; T195C	16571	Asia_W_America_N	X2b+226	X	X	33
3870	JQ705672.1	JQ705672.1; U2e2a1a; Asia_S_W_Europe; 41; A73G; T152C; T217C	16570	Asia_S_W_Europe	U2e2a1a	U	U2	41
3871	JQ705673.1	JQ705673.1; T2e; Asia_W; 37; A73G; C150T; A263G	16571	Asia_W	T2e	T	T	37
3872	JQ705674.1	JQ705674.1; H27a; Asia_W_Europe; 14; A263G; C315CC; A750G	16569	Asia_W_Europe	H27a	H	H	14
3873	JQ705675.1	JQ705675.1; U5b1b1+@16192; Asia_W_Europe_C; 31; A73G; C150T; A263G	16571	Asia_W_Europe_C	U5b1b1+@161[illegible]	U	U5	31
3874	JQ705676.1	JQ705676.1; C1c1b; Asia_NE_America_N_S; 43; A73G; A215G; A249d	16566	Asia_NE_America_N_S	C1c1b	C	C	43
3875	JQ705677.1	JQ705677.1; J2b1a2; Asia_W; 36; A73G; C150T; T152C	16570	Asia_W	J2b1a2	J	J	36
3876	JQ705678.1	JQ705678.1; H1cc; Asia_W_Europe; 14; T72C; A263G; C309CCCT	16571	Asia_W_Europe	H1cc	H	H	14
3877	JQ705679.1	JQ705679.1; H7a1c; Asia_W_Europe; 14; A263G; C315CC; A750G	16569	Asia_W_Europe	H7a1c	H	H	14
3878	JQ705680.1	JQ705680.1; T2b19b; Asia_W; 40; A73G; A263G; C309CCT	16568	Asia_W	T2b19b	T	T	40
3879	JQ705681.1	JQ705681.1; H1o; Asia_W_Europe; 16; C150T; A263G; C315CC	16569	Asia_W_Europe	H1o	H	H	16
3880	JQ705682.1	JQ705682.1; H80; Asia_W_Europe; 11; A263G; C315CC; A750G	16559	Asia_W_Europe	H80	H	H	11
3881	JQ705683.1	JQ705683.1; J1c14; Asia_W; 35; A73G; G185A; T195C	16569	Asia_W	J1c14	J	J	35
3882	JQ705684.1	JQ705684.1; H27a; Asia_W_Europe; 16; T152C; A263G; C315CC	16569	Asia_W_Europe	H27a	H	H	16
3883	JQ705685.1	JQ705685.1; K1b1a1a; Asia_W; 43; A73G; T152C; T199C	16570	Asia_W	K1b1a1a	K	K	43
3884	JQ705686.1	JQ705686.1; K1b1a1; Asia_W; 45; A73G; T152C; A263G	16572	Asia_W	K1b1a1	K	K	45
3885	JQ705687.1	JQ705687.1; U4a1a2; Asia_N_Europe_N; 38; A73G; T152C; T195C	16571	Asia_N_Europe_N	U4a1a2	U	U4	38
3886	JQ705688.1	JQ705688.1; U5b2a2c; Asia_W_Europe_C; 33; A73G; GGAGCA106-; C150T	16563	Asia_W_Europe_C	U5b2a2c	U	U5	33
3887	JQ705689.1	JQ705689.1; K1a26; Asia_W; 33; A73G; T195C; A263G	16569	Asia_W	K1a26	K	K	33
3888	JQ705690.1	JQ705690.1; T2k; Asia_W; 38; A73G; A263G; C309CCCT	16571	Asia_W	T2k	T	T	38
3889	JQ705691.1	JQ705691.1; H; Asia_W_Europe; 13; A200G; A263G; C309CCT	16568	Asia_W_Europe	H	H	H	13
3890	JQ705692.1	JQ705692.1; H44b; Asia_W_Europe; 14; C150T; T195C; A263G	16571	Asia_W_Europe	H44b	H	H	14
3891	JQ705693.1	JQ705693.1; U5a1b1d1; Asia_W_Europe_C; 29; A73G; C198T; A263G	16569	Asia_W_Europe_C	U5a1b1d1	U	U5	29
3892	JQ705694.1	JQ705694.1; H3g1a; Asia_W_Europe; 16; C151T; T152C; A263G	16569	Asia_W_Europe	H3g1a	H	H	16
3893	JQ705695.1	JQ705695.1; H1c; Asia_W_Europe; 13; T146C; A263G; C315CC	16570	Asia_W_Europe	H1c	H	H	13
3894	JQ705696.1	JQ705696.1; U5a2b1a; Asia_W_Europe_C; 28; A73G; A263G; C315CC	16570	Asia_W_Europe_C	U5a2b1a	U	U5	28
3895	JQ705697.1	JQ705697.1; K1a1c; Asia_W; 39; A73G; C114T; A263G	16569	Asia_W	K1a1c	K	K	39

Supplementary Table S4 Human mitochondrial database (hMITO DB v1.0) metadata[a]

Row	Name (accession no.)	Description	Size	Geo_Region	Haplogroup	Macro_ Haplo	Macro_ Haplo2	Total Variants
3896	JQ705698.1	JQ705698.1; H3g1; Asia_W_Europe; 16; T152C; A263G; C309CCT	16570	Asia_W_Europe	H3g1	H	H	16
3897	JQ705699.1	JQ705699.1; H13a2b2a; Asia_W_Europe; 17; A263G; C309CCT; T310C	16570	Asia_W_Europe	H13a2b2a	H	H	17
3898	JQ705700.1	JQ705700.1; B4a1a1+16126; Asia_SE_E_America_N_S; 31; A73G; T146C; A263G	16557	Asia_SE_E_America_N_S	B4a1a1+16126	B	B	31
3899	JQ705701.1	JQ705701.1; J1c3d; Asia_W; 28; A73G; G185A; A263G	16569	Asia_W	J1c3d	J	J	28
3900	JQ705702.1	JQ705702.1; H10e; Asia_W_Europe; 14; A263G; C309CCT; T310C	16570	Asia_W_Europe	H10e	H	H	14
3901	JQ705703.1	JQ705703.1; H4a1a3a; Asia_W_Europe; 19; T195C; A263G; C315CC	16567	Asia_W_Europe	H4a1a3a	H	H	19
3902	JQ705704.1	JQ705704.1; U1b1; Asia_W; 36; A73G; T146C; A263G	16569	Asia_W	U1b1	U	U1	36
3903	JQ705705.1	JQ705705.1; K1a; Asia_W; 32; A73G; A263G; C309CCT	16576	Asia_W	K1a	K	K	32
3904	JQ705706.1	JQ705706.1; H1c3; Asia_W_Europe; 15; G185A; T195C; A257G	16570	Asia_W_Europe	H1c3	H	H	15
3905	JQ705707.1	JQ705707.1; T1a1k1; Asia_W; 40; A73G; T146C; T152C	16570	Asia_W	T1a1k1	T	T	40
3906	JQ705708.1	JQ705708.1; H3; Asia_W_Europe; 11; A263G; C309CCT; T310C	16570	Asia_W_Europe	H3	H	H	11
3907	JQ705709.1	JQ705709.1; H1b1e; Asia_W_Europe; 14; A263G; C315CC; A750G	16569	Asia_W_Europe	H1b1e	H	H	14
3908	JQ705710.1	JQ705710.1; H39b; Asia_W_Europe; 13; A263G; C309CCCT; T310C	16571	Asia_W_Europe	H39b	H	H	13
3909	JQ705711.1	JQ705711.1; U2e1a1; Asia_S_W_Europe; 44; A73G; T152C; T217C	16573	Asia_S_W_Europe	U2e1a1	U	U2	44
3910	JQ705712.1	JQ705712.1; H1c; Asia_W_Europe; 11; A263G; C309CCCT; T310C	16571	Asia_W_Europe	H1c	H	H	11
3911	JQ705713.1	JQ705713.1; T1a; Asia_W; 35; A73G; A263G; C309CCT	16570	Asia_W	T1a	T	T	35
3912	JQ705714.1	JQ705714.1; K1a4a1a2; Asia_W; 39; A73G; T146C; A263G	16572	Asia_W	K1a4a1a2	K	K	39
3913	JQ705715.1	JQ705715.1; J1c2f; Asia_W; 31; A73G; G185A; A188G	16569	Asia_W	J1c2f	J	J	31
3914	JQ705716.1	JQ705716.1; H1q1; Asia_W_Europe; 13; A263G; C309CCT; T310C	16570	Asia_W_Europe	H1q1	H	H	13
3915	JQ705717.1	JQ705717.1; H1e2; Asia_W_Europe; 13; C150T; A263G; C315CC	16569	Asia_W_Europe	H1e2	H	H	13
3916	JQ705718.1	JQ705718.1; H10a1; Asia_W_Europe; 13; A263G; C309CCT; T310C	16570	Asia_W_Europe	H10a1	H	H	13
3917	JQ705719.1	JQ705719.1; H6a1b4; Asia_W_Europe; 19; T63C; C64T; G66A	16569	Asia_W_Europe	H6a1b4	H	H	19
3918	JQ705720.1	JQ705720.1; W7; Asia_W; 37; A73G; T119C; G185A	16570	Asia_W	W7	W	W	37
3919	JQ705721.1	JQ705721.1; H7b1; Asia_W_Europe; 16; T152C; A263G; C315CC	16569	Asia_W_Europe	H7b1	H	H	16
3920	JQ705722.1	JQ705722.1; H1c; Asia_W_Europe; 11; T146C; A263G; C315CC	16569	Asia_W_Europe	H1c	H	H	11
3921	JQ705723.1	JQ705723.1; H15b1; Asia_W_Europe; 15; T55C; T57G; A93G	16569	Asia_W_Europe	H15b1	H	H	15
3922	JQ705724.1	JQ705724.1; J1c2; Asia_W; 31; A73G; G185A; A188G	16569	Asia_W	J1c2	J	J	31
3923	JQ705725.1	JQ705725.1; H3c1; Asia_W_Europe; 14; A189G; A263G; C315CC	16569	Asia_W_Europe	H3c1	H	H	14
3924	JQ705726.1	JQ705726.1; H13a1a5; Asia_W_Europe; 15; C182T; A263G; C309CCT	16570	Asia_W_Europe	H13a1a5	H	H	15
3925	JQ705727.1	JQ705727.1; H3b4a; Asia_W_Europe; 15; A263G; C309CCT; T310C	16570	Asia_W_Europe	H3b4a	H	H	15
3926	JQ705728.1	JQ705728.1; H27f; Asia_W_Europe; 13; A263G; C315CC; A750G	16569	Asia_W_Europe	H27f	H	H	13
3927	JQ705729.1	JQ705729.1; U4a1b1; Asia_N_Europe_N; 34; A73G; T152C; T195C	16569	Asia_N_Europe_N	U4a1b1	U	U4	34
3928	JQ705730.1	JQ705730.1; H6a1a3; Asia_W_Europe; 17; T239C; A263G; C309CCCT	16571	Asia_W_Europe	H6a1a3	H	H	17
3929	JQ705731.1	JQ705731.1; K1a+195; Asia_W; 35; A73G; C140T; T195C	16569	Asia_W	K1a+195	K	K	35
3930	JQ705732.1	JQ705732.1; L1b1a3; Africa_C; 82; A73G; T152C; C182T	16568	Africa_C	L1b1a3	L1	L1	82
3931	JQ705733.1	JQ705733.1; K1a+195; Asia_W; 36; A73G; T195C; A263G	16572	Asia_W	K1a+195	K	K	36
3932	JQ705734.1	JQ705734.1; H2a2b4; Asia_W_Europe; 8; A263G; C309CCT; T310C	16570	Asia_W_Europe	H2a2b4	H	H	8
3933	JQ705735.1	JQ705735.1; H3b6; Asia_W_Europe; 16; A73G; A263G; C309CCT	16570	Asia_W_Europe	H3b6	H	H	16
3934	JQ705736.1	JQ705736.1; K1b2a2; Asia_W; 38; A73G; T146C; T152C	16574	Asia_W	K1b2a2	K	K	38
3935	JQ705737.1	JQ705737.1; U5a1d2b; Asia_W_Europe_C; 36; A73G; T146C; A257G	16572	Asia_W_Europe_C	U5a1d2b	U	U5	36
3936	JQ705738.1	JQ705738.1; H4a1a4b; Asia_W_Europe; 20; A93G; T195C; A263G	16567	Asia_W_Europe	H4a1a4b	H	H	20

Supplementary Table S4 Human mitochondrial database (hMITO DB v1.0) metadata[a]

Row	Name (accession no.)	Description	Size	Geo_Region	Haplogroup	Macro_ Haplo	Macro_ Haplo2	Total Variants
3937	JQ705739.1	JQ705739.1; U3a1c1; Africa_NE_Asia_W; 31; A73G; C150T; A263G	16569	Africa_NE_Asia_W	U3a1c1	U	U3	31
3938	JQ705740.1	JQ705740.1; H11a6; Asia_W_Europe; 18; T195C; A263G; C309CCCT	16571	Asia_W_Europe	H11a6	H	H	18
3939	JQ705741.1	JQ705741.1; V; Europe_S; 17; C64T; T72C; A263G	16571	Europe_S	V	V	V	17
3940	JQ705742.1	JQ705742.1; U5b1e1; Asia_W_Europe_C; 30; A73G; C150T; T152C	16567	Asia_W_Europe_C	U5b1e1	U	U5	30
3941	JQ705743.1	JQ705743.1; HV10; Asia_W; 14; A200G; A263G; C309CCT	16579	Asia_W	HV10	HV	HV	14
3942	JQ705744.1	JQ705744.1; N1b1a; Africa_NE_Asia_W; 37; A73G; T152C; A263G	16569	Africa_NE_Asia_W	N1b1a	N	N1	37
3943	JQ705745.1	JQ705745.1; K1a1b1a; Asia_W; 38; A73G; C114T; A263G	16569	Asia_W	K1a1b1a	K	K	38
3944	JQ705746.1	JQ705746.1; A2an; Asia_NE_America_N; 36; C64T; A73G; T146C	16571	Asia_NE_America_N	A2an	A	A	36
3945	JQ705747.1	JQ705747.1; K1a11; Asia_W; 36; A16T; A73G; C150T	16562	Asia_W	K1a11	K	K	36
3946	JQ705748.1	JQ705748.1; H5c; Asia_W_Europe; 14; A263G; C315CC; C456T	16567	Asia_W_Europe	H5c	H	H	14
3947	JQ705749.1	JQ705749.1; V19; Europe_S; 16; T72C; A263G; C309CCT	16570	Europe_S	V19	V	V	16
3948	JQ705750.1	JQ705750.1; K1a; Asia_W; 36; A73G; A263G; C315CC	16571	Asia_W	K1a	K	K	36
3949	JQ705751.1	JQ705751.1; K1a30; Asia_W; 35; A73G; C150T; A263G	16570	Asia_W	K1a30	K	K	35
3950	JQ705752.1	JQ705752.1; N3a; Africa_NE_Europe_E; 39; A73G; T146C; A210G	16570	Africa_NE_Europe_E	N3a	N	N3	39
3951	JQ705753.1	JQ705753.1; L3b1a; Africa_E; 38; A73G; A263G; C315CC	16566	Africa_E	L3b1a	L3	L3	38
3952	JQ705754.1	JQ705754.1; H1bk; Asia_W_Europe; 12; A95C; A263G; C315CC	16567	Asia_W_Europe	H1bk	H	H	12
3953	JQ705755.1	JQ705755.1; B2w; Asia_SE_E_America_N_S; 33; A73G; T146C; A263G	16562	Asia_SE_E_America_N_S	B2w	B	B	33
3954	JQ705756.1	JQ705756.1; H1; Asia_W_Europe; 9; A263G; C315CC; A750G	16569	Asia_W_Europe	H1	H	H	9
3955	JQ705757.1	JQ705757.1; H1c18; Asia_W_Europe; 12; A263G; C315CC; T477C	16569	Asia_W_Europe	H1c18	H	H	12
3956	JQ705758.1	JQ705758.1; T2b; Asia_W; 37; A73G; A263G; C309CCT	16570	Asia_W	T2b	T	T	37
3957	JQ705759.1	JQ705759.1; H3u1; Asia_W_Europe; 15; C151T; A263G; C315CC	16570	Asia_W_Europe	H3u1	H	H	15
3958	JQ705760.1	JQ705760.1; J1c8a1a; Asia_W; 32; A73G; G185A; G228A	16570	Asia_W	J1c8a1a	J	J	32
3959	JQ705761.1	JQ705761.1; C1c3; Asia_NE_America_N_S; 47; A73G; A249d; A263G	16567	Asia_NE_America_N_S	C1c3	C	C	47
3960	JQ705762.1	JQ705762.1; H5a1; Asia_W_Europe; 13; A263G; C315CC; C456T	16567	Asia_W_Europe	H5a1	H	H	13
3961	JQ705763.1	JQ705763.1; H1a1; Asia_W_Europe; 14; A73G; A263G; C309CCT	16570	Asia_W_Europe	H1a1	H	H	14
3962	JQ705764.1	JQ705764.1; H1a1; Asia_W_Europe; 14; A73G; A263G; C315CC	16569	Asia_W_Europe	H1a1	H	H	14
3963	JQ705765.1	JQ705765.1; W3b1; Asia_W; 35; A73G; T195C; T199C	16570	Asia_W	W3b1	W	W	35
3964	JQ705766.1	JQ705766.1; H1c3; Asia_W_Europe; 17; T195C; A257G; A263G	16571	Asia_W_Europe	H1c3	H	H	17
3965	JQ705767.1	JQ705767.1; H5a1a; Asia_W_Europe; 16; A263G; C309CCT; T310C	16568	Asia_W_Europe	H5a1a	H	H	16
3966	JQ705768.1	JQ705768.1; N9a3; Africa_NE_Asia_E; 29; A73G; C150T; A263G	16571	Africa_NE_Asia_E	N9a3	N	N9	29
3967	JQ705769.1	JQ705769.1; H4a1a1a1a1; Asia_W_Europe; 23; A73G; T152C; A263G	16567	Asia_W_Europe	H4a1a1a1a1	H	H	23
3968	JQ705770.1	JQ705770.1; J1c1f; Asia_W; 34; A73G; G185A; G228A	16569	Asia_W	J1c1f	J	J	34
3969	JQ705771.1	JQ705771.1; K1b2b; Asia_W; 41; A73G; T146C; T195C	16571	Asia_W	K1b2b	K	K	41
3970	JQ705772.1	JQ705772.1; K1c1e; Asia_W; 36; A73G; T146C; T152C	16568	Asia_W	K1c1e	K	K	36
3971	JQ705773.1	JQ705773.1; K1a1; Asia_W; 34; A73G; C114T; A263G	16569	Asia_W	K1a1	K	K	34
3972	JQ705774.1	JQ705774.1; J2a1a1e; Asia_W; 44; C44CC; A73G; C150T	16572	Asia_W	J2a1a1e	J	J	44
3973	JQ705775.1	JQ705775.1; K1a4d; Asia_W; 37; A73G; A263G; C309CCT	16572	Asia_W	K1a4d	K	K	37
3974	JQ705776.1	JQ705776.1; K1a+195; Asia_W; 34; A73G; T195C; A263G	16569	Asia_W	K1a+195	K	K	34
3975	JQ705777.1	JQ705777.1; U5a1a2b; Asia_W_Europe_C; 27; A73G; A263G; C309CCT	16572	Asia_W_Europe_C	U5a1a2b	U	U5	27
3976	JQ705778.1	JQ705778.1; T1a1; Asia_W; 36; A73G; T152C; A183G	16569	Asia_W	T1a1	T	T	36
3977	JQ705779.1	JQ705779.1; U5a2c4; Asia_W_Europe_C; 25; A73G; A263G; C315CC	16569	Asia_W_Europe_C	U5a2c4	U	U5	25

Supplementary Table S4 Human mitochondrial database (hMITO DB v1.0) metadata[a]

Row	Name (accession no.)	Description	Size	Geo_Region	Haplogroup	Macro_ Haplo	Macro_ Haplo2	Total Variants
3978	JQ705780.1	JQ705780.1; H13a1a; Asia_W_Europe; 13; A263G; C309CCCT; T310C	16571	Asia_W_Europe	H13a1a	H	H	13
3979	JQ705781.1	JQ705781.1; K1c1b; Asia_W; 35; A73G; T146C; T152C	16568	Asia_W	K1c1b	K	K	35
3980	JQ705782.1	JQ705782.1; C5c1a; Asia_NE_America_N_S; 48; A73G; T152C; A249d	16569	Asia_NE_America_N_S	C5c1a	C	C	48
3981	JQ705783.1	JQ705783.1; L3b1a1; Africa_E; 40; A73G; A263G; C315CC	16566	Africa_E	L3b1a1	L3	L3	40
3982	JQ705784.1	JQ705784.1; K1a3; Asia_W; 33; A73G; A263G; C315CC	16569	Asia_W	K1a3	K	K	33
3983	JQ705785.1	JQ705785.1; K1a4a1; Asia_W; 37; A73G; A263G; C315CC	16569	Asia_W	K1a4a1	K	K	37
3984	JQ705786.1	JQ705786.1; T2b3c; Asia_W; 38; A73G; C151T; A263G	16570	Asia_W	T2b3c	T	T	38
3985	JQ705787.1	JQ705787.1; H5a1i; Asia_W_Europe; 17; T42TG; T199C; A263G	16568	Asia_W_Europe	H5a1i	H	H	17
3986	JQ705788.1	JQ705788.1; X2c1a; Asia_W_America_N; 30; A73G; A153G; T195C	16569	Asia_W_America_N	X2c1a	X	X	30
3987	JQ705789.1	JQ705789.1; V3a; Europe_S; 17; T72C; T195C; A263G	16570	Europe_S	V3a	V	V	17
3988	JQ705790.1	JQ705790.1; H5a1c2; Asia_W_Europe; 18; A263G; C315CC; C456T	16567	Asia_W_Europe	H5a1c2	H	H	18
3989	JQ705791.1	JQ705791.1; V5; Europe_S; 17; T72C; A93G; A263G	16570	Europe_S	V5	V	V	17
3990	JQ705792.1	JQ705792.1; J1b1a1+146; Asia_W; 39; A73G; T146C; C242T	16570	Asia_W	J1b1a1+146	J	J	39
3991	JQ705793.1	JQ705793.1; W6; Asia_W; 39; A73G; A189G; C194T	16570	Asia_W	W6	W	W	39
3992	JQ705794.1	JQ705794.1; X2b+226; Asia_W_America_N; 29; A73G; A153G; T195C	16569	Asia_W_America_N	X2b+226	X	X	29
3993	JQ705795.1	JQ705795.1; X2d; Asia_W_America_N; 32; A73G; T152C; T195C	16569	Asia_W_America_N	X2d	X	X	32
3994	JQ705796.1	JQ705796.1; H13a1a1a; Asia_W_Europe; 21; A263G; C309CCT; T310C	16570	Asia_W_Europe	H13a1a1a	H	H	21
3995	JQ705797.1	JQ705797.1; X2b+226; Asia_W_America_N; 30; A73G; A153G; T195C	16569	Asia_W_America_N	X2b+226	X	X	30
3996	JQ705798.1	JQ705798.1; V7; Europe_S; 18; T72C; A93G; T152C	16570	Europe_S	V7	V	V	18
3997	JQ705799.1	JQ705799.1; H1e1b1; Asia_W_Europe; 15; A263G; C309CCT; T310C	16570	Asia_W_Europe	H1e1b1	H	H	15
3998	JQ705800.1	JQ705800.1; H55; Asia_W_Europe; 13; T204C; A263G; C315CC	16569	Asia_W_Europe	H55	H	H	13
3999	JQ705801.1	JQ705801.1; D4; Asia_E_America_N_S; 34; A73G; T152C; A263G	16570	Asia_E_America_N_S	D4	D	D	34
4000	JQ705802.1	JQ705802.1; M1a1b1c; Africa_E_Nile Valley; 45; A73G; T195C; A263G	16569	Africa_E_Nile Valley	M1a1b1c	M	M1	45
4001	JQ705803.1	JQ705803.1; H52; Asia_W_Europe; 12; T152C; A263G; C309CCT	16570	Asia_W_Europe	H52	H	H	12
4002	JQ705804.1	JQ705804.1; J1c3; Asia_W; 30; A73G; G185A; G228A	16569	Asia_W	J1c3	J	J	30
4003	JQ705805.1	JQ705805.1; K2a6; Asia_W; 33; A73G; T146C; T152C	16569	Asia_W	K2a6	K	K	33
4004	JQ705806.1	JQ705806.1; H2a1f1; Asia_W_Europe; 10; T127C; A263G; C309CCCT	16571	Asia_W_Europe	H2a1f1	H	H	10
4005	JQ705807.1	JQ705807.1; H11a; Asia_W_Europe; 15; T195C; G207A; A263G	16570	Asia_W_Europe	H11a	H	H	15
4006	JQ705808.1	JQ705808.1; H4a1a; Asia_W_Europe; 17; A263G; C309CCT; T310C	16568	Asia_W_Europe	H4a1a	H	H	17
4007	JQ705809.1	JQ705809.1; H1e1b1; Asia_W_Europe; 16; A263G; C309CCCT; T310C	16571	Asia_W_Europe	H1e1b1	H	H	16
4008	JQ705810.1	JQ705810.1; H73a1; Asia_W_Europe; 15; A263G; C309CCCT; T310C	16571	Asia_W_Europe	H73a1	H	H	15
4009	JQ705811.1	JQ705811.1; J1c14; Asia_W; 35; A73G; G185A; T195C	16570	Asia_W	J1c14	J	J	35
4010	JQ705812.1	JQ705812.1; H5a6; Asia_W_Europe; 13; T152C; A263G; C315CC	16569	Asia_W_Europe	H5a6	H	H	13
4011	JQ705813.1	JQ705813.1; U2e1f; Asia_S_W_Europe; 43; A73G; T152C; T217C	16571	Asia_S_W_Europe	U2e1f	U	U2	43
4012	JQ705814.1	JQ705814.1; C4c1a; Asia_NE_America_N_S; 45; A73G; A249d; A263G	16569	Asia_NE_America_N_S	C4c1a	C	C	45
4013	JQ705815.1	JQ705815.1; U8b1b1; Asia_W_Europe ; 25; A73G; T195C; C315CC	16569	Asia_W_Europe	U8b1b1	U	U8	25
4014	JQ705816.1	JQ705816.1; K1a4a1f; Asia_W; 37; A73G; T152C; A263G	16567	Asia_W	K1a4a1f	K	K	37
4015	JQ705817.1	JQ705817.1; U5a2c1; Asia_W_Europe_C; 28; A73G; A263G; C315CC	16567	Asia_W_Europe_C	U5a2c1	U	U5	28
4016	JQ705818.1	JQ705818.1; H+16291; Asia_W_Europe; 14; A263G; C309CCT; T310C	16570	Asia_W_Europe	H+16291	H	H	14
4017	JQ705819.1	JQ705819.1; U8a1a1a; Asia_W_Europe ; 32; A73G; A263G; T282C	16570	Asia_W_Europe	U8a1a1a	U	U8	32
4018	JQ705820.1	JQ705820.1; G2a2a; Asia_E; 43; A73G; T152C; G207A	16568	Asia_E	G2a2a	G	G	43

Supplementary Table S4 Human mitochondrial database (hMITO DB v1.0) metadata[a]

Row	Name (accession no.)	Description	Size	Geo_Region	Haplogroup	Macro_ Haplo	Macro_ Haplo2	Total Variants
4019	JQ705821.1	JQ705821.1; K1c2; Asia_W; 36; A73G; G143A; T146C	16568	Asia_W	K1c2	K	K	36
4020	JQ705822.1	JQ705822.1; H1y; Asia_W_Europe; 14; A263G; C315CC; A750G	16569	Asia_W_Europe	H1y	H	H	14
4021	JQ705823.1	JQ705823.1; H3b; Asia_W_Europe; 15; T195C; A263G; C309CCT	16570	Asia_W_Europe	H3b	H	H	15
4022	JQ705824.1	JQ705824.1; H5c1; Asia_W_Europe; 13; A263G; C315CC; C377T	16569	Asia_W_Europe	H5c1	H	H	13
4023	JQ705825.1	JQ705825.1; H73a1; Asia_W_Europe; 15; A263G; C309CCT; T310C	16570	Asia_W_Europe	H73a1	H	H	15
4024	JQ705826.1	JQ705826.1; C1b10; Asia_NE_America_N_S; 47; A73G; A249d; AA290d	16565	Asia_NE_America_N_S	C1b10	C	C	47
4025	JQ705827.1	JQ705827.1; C5c1a; Asia_NE_America_N_S; 46; A73G; T152C; A249d	16569	Asia_NE_America_N_S	C5c1a	C	C	46
4026	JQ705828.1	JQ705828.1; U4a2f; Asia_N_Europe_N; 34; A73G; C186T; T195C	16568	Asia_N_Europe_N	U4a2f	U	U4	34
4027	JQ705829.1	JQ705829.1; K2a9; Asia_W; 36; A73G; T146C; A263G	16569	Asia_W	K2a9	K	K	36
4028	JQ705830.1	JQ705830.1; H1c2a; Asia_W_Europe; 16; T152C; A263G; C315CC	16569	Asia_W_Europe	H1c2a	H	H	16
4029	JQ705831.1	JQ705831.1; U8b1b; Asia_W_Europe ; 35; A73G; T146C; T195C	16570	Asia_W_Europe	U8b1b	U	U8	35
4030	JQ705832.1	JQ705832.1; L1b1a10; Africa_C; 78; A73G; C151T; T152C	16567	Africa_C	L1b1a10	L1	L1	78
4031	JQ705833.1	JQ705833.1; U4a1a; Asia_N_Europe_N; 34; A73G; T152C; T195C	16572	Asia_N_Europe_N	U4a1a	U	U4	34
4032	JQ705834.1	JQ705834.1; T2b; Asia_W; 36; A73G; A263G; C309CCT	16570	Asia_W	T2b	T	T	36
4033	JQ705835.1	JQ705835.1; C1d; Asia_NE_America_N_S; 52; A73G; C151T; A153G	16566	Asia_NE_America_N_S	C1d	C	C	52
4034	JQ705836.1	JQ705836.1; H1b; Asia_W_Europe; 14; A263G; C271T; C315CC	16569	Asia_W_Europe	H1b	H	H	14
4035	JQ705837.1	JQ705837.1; H2a2a1; Asia_W_Europe; 1; C315CC; ;	16569	Asia_W_Europe	H2a2a1	H	H	1
4036	JQ705838.1	JQ705838.1; K1a4a1e; Asia_W; 37; A73G; T152C; G203A	16571	Asia_W	K1a4a1e	K	K	37
4037	JQ705839.1	JQ705839.1; W1e; Asia_W; 34; A73G; A189G; T195C	16569	Asia_W	W1e	W	W	34
4038	JQ705840.1	JQ705840.1; I1; Asia_W_SW; 37; A73G; T199C; T204C	16573	Asia_W_SW	I1	I	I	37
4039	JQ705841.1	JQ705841.1; K1a3a1; Asia_W; 35; A73G; A263G; C309CCT	16570	Asia_W	K1a3a1	K	K	35
4040	JQ705842.1	JQ705842.1; H2a2b1; Asia_W_Europe; 8; A263G; C309CCT; T310C	16570	Asia_W_Europe	H2a2b1	H	H	8
4041	JQ705843.1	JQ705843.1; H3+73; Asia_W_Europe; 13; A73G; A263G; C309CCT	16570	Asia_W_Europe	H3+73	H	H	13
4042	JQ705844.1	JQ705844.1; X2b+226; Asia_W_America_N; 30; A73G; A153G; T195C	16569	Asia_W_America_N	X2b+226	X	X	30
4043	JQ705845.1	JQ705845.1; H1b; Asia_W_Europe; 13; A263G; C309CCT; T310C	16570	Asia_W_Europe	H1b	H	H	13
4044	JQ705846.1	JQ705846.1; H1e1a6; Asia_W_Europe; 16; C150T; A263G; C309CCCT	16571	Asia_W_Europe	H1e1a6	H	H	16
4045	JQ705847.1	JQ705847.1; H31b; Asia_W_Europe; 13; T146C; T195C; A263G	16569	Asia_W_Europe	H31b	H	H	13
4046	JQ705848.1	JQ705848.1; H1q; Asia_W_Europe; 15; A249d; A263G; C309CCT	16569	Asia_W_Europe	H1q	H	H	15
4047	JQ705849.1	JQ705849.1; U5a1a2a1; Asia_W_Europe_C; 35; A73G; A263G; C309CCT	16571	Asia_W_Europe_C	U5a1a2a1	U	U5	35
4048	JQ705850.1	JQ705850.1; N1a1a1a3; Africa_NE_Asia_W; 45; A73G; G143A; T152C	16572	Africa_NE_Asia_W	N1a1a1a3	N	N1	45
4049	JQ705851.1	JQ705851.1; L3e3b2; Africa_E; 39; A73G; C150T; T195C	16567	Africa_E	L3e3b2	L3	L3	39
4050	JQ705852.1	JQ705852.1; H2a1a; Asia_W_Europe; 11; A263G; C309CCT; T310C	16570	Asia_W_Europe	H2a1a	H	H	11
4051	JQ705853.1	JQ705853.1; H3r1; Asia_W_Europe; 12; A263G; C315CC; A750G	16569	Asia_W_Europe	H3r1	H	H	12
4052	JQ705854.1	JQ705854.1; T2b; Asia_W; 34; A73G; A263G; C315CC	16569	Asia_W	T2b	T	T	34
4053	JQ705855.1	JQ705855.1; U5a1a2a; Asia_W_Europe_C; 31; A73G; A263G; C309CCT	16574	Asia_W_Europe_C	U5a1a2a	U	U5	31
4054	JQ705856.1	JQ705856.1; T1a1c; Asia_W; 38; A73G; T152C; T195C	16569	Asia_W	T1a1c	T	T	38
4055	JQ705857.1	JQ705857.1; T2f1a1; Asia_W; 42; A73G; T195C; C198T	16560	Asia_W	T2f1a1	T	T	42
4056	JQ705858.1	JQ705858.1; H6a1b3; Asia_W_Europe; 18; T204C; T239C; A263G	16570	Asia_W_Europe	H6a1b3	H	H	18
4057	JQ705859.1	JQ705859.1; H56a; Asia_W_Europe; 11; A263G; C315CC; A750G	16569	Asia_W_Europe	H56a	H	H	11
4058	JQ705860.1	JQ705860.1; J1c8a; Asia_W; 29; A73G; A153G; G185A	16569	Asia_W	J1c8a	J	J	29
4059	JQ705861.1	JQ705861.1; U2e1a1b; Asia_S_W_Europe; 42; A73G; A108G; T152C	16572	Asia_S_W_Europe	U2e1a1b	U	U2	42

Supplementary Table S4 Human mitochondrial database (hMITO DB v1.0) metadata[a]

Row	Name (accession no.)	Description	Size	Geo_Region	Haplogroup	Macro_ Haplo	Macro_ Haplo2	Total Variants
4060	JQ705862.1	JQ705862.1; H24a; Asia_W_Europe; 10; A263G; C315CC; A373G	16569	Asia_W_Europe	H24a	H	H	10
4061	JQ705863.1	JQ705863.1; H5a7; Asia_W_Europe; 18; T152C; A263G; C309CCCT	16569	Asia_W_Europe	H5a7	H	H	18
4062	JQ705864.1	JQ705864.1; L1c2b1a1; Africa_C; 101; A73G; C151T; T152C	16570	Africa_C	L1c2b1a1	L1	L1	101
4063	JQ705865.1	JQ705865.1; U4b1a1a; Asia_N_Europe_N; 34; A73G; T195C; A263G	16570	Asia_N_Europe_N	U4b1a1a	U	U4	34
4064	JQ705866.1	JQ705866.1; H10+(16093); Asia_W_Europe; 12; A263G; C309CCCT; T310C	16571	Asia_W_Europe	H10+(16093)	H	H	12
4065	JQ705867.1	JQ705867.1; H1u1; Asia_W_Europe; 13; A263G; C309CCCT; T310C	16573	Asia_W_Europe	H1u1	H	H	13
4066	JQ705868.1	JQ705868.1; J1c3e1; Asia_W; 33; A73G; G185A; G228A	16570	Asia_W	J1c3e1	J	J	33
4067	JQ705869.1	JQ705869.1; K1c1b; Asia_W; 35; A73G; T146C; T152C	16568	Asia_W	K1c1b	K	K	35
4068	JQ705870.1	JQ705870.1; U5b1c1; Asia_W_Europe_C; 32; A73G; G94A; C150T	16570	Asia_W_Europe_C	U5b1c1	U	U5	32
4069	JQ705871.1	JQ705871.1; U3a1a; Africa_NE_Asia_W; 32; A73G; C150T; A263G	16570	Africa_NE_Asia_W	U3a1a	U	U3	32
4070	JQ705872.1	JQ705872.1; H1j; Asia_W_Europe; 12; A263G; C309CCT; T310C	16570	Asia_W_Europe	H1j	H	H	12
4071	JQ705873.1	JQ705873.1; H6a1b2; Asia_W_Europe; 16; T239C; A263G; C315CC	16569	Asia_W_Europe	H6a1b2	H	H	16
4072	JQ705874.1	JQ705874.1; J1c1a; Asia_W; 32; A73G; G228A; A263G	16569	Asia_W	J1c1a	J	J	32
4073	JQ705875.1	JQ705875.1; H24; Asia_W_Europe; 14; T146C; A263G; C315CC	16569	Asia_W_Europe	H24	H	H	14
4074	JQ705876.1	JQ705876.1; H1; Asia_W_Europe; 11; A263G; C309CCT; T310C	16570	Asia_W_Europe	H1	H	H	11
4075	JQ705877.1	JQ705877.1; U5b2b4a; Asia_W_Europe_C; 32; A73G; C150T; G185A	16571	Asia_W_Europe_C	U5b2b4a	U	U5	32
4076	JQ705878.1	JQ705878.1; H2a2a1f; Asia_W_Europe; 4; A93G; C309CCT; T310C	16570	Asia_W_Europe	H2a2a1f	H	H	4
4077	JQ705879.1	JQ705879.1; T2f1a1; Asia_W; 42; A73G; T195C; A263G	16560	Asia_W	T2f1a1	T	T	42
4078	JQ705880.1	JQ705880.1; H7d1; Asia_W_Europe; 14; A263G; C309CCT; T310C	16570	Asia_W_Europe	H7d1	H	H	14
4079	JQ705881.1	JQ705881.1; B2+16278; Asia_SE_E_America_N_S; 35; A73G; A200G; A263G	16564	Asia_SE_E_America_N_S	B2+16278	B	B	35
4080	JQ705882.1	JQ705882.1; T2b25; Asia_W; 40; A73G; T146C; A263G	16574	Asia_W	T2b25	T	T	40
4081	JQ705883.1	JQ705883.1; T2b4b; Asia_W; 38; A73G; T152C; A263G	16570	Asia_W	T2b4b	T	T	38
4082	JQ705884.1	JQ705884.1; H1e1a5; Asia_W_Europe; 21; A263G; C315CC; A750G	16569	Asia_W_Europe	H1e1a5	H	H	21
4083	JQ705885.1	JQ705885.1; V27; Europe_S; 18; T72C; A263G; C309CCT	16570	Europe_S	V27	V	V	18
4084	JQ705886.1	JQ705886.1; U5a1a2b1; Asia_W_Europe_C; 33; A73G; A263G; C309CCT	16570	Asia_W_Europe_C	U5a1a2b1	U	U5	33
4085	JQ705887.1	JQ705887.1; H3t; Asia_W_Europe; 10; A263G; C315CC; A750G	16569	Asia_W_Europe	H3t	H	H	10
4086	JQ705888.1	JQ705888.1; H10e; Asia_W_Europe; 12; A263G; C309CCT; T310C	16570	Asia_W_Europe	H10e	H	H	12
4087	JQ705889.1	JQ705889.1; I1a1; Asia_W_SW; 42; A73G; T199C; G203A	16573	Asia_W_SW	I1a1	I	I	42
4088	JQ705890.1	JQ705890.1; H27a; Asia_W_Europe; 12; A263G; C315CC; A750G	16569	Asia_W_Europe	H27a	H	H	12
4089	JQ705891.1	JQ705891.1; J1c14; Asia_W; 36; A73G; G185A; T195C	16570	Asia_W	J1c14	J	J	36
4090	JQ705892.1	JQ705892.1; T2f1a1; Asia_W; 43; A73G; T195C; A263G	16560	Asia_W	T2f1a1	T	T	43
4091	JQ705893.1	JQ705893.1; H94; Asia_W_Europe; 11; A263G; C315CC; A750G	16569	Asia_W_Europe	H94	H	H	11
4092	JQ705894.1	JQ705894.1; H1c4b; Asia_W_Europe; 14; T152C; A263G; C315CC	16569	Asia_W_Europe	H1c4b	H	H	14
4093	JQ705895.1	JQ705895.1; T2b21a; Asia_W; 39; A73G; T152C; A263G	16569	Asia_W	T2b21a	T	T	39
4094	JQ705896.1	JQ705896.1; G3a2a; Asia_E; 40; A73G; G143A; T152C	16571	Asia_E	G3a2a	G	G	40
4095	JQ705897.1	JQ705897.1; T2f1a1; Asia_W; 41; A73G; T195C; A263G	16560	Asia_W	T2f1a1	T	T	41
4096	JQ705898.1	JQ705898.1; H11a; Asia_W_Europe; 14; T195C; A263G; C309CCCT	16571	Asia_W_Europe	H11a	H	H	14
4097	JQ705899.1	JQ705899.1; T2b19; Asia_W; 36; A73G; A263G; C315CC	16567	Asia_W	T2b19	T	T	36
4098	JQ705900.1	JQ705900.1; U2e2a; Asia_S_W_Europe; 44; A73G; T152C; T217C	16570	Asia_S_W_Europe	U2e2a	U	U2	44
4099	JQ705901.1	JQ705901.1; K1a+150; Asia_W; 33; A73G; C150T; T195C	16573	Asia_W	K1a+150	K	K	33
4100	JQ705902.1	JQ705902.1; L3e1a3a; Africa_E; 34; A73G; C150T; A200G	16569	Africa_E	L3e1a3a	L3	L3	34

Supplementary Table S4 Human mitochondrial database (hMITO DB v1.0) metadata[a]

Row	Name (accession no.)	Description	Size	Geo_Region	Haplogroup	Macro_ Haplo	Macro_ Haplo2	Total Variants
4101	JQ705903.1	JQ705903.1; H1e; Asia_W_Europe; 14; A263G; C309CCT; T310C	16570	Asia_W_Europe	H1e	H	H	14
4102	JQ705904.1	JQ705904.1; D1; Asia_E_America_N_S; 39; A73G; G143A; A263G	16569	Asia_E_America_N_S	D1	D	D	39
4103	JQ705905.1	JQ705905.1; H3ae; Asia_W_Europe; 12; A263G; C309CCT; T310C	16570	Asia_W_Europe	H3ae	H	H	12
4104	JQ705906.1	JQ705906.1; I4a1; Asia_W_SW; 34; A73G; T199C; T204C	16571	Asia_W_SW	I4a1	I	I	34
4105	JQ705907.1	JQ705907.1; U4a2a; Asia_N_Europe_N; 30; A73G; C150T; T195C	16570	Asia_N_Europe_N	U4a2a	U	U4	30
4106	JQ705908.1	JQ705908.1; U5a1c2a1; Asia_W_Europe_C; 31; A73G; A183G; G184A	16570	Asia_W_Europe_C	U5a1c2a1	U	U5	31
4107	JQ705909.1	JQ705909.1; J1b1a1; Asia_W; 38; A73G; C242T; A263G	16569	Asia_W	J1b1a1	J	J	38
4108	JQ705910.1	JQ705910.1; U5a1h; Asia_W_Europe_C; 35; A73G; C150T; A263G	16570	Asia_W_Europe_C	U5a1h	U	U5	35
4109	JQ705911.1	JQ705911.1; U1b1; Asia_W; 32; A73G; T146C; A263G	16572	Asia_W	U1b1	U	U1	32
4110	JQ705912.1	JQ705912.1; L3d1d; Africa_E; 42; A73G; T152C; A263G	16568	Africa_E	L3d1d	L3	L3	42
4111	JQ705913.1	JQ705913.1; T2b3+151; Asia_W; 39; A73G; C151T; A263G	16570	Asia_W	T2b3+151	T	T	39
4112	JQ705914.1	JQ705914.1; H1j8; Asia_W_Europe; 16; A263G; C309CCCT; T310C	16571	Asia_W_Europe	H1j8	H	H	16
4113	JQ705915.1	JQ705915.1; H3z; Asia_W_Europe; 12; T152C; A263G; T293C	16571	Asia_W_Europe	H3z	H	H	12
4114	JQ705916.1	JQ705916.1; R0a2m; Africa_NE_Asia_W; 21; T57TC; C64T; A263G	16570	Africa_NE_Asia_W	R0a2m	R	R0	21
4115	JQ705917.1	JQ705917.1; H34; Asia_W_Europe; 14; T152C; A263G; C315CC	16569	Asia_W_Europe	H34	H	H	14
4116	JQ705918.1	JQ705918.1; H1ad; Asia_W_Europe; 13; A263G; C309CCCT; T310C	16573	Asia_W_Europe	H1ad	H	H	13
4117	JQ705919.1	JQ705919.1; U4a2; Asia_N_Europe_N; 30; A73G; T195C; A263G	16570	Asia_N_Europe_N	U4a2	U	U4	30
4118	JQ705920.1	JQ705920.1; C1b10; Asia_NE_America_N_S; 46; A73G; A249d; AA290d	16565	Asia_NE_America_N_S	C1b10	C	C	46
4119	JQ705921.1	JQ705921.1; I2a2; Asia_W_SW; 41; A73G; T152C; T199C	16574	Asia_W_SW	I2a2	I	I	41
4120	JQ705922.1	JQ705922.1; J1c3; Asia_W; 30; A73G; C182T; G185A	16569	Asia_W	J1c3	J	J	30
4121	JQ705923.1	JQ705923.1; J1b1a1; Asia_W; 37; A73G; A153G; C242T	16569	Asia_W	J1b1a1	J	J	37
4122	JQ705924.1	JQ705924.1; H23; Asia_W_Europe; 11; A263G; C309CCT; T310C	16572	Asia_W_Europe	H23	H	H	11
4123	JQ705925.1	JQ705925.1; J2b1a; Asia_W; 32; A73G; C150T; T152C	16570	Asia_W	J2b1a	J	J	32
4124	JQ705926.1	JQ705926.1; U4d3; Asia_N_Europe_N; 33; A73G; T195C; A263G	16572	Asia_N_Europe_N	U4d3	U	U4	33
4125	JQ705927.1	JQ705927.1; T2f1a1; Asia_W; 40; A73G; T195C; A263G	16560	Asia_W	T2f1a1	T	T	40
4126	JQ705928.1	JQ705928.1; H11a; Asia_W_Europe; 16; T195C; A263G; C309CCT	16570	Asia_W_Europe	H11a	H	H	16
4127	JQ705929.1	JQ705929.1; H35a; Asia_W_Europe; 14; A263G; C309CCT; T310C	16570	Asia_W_Europe	H35a	H	H	14
4128	JQ705930.1	JQ705930.1; H1n1b; Asia_W_Europe; 16; T146C; A263G; C309CCCT	16573	Asia_W_Europe	H1n1b	H	H	16
4129	JQ705931.1	JQ705931.1; L1b1a12b; Africa_C; 78; A73G; T152C; C182T	16567	Africa_C	L1b1a12b	L1	L1	78
4130	JQ705932.1	JQ705932.1; I1c; Asia_W_SW; 43; A73G; T199C; T204C	16572	Asia_W_SW	I1c	I	I	43
4131	JQ705933.1	JQ705933.1; U2e1c1; Asia_S_W_Europe; 38; A73G; T152C; T217C	16569	Asia_S_W_Europe	U2e1c1	U	U2	38
4132	JQ705934.1	JQ705934.1; H; Asia_W_Europe; 10; A73G; A263G; C315CC	16569	Asia_W_Europe	H	H	H	10
4133	JQ705935.1	JQ705935.1; U5a1b+16362; Asia_W_Europe_C; 28; A73G; A263G; C309CCT	16570	Asia_W_Europe_C	U5a1b+16362	U	U5	28
4134	JQ705936.1	JQ705936.1; U2e1a1a; Asia_S_W_Europe; 41; A73G; T152C; T217C	16571	Asia_S_W_Europe	U2e1a1a	U	U2	41
4135	JQ705937.1	JQ705937.1; H10e1; Asia_W_Europe; 12; A263G; C309CCT; T310C	16570	Asia_W_Europe	H10e1	H	H	12
4136	JQ705938.1	JQ705938.1; K1a4a1e; Asia_W; 37; A73G; T152C; T204C	16571	Asia_W	K1a4a1e	K	K	37
4137	JQ705939.1	JQ705939.1; T2b4b; Asia_W; 35; A73G; T152C; A263G	16569	Asia_W	T2b4b	T	T	35
4138	JQ705940.1	JQ705940.1; H1bo; Asia_W_Europe; 15; A263G; C309CCCT; T310C	16571	Asia_W_Europe	H1bo	H	H	15
4139	JQ705941.1	JQ705941.1; U2e1b; Asia_S_W_Europe; 46; A73G; C151T; T152C	16571	Asia_S_W_Europe	U2e1b	U	U2	46
4140	JQ705942.1	JQ705942.1; V5; Europe_S; 17; T72C; A93G; A263G	16571	Europe_S	V5	V	V	17
4141	JQ705943.1	JQ705943.1; C1b5a; Asia_NE_America_N_S; 48; A73G; A249d; A263G	16565	Asia_NE_America_N_S	C1b5a	C	C	48

Supplementary Table S4 Human mitochondrial database (hMITO DB v1.0) metadata[a]

Row	Name (accession no.)	Description	Size	Geo_Region	Haplogroup	Macro_ Haplo	Macro_ Haplo2	Total Variants
4142	JQ705944.1	JQ705944.1; H1r; Asia_W_Europe; 12; A263G; C309CCT; T310C	16570	Asia_W_Europe	H1r	H	H	12
4143	JQ705945.1	JQ705945.1; V7b; Europe_S; 18; T72C; A93G; A263G	16570	Europe_S	V7b	V	V	18
4144	JQ705946.1	JQ705946.1; U2e1b2; Asia_S_W_Europe; 45; A73G; T152C; T217C	16574	Asia_S_W_Europe	U2e1b2	U	U2	45
4145	JQ705947.1	JQ705947.1; U5b2c2a; Asia_W_Europe_C; 32; A73G; C150T; A263G	16570	Asia_W_Europe_C	U5b2c2a	U	U5	32
4146	JQ705948.1	JQ705948.1; T2b; Asia_W; 35; A73G; T195C; A263G	16571	Asia_W	T2b	T	T	35
4147	JQ705949.1	JQ705949.1; H2c; Asia_W_Europe; 16; A263G; C309CCCT; T310C	16569	Asia_W_Europe	H2c	H	H	16
4148	JQ705950.1	JQ705950.1; H10e1; Asia_W_Europe; 13; A263G; C309CCT; T310C	16571	Asia_W_Europe	H10e1	H	H	13
4149	JQ705951.1	JQ705951.1; K1a1b1a; Asia_W; 38; A73G; C114T; A263G	16569	Asia_W	K1a1b1a	K	K	38
4150	JQ705952.1	JQ705952.1; A5a1a1; Asia_NE_America_N; 35; A73G; T195C; A235G	16569	Asia_NE_America_N	A5a1a1	A	A	35
4151	JQ705953.1	JQ705953.1; H; Asia_W_Europe; 8; A263G; C315CC; A750G	16569	Asia_W_Europe	H	H	H	8
4152	JQ705954.1	JQ705954.1; H11a2a2; Asia_W_Europe; 21; T195C; A263G; C315CC	16569	Asia_W_Europe	H11a2a2	H	H	21
4153	JQ705955.1	JQ705955.1; T1a1; Asia_W; 36; A73G; T152C; T195C	16570	Asia_W	T1a1	T	T	36
4154	JQ705956.1	JQ705956.1; H2a2a2; Asia_W_Europe; 9; T152C; A263G; C309CCT	16570	Asia_W_Europe	H2a2a2	H	H	9
4155	JQ705957.1	JQ705957.1; K1a3a2; Asia_W; 35; A73G; A263G; C315CC	16569	Asia_W	K1a3a2	K	K	35
4156	JQ705958.1	JQ705958.1; H1b1; Asia_W_Europe; 11; A263G; C315CC; A750G	16569	Asia_W_Europe	H1b1	H	H	11
4157	JQ705959.1	JQ705959.1; R31a; Asia_S_SE; 32; A73G; T146C; A263G	16568	Asia_S_SE	R31a	R	R31	32
4158	JQ705960.1	JQ705960.1; H10a; Asia_W_Europe; 12; A263G; C309CCCT; T310C	16571	Asia_W_Europe	H10a	H	H	12
4159	JQ705961.1	JQ705961.1; J1c2c1a; Asia_W; 37; A73G; T146C; G185A	16569	Asia_W	J1c2c1a	J	J	37
4160	JQ705962.1	JQ705962.1; X2b4; Asia_W_America_N; 33; A73G; A153G; T195C	16570	Asia_W_America_N	X2b4	X	X	33
4161	JQ705963.1	JQ705963.1; HV0e; Asia_W; 17; T195C; A263G; C309CCT	16570	Asia_W	HV0e	HV	HV	17
4162	JQ705964.1	JQ705964.1; J1b1a1c; Asia_W; 39; T10C; A73G; T146C	16569	Asia_W	J1b1a1c	J	J	39
4163	JQ705965.1	JQ705965.1; M5a1; Asia_S; 43; A73G; T146C; T195C	16570	Asia_S	M5a1	M	M5	43
4164	JQ705966.1	JQ705966.1; U7a3a; Asia_W; 37; A73G; C151T; T152C	16567	Asia_W	U7a3a	U	U7	37
4165	JQ705967.1	JQ705967.1; HV0e; Asia_W; 17; T72C; T195C; A263G	16570	Asia_W	HV0e	HV	HV	17
4166	JQ705968.1	JQ705968.1; U5b3; Asia_W_Europe_C; 27; A73G; C150T; A263G	16569	Asia_W_Europe_C	U5b3	U	U5	27
4167	JQ705969.1	JQ705969.1; H56; Asia_W_Europe; 13; A263G; C315CC; A750G	16569	Asia_W_Europe	H56	H	H	13
4168	JQ705970.1	JQ705970.1; X2b8; Asia_W_America_N; 33; A73G; T195C; G225A	16570	Asia_W_America_N	X2b8	X	X	33
4169	JQ705971.1	JQ705971.1; H5a1; Asia_W_Europe; 15; A263G; C309CCT; T310C	16568	Asia_W_Europe	H5a1	H	H	15
4170	JQ705972.1	JQ705972.1; T2b6b; Asia_W; 44; A73G; T146C; A263G	16570	Asia_W	T2b6b	T	T	44
4171	JQ705973.1	JQ705973.1; A2q; Asia_NE_America_N; 32; C64T; A73G; T146C	16567	Asia_NE_America_N	A2q	A	A	32
4172	JQ705974.1	JQ705974.1; H3+152; Asia_W_Europe; 14; T152C; A263G; C309CCT	16570	Asia_W_Europe	H3+152	H	H	14
4173	JQ705975.1	JQ705975.1; H66a; Asia_W_Europe; 11; A263G; C315CC; A750G	16569	Asia_W_Europe	H66a	H	H	11
4174	JQ705976.1	JQ705976.1; H1j; Asia_W_Europe; 11; T152C; A263G; C315CC	16569	Asia_W_Europe	H1j	H	H	11
4175	JQ705977.1	JQ705977.1; J1c9; Asia_W; 32; A73G; G185A; G228A	16569	Asia_W	J1c9	J	J	32
4176	JQ705978.1	JQ705978.1; H2a2a1; Asia_W_Europe; 1; C315CC; ;	16569	Asia_W_Europe	H2a2a1	H	H	1
4177	JQ705979.1	JQ705979.1; K1a1b1a; Asia_W; 35; A73G; A263G; C315CC	16569	Asia_W	K1a1b1a	K	K	35
4178	JQ705980.1	JQ705980.1; K1a4a1a+195; Asia_W; 38; A73G; T195C; A263G	16571	Asia_W	K1a4a1a+195	K	K	38
4179	JQ705981.1	JQ705981.1; H13a1a1a; Asia_W_Europe; 16; T152C; A263G; C309CCT	16570	Asia_W_Europe	H13a1a1a	H	H	16
4180	JQ705982.1	JQ705982.1; T1a1a; Asia_W; 36; A73G; T152C; T195C	16569	Asia_W	T1a1a	T	T	36
4181	JQ705983.1	JQ705983.1; H8c; Asia_W_Europe; 16; T146C; T152C; T195C	16569	Asia_W_Europe	H8c	H	H	16
4182	JQ705984.1	JQ705984.1; H11a; Asia_W_Europe; 14; T195C; A263G; C309CCCT	16571	Asia_W_Europe	H11a	H	H	14

Supplementary Table S4 Human mitochondrial database (hMITO DB v1.0) metadata[a]

Row	Name (accession no.)	Description	Size	Geo_Region	Haplogroup	Macro_ Haplo	Macro_ Haplo2	Total Variants
4183	JQ705985.1	JQ705985.1; H1f1; Asia_W_Europe; 14; A263G; C315CC; A750G	16569	Asia_W_Europe	H1f1	H	H	14
4184	JQ705986.1	JQ705986.1; J1c2b; Asia_W; 33; A73G; T152C; G185A	16570	Asia_W	J1c2b	J	J	33
4185	JQ705987.1	JQ705987.1; H1n+146; Asia_W_Europe; 13; T146C; A263G; C309CCT	16570	Asia_W_Europe	H1n+146	H	H	13
4186	JQ705988.1	JQ705988.1; X2n; Asia_W_America_N; 36; C64T; A73G; A153G	16568	Asia_W_America_N	X2n	X	X	36
4187	JQ705989.1	JQ705989.1; U2e1a1; Asia_S_W_Europe; 39; A73G; T152C; T217C	16571	Asia_S_W_Europe	U2e1a1	U	U2	39
4188	JQ705990.1	JQ705990.1; W3b; Asia_W; 41; A73G; A189G; C194T	16570	Asia_W	W3b	W	W	41
4189	JQ705991.1	JQ705991.1; M5a1b; Asia_S; 39; A73G; A263G; C309CCT	16570	Asia_S	M5a1b	M	M5	39
4190	JQ705992.1	JQ705992.1; H1c1; Asia_W_Europe; 14; A263G; C315CC; T477C	16569	Asia_W_Europe	H1c1	H	H	14
4191	JQ705993.1	JQ705993.1; H1a3b; Asia_W_Europe; 15; A73G; T195C; A263G	16567	Asia_W_Europe	H1a3b	H	H	15
4192	JQ705994.1	JQ705994.1; K1c2; Asia_W; 36; A73G; T146C; T152C	16568	Asia_W	K1c2	K	K	36
4193	JQ705995.1	JQ705995.1; T2b6b; Asia_W; 44; A73G; T146C; A263G	16570	Asia_W	T2b6b	T	T	44
4194	JQ705996.1	JQ705996.1; J1c2h; Asia_W; 31; A73G; G185A; A188G	16570	Asia_W	J1c2h	J	J	31
4195	JQ705997.1	JQ705997.1; H28a; Asia_W_Europe; 14; C186A; T195C; A263G	16569	Asia_W_Europe	H28a	H	H	14
4196	JQ705998.1	JQ705998.1; H5a1m; Asia_W_Europe; 16; T152C; A263G; C309CCT	16568	Asia_W_Europe	H5a1m	H	H	16
4197	JQ705999.1	JQ705999.1; U2d2; Asia_S_W_Europe; 37; A73G; T152C; T199C	16570	Asia_S_W_Europe	U2d2	U	U2	37
4198	JQ706000.1	JQ706000.1; H51a; Asia_W_Europe; 13; A263G; C309CCT; T310C	16570	Asia_W_Europe	H51a	H	H	13
4199	JQ706001.1	JQ706001.1; K1a2b; Asia_W; 35; A73G; T152C; A263G	16571	Asia_W	K1a2b	K	K	35
4200	JQ706002.1	JQ706002.1; H1c6; Asia_W_Europe; 13; C41T; A263G; C309CCT	16570	Asia_W_Europe	H1c6	H	H	13
4201	JQ706003.1	JQ706003.1; H11a+152; Asia_W_Europe; 17; T152C; T195C; A263G	16571	Asia_W_Europe	H11a+152	H	H	17
4202	JQ706004.1	JQ706004.1; H13a1a1a; Asia_W_Europe; 18; A263G; C309CCT; T310C	16570	Asia_W_Europe	H13a1a1a	H	H	18
4203	JQ706005.1	JQ706005.1; U4a1a; Asia_N_Europe_N; 35; A73G; C150T; T152C	16572	Asia_N_Europe_N	U4a1a	U	U4	35
4204	JQ706006.1	JQ706006.1; K1a1b1a; Asia_W; 38; A73G; C114T; T152C	16569	Asia_W	K1a1b1a	K	K	38
4205	JQ706007.1	JQ706007.1; K1c2; Asia_W; 36; A73G; T146C; T152C	16568	Asia_W	K1c2	K	K	36
4206	JQ706008.1	JQ706008.1; T2a1a; Asia_W; 34; A73G; A263G; C309CCT	16570	Asia_W	T2a1a	T	T	34
4207	JQ706009.1	JQ706009.1; H1c1; Asia_W_Europe; 13; A263G; C315CC; T477C	16569	Asia_W_Europe	H1c1	H	H	13
4208	JQ706010.1	JQ706010.1; H1b2; Asia_W_Europe; 14; A263G; C309CCT; T310C	16570	Asia_W_Europe	H1b2	H	H	14
4209	JQ706011.1	JQ706011.1; H2a2b; Asia_W_Europe; 8; A263G; C309CCCT; T310C	16571	Asia_W_Europe	H2a2b	H	H	8
4210	JQ706012.1	JQ706012.1; H3i1; Asia_W_Europe; 12; T152C; A263G; C315CC	16569	Asia_W_Europe	H3i1	H	H	12
4211	JQ706013.1	JQ706013.1; J1c3g; Asia_W; 29; A73G; G185A; A263G	16569	Asia_W	J1c3g	J	J	29
4212	JQ706014.1	JQ706014.1; L2a1a1; Africa_W_C; 62; A73G; T146C; T152C	16574	Africa_W_C	L2a1a1	L2	L2	62
4213	JQ706015.1	JQ706015.1; U5a1b1a1; Asia_W_Europe_C; 29; A73G; A263G; C315CC	16569	Asia_W_Europe_C	U5a1b1a1	U	U5	29
4214	JQ706016.1	JQ706016.1; HV0+195; Asia_W; 15; T72C; T195C; A263G	16570	Asia_W	HV0+195	HV	HV	15
4215	JQ706017.1	JQ706017.1; I4a; Asia_W_SW; 36; A73G; T199C; T204C	16572	Asia_W_SW	I4a	I	I	36
4216	JQ706018.1	JQ706018.1; H2a(H2a+152 16311); Asia_W_Europe; 2; C315CC; T16311C;	16569	Asia_W_Europe	H2a(H2a+152 1	H	H	2
4217	JQ706019.1	JQ706019.1; U5b2b; Asia_W_Europe_C; 28; A73G; C150T; A263G	16569	Asia_W_Europe_C	U5b2b	U	U5	28
4218	JQ706020.1	JQ706020.1; K1a4c1; Asia_W; 38; A73G; T152C; A263G	16571	Asia_W	K1a4c1	K	K	38
4219	JQ706021.1	JQ706021.1; W1c; Asia_W; 38; A73G; T119C; A189G	16569	Asia_W	W1c	W	W	38
4220	JQ706022.1	JQ706022.1; J1c17a; Asia_W; 31; A73G; G185A; G228A	16569	Asia_W	J1c17a	J	J	31
4221	JQ706023.1	JQ706023.1; X2b; Asia_W_America_N; 34; A73G; A153G; T195C	16570	Asia_W_America_N	X2b	X	X	34
4222	JQ706024.1	JQ706024.1; W3a1c; Asia_W; 38; A73G; A189G; C194T	16570	Asia_W	W3a1c	W	W	38
4223	JQ706025.1	JQ706025.1; U2d3; Asia_S_W_Europe; 34; A73G; T152C; T199C	16569	Asia_S_W_Europe	U2d3	U	U2	34

Supplementary Table S4 Human mitochondrial database (hMITO DB v1.0) metadata[a]

Row	Name (accession no.)	Description	Size	Geo_Region	Haplogroup	Macro_ Haplo	Macro_ Haplo2	Total Variants
4224	JQ706026.1	JQ706026.1; U2d2a; Asia_S_W_Europe; 35; A73G; T152C; A188G	16569	Asia_S_W_Europe	U2d2a	U	U2	35
4225	JQ706027.1	JQ706027.1; K1a; Asia_W; 33; A73G; A263G; C497T	16569	Asia_W	K1a	K	K	33
4226	JQ706028.1	JQ706028.1; K1c2; Asia_W; 38; A73G; T146C; T152C	16569	Asia_W	K1c2	K	K	38
4227	JQ706029.1	JQ706029.1; K1a7; Asia_W; 30; A73G; A263G; C431A	16569	Asia_W	K1a7	K	K	30
4228	JQ706030.1	JQ706030.1; K1a13a; Asia_W; 36; A73G; T146C; T195C	16569	Asia_W	K1a13a	K	K	36
4229	JQ706031.1	JQ706031.1; K1a; Asia_W; 34; A73G; T152C; A263G	16569	Asia_W	K1a	K	K	34
4230	JQ706032.1	JQ706032.1; U2d1; Asia_S_W_Europe; 36; A73G; T152C; T199C	16569	Asia_S_W_Europe	U2d1	U	U2	36
4231	JQ706033.1	JQ706033.1; K1a+195; Asia_W; 37; A73G; T152C; T195C	16569	Asia_W	K1a+195	K	K	37
4232	JQ706034.1	JQ706034.1; K1a17; Asia_W; 31; A73G; G247A; A263G	16569	Asia_W	K1a17	K	K	31
4233	JQ706035.1	JQ706035.1; K1a12a1; Asia_W; 35; A73G; A263G; C497T	16568	Asia_W	K1a12a1	K	K	35
4234	JQ706036.1	JQ706036.1; K1a12a1; Asia_W; 40; A73G; C150T; T152C	16569	Asia_W	K1a12a1	K	K	40
4235	JQ706037.1	JQ706037.1; K1a; Asia_W; 32; A73G; A263G; C497T	16569	Asia_W	K1a	K	K	32
4236	JQ706038.1	JQ706038.1; K1a; Asia_W; 28; A73G; A263G; C497T	16569	Asia_W	K1a	K	K	28
4237	JQ706039.1	JQ706039.1; K1a19a; Asia_W; 38; T63C; C64T; A73G	16569	Asia_W	K1a19a	K	K	38
4238	JQ706040.1	JQ706040.1; K1a19; Asia_W; 34; A73G; A263G; C497T	16568	Asia_W	K1a19	K	K	34
4239	JQ706041.1	JQ706041.1; U2d1; Asia_S_W_Europe; 35; A73G; T152C; C182T	16569	Asia_S_W_Europe	U2d1	U	U2	35
4240	JQ706042.1	JQ706042.1; K1a23; Asia_W; 34; A73G; T195C; A263G	16569	Asia_W	K1a23	K	K	34
4241	JQ706043.1	JQ706043.1; K1a8b; Asia_W; 35; A73G; T152C; A263G	16569	Asia_W	K1a8b	K	K	35
4242	JQ706044.1	JQ706044.1; K1a3; Asia_W; 31; A73G; A263G; C497T	16569	Asia_W	K1a3	K	K	31
4243	JQ706045.1	JQ706045.1; U2d3; Asia_S_W_Europe; 34; A73G; T152C; T199C	16569	Asia_S_W_Europe	U2d3	U	U2	34
4244	JQ706046.1	JQ706046.1; U2d2a; Asia_S_W_Europe; 34; A73G; T152C; T199C	16569	Asia_S_W_Europe	U2d2a	U	U2	34
4245	JQ706047.1	JQ706047.1; U2d1; Asia_S_W_Europe; 31; A73G; T152C; T199C	16569	Asia_S_W_Europe	U2d1	U	U2	31
4246	JQ706048.1	JQ706048.1; K1a18; Asia_W; 34; A73G; T152C; A263G	16569	Asia_W	K1a18	K	K	34
4247	JQ706049.1	JQ706049.1; K1a+150; Asia_W; 32; A73G; C150T; A243G	16569	Asia_W	K1a+150	K	K	32
4248	JQ706050.1	JQ706050.1; K1a12a; Asia_W; 35; A73G; A263G; C497T	16568	Asia_W	K1a12a	K	K	35
4249	JQ706051.1	JQ706051.1; K1a25; Asia_W; 36; A73G; T152C; A263G	16569	Asia_W	K1a25	K	K	36
4250	JQ706052.1	JQ706052.1; K1a8b; Asia_W; 36; A73G; A263G; C295T	16569	Asia_W	K1a8b	K	K	36
4251	JQ706053.1	JQ706053.1; K1a; Asia_W; 32; A73G; T173C; A263G	16569	Asia_W	K1a	K	K	32
4252	JQ706054.1	JQ706054.1; K1a18; Asia_W; 33; A73G; T152C; A263G	16569	Asia_W	K1a18	K	K	33
4253	JQ706055.1	JQ706055.1; K1a7; Asia_W; 31; A73G; A263G; C431A	16569	Asia_W	K1a7	K	K	31
4254	JQ706056.1	JQ706056.1; K1a31; Asia_W; 30; A73G; C150T; A263G	16569	Asia_W	K1a31	K	K	30
4255	JQ706057.1	JQ706057.1; K1a1b1e; Asia_W; 33; A73G; A263G; C497T	16569	Asia_W	K1a1b1e	K	K	33
4256	JQ706058.1	JQ706058.1; K1a19; Asia_W; 34; A73G; A263G; C497T	16569	Asia_W	K1a19	K	K	34
4257	JQ706059.1	JQ706059.1; K1a5b; Asia_W; 35; A73G; A153G; A263G	16569	Asia_W	K1a5b	K	K	35
4258	JQ706060.1	JQ706060.1; K1a; Asia_W; 34; A73G; T146C; A263G	16569	Asia_W	K1a	K	K	34
4259	JQ706061.1	JQ706061.1; K1a8b; Asia_W; 36; A73G; T152C; A263G	16569	Asia_W	K1a8b	K	K	36
4260	JQ706062.1	JQ706062.1; U2d1; Asia_S_W_Europe; 36; A73G; T152C; T199C	16569	Asia_S_W_Europe	U2d1	U	U2	36
4261	JQ706063.1	JQ706063.1; K1a19a; Asia_W; 40; T63C; C64T; A73G	16569	Asia_W	K1a19a	K	K	40
4262	JQ706064.1	JQ706064.1; K1a17a; Asia_W; 39; A73G; A200G; C242T	16569	Asia_W	K1a17a	K	K	39
4263	JQ706065.1	JQ706065.1; K1a12a1a; Asia_W; 37; A73G; T195C; A263G	16568	Asia_W	K1a12a1a	K	K	37
4264	JQ706066.1	JQ706066.1; K1a12a1a; Asia_W; 37; A73G; A263G; C497T	16568	Asia_W	K1a12a1a	K	K	37

Supplementary Table S4 Human mitochondrial database (hMITO DB v1.0) metadata[a]

Row	Name (accession no.)	Description	Size	Geo_Region	Haplogroup	Macro_ Haplo	Macro_ Haplo2	Total Variants
4265	JQ706067.1	JQ706067.1; U2d2; Asia_S_W_Europe; 39; A73G; T152C; T199C	16569	Asia_S_W_Europe	U2d2	U	U2	39
4266	DQ272116_M10	DQ272116_M10; M10; Asia_E_SE; 40; A73G; A263G; C315CC	16573	Asia_E_SE	M10	M	M10	40
4267	EF060330_M1a1	EF060330_M1a1; M1a1; Africa_E_Nile Valley; 42; A73G; T195C; A263G	16569	Africa_E_Nile Valley	M1a1	M	M1	42
4268	DQ112737_L1b1	DQ112737_L1b1; L1b1; Africa_C; 64; CA522d; G709A; T710C	15583	Africa_C	L1b1	L1	L1	64
4269	KF148510_M8a1	KF148510_M8a1; M8a1; Asia_C_E; 39; A73G; A234G; A263G	16568	Asia_C_E	M8a1	M	M8	39
4270	KC577355_R32	KC577355_R32; R32; Asia_S_SE; 29; A73G; T152C; A263G	16561	Asia_S_SE	R32	R	R32	29
4271	KC867130_N3	KC867130_N3; N3; Africa_NE_Europe_E; 36; A73G; T146C; T195C	16569	Africa_NE_Europe_E	N3	N	N3	36
4272	JX289118_N8	JX289118_N8; N8; Africa_NE_Asia_E; 34; A73G; T195C; A263G	16575	Africa_NE_Asia_E	N8	N	N8	34
4273	AY289059_O	AY289059_O; O; Asia_SE_Oceania; 44; C44CC; A73G; A263G	16575	Asia_SE_Oceania	O	O	O	44
4274	KC911472_HV	KC911472_HV; HV; Asia_W; 11; A263G; C315CC; A750G	16569	Asia_W	HV	HV	HV	11
4275	KC911379_R2b	KC911379_R2b; R2; Asia_S_SE; 32; A73G; T152C; A263G	16571	Asia_S_SE	R2	R	R2	32
4276	DQ408676_M3	DQ408676_M3; M3; Asia_S; 37; A73G; T195C; A263G	16568	Asia_S	M3	M	M3	37
4277	JN253391_D1	JN253391_D1; D1; Asia_E_America_N_S; 33; A73G; A263G; C315CC	16569	Asia_E_America_N_S	D1	D	D	33
4278	AP008373_M8a2b_Jap	AP008373_M8a2b_Jap; M8a2b; Asia_C_E; 38; A73G; T152C; A263G	16569	Asia_C_E	M8a2b	M	M8	38
4279	NC_012920_rCRS	NC_012920_rCRS;H2a2a1;Asia_W_Europe;0;REF;REF;REF	16569	Asia_W_Europe	H2a2a1	H	H	0
4280	FN673705_hs_Denisova_Altai	FN673705_hs_Denisova_Altai;H2a2;Asia_W_E;3;A263G;A8860G;A15326G	16570	Asia_W_E	Denisova	Denisova	Denisova	3
4281	NC_011137_hs_Neanderthal_re	NC_011137_hs_Neanderthal_refseq;L1'2'3'4'5'6;Asia_W_Europe ;44;A73G;T146	16565	Asia_W_Europe	Neanderthal	Neanderth	Neanderth	44
4282	MN849799_P	MN849799_P;P;Asia_SE_Oceania;12;A73G;A263G;A750G	16568	Asia_SE_Oceania	P	P	P	12
4283	AY956412_Q	AY956412_Q;Q2a3a;Asia_SE_Oceania;45;A73G;T152C;T195C	16572	Asia_SE_Oceania	Q2a3a	Q	Q	45
4284	AF346963_S1	AF346963_S1;S1;Asia_SE_Oceania;16;A73G;A263G;A750G	16569	Asia_SE_Oceania	S1	S	S1	16
4285	AY289051_S2	AY289051_S2;S2;Asia_SE_Oceania;17;A73G;A263G;A750G	16568	Asia_SE_Oceania	S2	S	S2	17
4286	EU007853_Y	EU007853_Y;Y1a+16189;Asia_NE;24;A73G;T146C;A263G	16570	Asia_NE	Y1a+16189	Y	Y	24

[a]See Material and Methods section of the manuscript for nomenclature used for the hMITO DB metadata.
