# Supplementary material for: A Customized Human Mitochondrial DNA Database (hMITO DB v1.0) for Rapid Sequence Analysis, Haplotyping and Geo-Mapping
Source: Int J Mol Sci. 2023 Aug 31;24(17):13505. doi: 10.3390/ijms241713505 (PMC10488239; doi:10.3390/ijms241713505)
Supplement: Supplementary file 1 [file ijms-24-13505-s001.zip › ijms-2550861-supplementary/Table S5.pdf]

Supplementary Table S5 Mitochondrial control region primers

Primer name[a]	Primer sequence[b]
N11_F15989-NxSeq-Rd1	CACTCTTTCCCTACACGACGCTCTTCCGATCTcccaaagctaagattctaat
N11_R16222-NxSeq-Rd2	CAGACGTGTGCTCTTCCGATCTggttgattgctgtacttgcttg
N13_F16347-NxSeq-Rd1	CACTCTTTCCCTACACGACGCTCTTCCGATCTtcaaatcccttctcgtccc
N13_R006-NxSeq-Rd2	CAGACGTGTGCTCTTCCGATCTgtgatccatcgtgatgtctt
N22_F140m-NxSeq-Rd1	CACTCTTTCCCTACACGACGCTCTTCCGATCTcctgccYcatYcYattattta
N22_R381-NxSeq-Rd2	CAGACGTGTGCTCTTCCGATCTgctggtgttagggttctttg
N12_F16159m-NxSeq-Rd1	CACTCTTTCCCTACACGACGCTCTTCCGATCTcataaaaacccaaYccacat
N12_R16400-NxSeq-Rd2	CAGACGTGTGCTCTTCCGATCTgtcaagggacccctatctga
N21_F16524-NxSeq-Rd1	CACTCTTTCCCTACACGACGCTCTTCCGATCTaagcctaaatagcccacacg
N21_R187-NxSeq-Rd2	CAGACGTGTGCTCTTCCGATCTcgcctgtaatattgaacgta
N30_F335g-NxSeq-Rd1	CACTCTTTCCCTACACGACGCTCTTCCGATCTaaacacatctctgccagacc
N30_R569m-NxSeq-Rd2	CAGACGTGTGCTCTTCCGATCTggtgtctttggggtttggtt

[a] N*n* is primer nomenclature of Lee et al. [18]. F*n*, forward primer; R*n*, reverse primer; *n* is 5' starting position on the mtDNA.
[b] Sequence data with NxSeq Universal forward or reverse sequence in upper case and mitochondrial sequence in lower case.
